# Pd-Catalyzed O‑Arylation of Phenols Mediated by a Weak, Soluble Organic Base: Methodology, Mechanism, and Compatibility with Enabling Technologies

**DOI:** 10.1021/jacs.5c13469

**Published:** 2025-09-26

**Authors:** Martyna I. Ostrowska, James A. Morris, Liam T. Ball

**Affiliations:** † School of Chemistry, University of Nottingham, Nottingham NG7 2RD, U.K.; ‡ Syngenta, Jealott’s Hill International Research Centre, Bracknell RG42 6EY, U.K.; § School of Chemistry, University of Bristol, Bristol BS8 1TS, U.K.

## Abstract

The low nucleophilicity of phenols represents a major obstacle to the Pd-catalyzed synthesis of diaryl ethers. This inherent challenge is typically exacerbated by the reliance of C–O couplings on an insoluble, inorganic base: the resulting reaction heterogeneity leads to irreproducibility across scales and between laboratories, and incompatibility with common enabling technologies. In this *Article*, we report the development and mechanistic interrogation of an homogeneous, Pd-catalyzed C–O coupling between phenols and aryl triflates that uses a weak, soluble tertiary amine as base. We show that the choice of ligand, aryl electrophile, and base is crucial if side reactions and catalyst poisoning are to be avoided, and productive catalysis achieved. Investigation of reaction kinetics and linear free energy relationships has revealed the identity of both the catalyst resting state and the turnover-limiting step. The combined mechanistic insight allows qualitative *a priori* prediction of reaction outcome as a function of substrate electronics, and provides an intuitive guide as to how diaryl ether targets should be disconnected. The resulting methodology exhibits broad substrate scope, with yields reproducible across both preparative ‘singleton’ scales and high throughput formats. The homogeneous nature of the couplings allows their safe execution under microwave heating, with elevated temperatures allowing for short reaction times even in the presence of air, and their facile translation to scale-up in continuous flow.

## Graphical abstract

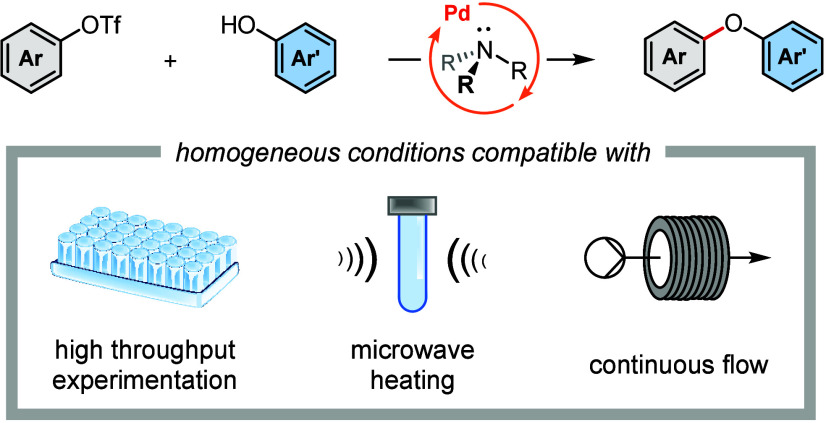

## Introduction

Diaryl ethers are an important substructure of numerous natural products,^1–5^ pharmaceuticals,^6,7^ and agrochemicals.^8–10^ Among the most powerful strategies^11^ for their synthesis are C–O cross-couplings catalyzed by Pd,^12–17^ Cu,^18–22^ and – increasingly – Ni^23,24^ (Scheme 1A). Although extant protocols employ a wide range of reaction conditions, the vast majority are united by use of an insoluble inorganic base that renders them heterogeneous. Mass transfer in these solid–liquid biphases is especially sensitive to (a) the efficiency of agitation, which demands careful reactor design particularly at plant-scale,^25^ and (b) the size and morphology of base particles,^26–28^ which may vary both between batches and over the course of a reaction.^29^ As a result, reproducibility issues are common when translating methods between laboratories or scales.^30^

**Scheme 1:**
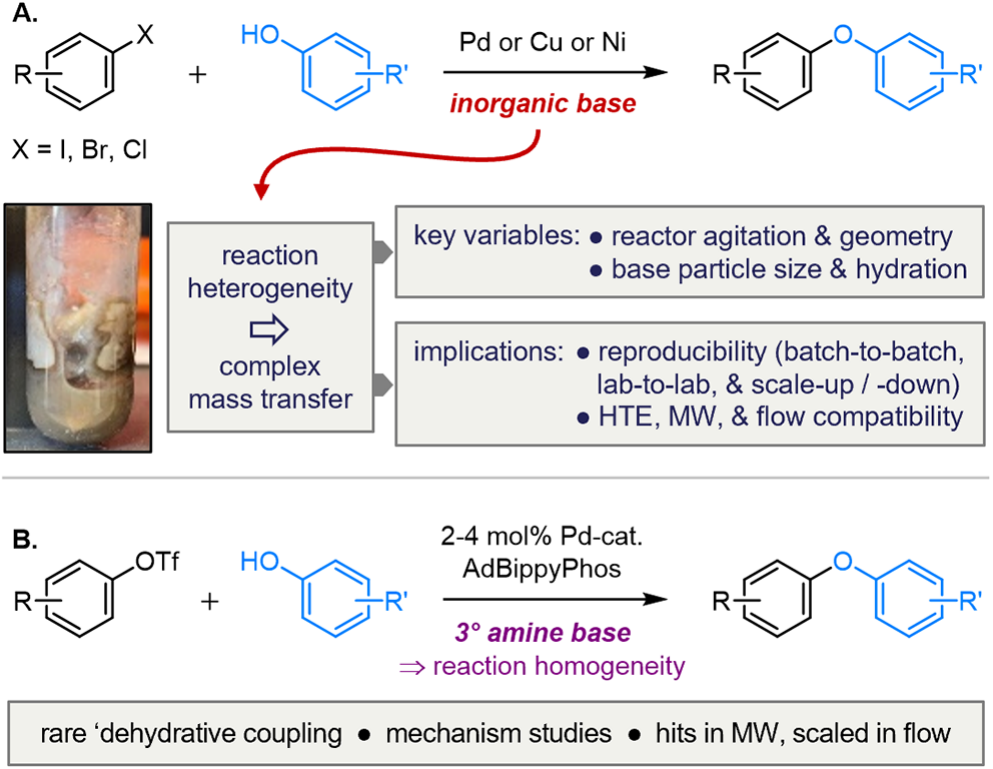
Catalytic O-Arylation of Phenols^a^

a Photograph in (A) shows a representative phenol O-arylation using K_3_PO_4_ in PhMe.

The negative impact of reaction heterogeneity on common enabling technologies is particularly acute. For example, the orbital shaking or tumble stirring used in high throughput experimentation (HTE) often fails to adequately mix heavy suspensions, resulting in misleading reactivity ‘trends’ that cannot be reproduced on preparative scales.^31^ Reactions heated by microwave irradiation are subject to considerations that extend beyond simple mass transfer: poor mixing also causes uneven exposure of the sample to the field, and hence establishment of temperature gradients within the reaction vessel.^32,33^ This can lead to (a) erroneous temperature measurements^32,33^ that impact reproducibility,^34–36^ and (b) localized superheating, which can result in thermal runaway and ultimately explosion.^37,38^ Translation from batch to flow is hindered by reactor clogging, prevention of which often requires bespoke technological interventions.^39^ Furthermore, reaction monitoring is challenging for any heterogeneous system,^40,41^ irrespective of scale or format. This is detrimental to the data rich experimentation that underpins rational reaction optimization, mechanism elucidation, and process control.^42–44^

A conceptually simple solution to these challenges is to use a soluble, organic base such that the resulting reaction mixture is homogeneous. Over the past decade, this concept has been demonstrated extensively for Pd,^45–53^ Cu^54,55^ and Ni^56–59^ catalyzed C–N couplings, and for Pd-catalyzed C–S couplings.^60^ In contrast, soluble base mediated C–O couplings are far less well explored, being limited to the arylation of aliphatic alcohols for Pd catalysis.^61^ Very recently, a method for the Ni-catalyzed synthesis of diaryl ethers has also been reported.^62^ Although this method represents a crucial proof of concept, it relies on precipitation of a halide salt to facilitate turnover – such that the reaction mixture is not truly homogeneous – and is limited to electron poor aryl halides and electron rich phenols.

There thus does not currently exist a general, homogeneous method for the C–O coupling of phenols with aryl electrophiles. Delivery of such a method would enable facile translation between laboratories and scales, would interface with enabling technologies, and would facilitate mechanistic studies. Herein we report realization of this objective through the development of an homogeneous, Pd-catalyzed coupling of phenols with aryl triflates that is promoted by a simple amine base (Scheme 1B). The method development is supported by experimental mechanistic studies that guide retrosynthesis and allow *a priori* prediction of reaction outcome. The coupling is shown to be compatible with automated dosing systems for parallel experimentation, microwave heating and flow synthesis.

## Results and Discussion

### Reaction Development

We initiated our studies by testing organic bases that spanned a range of both p*K*_aH_ values and steric profiles (Table 1). While tertiary amine bases did not promote the coupling of aryl bromide **1a** with phenol **2a** (entries 1–3, X = Br), low yields of diaryl ether **3** were obtained with stronger bases (entries 5–6, X = Br; representative p*K*_a(H)_ values in MeCN:^63–66^ PhOH, 29.2; DIPEA, 19.1; PMP, 20.3; BTMG, 23.6; BTPP, 28.4). In apparent contradiction to this trend, no coupling was observed in the presence of DBU (entry 4; p*K*_aH_ in MeCN = 24.3 ^65^); however, control reactions indicated that DBU acts as a catalyst poison (see Supporting Information (SI)), presumably through complexation to an intermediate oxidative addition complex. DBU adducts of oxidative addition complexes have been identified as off-cycle resting states during the N-arylation of anilines,^67–69^ and it is anticipated that displacement of DBU from Pd­(II) by a phenol would be significantly less facile than by an aniline.^70^ In contrast to aryl bromide **1a**, aryl triflate **1b** was coupled successfully in the presence of both tertiary amines and stronger bases (entries 1–6, X = OTf).

**Table 1:**
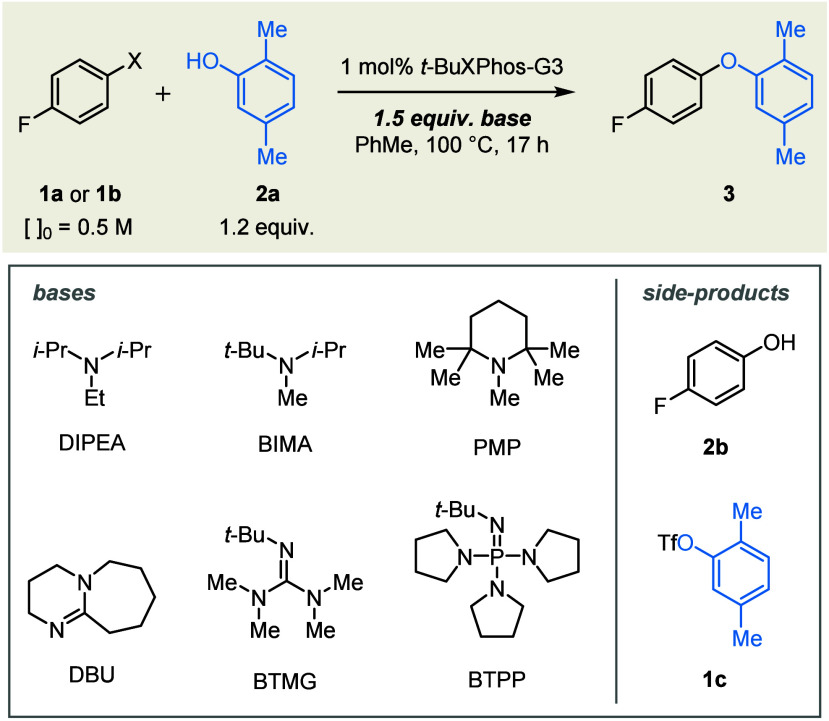
Assessment of Base as a Function of Aryl Electrophile^a^

		**1a** (X = Br)	**1b** (X = OTf)	
**entry**	**base**	% 3	% 3	% 2b, 1c
1	DIPEA	0	0	n.d.
2	BIMA	0	16	n.d.
3	PMP	0	24	n.d.
4	DBU	0	0	31, 32
5	BTMG	12	15	73, 70
6	BTPP	17	8	76, 77

a Yields determined by ^19^F NMR spectroscopic analysis vs internal standard. n.d. = not detected.

When performed in the presence of strong bases, the coupling of triflate **1b** was accompanied by competing transfer of the triflyl moiety to phenol **2a**, affording side-products **2b** and **1c** (Table 1, entries 4–6). Control reactions using BTPP as the base indicated that triflyl migration is appreciable at ambient temperature, and that it is not catalyzed by Pd. Triflyl metathesis between phenols has been reported as a general synthetic method in its own right,^71,72^ and related hydrolyses/alcoholyses of aryl triflates have been observed as side reactions in C–C cross-couplings mediated by inorganic bases.^73,74^ These competing processes presumably explain the dearth of precedent for couplings between aryl triflates and phenols.^16,75–77^

Considering the initial observations outlined above, we chose to investigate further the weak-base-mediated coupling of aryl triflates with phenols. In addition to furnishing an homogeneous reaction mixture, this system benefits from (1) the use of phenols as progenitors to *both* partners, such that the coupling formally represents a rare^16,75–79^ dehydrative approach to diaryl ethers; and (2) the use of a weak (tertiary amine) base, which has the potential to confer compatibility with sensitive motifs that are typically not well tolerated in cross-coupling.^80–85^

To probe the interdependence of the key reaction variables, an array of ligands was screened in combination with three sterically different tertiary amine bases (Scheme 2). Bidentate (**L1**–**L5**) and simple monodentate (**L6**–**L10**) phosphines—including CyPF-*t*Bu (**L5**),^86^ triadamantylphosphine (**L7**),^87^ and MorDalphos (**L9**),^88^ all of which have been employed previously in related reactions^14,53,89^ —failed to facilitate the coupling. *ortho*-Biarylphosphine ligands^90–92^ proved generally more competent (**L11**–**L28**), consistent with precedent set by inorganic-base-mediated phenol O-arylations.^12,16,13,93^ The following general trends emerged: (1) for a given biaryl scaffold, the dicyclohexylphosphine analog afforded lower yields than did the corresponding di-*tert*-butyl or di-1-adamantyl congeners (compare **L13** with **L14** and **L15**, **L16** with **L17**, **L21** with **L22** and **L23**, and **L24** with **L25** and **L26**); and (2) for a given ligand, yield increased with the bulk of the tertiary amine base (DIPEA < BIMA < PMP).^94,95^ Presumably the use of a sterically demanding phosphine ligand both accelerates reductive elimination – which is typically rate-determining for phenol O-arylations^12,16,96,97,17^ – and shields the metal from complexation by the base,^98,52,99^ an effect that would be amplified when the latter is hindered.

**Scheme 2:**
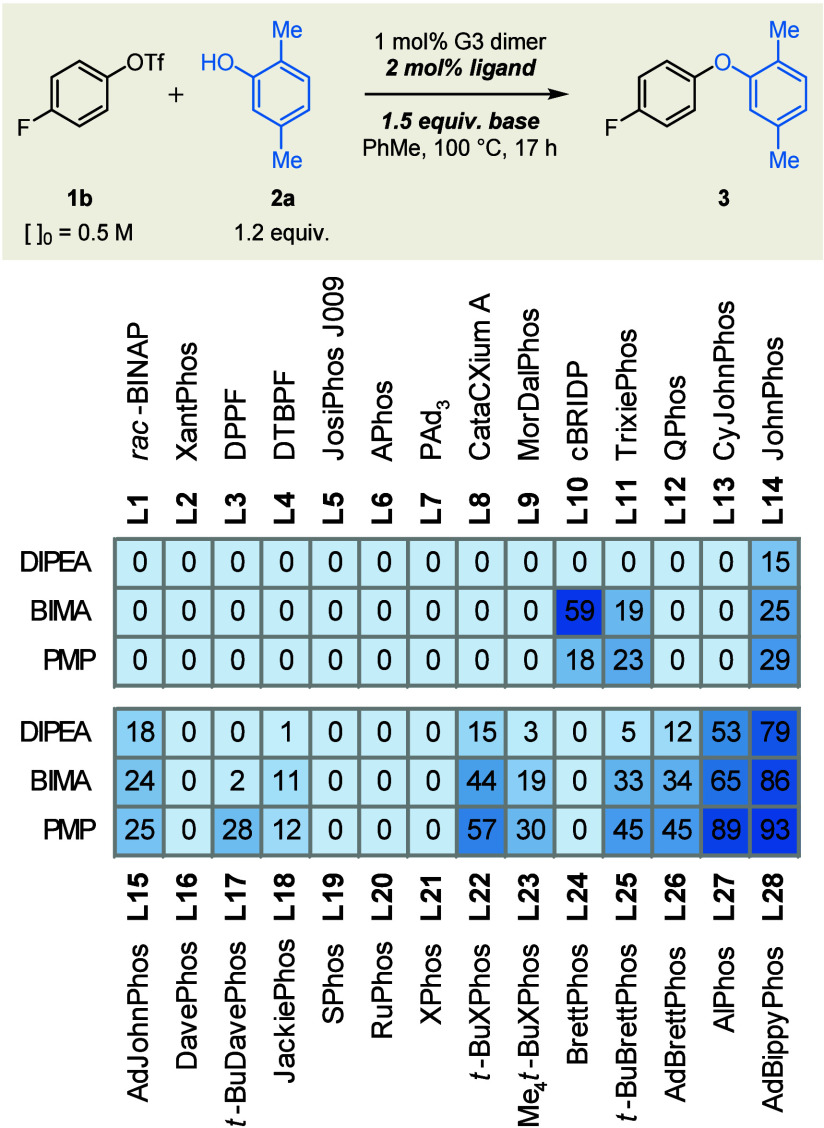
Assessment of Ligand as a Function of Base^a^

a Yields of diaryl ether **3** determined by ^19^F NMR spectroscopic analysis vs internal standard; ligand structures shown in the SI.

The combination of AdBippyPhos (**L28**)^92,100^ and PMP proved most efficient, and—with the following modifications—was therefore used in subsequent investigations: (1) a 2:1 ligand:metal ratio was used, as the higher stoichiometry was found to improve reaction reproducibility, and (2) Buchwald’s G3 palladacycle precatalyst^101^ was replaced with the G4 precatalyst^102^ to prevent formation of N-arylated carbazole side-products which were observed during reaction optimization. As per the ligand screening campaign (Scheme 2), an admixture of the precatalyst and the ligand was used because *tert*-alkyl BippyPhos ligands do not form isolable complexes with Buchwald’s palladacycles.^103^

### Investigation of Reaction Mechanism

Before studying the reaction scope, we sought to understand more fully the salient mechanistic features of the C–O coupling system outlined above.

First, the contrasting compatibility of different bases observed in the coupling of bromide **1a** and of triflate **1b** (Table 1) hint at a mechanistic disparity in the formation of key palladium aryloxide intermediate **III** (Figure 1A). In the case of aryl bromides, the requirement for a strong base suggests that the phenol must be deprotonated before its coordination to oxidative addition complex **IIa** (pathway *i*); the alternative scenario—in which a neutral phenol coordinates to **IIa** before deprotonation (pathway *ii*)—is deemed unlikely due to the poor Lewis basicity of phenols^104^ and the low propensity of the Pd–Br bond to dissociate in apolar solvents.^67^ In contrast, the enhanced electrophilicity of oxidative addition complex **IIb** presumably allows coordination of the neutral phenol (pathway *ii*; this is expected to result in significant acidification (by up to 10 p*K*_a_ units),^105–107^ such that even weak bases will be able to mediate formation of palladium aryloxide **III**. The significance of a Lewis acidic Pd­(II) intermediate in the coupling of aryl triflates is underlined by the inhibitory effect of added bromide salt (see SI).

**Figure 1:**
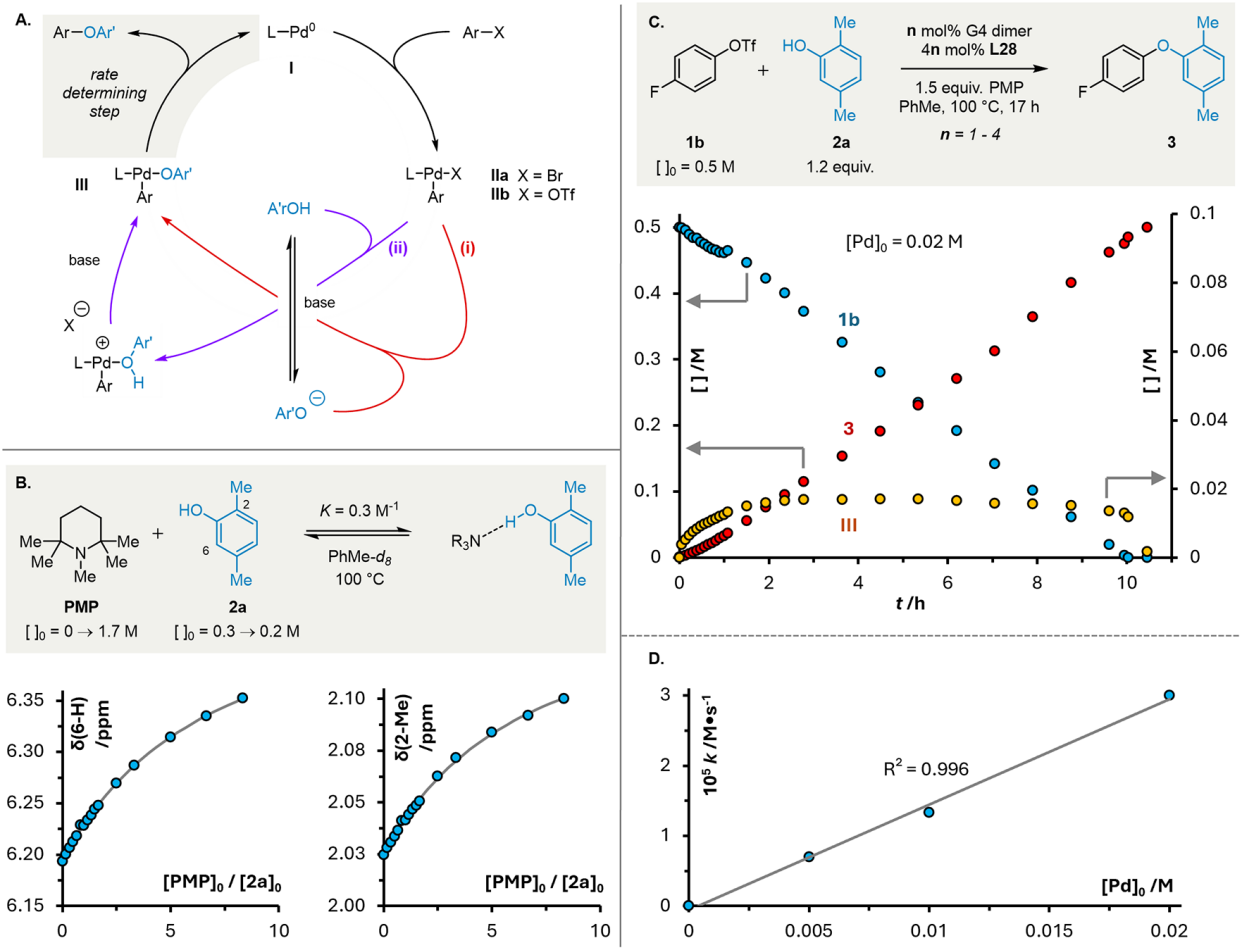
Putative catalytic cycle with limiting transmetalation pathways (A). Titration of phenol **2a** with PMP (B); solid lines represent the calculated 1:1 binding isotherm with *K*_assoc_ = 0.3 M^–1^. Representative reaction profile for C–O cross-coupling of triflate **1b** and phenol **2a** (C). Dependence of reaction rate on initial concentration of Pd (D).

Further insight into the formation of palladium aryloxide **III** was obtained by titration of phenol **2a** with PMP at 373 K (Figure 1B). Changes in the ^1^H NMR chemical shifts of both the 2-Me and 6-H protons of **2a** fit well to a 1:1 binding isotherm with *K*_assoc_ = 0.30 M^–1^, consistent with formation of a hydrogen-bonded complex.^108–110^ No significant self-association of phenol **2a** was observed in dilution experiments (see SI). The weakness of the phenol-PMP association indicates that only a small proportion of the phenol is ever involved in the H-bonded complex (calculated as 16% → 1% over the course of the reaction; see SI). Thus, while PMP does not deprotonate the phenol before its coordination to Pd­(II), it is plausible that one or both of two alternative transmetalation pathways operate. First, proton abstraction may accompany coordination of the phenol-PMP complex as a single entity; and second, coordination of the discrete phenol may be followed by a subsequent, bimolecular deprotonation event (*Cf*. pathway *ii*, Figure 1A).

Monitoring the coupling of triflate **1b** and phenol **2a** by *in situ* ^19^F NMR spectroscopy revealed a *pseudo* zeroth order kinetic profile (Figure 1C) and a first-order dependence on the concentration of Pd (Figure 1D). The observed catalyst resting state (δ_F_ = −122 ppm) was identified as arylpalladium phenoxide **III** by sequential addition of phenol **2a** and PMP to an independently prepared sample of oxidative addition complex **IIb** (see SI). Together, these observations are consistent with reductive elimination being turnover-limiting, as has been reported for C–O couplings mediated by inorganic bases.^12,16,96,97,17^

Hammett plots constructed from the absolute rate of reaction gave good correlations against standard σ values for both the triflate (ρ_ArOTf_ = +1.75; Figure 2A) and the phenol (ρ_Ar′OH_ = −2.21; Figure 2B). The sign and magnitude of the observed reaction constants are consistent with the proposed identity of the rate-determining step,^97,96^ and are similar to those reported previously for cross-couplings of anilines (N-arylation: ρ_ArX_ = +2.2, ρ_aniline_ = −1.1;^111^ N-alkylation: ρ_aniline_ = −1.96 ^112^).

**Figure 2:**
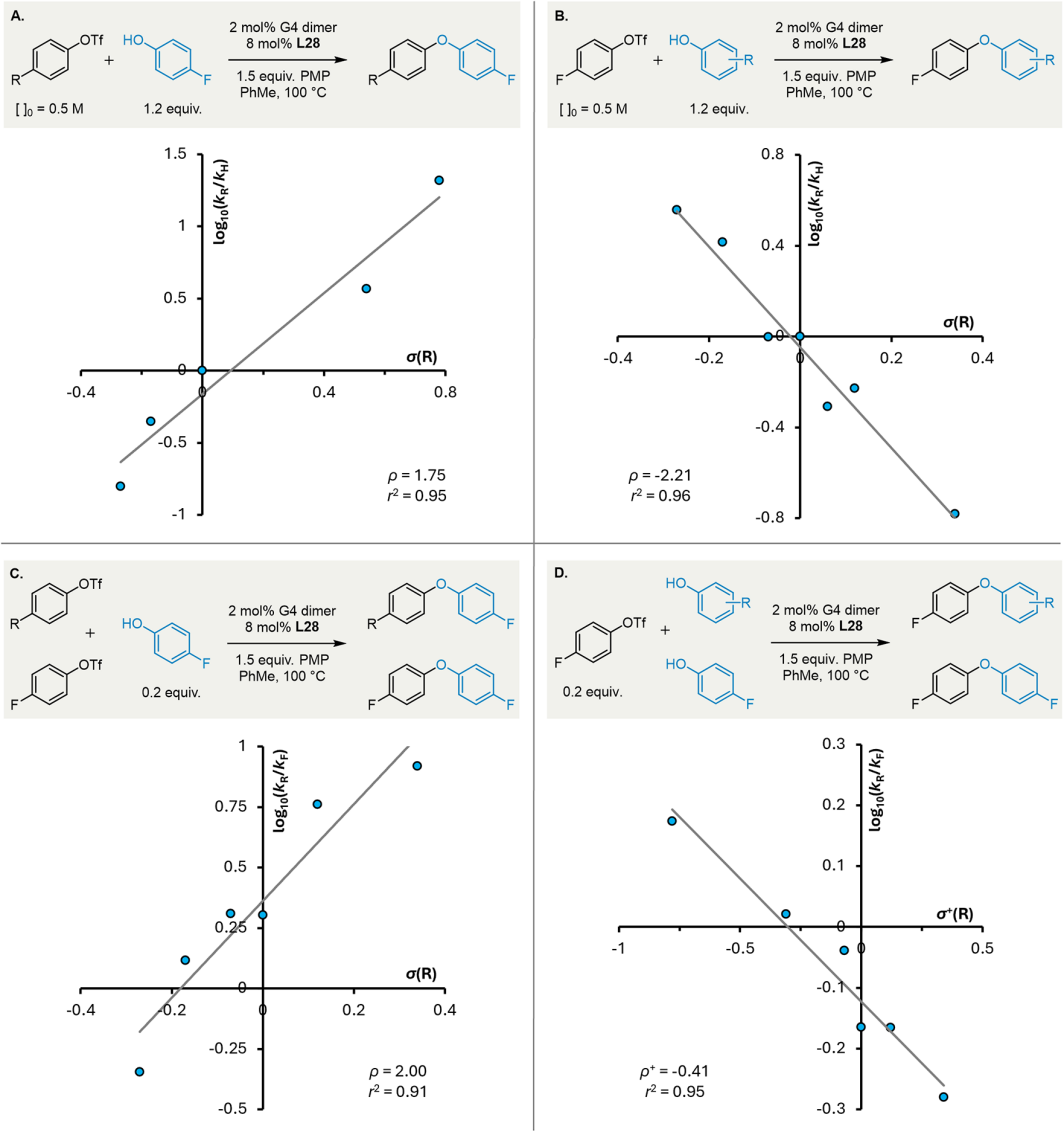
Hammett plots constructed using absolute rate measurements (A, B), and by pairwise intermolecular competitions (C, D).

A Hammett plot derived from intermolecular competition between pairs of aryl triflates gave a positive correlation against σ (Figure 2C), as anticipated for selectivity determining oxidative addition.^113^ The magnitude of the reaction constant (ρ = +2.00) is comparable with literature values for oxidative addition into both aryl iodides (ρ_ArI_(PhMe) = +2.3;^114^ ρ_ArI_(THF) = +2.0 ^115^) and aryl triflates (ρ_ArOTf_ = +2.55).^116^ In contrast, intermolecular competition between pairs of phenols gives a small, negative reaction constant (ρ^+^ = −0.41; Figure 2D). The disparity between this value and that calculated from absolute rate measurements (Figure 2B) indicates that the competition reaction is not under strict Curtin–Hammett control.

Together, this initial mechanistic study indicates that (1) the oxidative addition complex **II** must be weakly coordinated by its anion for transmetalation to proceed to **III**, such that the reaction is not compatible with aryl halide substrates and is likely to be sensitive to Lewis basic additives; and (2) when aryl triflate substrates are employed, palladium­(II) aryloxide **III** constitutes the resting state and the coupling is therefore likely to be sensitive to the electronic properties of both coupling partners (Figure 1A).

### Reaction Scope

As anticipated from the foregoing mechanistic studies (Figure 2), the reaction tolerates a wide range of aryl triflates (Scheme 3A), with good to excellent yields obtained for substrates featuring electron donating (**4**, **5**), withdrawing (**6**-**11**), or *ortho* (**12**-**13**) substituents. Notably, triflates derived from Trolox (**13**), tryptamine (**14**), and 2- or 4-pyridones (**15**-**17**) coupled smoothly. The mildness of the base employed confers compatibility with potentially sensitive functional groups – including esters, nitriles, and 2- and 4-fluoropyridines – and in no case was triflate metathesis observed. Control reactions indicated that Pd catalysis, rather than an uncatalyzed S_N_Ar process, was responsible for the observed diaryl etherification.

**Scheme 3:**
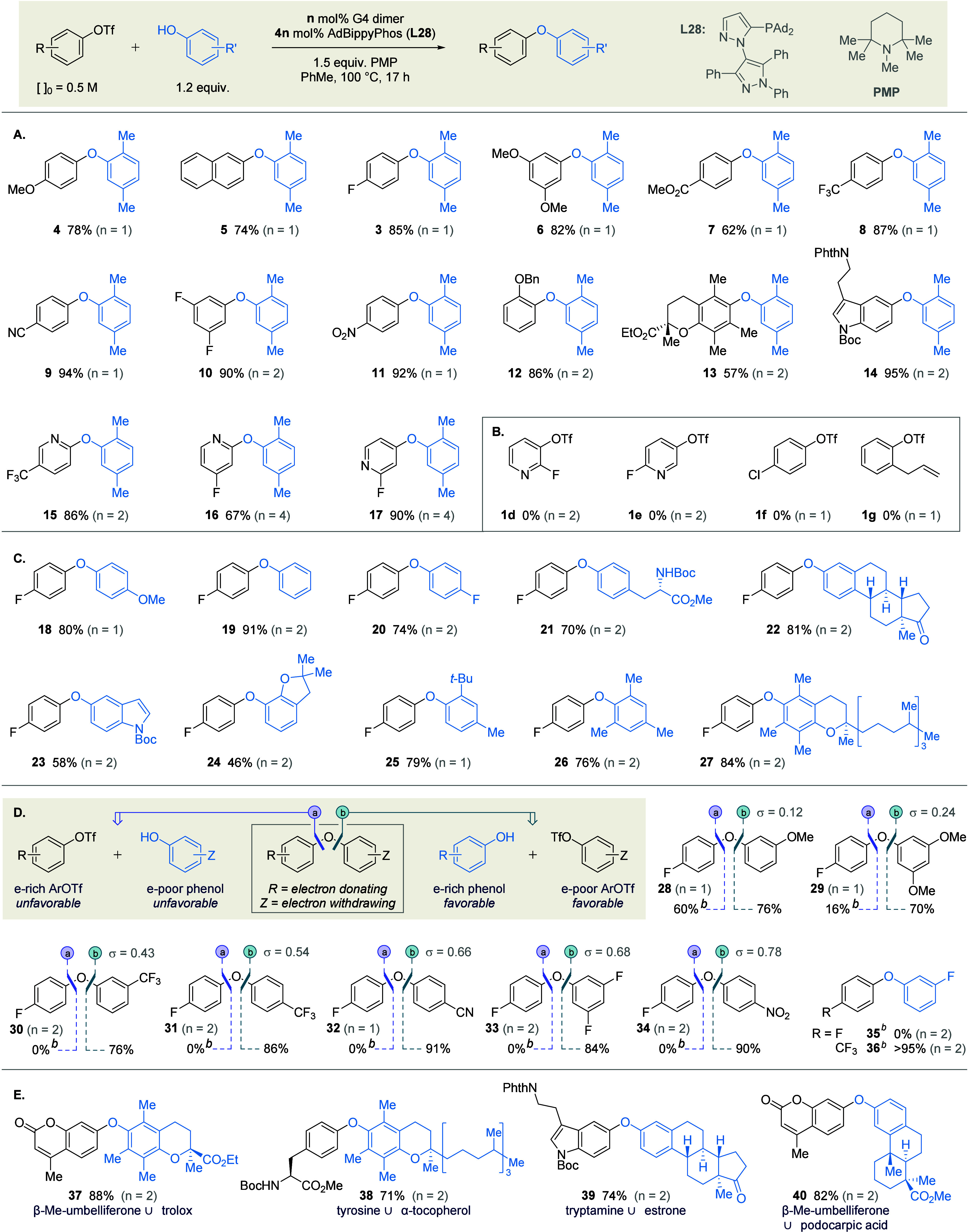
Scope and Limitations of the C–O Cross-Coupling (A–D), and Conjugation of Phenolic Natural Products^a^

a Yields refer to material isolated after purification.
b Yield determined by ^19^F NMR spectroscopic analysis vs internal standard.

Limitations of the methodology with respect to the triflate partner are shown in Scheme 3B. In contrast to the successful synthesis of fluoropyridyl ethers **16** and **17** (Scheme 3A), couplings of 2-fluoropyridines bearing triflates at the 3- or 5-positions (**1d**, **1e**) were accompanied by uncatalyzed displacement of fluoride. This divergent reactivity is consistent with the relatively slower oxidative addition of 3-(*pseudo*)­halopyridines to Pd(0),^117,118^ such that S_N_Ar of the activated fluoride (σ_m_(OTf) = 0.56; σ_p_(OTf) = 0.53)^119^ presumably becomes competitive. Attempted coupling of chlorophenyl triflate **1f** did not afford observable levels of any aryl ether products; we hypothesize that oxidative addition into the C–Cl bond competes with oxidative addition into the C-OTf bond, resulting in an arylpalladium chloride complex that is inert to transmetalation (*Cf*. couplings of aryl bromide **1a**, Table 1). Finally, while triflate **1g** was coupled quantitatively, the etherification was accompanied by double bond migration to give an intractable 1:1:1 mixture of the allyl, (*E*)-styryl, and (*Z*)-styryl isomers.

The coupling of electron-rich and -neutral phenols (**18**–**23**) proved facile (Scheme 3C), with similarly good yields obtained in the presence of (di)*ortho*-substitution (**24**–**27**). Although attempts to prepare tetra-*ortho*-substituted diaryl ethers proved unsuccessful (see SI), we are not aware of any Pd-catalyzed syntheses of this motif.

In contrast, the etherification of electron-poor phenols with electron neutral triflate **1b** proved challenging (**28**–**34**; Scheme 3D, *disconnection a*), resulting in negligible conversion for phenols with cumulative Hammett σ values ≥ 0.24. This observation is consistent with the mechanistic studies outlined above (Figure 2B), and can be used to guide effective deployment of the methodology in synthesis: because both partners are derived from phenols, the disconnection of either C–O bond is equally practical; it therefore falls to the chemist to simply select the most electronically favorable disconnection, and hence which phenol to convert into the triflate partner. To demonstrate the value of this strategic flexibility, we thus prepared the same diaryl ethers **28**–**34** according to *disconnection b*. This disconnection employs the more electron rich aryl moiety as the phenol, and the less electron rich component as the triflate, such that the electronic preferences are optimized with respect to *both* partners, and the couplings proceed in excellent yields.

Furthermore, the opposing ρ values obtained for the triflate and the phenol Hammett plots (Figure 2A, 2B) suggest that disfavorable electronics in one reaction component can be offset by highly favorable electronics in the other. For example, while 3-fluorophenol (σ_m_(F) = +0.34) does not couple with electron neutral triflate **1b** (σ_p_(F) = +0.06) to give diaryl ether **35** (Scheme 3D), the same phenol couples efficiently with an electron poor triflate (σ_p_(CF_3_) = 0.54) to give the corresponding ether **36** in excellent yield. This is consistent with empirical observations made independently by Tudge^120^ and by Stradiotto,^23,62^ who respectively reported the Pd- and Ni-catalyzed couplings of electron poor phenols with very electron poor (hetero)­aryl chlorides.

As the first general phenol etherification to use aryl triflates,^16,75–77^ our methodology significantly expands the potential pool of aryl electrophiles beyond that defined by aryl halides. This particular benefit is showcased in the formal dehydrative coupling between complex (naturally derived) phenols, where the corresponding halide is not necessarily available or is prohibitively expensive. Hence it is now possible to rapidly assemble complex diaryl ethers such as **37**–**40** by conjugation of widely available phenols (Scheme 3E).

### Compatibility with Enabling Technologies

The key motivation for this study has been to develop a phenol etherification that—by virtue of its homogeneity—can integrate directly with existing enabling technologies.^121^ We therefore sought to demonstrate its compatibility with (a) HTE and MW synthesis, which are established mainstays of contemporary discovery chemistry,^122–125^ and (b) continuous flow, which is increasingly employed not only for reaction scale-up, but also for optimization and parallel synthesis.^126,127^

To this end, automated dosing of plate-format reactions in a glovebox gave comparable yields to singleton reactions assembled manually and performed using standard Schlenk technique (Scheme 4A). This therefore demonstrates compatibility with reaction miniaturization, and should give users confidence that false-positives/negatives arising from mixing phenomena will be avoided.

**Scheme 4:**
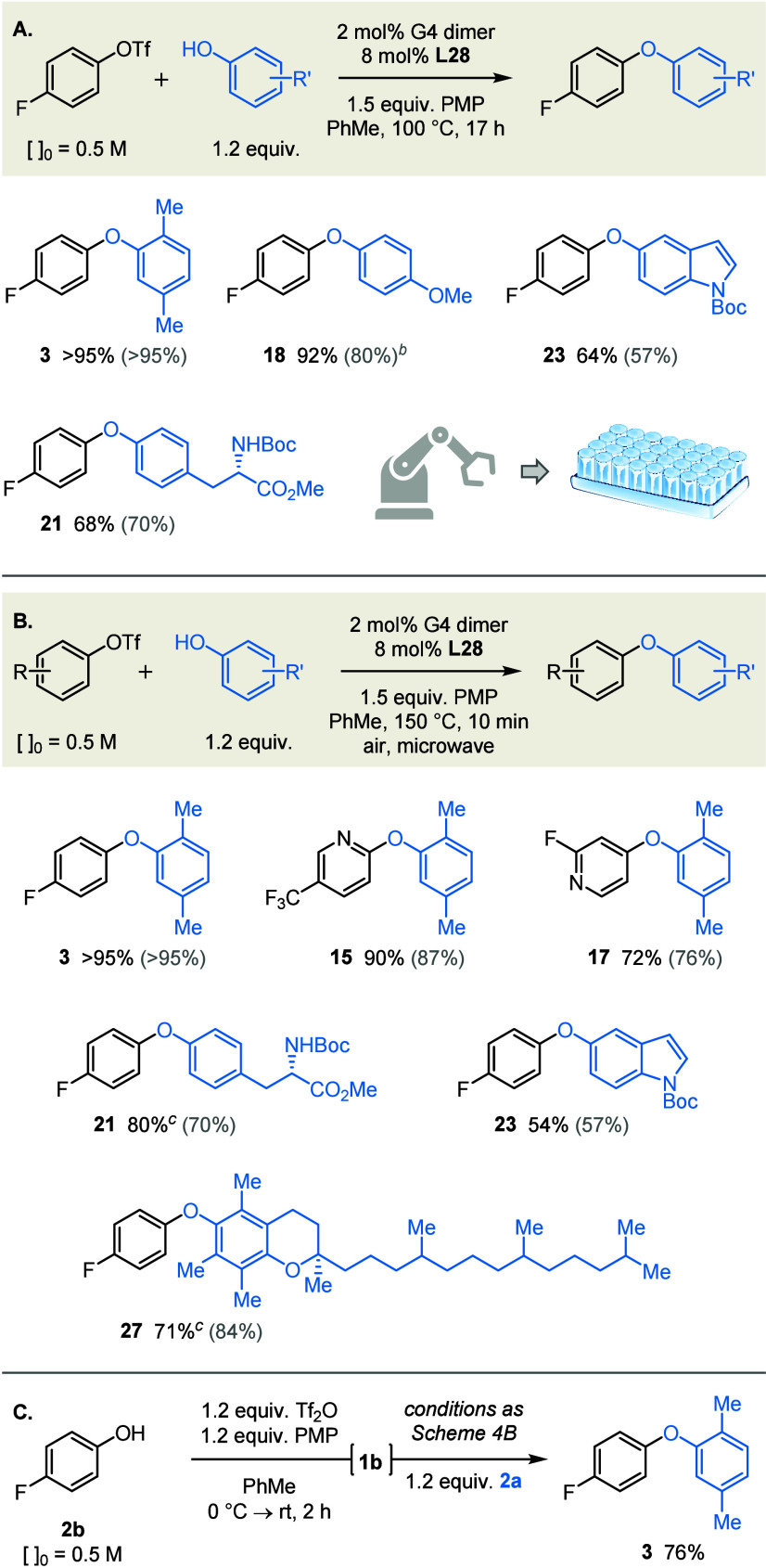
Compatibility with Automated Dosing (A) and Microwave Heating (B); Telescoped Phenol Triflation/Cross-Coupling (C)^a^

a Yields determined by ^19^F NMR spectroscopic analysis vs internal standard; values in parentheses refer to singleton reactions performed at 100 °C (conventional heating) for 17 h, as illustrated in Scheme 3.
b Reaction performed using 1 mol % G4 dimer and 4 mol % **L28**.
c Reaction performed for 1 h.

The ability to perform the couplings safely and reliably in a microwave reactor provides an opportunity to increase reaction rate simply by raising the temperature. Indeed, we found that the coupling of triflate **1b** and phenol **2a** was complete in under 10 min at 150 °C (Scheme 4B; see SI for optimization). Moreover, the reaction can be performed under an aerobic atmosphere without detriment, whereas the same coupling performed at 100 °C required rigorous exclusion of oxygen. Gratifyingly, these conditions also proved applicable to the coupling of more heavily functionalized partners that incorporate reactive fluoropyridine or thermally labile Boc^128^ moieties (**17**, **21**, **23**).

Given the convenience of performing couplings under air, we sought to develop a telescoped procedure for the synthesis and subsequent coupling of the aryl triflate partner. We anticipate that, by avoiding the need to isolate and purify the triflate, this approach will expedite the uptake of our methodology in discovery settings. As illustrated in Scheme 4C, the reaction of triflic anhydride and phenol **2b** in the presence of PMP gave complete conversion to triflate **1b** in under 2 h. Subsequent addition of phenol **2a** and the precatalyst/ligand, then heating to 150 °C, afforded the corresponding diaryl ether in good yield.

Finally, the “MW-to-flow paradigm”^129^ was used to expedite translation of our methodology to a continuous manufacturing regime. The industrially relevant target, ACCase inhibitor fluazifop **41** (Table 2),^130^ was disconnected according to the logic outlined previously. In batch, the desired coupling proceeded in excellent yields under both MW heating (entry 1), and – by changing the solvent to higher boiling anisole – conventional thermal heating (entry 2). Control reactions indicated that the etherification is Pd-catalyzed, and that there is not an appreciable background S_N_Ar reaction (entry 3).

**Table 2:**
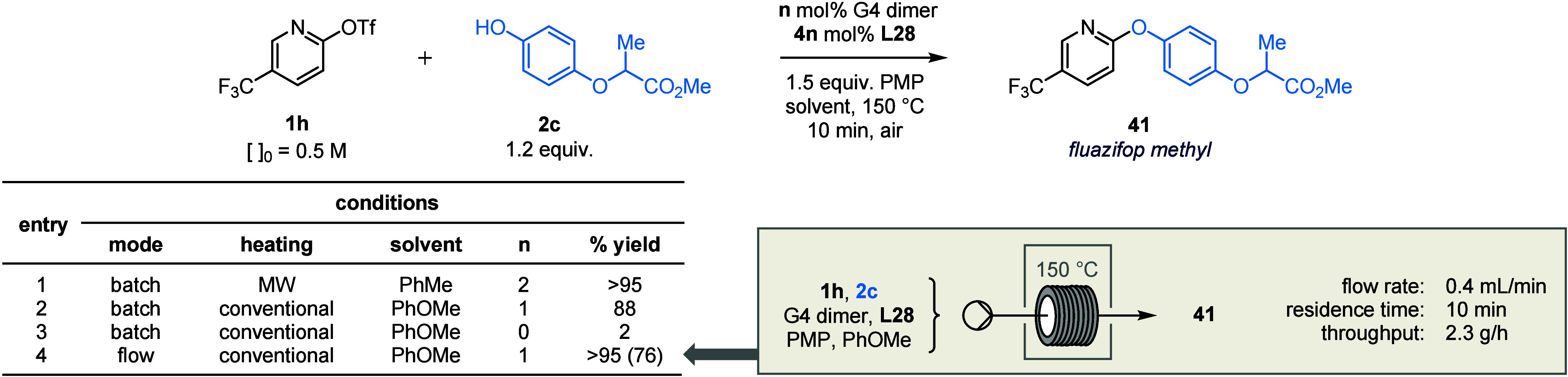
Synthesis of Fluazifop Methyl and Scale-up in Continuous Flow^a^

a Yields determined by ^19^F NMR spectroscopic analysis vs internal standard; yields in parentheses refer to material isolated after purification.

The coupling could be translated directly to flow without further optimization. Under the conditions shown in entry 4, > 1 g of fluazifop **41** (76% isolated yield) was obtained after less than 30 min of operation, corresponding to a throughput of 53 g/day. Notably, the experimental setup is extremely simple: all reagents are loaded into a single syringe under air, then are passed through a heated coil consisting of standard PTFE tubing immersed in a silicone oil bath (see SI). By eliminating the need for mixing tees, backpressure regulators or advanced pumps, this protocol is immediately amenable to laboratories with no prior experience of flow chemistry.

## Conclusion

In summary, we have reported the Pd-catalyzed cross-coupling of phenols and aryl triflates, which is both the first example of a general, homogeneous phenol O-arylation, and the first C–O coupling to employ aryl triflates as the electrophilic partner. The application of aryl triflates in this methodology both *enables* the use of a weak tertiary amine as base, and *mandates* that a weak base is used in order to avoid triflyl methathesis side-reactions. Mechanistic studies indicate that reductive elimination is turnover-limiting, and that the observed reaction rate is sensitive to the electronic properties of both coupling partners. This is borne out in substrate scope, where (a) the poor performance of electron-poor phenols can be offset by the retrosynthetic flexibility that results from coupling two phenol-derived partners, and (b) we demonstrate for the first time that it is the relative electronics of the coupling partners, and not the absolute electronic character of the phenol, that determines reaction outcome. The mild conditions of this coupling confer compatibility with sensitive functional groups—including pyridyl fluorides—and its homogeneity confers compatibility with automated dosing, microwave heating, and flow. Notably, by heating at 150 °C, reactions are complete in minutes and do not require an anaerobic atmosphere. This allows facile translation to flow, and progression from initial mg-scale to multi-g-scale synthesis in a matter of hours. We anticipate that this methodology will provide synthesis chemists with confidence in the scalability and translatability of a traditionally challenging cross-coupling.

## Supplementary Material



## Notes

Optimization data, experimental procedures, compound characterization data, reaction kinetics data, and NMR spectra (PDF)
