# Supplementary material for: Pd-Catalyzed O‑Arylation of Phenols Mediated by a Weak, Soluble Organic Base: Methodology, Mechanism, and Compatibility with Enabling Technologies
Source: J Am Chem Soc. 2025 Sep 26;147(40):36870–81. doi: 10.1021/jacs.5c13469 (PMC12512195; doi:10.1021/jacs.5c13469)

SUPPORTING INFORMATION

Pd-Catalyzed O-Arylation of Phenols Mediated by a Weak, Soluble Organic Base: Methodology, Mechanism, and Compatibility with Enabling Technologies

Martyna I. Ostrowska,[†] James A. Morris,[‡] and Liam T. Ball[†,¶,*]

e-mail: liam.ball@nottingham.ac.uk and liam.ball@bristol.ac.uk

orcid: L.T.B., 0000-0003-3849-9006; M.I.O., 0009-0005-2405-7196.

[†] School of Chemistry, University of Nottingham, Nottingham NG7 2RD, U.K.

[‡] Syngenta, Jealott's Hill International Research Centre, Bracknell RG42 6EY, U.K.

[¶] School of Chemistry, University of Bristol, Bristol BS8 1TS, U.K.

Table of Contents

1. General Information ... 3

1.1 Ligand Structures ... 4

2. Preparation of Starting Materials ... 5

2.1 Synthesis Procedures & Characterization Data for Aryl Triflates ... 5

2.2 Synthesis Procedures & Characterization Data for Phenols ... 34

3. Reaction Development ... 36

3.1 Base / Electrophile Screening (Manuscript Table 1) ... 36

3.2 Investigation into Catalyst Poisoning ... 37

3.3 Investigation into Triflyl Migration ... 39

3.4 Ligand Screening (Manuscript Scheme 2) ... 42

4. Mechanistic Investigations ... 43

4.1 Phenol / PMP Titration (Manuscript Fig. 1, Panel B) ... 43

4.2 Reaction Rate Law (Manuscript Fig. 1, Panel C) ... 49

4.3 Absolute Rates *vs* Aryl Triflate (Manuscript Fig. 2, Panel A) ... 56

4.4 Absolute Rates *vs* Phenol (Manuscript Fig. 2, Panel B) ... 58

4.5 Competition Between Aryl Triflates (Manuscript Fig. 2, Panel C) ... 60

4.6 Competition Between Phenols (Manuscript Fig. 2, Panel D) ... 61

5. Substrate Scope ... 63

5.1 Substrate Scope (Manuscript Scheme 3) ... 63

5.2 Attempted Synthesis of Tetra-*ortho*-Substituted Diaryl Ethers ... 104

6. Compatibility with Enabling Technologies ... 105

6.1 Automated Dosing (Manuscript Scheme 4, Panel A) ... 105

6.2 Microwave (MW) Heating (Manuscript Scheme 4, Panel B) ... 106

6.3 Telescoped Triflation / Coupling (Manuscript Scheme 4, Panel C) ... 107

6.4 Translation to Flow (Manuscript Table 2) ... 108

7. References ... 110

8. NMR Spectra ... 114

1. General Information

Procedures employing oxygen- and/or moisture-sensitive materials were performed with anhydrous solvents (*vide infra*) using standard inert-atmosphere techniques (atmosphere of anhydrous dinitrogen). Analytical thin-layer chromatography was performed on precoated aluminium-backed plates (Silica Gel 60 F254; Merck) and visualized using a combination of UV light (254 nm), and aqueous basic potassium permanganate stain. Automated flash column chromatography was performed on disposable columns pre-packed with 50 µm irregular silica gel (Interchim Puriflash or Büchi Flashpure Ecoflex) using a Büchi C-850 or Büchi C-815 equipped with a UV-vis DAD (200-800 nm) and an ELSD; manual flash column chromatography was performed using Sigma-Aldrich Supelco 60 Å silica gel (230 - 400 mesh).

NMR spectra were recorded at 25 °C on a Bruker Avance 500 or 400 spectrometer (^{1}H, 500 / 400 MHz, ^{13}C{^{1}H}, 126 / 101 MHz; ^{19}F NMR 376 MHz, ^{31}P 202 MHz). Chemical shifts are reported in ppm; coupling constants, *J*, are reported in Hz and are uncorrected for digitization. The following abbreviations (and their combinations) are used to label the multiplicities: s (singlet), d (doublet), t (triplet), q (quartet), p (pentet), hept (heptet), m (multiplet), br. (broad) and app. (apparent). ^{1}H and ^{13}C{^{1}H} chemical shifts are reported relative to tetramethylsilane, and are referenced to the appropriate residual solvent peaks:

- $CDCl_3$: δ_H = 7.26 ppm, δ_C = 77.16 ppm
- $(CD_3)_2SO$: δ_H = 2.50 ppm, δ_C = 39.52 ppm
- CD_3CN: δ_H = 1.94 ppm, δ_C = 118.26 ppm
- C_6D_6: δ_H = 7.16 ppm, δ_C = 128.06 ppm
- CD_2Cl_2: δ_H = 5.32 ppm, δ_C = 53.84 ppm

^{19}F chemical shifts are reported relative to $CFCl_3$. ^{31}P chemical shifts are reported relative to 85% aqueous H_3PO_4.

Infrared spectra of neat compounds were recorded over the range 4000-500 cm^{-1} using either a PerkinElmerSpectrum 1000 Series FTIR spectrometer with an ATR diamond cell, or a Bruker Alpha FTIR spectrometer fitted with a Bruker Platinum ATR Quicksnap™ diamond cell.

Melting points were measured using Stuart SMP10 or Gallenkamp melting point apparatus in open capillaries.

High resolution electron ionization (EI) mass spectra were obtained using a JEOL AccuTOF GCX. High resolution electrospray ionization (ESI) mass spectra were recorded using a Bruker ESI-TOF MicroTOF II spectrometer.

Reagent grade solvents (Fisher Technical) were employed. Toluene and THF were dried using an Inert PureSolv Grubbs-type system (alumina columns, argon atmosphere). Unless stated otherwise, all other reagents were used as received from commercial sources.

1.1 Ligand Structures

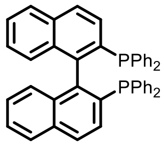

rac-BINAP (**L1**)

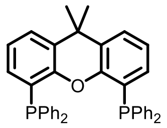

XantPhos (**L2**)

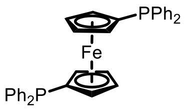

DPPF (**L3**)

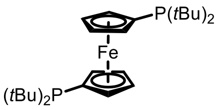

DTBPF (**L4**)

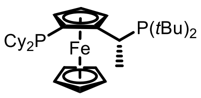

JosiPhos J009 (**L5**)

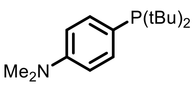

APhos (**L6**)

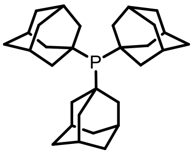

PAd$_3$ (**L7**)

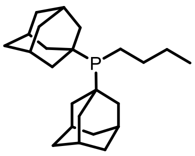

CataCXium A (**L8**)

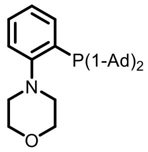

MorDalPhos (**L9**)

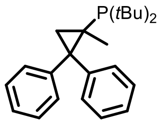

cBRDIP (**L10**)

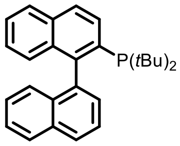

TrixiePhos (**L11**)

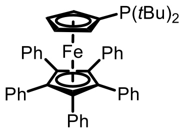

QPhos (**L12**)

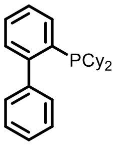

CyJohnPhos (**L13**)

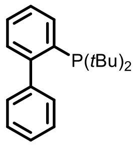

JohnPhos (**L14**)

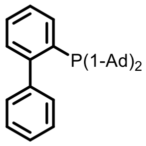

AdJohnPhos (**L15**)

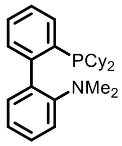

DavePhos (**L16**)

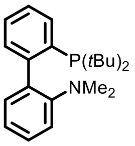

*t*BuDavePhos (**L17**)

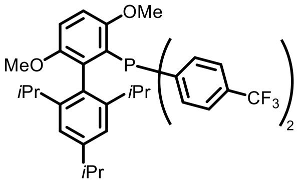

JackiePhos (**L18**)

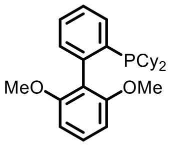

SPhos (**L19**)

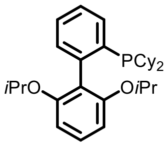

RuPhos (**L20**)

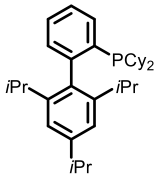

XPhos (**L21**)

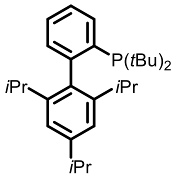

*t*BuPhos (**L22**)

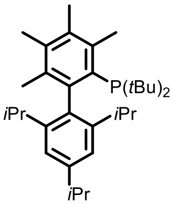

Me$_4$*t*BuPhos (**L23**)

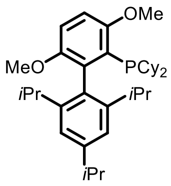

BrettPhos (**L24**)

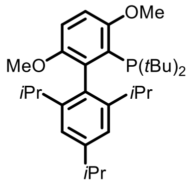

*t*BuBrettPhos (**L25**)

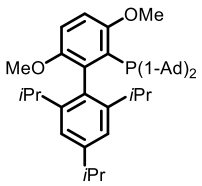

AdBrettPhos (**L26**)

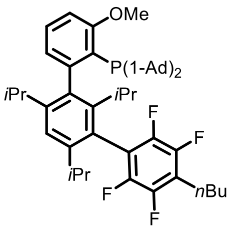

AlPhos (**L27**)

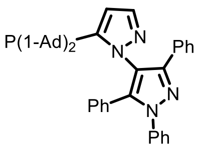

AdBippyPhos (**L28**)

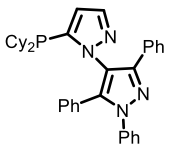

CyBippyPhos (**L29**)

2. Preparation of Starting Materials

2.1 Synthesis Procedures & Characterization Data for Aryl Triflates

General Procedure for the Synthesis of Aryl Triflates

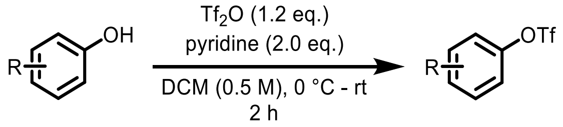

All aryl triflates were prepared according to the following modified literature procedure:[1]

An oven-dried round-bottomed flask equipped with a magnetic stir bar was charged sequentially with phenol (1.0 eq.), DCM (to give $[\text{phenol}]_0$ = 0.5 M) and pyridine (2.0 eq.). The flask was sealed with a rubber septum and the mixture was cooled to 0 °C in an ice bath. The septum was then pierced with an outlet needle and trifluoromethanesulfonic anhydride (1.2 eq.) was added dropwise *via* syringe. The resulting reaction mixture was left to stir for 2 h at room temperature. The reaction mixture was diluted with Et_2O (30 mL), washed with 2 M aq. HCl (2 ×), 2 M aq. NaOH (2 ×) and brine. The combined organic portions were dried over anhydrous Na_2SO_4, filtered, and concentrated to dryness. The purity of the crude material was examined by ^{1}H and ^{19}F NMR spectroscopy; when necessary, the aryl triflates were purified as described for individual entries.

4-Fluorophenyl trifluoromethanesulfonate (1b)

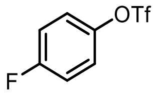

Prepared according to the *General Procedure for the Synthesis of Aryl Triflates* using 4-fluorophenol (1.22 g, 10.0 mmol). After purification by silica gel column chromatography (100% CyH), the title compound (2.19 g, 9.0 mmol, 90%) was obtained as a colourless oil.

Characterisation data were consistent with literature values: ^{1}H, ^{13}C and ^{19}F NMR,[2] ν.[1]

^{1}H NMR (500 MHz, $CDCl_3$): δ 7.30 – 7.25 (m, 2H), 7.17 – 7.12 (m, 2H).

^{13}C{^{1}H} NMR (126 MHz, $CDCl_3$): δ 161.8 (d, *J* = 249.1 Hz), 145.4 (d, *J* = 2.9 Hz), 123.3 (d, *J* = 8.8 Hz), 118.9 (q, *J* = 320.2 Hz), 117.2 (d, *J* = 24.3 Hz).

^{19}F NMR (376 MHz, $CDCl_3$): δ -72.70 (s, 3F), -112.31 (tt, *J* = 7.9, 4.4 Hz, 1F).

ν (ATR)/cm^{-1} (neat): 3087, 1598, 1424, 1206, 1136, 885, 841, 823, 608.

2,5-Dimethylphenyl trifluoromethanesulfonate (1c)

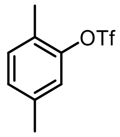

Prepared according to the *General Procedure for the Synthesis of Aryl Triflates* using 2,5-dimethylphenol (0.37 g, 3.0 mmol). The title compound (0.61 g, 2.4 mmol, 80%) was obtained as a light yellow oil. No further purification was needed.

Characterisation data were consistent with literature values: ^{1}H, ^{13}C, ^{19}F NMR and ν.[3]

^{1}H NMR (400 MHz, $CDCl_3$): δ 7.17 (d, *J* = 7.7 Hz, 1H), 7.10 – 7.03 (m, 2H), 2.35 (s, 3H), 2.33 (s, 3H).

^{13}C{^{1}H} NMR (101 MHz, $CDCl_3$): δ 148.5, 138.1, 131.9, 129.1, 127.6, 121.8, 118.8 (q, *J* = 320.3 Hz), 21.0, 16.0.

^{19}F NMR (376 MHz, $CDCl_3$): δ -73.95 (s, 3F).

ν (ATR)/cm^{-1} (neat): 2931, 1575, 1456, 1204, 1139, 1074, 996, 935, 869, 838, 605.

4-Methoxyphenyl trifluoromethanesulfonate

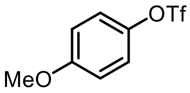

Prepared according to the *General Procedure for the Synthesis of Aryl Triflates* using 4-methoxyphenol (0.62 g, 5.0 mmol). The title compound (1.20 g, 4.7 mmol, 94%) was obtained as a yellow oil. No further purification was needed.

Characterisation data were consistent with literature values: ^{1}H, ^{13}C, ^{19}F NMR and ν.[4]

^{1}H NMR (400 MHz, $CDCl_3$): δ 7.20 (d, *J* = 9.2 Hz, 2H), 6.92 (d, *J* = 9.2 Hz, 2H), 3.82 (s, 3H).

$^{13}C\{^{1}H\}$ NMR (101 MHz, $CDCl_3$): δ 159.2, 143.2, 122.5, 118.9 (q, *J* = 321.8 Hz), 115.2, 55.9.

^{19}F NMR (376 MHz, $CDCl_3$): δ -72.78 (s, 3F).

ν (ATR)/cm^{-1} (neat): 2952, 2842, 1501, 1417, 1205, 1136, 885, 834, 609.

_p_-Tolyl trifluoromethanesulfonate

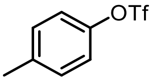

Prepared using *p*-cresol (0.54 g, 5.0 mmol). The title compound (1.10 g, 4.6 mmol, 92%) was obtained as a light-yellow oil. No further purification was needed.

Characterisation data were consistent with literature values: ^{1}H, ^{13}C, ^{19}F NMR and ν.[5]

^{1}H NMR (400 MHz, $CDCl_3$): δ 7.24 (d, *J* = 8.6 Hz, 2H), 7.16 (d, *J* = 8.7 Hz, 2H), 2.38 (s, 3H).

^{13}C{1H} NMR (101 MHz, $CDCl_3$): δ 147.7, 138.7, 130.8, 121.2, 118.9 (q, *J* = 320.9 Hz), 21.0.

^{19}F NMR (376 MHz, $CDCl_3$): δ -72.91 (s, 3F).

ν (ATR)/cm^{-1} (neat): 1501, 1420, 1203, 1132, 881, 821, 606.

***m*-Tolyl trifluoromethanesulfonate**

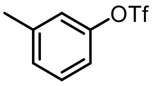

Prepared using *m*-cresol (0.54 g, 5.0 mmol). The title compound (1.01 g, 4.2 mmol, 84%) was obtained as a light-yellow oil. No further purification was needed.

Characterisation data were consistent with literature values: ^{1}H, ^{13}C and ^{19}F NMR,[6] ν.[7]

^{1}H NMR (500 MHz, $CDCl_3$): δ 7.35 – 7.30 (m, 1H), 7.20 (d, *J* = 7.6 Hz, 1H), 7.10 – 7.06 (m, 2H), 2.41 (s, 3H).

^{13}C{^{1}H} NMR (126 MHz, $CDCl_3$): δ 149.7, 141.1, 130.0, 129.3, 121.9, 118.9 (q, *J* = 322.4 Hz), 118.4, 21.4.

^{19}F NMR (376 MHz, $CDCl_3$): δ -73.01 (s, 3F).

ν (ATR)/cm^{-1} (neat): 1615, 1584, 1420, 1203, 1137, 926, 830, 603.

Naphthalen-2-yl trifluoromethanesulfonate

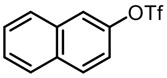

Prepared using naphthalen-2-ol (1.44 g, 10.0 mmol). After bulb-to-bulb distillation (0.75 mbar, 200 °C), the title compound (2.1 g, 7.6 mmol, 76%) was obtained as a white solid

Characterisation data were consistent with literature values: ^{1}H, ^{13}C, ^{19}F NMR and ν.[5]

^{1}H NMR (500 MHz, $CDCl_3$): δ 7.93 (d, *J* = 9.1 Hz, 1H), 7.91 – 7.86 (m, 2H), 7.76 (d, *J* = 2.5 Hz, 1H), 7.61 – 7.55 (m, 2H), 7.38 (dd, *J* = 9.0, 2.5 Hz, 1H).

^{13}C NMR (126 MHz, $CDCl_3$): δ 147.3, 133.5, 132.5, 130.8, 128.2, 128.1, 127.7, 127.4, 119.7, 119.4, 119.0 (q, *J* = 319.8 Hz).

^{19}F NMR (376 MHz, $CDCl_3$): δ -72.79 (s, 3F).

m. p. /°C: 48-49.

ν (ATR)/cm^{-1} (neat): 3064, 1595, 1580, 1201, 1129, 885, 751, 698, 642, 598.

3-Methoxyphenyl trifluoromethanesulfonate

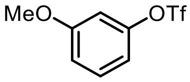

Prepared using 3-methoxyphenol (0.62 g, 5.0 mmol). The title compound (1.10 g, 4.3 mmol, 86%) was obtained as a yellow oil. No further purification was needed.

Characterisation data were consistent with literature values: ^{1}H, ^{13}C and ^{19}F NMR,[8] ν.[9]

^{1}H NMR (400 MHz, $CDCl_3$): δ 7.34 (app. t, *J* = 8.7 Hz, 1H), 6.93 (dd, *J* = 8.4, 2.4 Hz, 1H), 6.87 (dd, *J* = 8.3, 2.1 Hz, 1H), 6.81 (app. t, *J* = 2.2 Hz, 1H), 3.83 (s, 3H).

^{13}C{^{1}H} NMR (101 MHz, $CDCl_3$): δ 161.0, 150.4, 130.7, 118.9 (q, *J* = 320.6 Hz), 114.3, 113.4, 107.6, 55.8.

^{19}F NMR (376 MHz, $CDCl_3$): δ -72.92 (s, 3F).

ν (ATR)/cm^{-1} (neat): 2841, 1612, 1489, 1419, 1205, 1139, 929, 830, 606, 575.

3,5-Dimethoxyphenyl trifluoromethanesulfonate

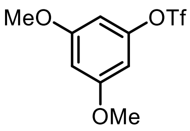

Prepared according to the *General Procedure for the Synthesis of Aryl Triflates* using 3,5-dimethoxyphenol (0.77 g, 5.0 mmol). After a bulb-to-bulb distillation (210 °C, 0.7 mbar), the title compound (0.59 g, 1.7 mmol, 34%) was obtained as a yellow oil.

Characterisation data were consistent with literature values: ^{1}H, ^{13}C and ^{19}F NMR,[10] ν.[11]

^{1}H NMR (400 MHz, $CDCl_3$): δ 6.45 (t, *J* = 2.1 Hz, 1H), 6.42 (d, *J* = 2.1 Hz, 2H), 3.80 (s, 6H).

^{13}C{^{1}H} NMR (101 MHz, $CDCl_3$): δ 161.6, 150.8, 118.9 (q, *J* = 319.2 Hz), 100.4, 100.1, 55.9.

^{19}F NMR (376 MHz, $CDCl_3$): δ -72.92 (s, 3F).

ν (ATR)/cm^{-1} (neat): 2946, 1622, 1587, 1460, 1204, 1139, 1105, 959, 840, 821, 605.

3-Fluorophenyl trifluoromethanesulfonate

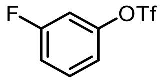

Prepared using 3-fluorophenol (0.49 g, 5.0 mmol). The title compound (0.83 g, 3.4 mmol, 68%) was obtained as a light-yellow oil. No further purification was needed.

Characterisation data were consistent with literature values: ^{1}H, ^{13}C and ^{19}F NMR ,[12] ν.[13]

^{1}H NMR (400 MHz, $CDCl_3$): δ 7.48 – 7.41 (m, 1H), 7.18 – 7.09 (m, 2H), 7.07 – 7.03 (m, 1H).

^{13}C{^{1}H} NMR (101 MHz, $CDCl_3$): δ 162.9 (d, *J* = 252.0 Hz), 149.8 (d, *J* = 10.9 Hz), 131.3 (d, *J* = 8.9 Hz), 118.8 (q, *J* = 320.5 Hz), 117.4 (d, *J* = 3.5 Hz), 115.9 (d, *J* = 21.2 Hz), 110.0 (d, *J* = 25.9 Hz).

^{19}F NMR (376 MHz, $CDCl_3$): δ -72.76 (s, 3F), -108.14 – -108.23 (m, 1F).

ν (ATR)/cm^{-1} (neat): 3098, 1603, 1486, 1424, 1208, 1102, 1072, 949, 83, 718, 573.

3-(Trifluoromethyl)phenyl trifluoromethanesulfonate

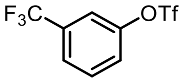

Prepared using 3-(trifluoromethyl)phenol (0.97 g, 6.0 mmol). The title compound (1.29 g, 4.4 mmol, 73%) was obtained as a bright yellow oil. No further purification was needed.

Characterisation data were consistent with literature values: ^{1}H, ^{13}C, ^{19}F NMR and ν.[14]

^{1}H NMR (500 MHz, $CDCl_3$): δ 7.69 (d, *J* = 7.8 Hz, 1H), 7.63 (app. t, *J* = 8.1 Hz, 1H), 7.56 – 7.53 (m, 1H), 7.50 (dd, *J* = 8.1, 2.0 Hz, 1H).

^{13}C{^{1}H} NMR (126 MHz, $CDCl_3$): δ 149.5, 133.2 (q, *J* = 33.7 Hz), 131.3, 125.5 (q, *J* = 3.9 Hz), 125.1, 123.0 (q, *J* = 272.9 Hz), 119.0 (q, *J* = 4.3 Hz), 118.9 (q, *J* = 320.2 Hz).

^{19}F NMR (377 MHz, $CDCl_3$): δ -62.98 (s, 3F), -72.79 (s, 3F).

ν (ATR)/cm^{-1} (neat): 1427, 1325, 1212, 1131, 892, 569.

Methyl 4-(((trifluoromethyl)sulfonyl)oxy)benzoate

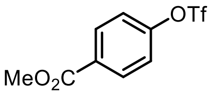

Prepared according to the *General Procedure for the Synthesis of Aryl Triflates* using methyl 4-hydroxybenzoate (0.761 g, 5.0 mmol). The title compound (1.39 g, 4.9 mmol, 90%) was obtained as a yellow oil. No further purification was needed.

Characterisation data were consistent with literature values: ^{1}H, ^{13}C and ^{19}F NMR,[15] ν.[16]

^{1}H NMR (400 MHz, $CDCl_3$): δ 8.14 (d, *J* = 8.8 Hz, 2H), 7.35 (d, *J* = 8.8 Hz, 2H), 3.94 (s, 3H).

^{13}C{^{1}H} NMR (101 MHz, $CDCl_3$): δ 165.6, 152.6, 132.1, 130.5, 121.6, 118.8 (q, *J* = 320.3 Hz), 52.7.

^{19}F NMR (377 MHz, $CDCl_3$): δ -72.78 (s, 3F).

ν (ATR)/cm^{-1} (neat): 2958, 1728, 1601, 1499, 1425, 1280, 1280, 1137, 861, 607.

4-(Trifluoromethyl)phenyl trifluoromethanesulfonate

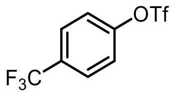

Prepared according to the *General Procedure for the Synthesis of Aryl Triflates* using methyl 4-(trifluoromethyl)phenol (0.648 g, 4.0 mmol). The title compound (0.79 g, 2.7 mmol, 68%) was obtained as a yellow oil. No further purification was needed.

Characterisation data were consistent with literature values: ^{1}H, ^{13}C and ^{19}F NMR, ν.[17]

^{1}H NMR (400 MHz, $CDCl_3$): δ 7.75 (d, *J* = 8.4 Hz, 2H), 7.42 (d, *J* = 8.4 Hz, 2H).

$^{13}C\{^{1}H\}$ NMR (101 MHz, $CDCl_3$): δ 151.7, 131.0 (q, *J* = 34.1 Hz), 127.9 (q, *J* = 3.4 Hz), 123.4 (q, *J* = 272.2 Hz), 122.2, 118.9 (q, *J* = 319.9 Hz).

^{19}F NMR (377 MHz, $CDCl_3$): δ -62.69 (s, 3F), -72.74 (s, 3F).

ν (ATR)/cm^{-1} (neat): 3123, 1671, 1429, 1323, 1211, 1132, 883, 573.

4-Cyanophenyl trifluoromethanesulfonate

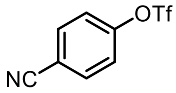

Prepared according to the *General Procedure for the Synthesis of Aryl Triflates* using 4-hydroxybenzonitrile (1.19 g, 10.0 mmol). After purification by silica gel column chromatography (9:1 pentane/EtOAc), the title compound (1.81 g, 7.2 mmol, 72%) was obtained as a colourless oil.

Characterisation data were consistent with literature values: ^{1}H, ^{13}C and ^{19}F NMR,[18] ν.[9]

^{1}H NMR (400 MHz, $CDCl_3$): δ 7.79 (d, *J* = 9.0 Hz, 2H), 7.43 (d, *J* = 9.0 Hz, 2H).

$^{13}C\{^{1}H\}$ NMR (101 MHz, $CDCl_3$): δ 152.1, 134.6, 122.8, 117.2 (q, *J* = 320.6 Hz), 117.2, 113.1.

^{19}F NMR (376 MHz, $CDCl_3$): δ -72.63 (s, 3F).

ν (ATR)/cm^{-1} (neat): 3106, 2238, 1422, 1208, 1134, 854, 798, 607, 580.

3,5-Difluorophenyl trifluoromethanesulfonate

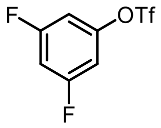

Prepared according to the *General Procedure for the Synthesis of Aryl Triflates* using 3,5-difluorophenol (0.65 g, 5.0 mmol). After purification by silica gel column chromatography (100% pentane), the title compound (1.02 g, 3.9 mmol, 78%) was obtained as a colourless oil.

Characterisation data were consistent with literature values: ^{1}H, ^{13}C and ^{19}F NMR.[19]

^{1}H NMR (400 MHz, $CDCl_3$): δ 6.94 – 6.86 (m, 3H).

^{13}C{^{1}H} NMR (101 MHz, $CDCl_3$): δ 164.6 – 161.8 (m), 150.1 – 149.7 (m), 118.8 (q, *J* = 321.2 Hz), 106.5 – 106.1 (m), 105.0 – 104.4 (m).

^{19}F NMR (377 MHz, $CDCl_3$): δ -72.61 (s, 3F), -105.35 (app. t, *J* = 8.2 Hz, 2F).

ν (ATR)/cm^{-1} (neat): 3108, 1613, 1465, 1429, 1212, 981, 857, 817, 584, 545.

4-Nitrophenyl trifluoromethanesulfonate

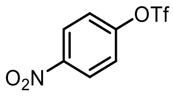

Prepared according to the *General Procedure for the Synthesis of Aryl Triflates* using 4-nitrophenol (0.696 g, 5.0 mmol). The title compound (1.19 g, 4.4 mmol, 88%) was obtained as an off-white solid. No further purification was needed.

Characterisation data were consistent with literature values: ^{1}H, ^{13}C, ^{19}F NMR, m. p. and ν.[19]

^{1}H NMR (400 MHz, CDCl$_3$): δ 8.37 (d, *J* = 9.2 Hz, 2H), 7.48 (d, *J* = 9.2 Hz, 2H).

^{13}C{^{1}H} NMR (101 MHz, CDCl$_3$): δ 153.3, 147.3, 126.2, 122.7, 118.8 (q, *J* = 320.6 Hz).

^{19}F NMR (376 MHz, CDCl$_3$): δ -72.52 (s, 3F).

m. p. /°C: 49-50.

ν (ATR)/cm^{-1} (neat): 3124, 1622, 1533, 1485, 1420, 1129, 1210, 858, 740, 608, 523.

2-(Benzyloxy)phenyl trifluoromethanesulfonate

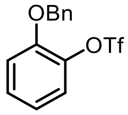

Prepared according to the *General Procedure for the Synthesis of Aryl Triflates* using 2-(benzyloxy)phenol (1.00 g, 5.0 mmol). After a bulb-to-bulb distillation (230 °C, 0.5 mbar), the title compound (14.0 g, 4.2 mmol, 84%) was obtained as a yellow oil.

^{1}H NMR (400 MHz, $CDCl_3$): δ 7.50 – 7.22 (m, 7H), 7.08 (dd, *J* = 8.3, 1.3 Hz, 1H), 7.02 – 6.96 (m, 1H), 5.19 (s, 2H).

^{13}C{^{1}H} NMR (101 MHz, $CDCl_3$): δ 150.7, 139.2, 135.9, 129.3, 128.8, 128.4, 127.4, 122.7, 121.3, 118.9 (q, *J* = 320.6 Hz), 114.7, 71.2.

^{19}F NMR (376 MHz, $CDCl_3$): δ -73.90 (s, 3F).

HMRS (ESI$^+$, *m/z*): calcd. for $C_{14}H_{11}F_3O_4S+Na^+$: 355.0222 $[M+Na]^+$. Found: 355.0221.

ν (ATR)/cm^{-1} (neat): 3036, 1611, 1498, 1454, 1419, 1205, 1138, 890, 755, 573.

Ethyl (*S*)-2,5,7,8-tetramethyl-6-(((trifluoromethyl)sulfonyl)oxy)chromane-2-carboxylate

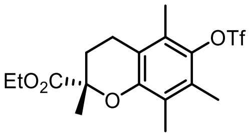

Prepared according to the *General Procedure for the Synthesis of Aryl Triflates* using **3a** (0.69 g, 2.5 mmol). After purification by silica gel column chromatography (0-20% EtOAc in pentane), the title compound (0.90 g, 2.2 mmol, 88%) was obtained as a viscous colourless oil.

^{1}H NMR (500 MHz, $CDCl_3$): δ 4.18 – 4.09 (m, 2H), 2.69 – 2.60 (m, 1H), 2.54 – 2.41 (m, 2H), 2.24 (s, 3H), 2.19 (s, 3H), 2.16 (s, 3H), 1.92 – 1.83 (m, 1H), 1.63 (s, 3H), 1.18 (app. t, *J* = 7.2 Hz, 3H).

^{13}C{^{1}H} NMR (126 MHz, $CDCl_3$): δ 173.2, 151.0, 140.4, 128.6, 126.8, 124.6, 118.8 (q, *J* = 319.9 Hz), 118.4, 77.7, 61.4, 30.2, 25.4, 21.1, 14.2, 14.6, 13.3, 12.1.

^{19}F NMR (376 MHz, $CDCl_3$): δ -73.64 (s, 3F).

HMRS (ESI$^+$, *m/z*): calcd. for $C_{17}H_{21}F_3O_6S$+Na^+: 433.0903 [M+Na]$^+$. Found: 433.0902.

ν (ATR)/cm^{-1} (neat): 2983, 2937, 1749, 1733, 1401, 1203, 1137, 1109, 1018, 877, 671, 612.

***tert*-Butyl 3-(2-(1,3-dioxoisoindolin-2-yl)ethyl)-5-(((trifluoromethyl)sulfonyl)oxy)-1*H*-indole-1-carboxylate**

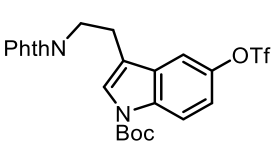

3-(2-(1,3-Dioxoisoindolin-2-yl)ethyl)-1H-indol-5-yl trifluoromethanesulfonate[20] (1.01 g, 2.30 mmol) was dissolved in MeCN (10 mL), then DMAP (28.1 mg, 0.23 mmol) and Boc anhydride (0.79 mL, 3.45 mmol) were added sequentially. The reaction was stirred at room temperature for 1 h, then concentrated *in vacuo*. Purification by silica gel column chromatography (100% CyH, then 0-20% EtOAc in CyH) afforded the title compound (1.07 g, 2.0 mmol, 87%) as a colourless crystalline solid.

^{1}H NMR (400 MHz, $CDCl_3$): δ 8.19 (d, *J* = 8.9 Hz, 1H), 7.90 – 7.81 (m, 2H), 7.76 – 7.68 (m, 2H), 7.58 (s, 1H), 7.55 (d, *J* = 1.9 Hz, 1H), 7.20 (dd, *J* = 9.0, 1.9 Hz, 1H), 3.99 (t, *J* = 7.6 Hz, 2H), 3.07 (t, *J* = 7.6 Hz, 2H), 1.65 (s, 9H).

^{13}C{^{1}H} NMR (101 MHz, $CDCl_3$): δ 168.3, 149.2, 145.4, 134.6, 134.2, 132.2, 131.4, 125.8, 123.5, 119.0 (q, *J* = 321.4 Hz), 117.6, 117.0, 116.7, 111.7, 84.6, 37.6, 28.3, 24.2.

^{19}F NMR (376 MHz, $CDCl_3$): δ -72.66 (s, 3F).

HMRS (ESI$^+$, *m/z*): calcd. for $C_{24}H_{21}F_3N_2O_7S+NH_4^+$: 556.1360 $[M+NH_4]^+$. Found: 556.1359.

m. p. /°C: 152-153.

ν (ATR)/cm^{-1} (neat): 2990, 1712, 1372, 1279, 1141, 915, 842.

5-(Trifluoromethyl)pyridin-2-yl trifluoromethanesulfonate

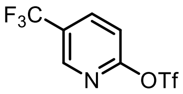

Prepared according to the *General Procedure for the Synthesis of Aryl Triflates* using 5-(trifluoromethyl)pyridin-2-ol (0.815 g, 5.0 mmol). After purification by silica gel column chromatography (0-3% EtOAc in CyH), the title compound (1.21 g, 4.1 mmol, 82%) was obtained as a colourless oil.

^{1}H NMR (400 MHz, CDCl$_3$): δ 8.72 – 8.69 (m, 1H), 8.15 (dd, *J* = 8.5, 2.4 Hz, 1H), 7.33 (d, *J* = 8.5 Hz, 1H).

^{13}C{^{1}H} NMR (101 MHz, CDCl$_3$): δ 157.9, 146.5 (q, *J* = 3.9 Hz), 138.7 (q, *J* = 3.4 Hz), 127.6 (q, *J* = 33.7 Hz), 122.8 (q, *J* = 272.0 Hz), 118.7 (q, *J* = 320.6 Hz), 115.5.

^{19}F NMR (376 MHz, CDCl$_3$): δ -62.21 (s, 3F), -72.82 (s, 3F).

MS (EI): calcd. for $C_7H_3NO_3F_6S^+$ ([M]$^+$): 294.97323. Found: 294.97352.

ν (ATR)/cm^{-1} (neat): 1599, 1479, 1426, 1327, 1212, 1171, 1131, 887, 730, 575.

4-Fluoropyridin-2-yl trifluoromethanesulfonate

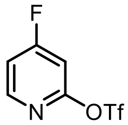

Prepared using 4-fluoropyridin-2-ol (0.677 g, 6.0 mmol). The title compound (1.10 g, 4.5 mmol, 75%) was obtained as a bright yellow oil. No further purification was needed.

^{1}H NMR (400 MHz, $CDCl_3$): δ 8.42 – 8.36 (m, 1H), 7.18 – 7.12 (m, 1H), 6.93 (dd, *J* = 7.9, 2.1 Hz, 1H).

^{13}C{^{1}H} NMR (101 MHz, $CDCl_3$): δ 170.8 (d, *J* = 266.1 Hz), 157.1 (d, *J* = 10.7 Hz), 150.6 (d, *J* = 9.2 Hz), 118.7 (q, *J* = 322.1 Hz), 112.8 (d, *J* = 17.0 Hz), 103.9 (d, *J* = 23.2 Hz).

^{19}F NMR (377 MHz, $CDCl_3$): δ -73.03 (s, 3F), -94.99 (app. q, *J* = 7.9 Hz, 1F).

MS (EI): calcd. for $C_6H_3NO_3F_4S^+$ ([M]$^+$): 244.97643. Found: 244.97704.

ν (ATR)/cm^{-1} (neat): 3110, 1604, 1585, 1422, 1402, 1205, 1118, 1075, 957, 828, 605, 511.

2-Fluoropyridin-4-yl trifluoromethanesulfonate

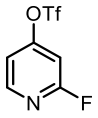

Prepared according to the *General Procedure for the Synthesis of Aryl Triflates* using 2-fluoropyridin-4-ol (0.677 g, 6.0 mmol). The title compound (1.40 g, 5.7 mmol, 95%) was obtained as a light-yellow oil. No further purification was needed.

^{1}H NMR (400 MHz, $CDCl_3$): δ 8.36 (d, *J* = 5.6 Hz, 1H), 7.17 (dd, *J* = 5.8, 1.9 Hz, 1H), 6.95 – 6.89 (m, 1H).

^{13}C{^{1}H} NMR (101 MHz, $CDCl_3$): δ 164.6 (d, *J* = 241.0 Hz), 158.8 (d, *J* = 10.8 Hz), 150.1 (d, *J* = 17.0 Hz), 118.7 (q, *J* = 321.0 Hz), 114.5 (d, *J* = 5.7 Hz), 103.3 (d, *J* = 40.7 Hz).

^{19}F NMR (377 MHz, $CDCl_3$): δ -61.84 (app. s, 1F), -72.67 (s, 3F).

MS (EI): calcd. for $C_6H_3NO_3F_4S^+$ ([M]$^+$): 244.97643. Found: 244.97646.

ν (ATR)/cm^{-1} (neat): 1671, 1609, 1508, 1323, 1132, 883, 608.

Methyl (*R*)-2-((*tert*-butoxycarbonyl)amino)-3-(4-(((trifluoromethyl)sulfonyl)oxy)phenyl) propanoate

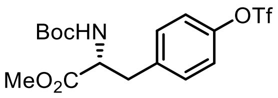

Prepared according to the *General Procedure for the Synthesis of Aryl Triflates* using methyl (*tert*-butoxycarbonyl)-*D*-tyrosinate (4.43 g, 15.0 mmol). *Adjusted work-up*: The reaction was diluted with DCM (20 mL), washed with 2 M aq. HCl (2 × 50 mL), 2 M aq. NaOH (2 × 50 mL) and brine (40 mL). After purification by silica gel column chromatography (0-20% EtOAc in CyHex), the title compound (5.81 g, 13.6 mmol, 91%) was obtained as an off-white solid.

Characterisation data were consistent with literature values: ^{1}H, ^{13}C NMR and m. p.,[21] ν.[22]

^{1}H NMR (400 MHz, $CDCl_3$): δ 7.27 – 7.16 (m, 4H), 5.04 (d, major rotamer, *J* = 8.2 Hz, 0.86H), 4.82 (br. s, minor rotamer, 0.14H), 4.66 – 4.51 (m, major rotamer, 0.88H), 4.40 (br. s, minor rotamer, 0.12H), 3.70 (s, 3H), 3.21 – 2.97 (m, 2H), 1.40 (s, 9H).

^{13}C{^{1}H} NMR (101 MHz, $CDCl_3$): δ 172.0, 155.1, 148.7, 137.1, 131.3, 121.5, 118.9 (q, *J* = 320.8 Hz), 80.3, 54.3, 52.5, 38.0, 28.4.

^{19}F NMR (376 MHz, $CDCl_3$): δ -72.90 (s, 3F).

m. p. /°C: 52-53.

ν (ATR)/cm^{-1} (neat): 3382, 2983, 1733, 1689, 1515, 1446, 1216, 1196, 1171, 1140, 895, 594.

4-Methyl-2-oxo-2*H*-chromen-7-yl trifluoromethanesulfonate

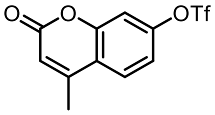

Prepared according to the *General Procedure for the Synthesis of Aryl Triflates* using 7-hydroxy-4-methyl-2*H*-chromen-2-one (1.34 g, 8.0 mmol). After purification by silica gel column chromatography (0-25% EtOAc in pentane), the title compound (2.31 g, 7.5 mmol, 94%) was obtained as an off-white solid.

Characterisation data were consistent with literature values: ^{1}H, ^{13}C and ^{19}F NMR,[18] m. p. and ν.[23]

^{1}H NMR (400 MHz, $CDCl_3$): δ 7.70 (d, *J* = 8.8 Hz, 1H), 7.27 (d, *J* = 2.4 Hz, 1H), 7.24 (dd, *J* = 8.8, 2.5 Hz, 1H), 6.35 (q, *J* = 1.3 Hz, 1H), 2.46 (d, *J* = 1.2 Hz, 3H).

^{13}C{^{1}H} NMR (101 MHz, $CDCl_3$): δ 159.6, 154.3, 151.3, 150.9, 126.5, 120.2, 118.8 (q, *J* = 321.0 Hz), 117.5, 116.1, 110.6, 18.9.

^{19}F NMR (377 MHz, $CDCl_3$): δ -72.58 (s, 3F).

m. p. /°C: 87-88.

ν (ATR)/cm^{-1} (neat): 3094, 1734, 1627, 1604, 1384, 1178, 1064, 1013, 844, 678, 636.

2-Fluoropyridin-3-yl trifluoromethanesulfonate

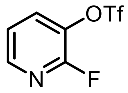

Prepared according to the *General Procedure for the Synthesis of Aryl Triflates* using 2-fluoropyridin-3-ol (0.678 g, 6.0 mmol). After purification by silica gel column chromatography (95:5 pentane/EtOAc), the title compound (1.20 g, 4.9 mmol, 82%) was obtained as a colourless oil.

Characterisation data were consistent with literature values: ^{1}H, ^{13}C and ^{19}F NMR.[24]

^{1}H NMR (400 MHz, CDCl$_3$): δ 8.27 – 8.22 (m, 1H), 7.83 – 7.74 (m, 1H), 7.36 – 7.29 (m, 1H).

^{13}C{^{1}H} NMR (101 MHz, CDCl$_3$): δ 154.8 (d, *J* = 241.9 Hz), 146.9 (d, *J* = 14.4 Hz), 133.9, 132.5 (d, *J* = 28.7 Hz), 122.8 (d, *J* = 4.9 Hz), 118.8 (q, *J* = 320.8 Hz).

^{19}F NMR (376 MHz, CDCl$_3$): δ -73.04 (app. d, *J* = 5.0 Hz, 3F), -79.06 (app. s br., 1F)

ν (ATR)/cm^{-1} (neat): 3085, 1604, 1428, 1209, 1134, 1096, 893, 828, 721, 576.

5-Fluoropyridin-2-yl trifluoromethanesulfonate

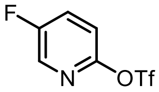

Prepared according to the *General Procedure for the Synthesis of Aryl Triflates* using 5-fluoropyridin-2-ol (0.678 g, 6.0 mmol). The title compound (1.30 g, 5.3 mmol, 88%) was obtained as a yellow oil. No further purification was needed.

^{1}H NMR (400 MHz, $CDCl_3$): δ 8.24 – 8.21 (m, 1H), 7.78 – 7.72 (m, 1H), 7.06 (dd, *J* = 8.9, 3.5 Hz, 1H).

^{13}C{^{1}H} NMR (101 MHz, $CDCl_3$): δ 162.0 (d, *J* = 242.9 Hz), 144.4 (d, *J* = 5.1 Hz), 140.9 (d, *J* = 16.8 Hz), 134.6 (d, *J* = 9.0 Hz), 118.8 (q, *J* = 321.0 Hz), 111.3 (d, *J* = 40.7 Hz).

^{19}F NMR (377 MHz, $CDCl_3$): δ -66.20 (app. s br., 1F), -72.33 (s, 3F).

MS (EI): calcd. for $C_6H_3NO_3F_4S^+$ ([M]$^+$): 244.97643. Found: 244.97693.

ν (ATR)/cm^{-1} (neat): 3110, 1605, 1475, 1426, 1391, 1206, 1164, 1134, 1018, 833, 711, 562.

4-Chlorophenyl trifluoromethanesulfonate

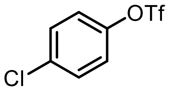

Prepared according to the *General Procedure for the Synthesis of Aryl Triflates* using 4-chlorophenol (0.64 g, 5.0 mmol). The title compound (0.63 g, 2.4 mmol, 48%) was obtained as a yellow oil. No further purification was needed.

Characterisation data were consistent with literature values: ^{1}H, ^{13}C, ^{19}F NMR and ν.[25]

^{1}H NMR (400 MHz, $CDCl_3$): δ 7.43 (d, *J* = 8.8 Hz, 2H), 7.23 (d, *J* = 8.8 Hz, 2H).

^{13}C{^{1}H} NMR (101 MHz, $CDCl_3$): δ 148.0, 134.5, 130.5, 122.9, 118.8 (q, *J* = 320.4 Hz).

^{19}F NMR (376 MHz, $CDCl_3$): δ -72.70 (s, 3F).

ν (ATR)/cm^{-1} (neat): 3105, 1484, 1424, 1173, 1137, 1090, 883, 834, 529.

2-Allylphenyl trifluoromethanesulfonate

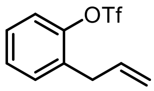

Prepared according to the *General Procedure for the Synthesis of Aryl Triflates* using 2-allylphenol (0.65 mL, 5.0 mmol). The title compound (0.93 g, 3.5 mmol, 70%) was obtained as orange oil. No further purification was needed.

Characterisation data were consistent with literature values: ^{1}H, ^{13}C NMR and ν.[26]

^{1}H NMR (400 MHz, $CDCl_3$): δ 7.37 – 7.25 (m, 4H), 5.98 – 5.87 (m, 1H), 5.18 – 5.09 (m, 2H), 3.52 – 3.46 (m, 2H).

^{13}C{^{1}H} NMR (101 MHz, $CDCl_3$): δ 148.1, 134.7, 133.0, 131.6, 128.6, 128.3, 121.5, 118.8 (q, *J* = 319.8 Hz), 117.6, 34.1.

^{19}F NMR (376 MHz, $CDCl_3$): δ -73.86 (s, 3F).

ν (ATR)/cm^{-1} (neat): 3084, 1487, 1453, 1418, 1208, 1138, 888, 766.

4-Cyano-2,6-dimethylphenyl trifluoromethanesulfonate

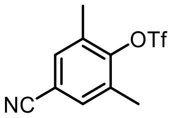

Prepared according to the *General Procedure for the Synthesis of Aryl Triflates* using 4-hydroxy-3,5-dimethylbenzonitrile (0.589 g, 4.0 mmol). The title compound (1.00 g, 3.6 mmol, 90%) was obtained as a crystalline white solid. No further purification was needed.

Characterisation data were consistent with literature values: ^{1}H and ^{13}C NMR, ν and m. p.,[27] ^{19}F NMR.[28]

^{1}H NMR (400 MHz, CDCl$_3$): δ 7.45 (s, 2H), 2.43 (s, 6H).

^{13}C{^{1}H} NMR (126 MHz, CDCl$_3$): δ 149.5, 133.8, 133.6, 118.6 (q, *J* = 320.0 Hz), 117.6, 112.5, 17.3.

^{19}F NMR (376 MHz, CDCl$_3$): δ -73.10 (s, 3F).

m. p. /°C: 52-53.

ν (ATR)/cm^{-1} (neat): 2229, 1406, 1382, 1216, 1135, 1092, 853, 633.

2.2 Synthesis Procedures & Characterization Data for Phenols

Ethyl (*S*)-6-hydroxy-2,5,7,8-tetramethylchromane-2-carboxylate

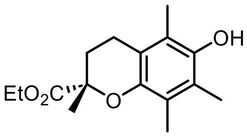

Prepared according to literature procedure.[29] Characterisation data were consistent with literature values: ^{1}H and ^{13}C NMR,[30] m. p. and HRMS.[31]

^{1}H NMR (400 MHz, CDCl$_3$): δ 4.26 (s, 1H), 4.12 (q, *J* = 7.1 Hz, 2H), 2.69 – 2.60 (m, 1H), 2.58 – 2.48 (m, 1H), 2.47 – 2.39 (m, 1H), 2.19 (s, 3H), 2.15 (s, 3H), 2.06 (s, 3H), 1.93 – 1.82 (m, 1H), 1.60 (s, 3H), 1.18 (app. t, *J* = 7.1 Hz, 3H).

^{13}C{^{1}H} NMR (101 MHz, CDCl$_3$): δ 174.0, 145.8, 145.4, 122.7, 121.3, 118.5, 117.1, 77.0, 61.1, 30.7, 25.5, 21.1, 14.2, 12.3, 11.9, 11.4.

HMRS (ESI$^+$, *m/z*): calcd. for $C_{16}H_{22}O_4$+Na$^+$: 301.1410 [M+Na]$^+$. Found: 301.1408.

m. p./ °C: 124-125.

ν (ATR)/cm^{-1} (neat): 3525, 2985, 1732, 1183, 1107, 1021.

Methyl (1*S*,4a*S*,10a*R*)-6-hydroxy-1,4a-dimethyl-1,2,3,4,4a,9,10,10a-octahydrophenanthrene-1-carboxylate

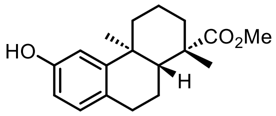

Prepared according to literature procedure.[32] After purification by silica gel column chromatography (9:1 pentane/EtOAc), the title compound (70 mg, 0.24 mmol, 56%) was obtained as an off-white crystalline solid.

Characterisation data were consistent with literature values: ^{1}H and ^{13}C NMR,[33] HRMS and m.p.,[32] ν.[34]

^{1}H NMR (400 MHz, CDCl$_3$): δ 6.91 (d, *J* = 8.2 Hz, 1H), 6.74 (d, *J* = 2.8 Hz, 1H), 6.59 (dd, *J* = 8.2, 2.8 Hz, 1H), 4.68 (s, 1H), 3.66 (s, 3H), 2.83 (ddd, *J* = 16.3, 5.3, 1.4 Hz, 1H), 2.77 – 2.65 (m, 1H), 2.32 – 2.24 (m, 1H), 2.22 – 2.13 (m, 2H), 2.07 – 1.87 (m, 2H), 1.66 – 1.58 (m, 1H), 1.52 (dd, *J* = 12.3, 1.7 Hz, 1H), 1.39 (app. td, *J* = 13.5, 4.1 Hz, 1H), 1.27 (s, 3H), 1.08 (app. tt, *J* = 13.5, 4.1 Hz, 1H), 1.02 (s, 3H).

^{13}C{^{1}H} NMR (101 MHz, CDCl$_3$): δ 178.2, 153.7, 149.7, 130.2, 127.7, 113.1, 112.2, 52.9, 51.4, 44.1, 39.5, 38.7, 37.7, 31.4, 28.7, 23.0, 21.2, 20.1.

HMRS (ESI$^+$, *m/z*): calcd. for $C_{18}H_{24}O_3+Na^+$: 311.1618 $[M+Na]^+$. Found: 311.1622.

m. p./ °C: 215-216.

ν (ATR)/cm^{-1} (neat): 3421.93, 2951.40 2846.75 1691.83

3. Reaction Development

3.1 Base / Electrophile Screening (Manuscript Table 1)

Preparation of Stock Solutions

<u>*t*-BuXPhos-Pd-G3 stock solution (A).</u> *t*BuXPhos-Pd-G3 (7.94 mg, 0.01 mmol) was added to a 1 mL volumetric flask. DCM was added to give 1 mL of a 0.01 M stock solution, which was used immediately.

<u>Coupling partner stock solution (B).</u> *One stock solution was prepared for each aryl (pseudo)halide to be investigated.* 2,5-Dimethylphenol (366 mg, 3.0 mmol) and internal standard (4,4'-bis(trifluoromethyl)biphenyl) were added to a flame-dried Schlenk flask. The vessel was sealed with a rubber septum, then evacuated and backfilled with N_2 (total of three cycles). Anhydrous PhMe (5.0 mL) was then added under N_2, followed by either 4-bromofluorobenzene (275 µL, 2.5 mmol) or 4-fluorophenyl triflate (610 mg, 2.5 mmol).

<u>Base stock solution (C).</u> *One stock solution was prepared for each organic base to be investigated.* A flame-dried microwave vial was sealed, then evacuated and backfilled with N_2 (total of three cycles). Anhydrous PhMe (5.0 mL) was then added under N_2, followed by the appropriate organic base (3.75 mmol).

Experimental Procedure for Base Screening

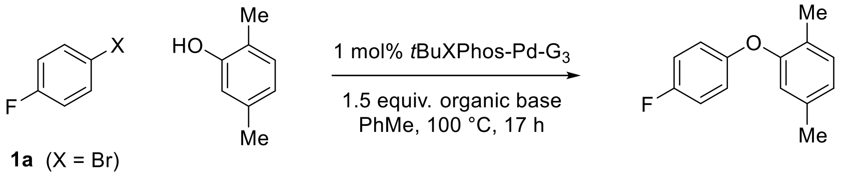

1a (X = Br)
1b (X = OTf)
2a (1.2 equiv.)
3

*t*BuXPhos-Pd-G3 *stock solution A* (0.01 M; 100 µL, 0.001 mmol) was added to a flame-dried microwave vial equipped with a magnetic stir bar. The vial was left open to allow solvent evaporation, then sealed, and evacuated and backfilled with N_2 (total of three cycles). The appropriate coupling partner *stock solution B* (0.5 M; 200 µL, 0.10 mmol) and the appropriate base *stock solution C* (0.75 M; 200 µL, 0.15 mmol) were added under N_2. The reaction mixture was stirred at 100 °C for 17 h before being quenched with acetic acid (10 µL). An aliquot (100 µL) was then removed and transferred into an NMR tube containing $CDCl_3$ (500 µL) for analysis by quantitative ^{19}F NMR spectroscopy.

The screening results are presented in Manuscript Table 1.

3.2 Investigation into Catalyst Poisoning

Poisoning Effects of Organic Bases

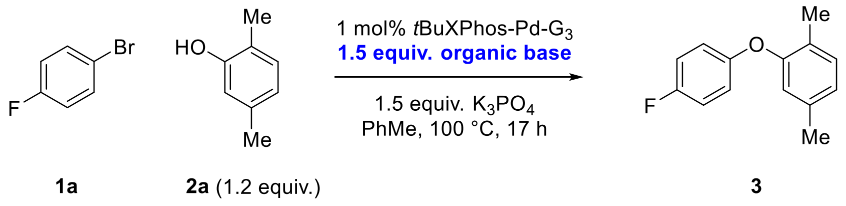

Entry	Organic base additive	% Yield 3
1	none	46
2	DIPEA	25
3	BIMA	36
4	PMP	32
5	DBU	0
6	BTMG	25
7	BTPP	20

Table S1. *Yields determined by quantitative ^{19}F NMR spectroscopic analysis vs. internal standard (4,4´-bis(trifluoromethyl)-1,1´-biphenyl).*

Experimental Procedure. Stock solutions were prepared as described in Section 3.1. *t*BuXPhos-Pd-G3 *stock solution A* (0.01 M; 100 µL, 0.001 mmol) was added to a flame-dried microwave vial equipped with a magnetic stir bar. The vial was left open to allow solvent evaporation, then sealed, and evacuated and backfilled with N_2 (total of three cycles). K_3PO_4 (31.8 mg, 0.15 mmol) was added against a flow of dinitrogen, then the flask was again sealed, evacuated and backfilled with N_2 (total of three cycles). The 4-fluorobromobenzene *stock solution B* (0.5 M; 200 µL, 0.10 mmol) and the appropriate base *stock solution C* (0.75 M; 200 µL, 0.15 mmol) were added under N_2. The reaction mixture was stirred at 100 °C for 17 h before being quenched with acetic acid (10 µL). An aliquot (100 µL) was then removed and transferred into an NMR tube containing $CDCl_3$ (500 µL) for analysis by quantitative ^{19}F NMR spectroscopy.

Commentary. The effects of organic base additives are apparent by comparing entries 2-7 (Table S1) with the organic-base-free control reaction (entry 1). In all cases, the yield is reduced relative to entry 1, potentially indicating that all organic bases have an inhibitory effect on the Pd catalyst. However, the inhibitory effect of DBU on the C-O coupling is particularly significant (entry 5), which is consistent with observations made in DBU-mediated C-N couplings.[35]

Poisoning Effects of Other Additives

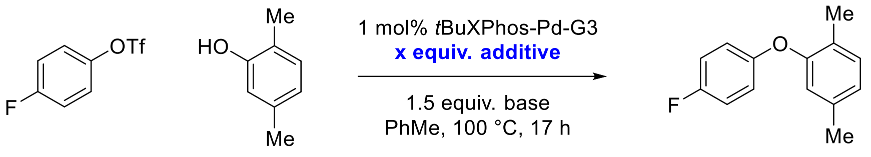

1a **2a** (1.2 equiv.) **3**

Entry	Additive (equiv.)	% Yield 3		
		DIPEA	BIMA	PMP
1	none	0	16	24
2	$[(C_{16}H_{33})_2NMe_2]Br$ (1.0)	0	0	0
3	Carbazole (0.1)	4	10	26

Table S2. *Yields determined by quantitative ^{19}F NMR spectroscopic analysis vs. internal standard (4,4´-bis(trifluoromethyl)-1,1´-biphenyl).*

Experimental Procedure. Stock solutions were prepared as described in Section 3.1. *t*BuXPhos-Pd-G3 *stock solution A* (0.01 M; 100 µL, 0.001 mmol) was added to a flame-dried microwave vial equipped with a magnetic stir bar. The vial was left open to allow solvent evaporation, then sealed, and evacuated and backfilled with N_2 (total of three cycles). The appropriate additive was added against a flow of dinitrogen, then the flask was again sealed, evacuated and backfilled with N_2 (total of three cycles). The 4-fluorophenyl triflate *stock solution B* (0.5 M; 200 µL, 0.10 mmol) and the appropriate base *stock solution C* (0.75 M; 200 µL, 0.15 mmol) were added under N_2. The reaction mixture was stirred at 100 °C for 17 h before being quenched with acetic acid (10 µL). An aliquot (100 µL) was then removed and transferred into an NMR tube containing $CDCl_3$ (500 µL) for analysis by quantitative ^{19}F NMR spectroscopy.

Commentary. Comparison of entry 1 (Table S2) with entry 2 indicates that the addition of 1 equiv. bromide completely inhibits the C-O coupling of the aryl triflate electrophile, irrespective of the base used. This is consistent with the need for a weakly coordinated oxidative addition intermediate of the type LPd(Ar)(OTf) for productive catalyst turnover, and suggests that the poor performance of the aryl bromide electrophiles with weaker (tertiary amine) bases is not necessarily due to different rates of oxidative addition into Ar-OTf vs Ar-Br. Comparison of entry 1 and entry 3 demonstrates that carbazole – the co-product of precatalyst activation – does not have a significant impact on reaction yield. However, it should be noted that N-arylation of carbazole was observed (^{19}F NMR spectroscopy; consistent with literature values[36]) for all bases in entry 3. To avoid the formation of this side-product, Buchwald's G4 palladacycle was used in subsequent investigations of reaction scope.

3.3 Investigation into Triflyl Migration

To gain a deeper insight into the triflyl migration observed when strong organic bases are used in the C-O coupling, the reaction was monitored by *in situ* ^{19}F NMR spectroscopy in the presence of BTPP both with (Figure S1) and without (Figure S2) a Pd-precatalyst. It was observed that the triflyl metathesis reaction commenced immediately at room temperature, and reaches equilibrium in less than 1 hour upon heating to 100 °C, irrespective of whether the Pd-precatalyst was added. When performed in the presence of a Pd-precatalyst, the triflyl migration significantly outcompetes the formation of diaryl ether **3** *via* Pd-catalysed C-O cross-coupling.

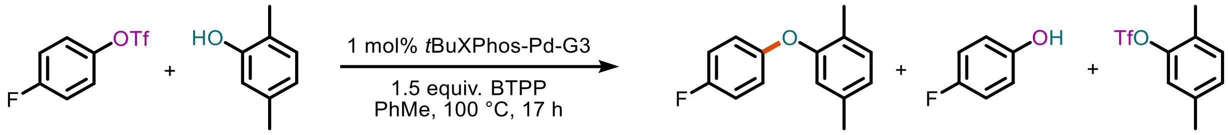

1b **2a** (1.2 eq.) **3** **2b** **1c**

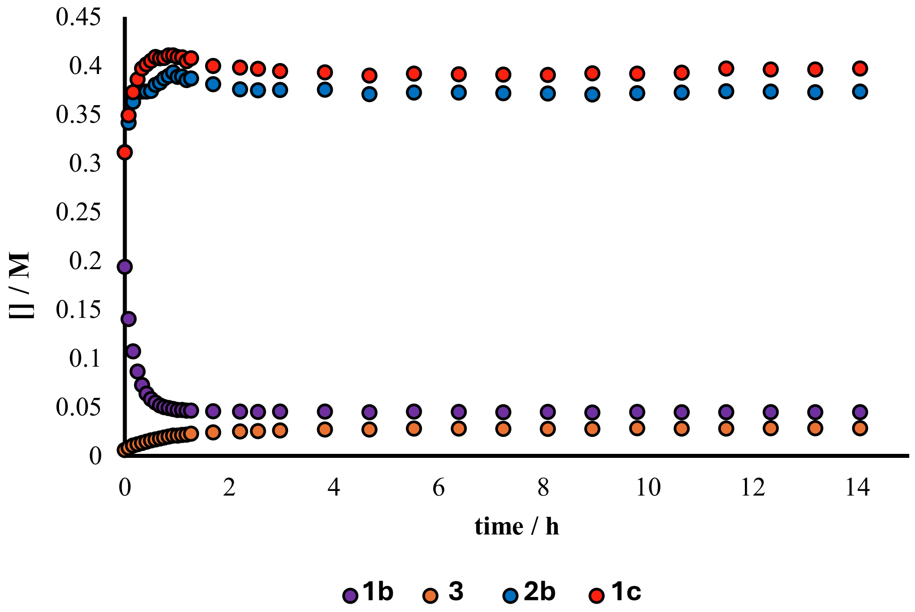

Figure S1. *Representative reaction profile for the triflyl metathesis in the presence of Pd. Reaction was monitored by in situ ^{19}F NMR spectroscopy.*

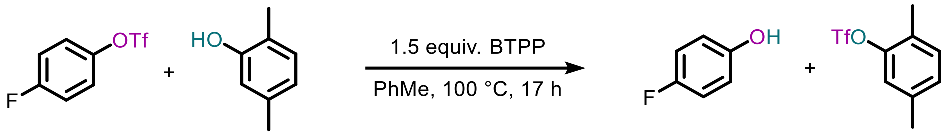

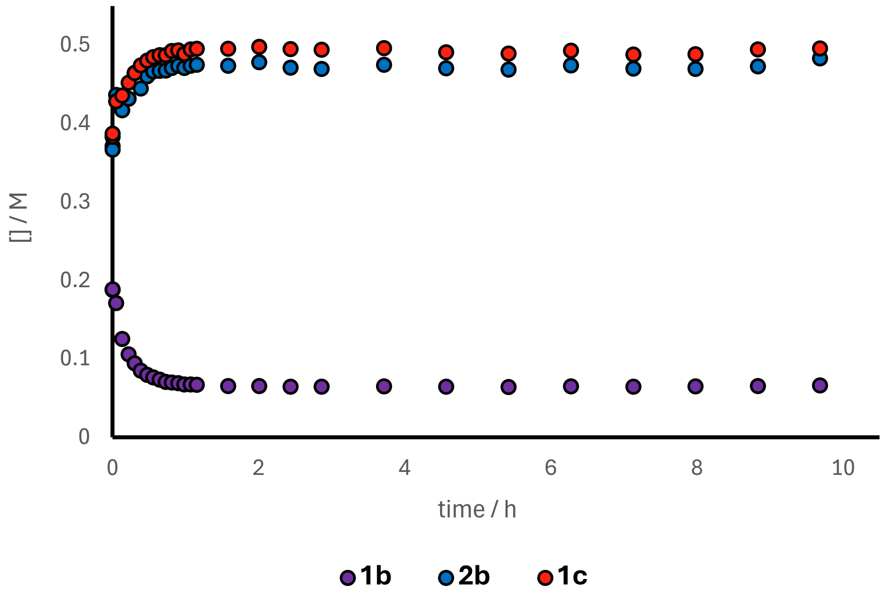

Figure S2. *Representative reaction profile for the triflyl metathesis without Pd precatalyst. Reaction was monitored by in situ ${}^{19}F$ NMR spectroscopy.*

Experimental Procedure. 4-Fluorophenyl triflate (122.1 mg, 0.50 mmol) and PMP (116.5 mg, 0.75 mmol) were added to a flame-dried 1 mL volumetric flask, then the volume was made up to 1 mL with anhydrous PhMe/PhMe-d_8 (3:1). Once all the components were added, the volumetric flask was assembled with a vacuum adapter and connected to the Schlenk manifold. The stock solution was degassed *via* freeze-pump-thaw (total of four cycles; the headspace of the flask was evacuated for three minutes per cycle). When necessary, the solution was topped up with anhydrous, degassed PhMe to the 1 mL mark.

2,5-Dimethylphenol (36.7 mg, 0.300 mmol), 4,4'-bis(trifluoromethyl)biphenyl (internal standard for ${}^{19}F$ NMR spectroscopy) and – where used – *t*BuXPhos-Pd-G3 (1.99 mg, 0.0025 mmol) were added to an NMR tube. The tube was evacuated, then backfilled with N_2 (total of four cycles). An aliquot of the stock solution (500 µL, 0.5 M) was then added to the NMR tube under N_2 atmosphere. Once sealed, the tube was shaken vigorously for 1 minute to ensure the homogeneity of the reaction mixture.

The Standard Reference NMR Sample was inserted to the spectrometer at room temperature. The instrument was locked on DMSO-d_6, then shimmed and tuned to ${}^{1}H$ frequency. The temperature was then gradually raised to 100 °C; once the target temperature was reached, at least four ${}^{1}H$

NMR spectra were recorded to confirm temperature stability. The Reference Sample was then ejected and replaced with the NMR tube containing the reaction mixture, and a stopwatch was started. The instrument was locked on PhMe-d_8, then shimmed and tuned to ^{19}F frequency. Once the temperature had stabilised back at 100 °C, a first spectrum was acquired. The kinetics experiment was then commenced, and NMR spectra were recorded until analysis was complete.

The spectra were stacked and bulk-processed. Baseline correction was applied and the phase was usually corrected manually. Each peak was integrated and concentrations of all products of interest were calculated in Excel by comparison to the 4,4'-bis(trifluoromethyl)biphenyl internal standard integral.

*Characterisation data for triflyl migration product **1c** are presented in Section 2.1.*

3.4 Ligand Screening (Manuscript Scheme 2)

Preparation of Stock Solutions

<u>*μ*OMs-Pd-G3 dimer stock solution (A).</u> *μ*OMs-Pd-G3 dimer (14.8 mg, 0.02 mmol) was added to a 1 mL volumetric flask. MeCN was added to give 1 mL of a 0.02 M stock solution, which was used immediately.

<u>Reagent stock solution (B).</u> *One stock solution was prepared for each base to be investigated.* 2,5-Dimethylphenol (366 mg, 3.0 mmol) and internal standard (4,4'-bis(trifluoromethyl)biphenyl) were added to a flame-dried Schlenk flask. The vessel was sealed with a rubber septum, then evacuated and backfilled with N_2 (total of three cycles). Anhydrous PhMe (5.0 mL) was then added under N_2, followed by 4-fluorophenyl triflate (610 mg, 2.5 mmol) and the appropriate organic base DIPEA, BIMA or PMP; 3.75 mmol).

Experimental Procedure for Ligand Screening

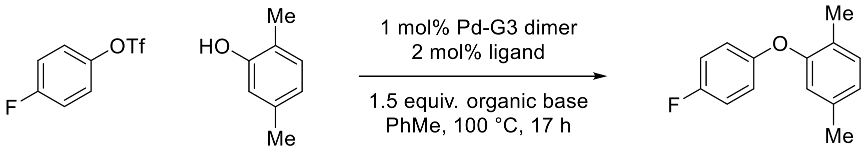

1b **2a** (1.2 equiv.) **3**

Screening reactions were performed on 0.2 mmol scale using a Radleys GreenHouse Plus Parallel Synthesiser (Figure S3).

*μ*OMs-Pd-G3 dimer *stock solution A* (0.02 M; 100 μL, 0.002 mmol; 2 mol% Pd) was added into each oven-dried reaction tube (equipped with a magnetic stir bar). The tubes were left open to allow solvent evaporation, before vacuum was applied to ensure dryness. The appropriate ligand (0.004 mmol; 2 mol%) was then added to each tube. The tubes were placed in the parallel synthesiser and the chamber was sealed, then evacuated and backfilled with N_2 (total of three cycles). The reagent *stock solution B* (400 μL) was added under N_2 to each tube. The reaction mixtures were stirred at 100 °C for 17 h before being quenched with acetic acid (10 μL). An aliquot (100 μL) was then removed and transferred into an NMR tube containing $CDCl_3$ (500 μL) for analysis by quantitative ^{19}F NMR spectroscopy. The procedure was repeated for each combination of ligand and organic base.

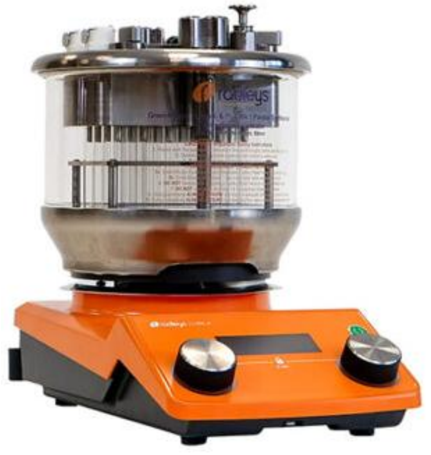

Figure S3. *Radleys GreenHouse Plus Parallel Synthesiser.*

The screening results are presented in Manuscript Scheme 2.

4. Mechanistic Investigations

4.1 Phenol / PMP Titration (Manuscript Fig. 1, Panel B)

Data Acquisition & Fitting

Binding isotherm constant, K_{assoc}, and predicted chemical shifts were determined using the procedure reported by Macomber.[37] ^{1}H NMR data (NS= 16, DS = 0, D1 = 1 sec) were recorded on a Bruker Avance 600 MHz NMR spectrometer at 100 °C.

Experimental Procedure for Phenol / PMP Titration

2,5-Dimethylphenol (18.3 mg, 0.15 mmol) was added to an NMR tube followed by PhMe-d_8 (500 µL). The tube was sealed and shaken vigorously for 1 minute to ensure sample homogeneity. A stock solution of PMP was prepared by adding PMP (905 µL, 5.00 mmol) to a 1 mL volumetric flask, before the volume was made up to 1.00 mL with PhMe-d_8.

the sample of 2,5-dimethylphenol in PhMe-d_8 was analysed by ^{1}H NMR spectroscopy at 100 °C. The sample was then removed from the spectrometer, allowed to cool down, then the first addition of PMP stock solution (5.0 M in PhMe-d_8; 5.0 µL, 0.025 mmol) was made. The sample was resealed and shaken vigorously for 1 minute, then analysed by ^{1}H NMR spectroscopy at 100 °C. Further additions of PMP stock solution were made according to Table S3. The changes in chemical shift for the 6-H and the 2-Me protons were fit using the Microsoft Excel Solver Plug-In, giving K_{assoc} = 0.3 M^{-1} for both environments.

Addition No.	Addition Volume (μL)	δ(6-CH) /ppm		δ(2-Me) /ppm	
		Predicted	Observed	Predicted	Observed
0	0	6.1937	6.1936	2.0248	2.0248
1	5	6.2002976	6.2003	2.0280578	2.0282
2	5	6.2066157	6.2066	2.0311701	2.0308
3	5	6.2126709	6.2127	2.0341462	2.0336
4	5	6.2184785	6.2181	2.0369941	2.0364
5	5	6.2240527	6.2289	2.0397217	2.0413
6	5	6.2294067	6.2286	2.042336	2.0416
7	5	6.2345529	6.2338	2.0448436	2.0442
8	5	6.2395026	6.2386	2.0472506	2.0468
9	5	6.2442665	6.244	2.0495628	2.0485
10	5	6.2488545	6.248	2.0517854	2.0506
11	25	6.2694586	6.2694	2.0617148	2.0628
12	25	6.2868227	6.2872	2.0700154	2.0717
13	50	6.3144228	6.3145	2.0830787	2.0839
14	50	6.33533768	6.3347	2.0928686	2.092
15	50	6.3517092	6.3522	2.1004645	2.1002

Table S3. *Volumes of PMP stock solution added, associated changes in observed chemical shift (^{1}H NMR spectroscopy, 100 °C in PhMe-d_8), and chemical shifts predicted following fitting.*

Phenol Dilution Studies

A stock solution of 2,5-dimethylphenol was prepared by adding the phenol (122.2 mg, 1.00 mmol) to a 1 mL volumetric flask, before the volume was made up to 1.00 mL with PhMe-d_8. The first aliquot of this stock solution (1.00 M in PhMe-d_8; 5.0 µL, 0.005 mmol) was added to an NMR tube containing PhMe-d_8 (500 µL). The tube was sealed and shaken vigorously for 1 minute, then analysed by ^{1}H NMR spectroscopy at 100 °C. Further additions of phenol stock solution were made according to Table S4.

Addition No.	Addition Vol. /µL	Total Vol. /µL	[ArOH] /M	δ(6-CH) /ppm	δ(OH) /ppm	δ(2-Me) /ppm
0	0	500	0	0	0	0
1	5	505	0.00990099	6.1821	3.7656	2.0199
2	5	510	0.019607843	6.1844	3.7668	2.02
3	5	515	0.029126214	6.1849	3.7673	2.0201
4	10	525	0.047619048	6.1846	3.7765	2.0206
5	10	535	0.065420561	6.187	3.7796	2.0209
6	10	545	0.082568807	6.1901	3.7849	2.0214
7	20	565	0.115044248	6.1905	3.7946	2.0219
8	20	585	0.145299145	6.1915	3.8054	2.0225
9	30	615	0.18699187	6.1945	3.8193	2.0235
10	40	655	0.236641221	6.1962	3.8342	2.0242
11	50	705	0.290780142	6.1992	3.8522	2.0254

Table S4. *Volumes of 2,5-dimethylphenol stock solution added, and associated changes in observed chemical shift (^{1}H NMR spectroscopy, 100 °C in PhMe-d_8).*

Simulation of the Change in Phenol-PMP Complex During C-O Cross-Coupling

The instantaneous concentrations ($[\ ]_{conv}$) of aryl triflate, phenol, and PMP were calculated from the % conversion of aryl triflate and the initial concentrations of each species, in increments of 5% conversion:

$[Ar'OTf]_{conv} = [Ar'OTf]_0 – ([Ar'OTf]_0 \times (conversion/100))$ *Where:* $[Ar'OTf]_0 = 0.5$ M

$[ArOH]_{conv} = [Ar'OTf]_{conv} + [ArOH]_0 - [Ar'OTf]_0$ $[ArOH]_0 = 0.6$ M

$[PMP]_{conv} = [Ar'OTf]_{conv} + [PMP]_0 - [Ar'OTf]_0$ $[PMP]_0 = 0.75$ M

The instantaneous concentrations were then used to calculate the predicted ^{1}H NMR chemical shift of the phenol 2-Me protons (δ_{pred}) using the method of Macomber,[37] the value of K_{assoc} (0.3 M^{-1}) obtained from fitting the ArOH-PMP titration data, and the ^{1}H NMR chemical shifts the phenol 2-Me measured in the absence of PMP (δ_H = 2.0248 ppm), and predicted at full complexation (δ_H = 2.3208 ppm). The following values were then calculated:

ArOH found as ArOH-PMP complex, as a percentage of the total amount of phenol remaining at a given conversion of Ar'OTf:

$$\%\ ArOH\ as\ \{PMP•HOAr\},\ wrt\ [ArOH]_{conv} = 100 \times (\delta_{pred} – 2.0248) / (2.3208 – 2.0248)$$

ArOH found as ArOH-PMP complex, as a percentage of the initial amount of phenol added at the start of the reaction:

$$\%\ ArOH\ as\ \{PMP•HOAr\},\ wrt\ [ArOH]_0 = [\{PMP•HOAr\}]_{conv} / [ArOH]_0$$

The data are presented in Table S5 and Figures S4-S5.

% conv Ar′OTf	[ ]$_{conv}$ /M			δ_{pred} /ppm	% ArOH as {PMP•HOAr}, wrt $[ArOH]_{conv}$	[{PMP•HOAr}] /M	% ArOH as {PMP•HOAr}, wrt $[ArOH]_0$
	Ar′OTf	ArOH	PMP				
0	0.5	0.6	0.75	2.0732135	16.35701	0.098142	16.35701
5	0.475	0.575	0.74	2.0728855	16.24618	0.093416	15.56925
10	0.45	0.55	0.73	2.0725535	16.13404	0.088737	14.78953
15	0.425	0.525	0.72	2.0722177	16.02056	0.084108	14.01799
20	0.4	0.5	0.71	2.0718778	15.90571	0.079529	13.25476
25	0.375	0.475	0.7	2.0715337	15.78947	0.075	12.5
30	0.35	0.45	0.69	2.0711854	15.67181	0.070523	11.75386
35	0.325	0.425	0.68	2.0708329	15.55269	0.066099	11.01649
40	0.3	0.4	0.67	2.0704759	15.43208	0.061728	10.28805
45	0.275	0.375	0.66	2.0701144	15.30995	0.057412	9.568721
50	0.25	0.35	0.65	2.0697484	15.18627	0.053152	8.858659
55	0.225	0.325	0.64	2.0693776	15.061	0.048948	8.158043
60	0.2	0.3	0.63	2.069002	14.93411	0.044802	7.467054
65	0.175	0.275	0.62	2.0686215	14.80555	0.040715	6.785878
70	0.15	0.25	0.61	2.068236	14.6753	0.036688	6.114707
75	0.125	0.225	0.6	2.0678453	14.5433	0.032722	5.453738
80	0.1	0.2	0.59	2.0674493	14.40952	0.028819	4.803175
85	0.075	0.175	0.58	2.067048	14.27392	0.024979	4.163227
90	0.05	0.15	0.57	2.0666411	14.13645	0.021205	3.534113
95	0.025	0.125	0.56	2.0662286	13.99707	0.017496	2.916056
100	0	0.1	0.55	2.0658102	13.85573	0.013856	2.309288

Table S5. *Simulated change in the amount of phenol-PMP complex during C-O cross-coupling.*

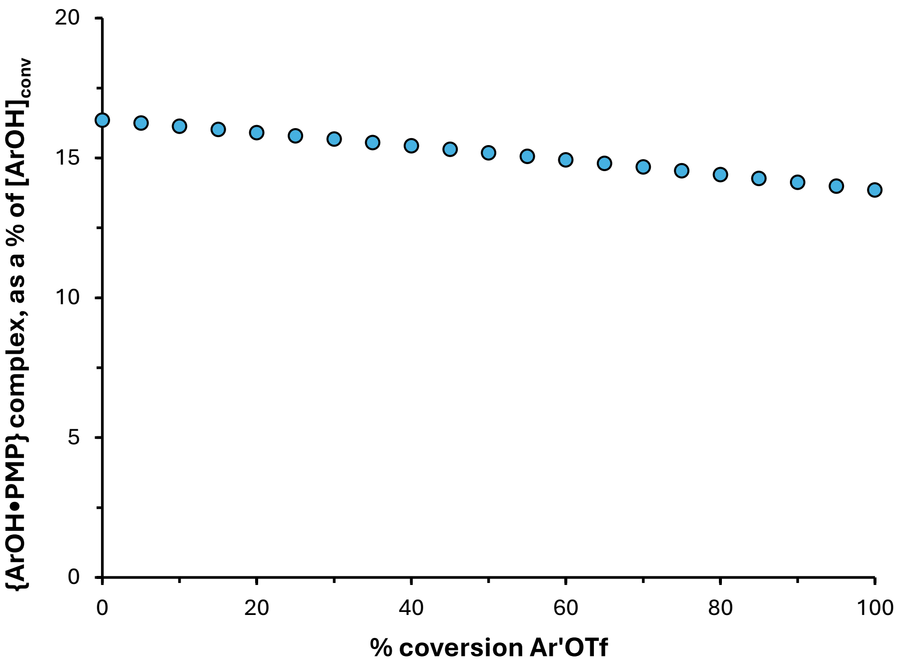

Figure S4. *Predicted partitioning of 2,5-dimethylphenol into the phenol-PMP complex during cross-coupling, as a function of reaction conversion.*

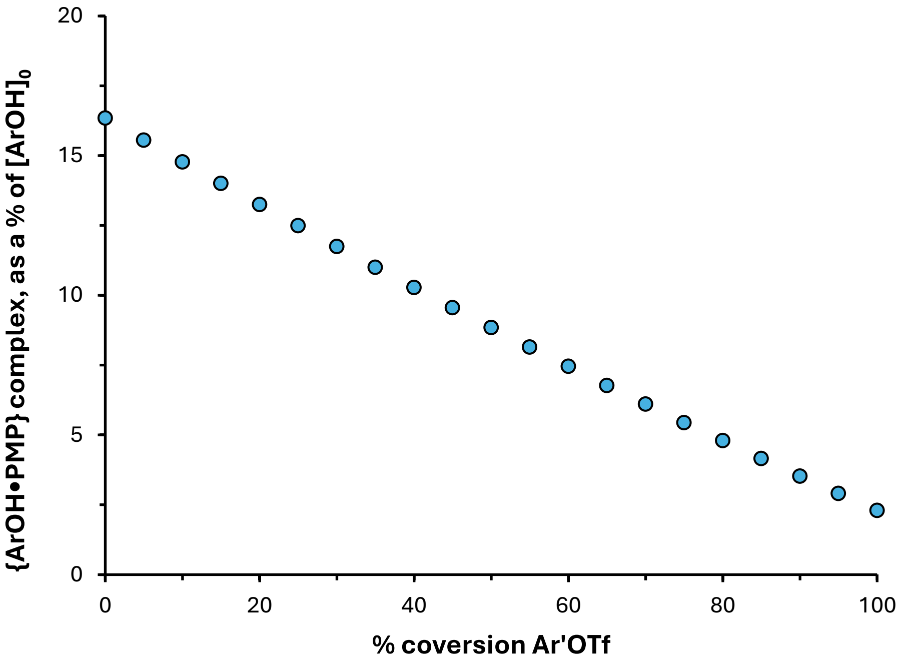

Figure S5. *Predicted partitioning of 2,5-dimethylphenol into the phenol-PMP complex during cross-coupling, as a function of reaction conversion.*

4.2 Reaction Rate Law (Manuscript Fig. 1, Panel C)

General Information for Reaction Monitoring Experiments

NMR spectra were recorded on a Bruker Avance 600 MHz NMR spectrometer at 100 °C, and processed using MestReNova. ^{1}H NMR chemical shifts are reported in parts per million (ppm) relative to the residual solvent signals. ^{19}F NMR chemical shifts are reported in ppm relative to an internal standard (4,4'-bis(trifluoromethyl)biphenyl; δ = -62.40 ppm in PhMe-d_8). The temperature was calibrated using the Standard Reference Sample for High Temperature (300 K - 380 K) Calibration (80% glycol in DMSO-d_6). Calibration was performed *via* ^{1}H NMR spectroscopy (NS = 1, DS = 0). Parameters for *in situ* reaction monitoring *via* ^{19}F NMR spectroscopy are as follows: NS = 4, DS = 0, D1 = 30 sec.

Reactions were performed in a mixture of 50:50 PhMe-d_8 and non-deuterated anhydrous PhMe. PhMe-d_8 (Fluorochem) was distilled from CaH_2 (Sigma Aldrich).

General Procedure for Reaction Monitoring

<u>Aryl triflate / PMP stock solution:</u> 4-Fluorophenyl triflate (122.1 mg, 0.50 mmol) and PMP (136 µL, 0.75 mmol) were added to a flame-dried 1 mL volumetric flask fitted with a vacuum adapter, which was made up to 1.00 mL with anhydrous PhMe (1:1 d_0: d_8). The stock solution was then degassed *via* freeze-pump-thaw (total of four cycles).

To an NMR tube in the following order were added: *µ*OMs-Pd-G4 dimer•1/5Et_2O (3.9 mg, 0.005 mmol, 4 mol% Pd), AdBippyPhos (13.26 mg, 0.020 mmol, 8 mol%), 2,5-dimethylphenol (36.7 mg, 0.300 mmol) and 4,4'-bis(trifluoromethyl)biphenyl (internal standard for ^{19}F NMR spectroscopy). The tube was evacuated, then backfilled with N_2 (total of four cycles). An aliquot of the stock solution ($[ArOTf]_0$ = 0.5 M; 500 µL, 0.250 mmol ArOTf) was then added to the NMR tube under N_2 atmosphere. Once sealed, the tube was shaken vigorously for 1 minute.

The Standard Reference Sample was inserted to the spectrometer at room temperature. The instrument was locked on DMSO-d_6, then shimmed and tuned to ^{1}H frequency. The temperature was then gradually raised to 100 °C; once the target temperature was reached, at least four ^{1}H NMR spectra were recorded to confirm temperature stability. The Reference Sample was then ejected and replaced with the NMR tube containing the reaction mixture, and a stopwatch was started. The instrument was locked on PhMe-d_8, then shimmed and tuned to ^{19}F frequency. Once the temperature had stabilised back at 100 °C (after 7 – 9 minutes), a first spectrum was acquired. The kinetics experiment was then commenced, and NMR spectra were recorded.

The spectra were stacked and bulk-processed. Baseline correction was applied and the phase was usually corrected manually. Each peak was integrated and concentrations of all species of interest were calculated by comparison to the integral of the 4,4'-bis(trifluoromethyl)biphenyl internal standard.

Determination of Reaction Rate Law

Reaction monitoring revealed a *pseudo*-zeroth order profile (Figure S6). As such, only the dependence of reaction rate on the concentration of Pd precatalyst was investigated:

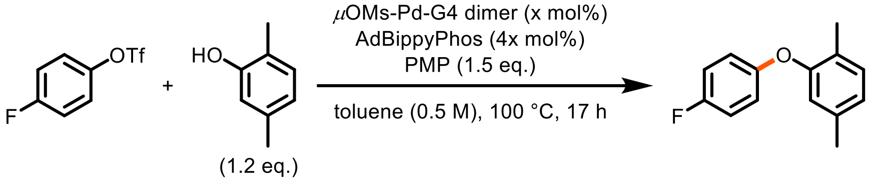

The experiments were set up and monitored according to *the General Procedure for Reaction Monitoring* (Section 4.2). All reactions were carried out on a 0.250 mmol scale, using the quantities of Pd precatalyst and AdBippyPhos indicated in Table S6. The resulting reaction profiles are presented in Figure S6, and the dependence of rate on $[\mathrm{Pd}]_{total}$ is presented in Figure S7. The observed pseudo-order behaviour (Figure S6), and the first order dependence of rate on $[\mathrm{Pd}]_{total}$ (Figure S7), are consistent with the following rate law:

$$\frac{d[\mathrm{ArOTf}]}{dt} = k[\mathrm{Pd}]_{total}$$

entry	reagent	mol%	mmol	amount /mg
**1**	μOMs-Pd-G4 dimer•1/5Et_2O	1	0.0025	1.95
	AdBippyPhos	4	0.010	6.63
**2**	μOMs-Pd-G4 dimer•1/5Et_2O	2	0.005	3.90
	AdBippyPhos	8	0.020	13.26
**3**	μOMs-Pd-G4 dimer•1/5Et_2O	4	0.010	7.81
	AdBippyPhos	16	0.040	26.51

Table S6. *Quantities of μOMs-Pd-G4 dimer and AdBippyPhos ligand used during rate law determination studies.*

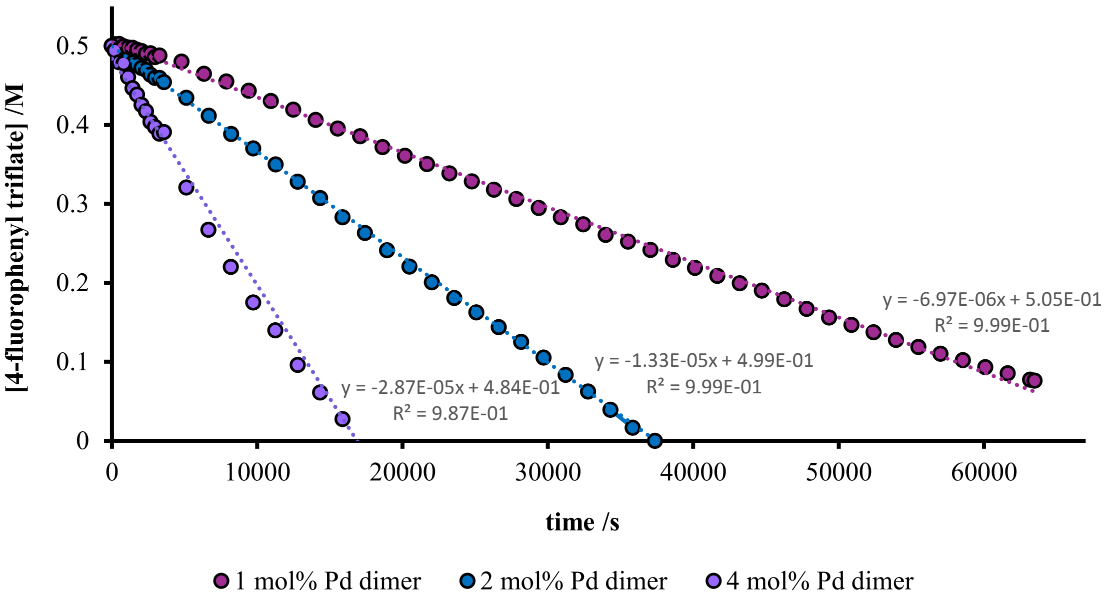

Figure S6. *Reaction profiles for coupling of 2,5-dimethylphenol and 4-fluorophenyl triflate at different Pd-loadings. Monitored by quantitative ^{19}F NMR spectroscopy vs internal standard.*

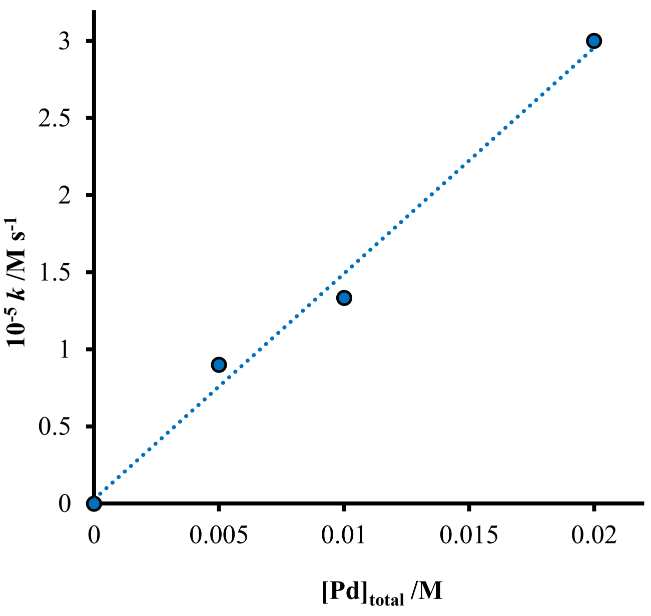

Figure S7. *First order dependence of reaction rate on catalyst concentration.*

Identification of Catalyst Resting State

An intermediate was observed by ^{19}F NMR spectroscopy (δ_F = -122 ppm; 100 °C, PhMe-d_8) when monitoring the coupling of 4-fluorophenyl triflate and 2,5-dimethylphenol (representative reaction profile shown in Figure S8). This intermediate was tentatively identified as a catalyst resting state because it grows in concentration at the start of the coupling reaction, remains at an approximately constant concentration (commensurate with the catalyst loading) across the steady-state phase of catalysis, and is consumed at the end of the coupling.

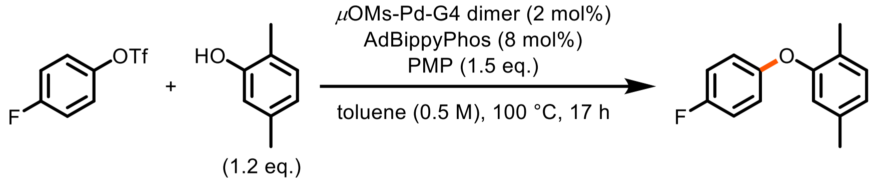

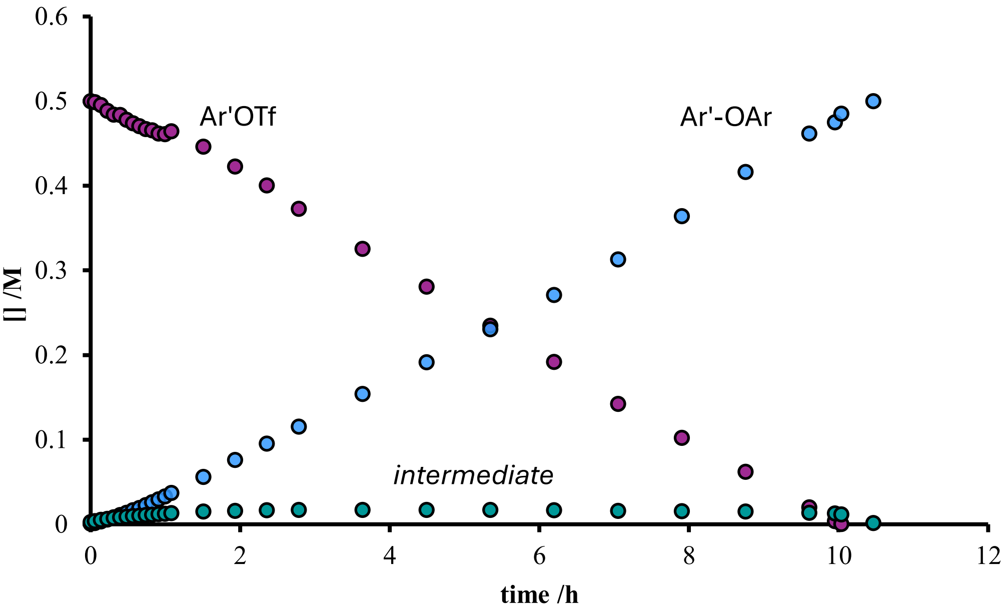

Figure S8. *Representative reaction profile showing the presence of an intermediate (monitored by quantitative ^{19}F NMR spectroscopy, as per the General Procedure for Reaction Monitoring).*

Commentary. The identity of the reaction intermediate was confirmed to be the arylpalladium phenoxide complex (AdBippyPhos)Pd(Ar')(OAr) through stoichiometric studies (*experimental details are provided below*): The oxidative addition complex (AdBippyPhos)Pd^{II}(Ar')OTf was prepared, and its ^{19}F NMR chemical shifts in toluene-d$_8$ were obtained (Figure S9A). Addition of 2,5-dimethylphenol to the NMR tube resulted in a change in chemical shift from -119.05 ppm to -118.23 ppm (Figure S9B), indicating a complete consumption of the oxidative addition complex and suggesting formation of the phenol-bound Pd(II) intermediate. Next, the addition of PMP resulted in formation of a sharp signal at -121.98 ppm (Figure S9C), which is the same chemical shift that is observed under catalytic conditions. Finally, heating the mixture at 100 °C for 1.5 h

initiated the reductive elimination, giving a resonance at -121.53 ppm, which was confirmed to belong to the diaryl ether coupling product by comparison to an authentic sample.

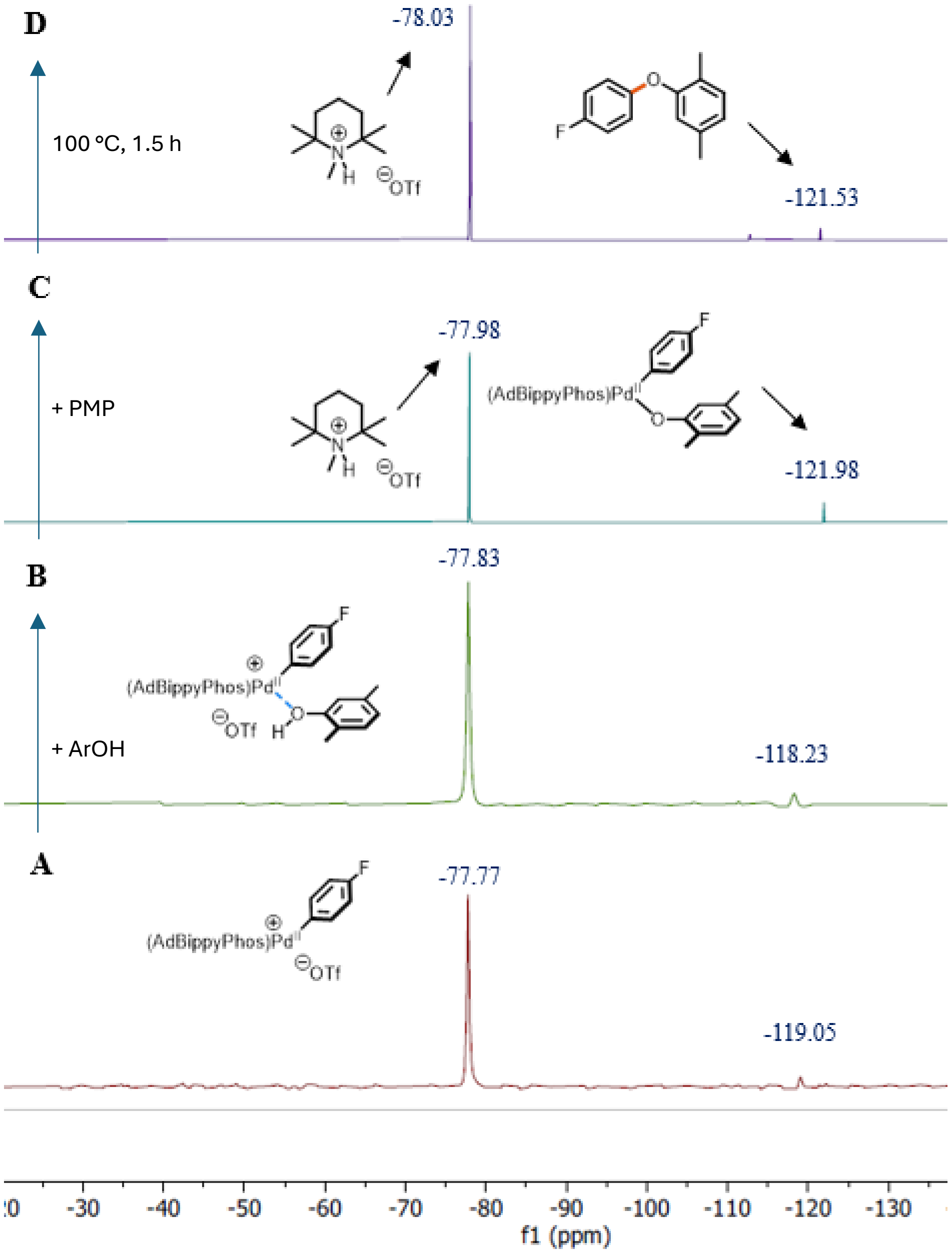

Figure S9. *^{19}F NMR spectra (PhMe-d8, 25 °C) of stoichiometric studies identifying the reaction intermediate as (AdBippyPhos)Pd(Ar')(OAr). (A) oxidative addition complex; (B) phenol-bound Pd(II) complex; (C) catalyst resting state; (D) diaryl ether product formation.*

Experimental Procedures. Parameters for quantitative ^{19}F NMR spectroscopy are as follows: NS = 8, DS = 0, D1 = 30 sec. PhMe-d_8 (Fluorochem) was distilled from CaH_2 (Sigma Aldrich) and degassed *via* freeze-pump-thaw (total of four cycles).

(AdBippyPhos)$Pd^{II}(C_6H_4F)OTf$ complex (12.0 mg, 0.0119 mmol; preparative procedure given below) was added to an NMR tube; the tube was evacuated, then backfilled with N_2 (total of four cycles). Anhydrous, degassed PhMe-d_8 (600 µL) was added under N_2. Once sealed, the tube was shaken vigorously for 2 minutes. The resulting mixture was analysed by quantitative ^{19}F NMR spectroscopy (Table S7, entry 1). Once the tube was placed back under inert atmosphere, 2,5-dimethylphenol (7.2 mg, 0.0593 mmol) was added, the tube was sealed and shaken vigorously for 2 minutes, and the sample was analysed by quantitative ^{19}F NMR spectroscopy (Table S7, entry 2). The NMR tube was again placed under N_2 atmosphere and PMP (10.8 µL, 0.0593 mmol) was added. Once sealed, the tube was shaken vigorously for 2 minutes, then analysed by quantitative ^{19}F NMR spectroscopy (Table S7, entry 3). Finally, the tube was placed in pre-heated heating block and was left at 100 °C for 1.5 h. Once cooled own, the final quantitative ^{19}F NMR data were obtained (Table S7, entry 4).

Entry	treatment	δ_F(OTf) /ppm	$\delta_F(C_6H_4F)$ /ppm
1	none	-77.77	-119.05
2	2,5-dimethyl phenol added	-78.83	-118.28
3	PMP added	-77.98	-121.98
4	heated at 100 °C for 1.5 h	-78.03	-121.53

Table S7. *Changes in ^{19}F NMR chemical shifts during catalyst resting state investigation.*

^{DMP}DAB-Pd-BTSM

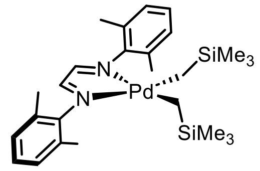

Prepared according to literature procedure from (COD)$PdCl_2$;[38] characterisation data were consistent with literature values: ^1H, ^{13}C NMR and HRMS.[38]

^1H NMR (400 MHz, C_6D_6): δ 6.97–6.95 (m, 6H), 6.88 (s, 2H), 2.18 (s, 12H), 0.44 (s, 4H), 0.23 (s, 18H).

$^{13}C\{^1H\}$ NMR (101 MHz, C_6D_6): δ 160.1, 147.6, 128.6, 128.5, 126.6, 18.8, 4.1, 0.3.

HMRS (ESI$^+$, *m/z*): calcd. for $C_{50}H_{51}FN_4PPd$ [^{DMP}DAB-Pd(CH_2SiMe_3)]$^+$ (ionised through protonolysis of one -CH_2SiMe_3 ligand): 457.1286 [M]$^+$. Found: 457.1287.

ν̃(ATR)/cm^{-1} (neat): 2942, 1537, 1236, 1195, 818, 770, 672.

(AdBippyPhos)Pd^{II}(C_6H_4F)(OTf)

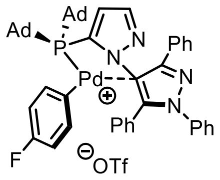

Prepared according to adapted literature procedure.[38] To a flame-dried microwave vial equipped with a magnetic stir bar in a following order were added: ^{DMP}DAB-Pd-BTSM (43.6 mg, 0.08 mmol), AdBippyPhos (53.0 mg, 0.08 mmol), CPME (2 mL, 0.04 M) and **2** (26.6 µL, 0.16 mmol). The vial was then sealed, attached to a Schlenk manifold and the reaction mixture was degassed by slowly opening to the vacuum until a noticeable first gas bubble, then immediately backfilling with N_2. The evacuation/backfilling process was then repeated two more times. The mixture was then stirred at 50 °C for 24 h. Once cooled down, the CPME was removed *in vacuo*. The crude solid was then washed with Et_2O until the washings became colourless. The residual solvent was removed under vacuum to afford the title compound (36.5 mg, 0.04 mmol, 50%) as light brown solid.

^{1}H NMR (500 MHz, $CDCl_3$): δ 8.19 (d, J = 2.2 Hz, 1H), 7.59 (s, br. 2H), 7.54 (dd, J = 7.8, 2.5 Hz, 2H), 7.51 – 7.46 (m, 2H), 7.45 – 7.36 (m, 6H), 7.31 (s, 1H), 7.30 (d, J = 2.5 Hz, 2H), 7.09 (d, J = 45.7 Hz, 2H), 6.81 – 6.75 (m, 2H), 6.74 (d, J = 2.2 Hz, 1H), 1.85 (s, 3H), 1.68 (s, 3H), 1.60 – 1.40 (m, 18H), 1.32 (d, J = 12.5 Hz, 3H), 1.23 – 1.15 (m, 3H).

^{13}C{^{1}H} NMR (126 MHz, $CDCl_3$): δ 162.1 (d, $J_{C\text{-}F}$ = 245.1 Hz), 154.5, 148.2, 144.5 (d, $J_{C\text{-}P}$ = 6.1 Hz), 139.5 (d, $J_{C\text{-}P}$ = 39.1 Hz), 138.5, 138.2, 137.0, 130.9, 130.1, 130.0, 129.1, 129.0, 128.6, 127.5, 126.3, 121.5 (q, $J_{C\text{-}F}$ = 320.5 Hz), 116.6, 115.1, 107.9, 43.1 (t, $J_{C\text{-}P}$ = 17.6 Hz), 40.1, 39.0, 35.8 (d, $J_{C\text{-}P}$ = 6.8 Hz), 28.6 (d, $J_{C\text{-}P}$ = 8.9 Hz), 28.4 (d, $J_{C\text{-}P}$ = 8.9 Hz).

** Three ^{13}C NMR signals in the aromatic region could not be resolved.*

^{31}P{^{1}H} NMR (162 MHz, $CDCl_3$): δ 39.50.

^{19}F NMR (377 MHz, $CDCl_3$): δ -78.17 (s, 3F), -117.97 (app. s, br. 1F).

^{19}F NMR (377 MHz, PhMe-d_8): δ -77.77 (s, 3F), -119.05 (app. s, 1F).

HMRS (ESI$^+$, *m/z*): calcd. for $C_{50}H_{51}FN_4PPd$ [(AdBippyPhos)Pd(^{F}Ar)]$^+$ (ionised by OTf ion loss): 863.2865 [M]$^+$. Found: 863.2885.

HMRS (ESI$^-$, *m/z*): calcd. for CF_3O_3S [OTf]$^-$: 148.9526 [M]$^-$. Found: 148.9529.

ν̃(ATR)/cm^{-1} (neat): 2902, 1498, 1475, 1317, 1228, 1202, 1026, 1009, 720, 631.

4.3 Absolute Rates *vs* Aryl Triflate (Manuscript Fig. 2, Panel A)

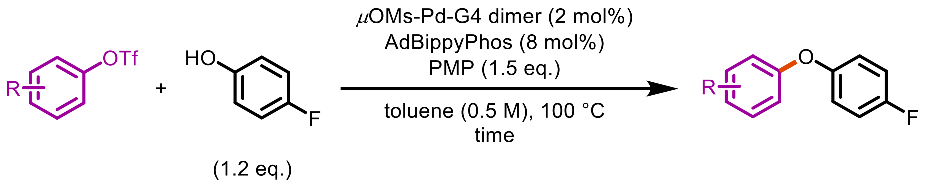

The experiments were set-up and monitored according to the *General Procedure for Reaction Monitoring*, modified according to the physical states of the coupling partners: 4-Fluorophenol (33.6 mg, 0.300 mmol) was added directly to an NMR tube containing 4,4'-bis(trifluoromethyl)biphenyl (internal standard), μOMs-Pd-G4 dimer•1/5Et_2O (3.9 mg, 0.005 mmol, 4 mol% Pd), and AdBippyPhos (13.26 mg, 0.020 mmol, 8 mol%). A stock solution of each aryl triflate (0.50 M in 1:1 PhMe / PhMe-d_8) was prepared as described in the *General Procedure for Reaction Monitoring*.

Reaction profiles (Figure S10), *pseudo*-zeroth order rate constants (Table S8), and a Hammett plot of the data (Figure S11) are presented below.

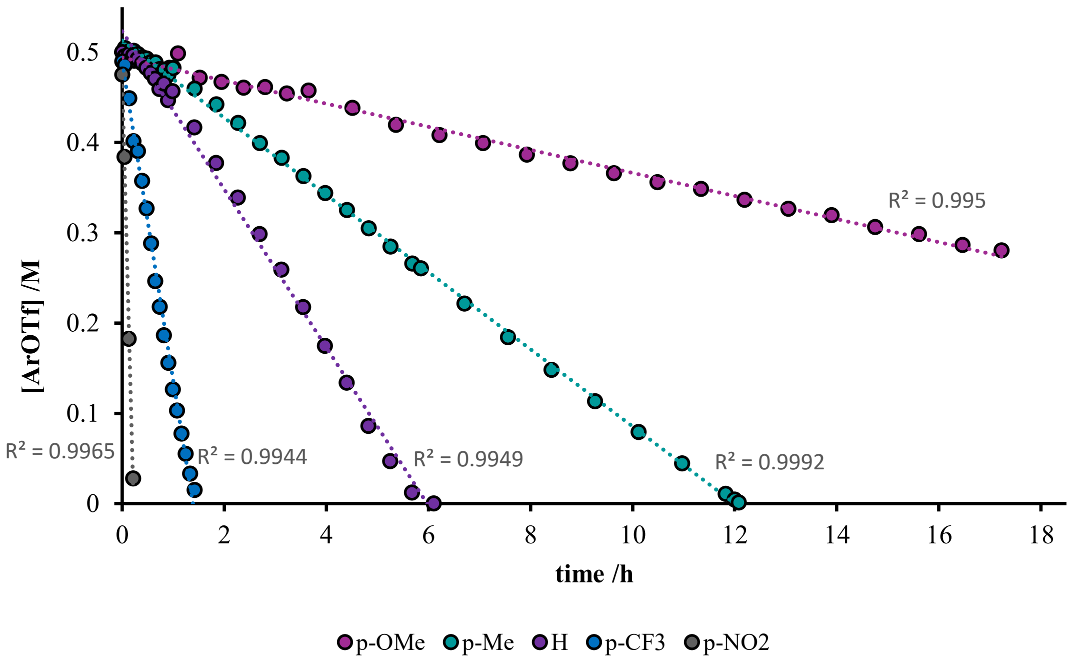

Figure S10. *Reaction profiles for the coupling of different aryl triflates with 4-fluorophenol. Reactions were monitored by in situ* [19]*F NMR spectroscopy.*

Entry	4R-C_6H_4OTf, R =	k /M h^{-1}
**1**	OMe	0.0128
**2**	Me	0.0427
**3**	H	0.0876
**4**	CF_3	0.353
**5**	NO_2	2.1186

Table S8. *Rate constants for the coupling of different aryl triflates with 4-fluorophenol.*

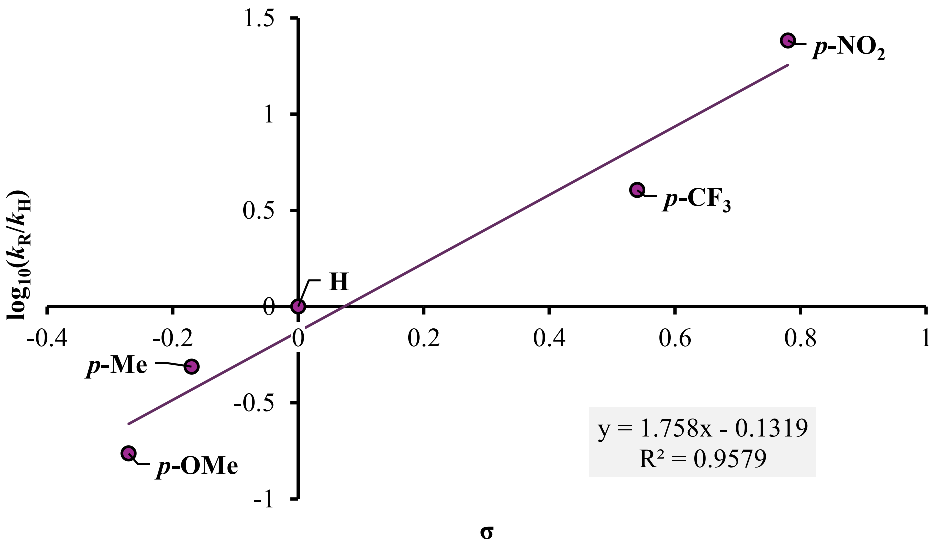

Figure S11. *Hammett plot for the coupling of different aryl triflates with 4-fluorophenol.*

4.4 Absolute Rates *vs* Phenol (Manuscript Fig. 2, Panel B)

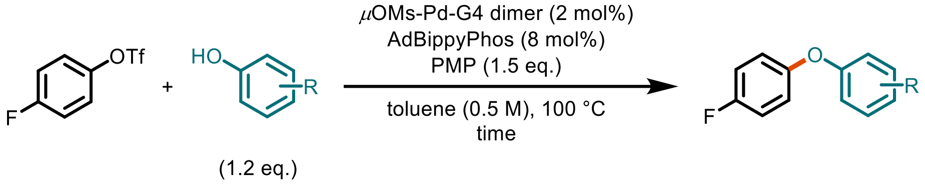

The experiments were set-up and monitored according to the *General Procedure for Reaction Monitoring*, modified according to the physical states of the coupling partners: For solid phenols, the phenol (0.300 mmol) was added directly to an NMR tube containing 4,4'-bis(trifluoromethyl)biphenyl (internal standard), μOMs-Pd-G4 dimer•1/5Et_2O (3.9 mg, 0.005 mmol, 4 mol% Pd), and AdBippyPhos (13.26 mg, 0.020 mmol, 8 mol%). For liquid phenols, the phenol (0.600 mmol) was added to the 1 mL volumetric flask used in the preparation of the 4-fluorophenyl triflate stock solution (see *General Procedure for Reaction Monitoring*), such that [Ar'OTf] = 0.50 M, [ArOH] = 0.6 M, and [PMP] = 0.75 M (in 1:1 PhMe / PhMe-d_8).

Reaction profiles (Figure S12), *pseudo*-zeroth order rate constants (Table S9), and a Hammett plot of the data (Figure S13) are presented below.

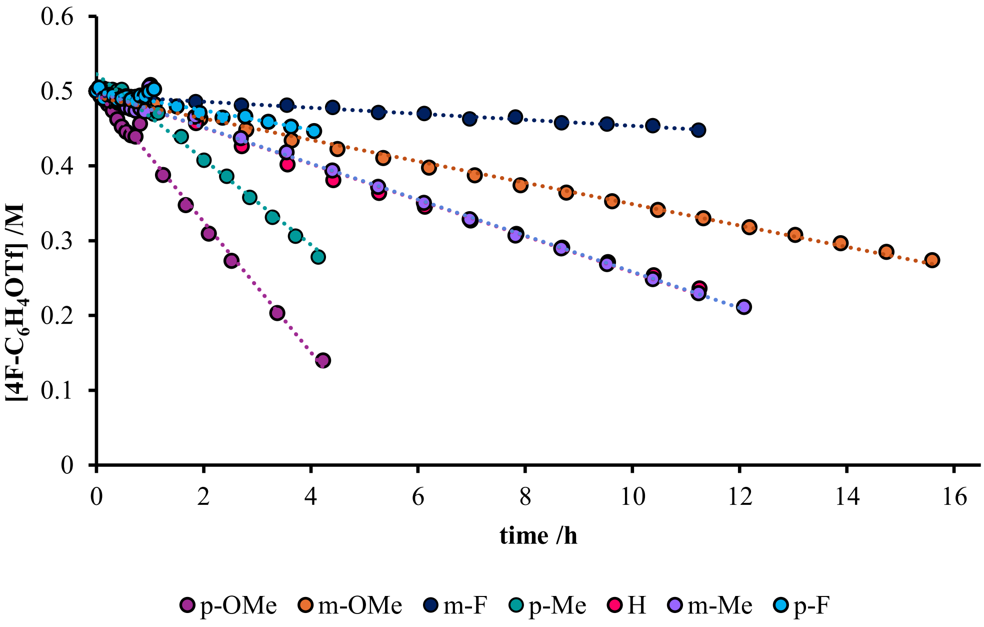

Figure S12. *Reaction profiles for the coupling of 4-fluorophenyl triflate with different phenols. Reactions were monitored by in situ ^{19}F NMR spectroscopy.*

Entry	R-C_6H_4-OH; R =	k /M h^{-1}
**1**	*p*-OMe	0.0872
**2**	*p*-Me	0.057
**3**	*m*-Me	0.0241
**4**	H	0.0242
**5**	*p*-F	0.0129
**6**	*m*-OMe	0.0143
**7**	*m*-F	0.004

Table S9. *Rate constants for the coupling of 4-fluorophenyl triflate with different phenols.*

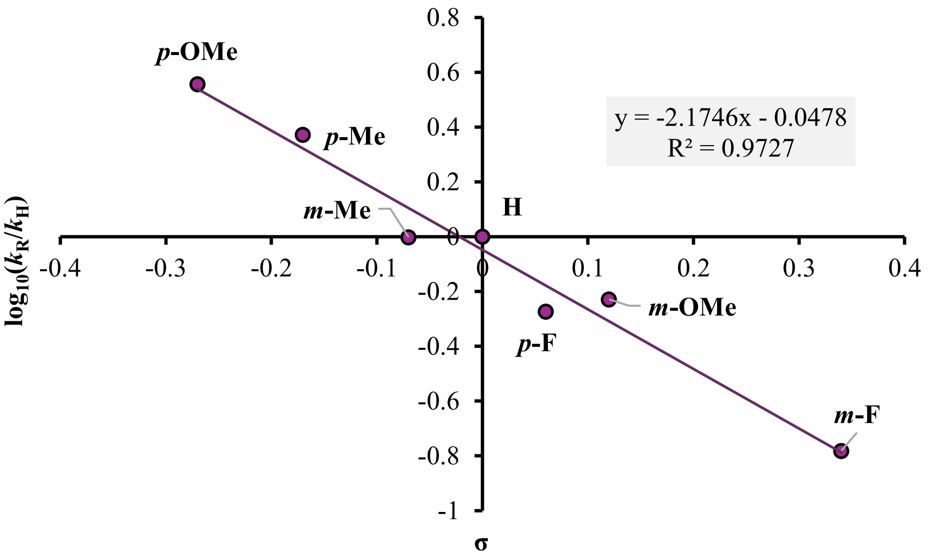

Figure S13. *Hammett plot for the coupling of 4-fluorophenyl triflate with different phenols.*

4.5 Competition Between Aryl Triflates (Manuscript Fig. 2, Panel C)

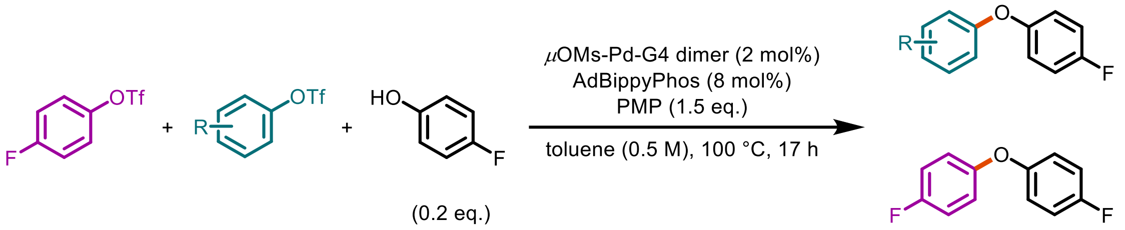

4-Fluorophenyl triflate (41.6 μL, 0.250 mmol), substituted aryl triflate (1.0 eq., 0.250 mmol), 4-fluorophenol (5.6 mg, 0.050 mmol), μOMs-Pd-G4 dimer•MeCN (4.3 mg, 0.005 mmol), AdBippyPhos (13.3 mg, 0.020 mmol), PMP (67.8 μL, 0.375 mmol), internal standard (4,4'-bis(trifluoromethyl)biphenyl), and anhydrous toluene (0.50 mL) were added to a flame-dried microwave vial. The vial was then sealed, attached to a Schlenk manifold and the reaction mixture was degassed by slowly opening to the vacuum until a noticeable first gas bubble, then immediately backfilling with N_2. The evacuation/backfilling process was then repeated four more times. The reactions were stirred at 100 °C for 17 h before being quenched with acetic acid (10 μL) and diluted with DMSO (200 μL). An aliquot (100 μL) was then removed and transferred into an NMR tube containing $CDCl_3$ (500 μL) for analysis by quantitative ^{19}F NMR spectroscopy.

Relative rate constants (Table S10) and a Hammett plot of the data (Figure S14) are given below.

Entry	R	k_R/k_F
**1**	*p*-OMe	0.45
**2**	*p*-Me	1.31
**3**	*m*-Me	2.04
**4**	H	2.01
**5**	*m*-OMe	5.77
**6**	*m*-F	8.29

Table S10. *Relative rate constants for intermolecular competition between R-C_6H_4OTf and 4-FC_6H_4OTf.*

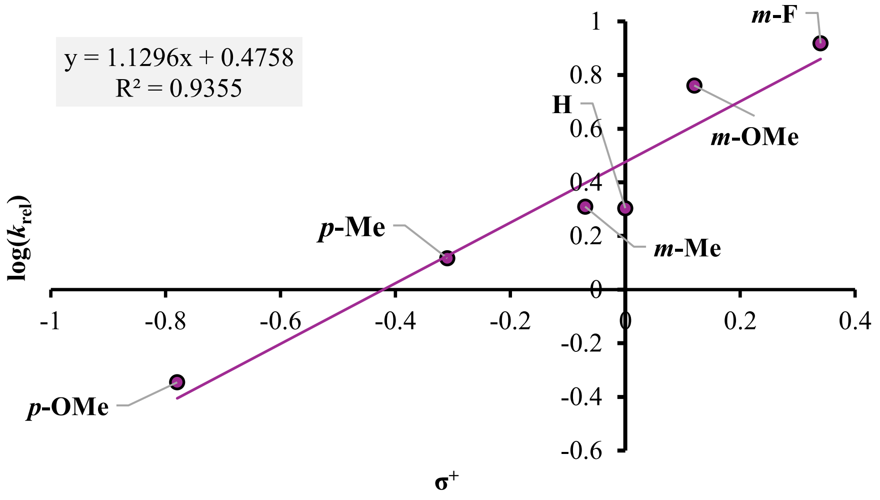

Figure S14. *Hammett plot for intermolecular competition between R-C_6H_4OTf and 4-FC_6H_4OTf.*

4.6 Competition Between Phenols (Manuscript Fig. 2, Panel D)

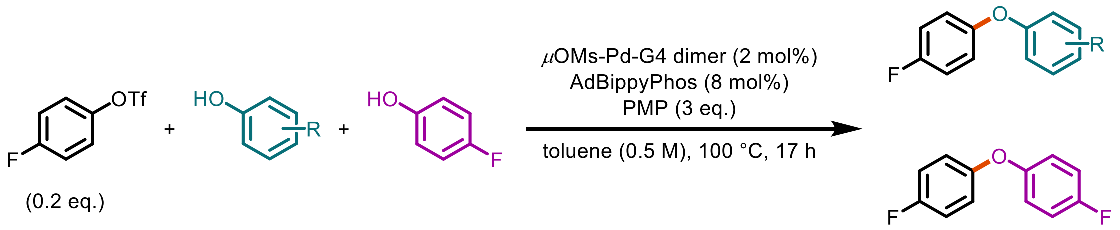

4-Fluorophenyl triflate (8.4 μL, 0.050 mmol), substituted phenol (1.0 eq., 0.250 mmol), 4-fluorophenol (28.0 mg, 0.250 mmol), μOMs-Pd-G4 dimer•MeCN (4.3 mg, 0.005 mmol), AdBippyPhos (13.3 mg, 0.020 mmol), PMP (136.0 μL, 0.750 mmol), internal standard (4,4'-bis(trifluoromethyl)biphenyl), and anhydrous toluene (0.50 mL) were added to a flame-dried microwave vial. The vial was then sealed, attached to a Schlenk manifold and the reaction mixture was degassed by slowly opening to the vacuum until a noticeable first gas bubble, then immediately backfilling with N_2. The evacuation/backfilling process was then repeated four more times. The reactions were stirred at 100 °C for 17 h before being quenched with acetic acid (10 μL) and diluted with DMSO (200 μL). An aliquot (100 μL) was then removed and transferred into an NMR tube containing $CDCl_3$ (500 μL) for analysis by quantitative ^{19}F NMR spectroscopy.

Relative rate constants (Table S11) and a Hammett plot of the data (Figure S15) are given below.

Entry	R	k_R/k_F
**1**	*p*-OMe	1.49
**2**	*p*-Me	1.05
**3**	*m*-Me	0.91
**4**	H	0.68
**5**	*m*-OMe	0.68
**6**	*m*-F	0.53

Table S11. *Relative rate constants for intermolecular competition between R-C_6H_4OH and 4-FC_6H_4OH.*

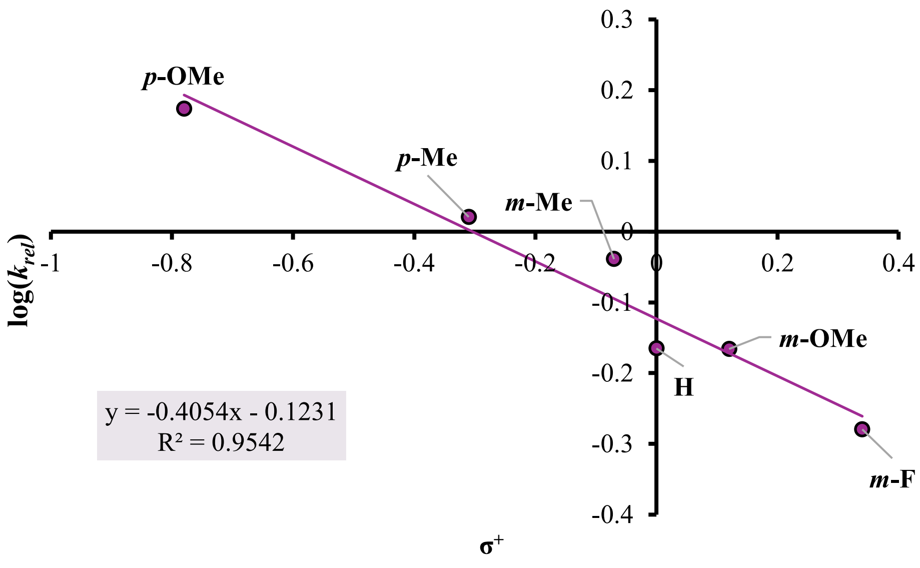

Figure S15. *Hammett plot for intermolecular competition between R-C_6H_4OH and 4-FC_6H_4OH.*

5. Substrate Scope

5.1 Substrate Scope (Manuscript Scheme 3)

General Procedure for C-O Cross-Coupling

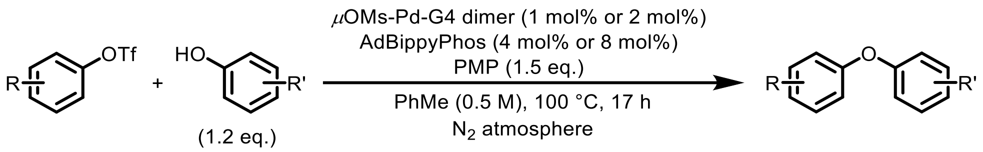

μOMs-Pd-G4 dimer (either 1 mol% or 2 mol%) and AdBippyPhos (4 mol% or 8 mol%; 4 equivalents relative to the amount of μOMs-Pd-G4 dimer used) were added to a flame-dried microwave vial equipped with a magnetic stir bar. Additions of aryl triflate, phenol, PMP and toluene were carried out according to *variation A* or *variation B*. The reaction mixture was stirred at 100 °C for 17 h before being quenched with acetic acid (10 μL). Once cooled to room temperature, DMSO (200 μL) was added to dissolve [PMP-H][OTf] by-product which precipitates at RT. The mixture was then diluted with Et_2O (5 mL), washed with 2 M aq. HCl (10 mL), 2 M aq. NaOH (10 mL), and brine (10 mL). The organic extracts were then dried over anhydrous Na_2SO_4 and concentrated to dryness. The crude material was purified by silica gel column chromatography.

Variation A: Aryl triflate (1.0 eq.), phenol (1.2 eq.) and PMP (1.5 eq.) were added to a flame-dried 5 mL Schlenk flask, followed by anhydrous PhMe (0.5 M).* The flask was sealed with a rubber septum and attached to a Schlenk manifold. The resulting stock solution was degassed *via* freeze-pump-thaw (total of three cycles).** A microwave vial containing μOMs-Pd-G4 dimer and AdBippyPhos was sealed, attached to a Schlenk manifold and evacuated, then backfilled with N_2 (total of four cycles). The degassed stock solution was then added to the vial under N_2.

> ** Solubilities of each solid aryl triflate and phenol in PhMe were tested prior to the stock solution preparation. In case of poor solubility, the reagent(s) were added to the microwave vial together with the pre-catalyst and ligand.*
> *** Liquid N_2 bath was used to freeze the contents of the Schlenk flask – it could be substituted with a dry ice / acetone bath if more convenient.*

Variation B: All the reagents were added to the flame-dried microwave vial in the following order: μOMs-Pd-G4 dimer (either 1 mol% or 2 mol%), AdBippyPhos (4 mol% or 8 mol%; 4 equivalents relative to the amount of μOMs-Pd-G4 dimer used), aryl triflate (1.0 eq.), phenol (1.2 eq.), anhydrous PhMe (0.5 M) and PMP (1.5 eq.). The vial was then sealed, attached to a Schlenk manifold and the reaction mixture was degassed by slowly opening to the vacuum until a noticeable first gas bubble, then immediately backfilling with N_2. The evacuation/backfilling process was then repeated four more times.

N.B. Variation A and Variation B were tested for reproducibility and can be used interchangeably.

2-(4-Fluorophenoxy)-1,4-dimethylbenzene (3)

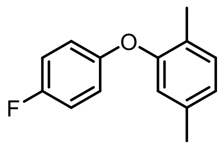

Prepared according to the *General Procedure for C-O Cross-Coupling, Variation A*. Using aryl triflate **1b** (83.4 μL, 0.500 mmol), 2,5-dimethylphenol (73.2 mg, 0.600 mmol), *μ*OMs-Pd-G4 dimer•3/10Et_2O (3.9 mg, 0.005 mmol, 2 mol% Pd) and AdBippyPhos (13.3 mg, 0.02 mmol, 4 mol%) afforded, after purification by silica gel column chromatography (100% pentane), the title compound (92 mg, 0.425 mmol, 85%) as a colourless oil.

^{1}H NMR (400 MHz, $CDCl_3$): δ 7.13 (d, *J* = 7.7 Hz, 1H), 7.04 – 6.95 (m, 2H), 6.91 – 6.83 (m, 3H), 6.68 (s, 1H), 2.28 (s, 3H), 2.20 (s, 3H).

^{13}C{^{1}H} NMR (101 MHz, $CDCl_3$): δ 158.0 (d, *J* = 241.9 Hz), 154.8, 153.9 (d, *J* = 2.7 Hz), 137.3, 131.3, 126.5, 124.8, 119.9, 118.8 (d, *J* = 8.0 Hz), 116.2 (d, *J* = 23.5 Hz), 21.1, 15.9.

^{19}F NMR (376 MHz, $CDCl_3$): δ -121.73 (tt, *J* = 8.2, 4.6 Hz).

MS (EI): calcd. for $C_{14}H_{13}OF^+$ ($[M]^+$): 216.09449. Found: 216.09385.

ν (ATR)/cm^{-1} (neat): 2952, 1579, 1428, 1195, 1018, 832, 702, 579.

2-(4-Methoxyphenoxy)-1,4-dimethylbenzene (4)

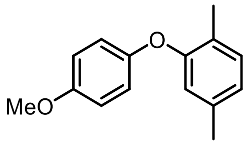

Prepared according to the *General Procedure for C-O Cross-Coupling, Variation A*. Using aryl triflate (128.1 mg, 0.500 mmol), 2,5-dimethylphenol (73.3 mg, 0.600 mmol), *μ*OMs-Pd-G4 dimer•3/10Et_2O (3.94 mg, 0.005 mmol, 2 mol% Pd) and AdBippyPhos (13.26 mg, 0.02 mmol, 4 mol%) afforded, after purification by silica gel column chromatography (100% pentane), the title compound (89 mg, 0.390 mmol, 78%) as a yellow oil.

^{1}H NMR (500 MHz, $CDCl_3$): δ 7.11 (d, *J* = 7.5 Hz, 1H), 6.92 – 6.85 (m, 4H), 6.84 (d, *J* = 7.5 Hz, 1H), 6.63 (s, 1H), 3.81 (s, 3H), 2.26 (s, 3H), 2.24 (s, 3H).

^{13}C{^{1}H} NMR (126 MHz, $CDCl_3$): δ 155.7, 155.3, 151.4, 137.0, 131.1, 126.0, 124.0, 119.4, 118.9, 114.9, 55.8, 21.2, 15.9.

MS (EI): calcd. for $C_{15}H_{16}O_2^+$ ([M]$^+$): 228.11448. Found: 228.11408.

ν (ATR)/cm^{-1} (neat): 2997, 2948, 1619, 1591, 1500, 1240, 1208, 1116, 808, 766, 698.

2-(2,5-dimethylphenoxy)naphthalene (5)

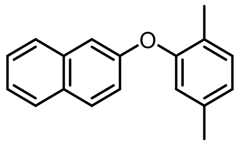

Prepared according to the *General Procedure for C-O Cross-Coupling, Variation A*. Using aryl triflate (138.1 mg, 0.500 mmol), 2,5-dimethylphenol (73.2 mg, 0.600 mmol), *μ*OMs-Pd-G4 dimer•1/5Et_2O (3.9 mg, 0.005 mmol, 2 mol% Pd) and AdBippyPhos (13.26 mg, 0.02 mmol, 4 mol%) afforded, after purification by silica gel column chromatography (100% pentane), the title compound (92 mg, 0.370 mmol, 74%) as a yellow oil.

^{1}H NMR (400 MHz, $CDCl_3$): δ 7.83 (d, *J* = 3.5 Hz, 1H), 7.81 (d, *J* = 2.6 Hz, 1H), 7.67 (d, *J* = 8.0 Hz, 1H), 7.47 – 7.35 (m, 2H), 7.28 – 7.24 (m, 1H), 7.19 (d, *J* = 7.7 Hz, 1H), 7.10 (d, *J* = 2.6 Hz, 1H), 6.95 (d, *J* = 7.7 Hz, 1H), 6.82 (s, 1H), 2.31 (s, 3H), 2.23 (s, 3H).

^{13}C NMR (101 MHz, $CDCl_3$): δ 156.1, 154.2, 137.4, 134.5, 131.4, 129.9, 129.8, 127.8, 127.1, 127.1, 126.6, 125.2, 124.4, 121.0, 119.3, 111.7, 21.1, 15.9.

MS (EI): calcd. for $C_{15}H_{13}OF_3^+$ ([M]$^+$): 248.11957. Found: 248.11956.

ν (ATR)/cm^{-1} (neat): 3053, 2920, 1630, 1505, 1462, 1212, 1122, 809, 775, 693.

2-(3,5-Dimethoxyphenoxy)-1,4-dimethylbenzene (6)

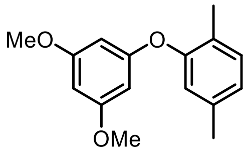

Prepared according to the *General Procedure for C-O Cross-Coupling, Variation A*. Using aryl triflate (143.1 mg, 0.500 mmol), 2,5-dimethylphenol (73.2 mg, 0.600 mmol), *μ*OMs-Pd-G4 dimer•1/5Et_2O (3.9 mg, 0.005 mmol, 2 mol% Pd) and AdBippyPhos (13.3 mg, 0.02 mmol, 4 mol%) afforded, after purification by silica gel column chromatography (0-3% EtOAc in pentane), the title compound (106 mg, 0.410 mmol, 82%) as a yellow oil.

^{1}H NMR (400 MHz, $CDCl_3$): δ 7.11 (d, *J* = 7.6 Hz, 1H), 6.89 (d, *J* = 7.6 Hz, 1H), 6.77 (s, 1H), 6.16 (t, *J* = 2.3 Hz, 1H), 6.06 (d, *J* = 2.3 Hz, 2H), 3.75 (s, 6H), 2.28 (s, 3H), 2.17 (s, 3H).

^{13}C{^{1}H} NMR (101 MHz, $CDCl_3$): δ 161.7, 160.2, 153.8, 137.3, 131.3, 127.1, 125.3, 121.1, 95.9, 94.4, 55.5, 21.1, 15.8.

HMRS (ESI$^+$, *m/z*): calcd. for $C_{16}H_{18}O_3+H^+$: 259.1329 $[M+H]^+$. Found: 259.1344.

ν (ATR)/cm^{-1} (neat): 2954, 2848, 1597, 1471, 1253, 1205, 1152, 1137, 1111, 1055, 806.

Methyl 4-(2,5-dimethylphenoxy)benzoate (7)

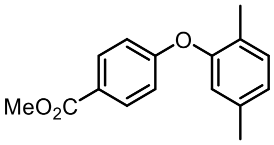

Prepared according to the *General Procedure for C-O Cross-Coupling, Variation A*. Using aryl triflate (142.1 mg, 0.500 mmol), 2,5-dimethylphenol (73.3 mg, 0.600 mmol), *µ*OMs-Pd-G4 dimer•1/5Et_2O (3.9 mg, 0.005 mmol, 2 mol% Pd) and AdBippyPhos (13.26 mg, 0.02 mmol, 4 mol%) afforded, after purification by silica gel column chromatography (99:1 pentane/EtOAc), the title compound (79 mg, 0.308 mmol, 62%) as a yellow oil.

^{1}H NMR (400 MHz, $CDCl_3$): δ 7.98 (d, *J* = 9.0 Hz, 2H), 7.15 (d, *J* = 7.7 Hz, 1H), 6.96 (d, *J* = 7.7 Hz, 1H), 6.87 (d, *J* = 9.0 Hz, 2H), 6.81 (s, 1H), 3.89 (s, 3H), 2.31 (s, 3H), 2.13 (s, 3H).

^{13}C{^{1}H} NMR (101 MHz, $CDCl_3$): δ 166.8, 162.3, 153.0, 137.6, 131.8, 131.5, 127.3, 126.1, 123.9, 121.7, 116.1, 52.1, 21.1, 15.8.

HMRS (ESI$^+$, *m/z*): calcd. for $C_{16}H_{16}O_3$+H^+: 257.1172 $[M+H]^+$. Found: 257.1167.

ν (ATR)/cm^{-1} (neat): 2950, 2921, 1717, 1603, 1503, 1250, 1227, 1192, 1108, 849, 769.

1,4-Dimethyl-2-(4-(trifluoromethyl)phenoxy)benzene (8)

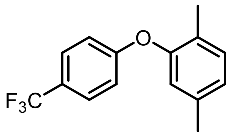

Prepared according to the *General Procedure for C-O Cross-Coupling, Variation A*. Using aryl triflate (147.1 mg, 0.500 mmol), 2,5-dimethylphenol (73.3 mg, 0.600 mmol), *μ*OMs-Pd-G4 dimer•3/10Et_2O (3.94 mg, 0.005 mmol, 2 mol% Pd) and AdBippyPhos (13.26 mg, 0.02 mmol, 4 mol%) afforded, after purification by silica gel column chromatography (100% pentane), the title compound (116 mg, 0.436 mmol, 87%) as a colourless oil.

^{1}H NMR (500 MHz, $CDCl_3$): δ 7.54 (d, *J* = 8.6 Hz, 2H), 7.17 (d, *J* = 7.7 Hz, 1H), 6.97 (d, *J* = 7.7 Hz, 1H), 6.93 (d, *J* = 8.6 Hz, 2H), 6.80 (s, 1H), 2.32 (s, 3H), 2.15 (s, 3H).

^{13}C{^{1}H} NMR (101 MHz, $CDCl_3$): δ 161.1, 153.0, 137.7, 131.6, 127.3, 127.2 (q, *J* = 3.9 Hz), 126.1, 124.5 (q, *J* = 271.0 Hz), 124.2 (q, *J* = 33.2 Hz), 121.6, 116.5, 21.1, 15.8.

^{19}F NMR (376 MHz, $CDCl_3$): δ -61.61 (s, 3F).

MS (EI): calcd. for $C_{15}H_{13}OF_3^+$ ($[M]^+$): 266.09130. Found: 266.09077.

ν (ATR)/cm^{-1} (neat): 2925, 1614, 1323, 1253, 1115, 1065, 838, 811, 632, 613, 505.

4-(2,5-Dimethylphenoxy)benzonitrile (9)

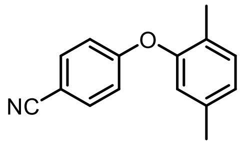

Prepared according to the *General Procedure for C-O Cross-Coupling, Variation A*. Using aryl triflate (125.6 mg, 0.500 mmol), 2,5-dimethylphenol (73.2 mg, 0.600 mmol), *μ*OMs-Pd-G4 dimer•3/10Et_2O (3.94 mg, 0.005 mmol, 2 mol% Pd) and AdBippyPhos (13.3 mg, 0.02 mmol, 4 mol%) afforded, after purification by silica gel column chromatography (0-2% EtOAc in pentane), the title compound (105 mg, 0.470 mmol, 94%) as a yellow oil.

^{1}H NMR (400 MHz, $CDCl_3$): δ 7.57 (d, *J* = 8.9 Hz, 2H), 7.16 (d, *J* = 7.7 Hz, 1H), 6.98 (d, *J* = 7.7 Hz, 1H), 6.90 (d, *J* = 8.9 Hz, 2H), 6.80 (app. s, 1H), 2.32 (s, 3H), 2.11 (s, 3H).

^{13}C{^{1}H} NMR (101 MHz, $CDCl_3$): δ 162.0, 152.3, 137.8, 134.2, 131.7, 127.3, 126.6, 121.7, 119.1, 116.8, 105.2, 21.0, 15.7.

HMRS (ESI$^+$, *m/z*): calcd. for $C_{15}H_{13}NO+H^+$: 224.1070 $[M+H]^+$. Found: 224.1069.

ν (ATR)/cm^{-1} (neat): 2923, 2225, 1603, 1499, 1250, 1165, 1112, 813, 767.

2-(3,5-Difluorophenoxy)-1,4-dimethylbenzene (10)

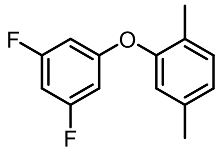

Prepared according to the *General Procedure for C-O Cross-Coupling, Variation A*. Using aryl triflate (131.1 mg, 0.500 mmol), 2,5-dimethylphenol (73.2 mg, 0.600 mmol), μOMs-Pd-G4 dimer•1/5Et_2O (7.8 mg, 0.01 mmol, 4 mol% Pd) and AdBippyPhos (26.5 mg, 0.04 mmol, 8 mol%) afforded, after purification by silica gel column chromatography (100% pentane), the title compound (105 mg, 0.448 mmol, 90%) as a yellow oil.

^{1}H NMR (400 MHz, $CDCl_3$): δ 7.15 (d, *J* = 7.7 Hz, 1H), 6.97 (d, *J* = 7.7 Hz, 1H), 6.80 (s, 1H), 6.47 (tt, *J* = 8.9, 2.3 Hz, 1H), 6.38 (app. dd, *J* = 8.7, 2.3 Hz, 2H), 2.32 (s, 3H), 2.14 (s, 3H).

^{13}C{^{1}H} NMR (101 MHz, $CDCl_3$): δ 165.3 – 162.4 (m), 160.8 – 160.4 (m), 152.7, 137.7, 131.6, 127.3, 126.4, 121.7, 100.5 – 99.9 (m), 98.0 – 97.2 (m), 21.1, 15.7.

^{19}F NMR (377 MHz, $CDCl_3$): δ -108.96 (app. t, *J* = 9.1 Hz, 2F).

MS (EI): calcd. for $C_{14}H_{12}OF_2^+$ ([M]$^+$): 234.08507. Found: 234.08548.

ν (ATR)/cm^{-1} (neat): 2953, 1616, 1600, 1465, 1250, 1133, 1115, 993, 838, 811, 670.

1,4-Dimethyl-2-(4-nitrophenoxy)benzene (11)

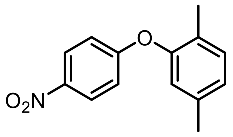

Prepared according to the *General Procedure for C-O Cross-Coupling, Variation A*. Using aryl triflate (135.6 mg, 0.500 mmol), 2,5-dimethylphenol (73.2 mg, 0.600 mmol), *μ*OMs-Pd-G4 dimer•3/10Et_2O (3.94 mg, 0.005 mmol, 2 mol% Pd) and AdBippyPhos (13.3 mg, 0.02 mmol, 4 mol%) afforded, after purification by silica gel column chromatography (99:1 pentane/EtOAc), the title compound (112 mg, 0.460 mmol, 92%) as a dark orange oil.

^{1}H NMR (500 MHz, $CDCl_3$): δ 8.18 (d, *J* = 9.3 Hz, 2H), 7.18 (d, *J* = 7.8 Hz, 1H), 7.01 (d, *J* = 7.8 Hz, 1H), 6.91 (d, *J* = 9.3 Hz, 2H), 6.83 (s, 1H), 2.33 (s, 3H), 2.12 (s, 3H).

^{13}C{^{1}H} NMR (126 MHz, $CDCl_3$): δ 163.7, 152.3, 142.4, 138.0, 131.8, 127.3, 126.8, 126.1, 121.8, 116.1, 21.0, 15.7.

HMRS (ESI$^+$, *m/z*): calcd. for $C_{14}H_{13}NO_3$+H^+: 244.0968 [M+H]$^+$. Found: 244.0966.

ν (ATR)/cm^{-1} (neat): 2922, 2860, 1609, 1457, 1407, 1341, 1250, 1163, 1110, 1041, 810, 750, 615.

2-(2-(Benzyloxy)phenoxy)-1,4-dimethylbenzene (12)

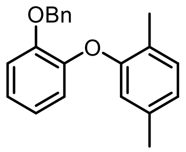

Prepared according to the *General Procedure for C-O Cross-Coupling, Variation A*. Using aryl triflate (166.1 mg, 0.500 mmol), 2,5-dimethylphenol (73.2 mg, 0.600 mmol), *μ*OMs-Pd-G4 dimer•1/5Et_2O (7.8 mg, 0.01 mmol, 4 mol% Pd) and AdBippyPhos (26.5 mg, 0.04 mmol, 8 mol%) afforded, after purification by silica gel column chromatography (0-3% EtOAc in pentane), the title compound (131 mg, 0.430 mmol, 86%) as a colourless oil.

^{1}H NMR (400 MHz, $CDCl_3$): δ 7.35 – 7.26 (m, 5H), 7.12 (d, *J* = 7.6 Hz, 1H), 7.07 – 7.01 (m, 2H), 6.96 – 6.87 (m, 2H), 6.84 (d, *J* = 7.6 Hz, 1H), 6.60 (s, 1H), 5.15 (s, 2H), 2.28 (s, 3H), 2.26 (s, 3H).

$^{13}C\{^1H\}$ NMR (101 MHz, $CDCl_3$): δ 155.6, 149.9, 146.9, 137.3, 136.9, 131.0, 128.5, 127.8, 127.2, 125.6, 123.9, 123.8, 121.8, 120.1, 118.1, 115.6, 71.1, 21.2, 15.9.

HMRS (ESI$^+$, *m/z*): calcd. for $C_{21}H_{20}O_2$+Na^+: 327.1356 [M+Na]$^+$. Found: 327.1357.

ν (ATR)/cm^{-1} (neat): 3031, 2921, 1578, 1495, 1451, 1258, 1200, 1118, 996, 860, 744.

Ethyl (*S*)-6-(2,5-dimethylphenoxy)-2,5,7,8-tetramethylchromane-2-carboxylate (13)

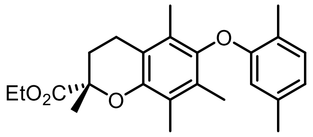

Prepared according to the *General Procedure for C-O Cross-Coupling, Variation A*. Using aryl triflate (205.2 mg, 0.500 mmol), 2,5-dimethylphenol (73.2 mg, 0.600 mmol), *μ*OMs-Pd-G4 dimer•1/5Et_2O (7.8 mg, 0.01 mmol, 4 mol% Pd) and AdBippyPhos (26.5 mg, 0.04 mmol, 8 mol%) afforded, after purification by silica gel column chromatography (0-4% EtOAc in pentane), the title compound (110 mg, 0.286 mmol, 57%) as a colourless solid.

^{1}H NMR (400 MHz, $CDCl_3$): δ 7.08 (d, *J* = 7.5 Hz, 1H), 6.66 (d, *J* = 7.5 Hz, 1H), 6.07 – 5.99 (m, 1H), 4.33 – 4.06 (m, 2H), 2.72 – 2.62 (m, 1H), 2.61 – 2.43 (m, 2H), 2.37 (s, 3H), 2.22 (s, 3H), 2.15 (s, 3H), 2.01 (s, 3H), 1.98 – 1.88 (m, 4H), 1.65 (s, 3H), 1.19 (app. t, *J* = 7.1 Hz, 3H).

^{13}C{^{1}H} NMR (101 MHz, $CDCl_3$): δ 173.9, 156.5, 148.8, 144.5, 136.6, 130.7, 128.6, 126.3, 123.4, 122.7, 121.3, 117.7, 112.8, 61.2, 30.6, 25.7, 25.1, 21.4, 21.0, 16.1, 14.2, 12.9, 12.02, 11.99.

HMRS (ESI$^+$, *m/z*): calcd. for $C_{24}H_{30}O_4$+Na^+: 405.2036 $[M+Na]^+$. Found: 405.2035.

m. p./ °C: 106-107.

ν (ATR)/cm^{-1} (neat): 2984, 2922, 1730, 1730, 1506, 1452, 1403, 1247, 1197, 1122, 1105, 860, 813, 561.

***tert*-Butyl 5-(2,5-dimethylphenoxy)-3-(2-(1,3-dioxoisoindolin-2-yl)ethyl)-1*H*-indole-1-carboxylate (14)**

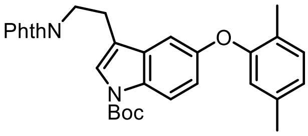

Prepared according to the *General Procedure for C-O Cross-Coupling, Variation A*. Using aryl triflate (269.2 mg, 0.500 mmol), 2,5-dimethylphenol (73.2 mg, 0.600 mmol), μOMs-Pd-G4 dimer•1/5Et$_2$O (7.8 mg, 0.01 mmol, 4 mol% Pd) and AdBippyPhos (26.5 mg, 0.04 mmol, 8 mol%) afforded, after purification by silica gel column chromatography (0-15% EtOAc in pentane), the title compound (243 mg, 0.476 mmol, 95%) as a colourless crystalline solid.

^{1}H NMR (500 MHz, CDCl$_3$): δ 8.04 (app.s, 1H), 7.86 – 7.81 (m, 2H), 7.73 – 7.68 (m, 2H), 7.48 (s, 1H), 7.19 (d, *J* = 2.3 Hz, 1H), 7.12 (d, *J* = 7.7 Hz, 1H), 6.92 (dd, *J* = 8.9, 2.3 Hz, 1H), 6.83 (app. dd, *J* = 7.7, 1.2 Hz, 1H), 6.62 (s, 1H), 4.02 – 3.96 (m, 2H), 3.05 – 2.99 (m, 2H), 2.25 (s, 3H), 2.24 (s, 3H), 1.65 (s, 9H).

^{13}C{^{1}H} NMR (101 MHz, DMSO-d$_6$): δ 167.8, 155.1, 152.6, 148.8, 136.7, 134.4, 131.5, 131.3, 131.1, 130.8, 125.1, 124.5, 124.1, 123.0, 118.6, 117.1, 115.9, 115.4, 107.8, 83.6, 37.3, 27.6, 23.2, 20.6, 15.5.

HMRS (ESI$^+$, *m/z*): calcd. for $C_{31}H_{30}N_2O_5+Na^+$: 533.2047 $[M+Na]^+$. Found: 533.2049.

m. p./ °C: 72-74.

ν (ATR)/cm^{-1} (neat): 2977, 1710, 1376, 1251, 1156, 1070, 868, 714.

2-(2,5-Dimethylphenoxy)-5-(trifluoromethyl)pyridine (15)

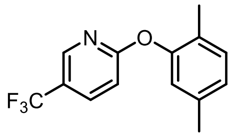

Prepared according to the *General Procedure for C-O Cross-Coupling, Variation A*. Using aryl triflate (147.6 mg, 0.500 mmol), 2,5-dimethylphenol (73.2 mg, 0.600 mmol), μOMs-Pd-G4 dimer•1/5Et_2O (7.8 mg, 0.01 mmol, 4 mol% Pd) and AdBippyPhos (26.5 mg, 0.04 mmol, 8 mol%) afforded, after purification by silica gel column chromatography (0-5% EtOAc in pentane), the title compound (115 mg, 0.430 mmol, 86%) as a colourless oil.

^{1}H NMR (400 MHz, $CDCl_3$): δ 8.47 – 8.43 (m, 1H), 7.88 (dd, *J* = 8.7, 2.4 Hz, 1H), 7.18 (d, *J* = 7.7 Hz, 1H), 7.01 (d, *J* = 7.7 Hz, 1H), 6.97 (d, *J* = 8.7 Hz, 1H), 6.89 (s, 1H), 2.35 (s, 3H), 2.12 (s, 3H).

^{13}C{^{1}H} NMR (101 MHz, $CDCl_3$): δ 166.0, 151.4, 145.8 (q, *J* = 4.2 Hz), 137.4, 136.7 (q, *J* = 3.5 Hz), 131.4, 127.5, 126.9, 123.9 (q, *J* = 270.7 Hz), 122.5, 121.2 (q, *J* = 33.5 Hz), 110.7, 21.1, 16.0.

^{19}F NMR (376 MHz, $CDCl_3$): δ -61.60 (s, 3F).

HMRS (ESI$^+$, *m/z*): calcd. for $C_{14}H_{12}F_3NO+Na^+$: 290.0763 $[M+Na]^+$. Found: 290.0768.

ν (ATR)/cm^{-1} (neat): 2918, 1608, 1578, 1325, 1284, 1160, 1123, 1011, 810, 759, 711.

2-(2,5-Dimethylphenoxy)-4-fluoropyridine (16)

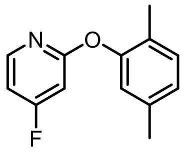

Prepared according to the *General Procedure for C-O Cross-Coupling, Variation B*. Using aryl triflate (122.6 mg, 0.500 mmol), 2,5-dimethylphenol (73.2 mg, 0.600 mmol), μOMs-Pd-G4 dimer•MeCN (17.0 mg, 0.02 mmol, 8 mol% Pd) and AdBippyPhos (53.0 mg, 0.08 mmol, 16 mol%) afforded, after purification by silica gel column chromatography (93:7 pentane/EtOAc), the title compound (73 mg, 0.336 mmol, 67%) as a yellow oil.

^{1}H NMR (400 MHz, $CDCl_3$): δ 8.14 (dd, *J* = 5.8, 3.1 Hz, 1H), 7.16 (d, *J* = 7.6 Hz, 1H), 6.98 (d, *J* = 7.7 Hz, 1H), 6.88 (s, 1H), 6.76 – 6.69 (m, 1H), 6.54 (dd, *J* = 10.1, 2.1 Hz, 1H), 2.33 (s, 3H), 2.12 (s, 3H).

^{13}C{^{1}H} NMR (101 MHz, $CDCl_3$): δ 170.8 (d, *J* = 259.9 Hz), 166.0 (d, *J* = 11.5 Hz), 151.7, 149.9 (d, *J* = 8.9 Hz), 137.3, 131.3, 127.5, 126.6, 122.5, 106.9 (d, *J* = 18.4 Hz), 97.9 (d, *J* = 21.2 Hz), 21.1, 16.0.

^{19}F NMR (376 MHz, $CDCl_3$): δ -100.3 (app. q, *J* = 9.0 Hz).

HMRS (ESI^{+}, *m/z*): calcd. for $C_{13}H_{12}FNO+H^{+}$: 218.0976 $[M+H]^{+}$. Found: 218.0975.

ν (ATR)/cm^{-1} (neat): 2923, 1583, 1472, 1399, 1247, 1112, 1005, 966, 845, 809.

4-(2,5-Dimethylphenoxy)-2-fluoropyridine (17)

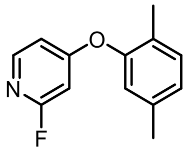

Prepared according to the *General Procedure for C-O Cross-Coupling, Variation B*. Using aryl triflate (122.6 mg, 0.500 mmol), 2,5-dimethylphenol (73.2 mg, 0.600 mmol), *μ*OMs-Pd-G4 dimer•MeCN (17.0 mg, 0.02 mmol, 8 mol% Pd) and AdBippyPhos (53.0 mg, 0.08 mmol, 16 mol%) afforded, after purification by silica gel column chromatography (93:7 pentane/EtOAc), the title compound (98 mg, 0.451 mmol, 90%) as an orange oil.

^{1}H NMR (400 MHz, $CDCl_3$): δ 8.04 (d, *J* = 5.8 Hz, 1H), 7.17 (d, *J* = 7.7 Hz, 1H), 7.01 (d, *J* = 7.9 Hz, 1H), 6.84 (s, 1H), 6.71 – 6.66 (m, 1H), 6.26 (d, *J* = 2.0 Hz, 1H), 2.33 (s, 3H), 2.10 (s, 3H).

^{13}C{^{1}H} NMR (101 MHz, $CDCl_3$): δ 168.7 (d, *J* = 12.2 Hz), 165.4 (d, *J* = 235.3 Hz), 151.5, 148.6 (d, *J* = 18.8 Hz), 138.0, 131.8, 127.3, 127.2, 121.9, 110.1 (d, *J* = 4.0 Hz), 96.2 (d, *J* = 42.5 Hz), 21.0, 15.6.

^{19}F NMR (376 MHz, $CDCl_3$): δ -66.3 (app. s).

HMRS (ESI^{+}, *m/z*): calcd. for $C_{13}H_{12}FNO+H^{+}$: 218.0976 $[M+H]^{+}$. Found: 218.0982.

ν (ATR)/cm^{-1} (neat): 2924, 1604, 1578, 1473, 1413, 1247, 1155, 976, 929, 814, 788.

1-Fluoro-4-(4-methoxyphenoxy)benzene (18)

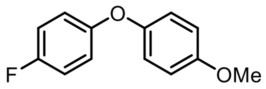

Prepared according to the *General Procedure for C-O Cross-Coupling, Variation A*. Using aryl triflate **1b** (83.4 µL, 0.500 mmol), 4-methoxyphenol (74.5 mg, 0.600 mmol), *µ*OMs-Pd-G4 dimer•1/5Et_2O (3.9 mg, 0.005 mmol, 2 mol% Pd) and AdBippyPhos (13.3 mg, 0.020 mmol, 4 mol%) afforded, after purification by silica gel column chromatography (100% pentane), the title compound (87 mg, 0.400 mmol, 80%) as a yellow oil.

Characterisation data were consistent with literature values: ^{1}H, ^{13}C and ^{19}F NMR.[39]

^{1}H NMR (500 MHz, $CDCl_3$): δ 7.02 – 6.86 (m, 8H), 3.80 (s, 3H).

^{13}C{^{1}H} NMR (126 MHz, $CDCl_3$): δ 158.5 (d, *J* = 240.7 Hz), 156.0, 154.4 (d, *J* = 2.5 Hz), 150.8, 120.4, 119.3 (d, *J* = 8.4 Hz), 116.2 (d, *J* = 23.2 Hz), 115.0, 55.8.

^{19}F NMR (376 MHz, $CDCl_3$): δ -121.35 (tt, *J* = 8.0, 4.2 Hz).

ν (ATR)/cm^{-1} (neat): 2952, 2908, 2835, 1492, 1465, 1441, 1296, 1204, 1035, 824, 772.

1-Fluoro-4-phenoxybenzene (19)

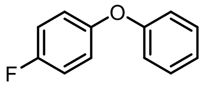

Prepared according to the *General Procedure for C-O Cross-Coupling, Variation A*. Using aryl triflate **1b** (83.4 μL, 0.500 mmol), phenol (56.5 mg, 0.600 mmol), *μ*OMs-Pd-G4 dimer•1/5Et_2O (7.8 mg, 0.01 mmol, 4 mol% Pd) and AdBippyPhos (26.5 mg, 0.04 mmol, 8 mol%) afforded, after purification by silica gel column chromatography (100% pentane), the title compound (86 mg, 0.455 mmol, 91%) as a yellow oil.

Characterisation data were consistent with literature values: ^{1}H, ^{13}C, ^{19}F NMR[40] and IR.[41]

^{1}H NMR (400 MHz, $CDCl_3$): δ 7.37 – 7.31 (m, 2H), 7.13 – 7.08 (m, 1H), 7.07 – 6.96 (m, 6H).

^{13}C{^{1}H} NMR (101 MHz, $CDCl_3$): δ 159.0 (d, *J* = 234.1 Hz), 157.8, 153.0 (d, *J* = 2.2 Hz), 129.9, 123.3, 120.7 (d, *J* = 7.5 Hz), 118.4, 116.5 (d, *J* = 23.5 Hz).

^{19}F NMR (376 MHz, $CDCl_3$): δ -120.12 (tt, *J* = 8.2, 4.8 Hz).

ν (ATR)/cm^{-1} (neat): 3065, 2924, 1589, 1501, 1486, 1248, 1209, 837, 811, 769, 751, 691.

4,4'-Oxybis(fluorobenzene) (20)

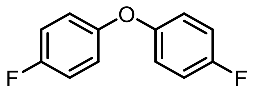

Prepared according to the *General Procedure for C-O Cross-Coupling, Variation A*. Using aryl triflate **1b** (83.4 μL, 0.500 mmol), 4-fluorophenol (67.3 mg, 0.600 mmol), *μ*OMs-Pd-G4 dimer•1/5Et_2O (7.8 mg, 0.01 mmol, 4 mol% Pd) and AdBippyPhos (26.5 mg, 0.04 mmol, 8 mol%) afforded, after purification by silica gel column chromatography (100% pentane), the title compound (86 mg, 0.368 mmol, 74%) as a colourless oil.

Characterisation data were consistent with literature values: ^{1}H, ^{13}C and ^{19}F NMR.[42]

^{1}H NMR (400 MHz, $CDCl_3$): δ 7.06 – 6.99 (m, 4H), 6.98 – 6.91 (m, 4H).

^{13}C{^{1}H} NMR (101 MHz, $CDCl_3$): δ 158.9 (d, *J* = 241.8 Hz), 153.5 (d, *J* = 2.1 Hz), 120.1 (d, *J* = 8.6 Hz), 116.5 (t, *J* = 23.3 Hz).

^{19}F NMR (377 MHz, $CDCl_3$): δ -120.23 (tt, *J* = 8.3, 4.4 Hz, 2F).

ν (ATR)/cm^{-1} (neat): 1490, 1198, 826, 782.

Methyl (*S*)-2-((*tert*-Butoxycarbonyl)amino)-3-(4-(4-fluorophenoxy)phenyl)propanoate (21)

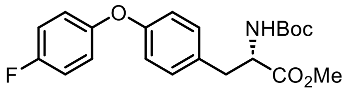

Prepared according to the *General Procedure for C-O Cross-Coupling, Variation A*. Using aryl triflate **1b** (83.4 μL, 0.500 mmol), Boc-L-tyrosine methyl ester (177.2 mg, 0.600 mmol),* *μ*OMs-Pd-G4 dimer•1/5Et_2O (7.8 mg, 0.01 mmol, 4 mol% Pd) and AdBippyPhos (26.5 mg, 0.04 mmol, 8 mol%) afforded, after purification by silica gel column chromatography (0 - 6% EtOAc in pentane), the title compound (136 mg, 0.350 mmol, 70%) as an orange gel.

* *Added to the reaction vessel as a solid.*

^{1}H NMR (400 MHz, $CDCl_3$): δ 7.07 (d, *J* = 8.5 Hz, 2H), 7.05 – 6.99 (m, 2H), 6.99 – 6.93 (m, 2H), 6.88 (d, *J* = 8.7 Hz, 2H), 5.00 (d, major rotamer, *J* = 7.7 Hz, 0.89H), 4.70 (br. s, minor rotamer, 0.11H), 4.62 – 4.51 (m, major rotamer, 0.88H), 4.38 (br. s, minor rotamer, 0.12H), 3.72 (s, 3H), 3.14 – 2.95 (m, 2H), 1.42 (s, 9H).

^{13}C{^{1}H} NMR (101 MHz, $CDCl_3$): δ 172.4, 159.0 (d, *J* = 241.9 Hz), 156.9, 155.2, 152.9, 130.9, 130.8, 120.7 (d, *J* = 8.3 Hz), 118.4, 116.4 (d, *J* = 23.3 Hz), 80.1, 54.6, 52.4, 37.8, 28.4.

^{19}F NMR (376 MHz, $CDCl_3$): δ -119.88 (br. s, minor rotamer, 0.17F), -120.02 (tt, *J* = 8.0, 4.8 Hz).

HMRS (ESI$^+$, *m/z*): calcd. for $C_{21}H_{24}FNO_5$+H^+: 390.1711 $[M+H]^+$. Found: 390.1706.

ν (ATR)/cm^{-1} (neat): 3369, 2978, 2931, 1743, 1711, 1494, 1365, 1248, 1210, 1161, 1090, 1055, 833.

(8*R*,9*S*,13*S*,14*S*)-3-(4-fluorophenoxy)-13-methyl-6,7,8,9,11,12,13,14,15,16-decahydro-17H-cyclopenta[*a*]phenanthren-17-one (22)

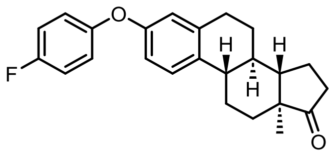

Prepared according to the *General Procedure for C-O Cross-Coupling, Variation A*. Using aryl triflate **1b** (83.4 µL, 0.500 mmol), estrone (162.2 mg, 0.600 mmol),* *µ*OMs-Pd-G4 dimer•1/5Et_2O (7.8 mg, 0.01 mmol, 4 mol% Pd) and AdBippyPhos (26.5 mg, 0.04 mmol, 8 mol%) afforded, after purification by silica gel column chromatography (0 - 10% EtOAc in pentane), the title compound (148 mg, 0.410 mmol, 81%) as an off-white solid.

** Added to the reaction vessel as a solid.*

^{1}H NMR (400 MHz, $CDCl_3$): δ 7.23 (d, *J* = 8.5 Hz, 1H), 7.05 – 6.94 (m, 4H), 6.76 (dd, *J* = 8.5, 2.6 Hz, 1H), 6.70 (d, *J* = 2.6 Hz, 1H), 2.91 – 2.83 (m, 2H), 2.56 – 2.46 (m, 1H), 2.45 – 2.37 (m, 1H), 2.33 – 2.23 (m, 1H), 2.21 – 1.91 (m, 4H), 1.70 – 1.38 (m, 6H), 0.92 (s, 3H).

^{13}C{^{1}H} NMR (101 MHz, $CDCl_3$): δ 220.9, 158.8 (d, *J* = 241.3 Hz), 155.7, 153.2 (d, *J* = 2.6 Hz), 138.4, 134.8, 126.8, 120.5 (d, *J* = 8.5 Hz), 118.5, 116.3 (d, *J* = 23.4 Hz), 115.9, 50.6, 48.1, 44.2, 38.4, 36.0, 31.7, 29.7, 26.6, 26.0, 21.7, 14.0.

^{19}F NMR (376 MHz, $CDCl_3$): δ -120.45 (tt, *J* = 8.1, 4.6 Hz).

HMRS (ESI$^+$, *m/z*): calcd. for $C_{24}H_{25}FO_2$+Na^+: 387.1731 $[M+Na]^+$. Found: 387.1735.

m. p./ °C: 142-143

ν (ATR)/cm^{-1} (neat): 2928, 2877, 1730, 1489, 1281, 1247, 1224, 1197, 1184, 830, 790, 765, 500.

tert-Butyl 5-(4-fluorophenoxy)-1H-indole-1-carboxylate (23)

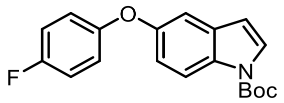

Prepared according to the *General Procedure for C-O Cross-Coupling, Variation A*. Using aryl triflate **1b** (83.4 μL, 0.500 mmol), **3** (137.0 mg, 0.600 mmol), *μ*OMs-Pd-G4 dimer•1/5Et_2O (7.8 mg, 0.01 mmol, 4 mol% Pd) and AdBippyPhos (26.51 mg, 0.04 mmol, 8 mol%) afforded, after purification by silica gel column chromatography (0-20% EtOAc in pentane), followed by trituration with pentane, the title compound (95 mg, 0.290 mmol, 58%) as a white solid.

^{1}H NMR (400 MHz, $CDCl_3$): δ 7.48 – 7.38 (m, 4H), 7.30 (d, *J* = 3.2 Hz, 1H), 7.24 – 7.18 (m, 2H), 7.03 (dd, *J* = 8.9, 2.2 Hz, 1H), 6.65 (dd, *J* = 3.2, 0.5 Hz, 1H), 1.58 (s, 9H).

^{13}C{^{1}H} NMR (101 MHz, DMSO-d$_6$): δ 160.4 (d, *J* = 244.1 Hz), 152.1, 144.8, 135.3 (d, *J* = 3.1 Hz), 133.1, 130.2, 129.1, 126.1 (d, *J* = 8.2 Hz), 116.6 (d, *J* = 22.8 Hz), 116.4, 112.8, 110.7, 103.5, 82.7, 27.3.

^{19}F NMR (376 MHz, $CDCl_3$): δ -114.92 (tt, *J* = 8.2, 4.7 Hz).

HMRS (ESI$^+$, *m/z*): calcd. for $C_{19}H_{18}FNO_3+H^+$: 328.1343 $[M+H]^+$. Found: 328.1345.

ν (ATR)/cm^{-1} (neat): 2942, 1745, 1616, 1511, 1275, 1240, 1212, 1139, 841, 744.

7-(4-Fluorophenoxy)-2,2-dimethyl-2,3-dihydrobenzofuran (24)

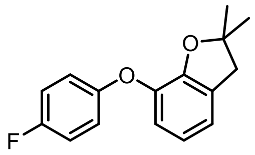

Prepared according to the *General Procedure for C-O Cross-Coupling, Variation A*. Using aryl triflate **1b** (83.4 µL, 0.500 mmol), 2,3-dihydro-2,2-dimethylbenzofuran-7-ol (89 µL, 0.600 mmol), *µ*OMs-Pd-G4 dimer•1/5Et_2O (7.8 mg, 0.01 mmol, 4 mol% Pd) and AdBippyPhos (26.51 mg, 0.04 mmol, 8 mol%) afforded, after purification by silica gel column chromatography (100% pentane), the title compound (59 mg, 0.228 mmol, 46%) as a light yellow oil.

^{1}H NMR (400 MHz, $CDCl_3$): δ 7.02 – 6.91 (m, 5H), 6.79 – 6.70 (m, 2H), 3.07 (s, 2H), 1.50 (s, 6H).

^{13}C{^{1}H} NMR (101 MHz, $CDCl_3$): δ 158.6 (d, *J* = 240.2 Hz), 153.3 (d, *J* = 2.1 Hz), 149.6, 140.9, 129.8, 120.6, 120.6, 119.3 (d, *J* = 8.2 Hz), 118.6, 115.9 (d, *J* = 23.7 Hz), 88.2, 43.3, 28.3.

^{19}F NMR (376 MHz, $CDCl_3$): δ -121.30 (tt, *J* = 7.3, 4.9 Hz).

HMRS (ESI$^+$, *m/z*): calcd. for $C_{16}H_{15}FO_2+Na^+$: 281.0948 $[M+Na]^+$. Found: 281.0952.

ν (ATR)/cm^{-1} (neat): 2973, 1593, 1499, 1479, 1463, 1194, 1135, 875, 830, 777.

2-(*tert*-Butyl)-1-(4-fluorophenoxy)-4-methylbenzene (25)

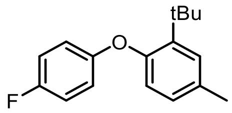

Prepared according to the *General Procedure for C-O Cross-Coupling, Variation A*. Using aryl triflate **1b** (83.4 µL, 0.500 mmol), 2-*tert*-butyl-4-methylphenol (98.5 mg, 0.600 mmol), *µ*OMs-Pd-G4 dimer•3/10Et_2O (3.94 mg, 0.005 mmol, 2 mol% Pd) and AdBippyPhos (13.26 mg, 0.02 mmol, 4 mol%) afforded, after purification by silica gel column chromatography (100% pentane), the title compound (102 mg, 0.395 mmol, 79%) as a yellow oil.

^{1}H NMR (400 MHz, $CDCl_3$): δ 7.20 (d, *J* = 2.0 Hz, 1H), 7.05 – 6.97 (m, 2H), 6.97 – 6.89 (m, 3H), 6.69 (d, *J* = 8.1 Hz, 1H), 2.34 (s, 3H), 1.41 (s, 9H).

^{13}C{^{1}H} NMR (101 MHz, $CDCl_3$): δ 158.4 (d, *J* = 239.5 Hz), 154.1 (d, *J* = 3.1 Hz), 153.9, 140.7, 132.7, 128.1, 127.7, 120.0, 119.7 (d, *J* = 8.0 Hz), 116.2 (d, *J* = 23.3 Hz), 34.8, 30.3, 21.2.

^{19}F NMR (376 MHz, $CDCl_3$): δ -121.63 (tt, *J* = 8.1, 4.5 Hz).

MS (EI): calcd. for $C_{17}H_{19}OF^+$ ($[M]^+$): 258.14144. Found: 258.14039.

ν (ATR)/cm^{-1} (neat): 2996, 1502, 1489, 1206, 1024, 840, 816, 793.

2-(4-Fluorophenoxy)-1,3,5-trimethylbenzene (26)

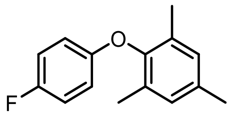

Prepared according to the *General Procedure for C-O Cross-Coupling, Variation A*. Using aryl triflate **1b** (83.4 µL, 0.500 mmol), 2,4,6-trimethylphenol (81.7 mg, 0.600 mmol), *µ*OMs-Pd-G4 dimer•1/5Et_2O (7.8 mg, 0.01 mmol, 4 mol% Pd) and AdBippyPhos (26.51 mg, 0.04 mmol, 8 mol%) afforded, after purification by silica gel column chromatography (100% pentane), the title compound (87 mg, 0.380 mmol, 76%) as a colourless oil.

^{1}H NMR (500 MHz, $CDCl_3$): δ 6.96 – 6.92 (m, 2H), 6.92 – 6.90 (m, 2H), 6.73 – 6.68 (m, 2H), 2.31 (s, 3H), 2.09 (s, 6H).

^{13}C{^{1}H} NMR (126 MHz, $CDCl_3$): δ 157.6 (d, *J* = 238.7 Hz), 154.2 (d, *J* = 2.2 Hz), 149.1, 134.7, 131.1, 129.8, 116.1 (d, *J* = 23.9 Hz), 115.5 (d, *J* = 8.1 Hz), 20.9, 16.4.

^{19}F NMR (376 MHz, $CDCl_3$): δ -123.67 (tt, *J* = 8.1, 4.2 Hz).

MS (EI): calcd. for $C_{15}H_{15}OF^+$ ($[M]^+$): 230.11014. Found: 230.11000.

ν (ATR)/cm^{-1} (neat): 2919, 1498, 1479, 1239, 1202, 1142, 855, 830, 802, 770.

(2*R*)-6-(4-Fluorophenoxy)-2,5,7,8-tetramethyl-2-(4,8,12-trimethyltridecyl)chromane (27)

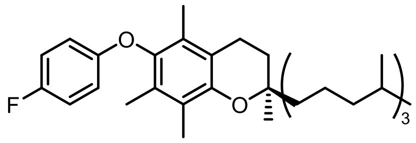

Prepared according to the *General Procedure for C-O Cross-Coupling, Variation A*. Using aryl triflate **1b** (83.4 μL, 0.500 mmol), (±)-α-tocopherol (258.4 mg, 0.600 mmol),* *μ*OMs-Pd-G4 dimer•1/5Et_2O (7.8 mg, 0.01 mmol, 4 mol% Pd) and AdBippyPhos (26.5 mg, 0.04 mmol, 8 mol%) afforded, after purification by silica gel column chromatography (0 - 10% EtOAc in pentane), the title compound (220 mg, 0.419 mmol, 84%) as a light yellow, viscous oil.

** Added to the reaction vessel separately due to its high viscosity.*

N. B. The product is a mixture of diastereoisomers due to the side-chain in the starting material.

^{1}H NMR (400 MHz, $CDCl_3$): δ 6.95 – 6.88 (m, 2H), 6.71 – 6.64 (m, 2H), 2.61 (app. t, *J* = 6.8 Hz, 2H), 2.12 (s, 3H), 2.01 (s, 3H), 1.97 (s, 3H), 1.90 – 1.74 (m, 2H), 1.69 – 1.28 (m, 11H), 1.27 (s, 3H), 1.26 – 1.00 (m, 10H), 0.90 – 0.84 (m, 12H).

^{13}C{^{1}H} NMR (101 MHz, $CDCl_3$): δ 157.4 (d, *J* = 238.7 Hz), 155.0 (d, *J* = 1.9 Hz), 148.9, 143.7, 128.3, 126.4, 123.5, 118.1, 115.9 (d, *J* = 23.2 Hz), 115.6 (d, *J* = 8.0 Hz), 75.2, 40.1, 39.5, 37.7, 37.6, 37.6, 37.6, 37.5, 37.5, 33.0, 32.9, 32.8, 31.4, 31.4, 28.1, 25.0, 24.6, 24.0, 22.9, 22.8, 21.2, 20.8, 19.9, 19.8, 19.8, 13.0, 12.1, 12.0.

^{19}F NMR (376 MHz, $CDCl_3$): δ -124.30 (tt, *J* = 8.2, 4.5 Hz).

HMRS (ESI$^+$, *m/z*): calcd. for $C_{35}H_{53}FNO_2$+Na^+: 547.3922 $[M+Na]^+$. Found: 547.3919.

ν (ATR)/cm^{-1} (neat): 2924, 1459, 1411, 1159, 829, 766.

1-(4-Fluorophenoxy)-3-methoxybenzene (28)

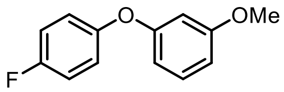

Prepared according to the *General Procedure for C-O Cross-Coupling, Variation A*. Using aryl triflate (128.1 mg, 0.500 mmol), 4-fluorophenol (67.3 mg, 0.600 mmol), *μ*OMs-Pd-G4 dimer•1/5Et_2O (3.9 mg, 0.005 mmol, 2 mol% Pd) and AdBippyPhos (13.3 mg, 0.02 mmol, 4 mol%) afforded, after purification by silica gel column chromatography (100% pentane), the title compound (83 mg, 0.380 mmol, 76%) as a yellow oil.

Characterisation data were consistent with literature values: ^{1}H and ^{13}C NMR.[43]

^{1}H NMR (400 MHz, $CDCl_3$): δ 7.25 – 7.19 (m, 1H), 7.07 – 6.97 (m, 4H), 6.65 (ddd, *J* = 8.4, 2.3, 1.1 Hz, 1H), 6.57 – 6.52 (m, 2H), 3.78 (s, 3H).

$^{13}C\{^{1}H\}$ NMR (101 MHz, $CDCl_3$): δ 161.1, 159.1, 159.1 (d, *J* = 241.7 Hz), 152.7 (d, *J* = 3.6 Hz), 130.3, 120.9 (d, *J* = 8.7 Hz), 116.4 (d, *J* = 24.0 Hz), 110.4, 108.8, 104.5, 55.5.

^{19}F NMR (376 MHz, $CDCl_3$): δ -119.9 (tt, *J* = 7.9, 4.7 Hz).

MS (EI): calcd. for $C_{13}H_{11}O_2F^+$ ($[M]^+$): 218.07376. Found: 218.07387.

ν (ATR)/cm^{-1} (neat): 2942, 1588, 1486, 1198, 1136, 1089, 833, 777.

1-(4-Fluorophenoxy)-3,5-dimethoxybenzene (29)

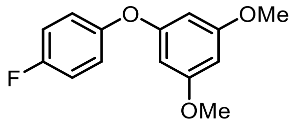

Prepared according to the *General Procedure for C-O Cross-Coupling, Variation B*. Using aryl triflate (143.1 mg, 0.500 mmol), 4-fluorophenol (67.3 mg, 0.600 mmol), *μ*OMs-Pd-G4 dimer•MeCN (4.3 mg, 0.005 mmol, 2 mol% Pd) and AdBippyPhos (13.3 mg, 0.02 mmol, 4 mol%) afforded, after purification by silica gel column chromatography (95:5 pentane/EtOAc), the title compound (87 mg, 0.350 mmol, 70%) as a light yellow oil.

^{1}H NMR (500 MHz, $CDCl_3$): δ 7.06 – 6.98 (m, 4H), 6.21 (t, *J* = 2.2 Hz, 1H), 6.12 (d, *J* = 2.1 Hz, 2H), 3.75 (s, 6H).

^{13}C{^{1}H} NMR (126 MHz, $CDCl_3$): δ 161.8, 159.8, 159.1 (d, *J* = 243.3 Hz), 152.5 (d, *J* = 2.3 Hz), 121.1 (d, *J* = 8.5 Hz), 116.4 (d, *J* = 23.4 Hz), 96.8, 95.4, 55.5.

^{19}F NMR (376 MHz, $CDCl_3$): δ -119.70 (tt, *J* = 7.8, 4.9 Hz).

HMRS (ESI$^+$, *m/z*): calcd. for $C_{14}H_{13}FO_3$+H^+: 249.0921 $[M+H]^+$. Found: 249.0929.

ν (ATR)/cm^{-1} (neat): 2959, 2839, 1591, 1499, 1197, 1151, 1129, 1011, 826.

1-(4-Fluorophenoxy)-3-(trifluoromethyl)benzene (30)

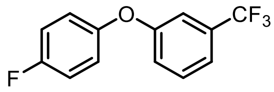

Prepared according to the *General Procedure for C-O Cross-Coupling, Variation B*. Using aryl triflate (147.1 mg, 0.500 mmol), 4-fluorophenol (67.3 mg, 0.600 mmol), *μ*OMs-Pd-G4 dimer•MeCN (8.5 mg, 0.01 mmol, 4 mol% Pd) and AdBippyPhos (26.5 mg, 0.04 mmol, 8 mol%) afforded, after purification by silica gel column chromatography (100% pentane), the title compound (97 mg, 0.379 mmol, 76%) as a colourless oil.

^{1}H NMR (500 MHz, $CDCl_3$): δ 7.43 (app. t, *J* = 8.0 Hz, 1H), 7.34 (app. d, *J* = 7.8 Hz, 1H), 7.23 – 7.18 (m, 1H), 7.12 (app. dd, *J* = 8.0, 2.4 Hz, 1H), 7.11 – 7.05 (m, 2H), 7.04 – 6.99 (m, 2H).

^{13}C{^{1}H} NMR (126 MHz, $CDCl_3$): δ 159.5 (d, *J* = 243.8 Hz), 158.4, 152.0 (d, *J* = 2.6 Hz), 132.4 (q, *J* = 33.2 Hz), 130.5, 123.8 (q, *J* = 272.7 Hz), 121.3 (d, *J* = 8.3 Hz), 121.1, 119.7 (q, *J* = 3.7 Hz), 116.8 (d, *J* = 23.5 Hz), 114.9 (q, *J* = 3.7 Hz).

^{19}F NMR (377 MHz, $CDCl_3$): δ -62.75 (s, 3F), -118.64 (tt, *J* = 8.0, 4.6 Hz, 1F).

MS (EI): calcd. for $C_{13}H_8OF_4^+$ ([M]$^+$): 256.05058. Found: 256.04996.

ν (ATR)/cm^{-1} (neat): 1501, 1449, 1325, 1124, 913, 832, 698.

1-Fluoro-4-(4-(trifluoromethyl)phenoxy)benzene (31)

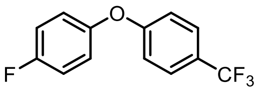

Prepared according to the *General Procedure for C-O Cross-Coupling, Variation B*. Using aryl triflate (147.1 mg, 0.500 mmol), 4-fluorophenol (67.3 mg, 0.600 mmol), *μ*OMs-Pd-G4 dimer•1/5Et_2O (7.8 mg, 0.01 mmol, 4 mol% Pd) and AdBippyPhos (26.5 mg, 0.02 mmol, 8 mol%) afforded, after purification by silica gel column chromatography (100% pentane), the title compound (110 mg, 0.429 mmol, 86%) as a yellow oil.

^{1}H NMR (500 MHz, $CDCl_3$): δ 7.57 (d, *J* = 8.7 Hz, 2H), 7.12 – 7.06 (m, 2H), 7.06 – 7.03 (m, 2H), 7.01 (d, *J* = 8.7 Hz, 2H).

$^{13}C\{^1H\}$ NMR (126 MHz, $CDCl_3$): δ 160.9, 159.7 (d, *J* = 243.6 Hz), 151.6 (d, *J* = 2.6 Hz), 127.3 (q, *J* = 3.7 Hz), 125.0 (q, *J* = 33.5 Hz), 124.3 (q, *J* = 271.8 Hz), 121.7 (d, *J* = 9.0 Hz), 117.5, 116.9 (d, *J* = 22.8 Hz).

^{19}F NMR (376 MHz, $CDCl_3$): δ -61.78 (s, 3F), -118.24 (tt, *J* = 8.1, 4.6 Hz).

MS (EI): calcd. for $C_{13}H_8OF_4^+$ ([M]$^+$): 256.05058. Found: 256.04951.

ν (ATR)/cm^{-1} (neat): 3056, 1616, 1497, 1323, 1218, 1064, 834.

4-(4-Fluorophenoxy)benzonitrile (32)

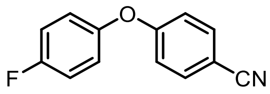

Prepared according to the *General Procedure for C-O Cross-Coupling, Variation A*. Using aryl triflate (125.6 mg, 0.500 mmol), 4-fluorophenol (67.3 mg, 0.600 mmol), μOMs-Pd-G4 dimer•3/10Et_2O (3.9 mg, 0.005 mmol, 2 mol% Pd) and AdBippyPhos (13.3 mg, 0.02 mmol, 4 mol%) afforded, after purification by silica gel column chromatography (0-3% EtOAc in pentane), the title compound (97 mg, 0.455 mmol, 91%) as a white crystalline solid.

Characterisation data were consistent with literature values: ^{1}H, ^{13}C and ^{19}F NMR,[44] m.p.,[45] and IR.[46]

^{1}H NMR (400 MHz, $CDCl_3$): δ 7.60 (d, *J* = 9.0 Hz, 2H), 7.14 – 7.07 (m, 2H), 7.07 – 7.01 (m, 2H), 6.97 (d, *J* = 9.0 Hz, 2H).

^{13}C{^{1}H} NMR (101 MHz, $CDCl_3$): δ 161.9, 159.9 (d, *J* = 244.6 Hz), 150.7 (d, *J* = 2.7 Hz), 134.3, 122.2 (d, *J* = 8.9 Hz), 118.9, 117.7, 117.0 (d, *J* = 24.0 Hz), 106.1.

^{19}F NMR (376 MHz, $CDCl_3$): δ -117.2 (tt, *J* = 7.9, 4.5 Hz).

HMRS (ESI$^+$, *m/z*): calcd. for $C_{13}H_8FNO+Na^+$: 236.0482 $[M+Na]^+$. Found: 236.0476.

m. p./ °C: 69-70.

ν (ATR)/cm^{-1} (neat): 3063, 2222, 1604, 1491, 1249, 1163, 831, 543.

1,3-Difluoro-5-(4-fluorophenoxy)benzene (33)

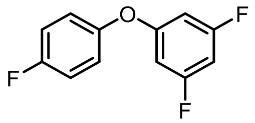

Prepared according to the *General Procedure for C-O Cross-Coupling, Variation A*. Using aryl triflate (131.1 mg, 0.500 mmol), 4-fluorophenol (67.3 mg, 0.600 mmol), *µ*OMs-Pd-G4 dimer•1/5Et_2O (7.8 mg, 0.01 mmol, 4 mol% Pd) and AdBippyPhos (26.5 mg, 0.02 mmol, 8 mol%) afforded, after purification by silica gel column chromatography (100% pentane), the title compound (194 mg, 0.419 mmol, 84%) as a colourless oil.

^{1}H NMR (400 MHz, $CDCl_3$): δ 7.13 – 7.00 (m, 4H), 6.51 (tt, *J* = 8.7, 2.3 Hz, 1H), 6.44 (dd, *J* = 8.5, 2.3 Hz, 2H).

^{13}C{^{1}H} NMR (101 MHz, $CDCl_3$): δ 165.1 – 162.3 (m), 160.5 – 160.1 (m), 159.8 (d, *J* = 243.6 Hz), 151.2 (d, *J* = 2.6 Hz), 121.9 (d, *J* = 8.3 Hz), 116.9 (d, *J* = 24.2 Hz), 101.4 – 100.9 (m), 98.8 – 98.0 (m).

^{19}F NMR (376 MHz, $CDCl_3$): δ -108.38 (app. t, *J* = 8.5 Hz, 2F), -117.77 (tt, *J* = 8.1, 4.6 Hz, 1F).

MS (EI): calcd. for $C_{12}H_7OF_3^+$ ([M]$^+$): 224.04435. Found: 224.04420.

ν (ATR)/cm^{-1} (neat): 2918, 1623, 1599, 1501, 1463, 1195, 1115, 1009, 832.

1-Fluoro-4-(4-nitrophenoxy)benzene (34)

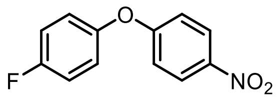

Prepared according to the *General Procedure for C-O Cross-Coupling, Variation A*. Using aryl triflate (135.6 mg, 0.500 mmol), 4-fluorophenol (67.3 mg, 0.600 mmol), *μ*OMs-Pd-G4 dimer•1/5Et_2O (7.8 mg, 0.01 mmol, 4 mol% Pd) and AdBippyPhos (26.5 mg, 0.02 mmol, 8 mol%) afforded, after purification by silica gel column chromatography (100% pentane), the title compound (105 mg, 0.450 mmol, 90%) as a beige solid.

Characterisation data were consistent with literature values: ^{1}H, ^{13}C, ^{19}F NMR, m. p. and IR.[47]

^{1}H NMR (400 MHz, $CDCl_3$): δ 8.20 (d, *J* = 9.1 Hz, 2H), 7.17 – 7.03 (m, 4H), 6.99 (d, *J* = 9.1 Hz, 2H).

^{13}C{^{1}H} NMR (101 MHz, $CDCl_3$): δ 163.6, 160.1 (d, *J* = 244.3 Hz), 150.6 (d, *J* = 3.2 Hz), 142.8, 126.1, 122.3 (d, *J* = 8.5 Hz), 117.2 (d, *J* = 23.6 Hz), 116.9.

^{19}F NMR (376 MHz, $CDCl_3$): δ -116.85 (tt, *J* = 7.9, 4.6 Hz).

HMRS (ESI$^+$, *m/z*): calcd. for $C_{12}H_8FNO_3$+H^+: 234.0561 $[M+H]^+$. Found: 234.0571.

m. p./ °C: 72-73

ν (ATR)/cm^{-1} (neat): 3078, 1586, 1512, 1486, 1341, 1248, 1188, 1110, 847, 749, 531.

1-Fluoro-3-(4-(trifluoromethyl)phenoxy)benzene (36)

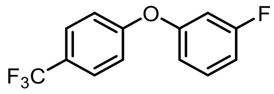

Prepared according to the *General Procedure for C-O Cross-Coupling, Variation A*, using aryl triflate (147.1 mg, 0.500 mmol), 3-fluorophenol (67.3 mg, 0.600 mmol), *μ*OMs-Pd-G4 dimer•1/5Et$_2$O (7.8 mg, 0.01 mmol, 4 mol% Pd) and AdBippyPhos (26.5 mg, 0.02 mmol, 8 mol%). Due to product volatility, yield was determined by quantitative ^{19}F NMR spectroscopic analysis *vs* internal standard (α,α,α-trifluorotoluene) and characterisation was performed *in situ*, following careful concentration *in vacuo* but without full purification. Representative 1D and 2D spectra are shown in Figures S16-S20; signals for the major contaminant (4-trifluoromethylphenyl triflate) are indicated with grey boxes.

^{1}H NMR (400 MHz, CDCl$_3$): δ 7.61 (d, *J* = 8.4 Hz, 2H), 7.33 (app. td, *J* = 8.3, 6.6 Hz, 1H), 7.08 (d, *J* = 8.3 Hz, 2 H), 6.89 (app. tdd, *J* = 8.3, 2.5, 0.9 Hz, 1H), 6.83 (dd, *J* = 8.1, 2.3 Hz, 1H), 6.76 (app. td, *J* = 9.8, 2.4 Hz, 1H).

^{13}C{^{1}H} NMR (101 MHz, CDCl$_3$): δ 163.7 (d, *J* = 247.8 H5), 159.7, 157.4 (d, *J* = 10.4 Hz), 131.0 (d, *J* = 9.5 Hz), 128.4 (q, *J* = 3.7 Hz), 125.8 (q, *J* = 32.7 Hz), 124.2 (q, *J* = 271.2 Hz), 118.7 (s), 115.2 (d, *J* = 3.2 Hz), 111.4 (d, *J* = 21.2 Hz), 107.4 (d, *J* = 24.1 Hz).

^{19}F NMR (376 MHz, CDCl$_3$): δ -61.89 (s, 3F), -110.24 (ddd, *J* = 9.8, 8.3, 6.6 Hz, 1F).

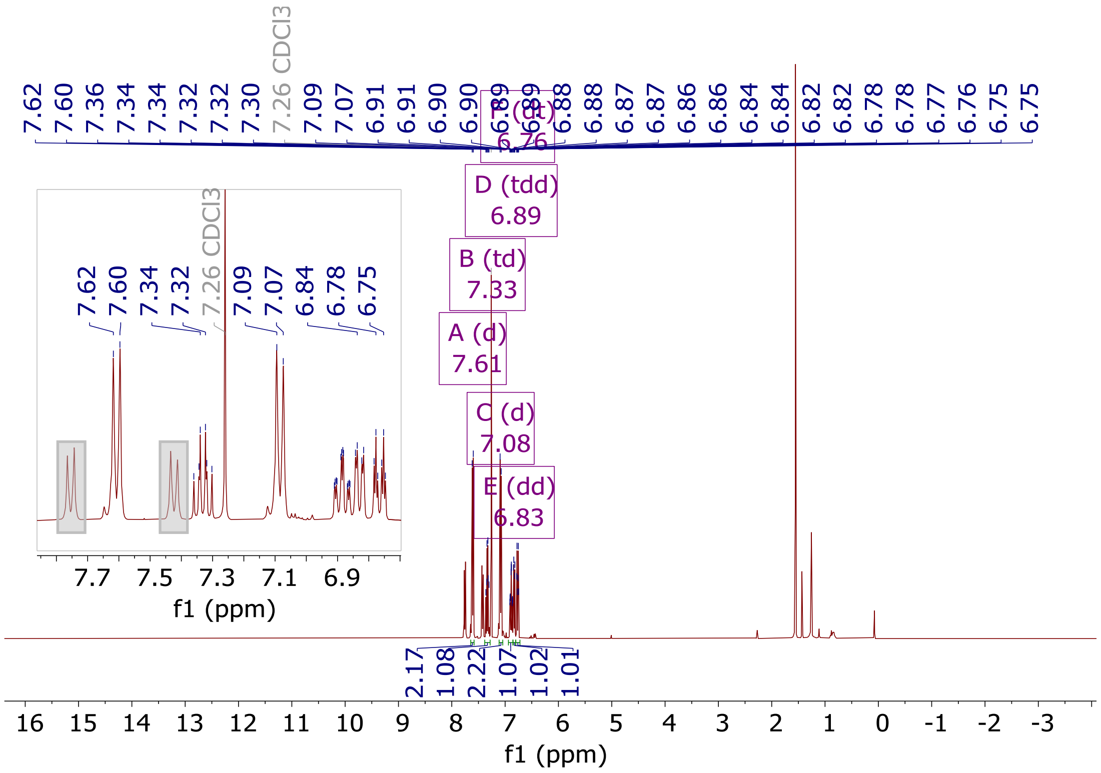

***Figure S16.** ^{1}H NMR spectrum (400 MHz, $CDCl_3$) of 1-fluoro-3-(4-(trifluoromethyl)phenoxy) benzene **36**.*

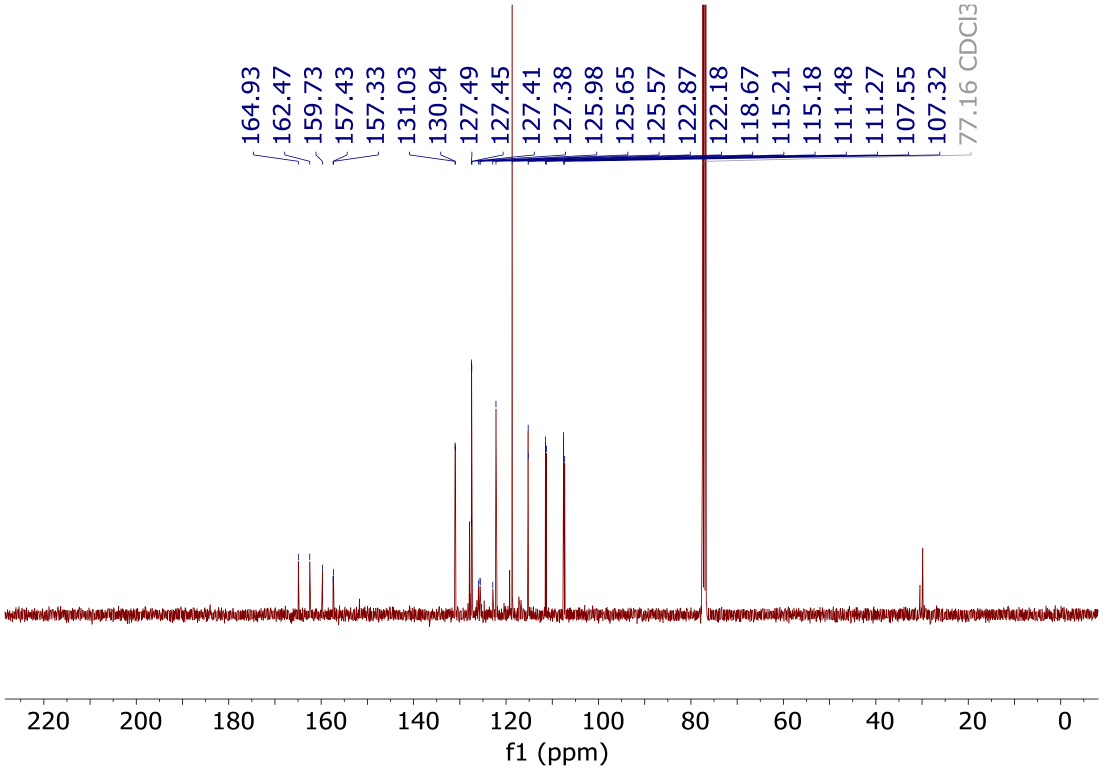

***Figure S17.** $^{13}C\{^{1}H\}$ NMR spectrum (101 MHz, $CDCl_3$) of 1-fluoro-3-(4-(trifluoromethyl)phenoxy) benzene **36**.*

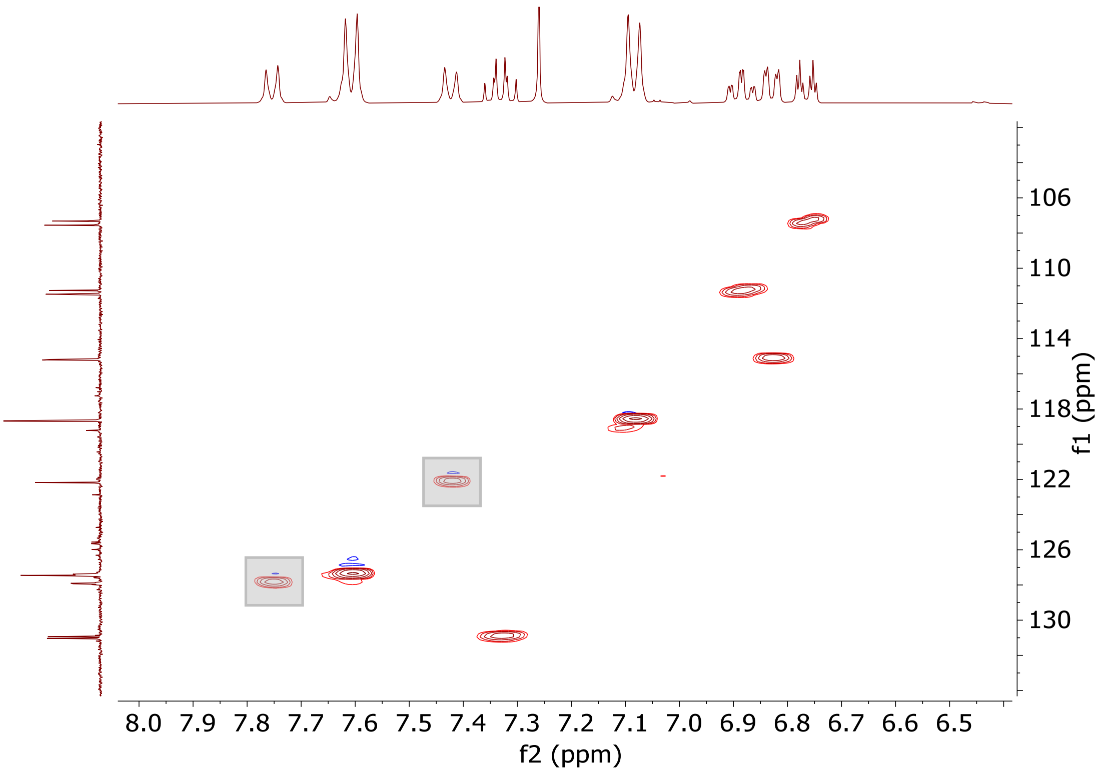

Figure S18. *HSQC spectrum (400 MHz, $CDCl_3$) of 1-fluoro-3-(4-(trifluoromethyl)phenoxy) benzene* ***36****.*

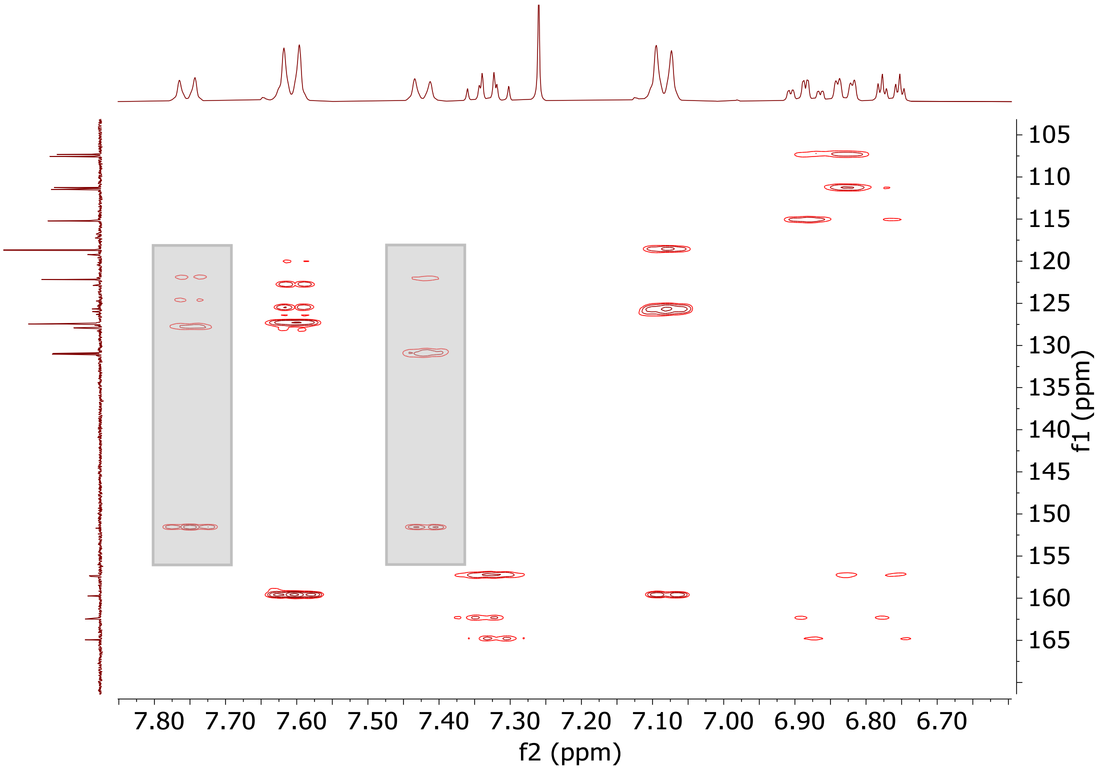

Figure S19. *HMBC spectrum (400 MHz, $CDCl_3$) of 1-fluoro-3-(4-(trifluoromethyl)phenoxy) benzene* ***36****.*

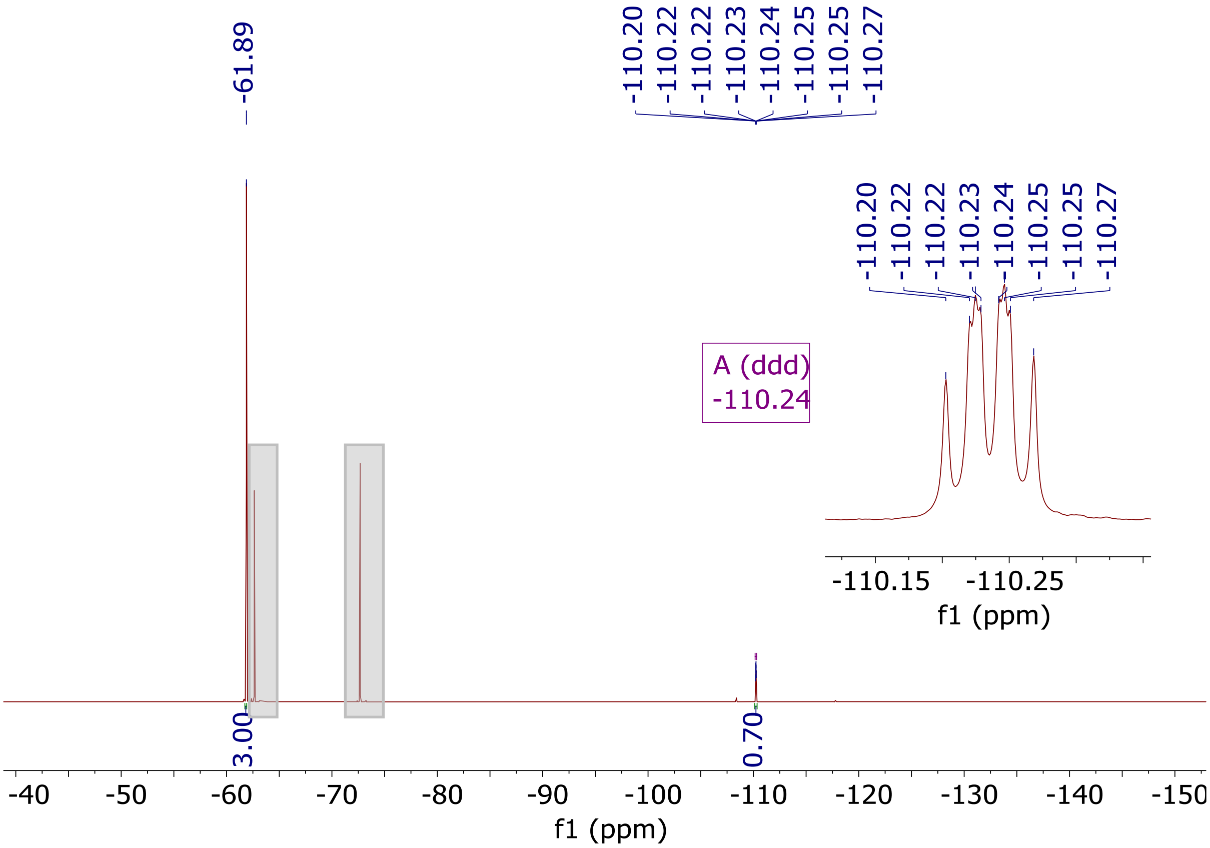

Figure S20. *^{19}F NMR (376 MHz, $CDCl_3$) of 1-fluoro-3-(4-(trifluoromethyl)phenoxy) benzene* ***36***.

Methyl (*S*)-2,5,7,8-tetramethyl-6-((4-methyl-2-oxo-2*H*-chromen-7-yl)oxy)chromane-2-carboxylate (37)

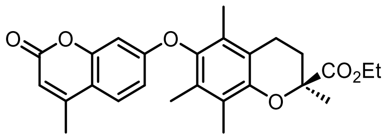

Prepared according to the *General Procedure for C-O Cross-Coupling, Variation B*. Using aryl triflate (154.1 mg, 0.500 mmol), **3a** (166.9 mg, 0.600 mmol), *μ*OMs-Pd-G4 dimer•MeCN (8.5 mg, 0.01 mmol, 4 mol% Pd) and AdBippyPhos (26.5 mg, 0.04 mmol, 8 mol%) afforded, after purification by silica gel column chromatography (0-20% EtOAc in pentane), the title compound (251 mg, 0.355 mmol, 71%) as a colourless gel.

^{1}H NMR (400 MHz, $CDCl_3$): δ 7.49 (d, *J* = 8.8 Hz, 1H), 6.83 (dd, *J* = 8.7, 2.5 Hz, 1H), 6.55 (d, *J* = 2.3 Hz, 1H), 6.12 (q, *J* = 1.3 Hz, 1H), 4.23 – 4.09 (m, 2H), 2.70 – 2.60 (m, 1H), 2.58 – 2.50 (m, 1H), 2.49 – 2.43 (m, 1H), 2.39 (d, *J* = 1.2 Hz, 3H), 2.20 (s, 3H), 2.00 (s, 3H), 1.96 – 1.86 (m, 4H), 1.64 (s, 3H), 1.21 (app. t, *J* = 7.2 Hz, 3H).

$^{13}C\{^1H\}$ NMR (101 MHz, $CDCl_3$): δ 173.6, 162.0, 161.2, 155.5, 152.6, 149.5, 143.6, 128.1, 126.0, 125.9, 123.9, 118.0, 114.2, 112.4, 112.2, 102.6, 77.4, 61.3, 30.4, 25.4, 21.0, 18.8, 14.3, 12.9, 12.03, 11.99.

HMRS (ESI$^+$, *m/z*): calcd. for $C_{26}H_{28}O_6$+H^+: 437.1959 $[M+H]^+$. Found: 437.1948.

ν (ATR)/cm^{-1} (neat): 2981, 2930, 1725, 1611, 1560, 1409, 1272, 1130, 848, 814, 731, 667.

Methyl (2*R*)-2-((*tert*-butoxycarbonyl)amino)-3-(4-(((2*R*)-2,5,7,8-tetramethyl-2-(4,8,12-trimethyltridecyl)chroman-6-yl)oxy)phenyl)propanoate (38)

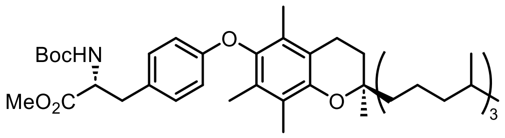

Prepared according to the *General Procedure for C-O Cross-Coupling, Variation B*. Using aryl triflate (213.7 mg, 0.500 mmol), (±)-α-Tocopherol (258.4 mg, 0.600 mmol), *μ*OMs-Pd-G4 dimer•MeCN (8.5 mg, 0.01 mmol, 4 mol% Pd) and AdBippyPhos (26.5 mg, 0.04 mmol, 8 mol%) afforded, after purification by silica gel column chromatography (0-10% EtOAc in pentane), the title compound (251 mg, 0.355 mmol, 71%) as a bright yellow gel.

N. B. The product is a mixture of diastereoisomers due to the starting material side-chain.

^{1}H NMR (400 MHz, CDCl$_3$): δ 7.00 (d, *J* = 8.6 Hz, 2H), 6.69 (d, *J* = 8.6 Hz, 2H), 5.00 (d, major rotamer, *J* = 8.6 Hz, 0.86H), 4.70 (s, br., minor rotamer, 0.14H), 4.62 – 4.51 (m, major rotamer, 85H), 4.38 (s, br., minor rotamer 0.15H), 3.72 (s, 3H), 3.11 – 2.92 (m, 2H), 2.63 (t, *J* = 6.5 Hz, 2H), 2.14 (s, 3H), 2.02 (s, 3H), 1.98 (s, 3H), 1.93 – 1.76 (m, 2H), 1.70 – 1.46 (m, 6H), 1.44 (s, 9H), 1.41 – 1.31 (m, 6H), 1.29 (s, 3H), 1.28 – 1.03 (m, 9H), 0.93 – 0.85 (m, 12H).

^{13}C{^{1}H} NMR (101 MHz, CDCl$_3$): δ 172.6, 158.1, 155.2, 148.8, 143.5, 130.5, 128.3, 128.25, 126.4, 123.4, 118.0, 114.9, 80.0, 75.2, 54.7, 52.3, 40.2, 39.5, 37.8, 37.7, 37.63, 37.6, 37.55, 37.54, 37.5, 37.4, 32.94, 32.92, 32.8, 31.42, 31.38, 28.4, 28.1, 25.0, 24.9, 24.6, 24.0, 22.9, 22.8, 21.2, 20.8, 19.9, 19.83, 19.76, 13.0, 12.2, 11.9.

HMRS (ESI^{+}, *m/z*): calcd. for $C_{44}H_{69}NO_6$+Na^{+}: 730.5017 [M+Na]$^{+}$. Found: 730.4995.

ν (ATR)/cm^{-1} (neat): 2925, 1747, 1717, 1505, 1250, 1221, 1081, 839, 692.

tert-Butyl 3-(2-(1,3-dioxoisoindolin-2-yl)ethyl)-5-(((8R,9S,13S,14S)-13-methyl-17-oxo-7,8,9,11,12,13,14,15,16,17-decahydro-6_H_-cyclopenta[a]phenanthren-3-yl)oxy)-1_H_-indole-1-carboxylate (39)

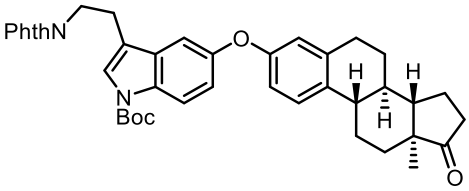

Prepared according to the *General Procedure for C-O Cross-Coupling, Variation B*. Using aryl triflate (262,2 mg, 0.500 mmol), estrone (162.2 mg, 0.600 mmol), *μ*OMs-Pd-G4 dimer•MeCN (8.5 mg, 0.01 mmol, 4 mol% Pd) and AdBippyPhos (26.5 mg, 0.04 mmol, 8 mol%) afforded, after purification by silica gel column chromatography (0-30% EtOAc in pentane), the title compound (244 mg, 0.370 mmol, 74%) as a light yellow crystalline solid.

^{1}H NMR (400 MHz, CDCl$_3$): δ 8.08 (d, *J* = 8.9 Hz, 1H), 7.89 – 7.78 (m, 2H), 7.71 (dd, *J* = 5.4, 3.0 Hz, 2H), 7.49 (s, 1H), 7.29 (d, *J* = 2.3 Hz, 1H), 7.21 (d, *J* = 8.6 Hz, 1H), 7.01 (dd, *J* = 8.9, 2.3 Hz, 1H), 6.76 (dd, *J* = 8.6, 2.6 Hz, 1H), 6.69 (d, *J* = 2.6 Hz, 1H), 4.03 – 3.94 (m, 2H), 3.05 – 2.99 (m, 2H), 2.89 – 2.82 (m, 2H), 2.55 – 2.45 (m, 1H), 2.44 – 2.36 (m, 1H), 2.33 – 2.23 (m, 1H), 2.20 – 1.92 (m, 4H), 1.69 – 1.59 (m, 11H), 1.56 – 1.37 (m, 4H), 0.92 (s, 3H).

^{13}C{^{1}H} NMR (101 MHz, CDCl$_3$): δ 221.1, 168.3, 156.7, 152.5, 149.6, 138.2, 134.1, 134.0, 132.2, 132.1, 131.6, 126.6, 124.4, 123.4, 117.8, 117.3, 117.0, 116.3, 115.4, 109.7, 83.7, 50.6, 48.1, 44.2, 38.4, 37.6, 36.0, 31.7, 29.7, 28.3, 26.6, 26.0, 24.3, 21.7, 14.0.

HMRS (ESI$^+$, *m/z*): calcd. for $C_{41}H_{42}N_2O_6+Na^+$: 681.2935 [M+Na]$^+$. Found: 681.2936.

m. p./ °C: 163-165

ν (ATR)/cm^{-1} (neat): 2929, 1710, 1597, 1465, 1374, 1256, 1155, 1070, 715.

Methyl (1*S*,4a*S*,10a*R*)-1,4a-dimethyl-6-((4-methyl-2-oxo-2*H*-chromen-7-yl)oxy)-1,2,3,4,4a,9,10,10a-octahydrophenanthrene-1-carboxylate (40)

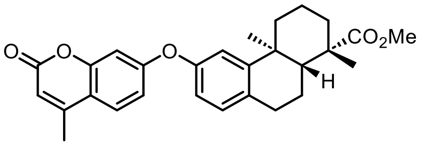

Prepared on 0.117 mmol scale, according to the *General Procedure for C-O Cross-Coupling, Variation B*. Adjusted work-up: The reaction was diluted with DCM (1 mL), washed with 2 M aq. HCl (2 × 1 mL), 2 M aq. NaOH (2 × 1 mL) and brine (1 mL). Using aryl triflate (36 mg, 0.117 mmol), **3b** (40.4 mg, 0.140 mmol), *μ*OMs-Pd-G4 dimer•MeCN (1.7 mg, 0.002 mmol, 4 mol% Pd), AdBippyPhos (6.0 mg, 0.009 mmol, 8 mol%), anhydrous PhMe (235 μL, 0.5 M) and PMP (31.8 μL, 0.16 mmol) afforded, after purification by silica gel column chromatography (0-20% EtOAc in pentane), the title compound (43 mg, 0.096 mmol, 71%) as an off-white solid.

^{1}H NMR (400 MHz, $CDCl_3$): δ 7.50 (d, *J* = 8.8 Hz, 1H), 7.06 (d, *J* = 8.2 Hz, 1H), 6.99 (d, *J* = 2.4 Hz, 1H), 6.90 (dd, *J* = 8.8, 2.4 Hz, 1H), 6.79 (dd, *J* = 8.2, 2.4 Hz, 1H), 6.76 (d, *J* = 2.4 Hz, 1H), 6.14 (q, *J* = 1.2 Hz, 1H), 3.66 (s, 3H), 2.92 (ddd, *J* = 17.0, 5.5, 1.4 Hz, 1H), 2.86 – 2.73 (m, 1H), 2.39 (d, *J* = 1.0 Hz, 3H), 2.31 – 2.17 (m, 2H), 2.16 – 2.09 (m, 1H), 2.06 – 1.89 (m, 2H), 1.64 – 1.58 (m, 1H), 1.55 (dd, *J* = 12.2, 1.4 Hz, 1H), 1.39 (app. td, *J* = 13.6, 4.3 Hz, 1H), 1.28 (s, 3H), 1.09 (app. td, *J* = 13.6, 4.3 Hz, 1H), 1.02 (s, 3H).

^{13}C{^{1}H} NMR (101 MHz, $CDCl_3$): δ 177.9, 161.9, 161.1, 155.1, 153.0, 152.4, 150.5, 132.4, 130.8, 125.8, 117.8, 117.7, 114.8, 113.9, 112.7, 104.7, 52.6, 51.4, 44.1, 39.4, 38.8, 37.6, 31.6, 28.6, 23.1, 21.0, 20.0, 18.8.

HMRS (ESI$^+$, *m/z*): calcd. for $C_{28}H_{30}O_5+H^+$: 447.2166 $[M+H]^+$. Found: 447.2165.

m. p./°C: 175-176.

ν (ATR)/cm^{-1} (neat): 2950, 1721, 1614, 1488, 1434, 1386, 1132, 853.

5.2 Attempted Synthesis of Tetra-*ortho*-Substituted Diaryl Ethers

Attempts to synthesise a tetra-*ortho*-substituted diaryl ether were unsuccessful (Table S12; reactions performed as per the *General Procedure for C-O Cross-Coupling, Variation B*). Given that the desired diaryl ether was not formed under standard reaction conditions with AdBippyPhos as ligand (entry 1), it was hypothesised that the steric demand associated with both the coupling partners and the ligand may be preventing the coupling. However, repeating the coupling with a less sterically-demanding ligand (CyBippyPhos, entry 2) was also unsuccessful. Up to this date, Pd-catalysed synthesis of tetra-*ortho*-substituted diaryl ethers has not been reported in the literature.

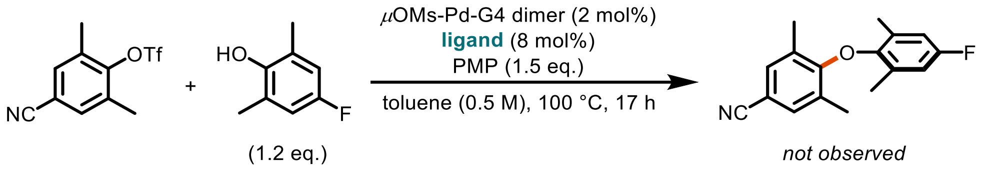

Entry	Ligand	% Conversion ArOTf	% Diaryl Ether
**1**	AdBippyPhos	60	1
**2**	CyBippyPhos	18	<1

Table S12. *Yields determined by quantitative ^{19}F NMR spectroscopic analysis vs internal standard (α,α,α-trifluorotoluene). Reactions carried out on 0.5 mmol scale.*

6. Compatibility with Enabling Technologies

6.1 Automated Dosing (Manuscript Scheme 4, Panel A)

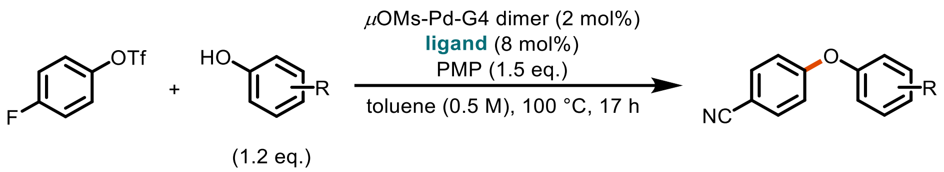

Reactions were performed on a 0.20 mmol scale, and were prepared in a N_2-filled glovebox.

<u>*Stock solution A.*</u> Using a Mettler Toledo Quantos weighing robot on the bench, AdBippyPhos (53.0 mg, 0.08 mmol) was weighed into a vial. The vial was taken into the glovebox, and toluene (1 mL) was added to give a homogeneous solution ([AdBippyPhos] = 0.08 M).

<u>*Stock solution B.*</u> Prepared in the glovebox: PMP (271 µL, 1.5 mmol) was added to a 1 mL volumetric flask, which was made to 1.00 mL with toluene to give a homogeneous solution ([PMP] = 1.5 M).

Using a Mettler Toledo Quantos weighing robot on the bench, each reaction vial was charged sequentially with μOMs-Pd-G4 dimer (3.39 mg, 0.004 mmol) and the appropriate phenol (0.24 mmol). The vials were taken into the glovebox, then *stock solution A* (200 µL) and 4- fluorophenyl triflate (33.4 µL, 0.20 mmol) were added. *Stock solution B* (200 µL) was then added to each vial. The vials were sealed, removed from the glovebox and heated at 100 °C for 17 h. Once the reactions had cooled to RT, $PhCF_3$ (internal standard), acetic acid (10 µL; quench) and DMSO (100 µL) were added. An aliquot (100 µL) was then removed and transferred into an NMR tube containing $CDCl_3$ (500 µL) for analysis by quantitative ^{19}F NMR spectroscopy.

6.2 Microwave (MW) Heating (Manuscript Scheme 4, Panel B)

Investigation and Optimisation of Conditions for MW Heating

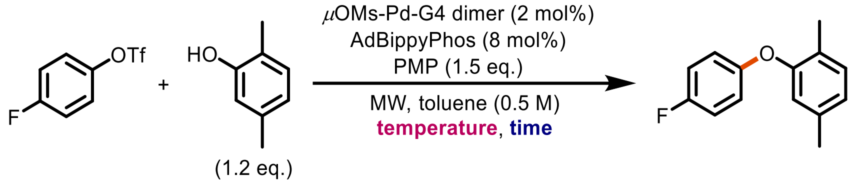

Entry	*T* /°C	Time	Atmosphere	% yield
1	100	1 h	Inert	9
2	150	1 h	Inert	100
3	150	1 h	Aerobic	89
4	100	17 h	Aerobic	trace
5	150	30 min	Aerobic	94
6	150	15 min	Aerobic	97
7	150	10 min	Aerobic	100

Table S13. *Yields determined by quantitative ^{19}F NMR spectroscopic analysis vs internal standard (4,4´-bis(trifluoromethyl)-1,1´-biphenyl). Reactions carried out on 0.3 mmol scale.*

Commentary. To test the compatibility of the C-O coupling with MW heating, the reaction was examined under a number of different conditions (Table S13). Increasing the temperature from 100 °C to 150 °C improved the product yield from 9% to 100% after just 1 h (entries 1-2). Control reactions demonstrated that the reaction works well under aerobic atmosphere at 150 °C (entry 3), whereas reactions heated to 100 °C require strict inert atmosphere (entriy 4). Variation of the reaction time (entries 5-7) indicated that the cross-coupling is complete in under 10 minutes at 150 °C, affording the desired diaryl ether in excellent yield without need to exclude air (entry 7).

Procedure. Reactions were performed as per the *General Procedure for C-O Cross-Coupling with MW Heating*, below.

General Procedure for C-O Cross-Coupling with MW Heating

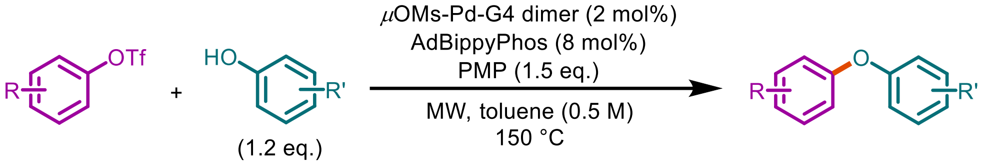

All experiments were performed on 0.3 mmol scale using Biotage 0.5-2 mL microwave reaction vials. Reactions were carried out in Biotage Microwave Synthesiser (Initiator 4. 2.1).

To a microwave vial in a following order were added: μOMs-Pd-G4 dimer•MeCN (5.1 mg, 0.006 mmol), AdBippyPhos (15.9 mg, 0.024 mmol),aryl triflate (0.30 mmol, 1.0 eq.), phenol (0.36 mmol, 1.2 eq.), anhydrous PhMe (0.6 mL) and PMP (81.4 μL, 0.45 mmol). The vial was sealed with a metal crimp cap fitted with a ptfe-faced silicone septum and placed in a microwave reactor. The reaction mixture was firstly pre-stirred for 1 minute at room temperature, then the temperature was set to 150 °C for the desired time. Once completed and cooled to RT, the reaction was quenched with acetic acid (10 μL) and DMSO (200 μL) was added, followed by internal standard (α,α,α-trifluorotoluene). An aliquot (100 μL) was then removed and transferred into an NMR tube containing $CDCl_3$ (500 μL) for analysis by quantitative ^{19}F NMR spectroscopy.

6.3 Telescoped Triflation / Coupling (Manuscript Scheme 4, Panel C)

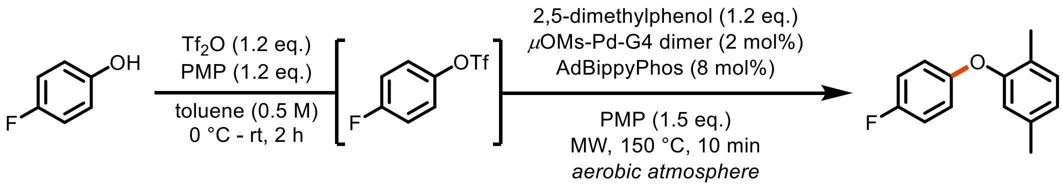

Anhydrous toluene (0.6 mL) was added to 4-fluorophenol (33.6 mg, 0.30 mmol) and 4,4′-bis(trifluoromethyl)-1,1′-biphenyl (internal standard) in a flame dried microwave vial sealed with a rubber septum. PMP (65.2 μL, 0.36 mmol) was added, the resulting solution was cooled to 0 °C in an ice bath, and triflic anhydride (60.6 μL, 0.36 mmol) was added dropwise. The reaction was allowed to warm to room temperature over 2 hours, then an aliquot was removed, diluted into $CDCl_3$ (500 μL) and analysed by quantitative ^{19}F NMR spectroscopy. The septum was removed, and 2,5-dimethylphenol (44.0 mg, 0.36 mmol), PMP (81.m μL, 0.45 mmol), μOMs-Pd-G4 dimer (μOMs-Pd-G4 dimer•MeCN (5.1 mg, 0.006 mmol), and AdBippyPhos (15.9 mg, 0.024 mmol) were added. The vial was sealed with a metal crimp cap fitted with a ptfe-faced silicone septum and placed in a microwave reactor (Biotage Microwave Synthesiser - Initiator 4. 2.1). The reaction was pre-stirred for 1 min at room temperature, then at 150 °C for 10 min. Once completed and cooled to room temperature, the reaction was quenched with acetic acid (10 μL) and DMSO (200 μL) was added. An aliquot (100 μL) was then removed and transferred into an NMR tube containing $CDCl_3$ (500 μL) for analysis by quantitative ^{19}F NMR spectroscopy.

6.4 Translation to Flow (Manuscript Table 2)

Synthesis of Fluazifop Methyl: Reaction Development in Batch

Screening reactions were performed using 5-(trifluoromethyl)pyridin-2-yl triflate (88.5 mmol, 0.30 mmol), methyl (*R*)-2-(4-hydroxyphenoxy)propanoate (70.6 mg, 0.36 mmol), and other reagents as indicated in Manuscript Table 2. Reactions were assembled and analysed as per the *General Procedure for C-O Cross-Coupling with MW Heating*, with either microwave heating, or conventional heating in a pre-heated aluminium block.

Procedure for Flow Synthesis of Fluazifop Methyl

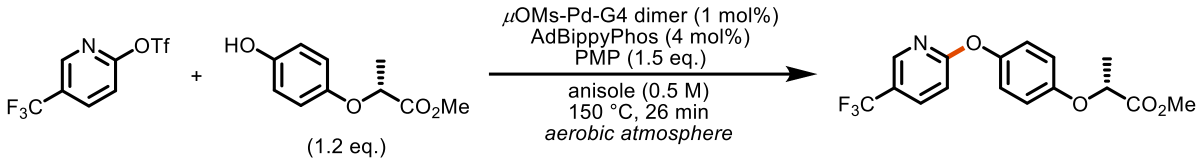

An 8 dram vial was charged with reagents in the following order: *μ*OMs-Pd-G4 dimer•1/5Et$_2$O (31.2 mg, 0.04 mmol, 1 mol%), AdBippyPhos (106.1 mg, 0.16 mmol, 4 mol%), methyl (*R*)-(+)-2-(4-hydroxyphenoxy)propionate (0.941g, 4.8 mmol), 5-(trifluoromethyl)pyridin-2-yl triflate (737.8 μL, 4.0 mmol), anisole (8 mL, 0.5 M) and PMP (1.1 mL, 6.0 mmol). The vial was sealed and shaken vigorously to ensure reaction mixture homogeneity.

The flow set-up is shown in Figure S21, below. The reaction mixture was drawn into a 10 mL Luer-lock syringe (internal diameter = 16 mm). The syringe was fitted to PTFE tubing (total length = 2 m, internal diameter 1.6 mm), which was coiled and immersed in a silicone oil bath (internal volume of coiled PTFE tubing in bath = 4.0 mL). The outlet of the PTFE tubing and was connected to a 100 mL Schlenk round-bottomed flask (the outlet) that was placed in an ice bath and open to N_2. The oil bath was heated to 150 °C, then the reaction mixture was passed through the flow set-up using a syringe pump (flow rate = 0.4 mL/min, residence time = 10.05 min). After collection of the crude reaction mixture, an extra amount of anisole (4 mL) was immediately passed through the PTFE coil at the same flow rate to ensure full product recovery.

The reaction mixture was diluted with Et$_2$O (15 mL), washed with 2 M aq. HCl (3 × 10 mL), 2 M aq. NaOH (3 × 10 mL) and brine (15 mL). The organic extracts were then dried over anhydrous Na_2SO_4 and concentrated *in vacuo*. After purification by silica gel column chromatography (0-5% EtOAc in pentane), the title compound (1.04 g, 3.05 mmol, 76%) was obtained as a bright yellow oil.

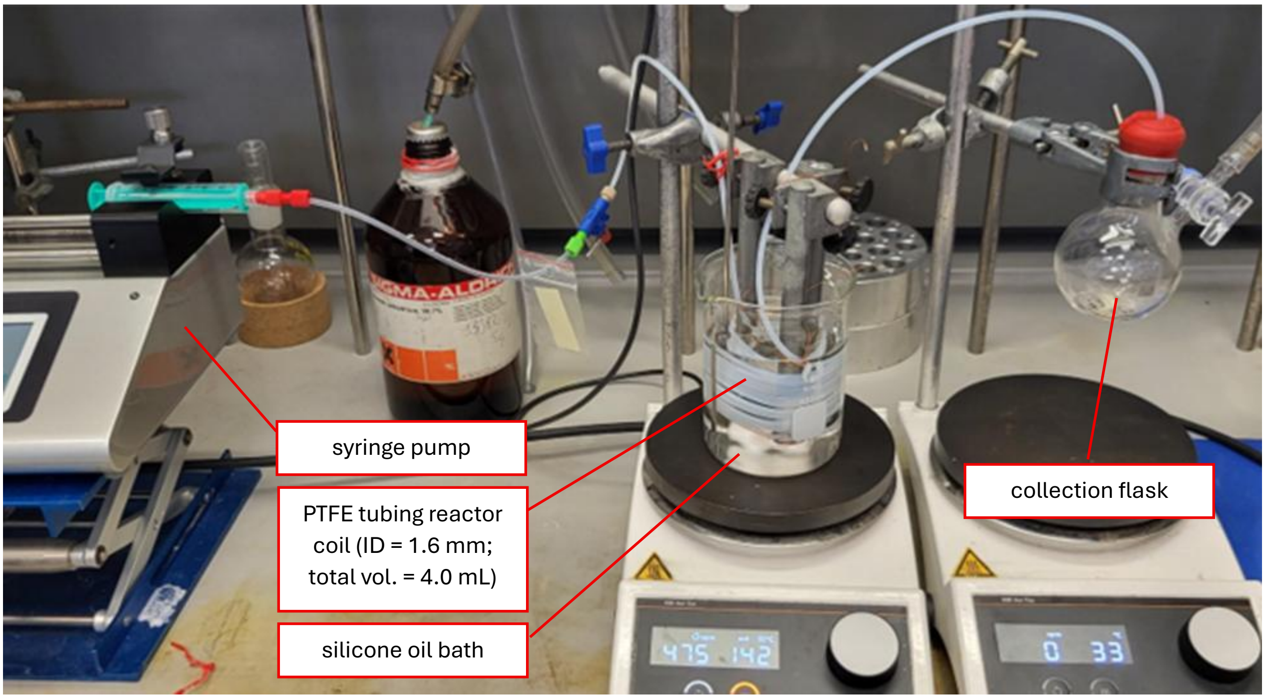

Figure S21. *Experimental set-up for reaction in flow.*

Methyl (*R*)-2-(4-((5-(trifluoromethyl)pyridin-2-yl)oxy)phenoxy)propanoate (41)

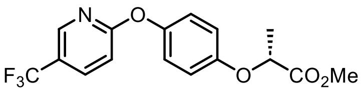

^{1}H NMR (400 MHz, $CDCl_3$): δ 8.45 – 8.40 (m, 1H), 7.87 (dd, *J* = 8.6, 2.2 Hz, 1H), 7.08 – 7.03 (m, 2H), 6.97 (d, *J* = 8.8 Hz, 1H), 6.95 – 6.89 (m, 2H), 4.75 (q, *J* = 6.8 Hz, 1H), 3.77 (s, 3H), 1.63 (d, *J* = 6.8 Hz, 3H).

^{13}C{^{1}H} NMR (101 MHz, $CDCl_3$): δ 172.7, 166.2, 155.2, 147.4, 145.6 (q, *J* = 4.6 Hz), 136.7 (q, *J* = 3.1 Hz), 123.8 (q, *J* = 271.4 Hz), 122.6, 121.5 (q, *J* = 33.1 Hz), 116.3, 111.2, 73.3, 52.5, 18.8.

^{19}F NMR (376 MHz, $CDCl_3$): δ -61.66 (s, 3F).

HMRS (ESI$^+$, *m/z*): calcd. for $C_{16}H_{14}FN_1O_4$+Na^+: 364.0767 [M+Na]$^+$. Found: 364.0770.

v(ATR)/cm^{-1} (neat): 2993, 1755, 1738, 1738, 1613, 1505, 1485, 1325, 1195, 1123, 836.

7. References

(1) Quesnelle Victor, C. A. . S. Directed Ortho Metalation (DoM)-Linked Corriu–Kumada, Negishi, and Suzuki–Miyaura Cross-Coupling Protocols: A Comparative Study. *Synthesis* **2018**, *50* (22), 4395–4412. https://doi.org/10.1055/s-0037-1611053.

(2) Pu, X.; Zhang, Y.; Su, M.; He, X.; Qiu, L. Palladium-Catalyzed Selective Buchwald–Hartwig C–N Coupling of Chloroaryl Triflates with Amines. *Tetrahedron Lett.* **2024**, *142*, 155096. https://doi.org/https://doi.org/10.1016/j.tetlet.2024.155096.

(3) Kale, A. P.; Pawar, G. G.; Kapur, M. Regioselectivity Switch Achieved in the Palladium Catalyzed α-Arylation of Enones by Employing the Modified Kuwajima–Urabe Conditions. *Org. Lett.* **2012**, *14* (7), 1808–1811. https://doi.org/10.1021/ol300401c.

(4) Taeufer, T.; Pospech, J. Palladium-Catalyzed Synthesis of N,N-Dimethylanilines via Buchwald–Hartwig Amination of (Hetero)Aryl Triflates. *J. Org. Chem.* **2020**, *85* (11), 7097–7111. https://doi.org/10.1021/acs.joc.0c00491.

(5) Taeufer, T.; Pospech, J. Palladium-Catalyzed Synthesis of N,N-Dimethylanilines via Buchwald–Hartwig Amination of (Hetero)Aryl Triflates. *J. Org. Chem.* **2020**, *85* (11), 7097–7111. https://doi.org/10.1021/acs.joc.0c00491.

(6) Henning, R.; Urbach, H. Diastereoselective Synthesis of Bicyclic Amino Acids via Ring Contraction of α-Chlorolactams. *Tetrahedron Lett.* **1983**, *24* (48), 5339–5342. https://doi.org/https://doi.org/10.1016/S0040-4039(00)87863-6.

(7) Cabri, W.; Candiani, I.; Bedeschi, A.; Penco, S.; Santi, R. ..Alpha.-Regioselectivity in Palladium-Catalyzed Arylation of Acyclic Enol Ethers. *J. Org. Chem.* **1992**, *57* (5), 1481–1486. https://doi.org/10.1021/jo00031a029.

(8) Oechsner, R. M.; Lindenmaier, I. H.; Fleischer, I. Nickel Catalyzed Cross-Coupling of Aryl and Alkenyl Triflates with Alkyl Thiols. *Org. Lett.* **2023**, *25* (10), 1655–1660. https://doi.org/10.1021/acs.orglett.3c00218.

(9) Cabri, W.; Candiani, I.; Bedeschi, A.; Penco, S.; Santi, R. ..Alpha.-Regioselectivity in Palladium-Catalyzed Arylation of Acyclic Enol Ethers. *J. Org. Chem.* **1992**, *57* (5), 1481–1486. https://doi.org/10.1021/jo00031a029.

(10) Pauvert, Y.; Charette, A. B. Asymmetric Synthesis of (−)-Cannabidiol (CBD), (−)-Δ9-Tetrahydrocannabinol (Δ9-THC) and Their Cis Analogs Using an Enantioselective Organocatalyzed Diels–Alder Reaction. *Org. Lett.* **2024**, *26* (29), 6081–6085. https://doi.org/10.1021/acs.orglett.4c01622.

(11) Subramanian, L. R.; Hanack, M.; Chang, L. W. K.; Imhoff, M. A.; Schleyer, P. v. R.; Effenberger, F.; Kurtz, W.; Stang, P. J.; Dueber, T. E. On Attempts at Solvolytic Generation of Aryl Cations. *J. Org. Chem.* **1976**, *41* (26), 4099–4103. https://doi.org/10.1021/jo00888a012.

(12) Schimler, S. D.; D. J. Froese, R.; C. Bland, D.; S. Sanford, M. Reactions of Arylsulfonate Electrophiles with NMe4F: Mechanistic Insight, Reactivity, and Scope. *J. Org. Chem.* **2018**, *83* (18), 11178–11190. https://doi.org/10.1021/acs.joc.8b01762.

(13) Stang, P. J.; Anderson, A. G. Hammett and Taft Substituent Constants for the Mesylate, Tosylate, and Triflate Groups. *J. Org. Chem.* **1976**, *41* (5), 781–785. https://doi.org/10.1021/jo00867a007.

(14) Gill, D.; Hester, A. J.; Lloyd-Jones, G. C. On the Preparation of Ortho-Trifluoromethyl Phenyl Triflate. *Org. Biomol. Chem.* **2004**, *2* (17), 2547–2548. https://doi.org/10.1039/B406803C.

(15) Byrne, T. J. M.; Mylrea, M. E.; Cuthbertson, J. D. A Redox-Relay Heck Approach to Substituted Tetrahydrofurans. *Org. Lett.* **2023**, *25* (13), 2361–2365. https://doi.org/10.1021/acs.orglett.3c00769.

(16) Mowery, M. E.; DeShong, P. Synthesis of Unsymmetrical Biaryls by Palladium-Catalyzed Cross Coupling Reactions of Arenes with Tetrabutylammonium Triphenyldifluorosilicate, a Hypervalent Silicon Reagent. *J. Org. Chem.* **1999**, *64* (9), 3266–3270. https://doi.org/10.1021/jo990072c.

(17) Kale, A. P.; Pawar, G. G.; Kapur, M. Regioselectivity Switch Achieved in the Palladium Catalyzed α-Arylation of Enones by Employing the Modified Kuwajima–Urabe Conditions. *Org. Lett.* **2012**, *14* (7), 1808–1811. https://doi.org/10.1021/ol300401c.

(18) Oechsner, R. M.; Lindenmaier, I. H.; Fleischer, I. Nickel Catalyzed Cross-Coupling of Aryl and Alkenyl Triflates with Alkyl Thiols. *Org. Lett.* **2023**, *25* (10), 1655–1660. https://doi.org/10.1021/acs.orglett.3c00218.

(19) Chang, J. W. W.; Chia, E. Y.; Chai, C. L. L.; Seayad, J. Scope of Direct Arylation of Fluorinated Aromatics with Aryl Sulfonates. *Org. Biomol. Chem.* **2012**, *10* (11), 2289–2299. https://doi.org/10.1039/C2OB06840K.

(20) Barf, T. A.; de Boer, P.; Wikström, H.; Peroutka, S. J.; Svensson, K.; Ennis, M. D.; Ghazal, N. B.; McGuire, J. C.; Smith, M. W. 5-HT1D Receptor Agonist Properties of Novel 2-[5-[[(Trifluoromethyl)Sulfonyl]Oxy]Indolyl]Ethylamines and Their Use as Synthetic Intermediates. *J. Med. Chem.* **1996**, *39* (24), 4717–4726. https://doi.org/10.1021/jm9604890.

(21) Zhao, M. M.; Zhang, H.; Iimura, S.; Bednarz, M. S.; Kanamarlapudi, R. C.; Yan, J.; Lim, N.-K.; Wu, W. Process Development of Tryptophan Hydroxylase Inhibitor LX1031, a Drug Candidate for the Treatment of Irritable Bowel Syndrome. *Org. Process Res. Dev.* **2020**, *24* (2), 261–273. https://doi.org/10.1021/acs.oprd.9b00520.

(22) Papst, S.; Noisier, A. F. M.; Brimble, M. A.; Yang, Y.; Krissansen, G. W. Synthesis and Biological Evaluation of Tyrosine Modified Analogues of the A4β7 Integrin Inhibitor Biotin-R8ERY. *Bioorg. Med. Chem.* **2012**, *20* (17), 5139–5149. https://doi.org/https://doi.org/10.1016/j.bmc.2012.07.010.

(23) Guo, P.; Wang, K.; Jin, W.-J.; Xie, H.; Qi, L.; Liu, X.-Y.; Shu, X.-Z. Dynamic Kinetic Cross-Electrophile Arylation of Benzyl Alcohols by Nickel Catalysis. *J. Am. Chem. Soc.* **2021**, *143* (1), 513–523. https://doi.org/10.1021/jacs.0c12462.

(24) Kundu, G.; Opincal, F.; Sperger, T.; Schoenebeck, F. Air-Stable PdI Dimer Enabled Remote Functionalization: Access to Fluorinated 1,1-Diaryl Alkanes with Unprecedented Speed. *Angew. Chem. Int. Ed.* **2022**, *61* (1), e202113667. https://doi.org/https://doi.org/10.1002/anie.202113667.

(25) Huang, H.; Wu, Y.; Zhang, W.; Feng, C.; Wang, B.-Q.; Cai, W.-F.; Hu, P.; Zhao, K.-Q.; Xiang, S.-K. Copper-Catalyzed Regioselective C-H Sulfonyloxylation of Electron-Rich Arenes with p-Toluenesulfonic Acid and Sulfonyloxylation of Aryl(Mesityl)Iodonium Sulfonates. *J. Org. Chem.* **2017**, *82* (6), 3094–3101. https://doi.org/10.1021/acs.joc.7b00081.

(26) Ganton, M. D.; Kerr, M. A. A Domino Amidation Route to Indolines and Indoles: Rapid Syntheses of Anhydrolycorinone, Hippadine, Oxoassoanine, and Pratosine. *Org. Lett.* **2005**, *7* (21), 4777–4779. https://doi.org/10.1021/ol052086c.

(27) Noël, T.; Maimone, T. J.; Buchwald, S. L. Accelerating Palladium-Catalyzed C�F Bond Formation: Use of a Microflow Packed-Bed Reactor. *Angew. Chem. Int. Ed.* **2011**, *50* (38), 8900–8903. https://doi.org/https://doi.org/10.1002/anie.201104652.

(28) Si, T.; Li, B.; Xiong, W.; Xu, B.; Tang, W. Efficient Cross-Coupling of Aryl/Alkenyl Triflates with Acyclic Secondary Alkylboronic Acids. *Org. Biomol. Chem.* **2017**, *15* (46), 9903–9909. https://doi.org/10.1039/C7OB02531A.

(29) Peng, H. M.; Webster, R. D. Investigation into Phenoxonium Cations Produced during the Electrochemical Oxidation of Chroman-6-Ol and Dihydrobenzofuran-5-Ol Substituted Compounds. *J. Org. Chem.* **2008**, *73* (6), 2169–2175. https://doi.org/10.1021/jo702415q.

(30) Peng, H. M.; Webster, R. D. Investigation into Phenoxonium Cations Produced during the Electrochemical Oxidation of Chroman-6-Ol and Dihydrobenzofuran-5-Ol Substituted Compounds. *J. Org. Chem.* **2008**, *73* (6), 2169–2175. https://doi.org/10.1021/jo702415q.

(31) Browne, E. C.; Langford, S. J.; Abbott, B. M. Synthesis and Effects of Conjugated Tocopherol Analogues on Peptide Nucleic Acid Hybridisation. *Org. Biomol. Chem.* **2013**, *11* (39), 6744–6750. https://doi.org/10.1039/C3OB41613E.

(32) Cui, Y.-M.; Yasutomi, E.; Otani, Y.; Ido, K.; Yoshinaga, T.; Sawada, K.; Ohwada, T. Design, Synthesis, and Characterization of BK Channel Openers Based on Oximation of Abietane Diterpene Derivatives. *Bioorg. Med. Chem.* **2010**, *18* (24), 8642–8659. https://doi.org/https://doi.org/10.1016/j.bmc.2010.09.072.

(33) Lapuh, M. I.; Dana, A.; Di Chenna, P. H.; Darses, B.; Durán, F. J.; Dauban, P. Late-Stage C–H Amination of Abietane Diterpenoids. *Org. Biomol. Chem.* **2019**, *17* (19), 4736–4746. https://doi.org/10.1039/C9OB00272C.

(34) Nguyen, D. M.; and Miles, D. H. Copper(I)-Catalyzed Cycloaddition of Methyl O-Propargylpodocarpate and Azides at Room Temperature. *Synth. Commun.* **2011**, *41* (12), 1759–1771. https://doi.org/10.1080/00397911.2010.492081.

(35) Dennis, J. M.; White, N. A.; Liu, R. Y.; Buchwald, S. L. Pd-Catalyzed C–N Coupling Reactions Facilitated by Organic Bases: Mechanistic Investigation Leads to Enhanced Reactivity in the Arylation of Weakly Binding Amines. *ACS Catal.* **2019**, *9* (5), 3822–3830. https://doi.org/10.1021/acscatal.9b00981.

(36) Nakayama, Y.; Yokoyama, N.; Nara, H.; Kobayashi, T.; Fujiwhara, M. An Efficient Synthesis of N-(Hetero)Arylcarbazoles: Palladium-Catalyzed Coupling Reaction between (Hetero)Aryl Chlorides and N-Carbazolylmagnesium Chloride. *Adv. Synth. Catal.* **2015**, *357* (10), 2322–2330. https://doi.org/https://doi.org/10.1002/adsc.201500301.

(37) Macomber, R. S. An Introduction to NMR Titration for Studying Rapid Reversible Complexation. *J. Chem. Educ.* **1992**, *69* (5), 375. https://doi.org/10.1021/ed069p375.

(38) Huang, J.; Ho, D. B.; Gaube, G.; Celuszak, H.; Becica, J.; Thomas, G. T.; Schley, N. D.; Leitch, D. C. A Thermally Stable, Alkene-Free Palladium Source for Oxidative Addition Complex Formation and High-Turnover Catalysis. *Organometallics* **2024**, *43* (20), 2403–2412. https://doi.org/10.1021/acs.organomet.4c00125.

(39) Matsushita, N.; Kashihara, M.; Formica, M.; Nakao, Y. Pd-Catalyzed Etherification of Nitroarenes. *Organometallics* **2021**, *40* (14), 2209–2214. https://doi.org/10.1021/acs.organomet.1c00183.

(40) Spiller, T. E.; Donabauer, K.; Brooks, A. F.; Witek, J. A.; Bowden, G. D.; Scott, P. J. H.; Sanford, M. S. Room-Temperature Photochemical Copper-Mediated Fluorination of Aryl Iodides. *Org. Lett.* **2024**, *26* (30), 6433–6437. https://doi.org/10.1021/acs.orglett.4c02227.

(41) Kumar, A.; Bhakuni, B. S.; Prasad, C. D.; Kumar, S.; Kumar, S. Potassium Tert-Butoxide-Mediated Synthesis of Unsymmetrical Diaryl Ethers, Sulfides and Selenides from Aryl Bromides. *Tetrahedron* **2013**, *69* (26), 5383–5392. https://doi.org/https://doi.org/10.1016/j.tet.2013.04.113.

(42) Mkrtchyan, S.; Shalimov, O.; Purohit, V. B.; Zapletal, J.; Prajapati, V. D.; Prajapati, R. V; Elumalai, D.; Garcia, M. G.; Filo, J.; Addová, G.; Benická, B.; Iaroshenko, V. O. Nanocellulose as Reaction Medium for FeCl3-Mediated Mechanochemical Deaminative Fluorination of (Hetero)Aromatic Amines. *Adv. Synth. Catal.* **2024**, *366* (15), 3269–3276. https://doi.org/https://doi.org/10.1002/adsc.202400303.

(43) Best, D.; Jean, M.; van de Weghe, P. Modular Synthesis of Arylacetic Acid Esters, Thioesters, and Amides from Aryl Ethers via Rh(II)-Catalyzed Diazo Arylation. *J. Org. Chem.* **2016**, *81* (17), 7760–7770. https://doi.org/10.1021/acs.joc.6b01426.

(44) Fang, X.; Qi, C.; Cao, X.; Ren, Z.-G.; Young, D. J.; Li, H.-X. Copper-Catalyzed O-Arylation of Phenols with Diazonium Salts. *Green Chem.* **2023**, *25* (20), 8068–8073. https://doi.org/10.1039/D3GC02541A.

(45) Buck, E.; Song, Z. J.; Tschaen, D.; Dormer, P. G.; Volante, R. P.; Reider, P. J. Ullmann Diaryl Ether Synthesis: Rate Acceleration by 2,2,6,6-Tetramethylheptane-3,5-Dione. *Org. Lett.* **2002**, *4* (9), 1623–1626. https://doi.org/10.1021/ol025839t.

(46) Pichette Drapeau, M.; Ollevier, T.; Taillefer, M. On the Frontier Between Nucleophilic Aromatic Substitution and Catalysis. *Chem. – A Eur. J.* **2014**, *20* (18), 5231–5236. https://doi.org/https://doi.org/10.1002/chem.201304164.

(47) Bispat, A. S.; Cardoso, F. C.; Hasan, M. M.; Dongol, Y.; Wilcox, R.; Lewis, R. J.; Duggan, P. J.; Tuck, K. L. Inhibition of N-Type Calcium Channels by Phenoxyaniline and Sulfonamide Analogues. *RSC Med. Chem.* **2024**, *15* (3), 916–936. https://doi.org/10.1039/D3MD00714F.

8. NMR Spectra

1b - ^{1}H NMR (500 MHz, $CDCl_3$)

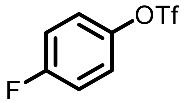

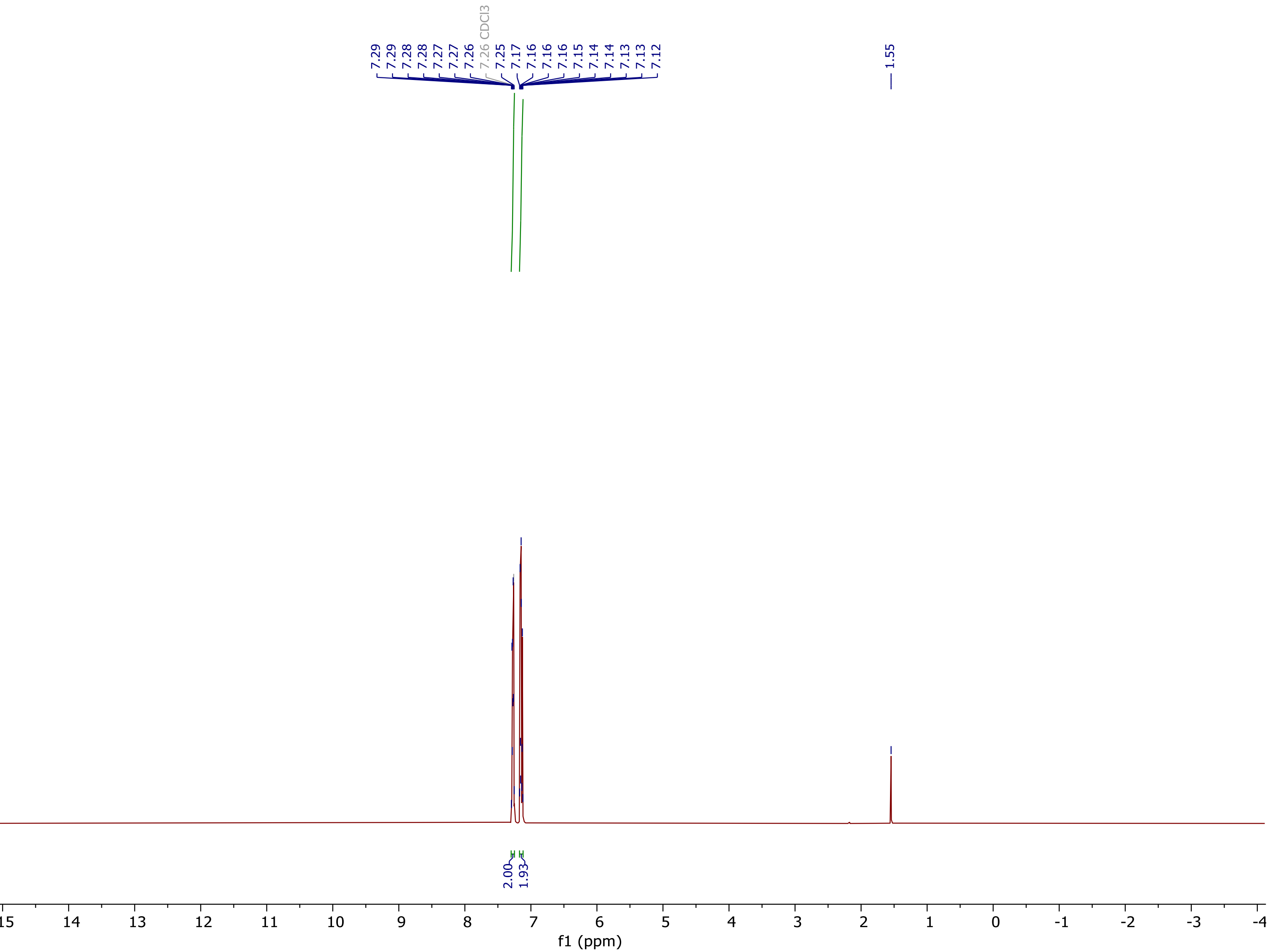

1b - $^{13}C\{^{1}H\}$ NMR (126 MHz, $CDCl_3$)

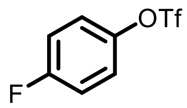

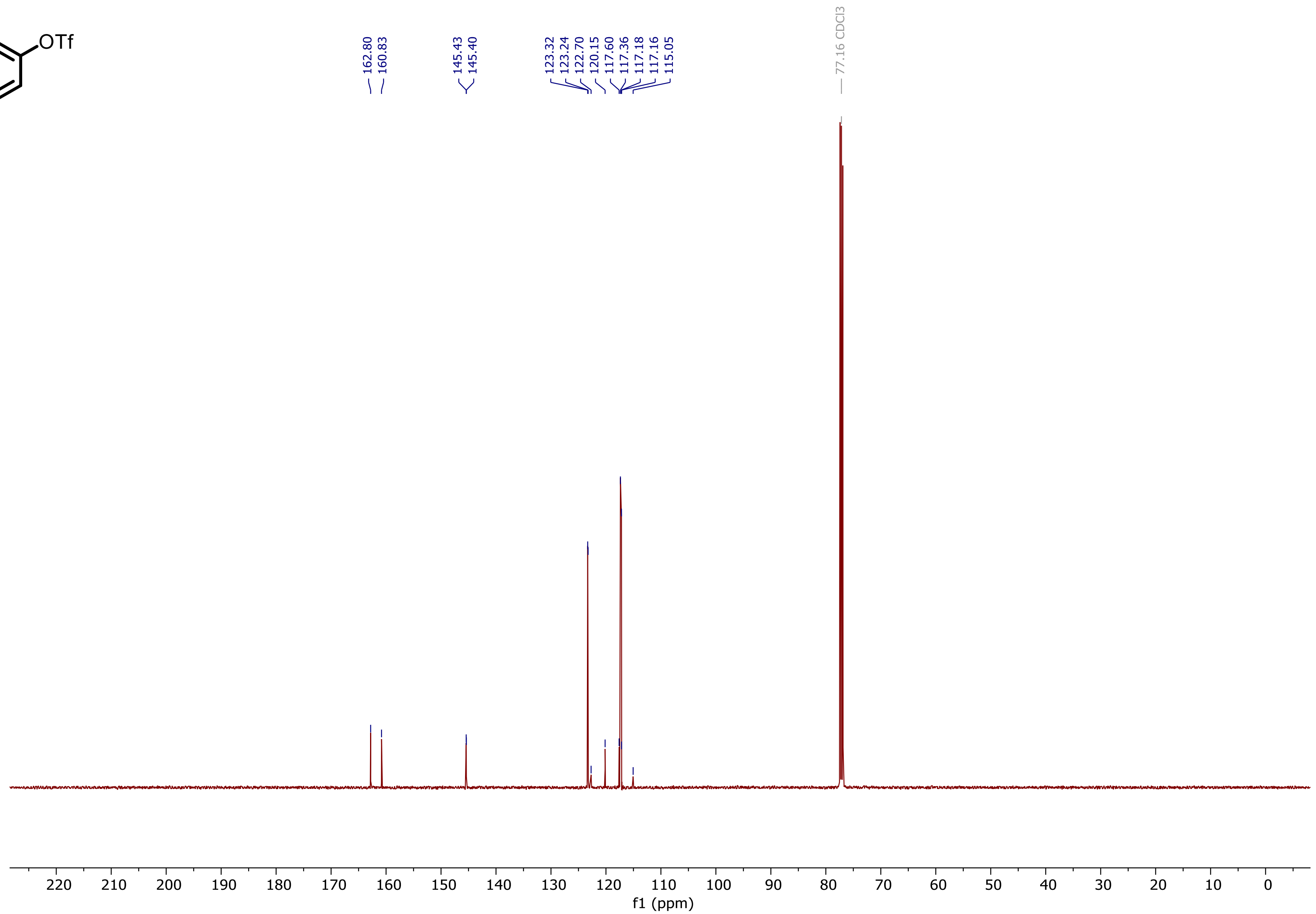

1b - ^{19}F NMR (376 MHz, $CDCl_3$)

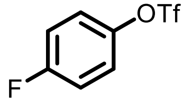

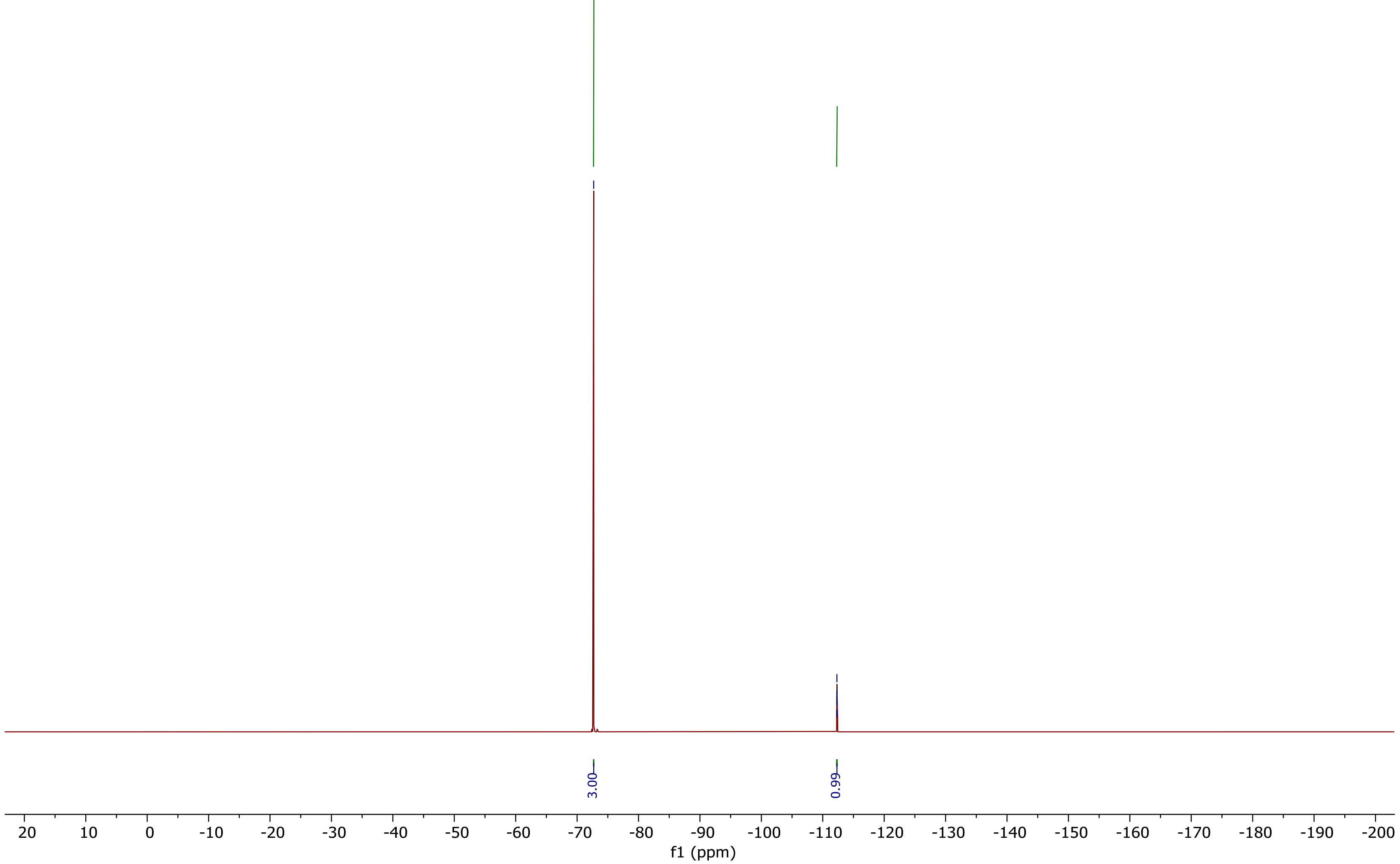

1c - ^{1}H NMR (400 MHz, $CDCl_3$)

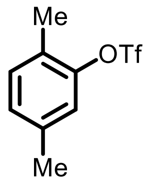

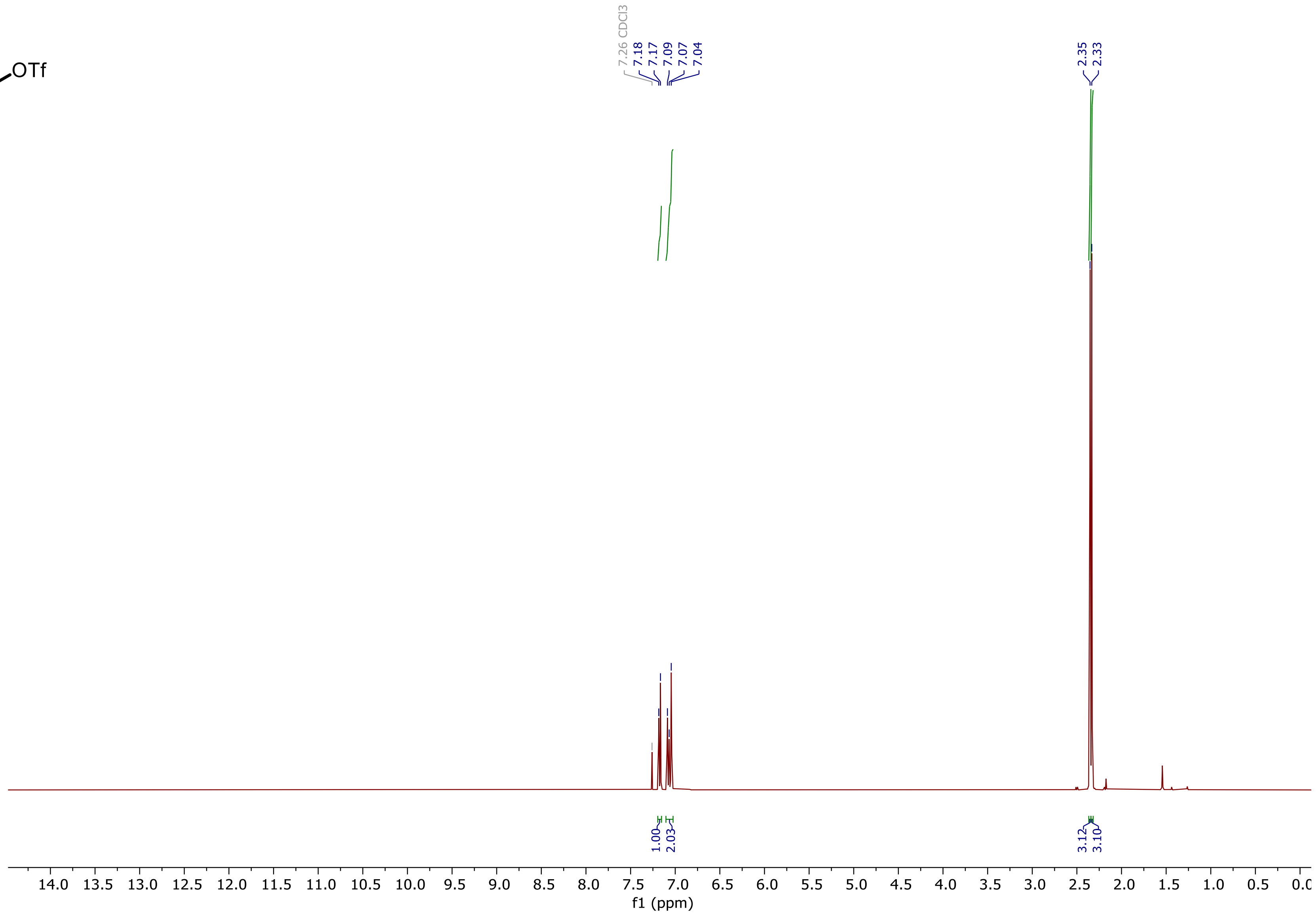

1c - $^{13}C\{^1H\}$ NMR (101 MHz, $CDCl_3$)

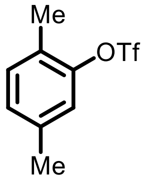

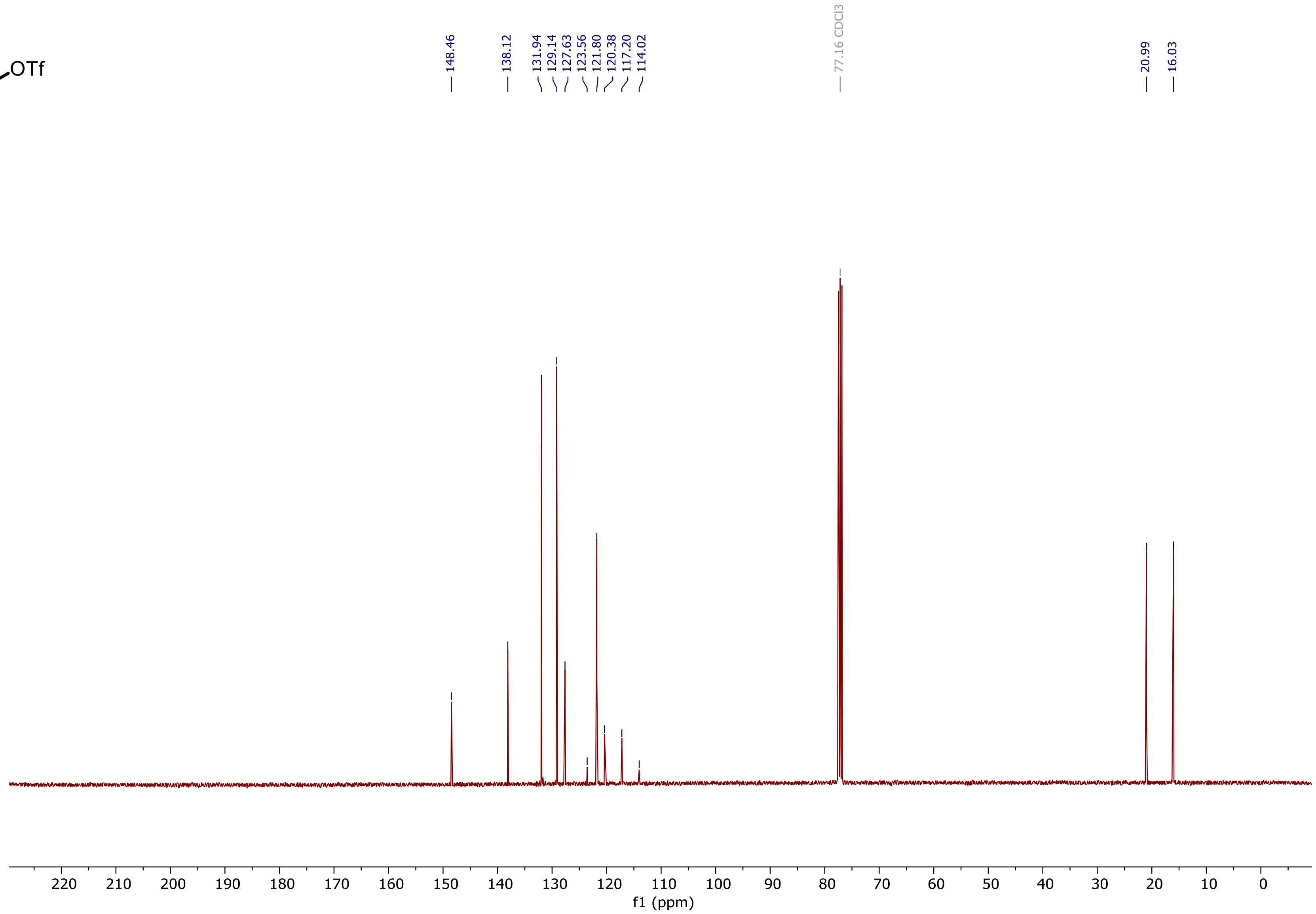

1c - ^{19}F NMR (376 MHz, $CDCl_3$)

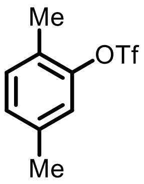

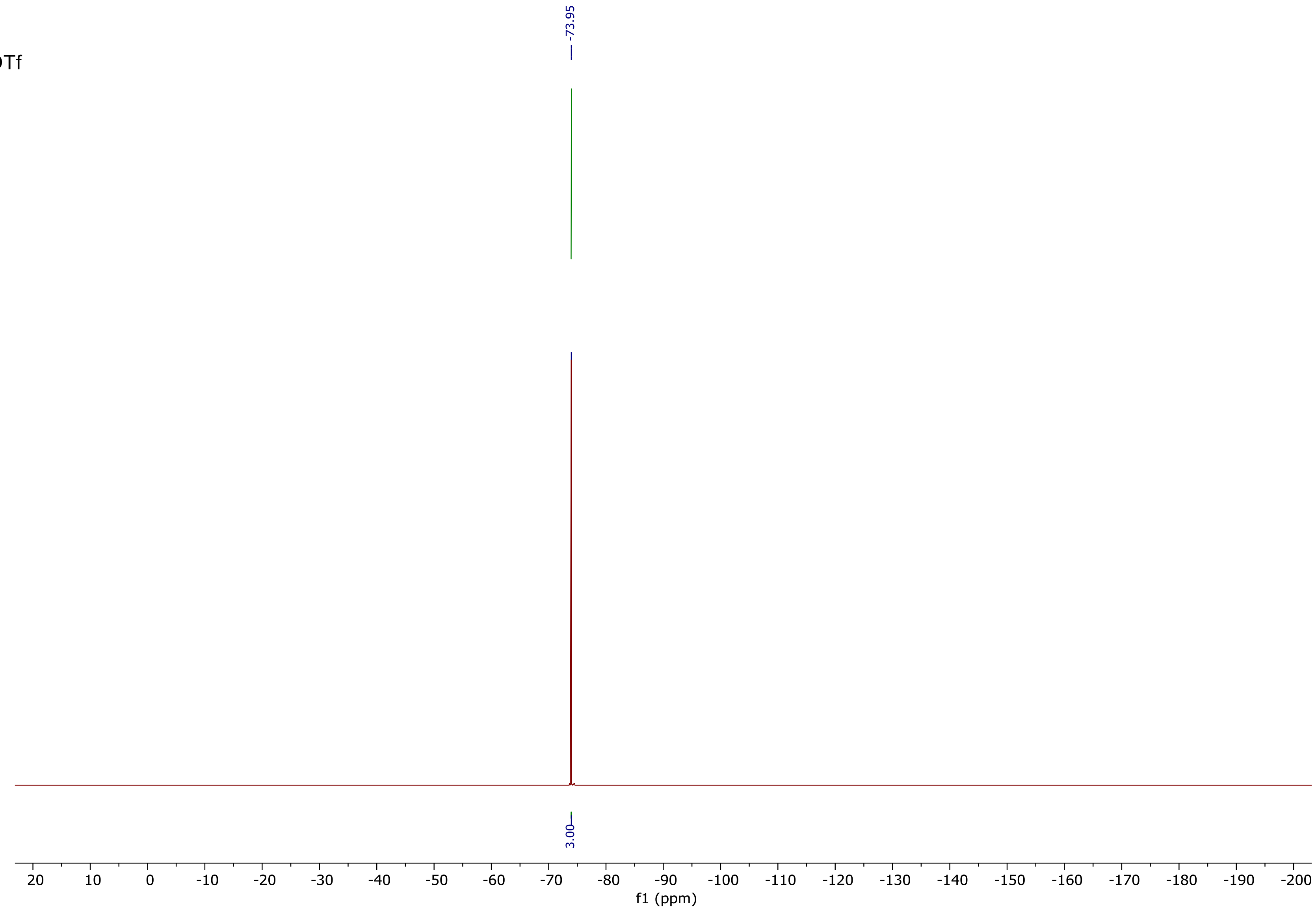

4-Methoxyphenyl trifluoromethanesulfonate - ^{1}H NMR (400 MHz, $CDCl_3$)

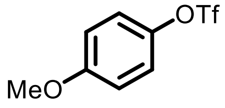

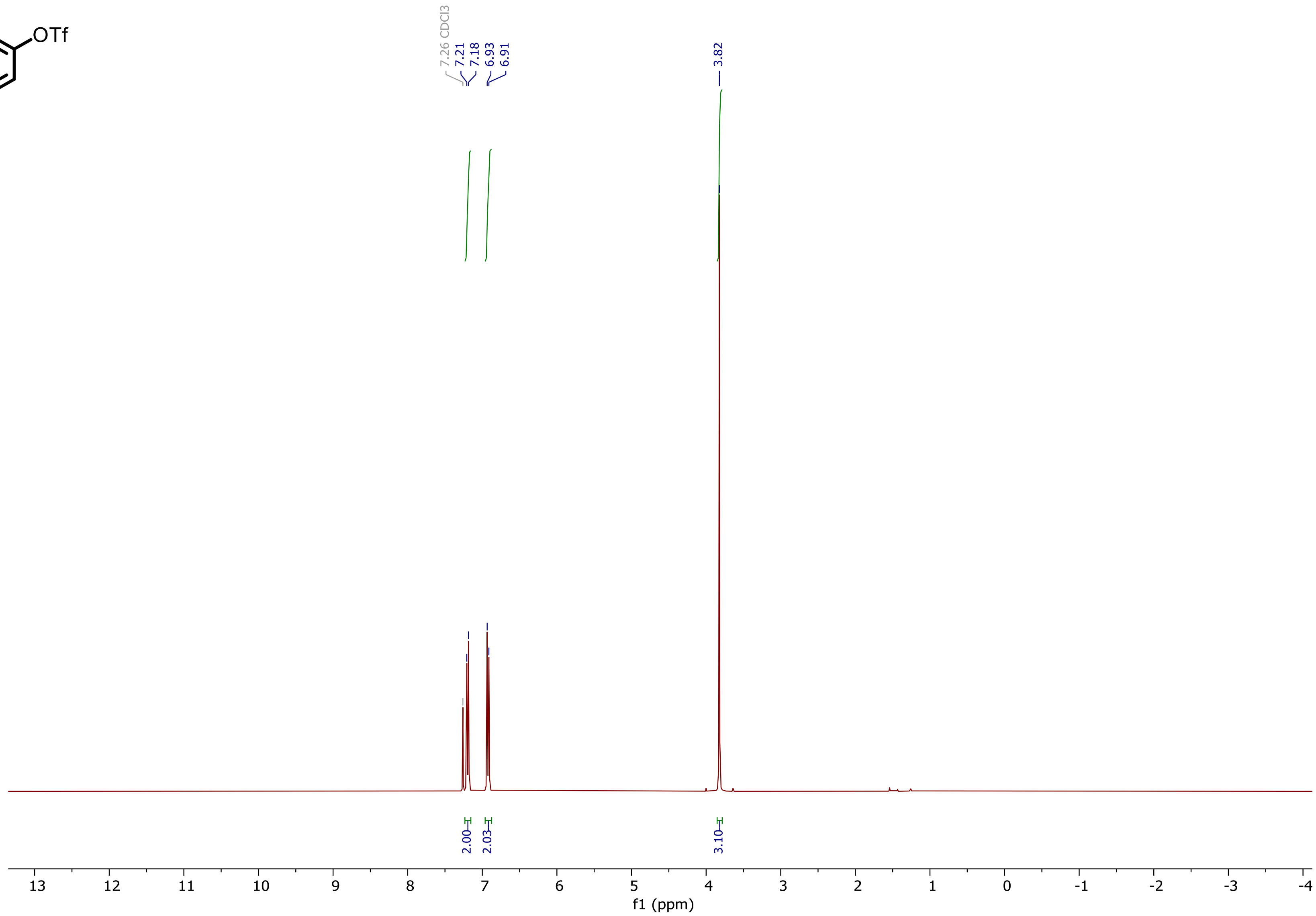

4-Methoxyphenyl trifluoromethanesulfonate - $^{13}C\{^{1}H\}$ NMR (101 MHz, $CDCl_3$)

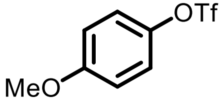

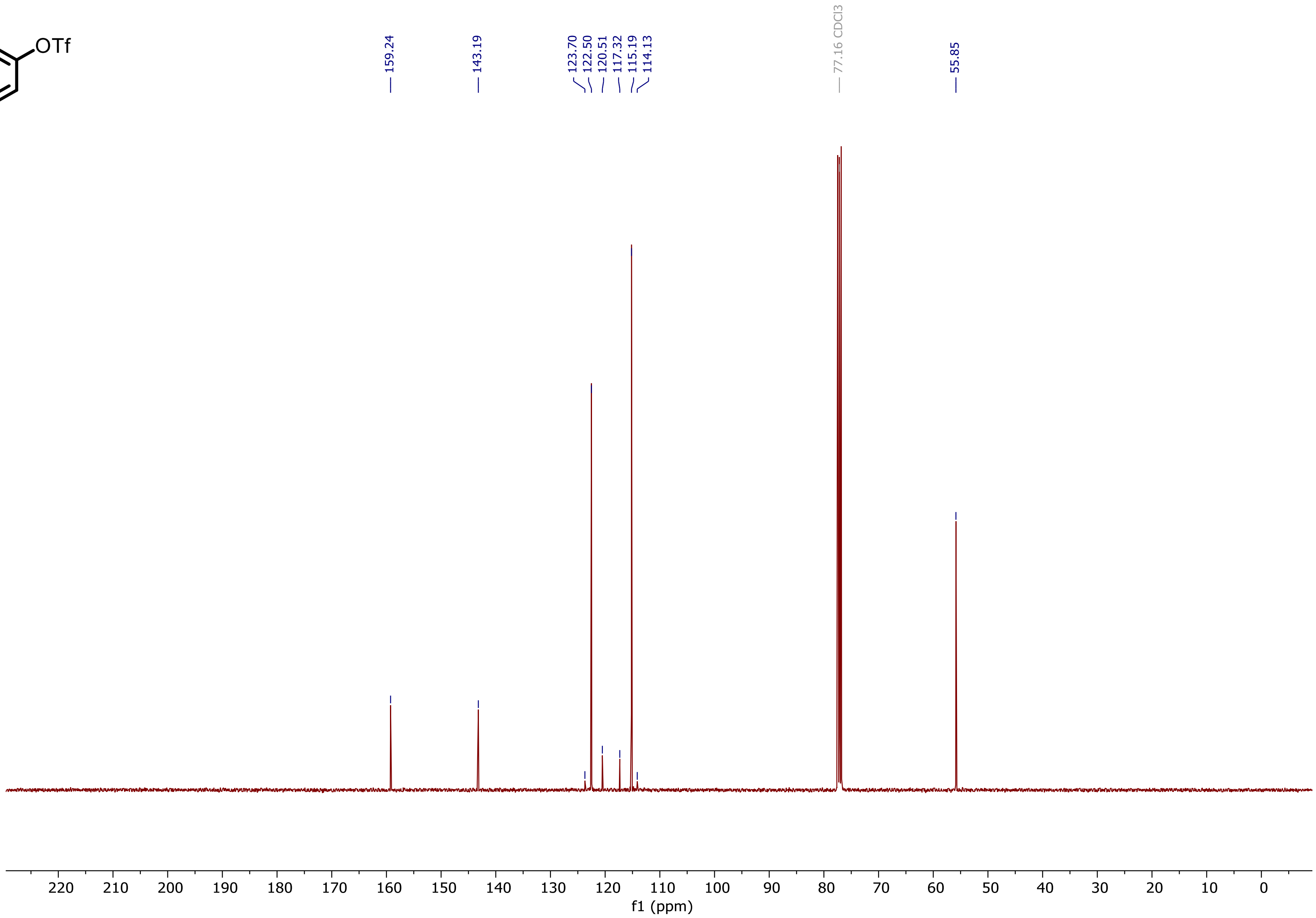

4-Methoxyphenyl trifluoromethanesulfonate - ^{19}F NMR (376 MHz, $CDCl_3$)

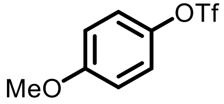

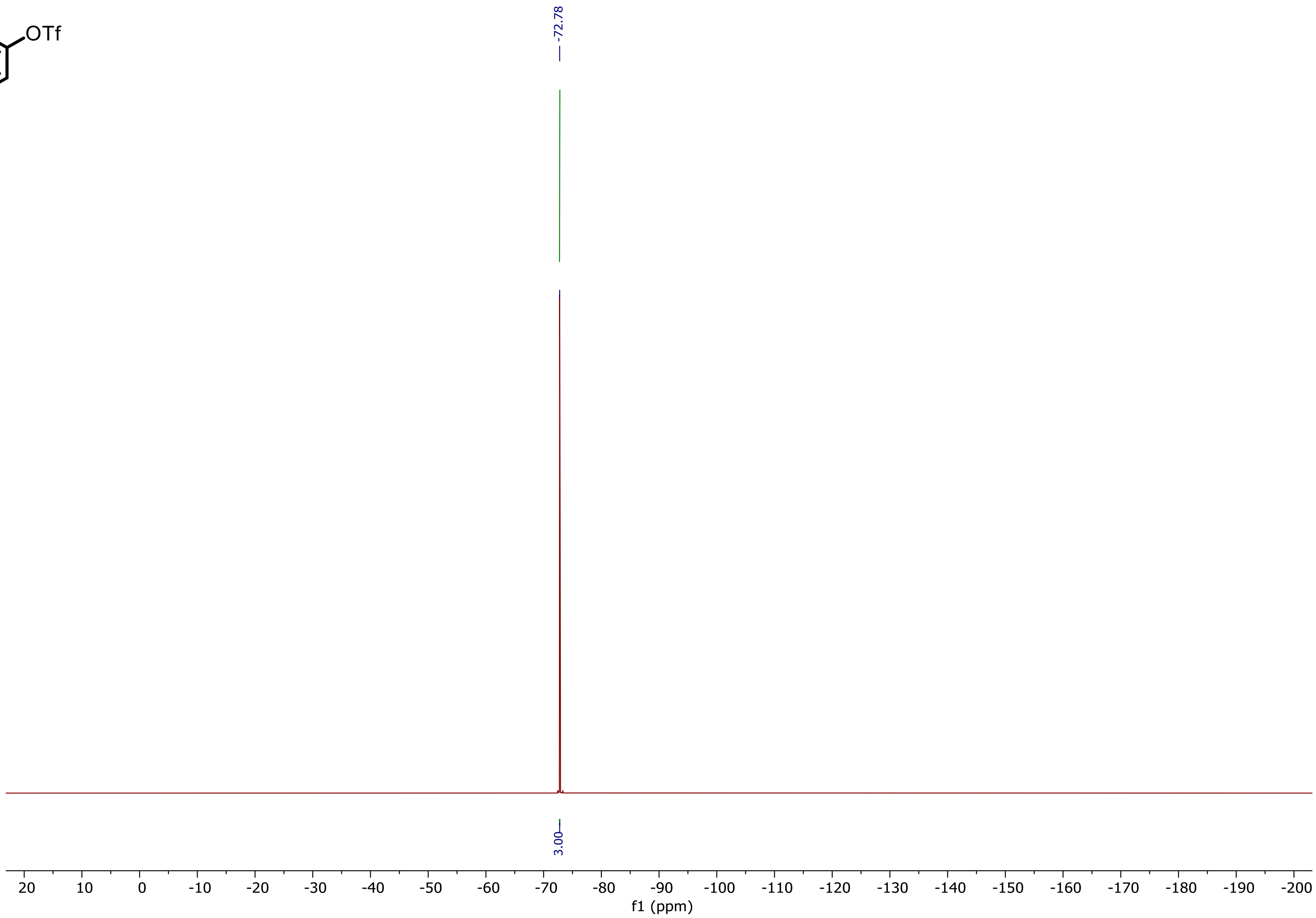

***p*-Tolyl trifluoromethanesulfonate - ^{1}H NMR (400 MHz, $CDCl_3$)**

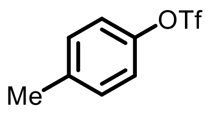

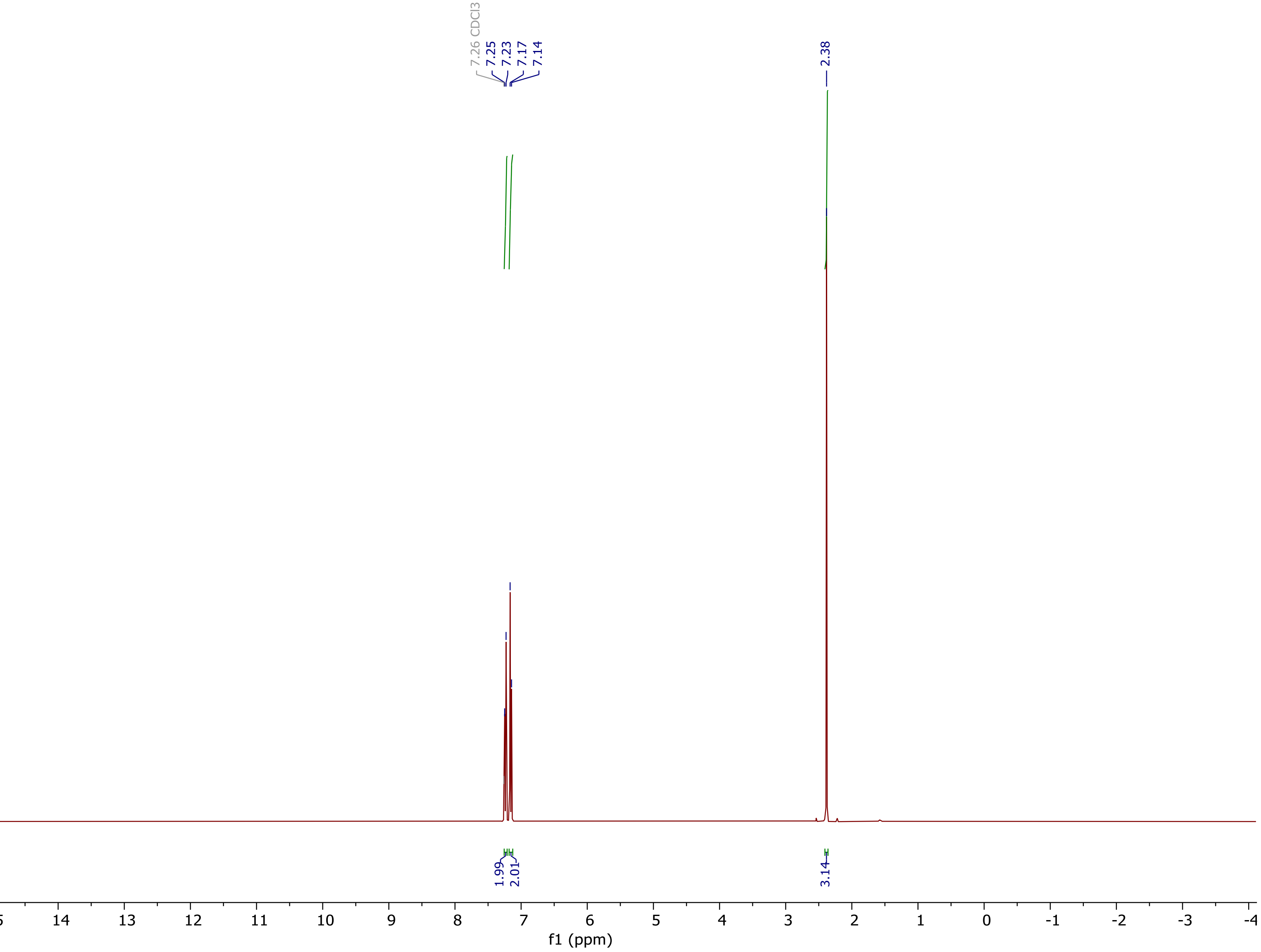

Naphthalen-2-yl trifluoromethanesulfonate - ^{1}H NMR (500 MHz, $CDCl_3$)

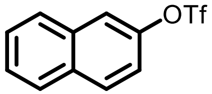

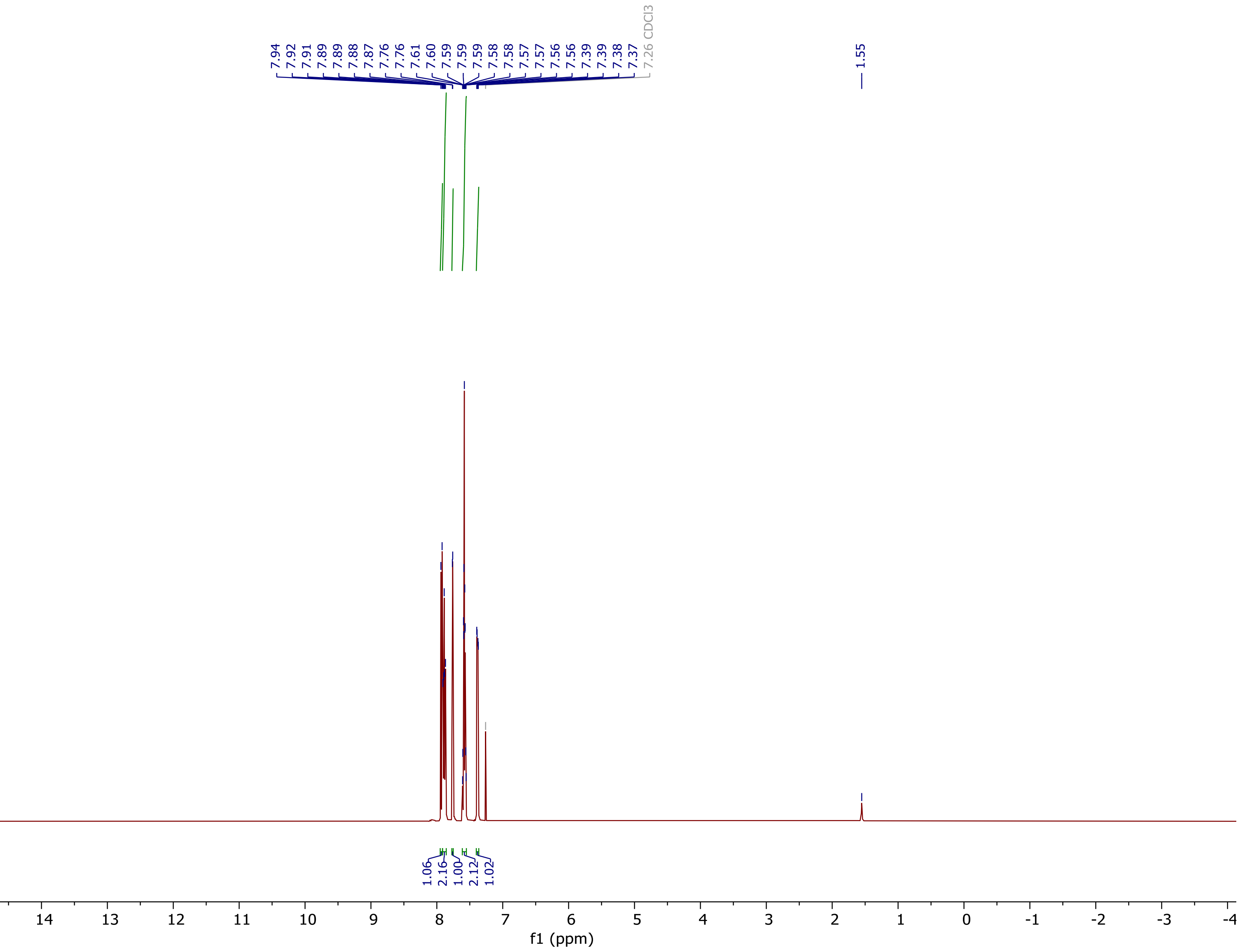

Naphthalen-2-yl trifluoromethanesulfonate - $^{13}C\{^{1}H\}$ NMR (126 MHz, $CDCl_3$)

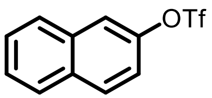

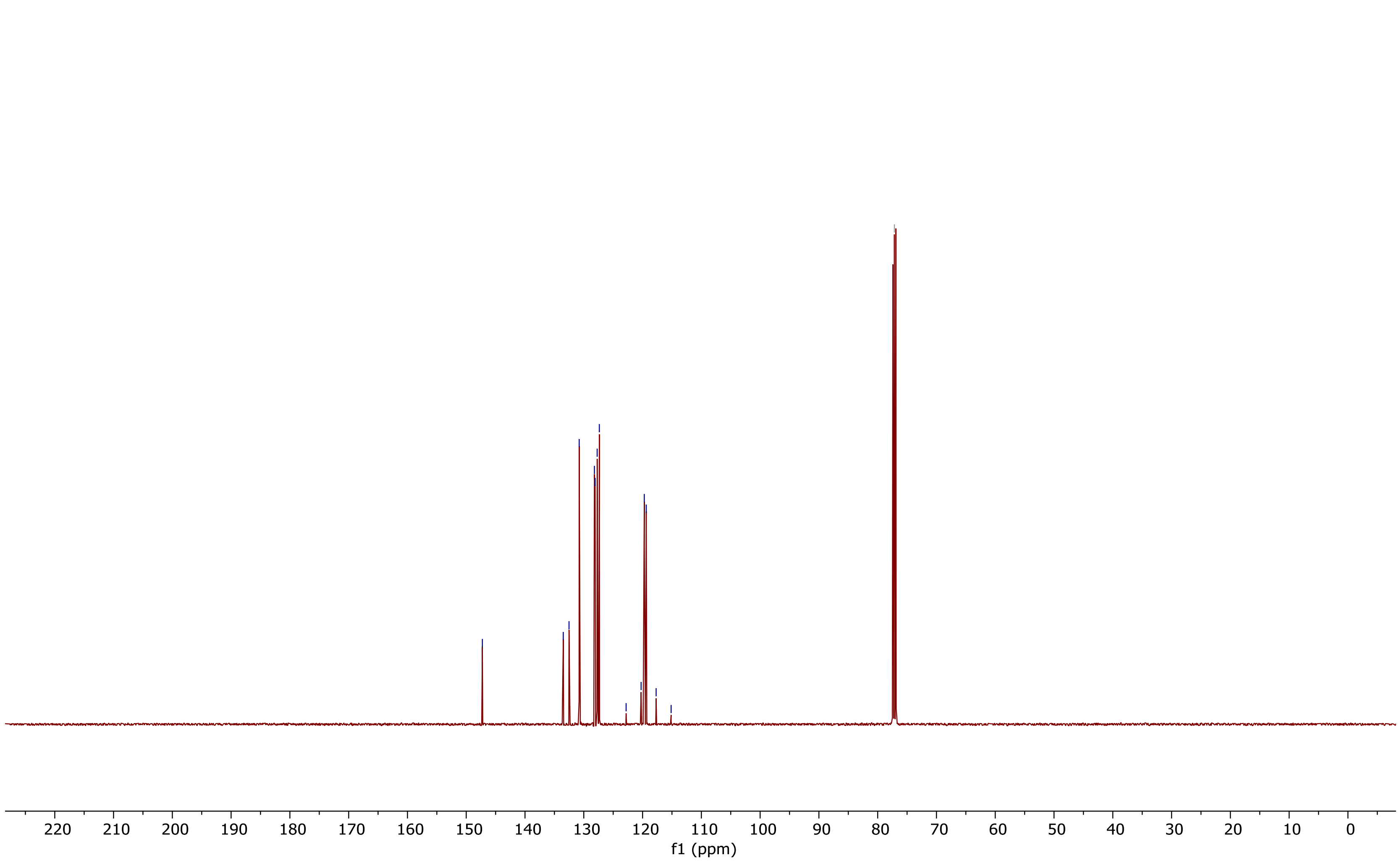

Naphthalen-2-yl trifluoromethanesulfonate - ^{19}F NMR (376 MHz, $CDCl_3$)

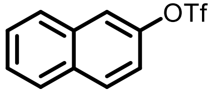

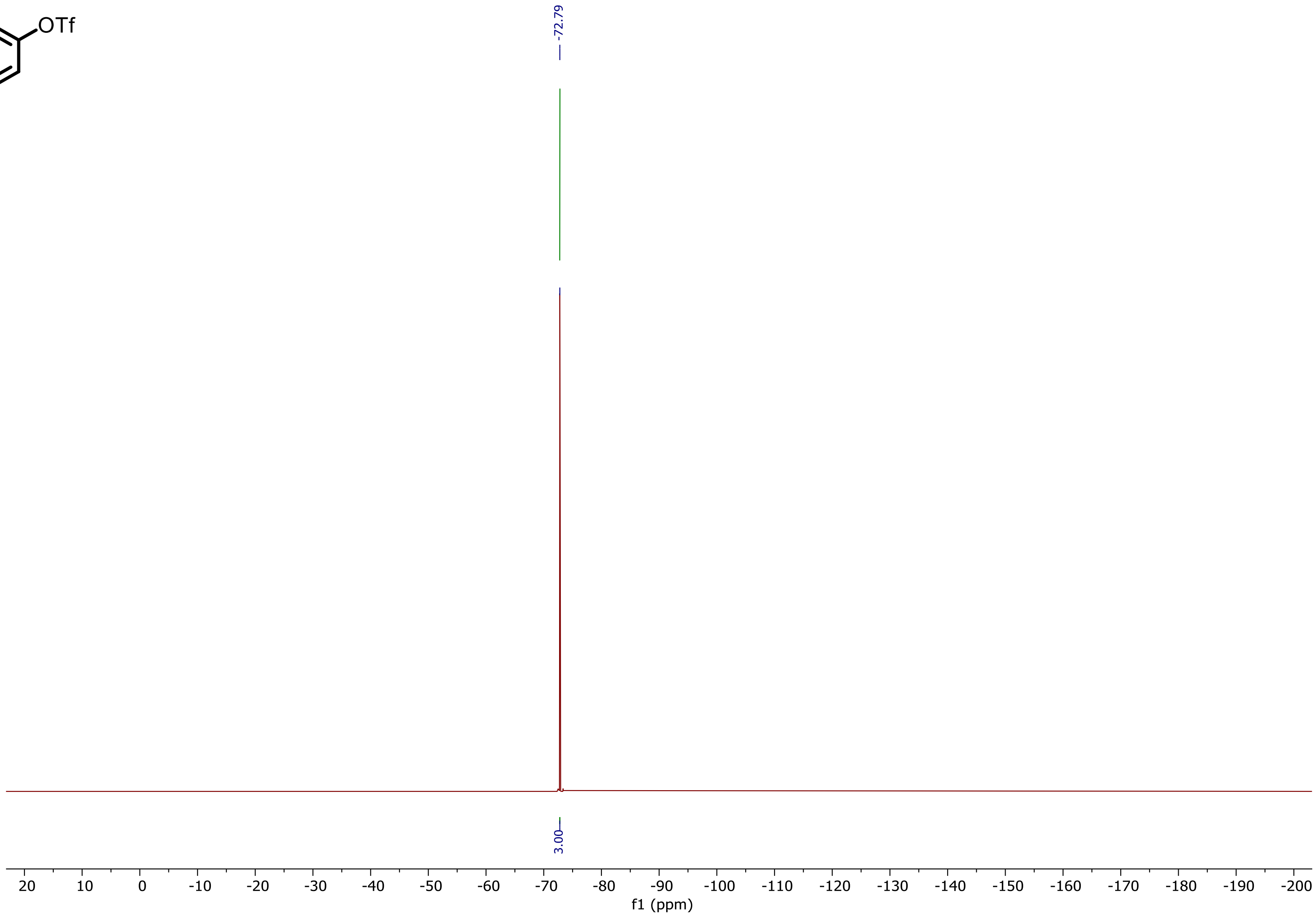

***p*-Tolyl trifluoromethanesulfonate - $^{13}C\{^1H\}$ NMR (101 MHz, $CDCl_3$)**

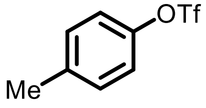

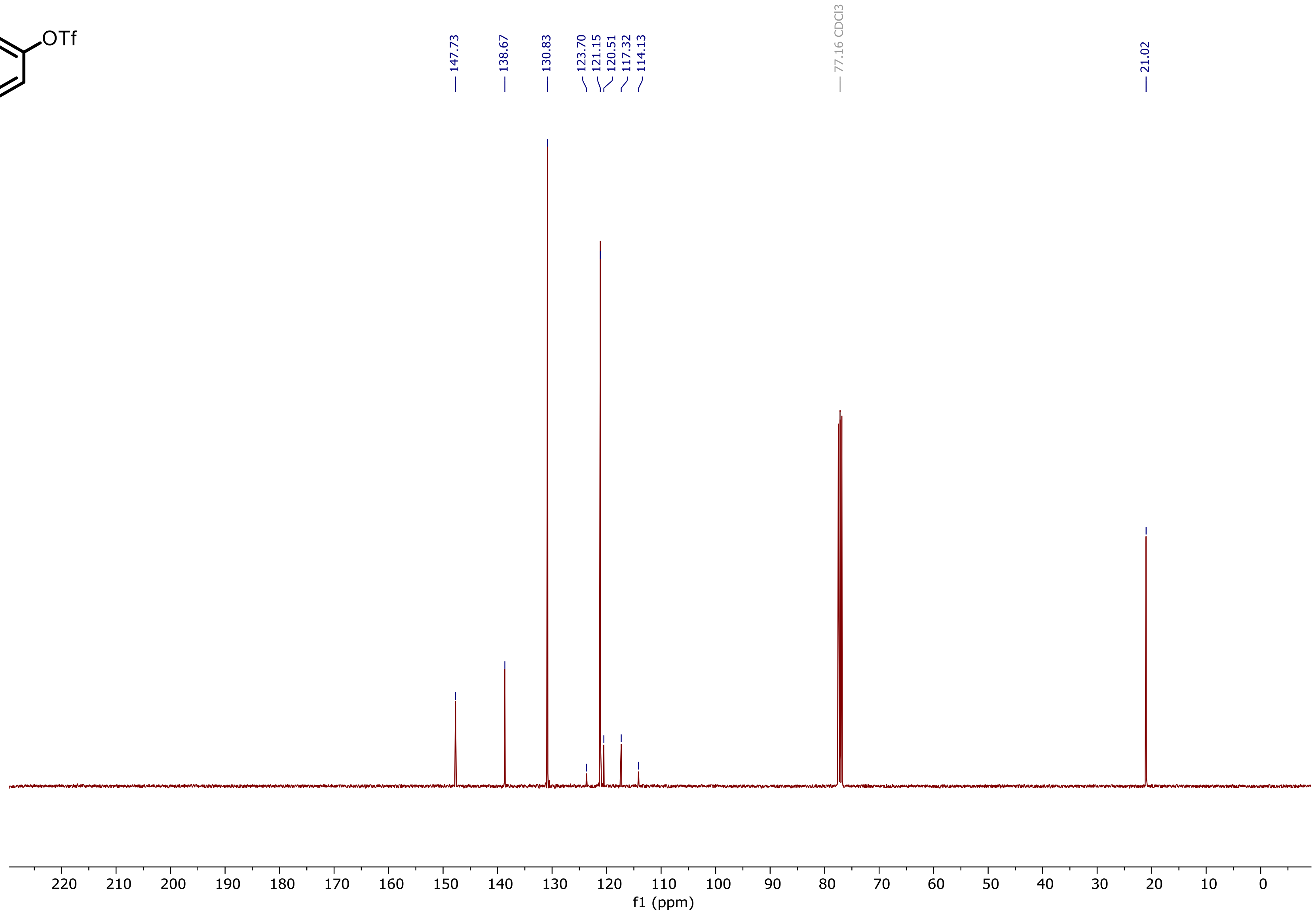

_p_-Tolyl trifluoromethanesulfonate - ^{19}F NMR (376 MHz, $CDCl_3$)

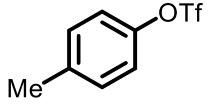

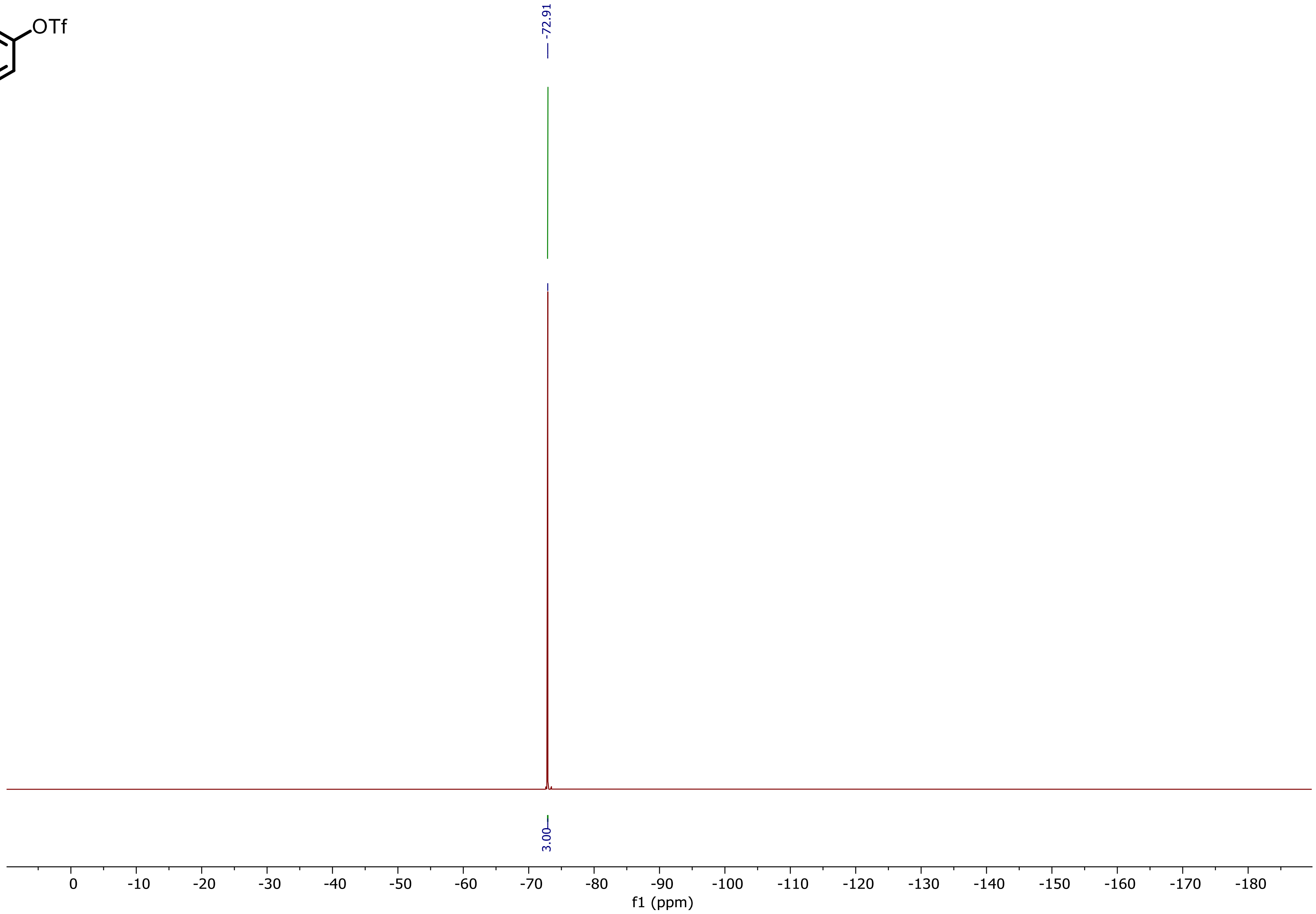

m-Tolyl trifluoromethanesulfonate - ^{1}H NMR (500 MHz, $CDCl_3$)

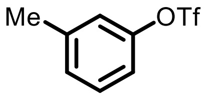

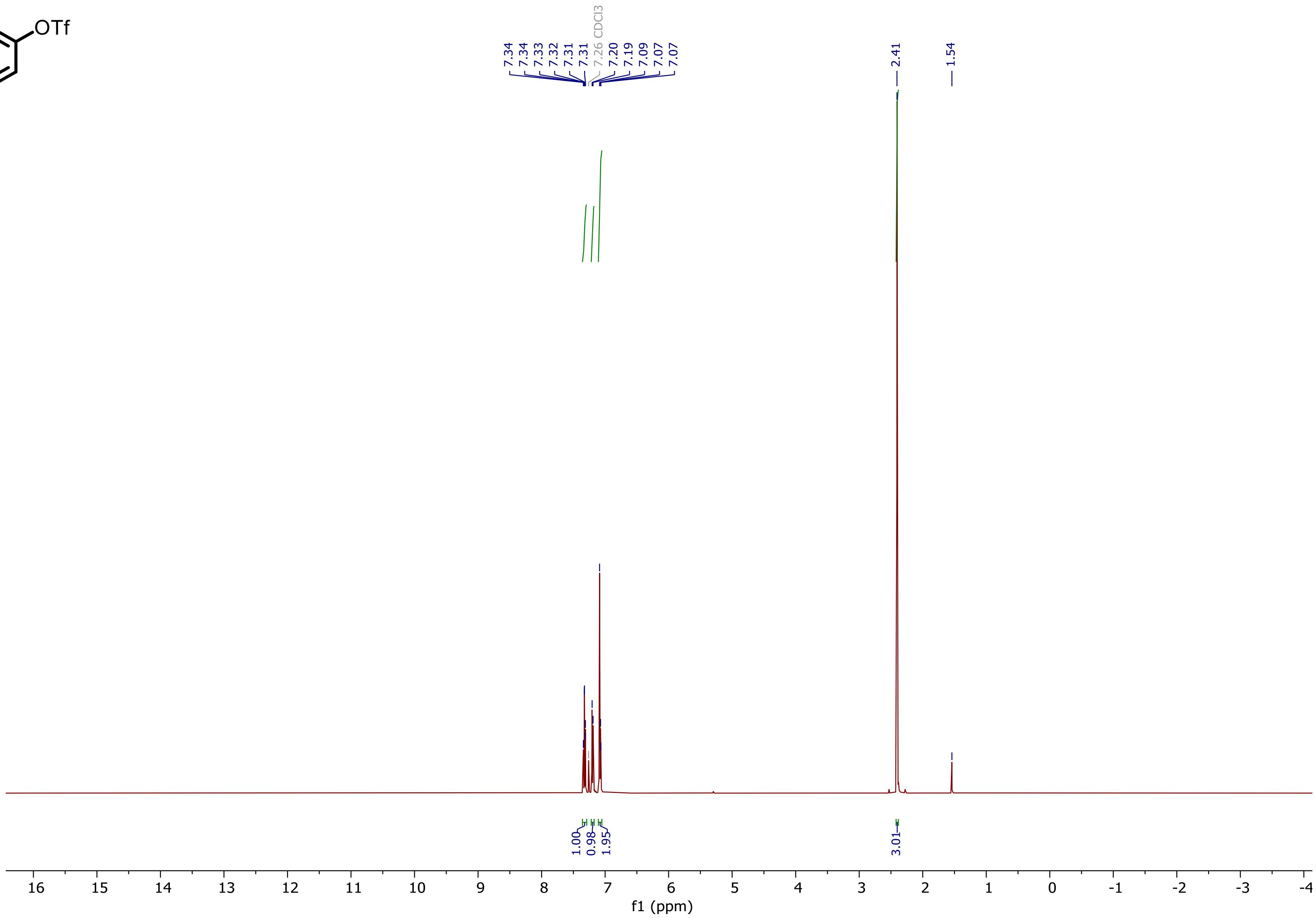

***m*-Tolyl trifluoromethanesulfonate - $^{13}C\{^1H\}$ NMR (126 MHz, $CDCl_3$)**

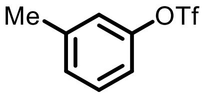

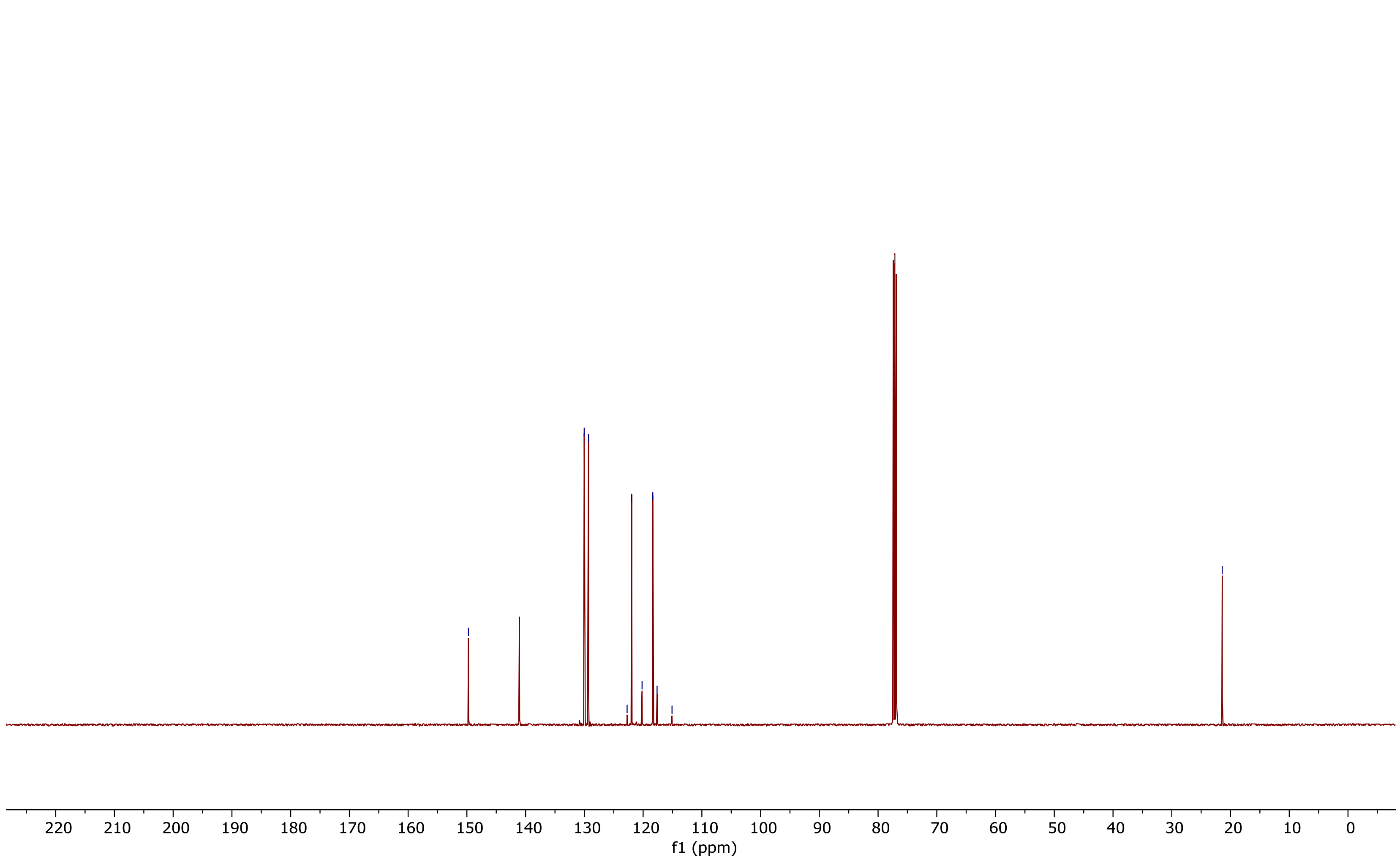

***m*-Tolyl trifluoromethanesulfonate - ^{19}F NMR (376 MHz, $CDCl_3$)**

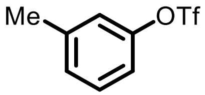

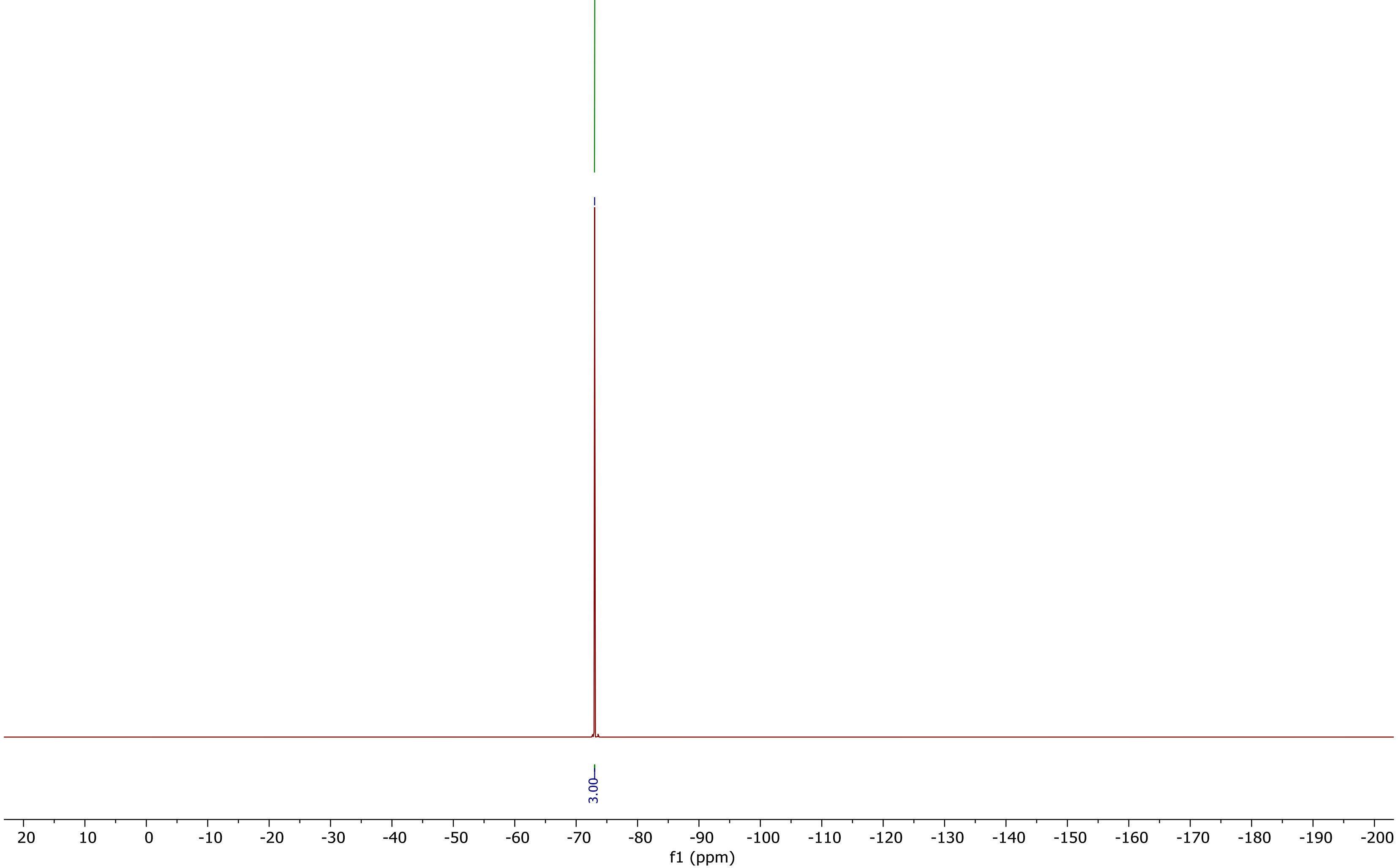

3-Methoxyphenyl trifluoromethanesulfonate - ^{1}H NMR (400 MHz, $CDCl_3$)

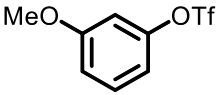

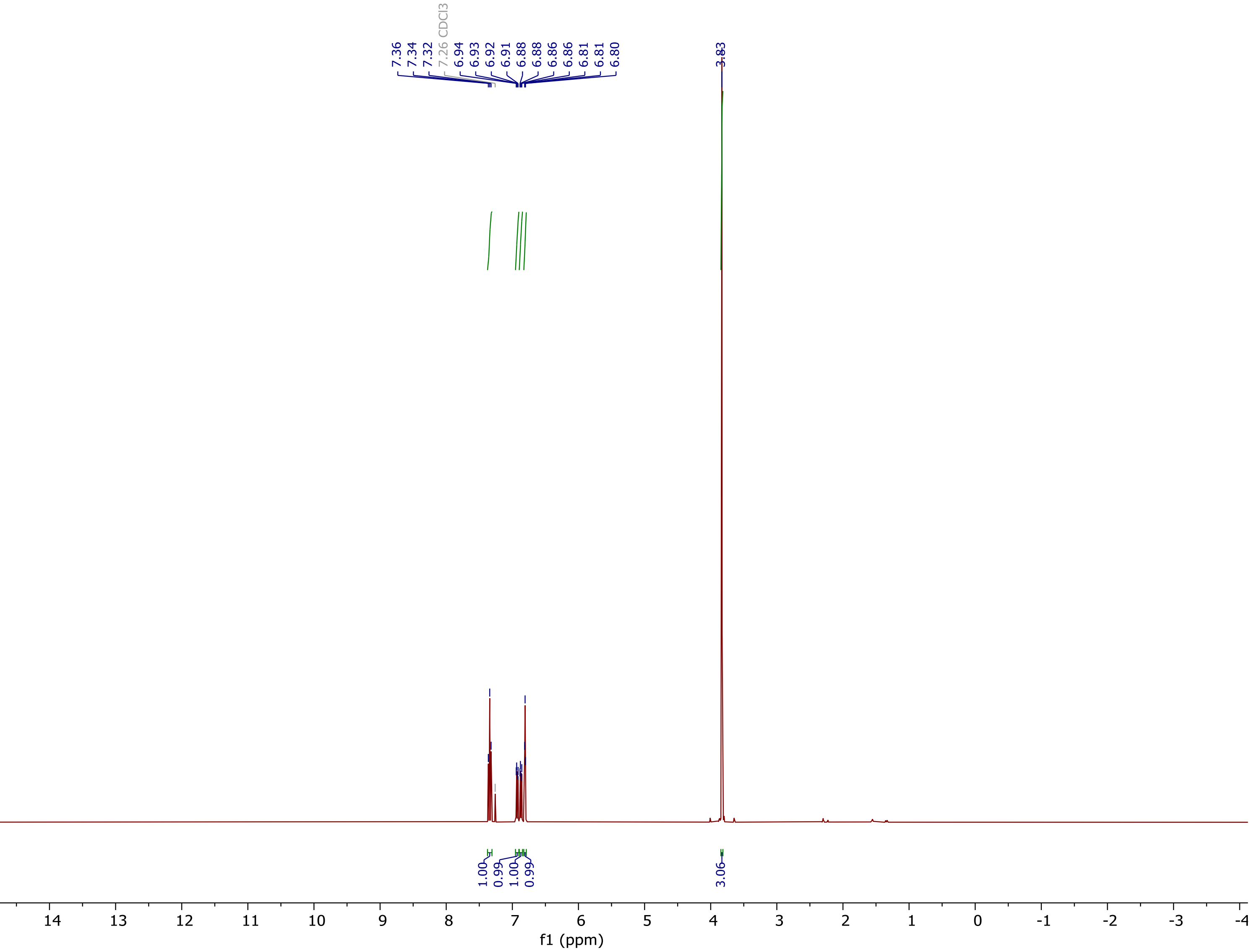

3-Methoxyphenyl trifluoromethanesulfonate - $^{13}C\{^1H\}$ NMR (101 MHz, $CDCl_3$)

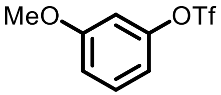

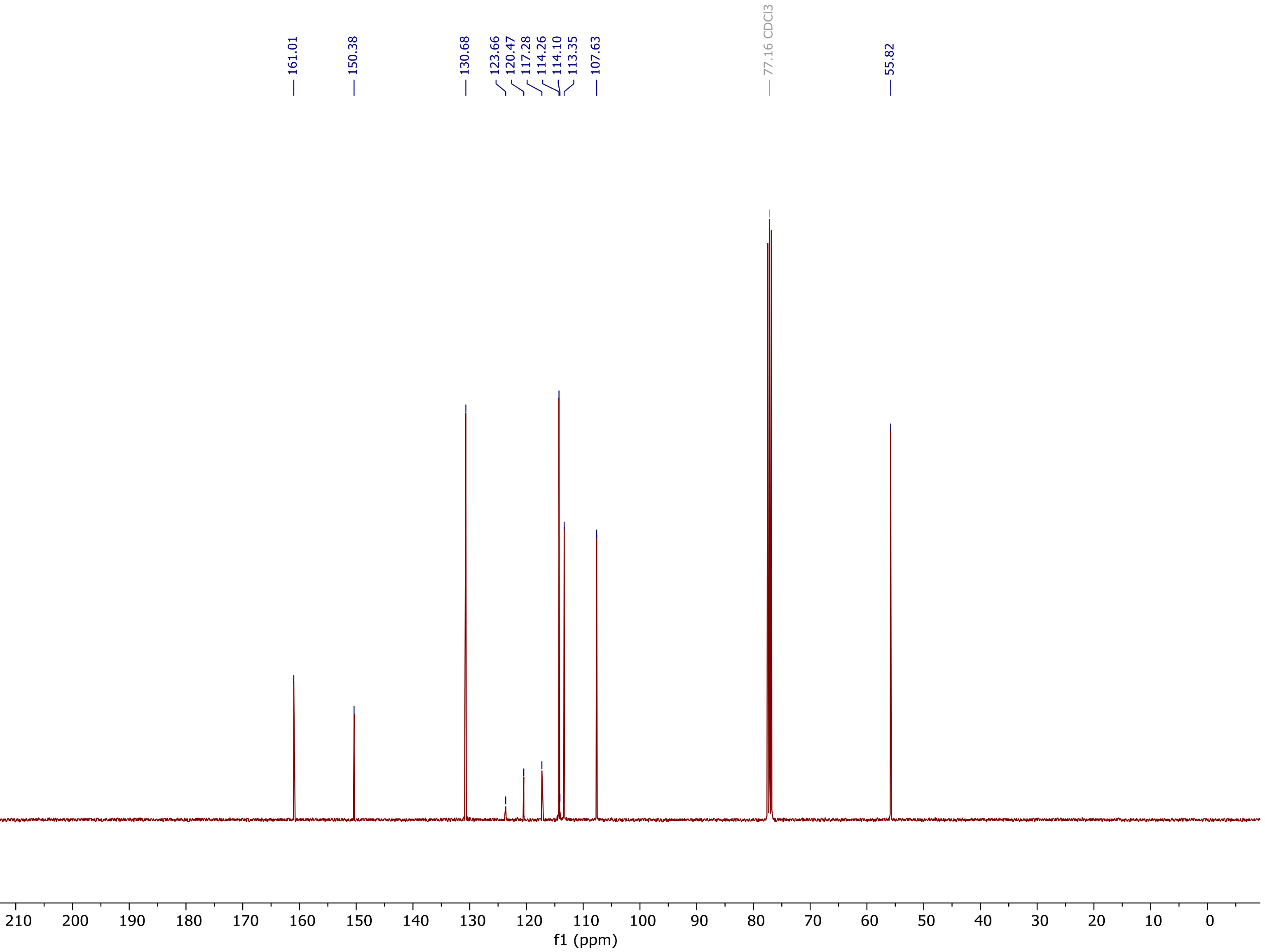

3-Methoxyphenyl trifluoromethanesulfonate - ^{19}F NMR (376 MHz, $CDCl_3$)

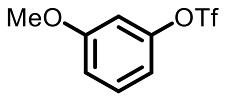

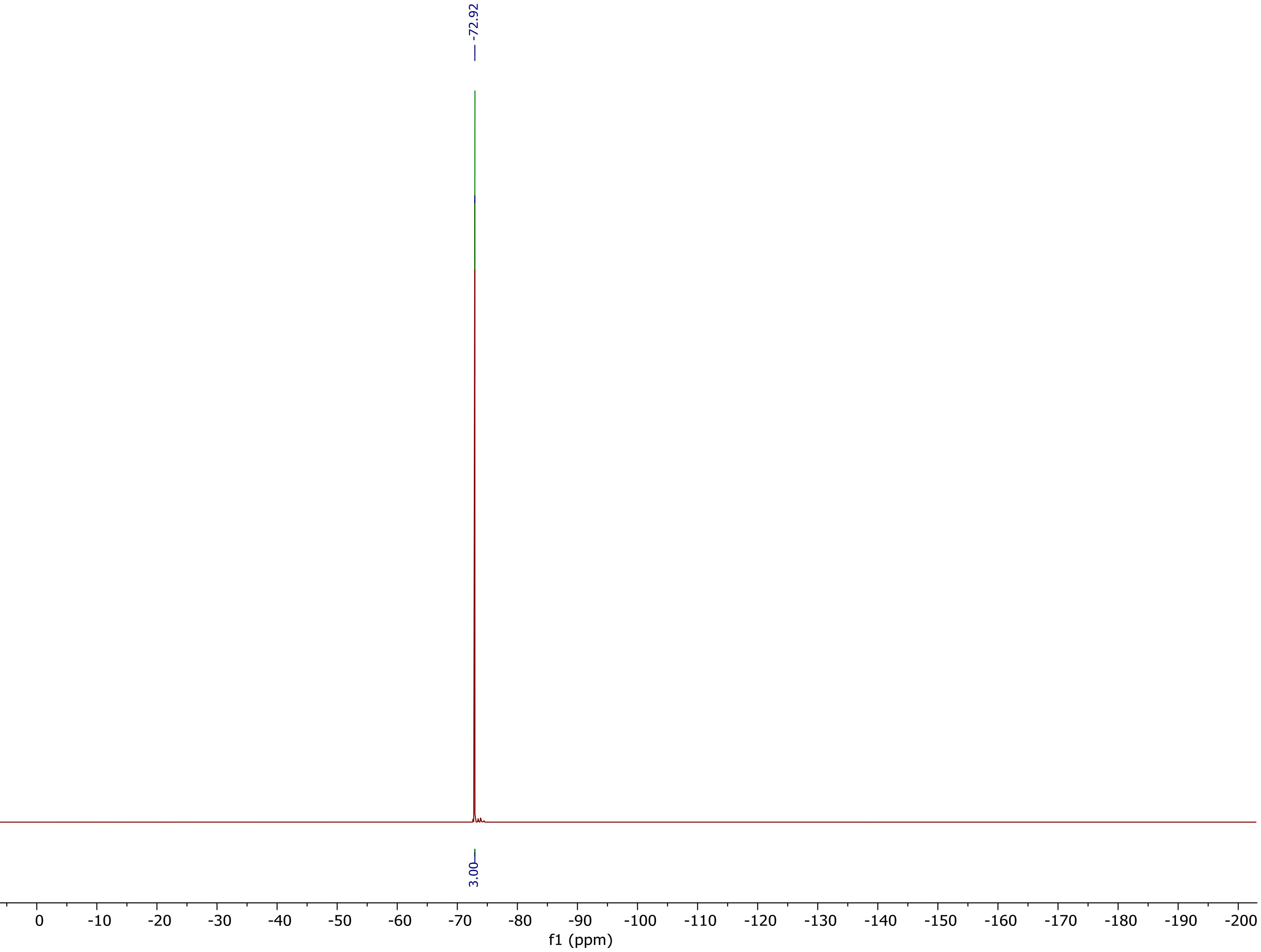

3,5-Dimethoxyphenyl trifluoromethanesulfonate - ^{1}H NMR (400 MHz, $CDCl_3$)

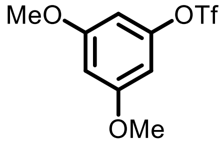

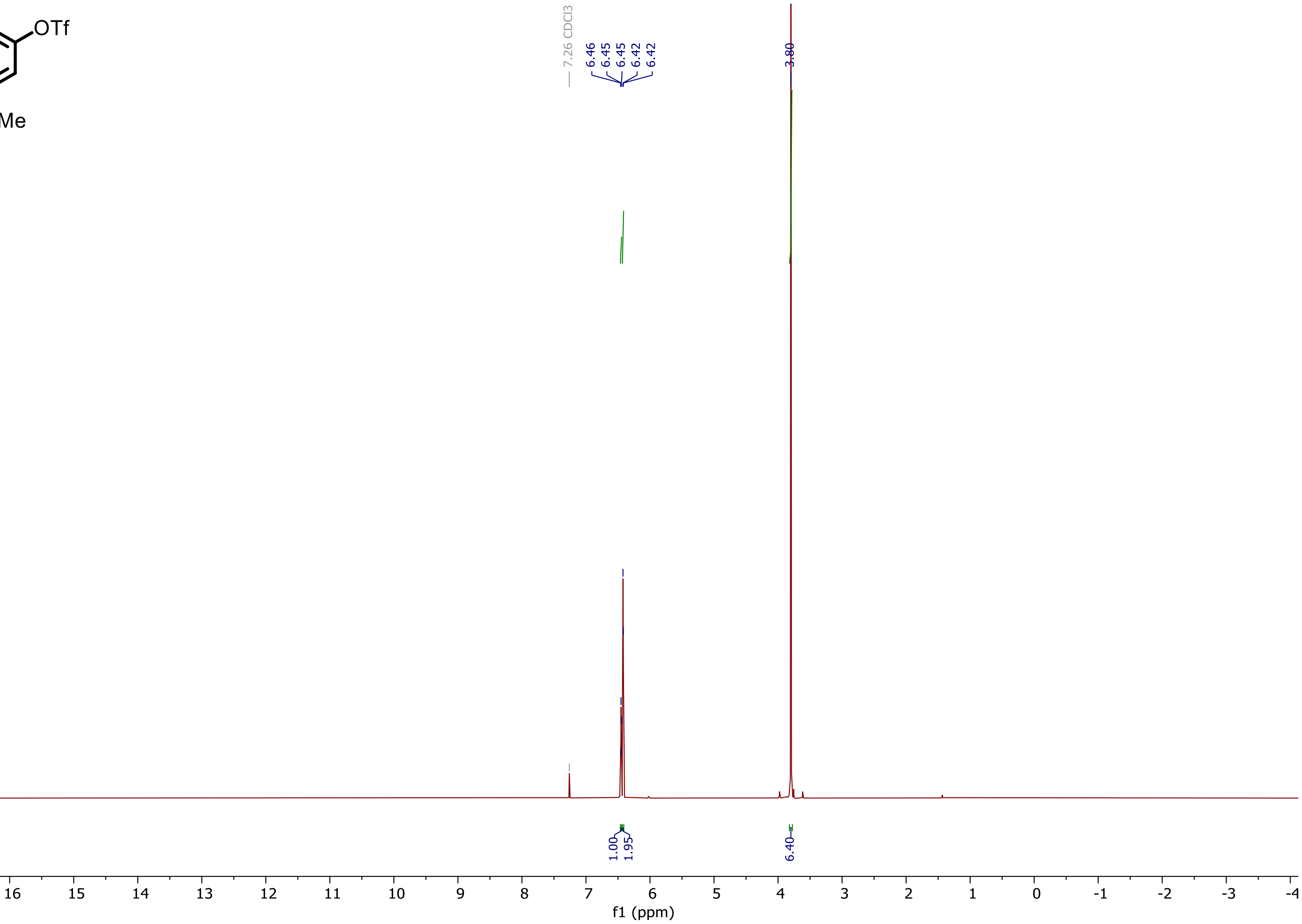

3,5-Dimethoxyphenyl trifluoromethanesulfonate - $^{13}C\{^{1}H\}$ NMR (101 MHz, $CDCl_3$)

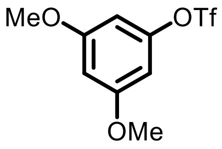

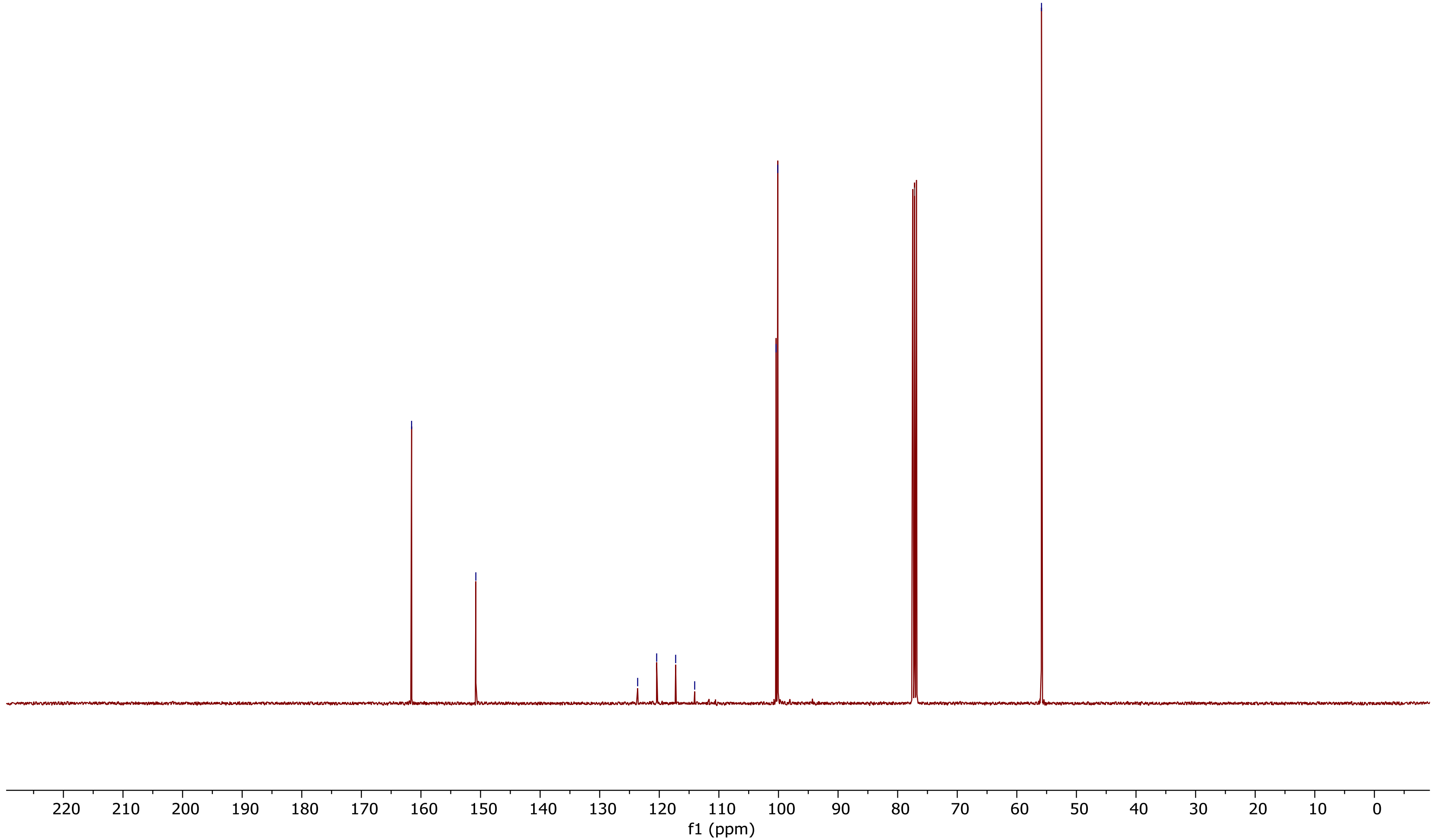

3,5-Dimethoxyphenyl trifluoromethanesulfonate - ^{19}F NMR (376 MHz, $CDCl_3$)

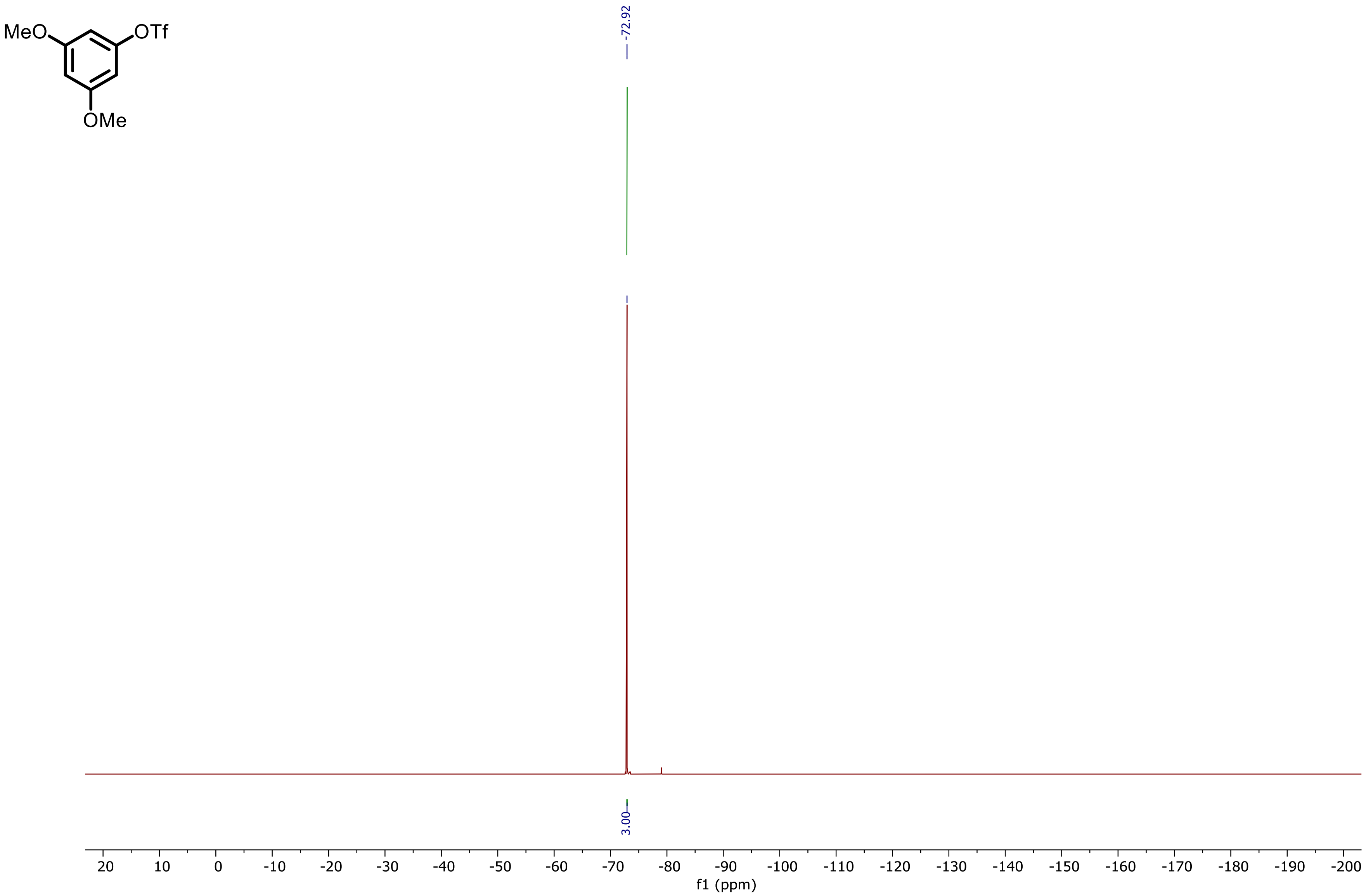

3-Fluorophenyl trifluoromethanesulfonate - ^{1}H NMR (400 MHz, $CDCl_3$)

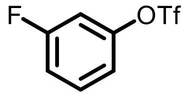

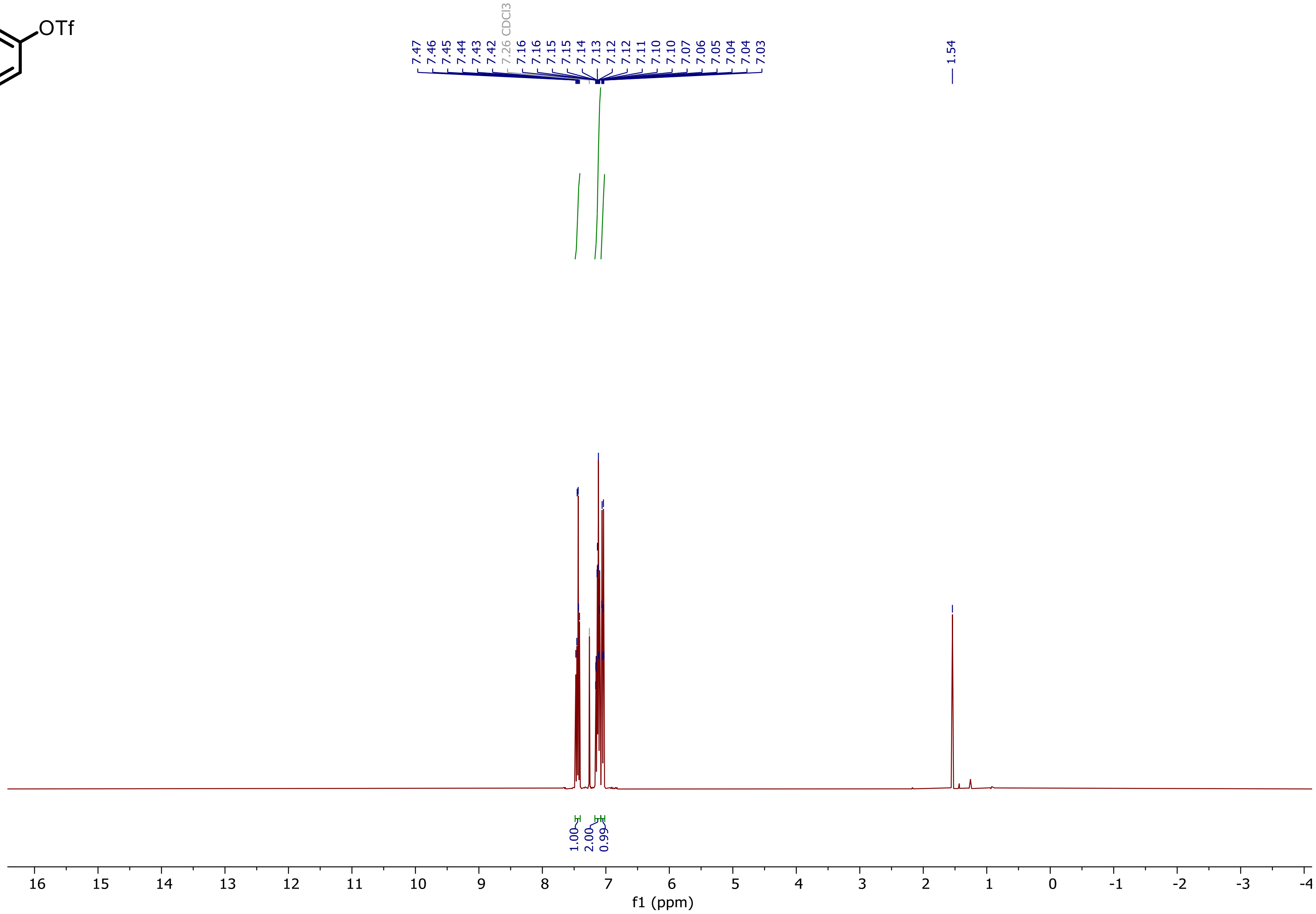

3-Fluorophenyl trifluoromethanesulfonate - $^{13}C\{^{1}H\}$ NMR (101 MHz, $CDCl_3$)

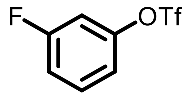

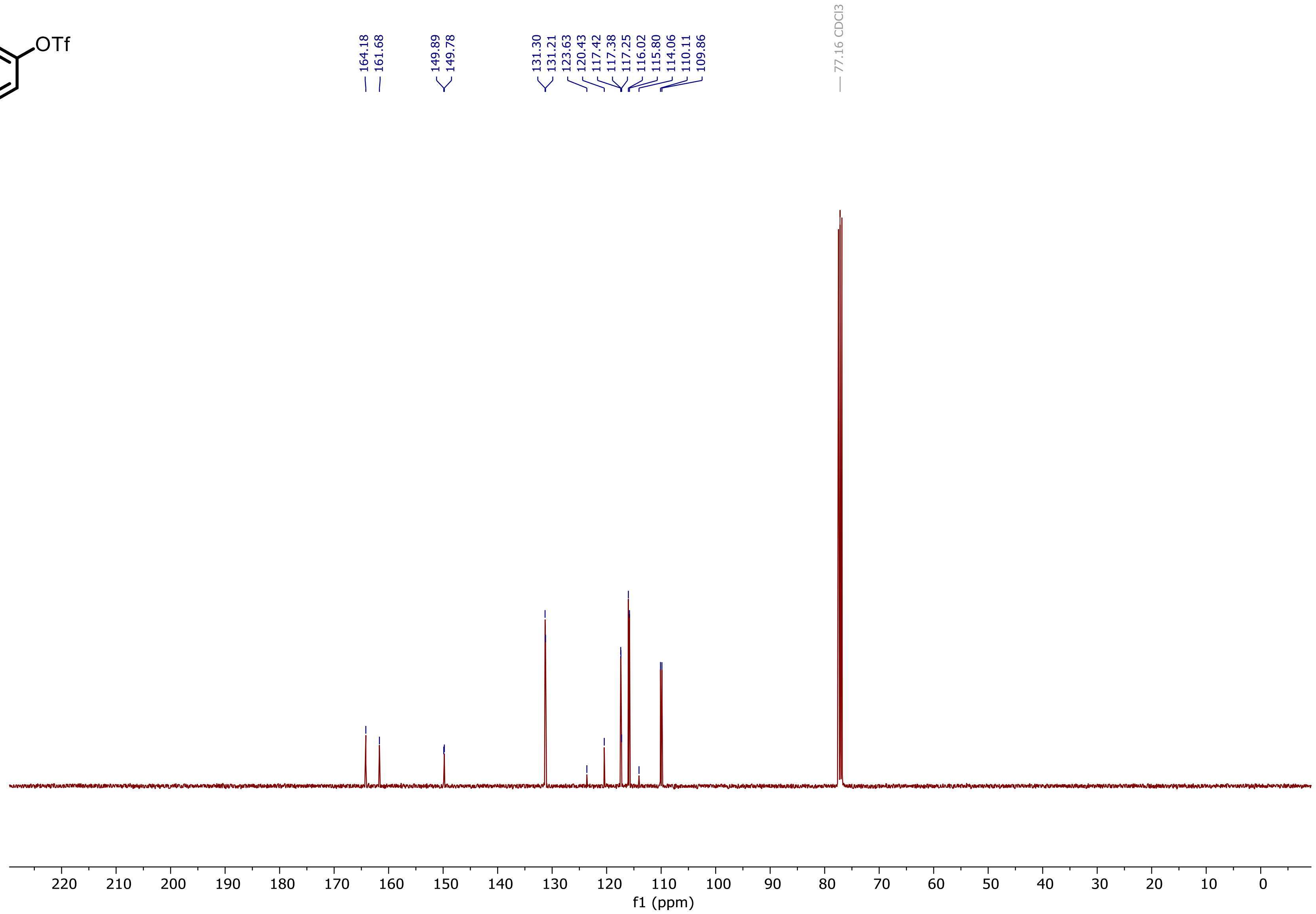

3-Fluorophenyl trifluoromethanesulfonate - ^{19}F NMR (376 MHz, $CDCl_3$)

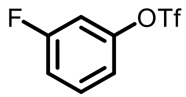

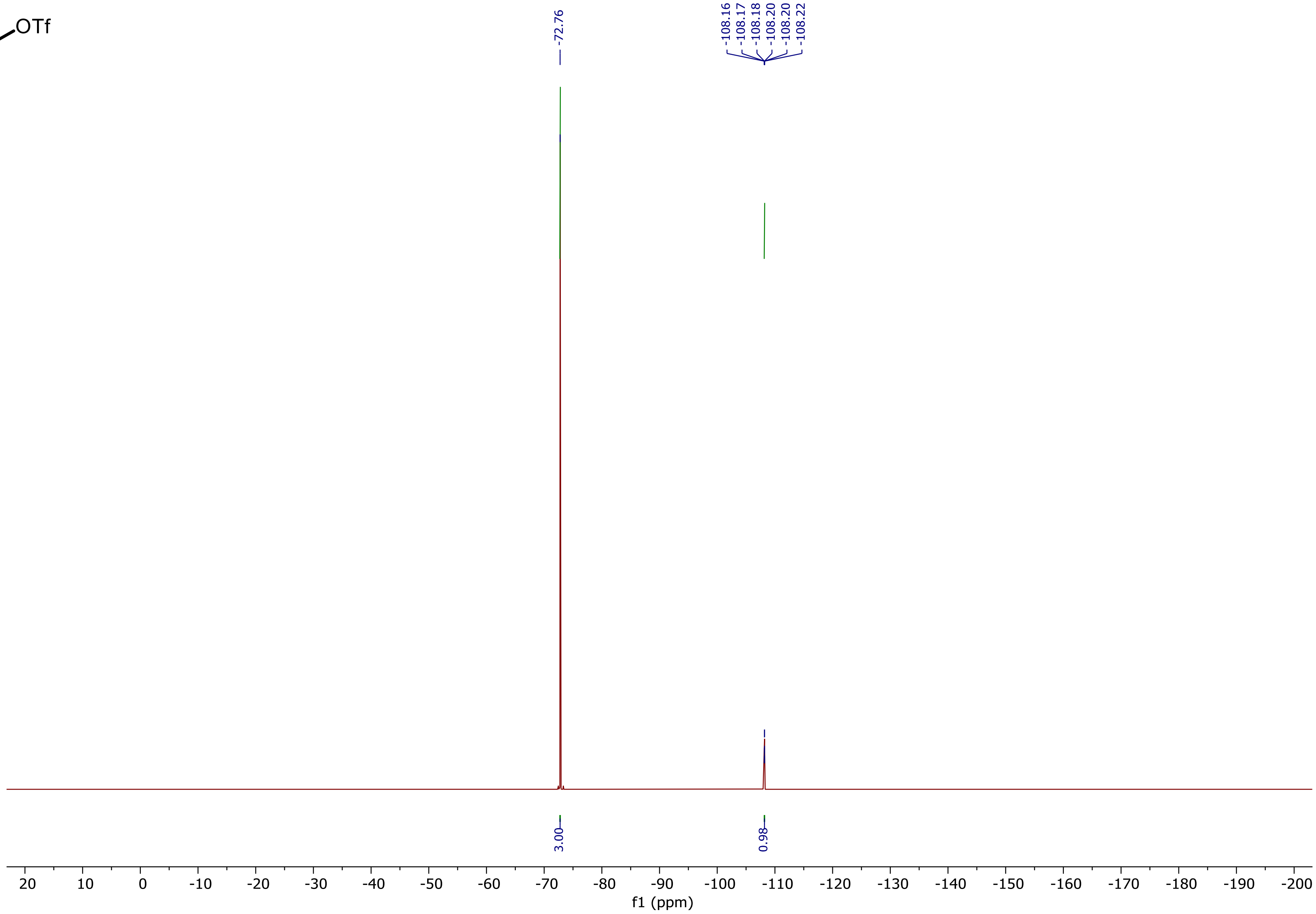

3-(Trifluoromethyl)phenyl trifluoromethanesulfonate - ^{1}H NMR (500 MHz, $CDCl_3$)

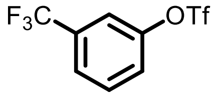

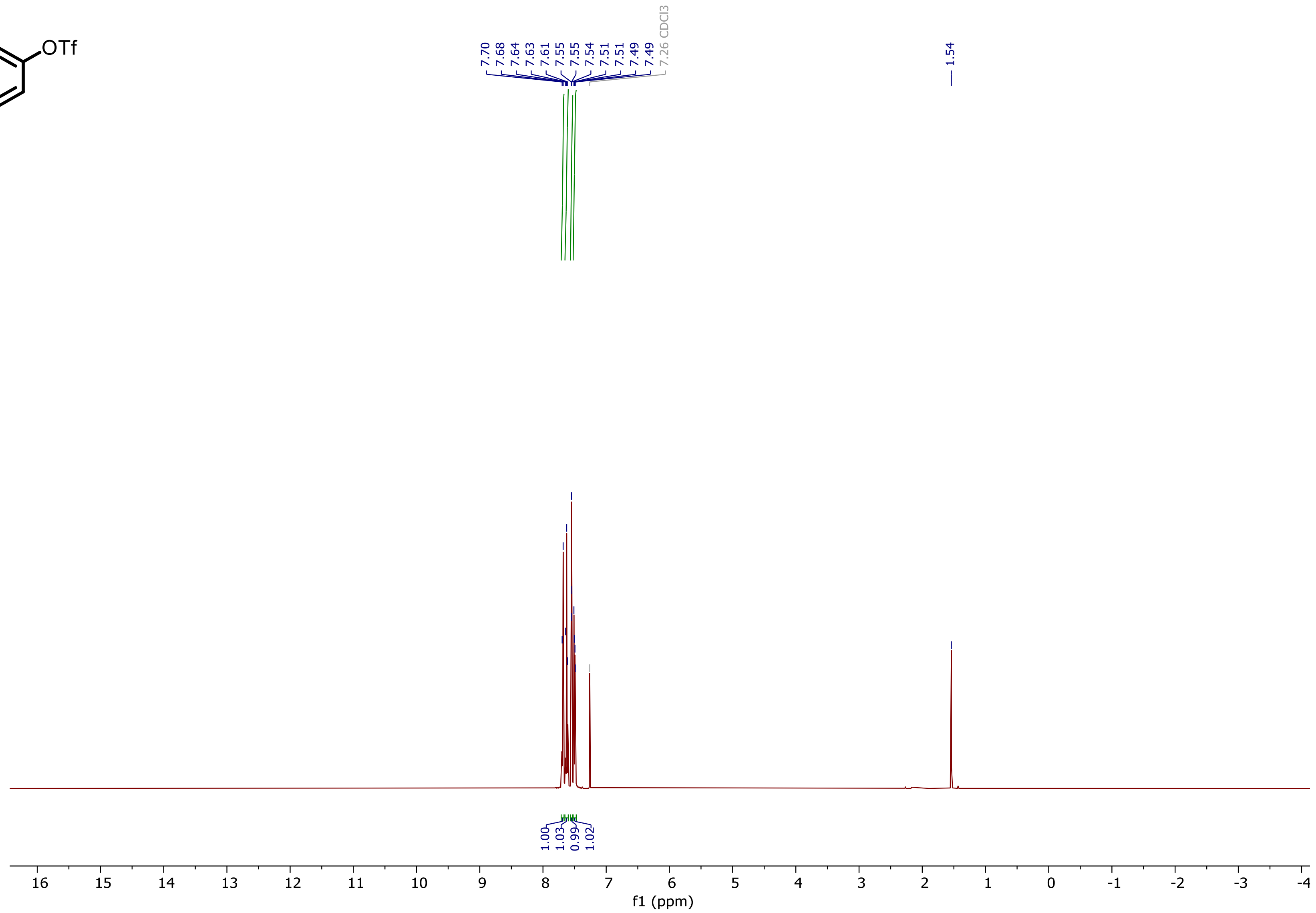

3-(Trifluoromethyl)phenyl trifluoromethanesulfonate - $^{13}C\{^1H\}$ NMR (126 MHz, $CDCl_3$)

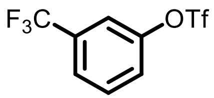

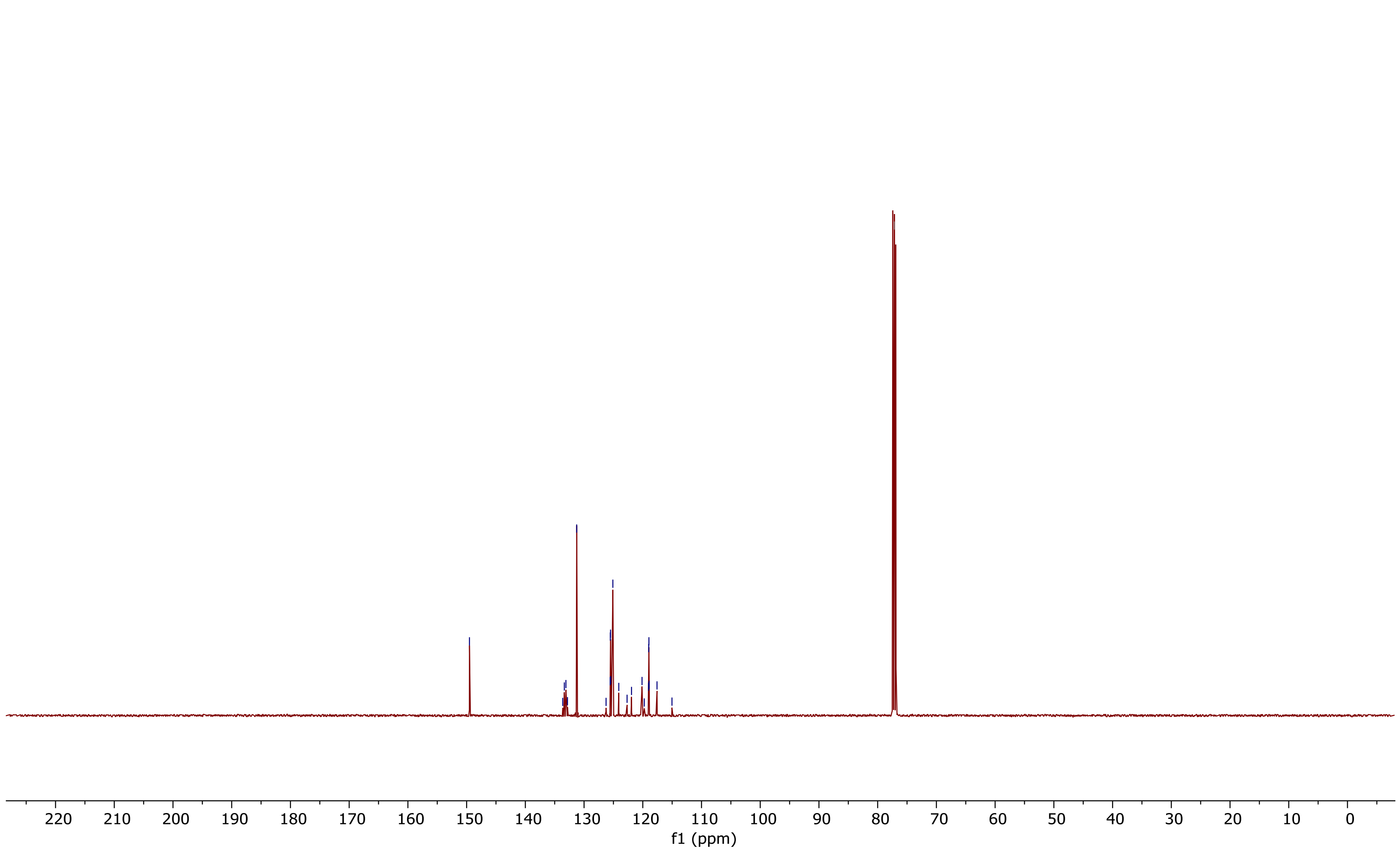

3-(Trifluoromethyl)phenyl trifluoromethanesulfonate - ^{19}F NMR (377 MHz, $CDCl_3$)

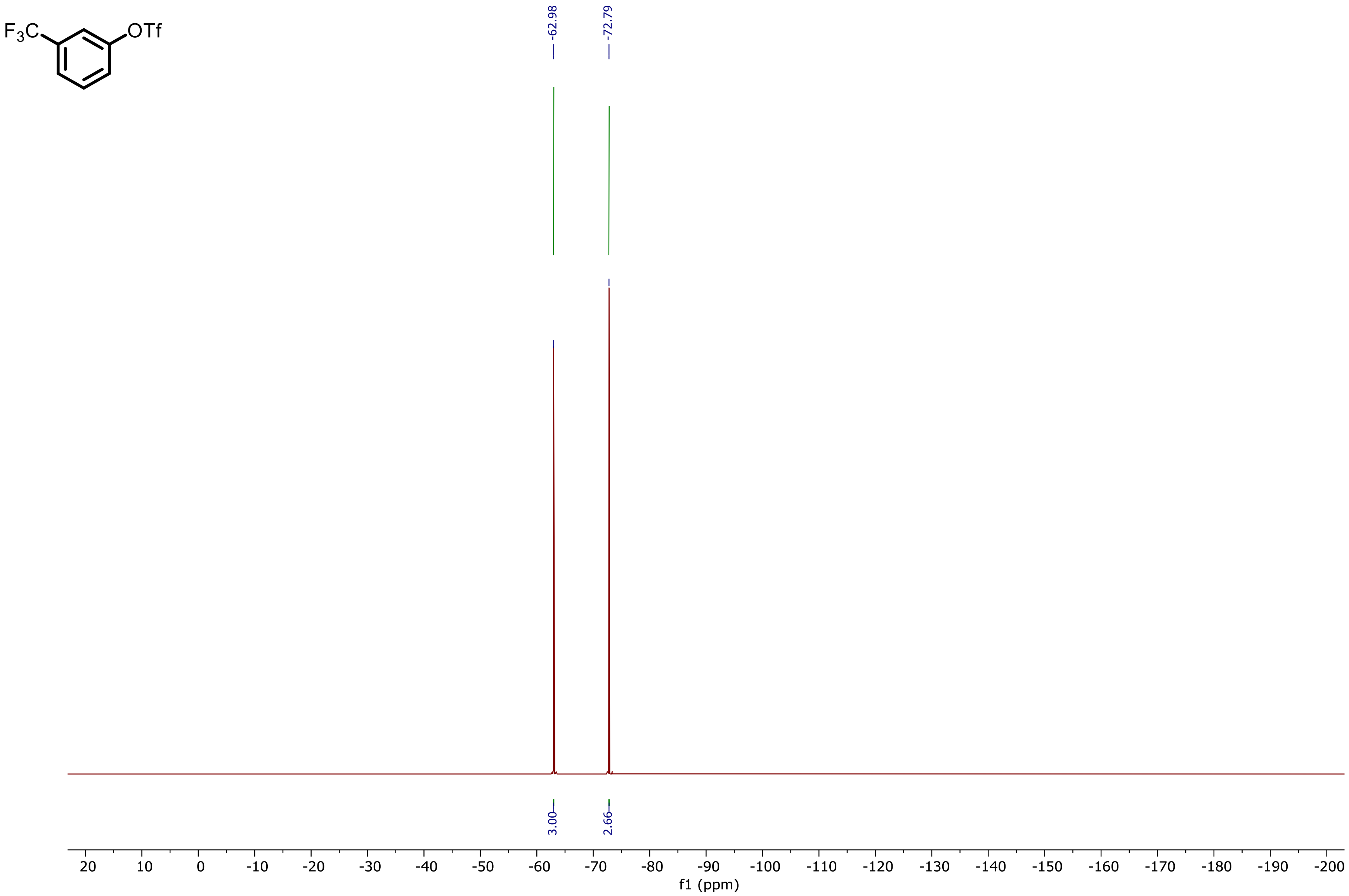

Methyl 4-(((trifluoromethyl)sulfonyl)oxy)benzoate - ^{1}H NMR (400 MHz, $CDCl_3$)

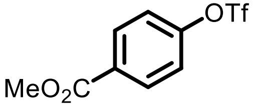

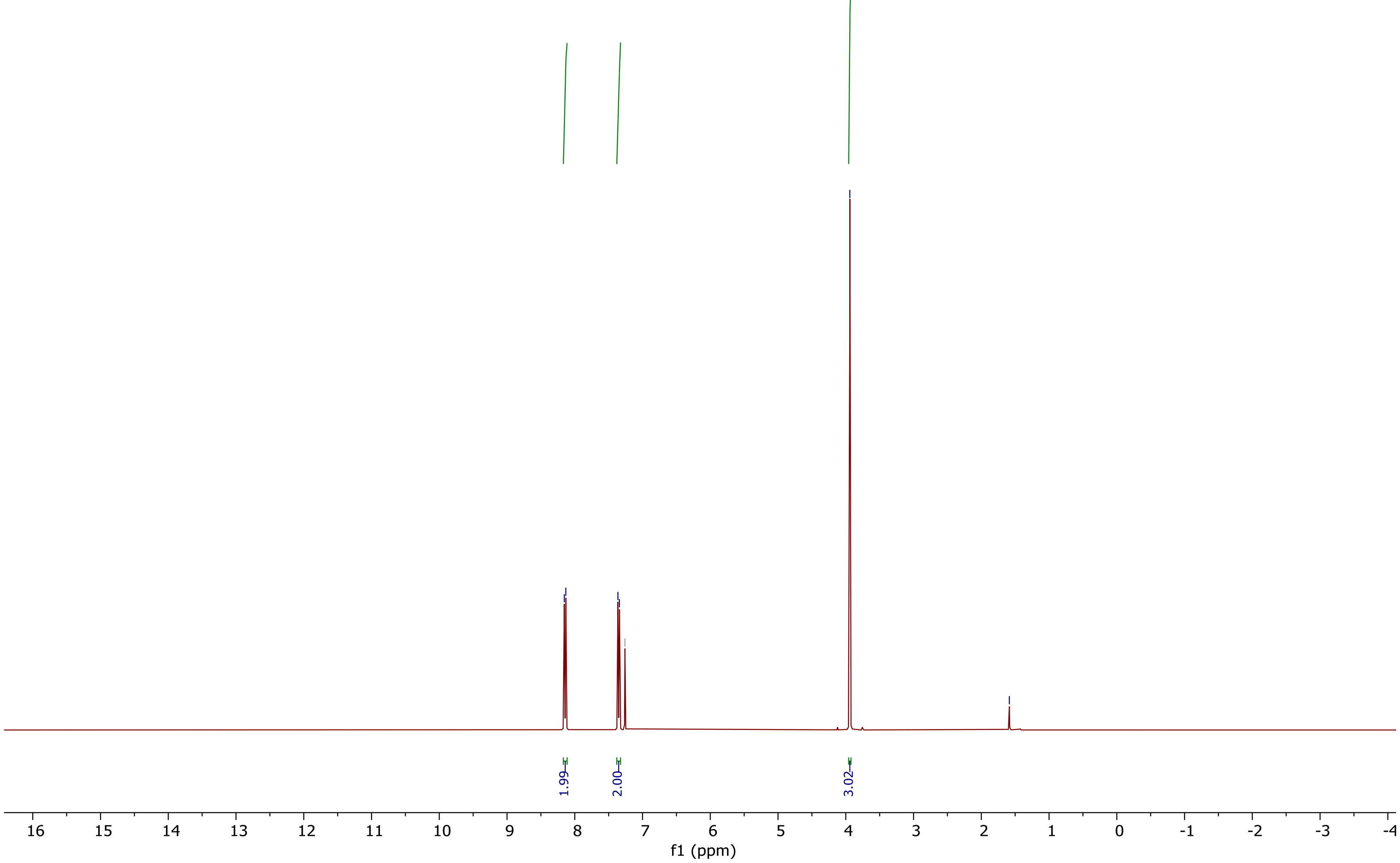

Methyl 4-(((trifluoromethyl)sulfonyl)oxy)benzoate - $^{13}C\{^1H\}$ NMR (101 MHz, $CDCl_3$)

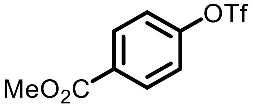

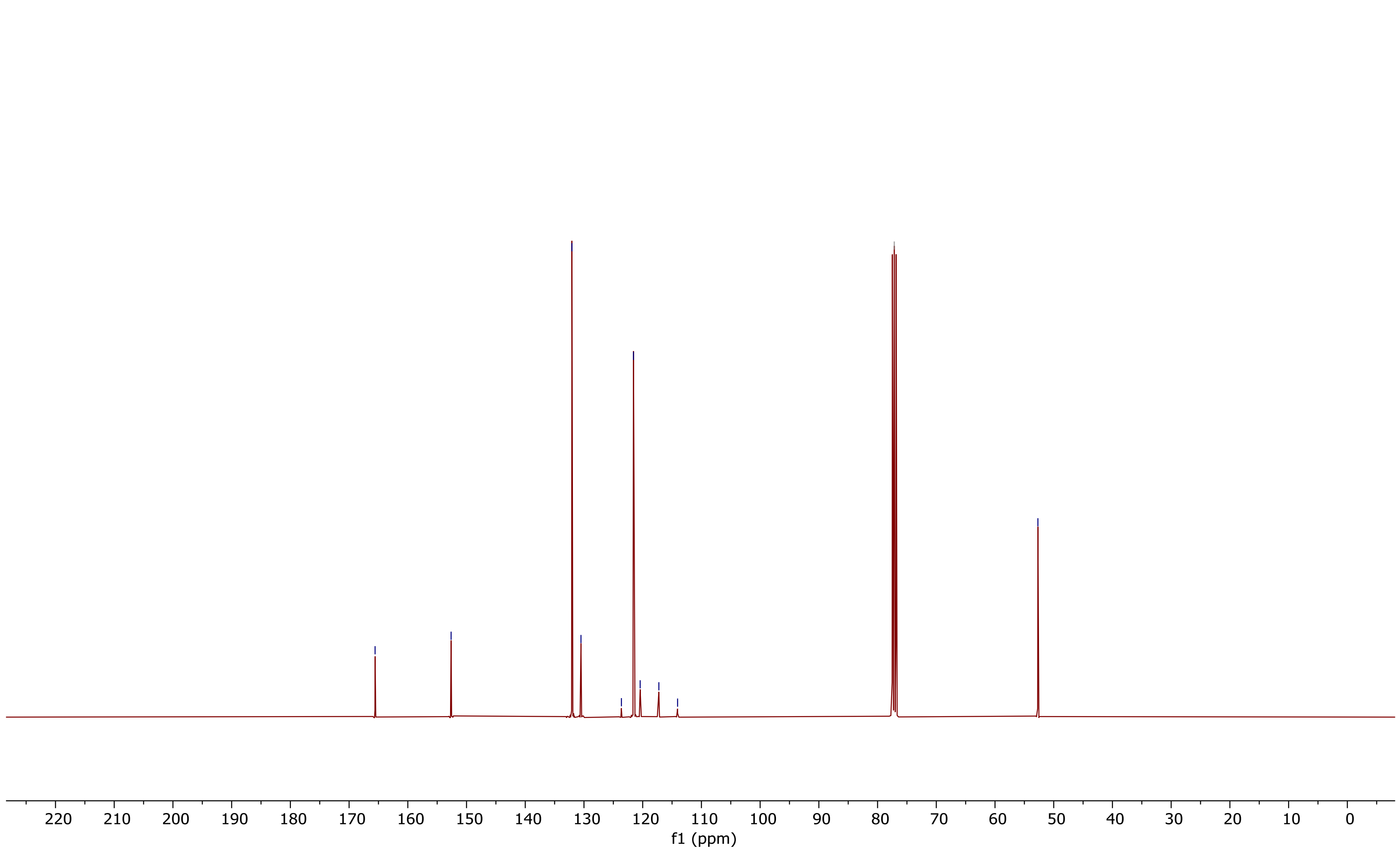

Methyl 4-(((trifluoromethyl)sulfonyl)oxy)benzoate - ^{19}F NMR (377 MHz, $CDCl_3$)

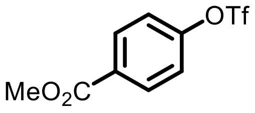

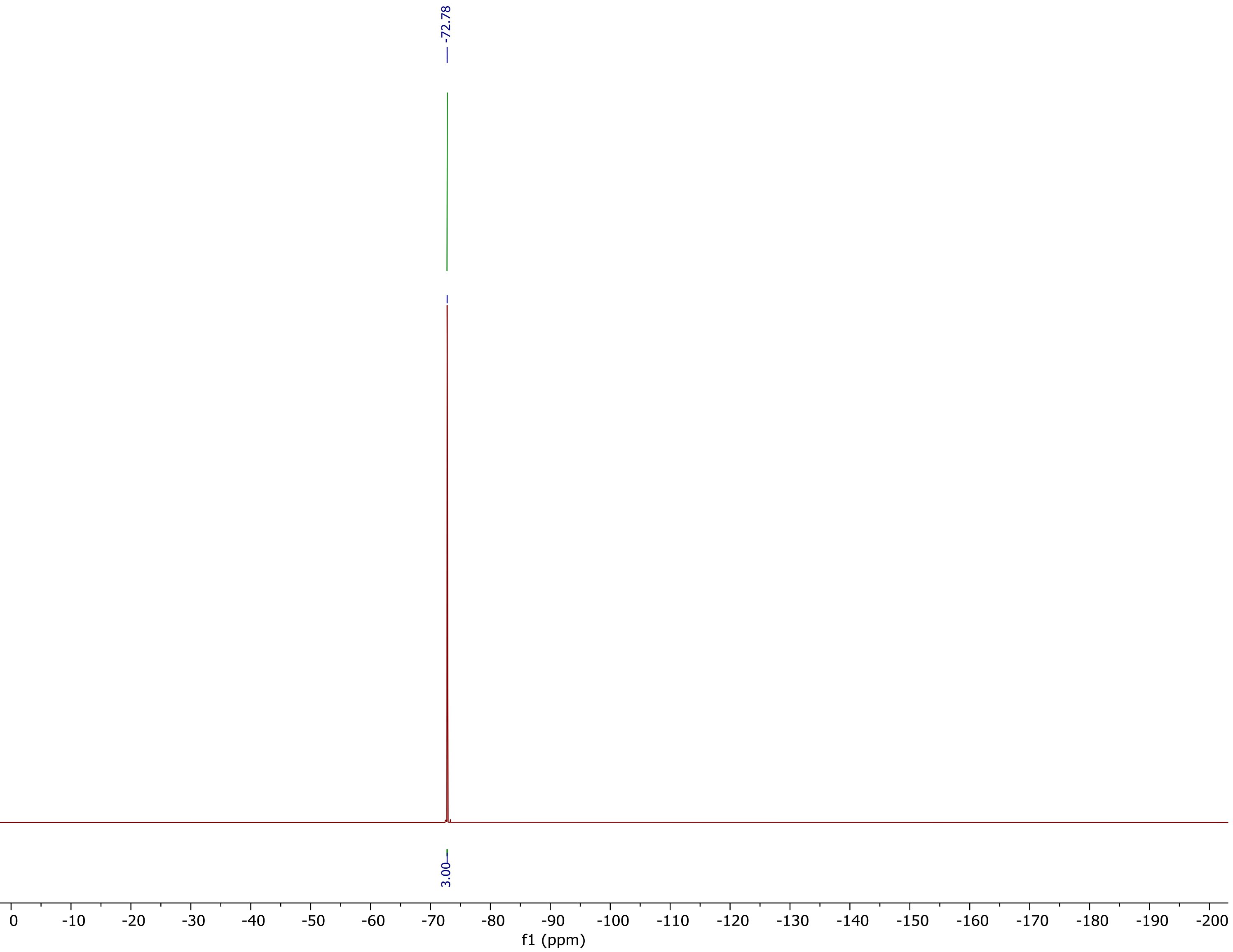

4-(Trifluoromethyl)phenyl trifluoromethanesulfonate - ^1H NMR (400 MHz, $CDCl_3$)

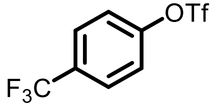

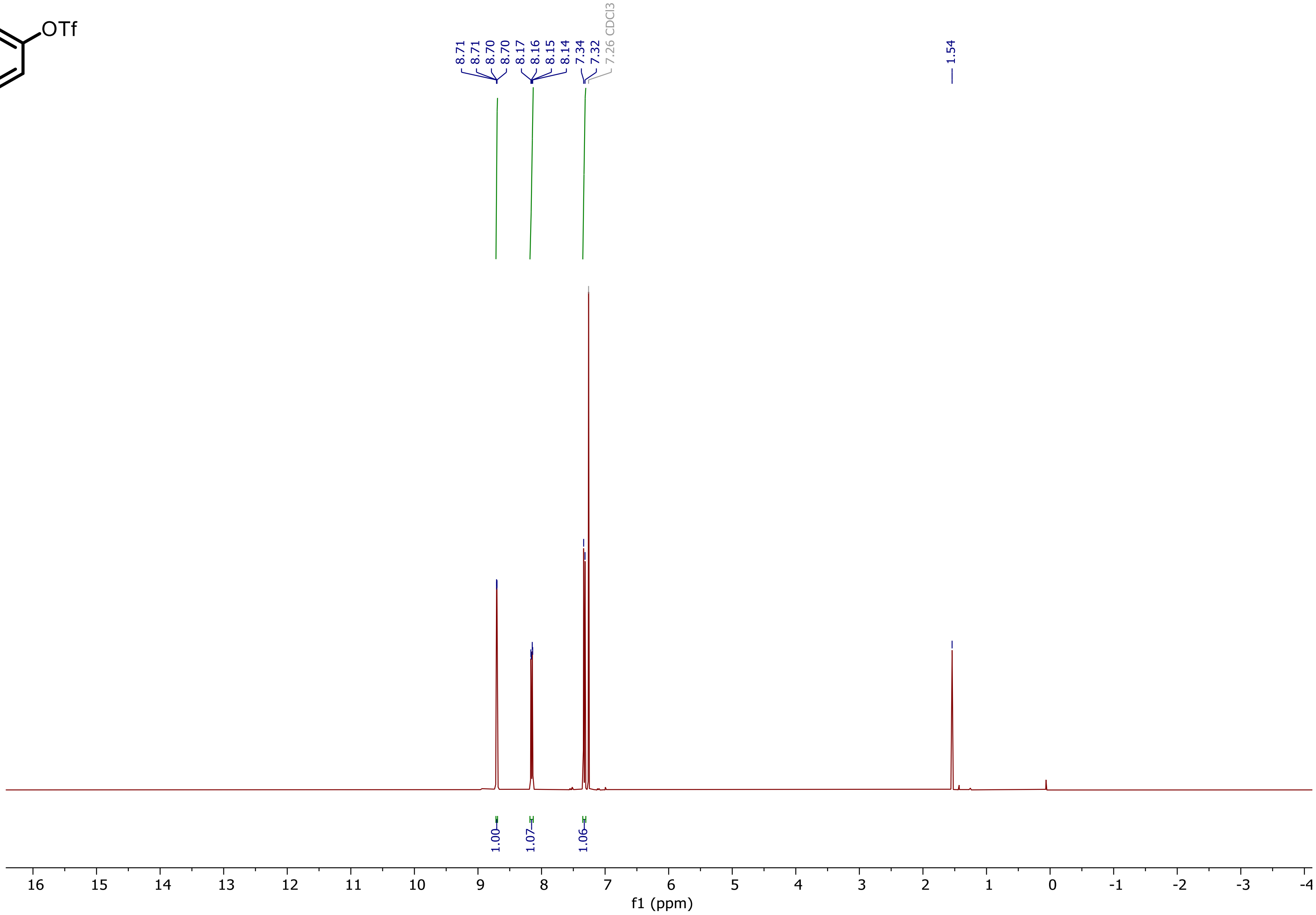

4-(Trifluoromethyl)phenyl trifluoromethanesulfonate - $^{13}C\{^1H\}$ NMR (101 MHz, $CDCl_3$)

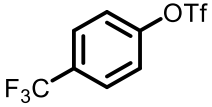

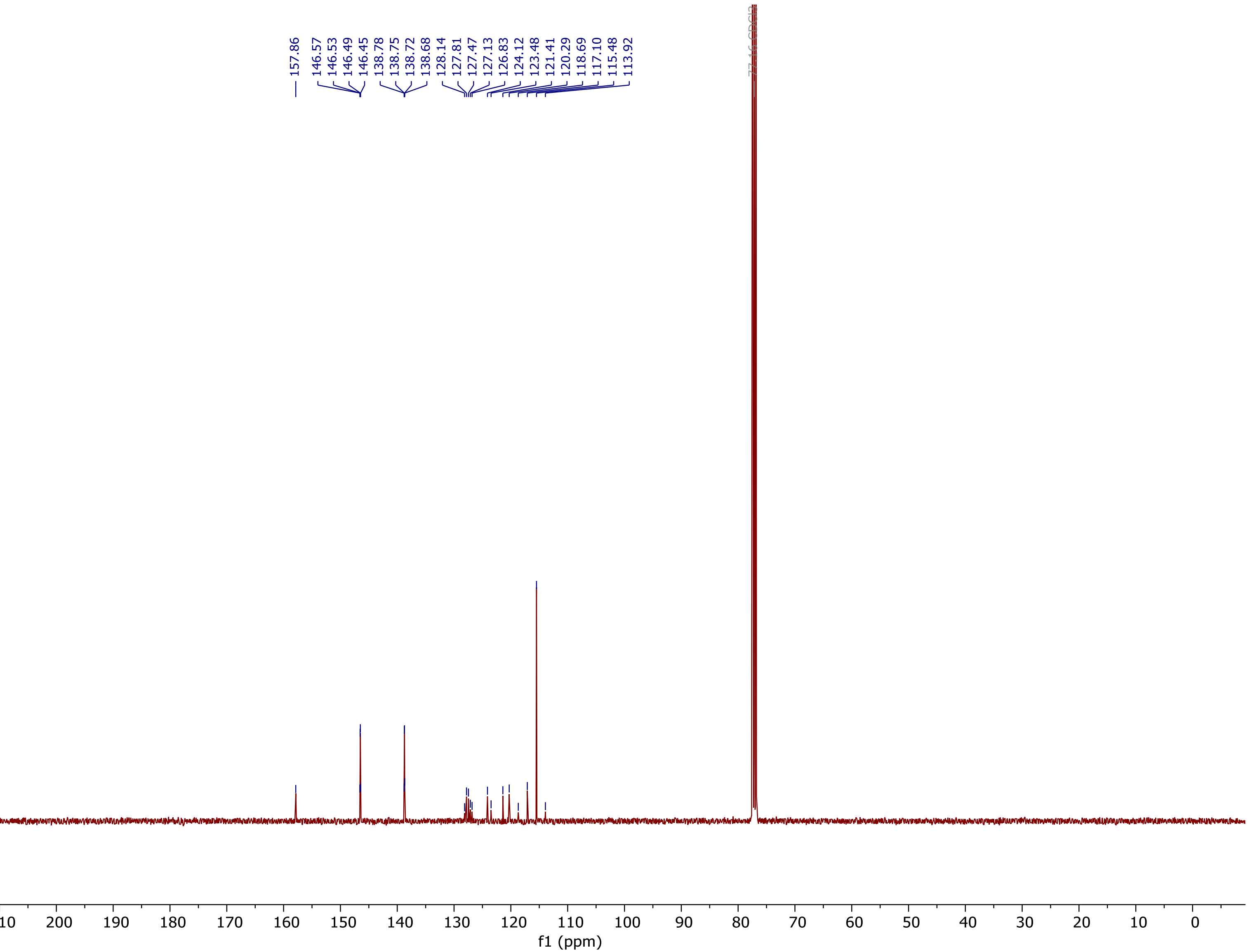

4-(Trifluoromethyl)phenyl trifluoromethanesulfonate - ^{19}F NMR (377 MHz, $CDCl_3$)

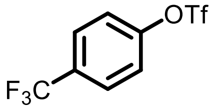

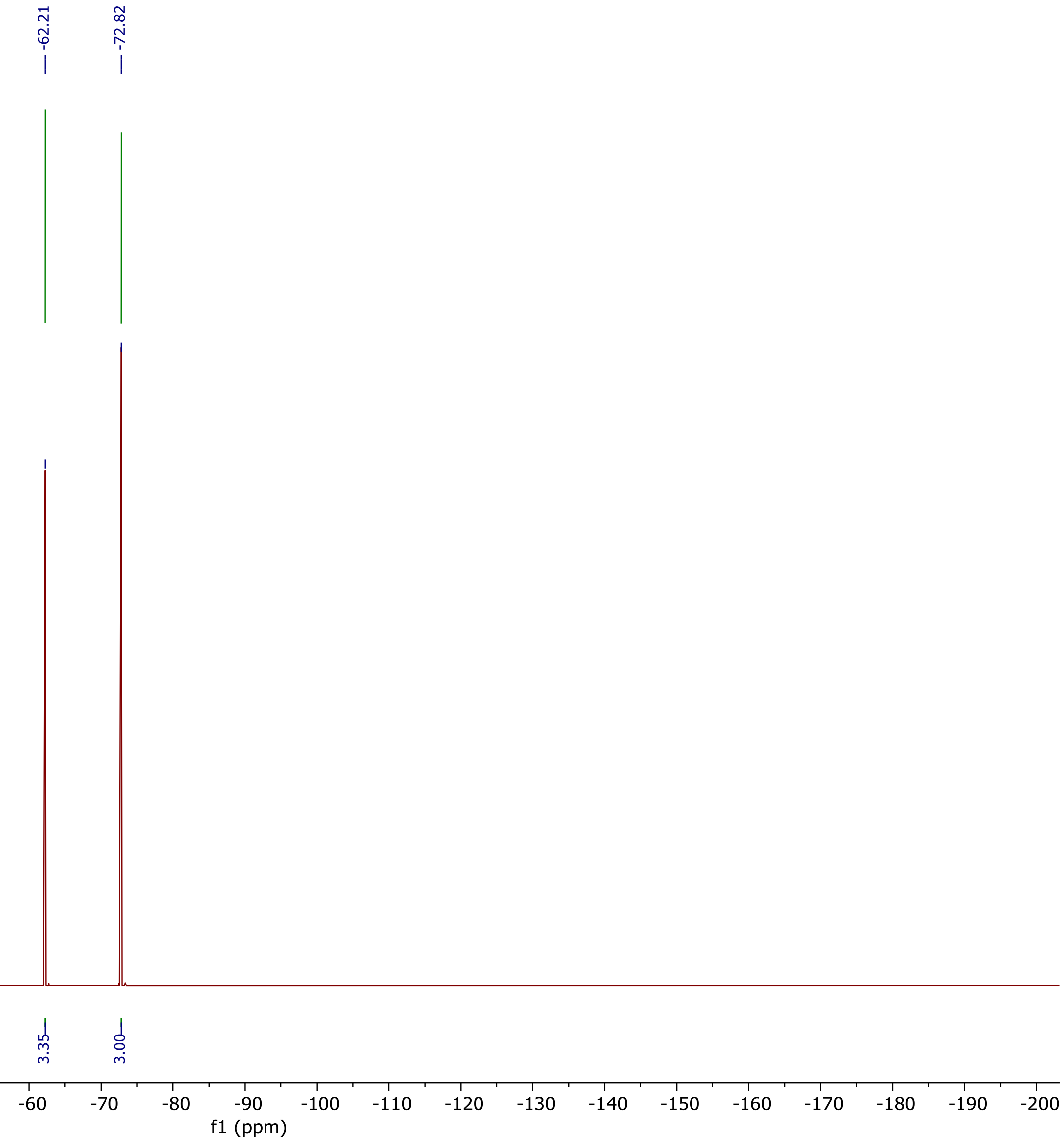

4-Cyanophenyl trifluoromethanesulfonate - ^{1}H NMR (400 MHz, $CDCl_3$)

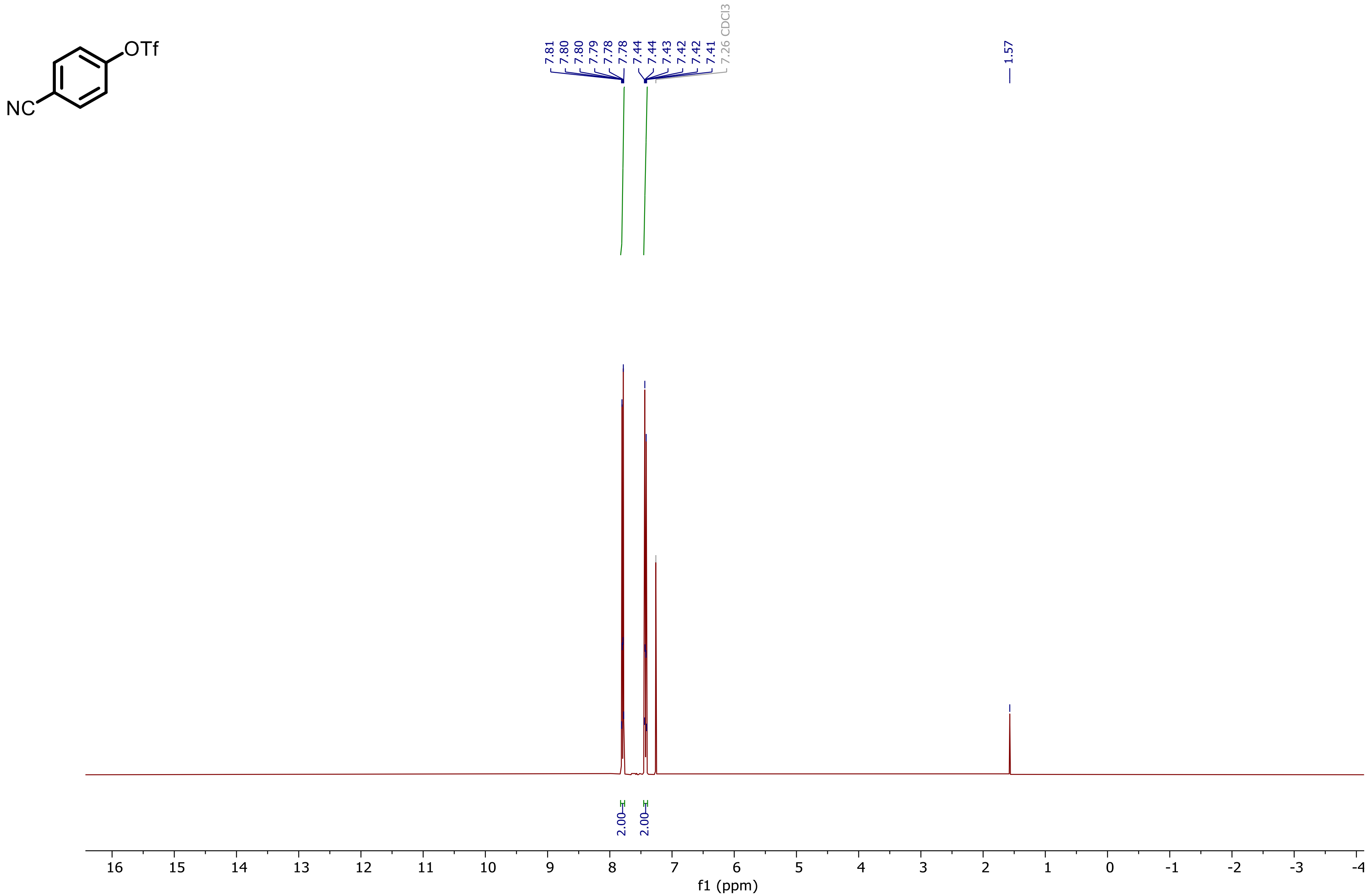

4-Cyanophenyl trifluoromethanesulfonate - $^{13}C\{^1H\}$ NMR (101 MHz, $CDCl_3$)

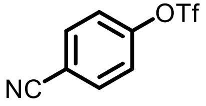

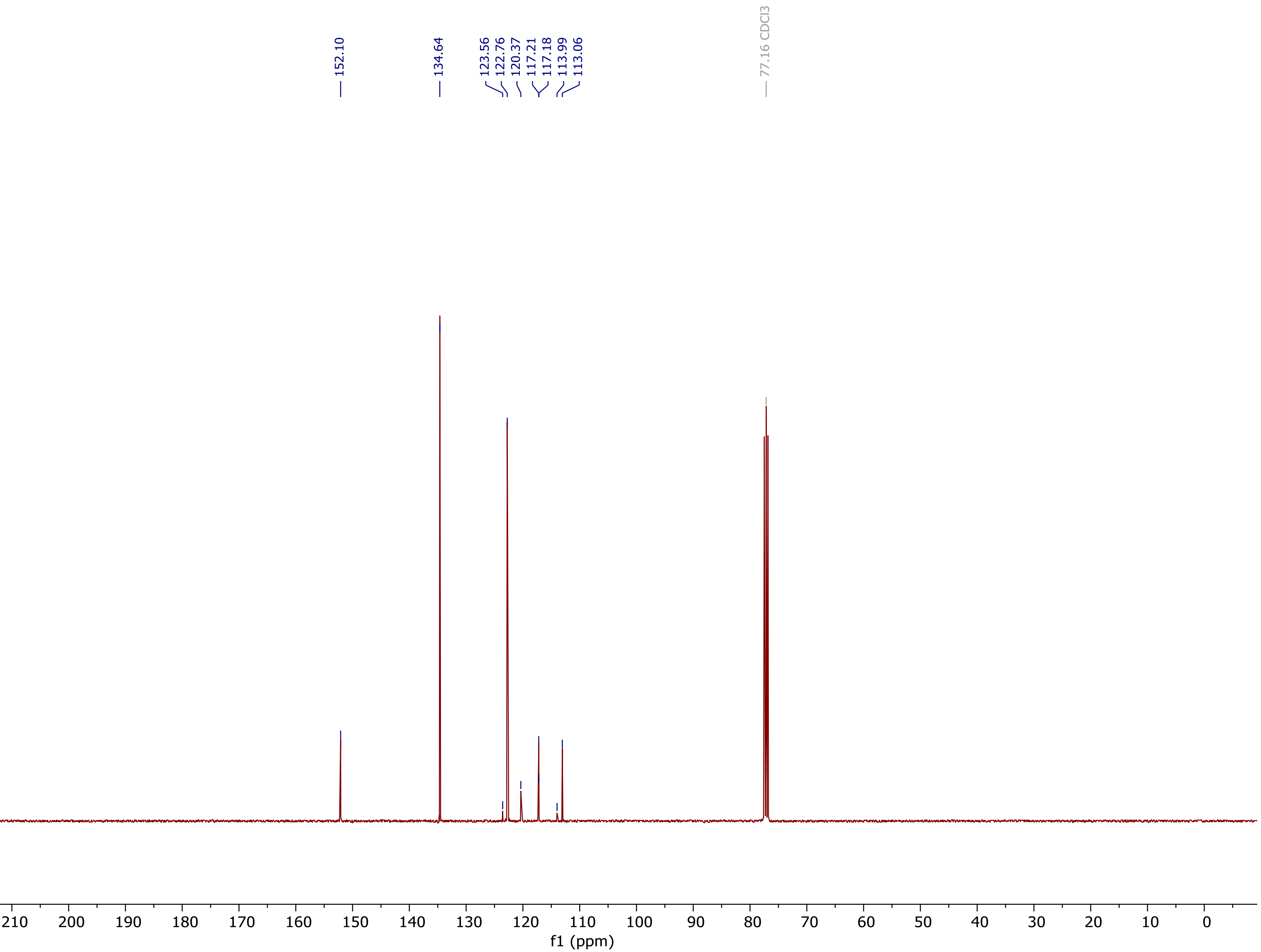

4-Cyanophenyl trifluoromethanesulfonate - ^{19}F NMR (376 MHz, $CDCl_3$)

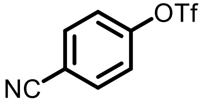

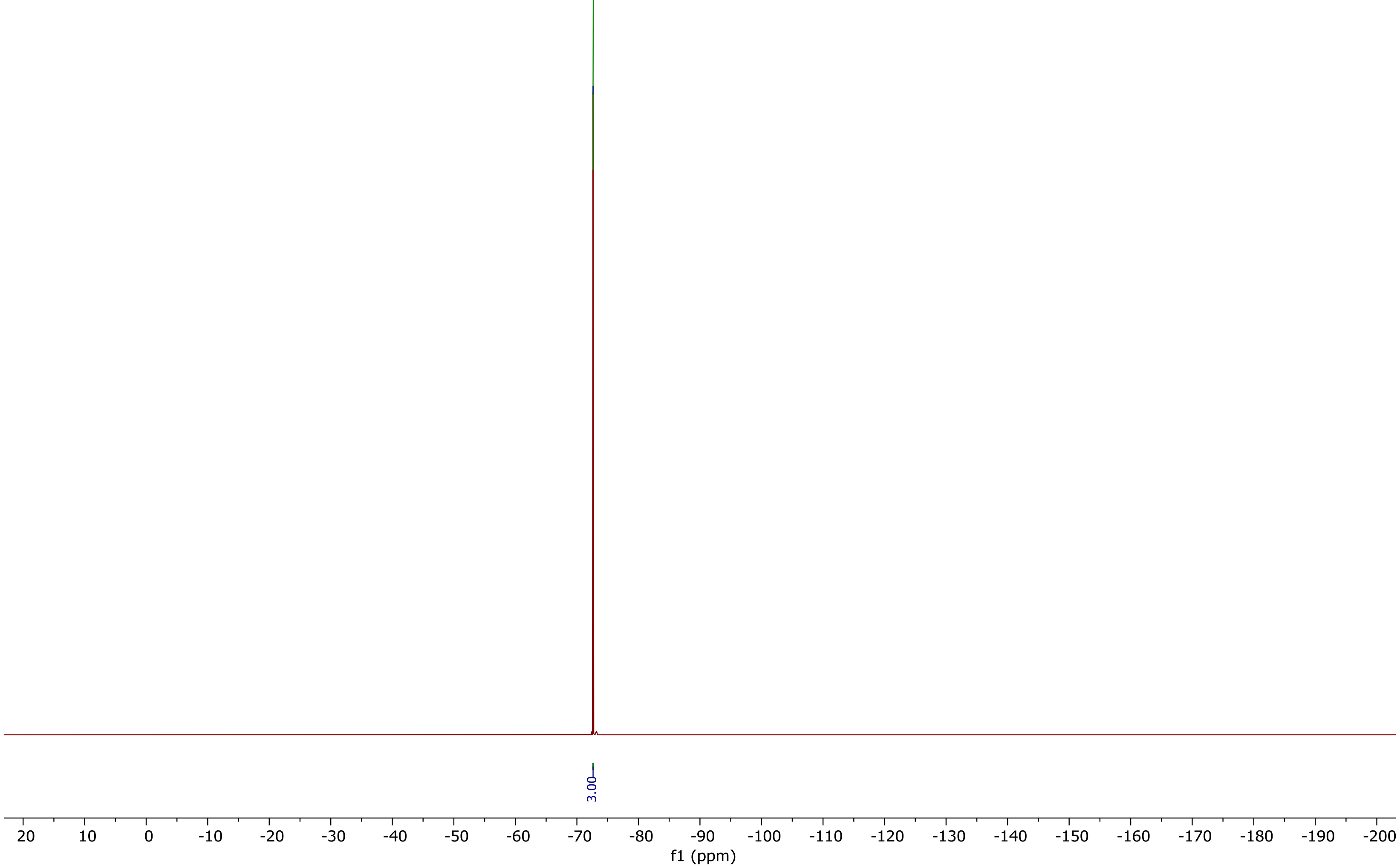

3,5-Difluorophenyl trifluoromethanesulfonate - ^{1}H NMR (400 MHz, $CDCl_3$)

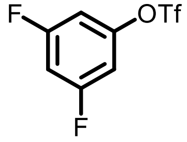

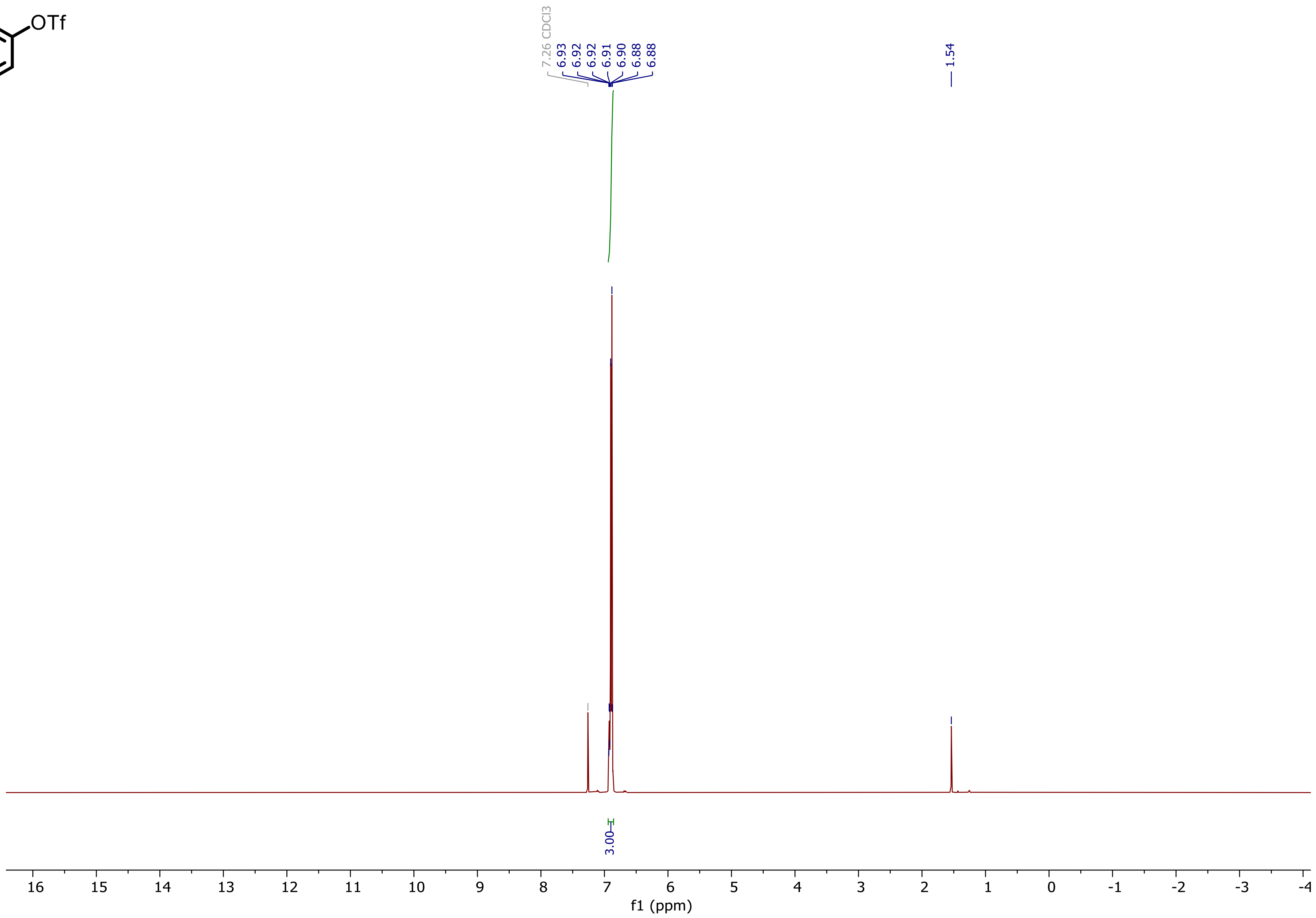

3,5-Difluorophenyl trifluoromethanesulfonate - $^{13}C\{^1H\}$ NMR (101 MHz, $CDCl_3$)

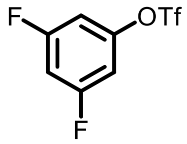

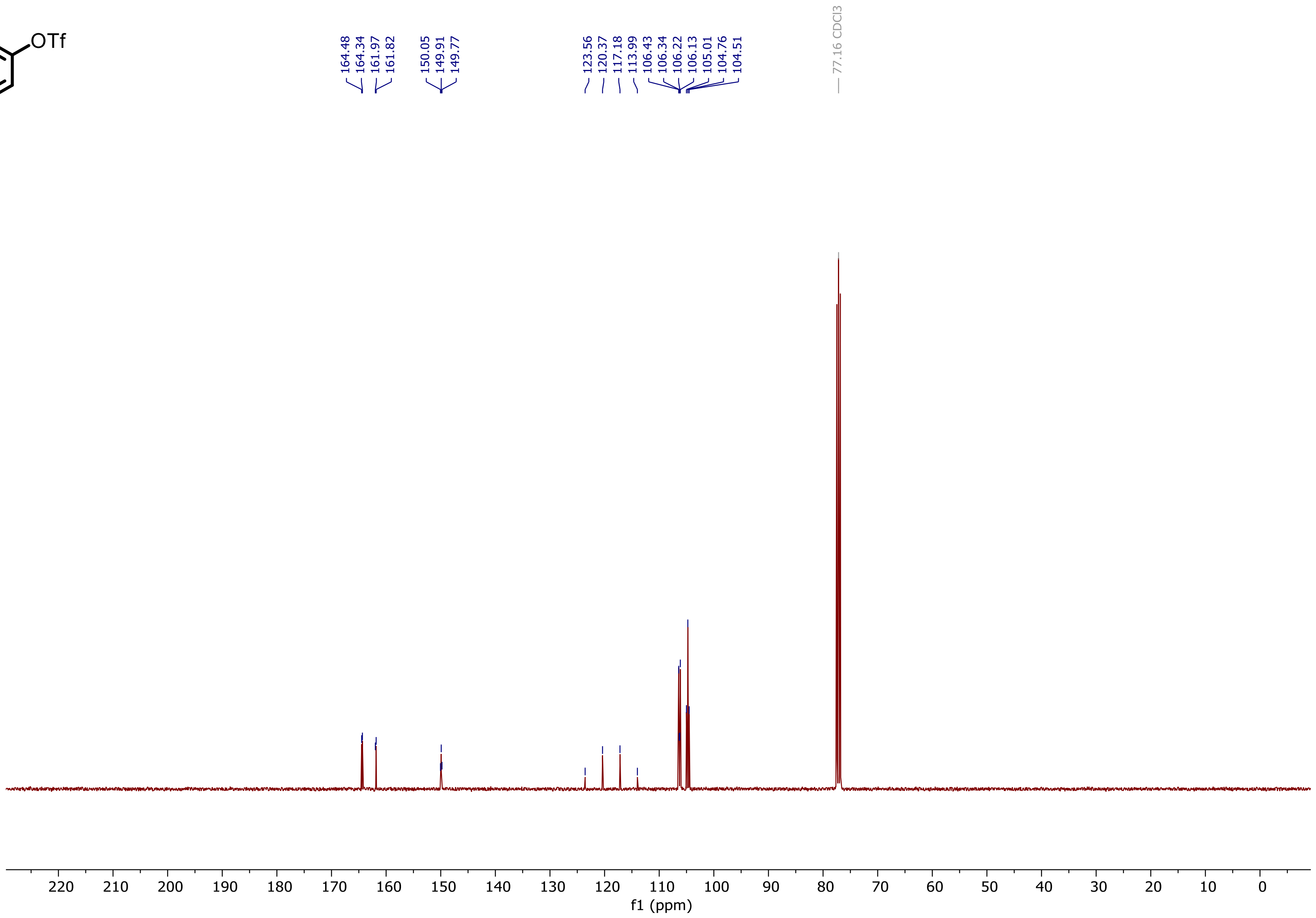

3,5-Difluorophenyl trifluoromethanesulfonate - ^{19}F NMR (377 MHz, $CDCl_3$)

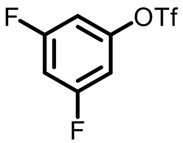

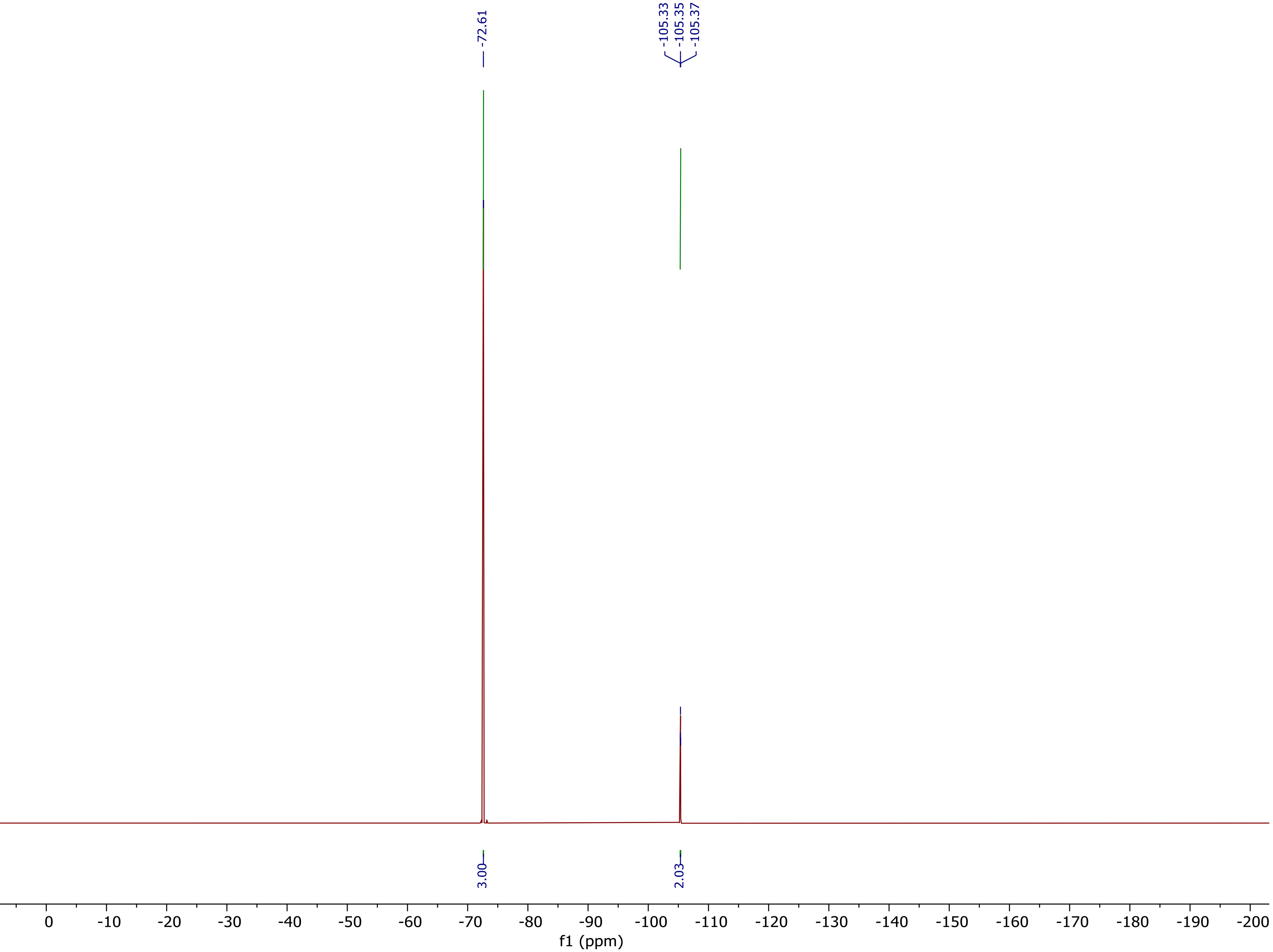

4-Nitrophenyl trifluoromethanesulfonate - ^{1}H NMR (400 MHz, $CDCl_3$)

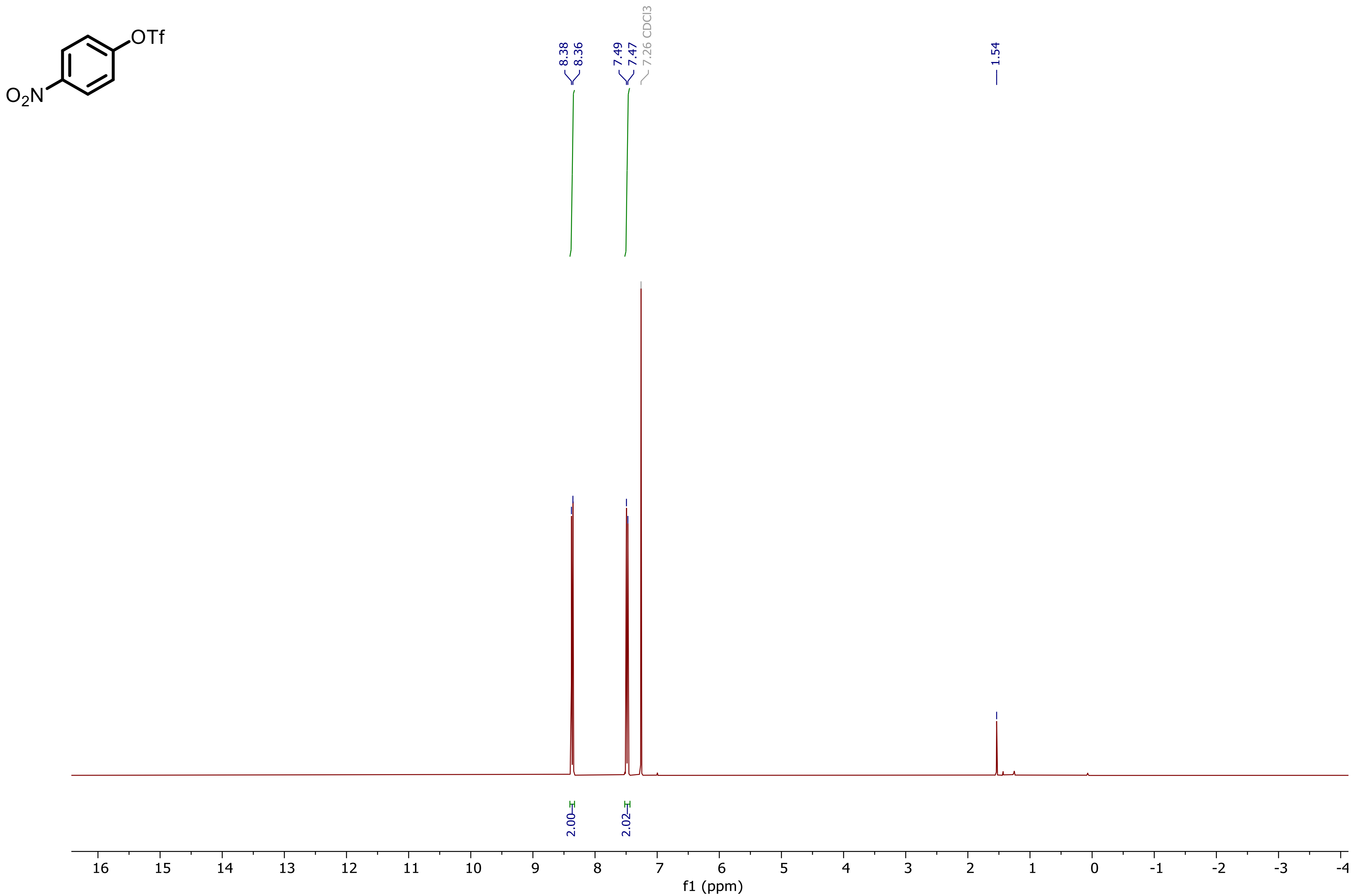

4-Nitrophenyl trifluoromethanesulfonate - $^{13}C\{^{1}H\}$ NMR (101 MHz, $CDCl_3$)

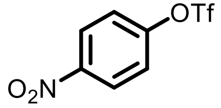

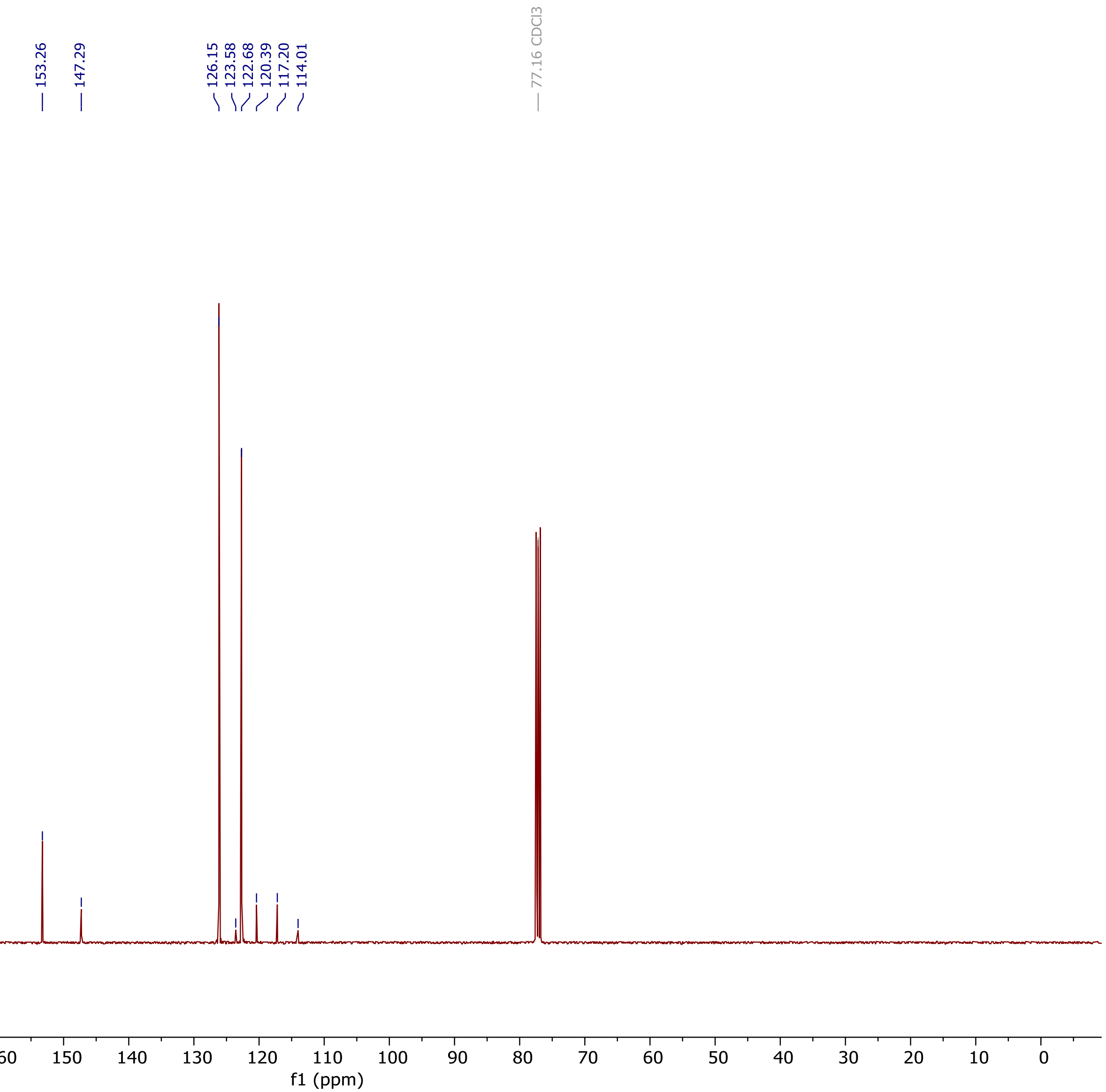

4-Nitrophenyl trifluoromethanesulfonate - ^{19}F NMR (376 MHz, $CDCl_3$)

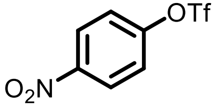

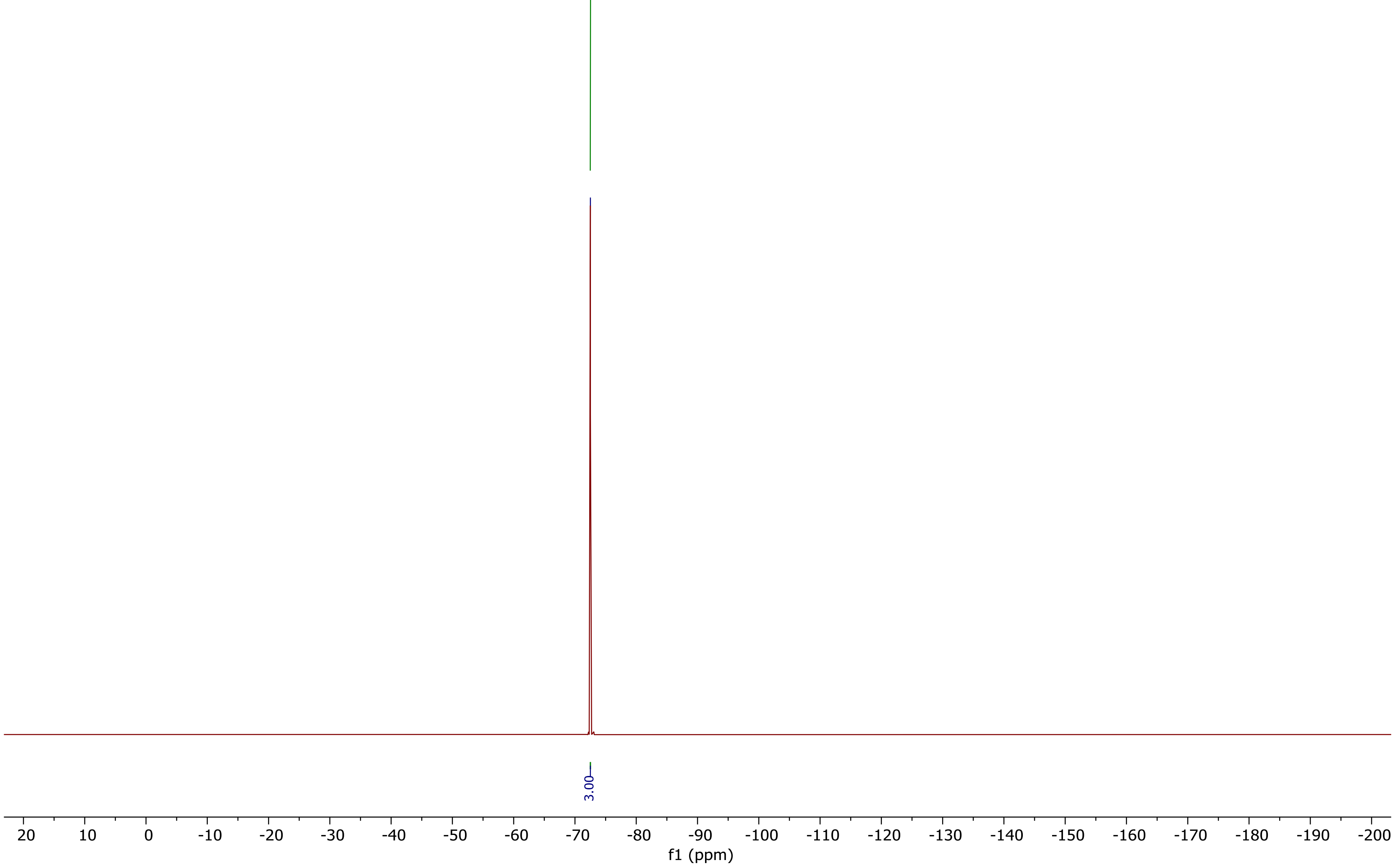

2-(Benzyloxy)phenyl trifluoromethanesulfonate - ^{1}H NMR (400 MHz, $CDCl_3$)

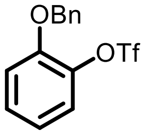

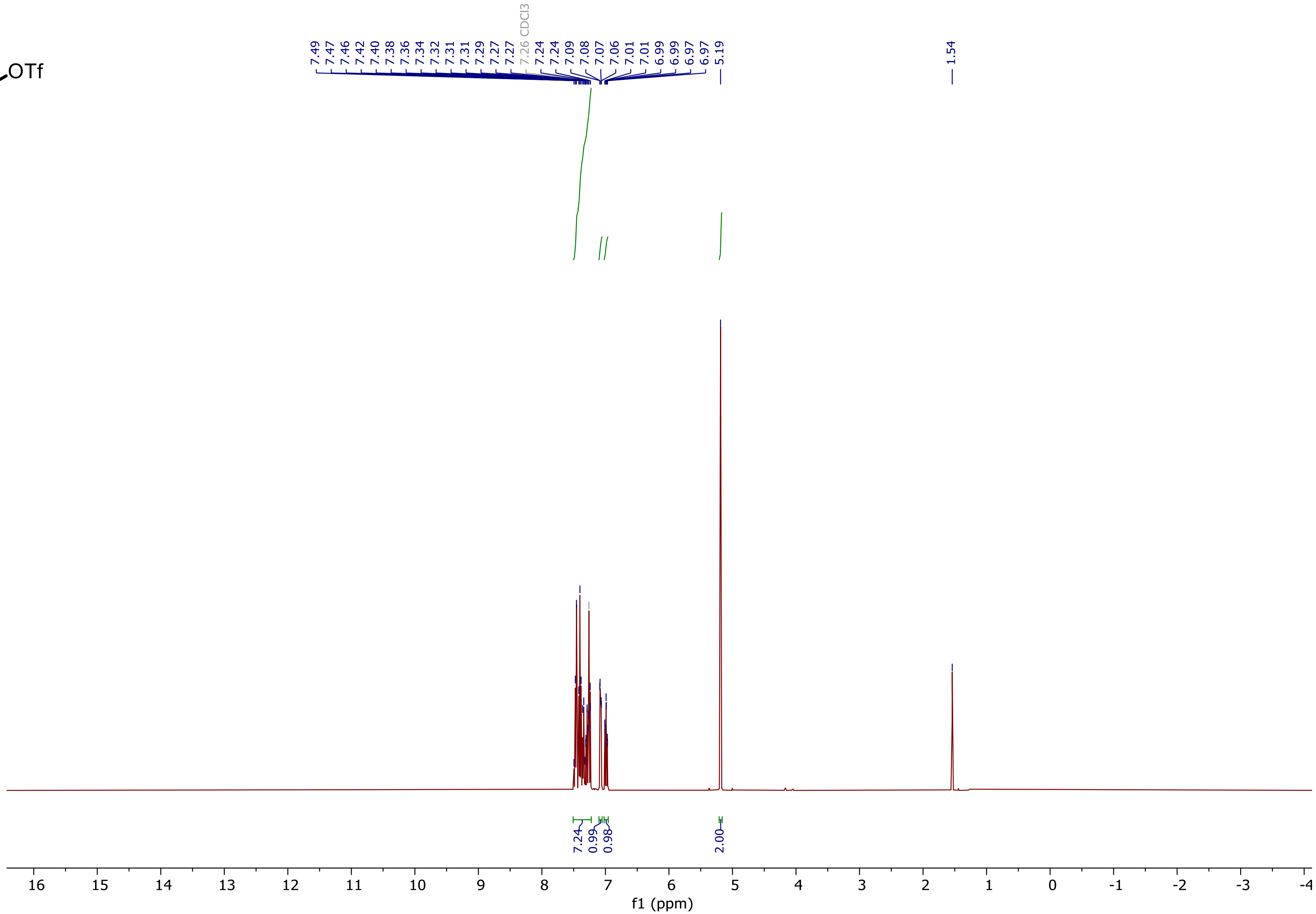

2-(Benzyloxy)phenyl trifluoromethanesulfonate - $^{13}C\{^1H\}$ NMR (101 MHz, $CDCl_3$)

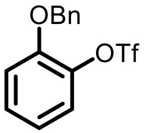

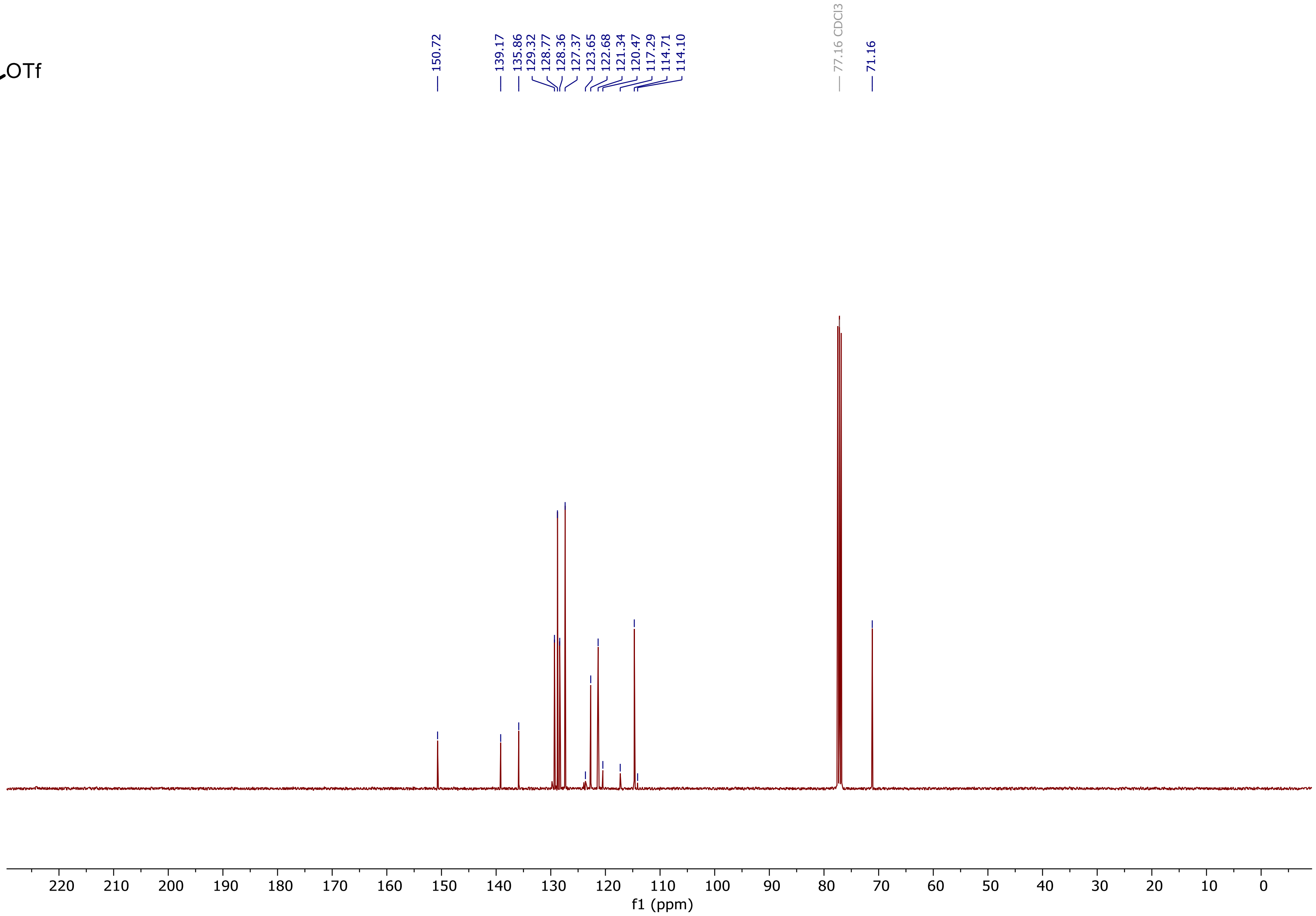

2-(Benzyloxy)phenyl trifluoromethanesulfonate - ^{19}F NMR (376 MHz, $CDCl_3$)

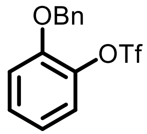

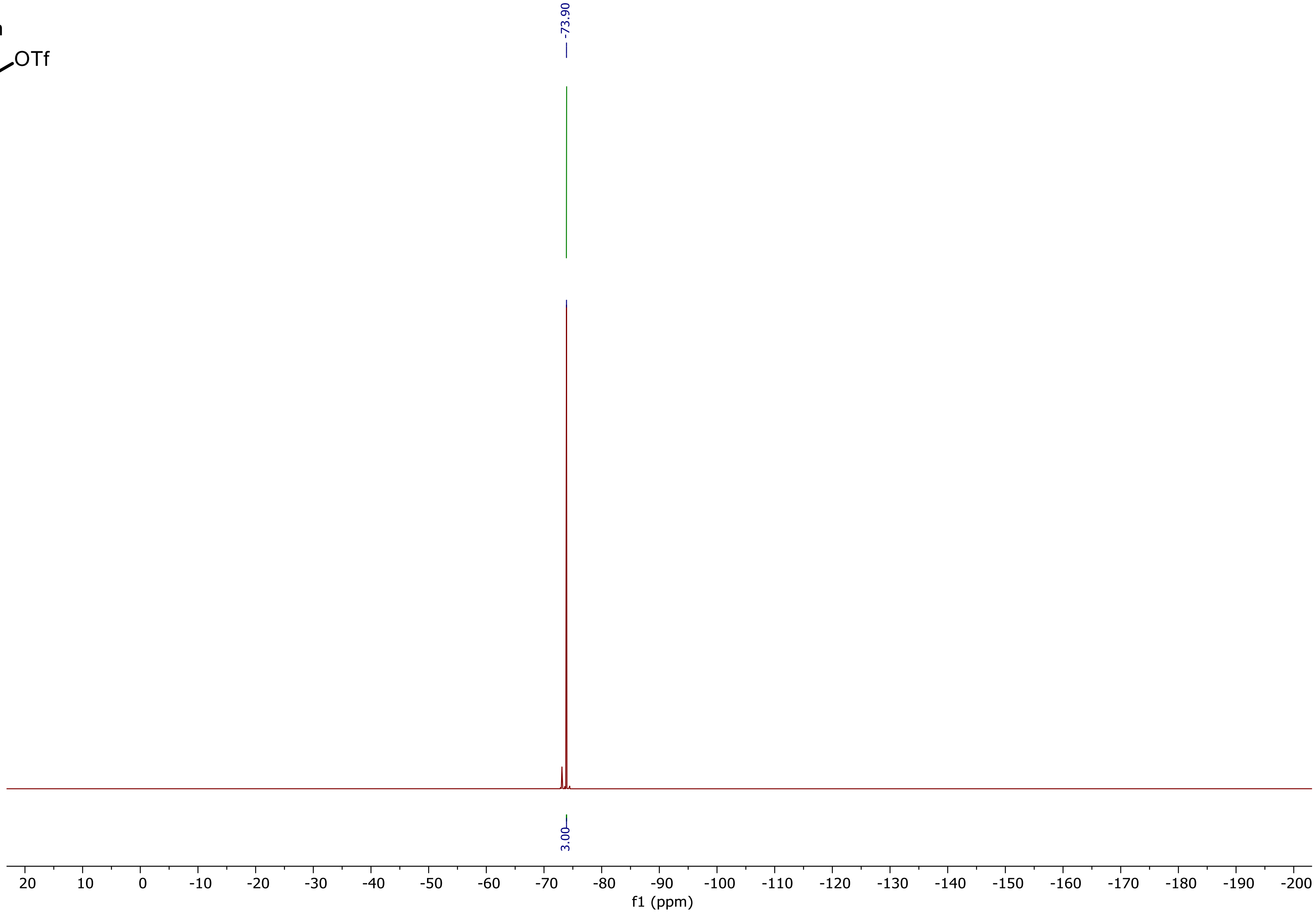

Ethyl (*S*)-2,5,7,8-tetramethyl-6-(((trifluoromethyl)sulfonyl)oxy)chromane-2-carboxylate - ^{1}H NMR (500 MHz, $CDCl_3$)

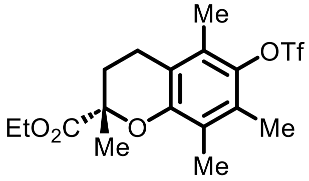

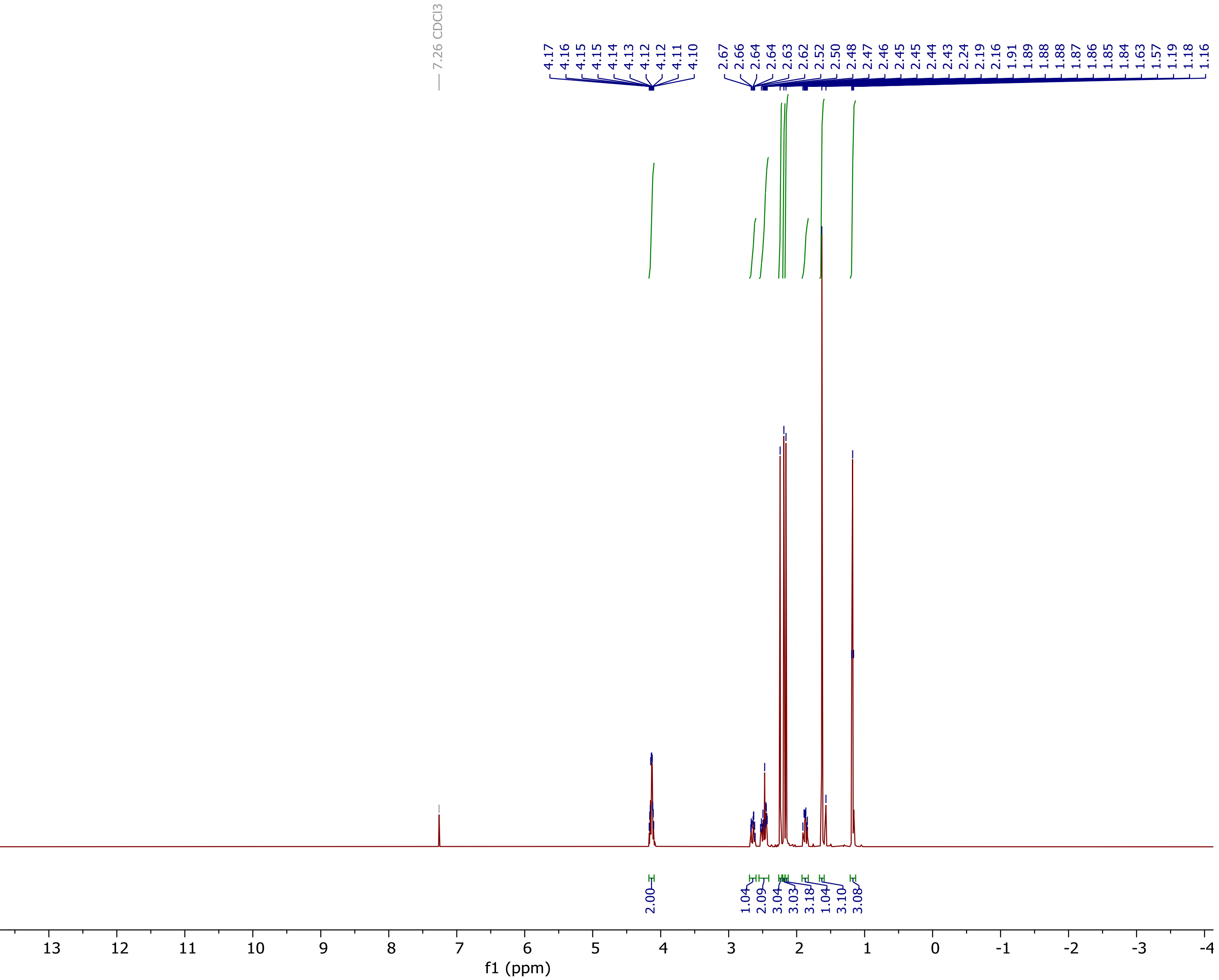

Ethyl (*S*)-2,5,7,8-tetramethyl-6-(((trifluoromethyl)sulfonyl)oxy)chromane-2-carboxylate - $^{13}C\{^{1}H\}$ NMR (126 MHz, $CDCl_3$)

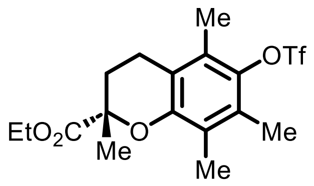

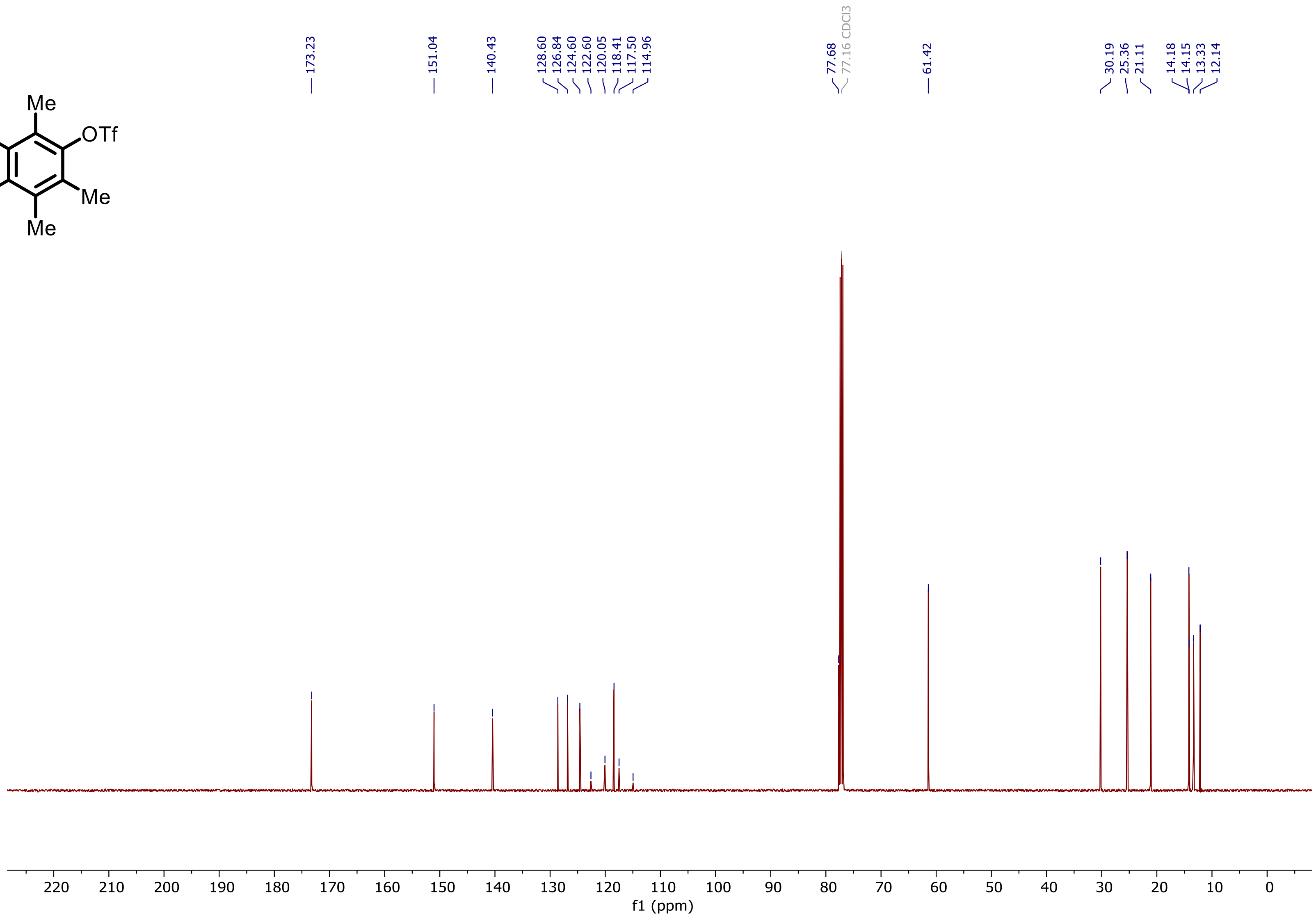

Ethyl (*S*)-2,5,7,8-tetramethyl-6-(((trifluoromethyl)sulfonyl)oxy)chromane-2-carboxylate - ^{19}F NMR (376 MHz, $CDCl_3$)

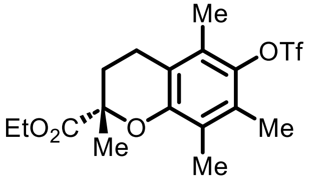

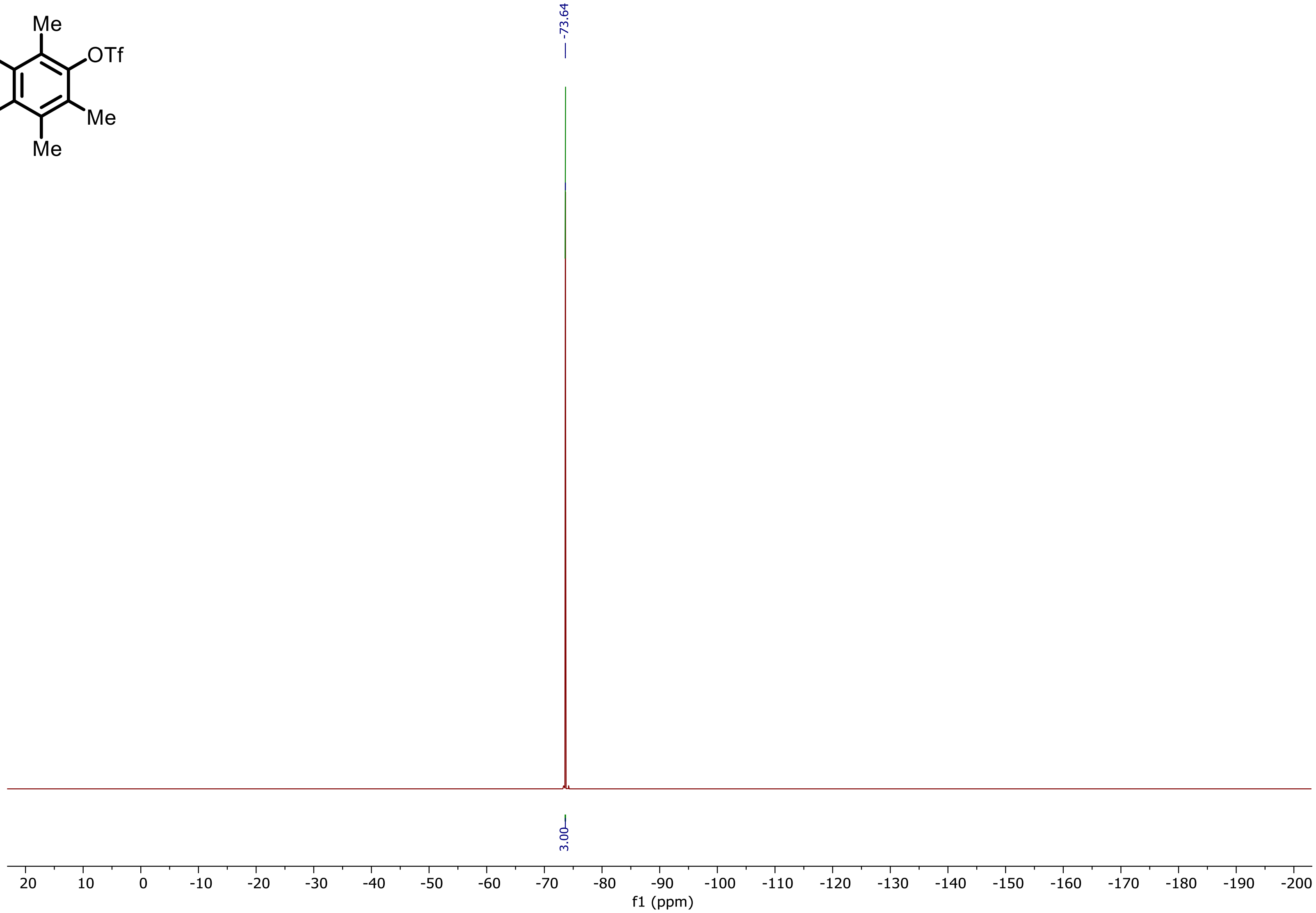

tert-Butyl 3-(2-(1,3-dioxoisoindolin-2-yl)ethyl)-5-(((trifluoromethyl)sulfonyl)oxy)-1*H*-indole-1-carboxylate - ^{1}H NMR (400 MHz, $CDCl_3$)

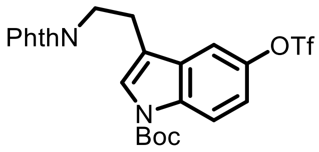

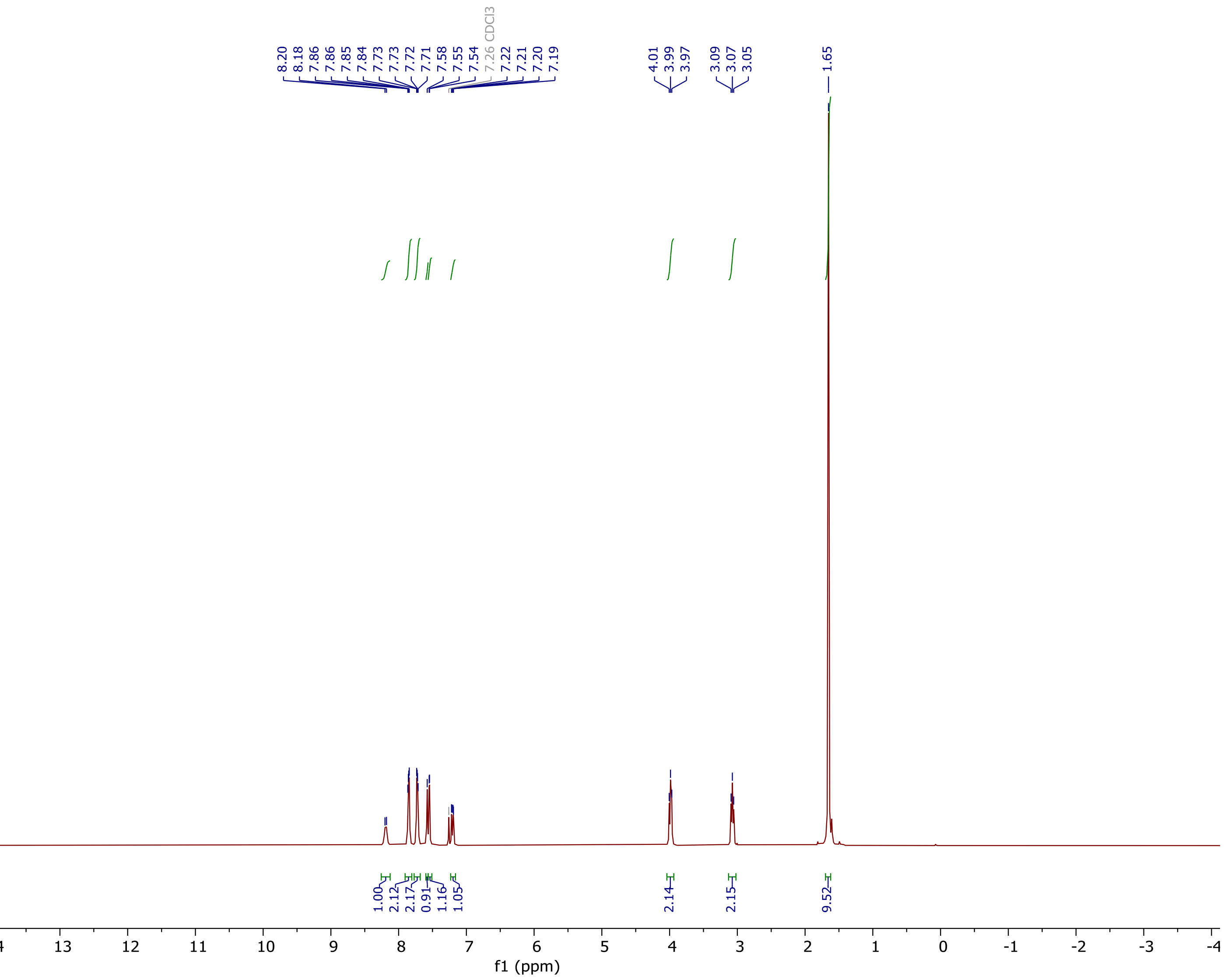

tert-Butyl 3-(2-(1,3-dioxoisoindolin-2-yl)ethyl)-5-(((trifluoromethyl)sulfonyl)oxy)-1H-indole-1-carboxylate - $^{13}C\{^1H\}$ NMR (101 MHz, $CDCl_3$)

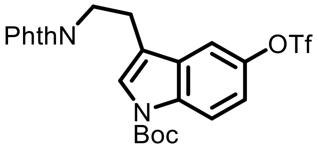

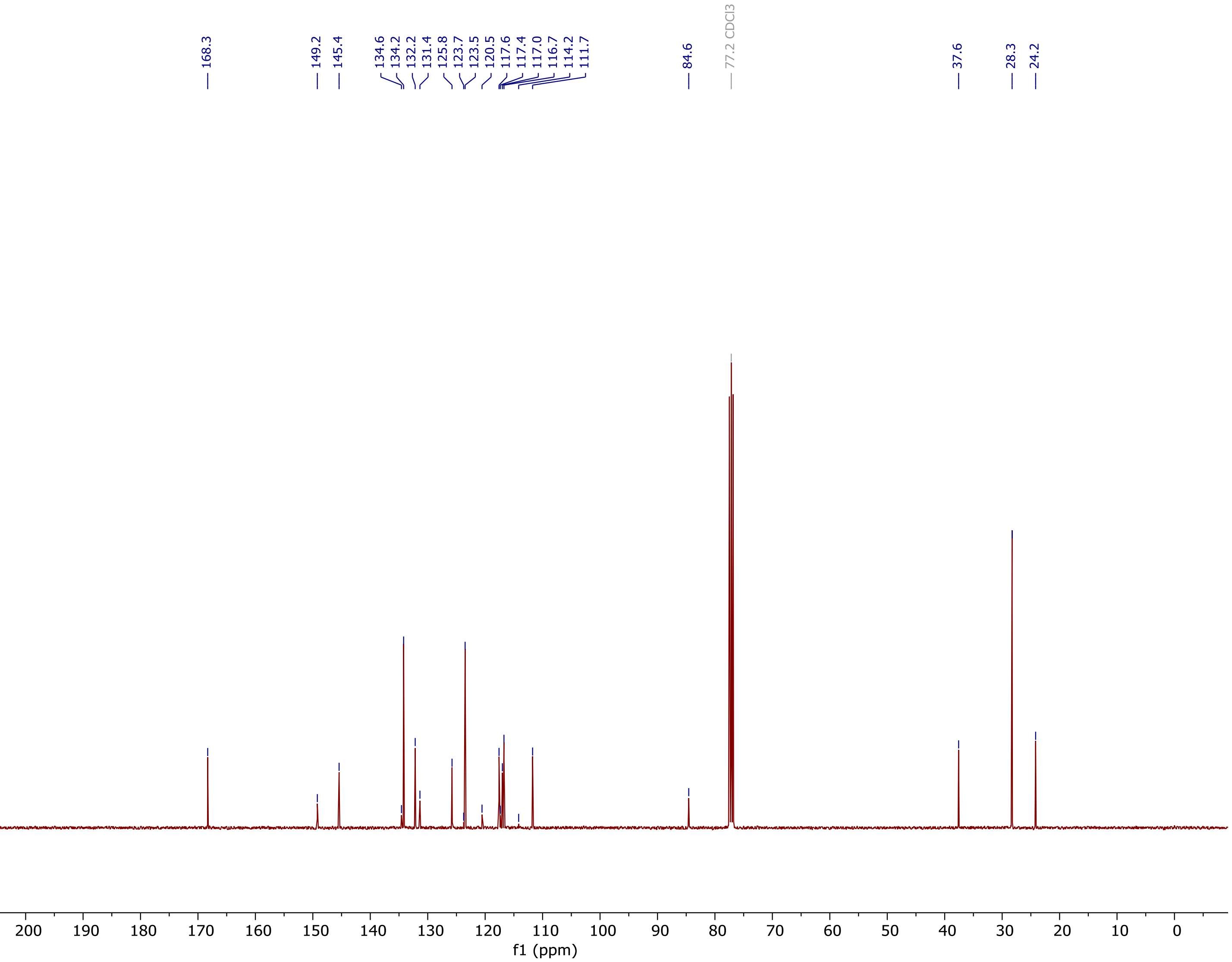

***tert*-Butyl 3-(2-(1,3-dioxoisoindolin-2-yl)ethyl)-5-(((trifluoromethyl)sulfonyl)oxy)-1*H*-indole-1-carboxylate - ^{19}F NMR (376 MHz, $CDCl_3$)**

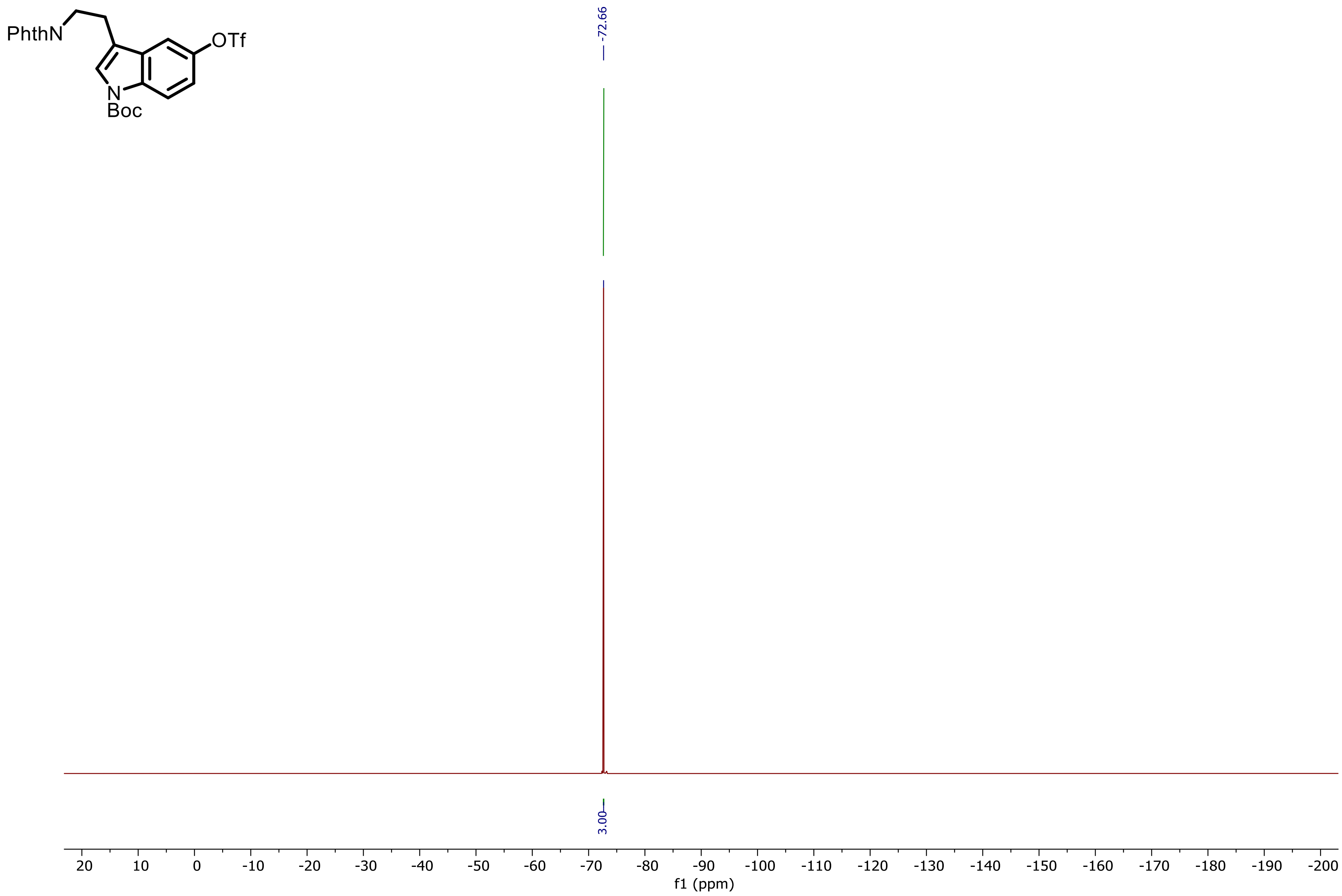

5-(Trifluoromethyl)pyridin-2-yl trifluoromethanesulfonate - ^{1}H NMR (400 MHz, $CDCl_3$)

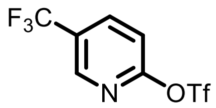

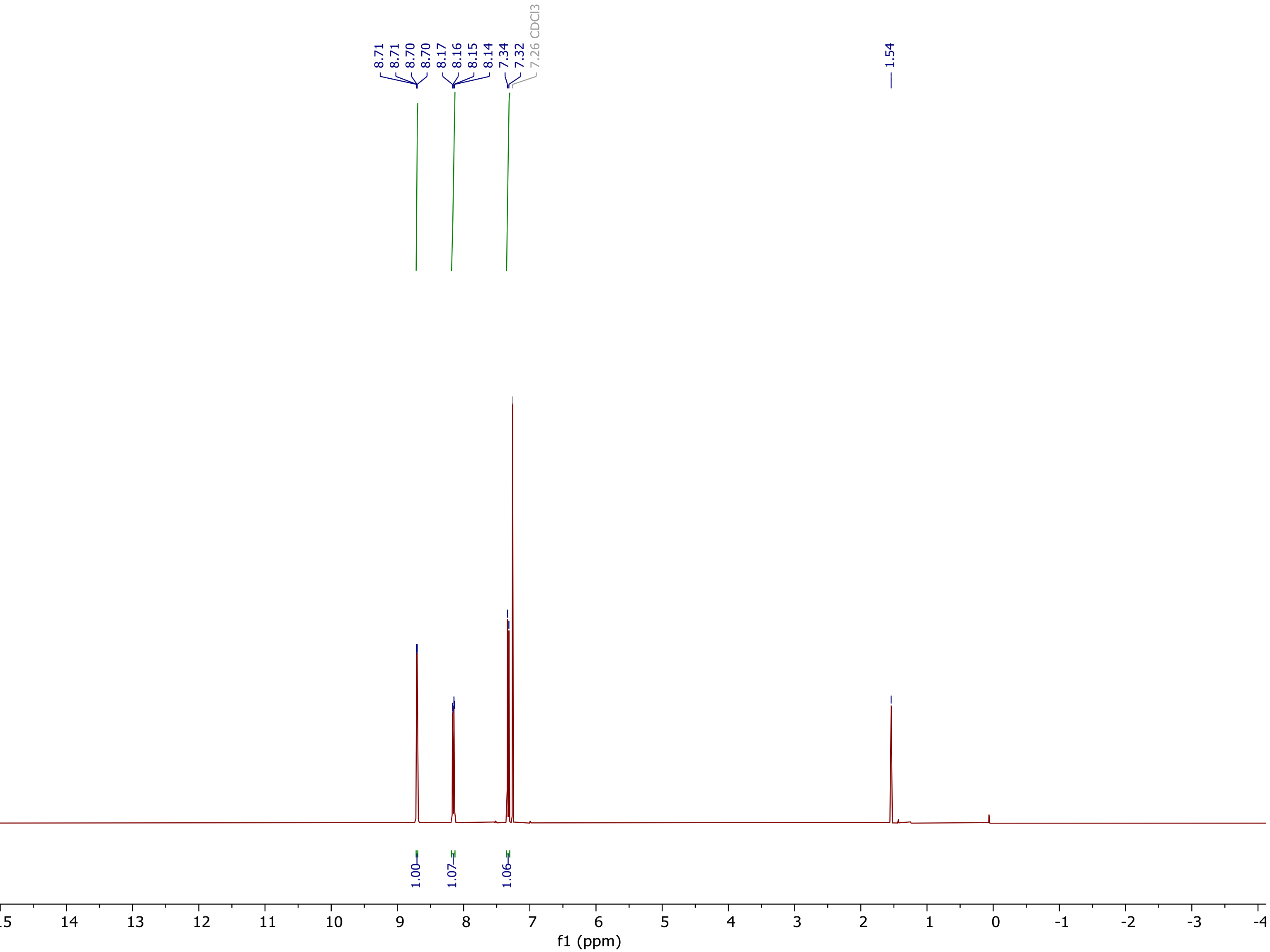

5-(Trifluoromethyl)pyridin-2-yl trifluoromethanesulfonate - $^{13}C\{^{1}H\}$ NMR (101 MHz, $CDCl_3$)

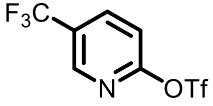

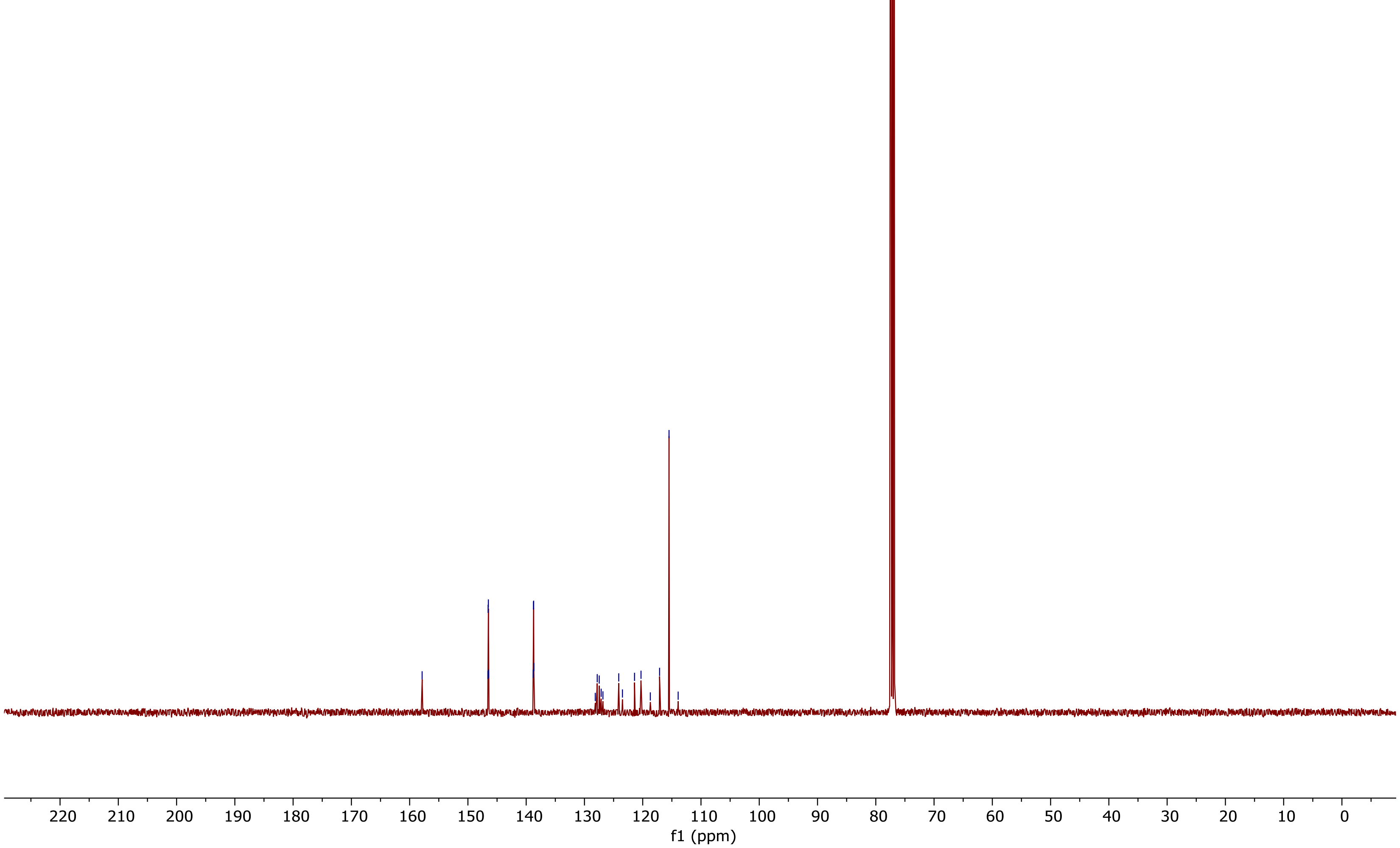

5-(Trifluoromethyl)pyridin-2-yl trifluoromethanesulfonate - ^{19}F NMR (376 MHz, $CDCl_3$)

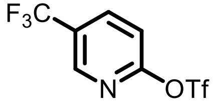

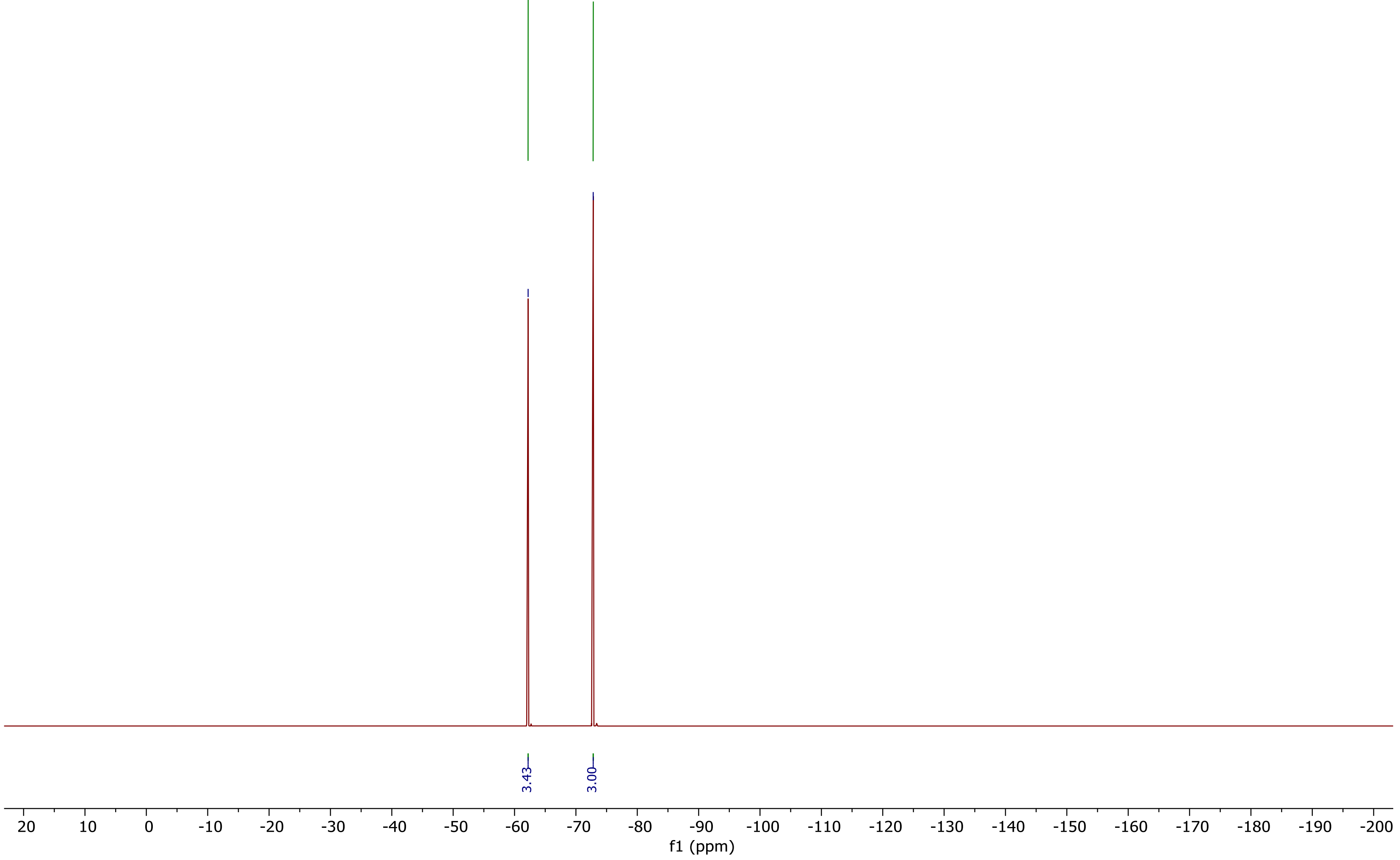

4-Fluoropyridin-2-yl trifluoromethanesulfonate – ¹H NMR (400 MHz, $CDCl_3$)

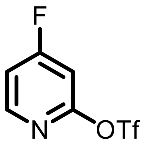

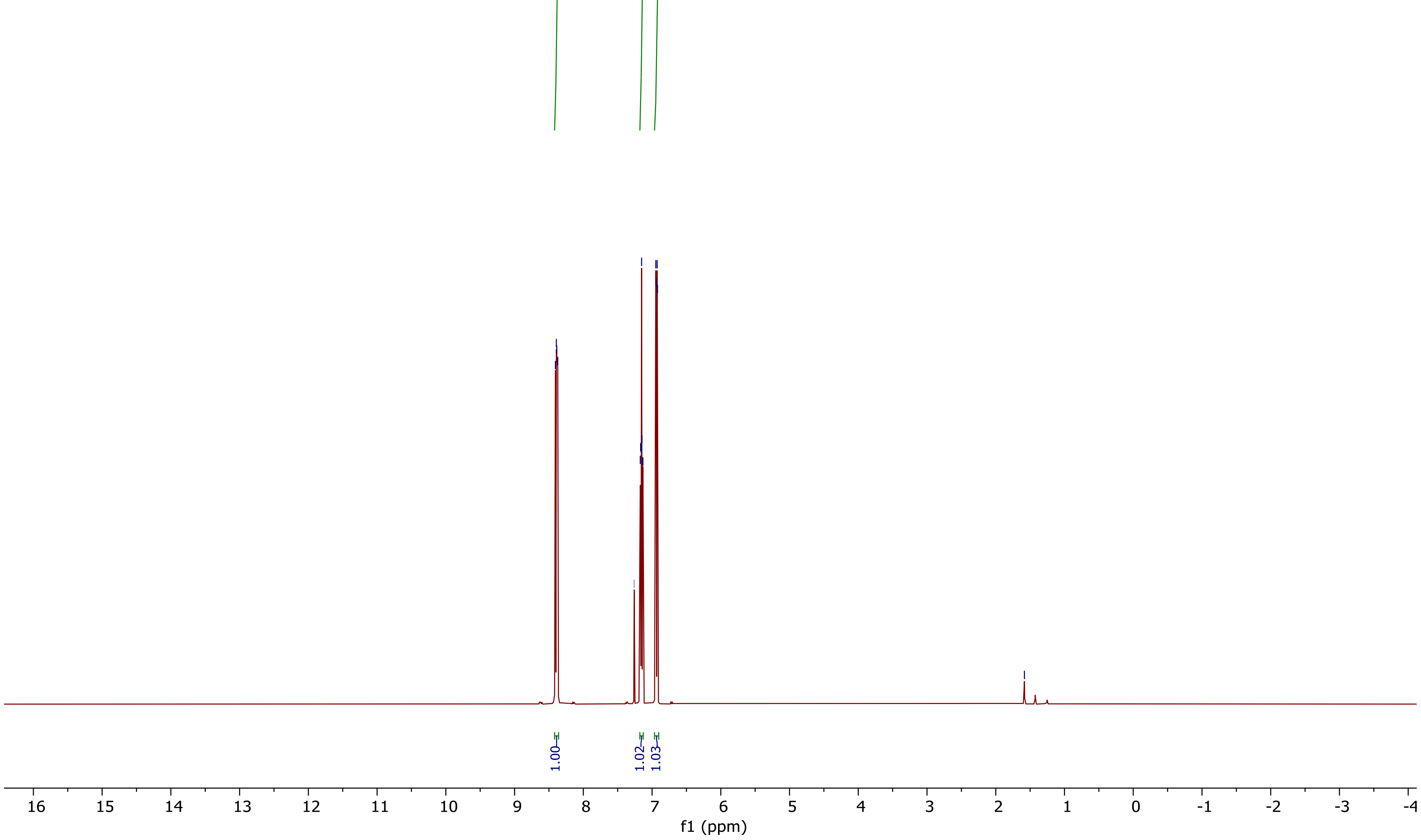

4-Fluoropyridin-2-yl trifluoromethanesulfonate – $^{13}C\{^{1}H\}$ NMR (101 MHz, $CDCl_3$)

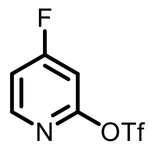

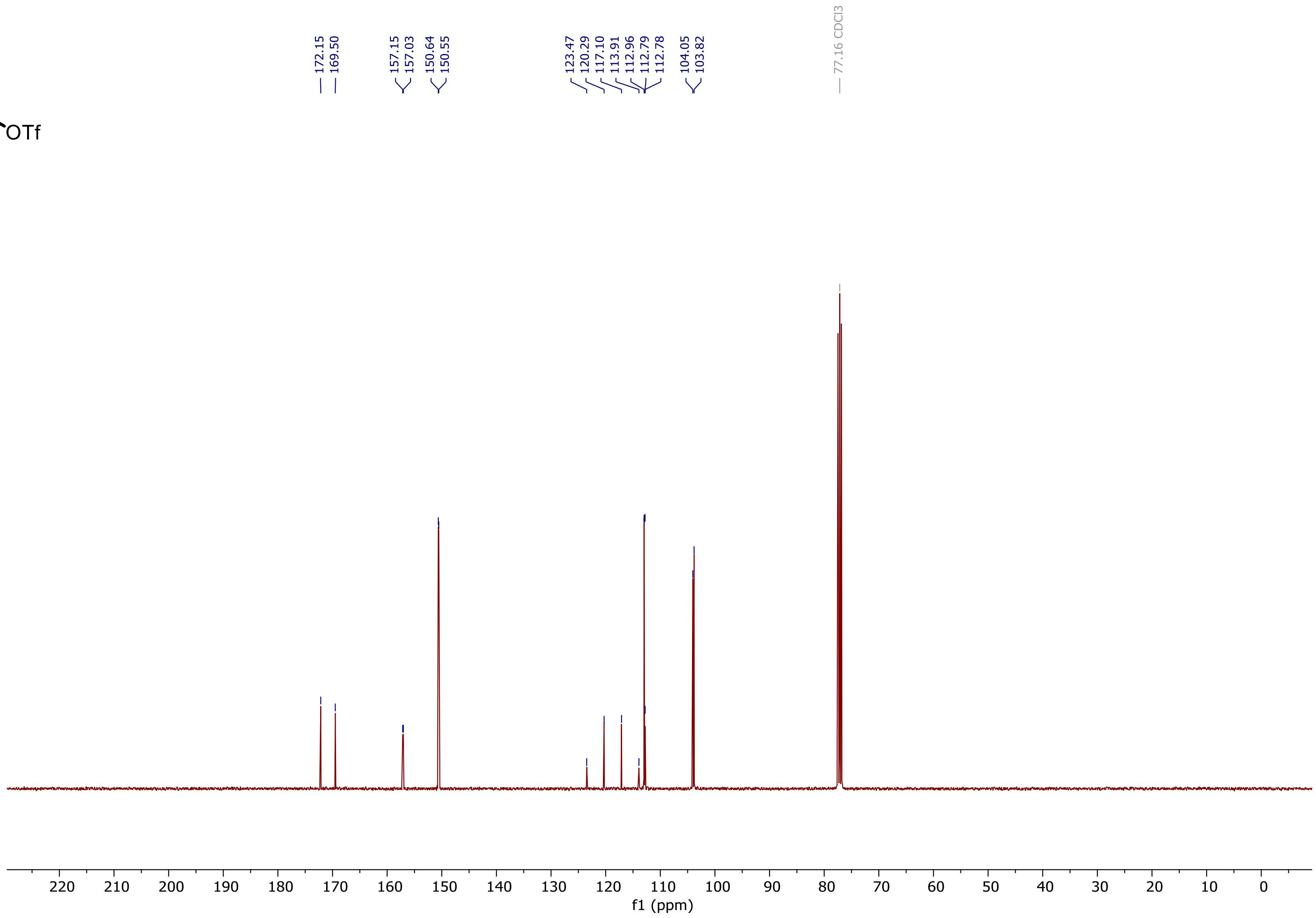

4-Fluoropyridin-2-yl trifluoromethanesulfonate – ^{19}F NMR (377 MHz, $CDCl_3$)

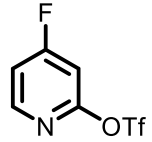

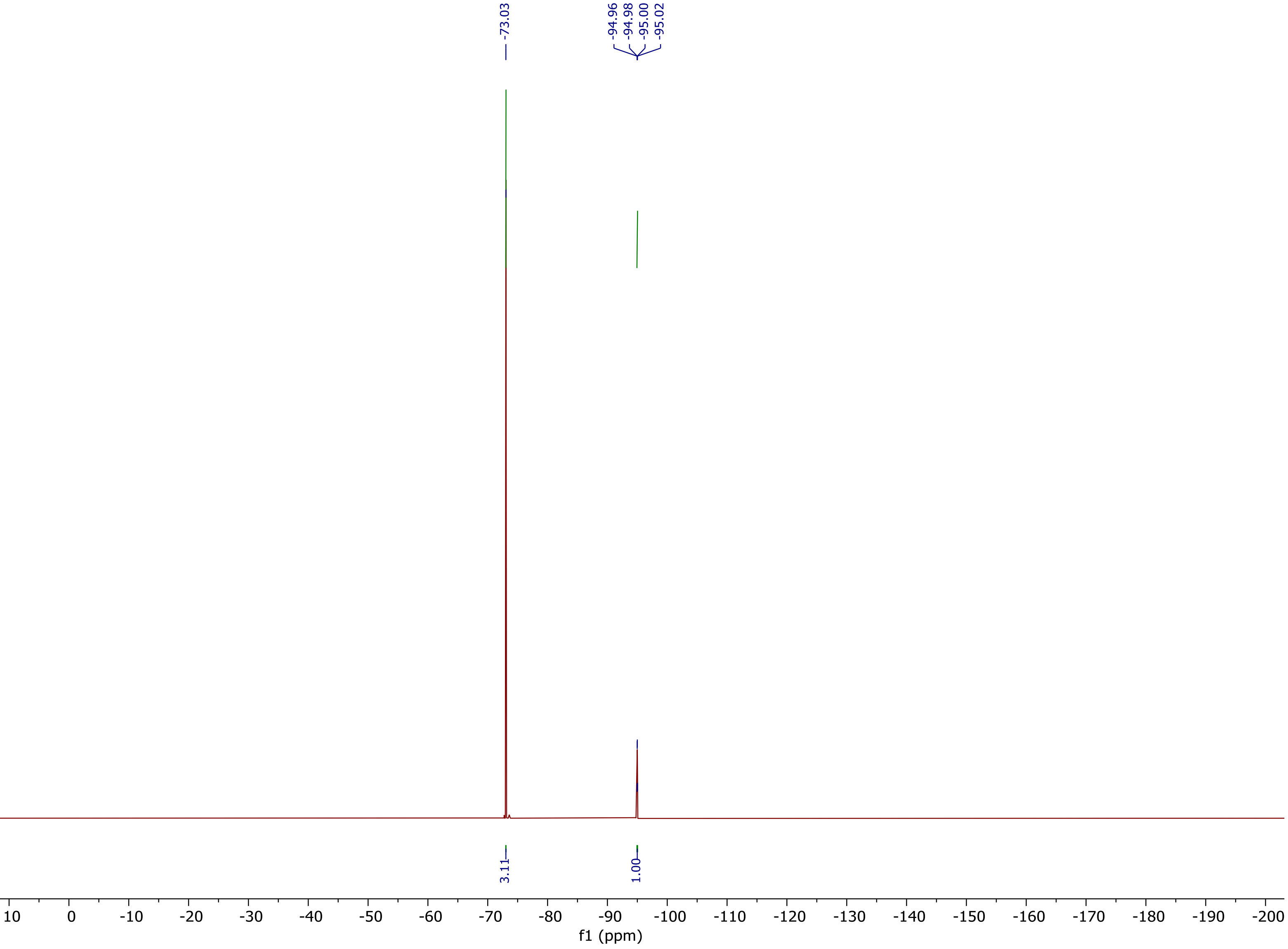

2-Fluoropyridin-4-yl trifluoromethanesulfonate - ^{1}H NMR (400 MHz, $CDCl_3$)

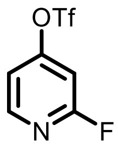

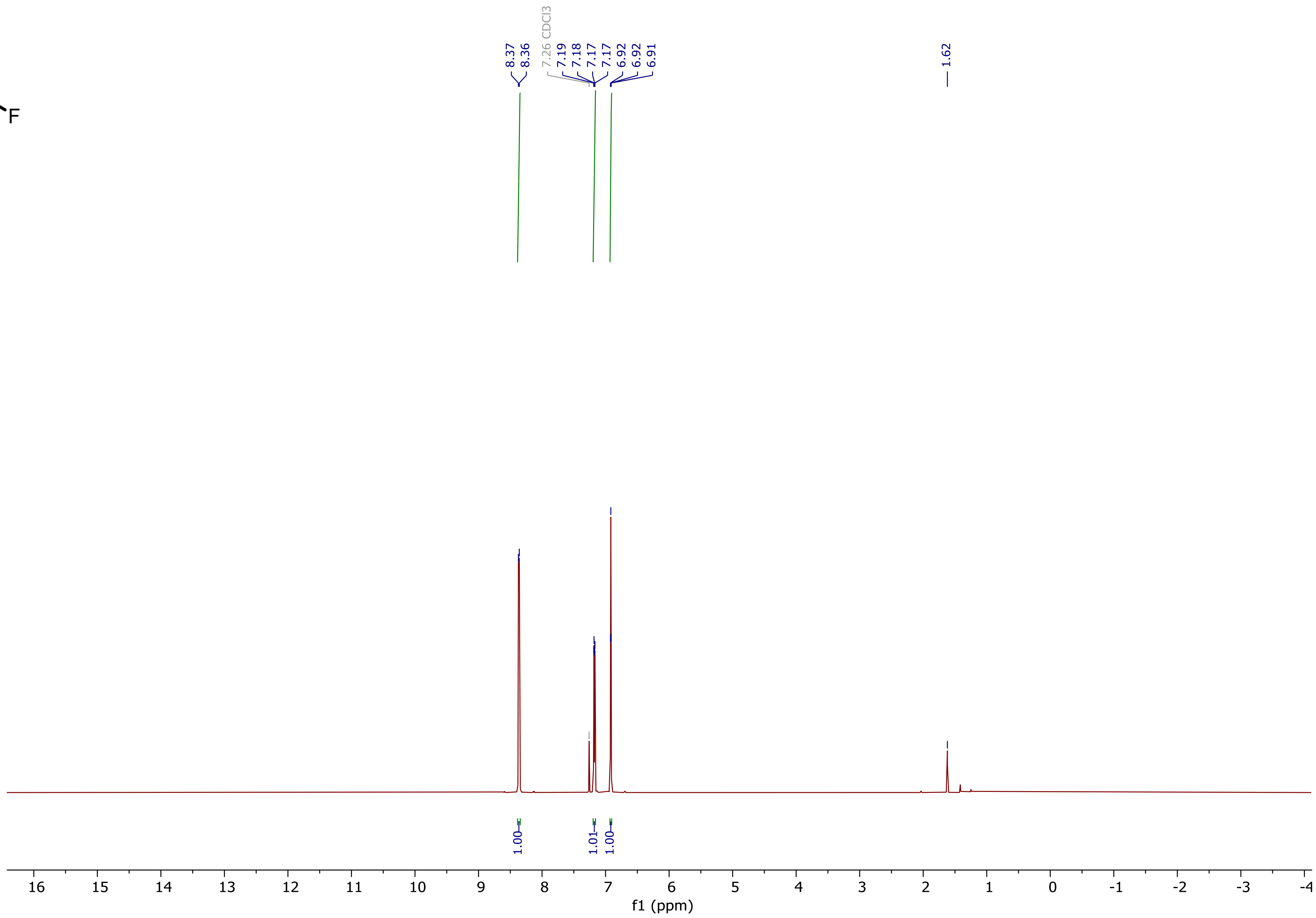

2-Fluoropyridin-4-yl trifluoromethanesulfonate - $^{13}C\{^1H\}$ NMR (101 MHz, $CDCl_3$)

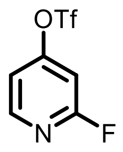

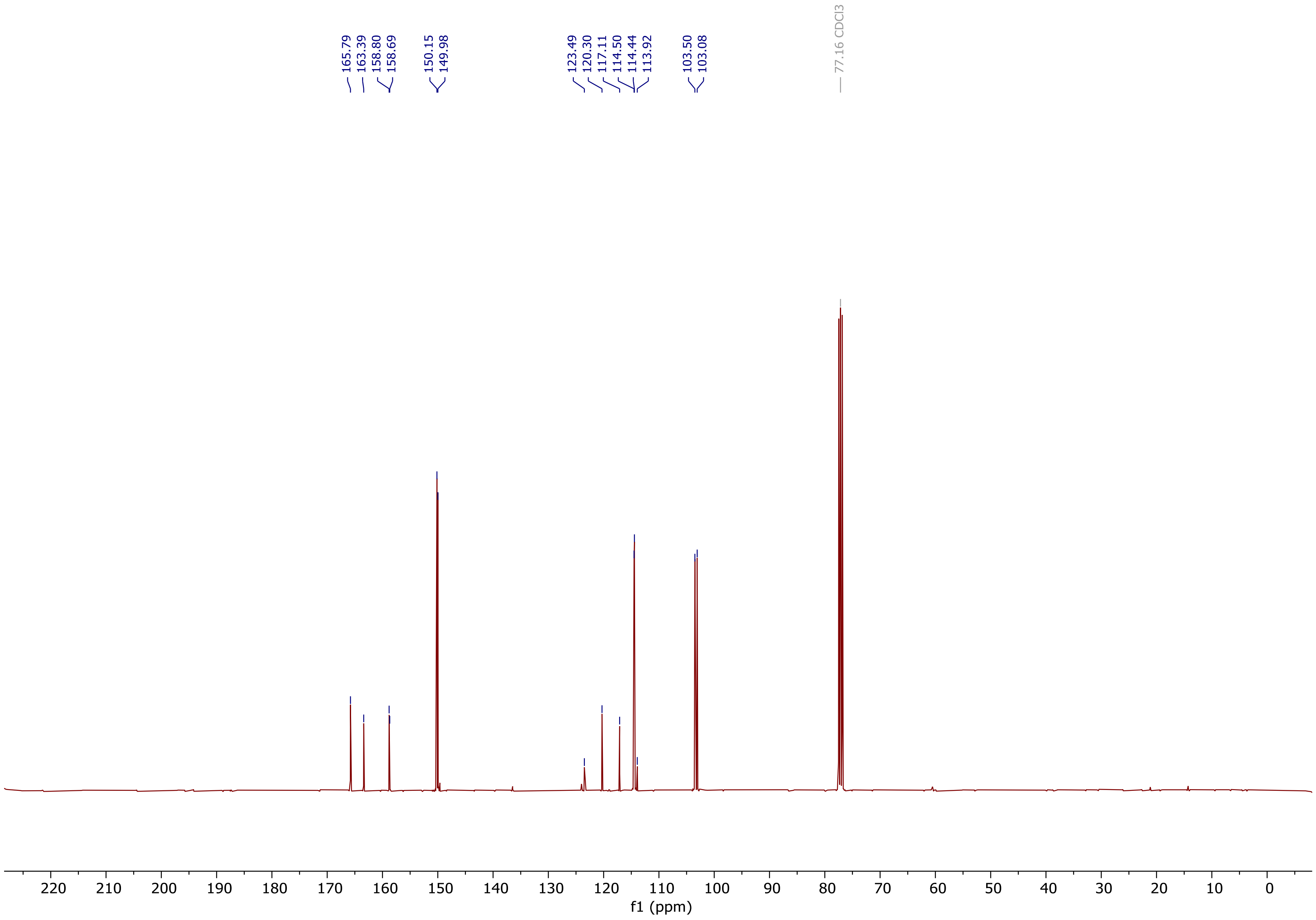

2-Fluoropyridin-4-yl trifluoromethanesulfonate - ^{19}F NMR (377 MHz, $CDCl_3$)

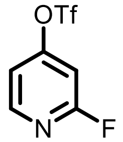

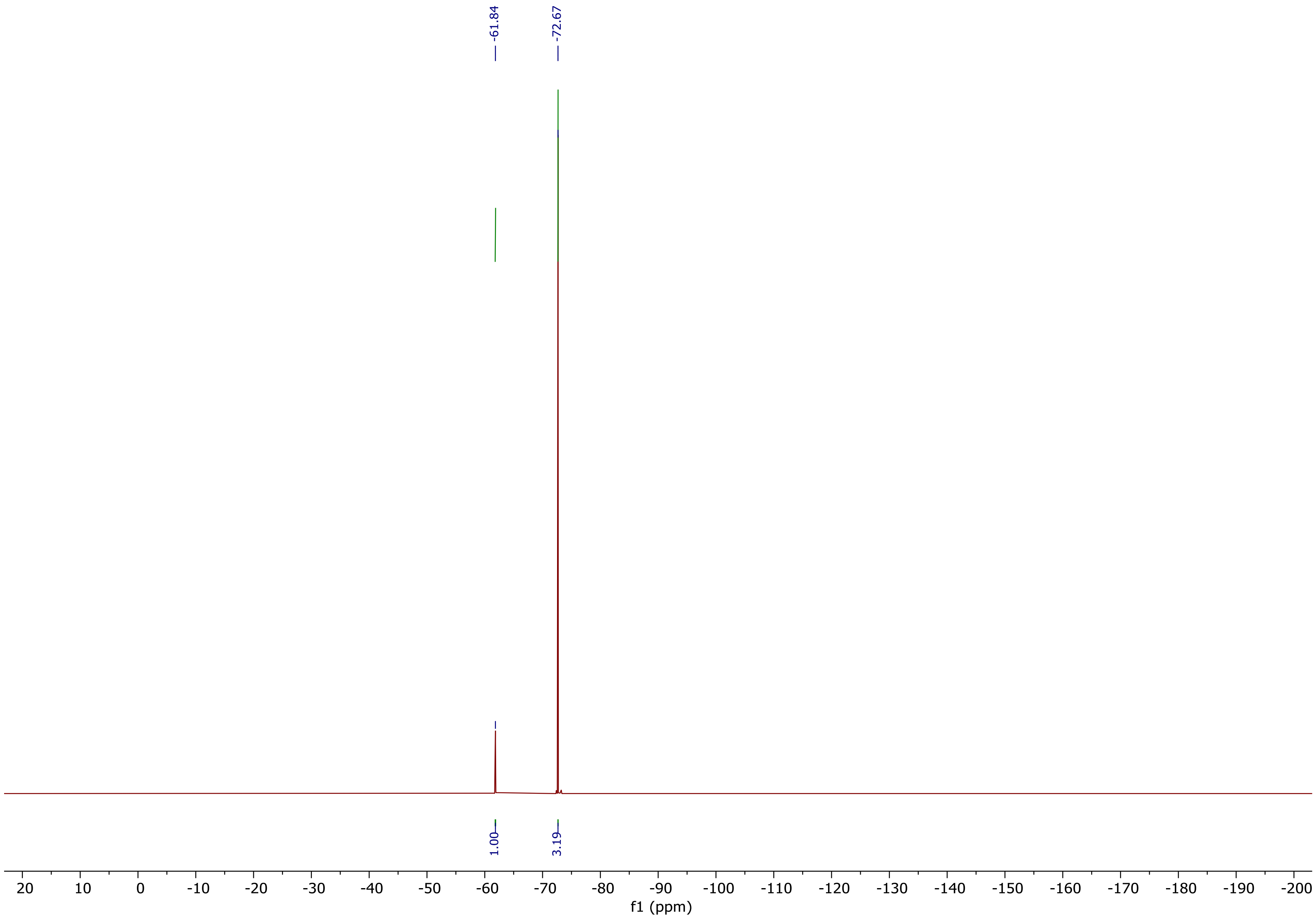

Methyl (*R*)-2-((*tert*-butoxycarbonyl)amino)-3-(4-(((trifluoromethyl)sulfonyl)oxy)phenyl) propanoate - ^{1}H NMR (400 MHz, $CDCl_3$)

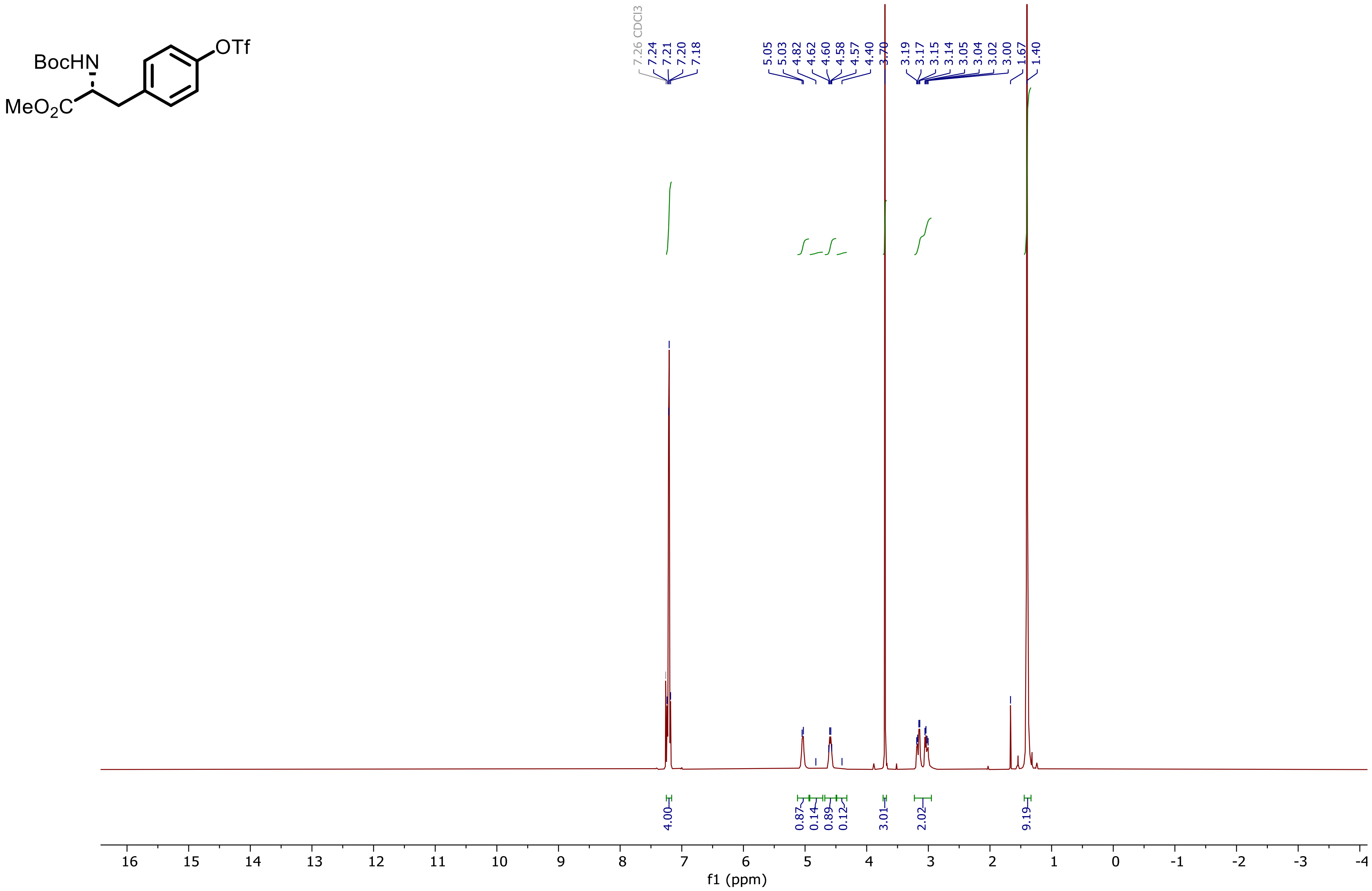

Methyl (*R*)-2-((*tert*-butoxycarbonyl)amino)-3-(4-(((trifluoromethyl)sulfonyl)oxy)phenyl) propanoate - $^{13}C\{^1H\}$ NMR (101 MHz, $CDCl_3$)

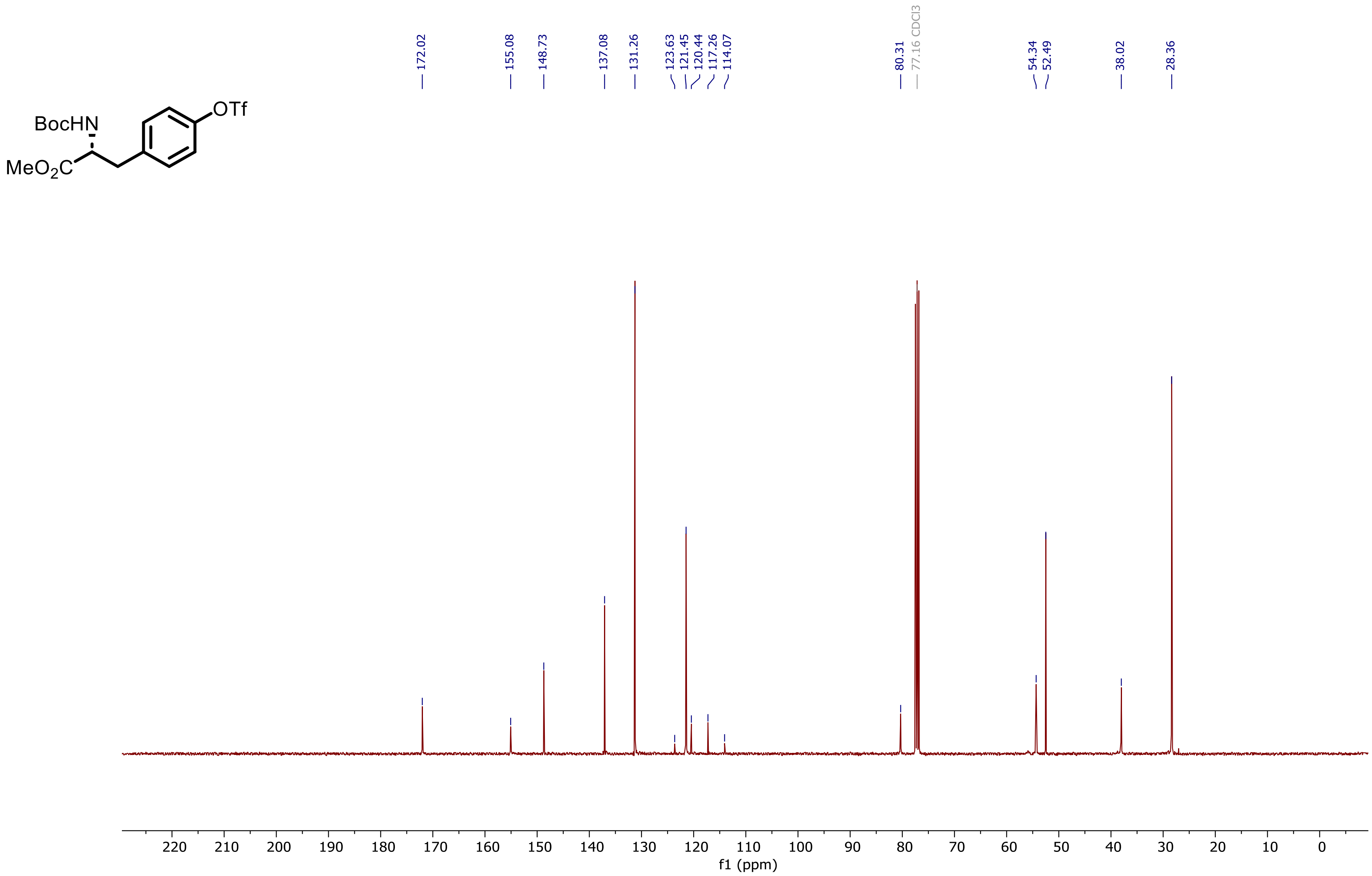

Methyl (*R*)-2-((*tert*-butoxycarbonyl)amino)-3-(4-(((trifluoromethyl)sulfonyl)oxy)phenyl) propanoate - ^{19}F NMR (376 MHz, $CDCl_3$)

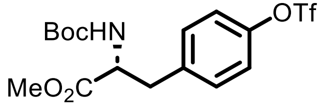

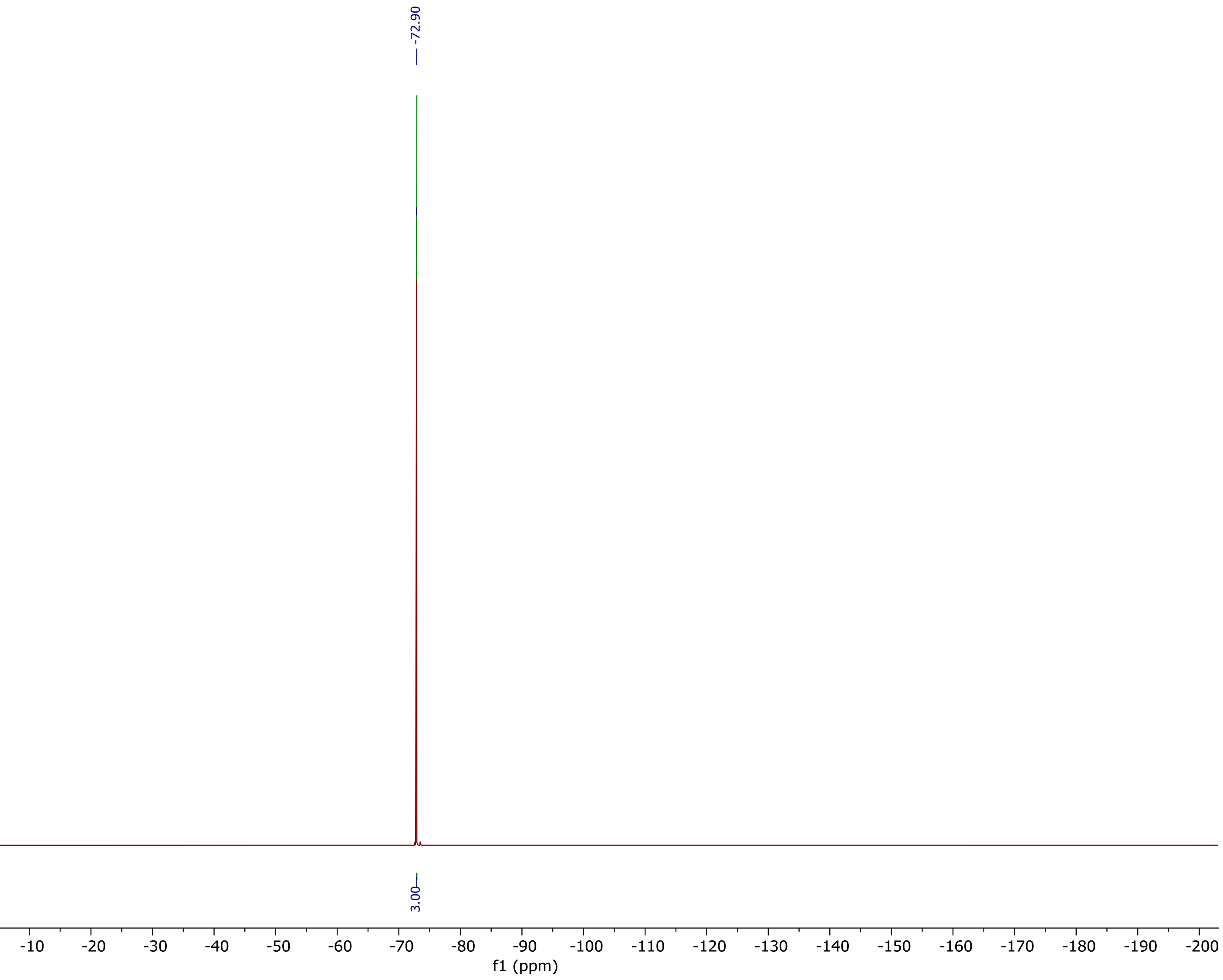

4-Methyl-2-oxo-2*H*-chromen-7-yl trifluoromethanesulfonate - ^{1}H NMR (400 MHz, $CDCl_3$)

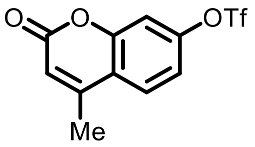

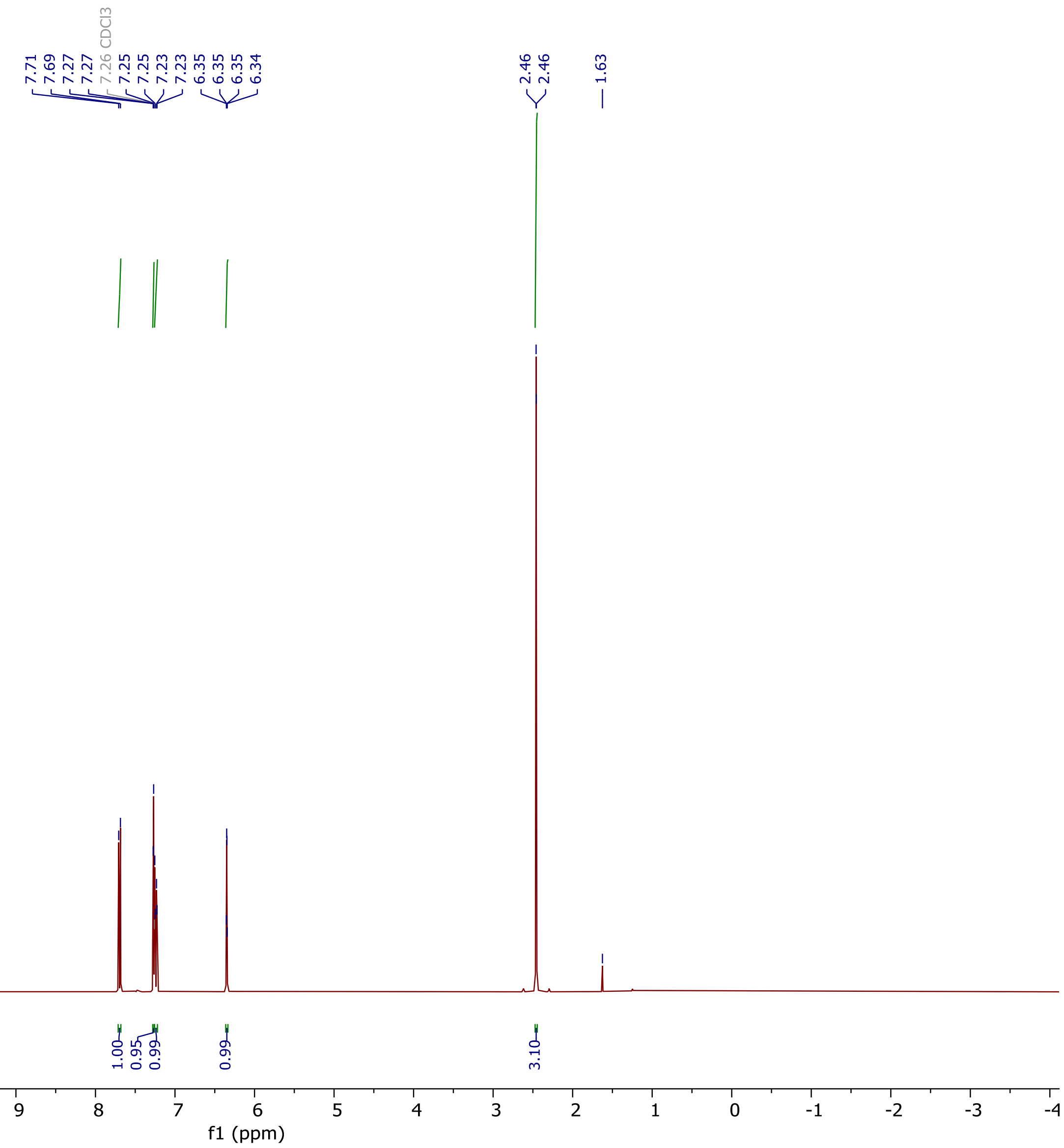

4-Methyl-2-oxo-2*H*-chromen-7-yl trifluoromethanesulfonate - $^{13}C\{^1H\}$ NMR (101 MHz, $CDCl_3$)

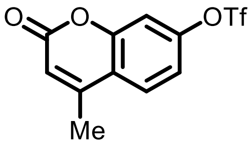

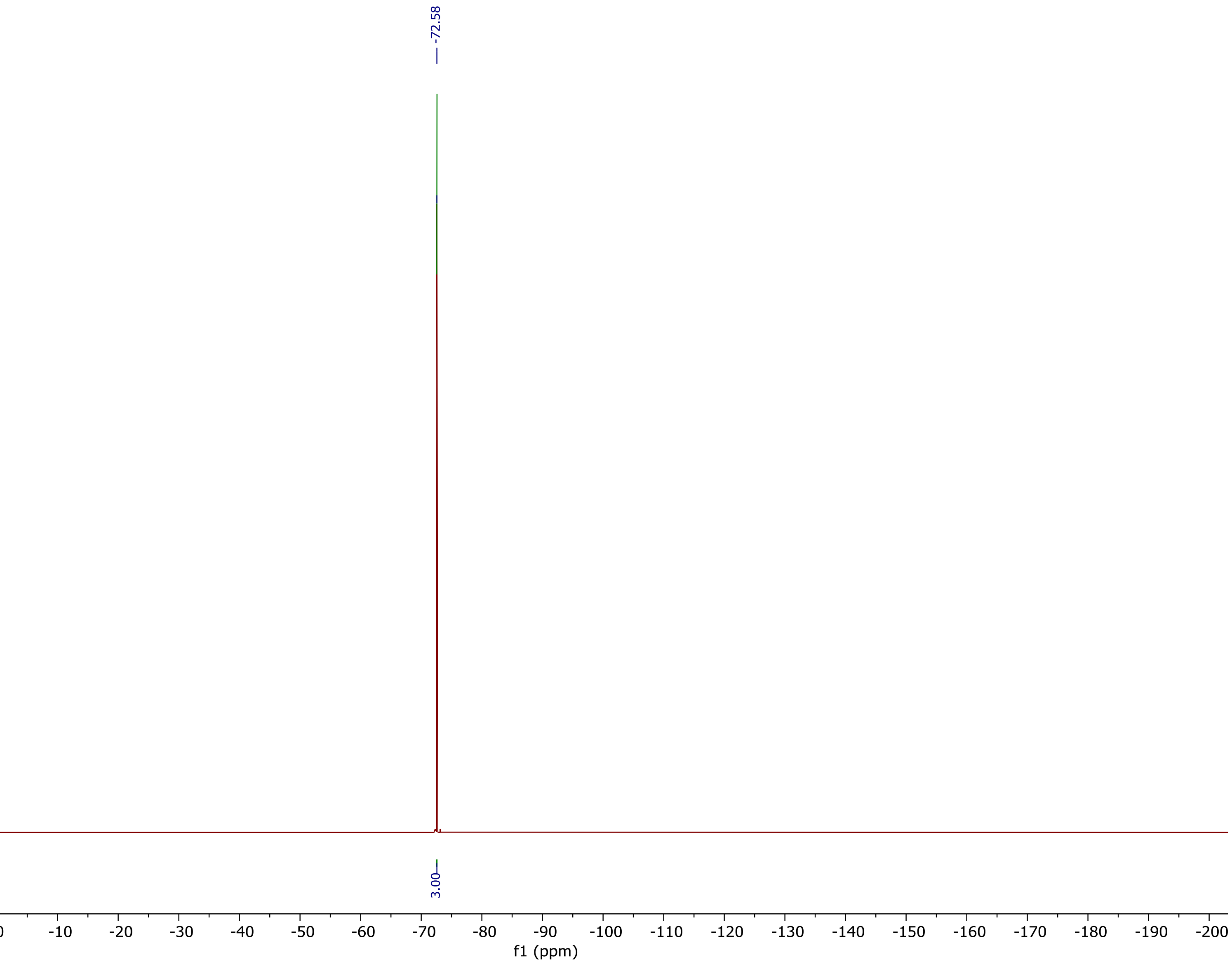

2-Fluoropyridin-3-yl trifluoromethanesulfonate - ^{1}H NMR (400 MHz, $CDCl_3$)

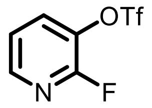

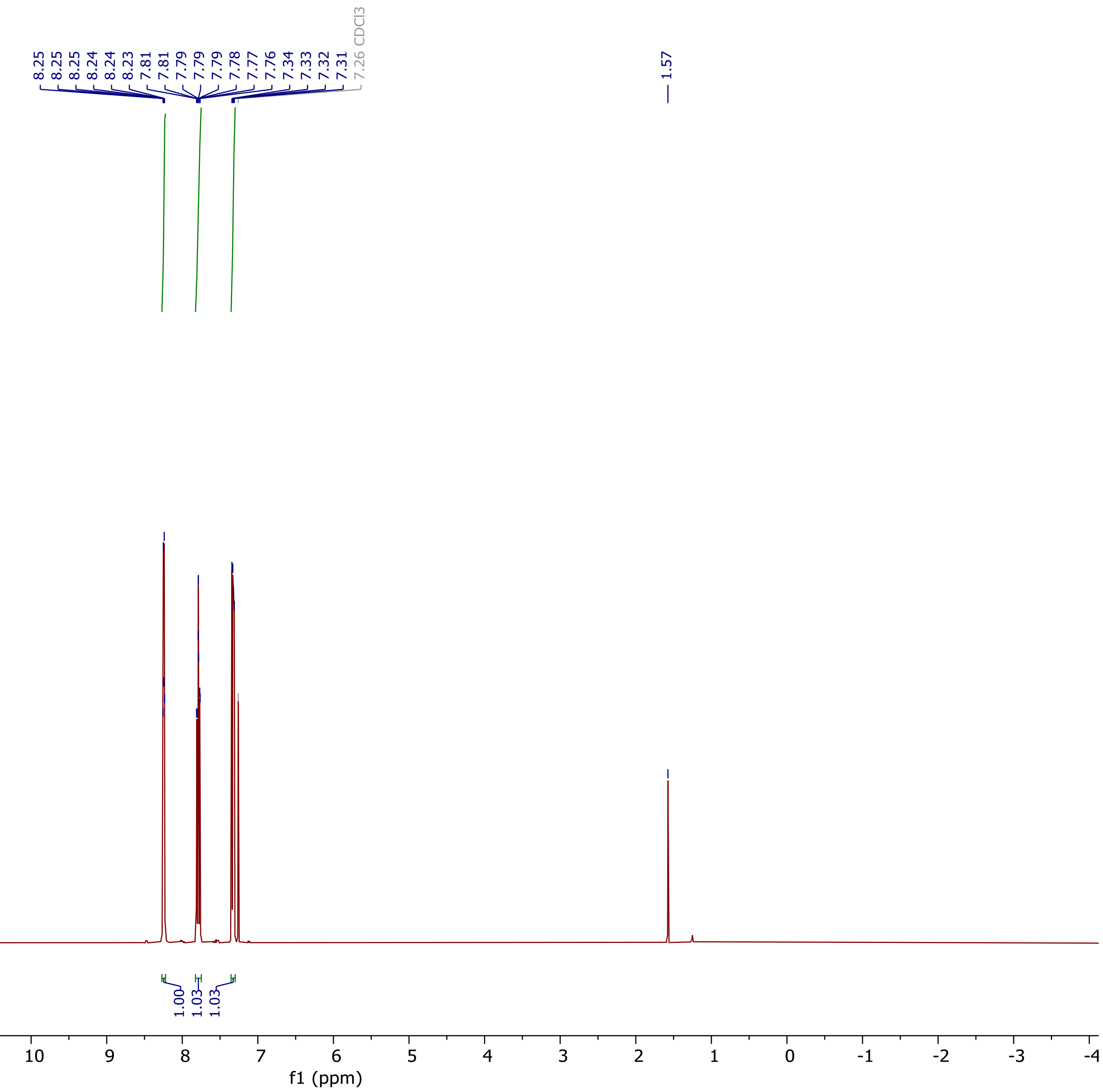

2-Fluoropyridin-3-yl trifluoromethanesulfonate - $^{13}C\{^{1}H\}$ NMR (101 MHz, $CDCl_3$)

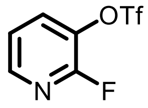

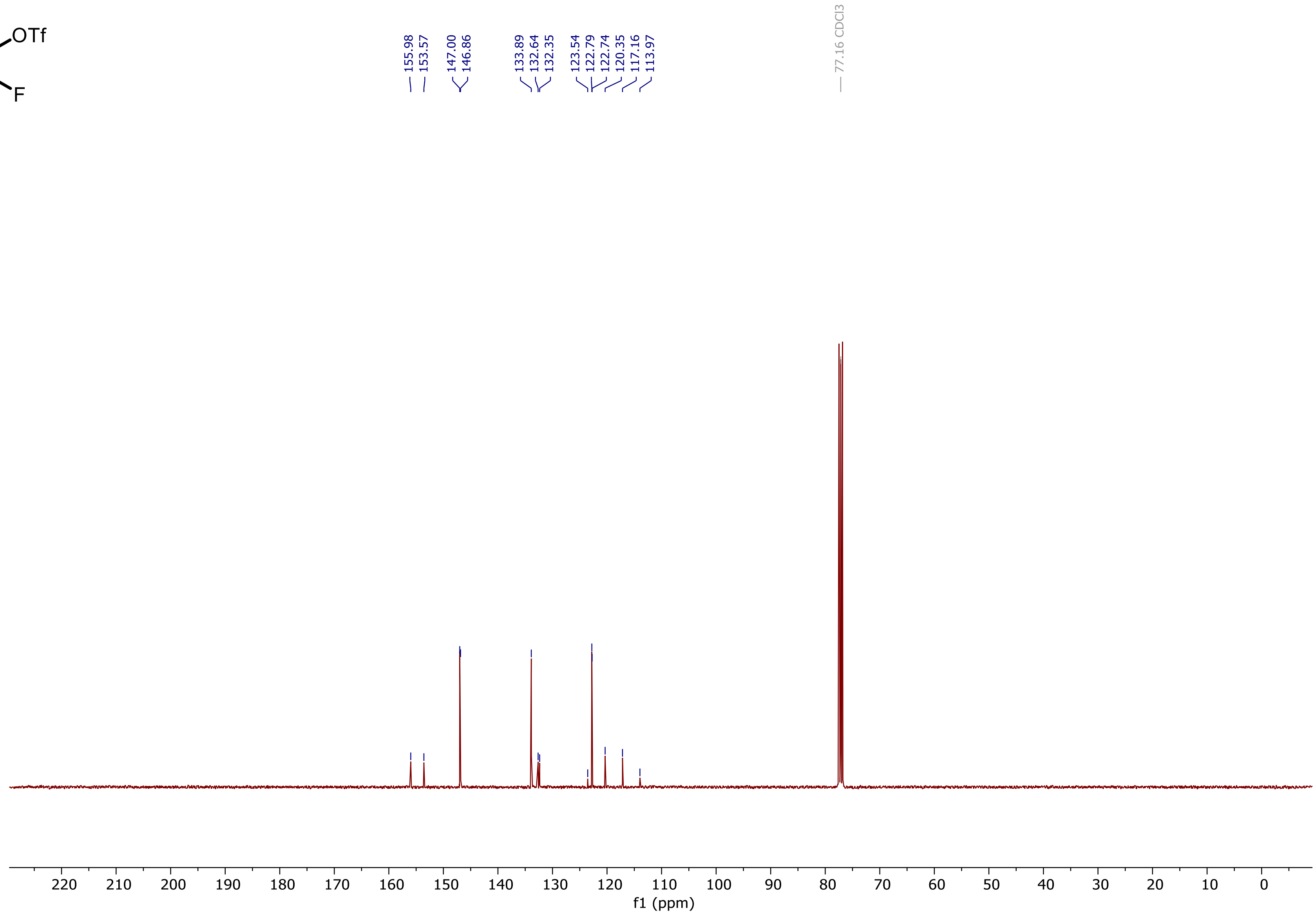

2-Fluoropyridin-3-yl trifluoromethanesulfonate - ^{19}F NMR (376 MHz, $CDCl_3$)

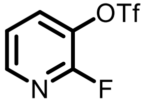

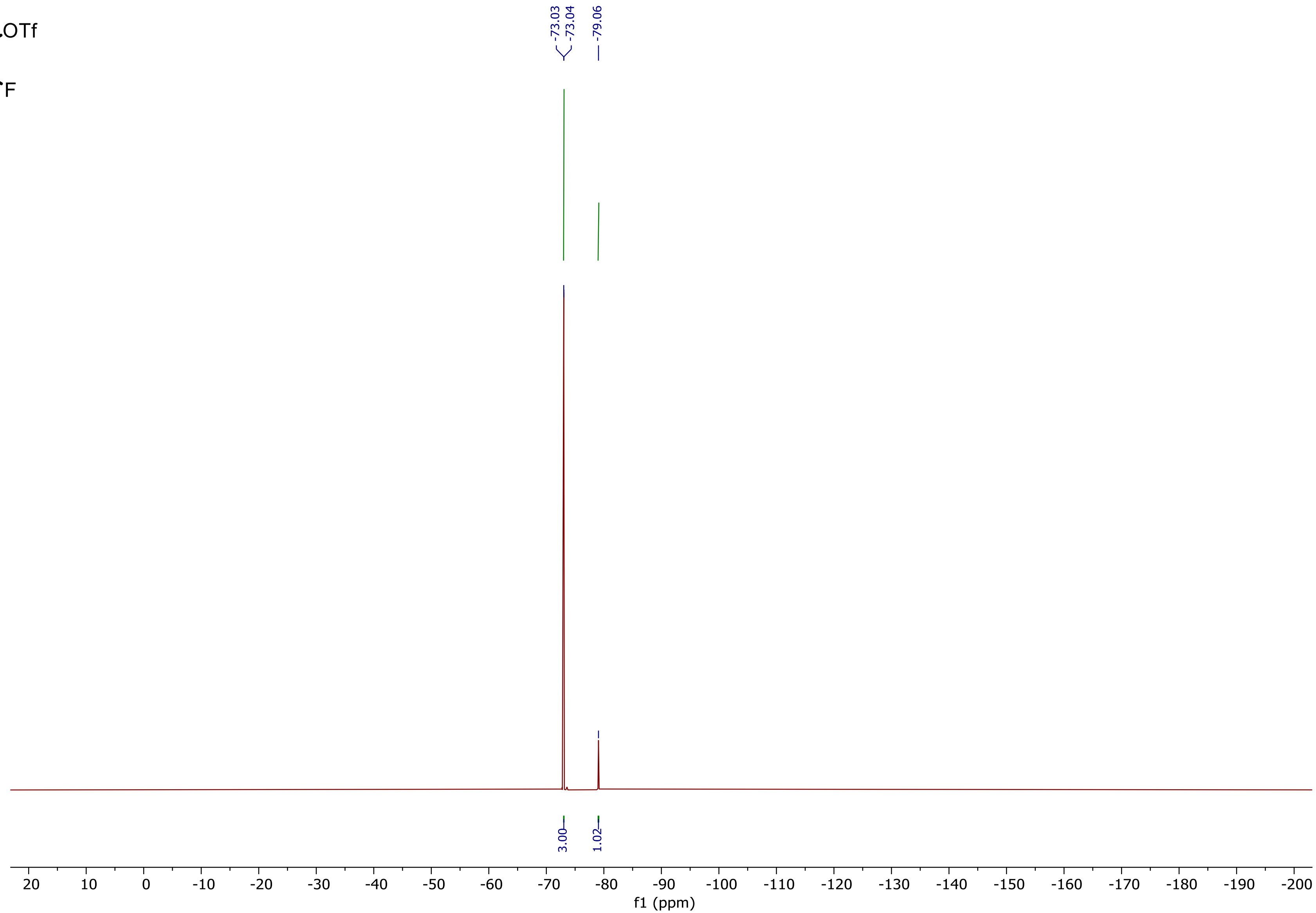

5-Fluoropyridin-2-yl trifluoromethanesulfonate - ^{1}H NMR (400 MHz, $CDCl_3$)

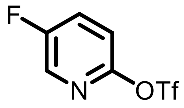

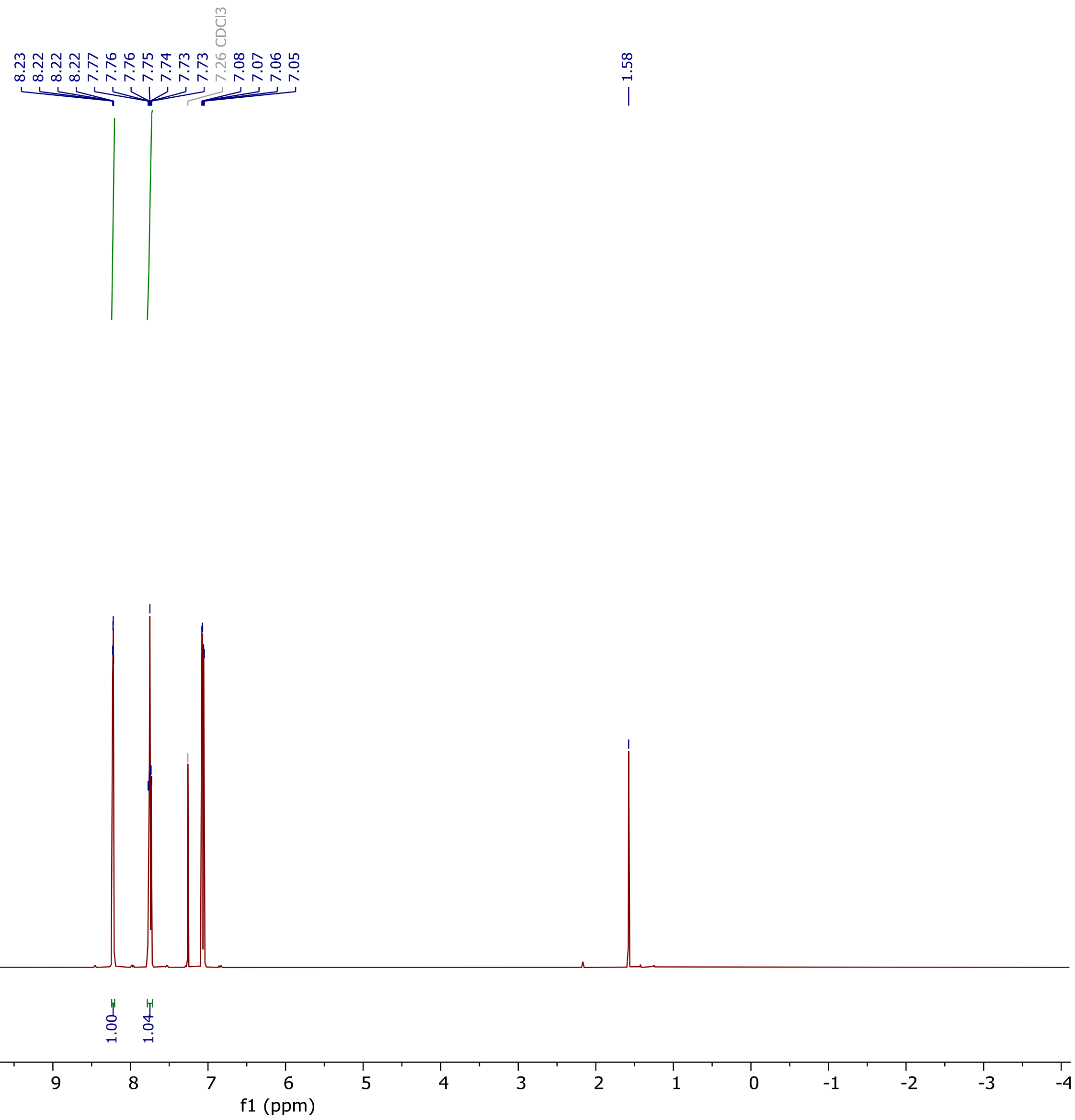

5-Fluoropyridin-2-yl trifluoromethanesulfonate - $^{13}C\{^1H\}$ NMR (101 MHz, $CDCl_3$)

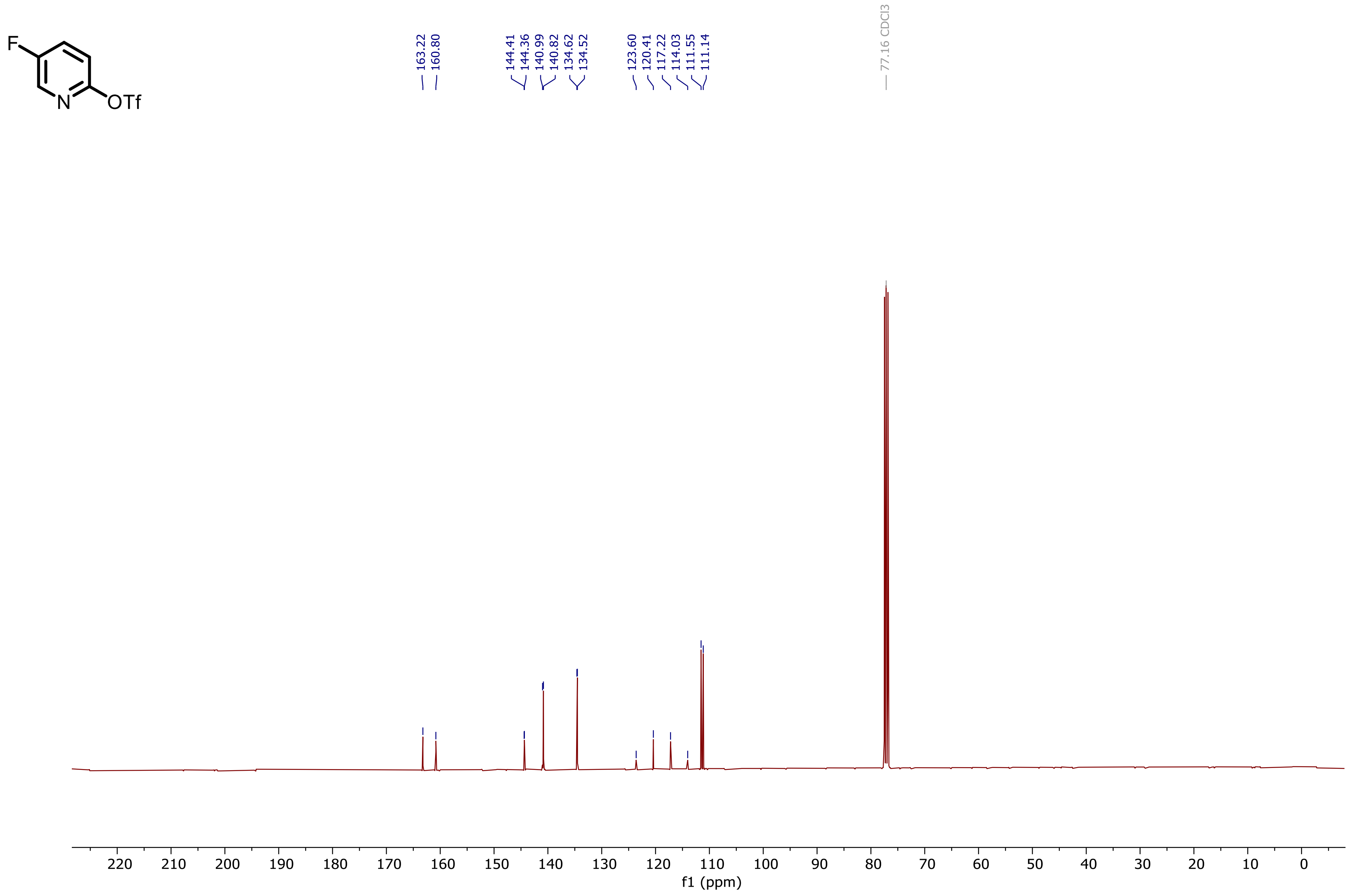

5-Fluoropyridin-2-yl trifluoromethanesulfonate - ^{19}F NMR (377 MHz, $CDCl_3$)

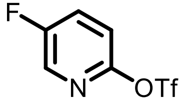

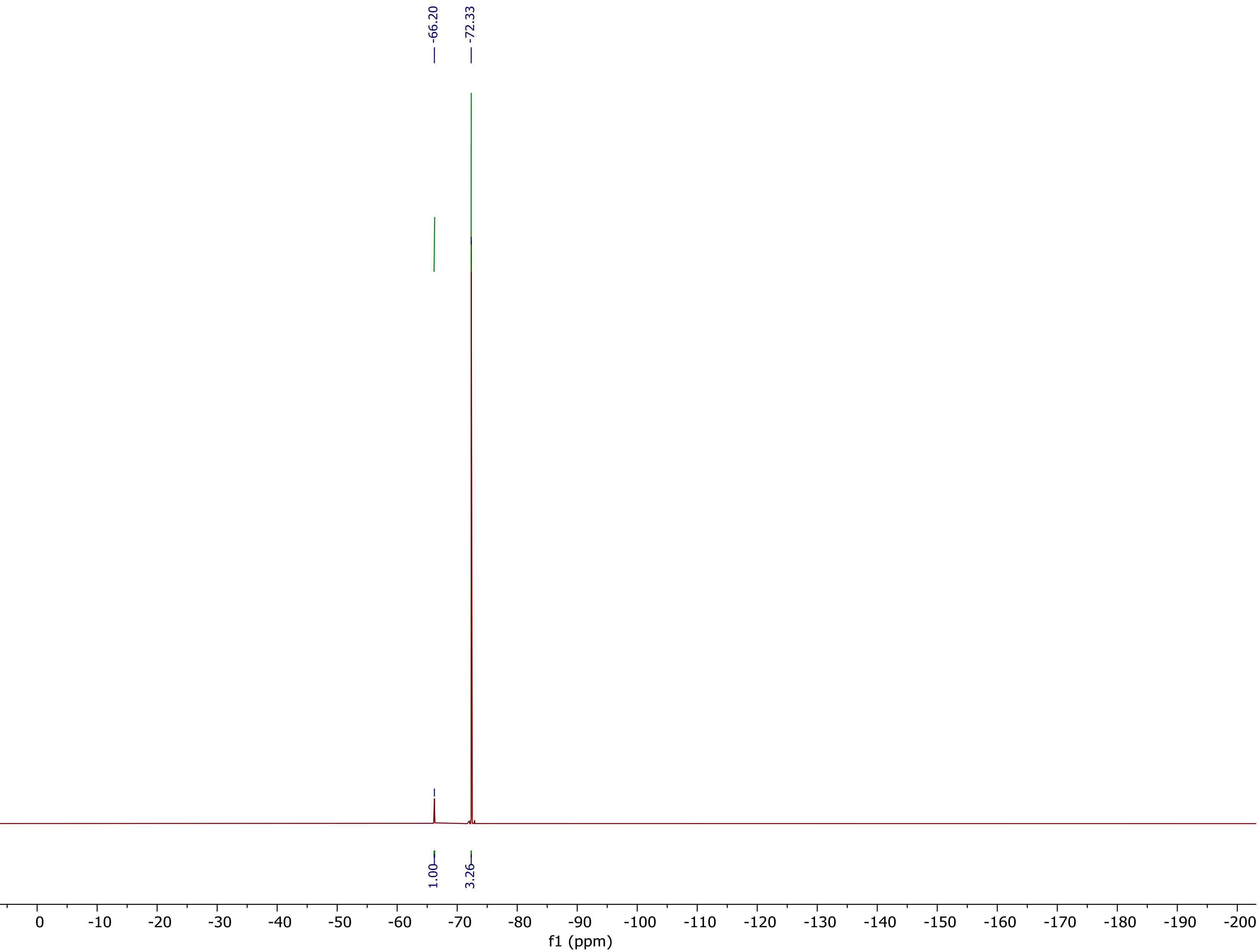

4-Chlorophenyl trifluoromethanesulfonate - ^{1}H NMR (400 MHz, $CDCl_3$)

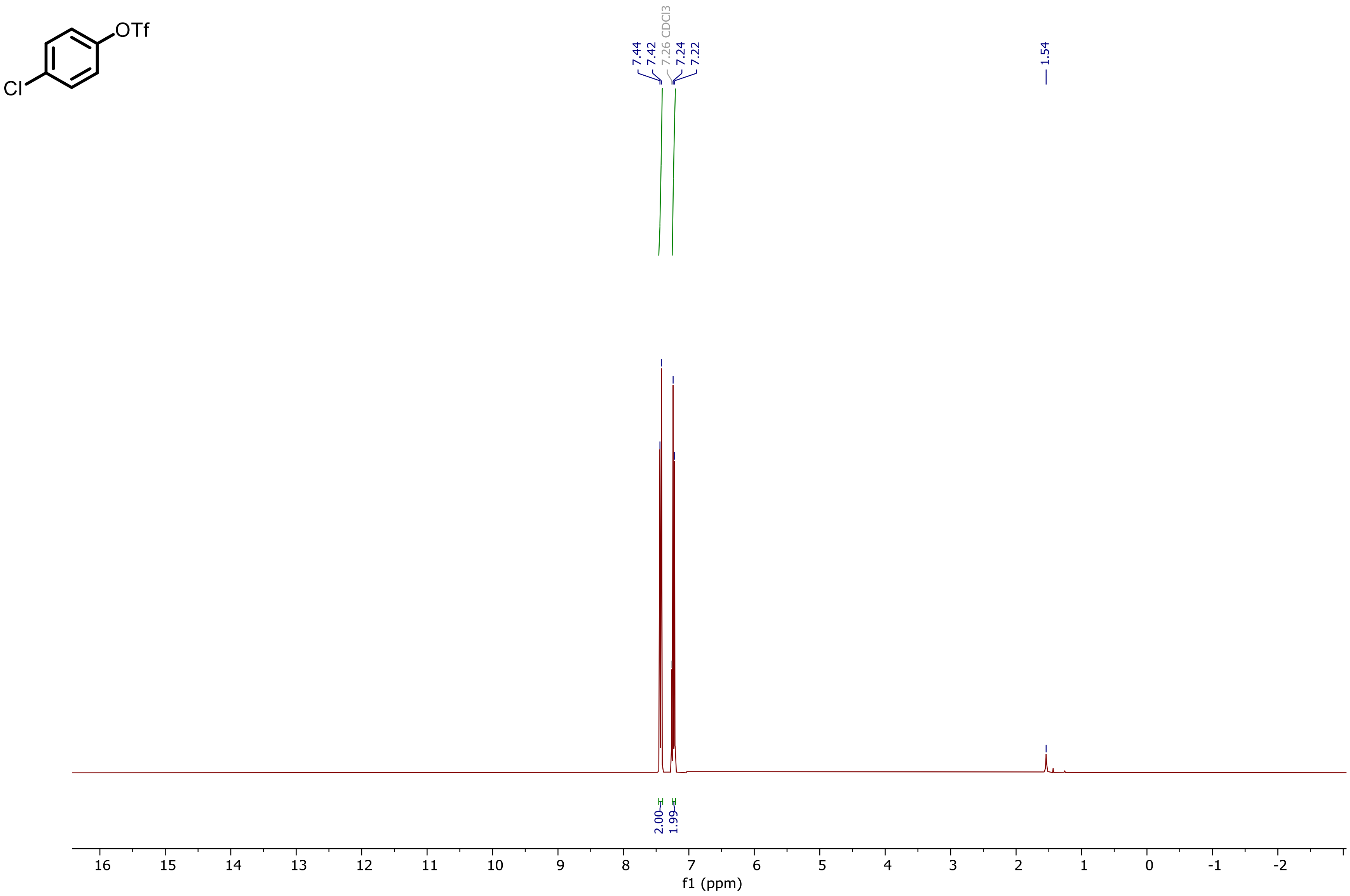

4-Chlorophenyl trifluoromethanesulfonate - $^{13}C\{^1H\}$ NMR (101 MHz, $CDCl_3$)

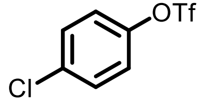

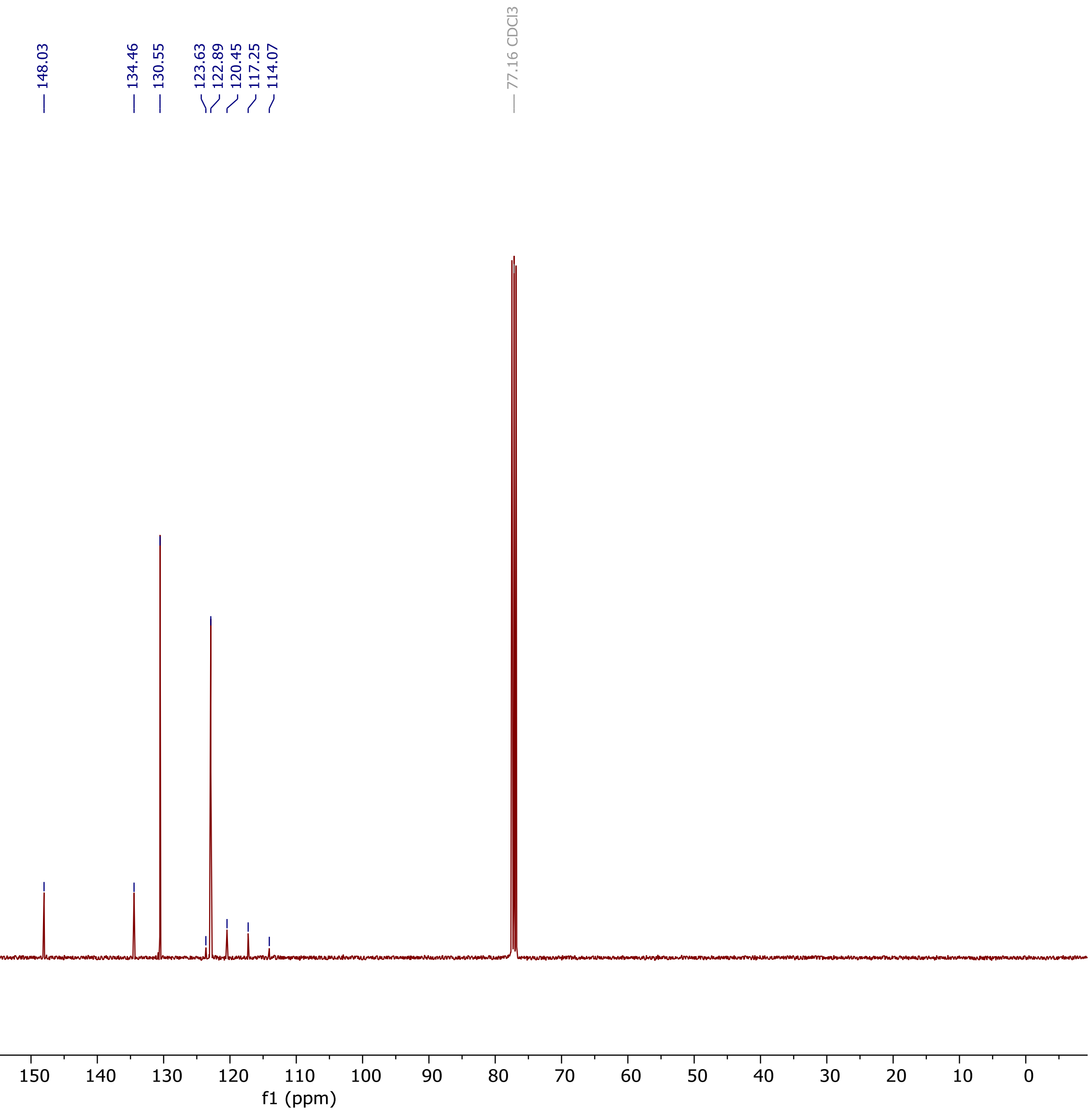

4-Chlorophenyl trifluoromethanesulfonate - ^{19}F NMR (376 MHz, $CDCl_3$)

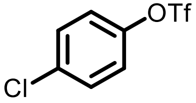

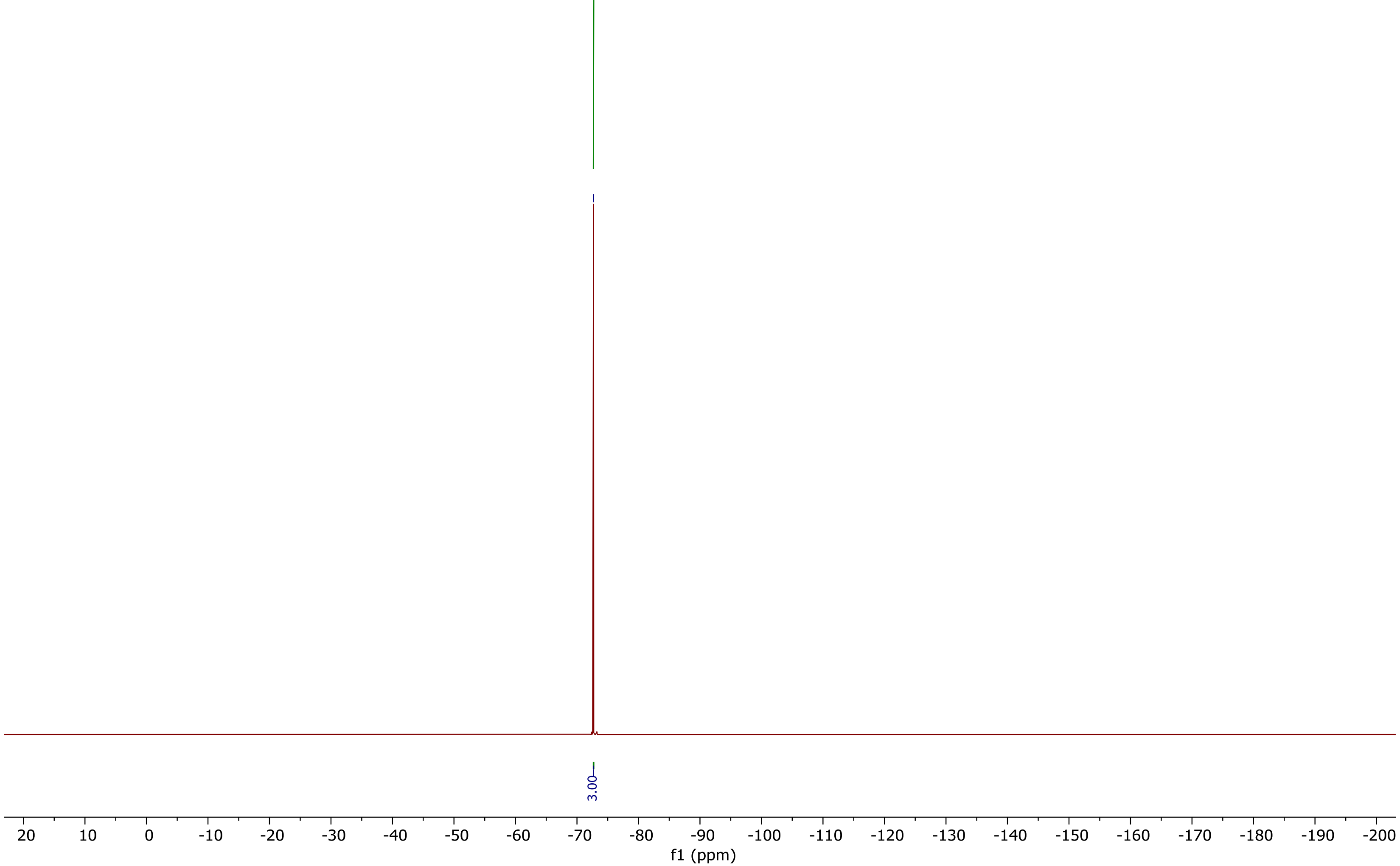

2-Allylphenyl trifluoromethanesulfonate - ^{1}H NMR (400 MHz, $CDCl_3$)

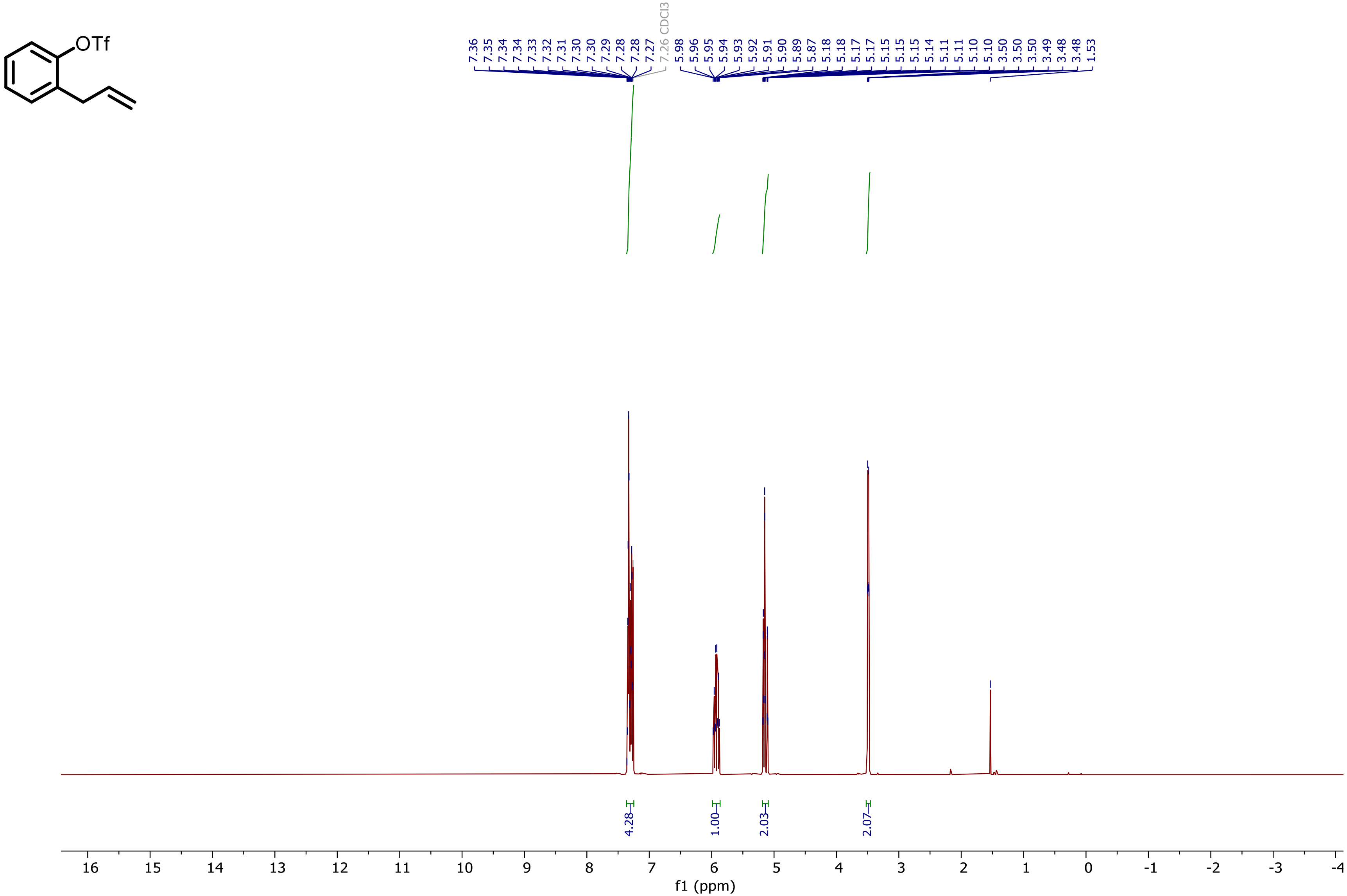

2-Allylphenyl trifluoromethanesulfonate - $^{13}C\{^{1}H\}$ NMR (101 MHz, $CDCl_3$)

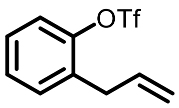

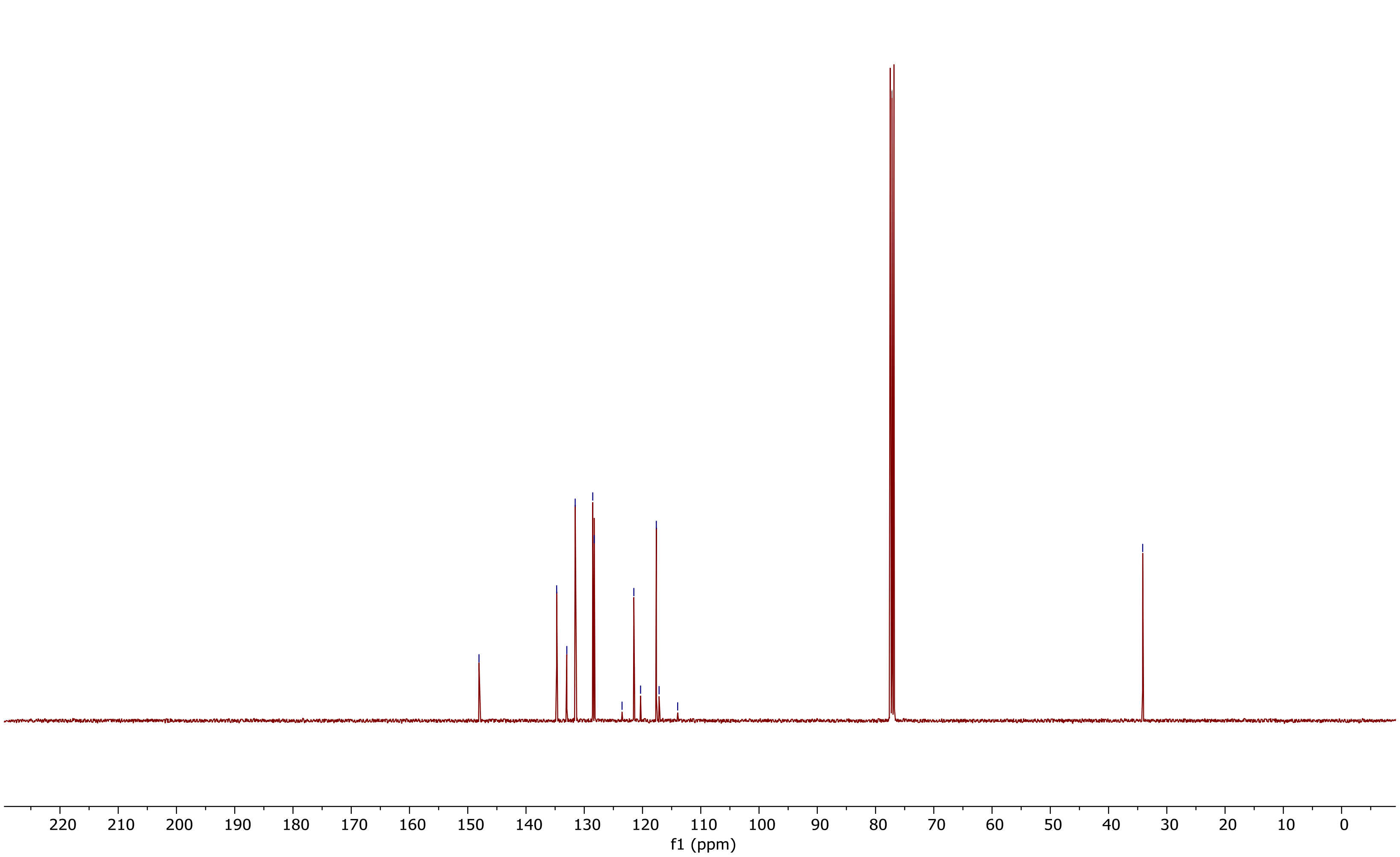

2-Allylphenyl trifluoromethanesulfonate - ^{19}F NMR (376 MHz, $CDCl_3$)

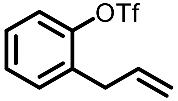

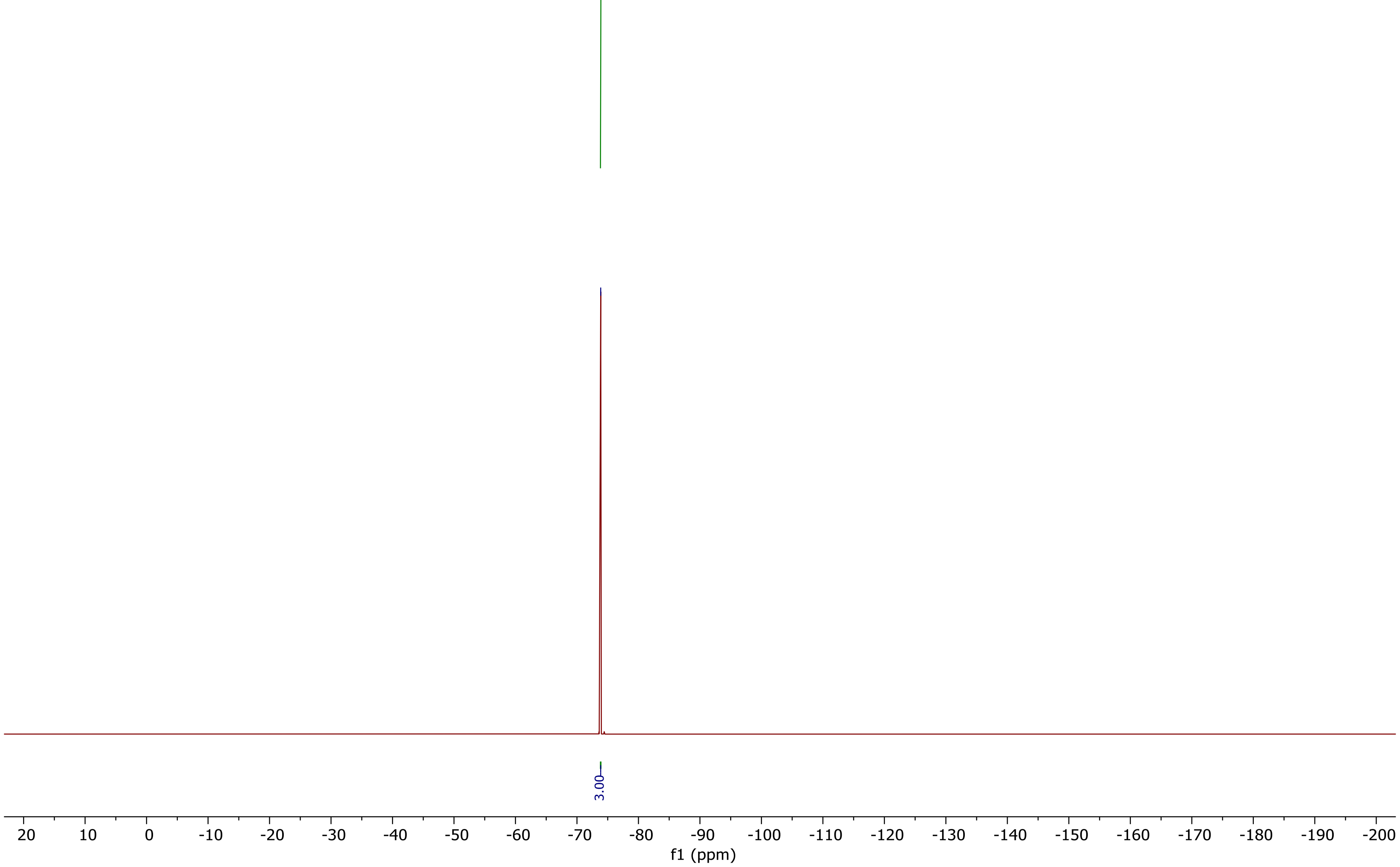

4-Cyano-2,6-dimethylphenyl trifluoromethanesulfonate - ^{1}H NMR (400 MHz, $CDCl_3$)

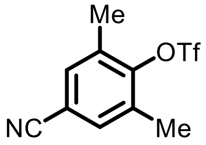

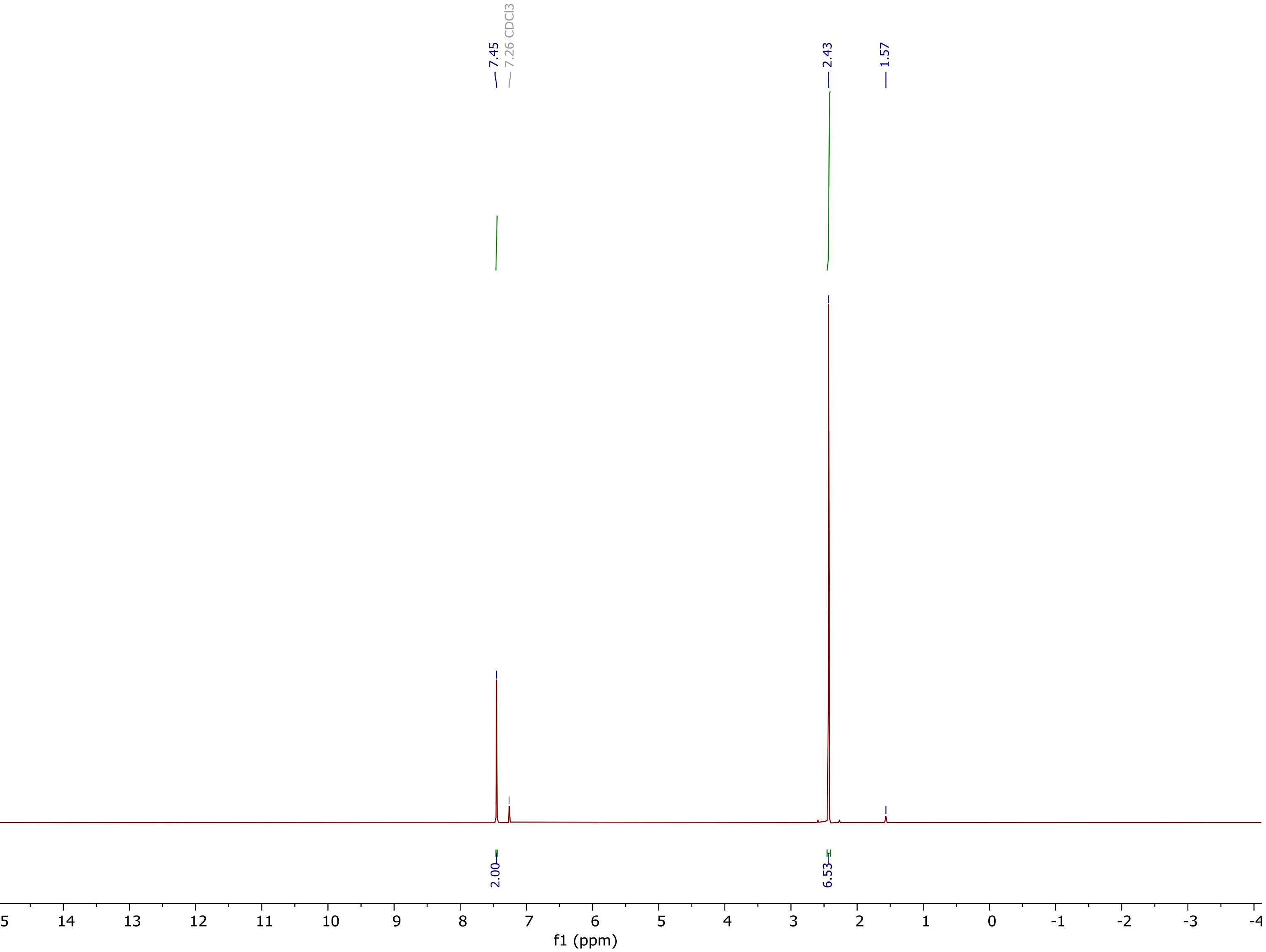

4-Cyano-2,6-dimethylphenyl trifluoromethanesulfonate - $^{13}C\{^1H\}$ NMR (126 MHz, $CDCl_3$)

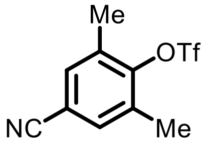

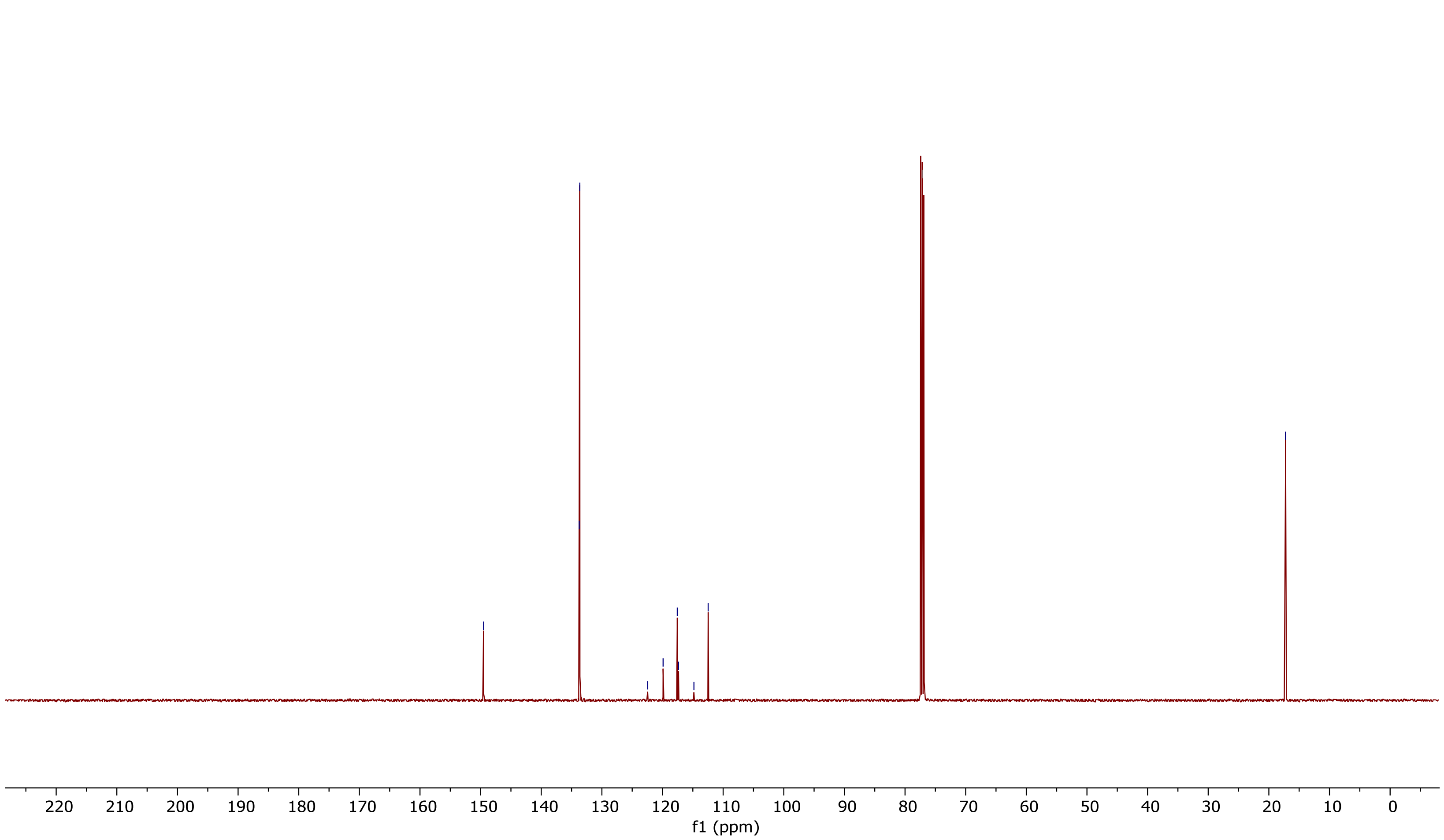

4-Cyano-2,6-dimethylphenyl trifluoromethanesulfonate - ^{19}F NMR (376 MHz, $CDCl_3$)

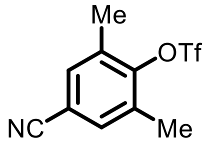

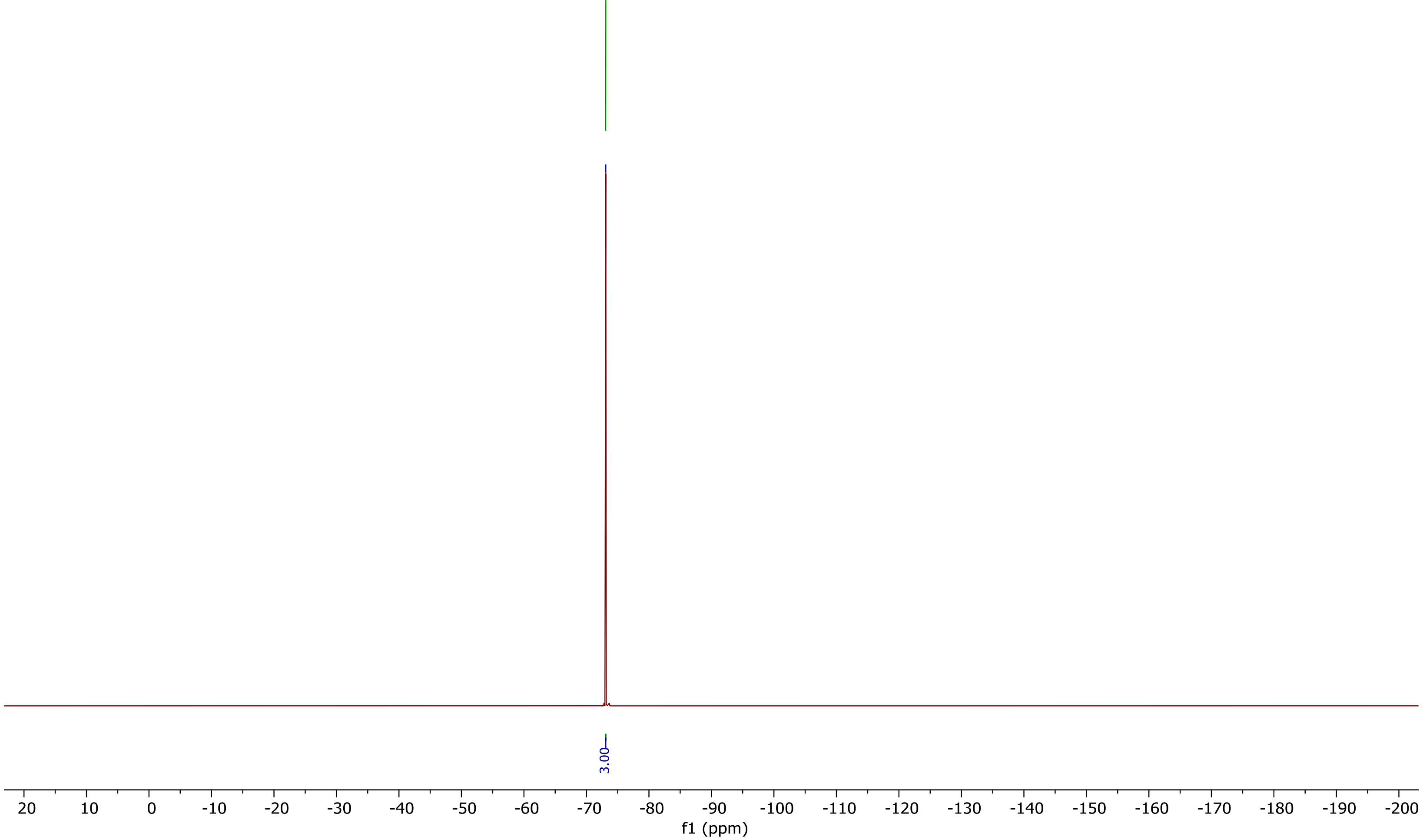

Ethyl (*S*)-6-hydroxy-2,5,7,8-tetramethylchromane-2-carboxylate - ^{1}H NMR (400 MHz, $CDCl_3$)

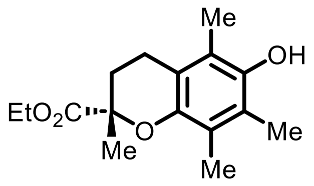

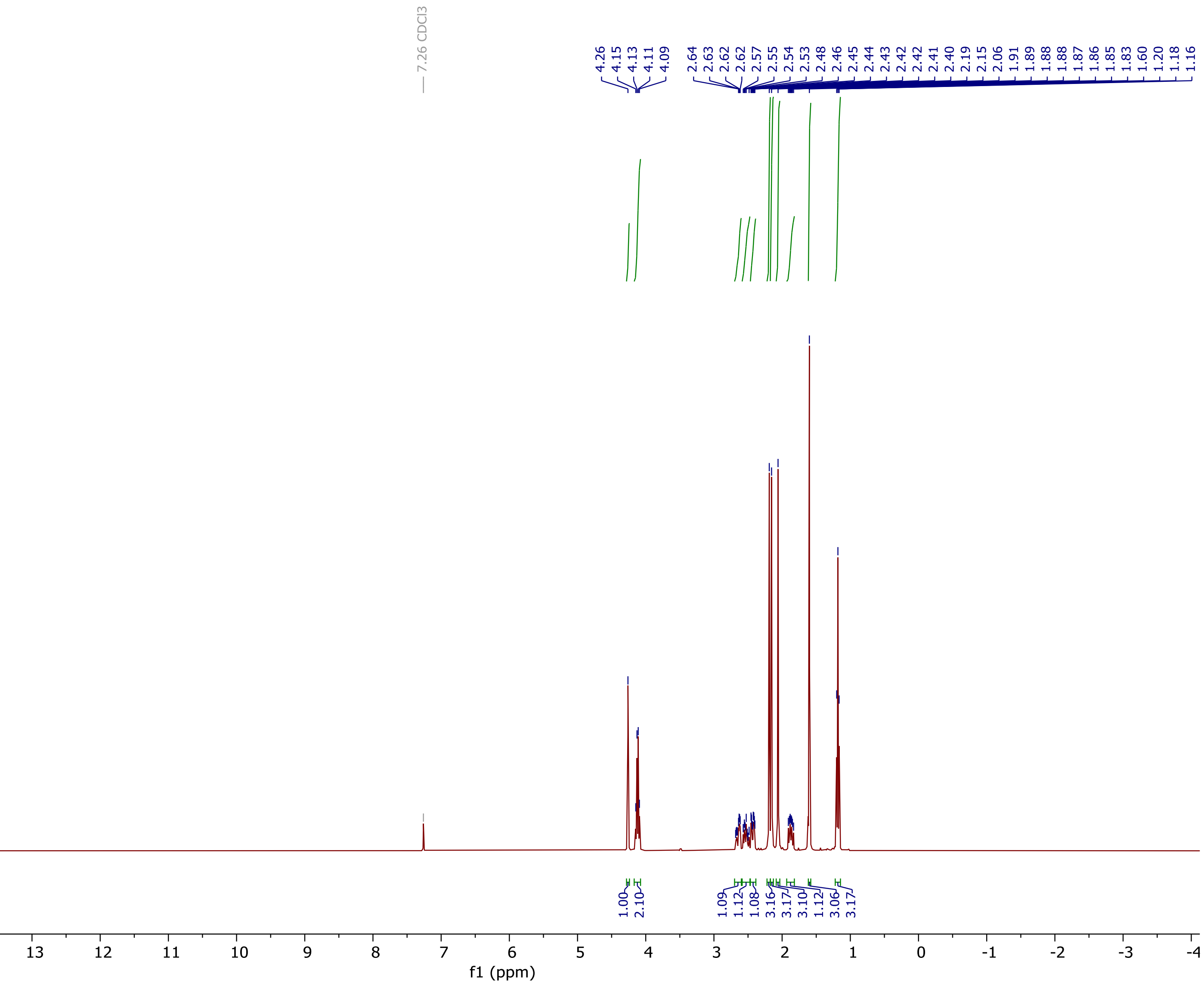

Ethyl (*S*)-6-hydroxy-2,5,7,8-tetramethylchromane-2-carboxylate - $^{13}C\{^1H\}$ NMR (101 MHz, $CDCl_3$)

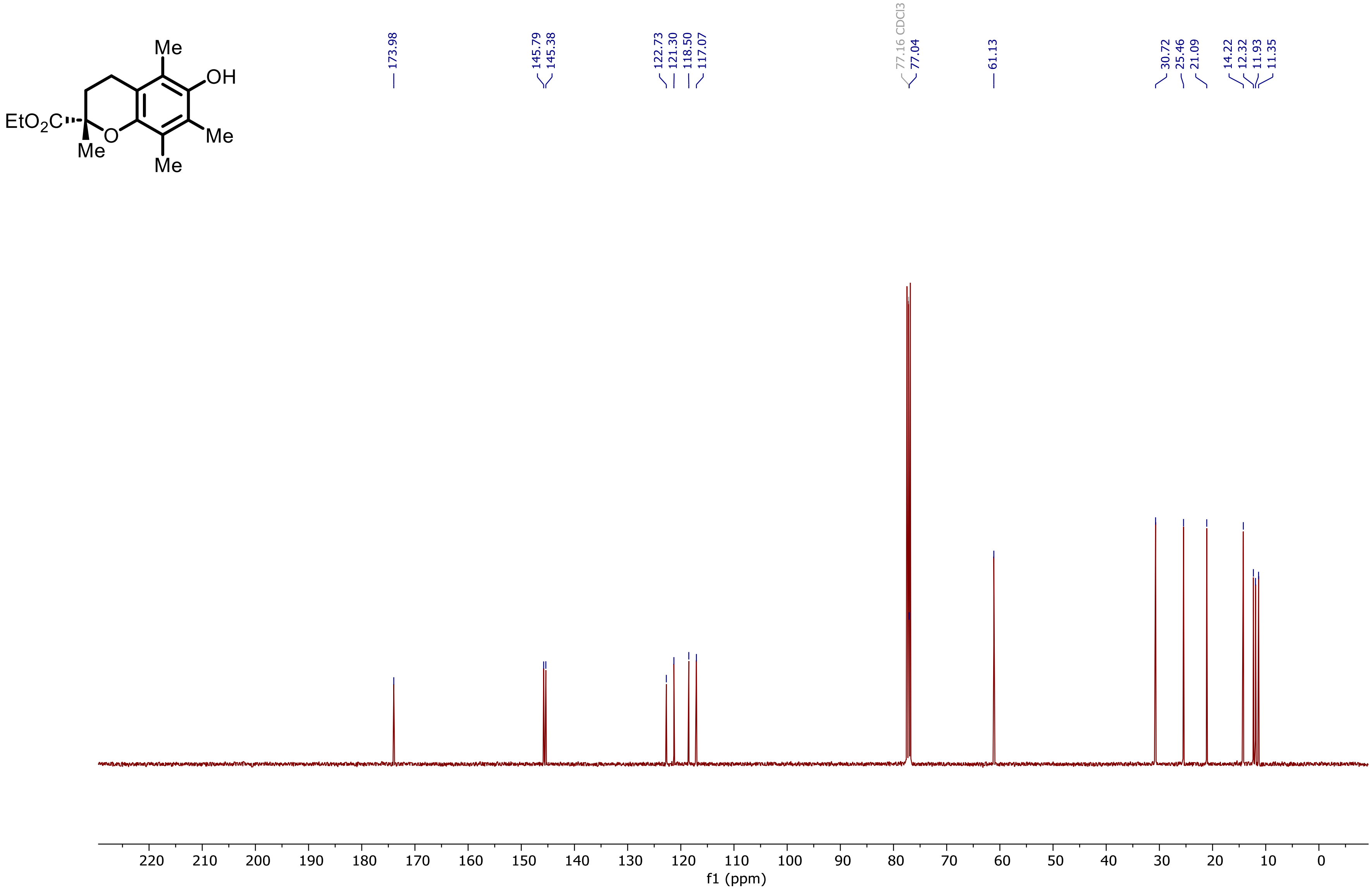

Methyl (1*S*,4a*S*,10a*R*)-6-hydroxy-1,4a-dimethyl-1,2,3,4,4a,9,10,10a-octahydrophenanthrene-1-carboxylate - ^{1}H NMR (400 MHz, $CDCl_3$)

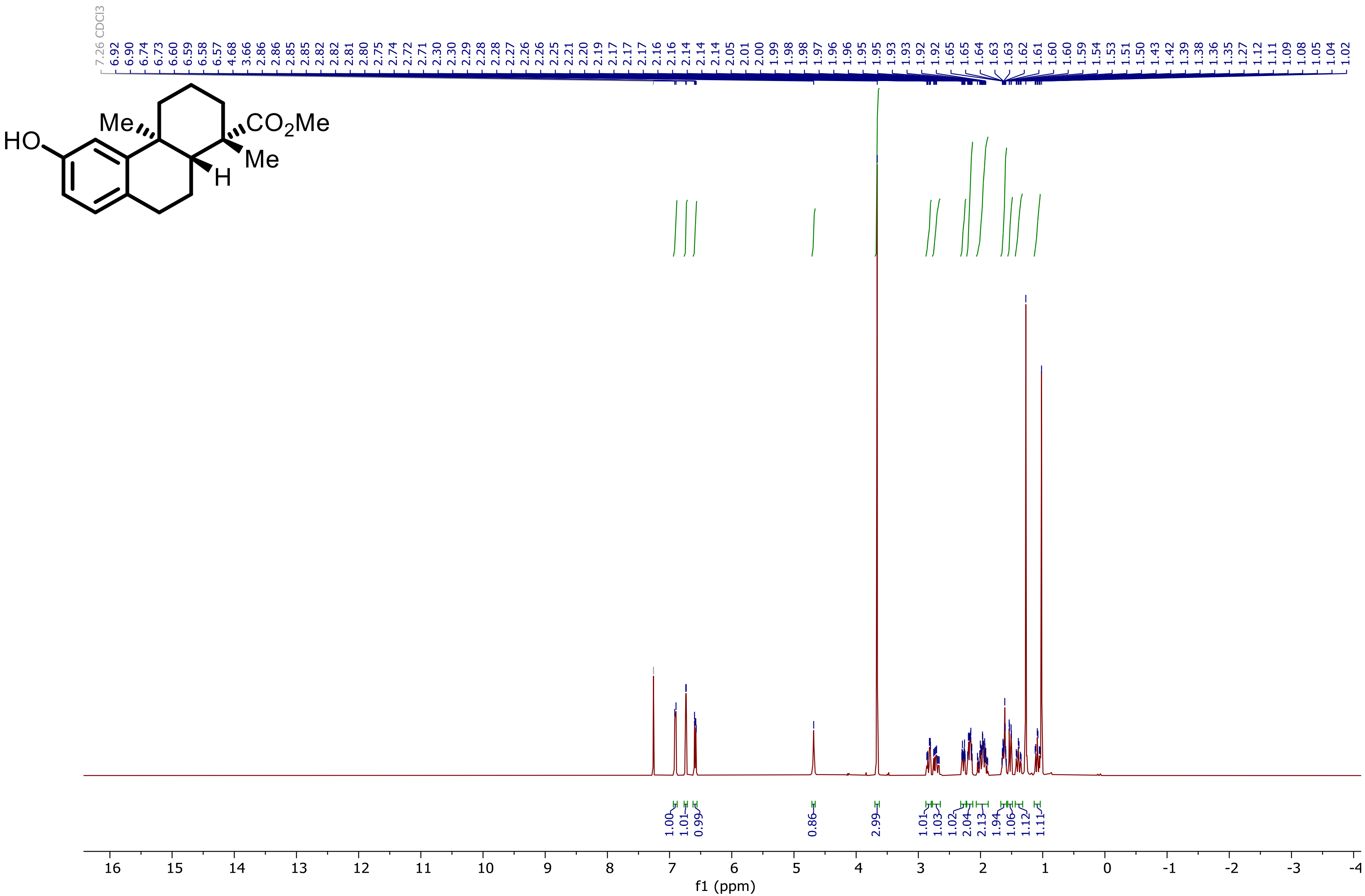

Methyl (1*S*,4a*S*,10a*R*)-6-hydroxy-1,4a-dimethyl-1,2,3,4,4a,9,10,10a-octahydrophenanthrene-1-carboxylate - $^{13}C\{^1H\}$ NMR (101 MHz, $CDCl_3$)

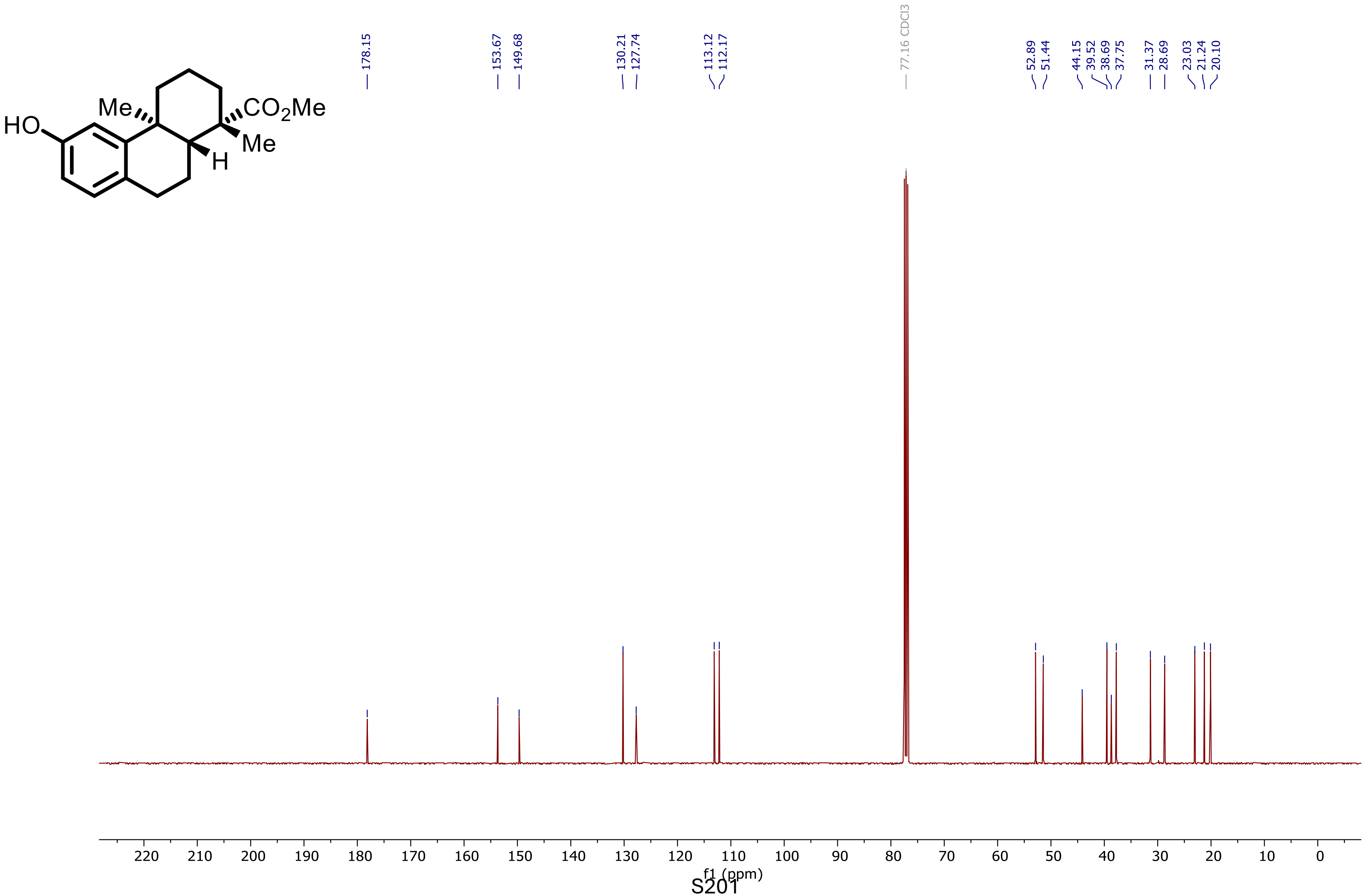

^{DMP}DAB-Pd-BTSM - ^{1}H NMR (400 MHz, C_6D_6)

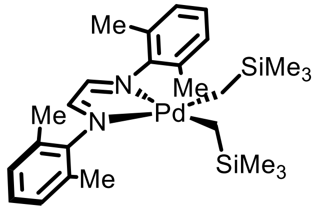

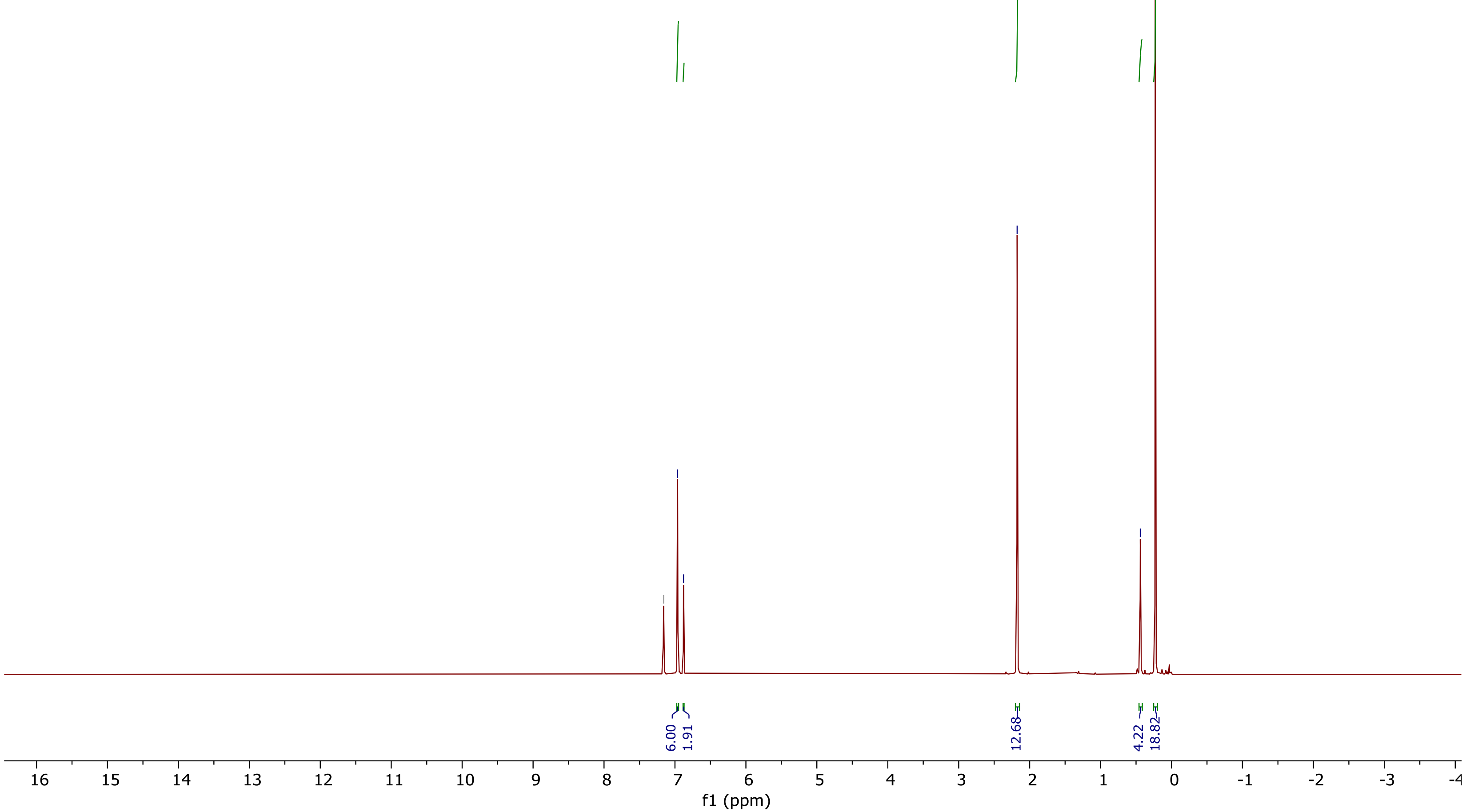

^{DMP}DAB-Pd-BTSM - $^{13}C\{^1H\}$ NMR (101 MHz, C_6D_6)

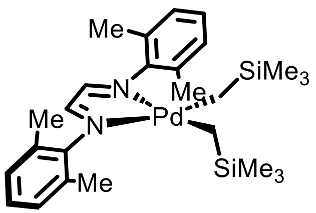

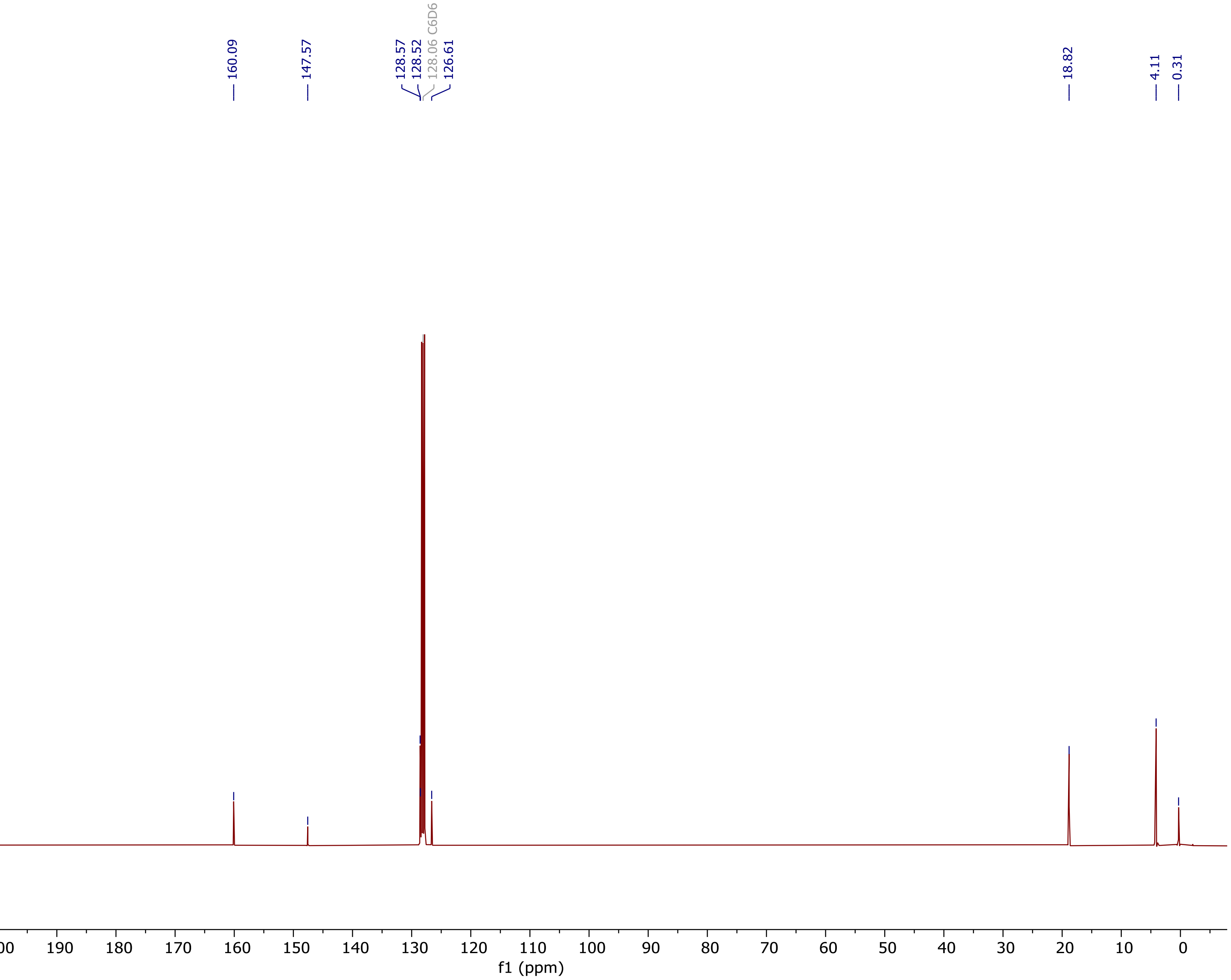

(AdBippyPhos)Pd^{II}(C_6H_4F)(OTf) - ^{1}H NMR (500 MHz, $CDCl_3$)

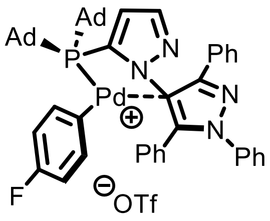

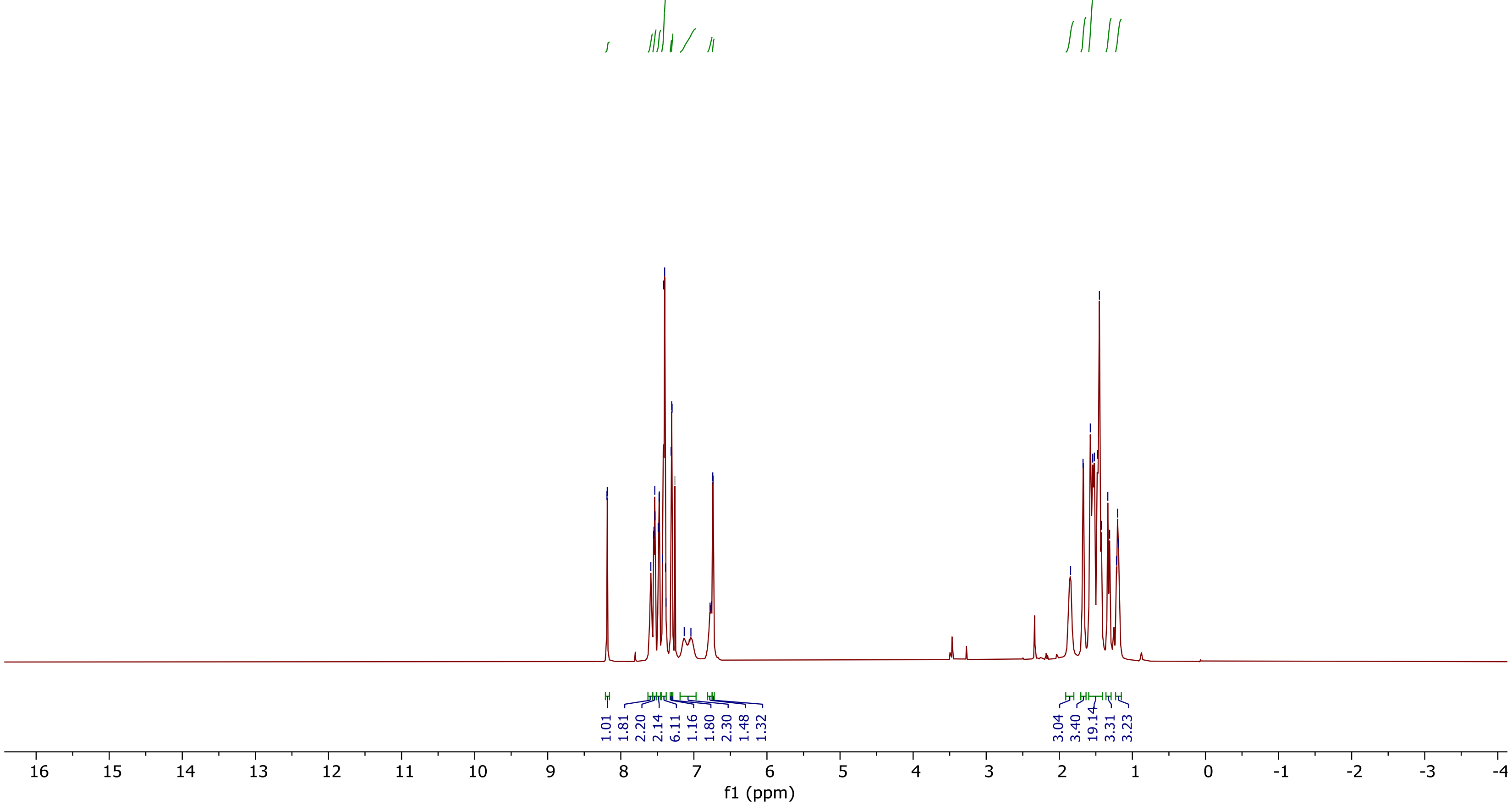

(AdBippyPhos)Pd^{II}(C_6H_4F)(OTf) - $^{13}C\{^1H\}$ NMR (126 MHz, $CDCl_3$)

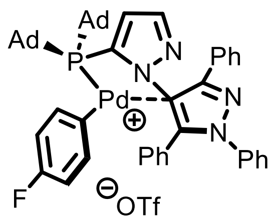

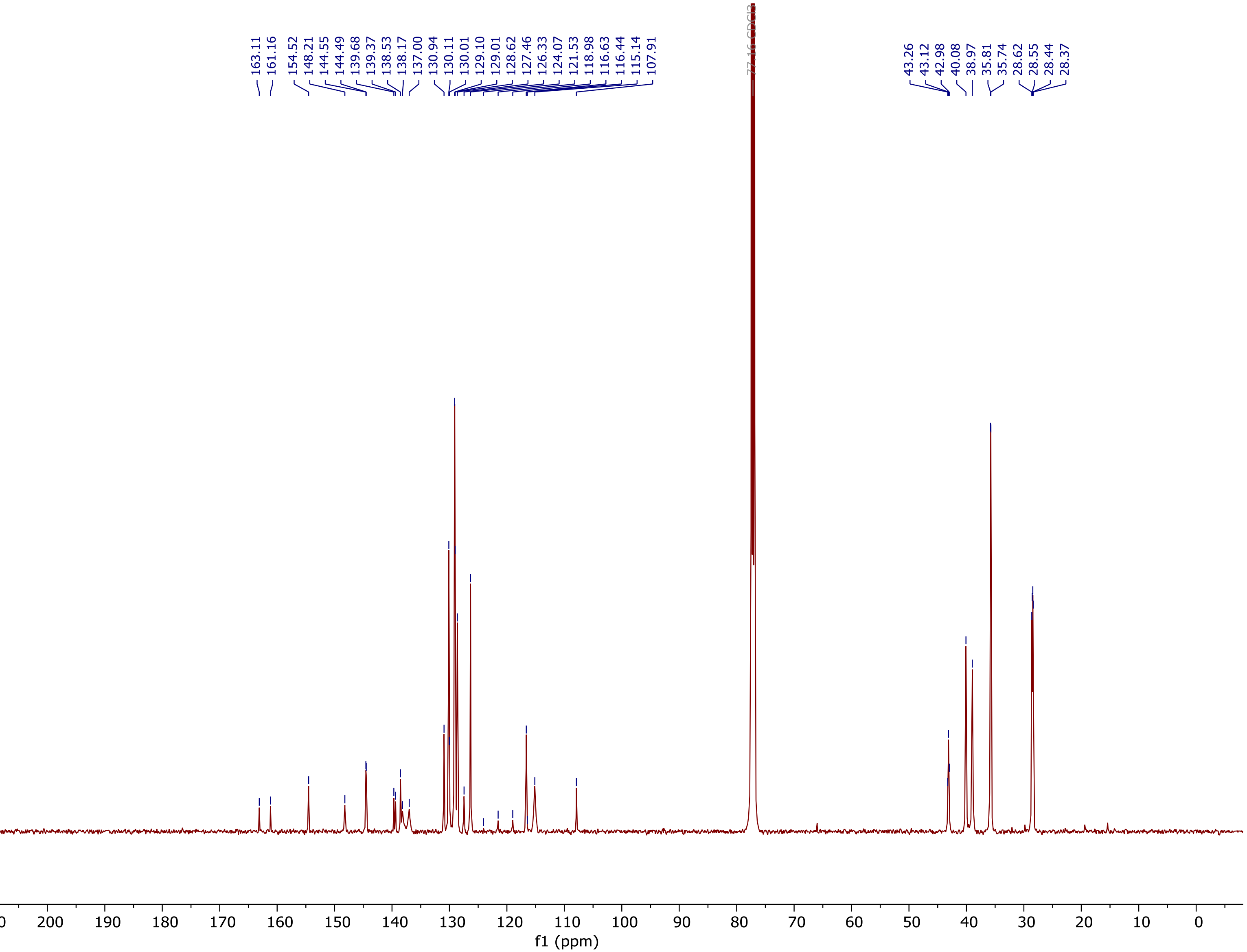

(AdBippyPhos)Pd^{II}(C_6H_4F)(OTf) - $^{31}P\{^1H\}$ NMR (162 MHz, $CDCl_3$)

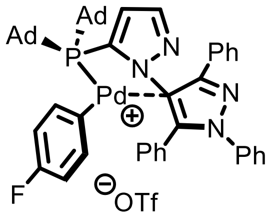

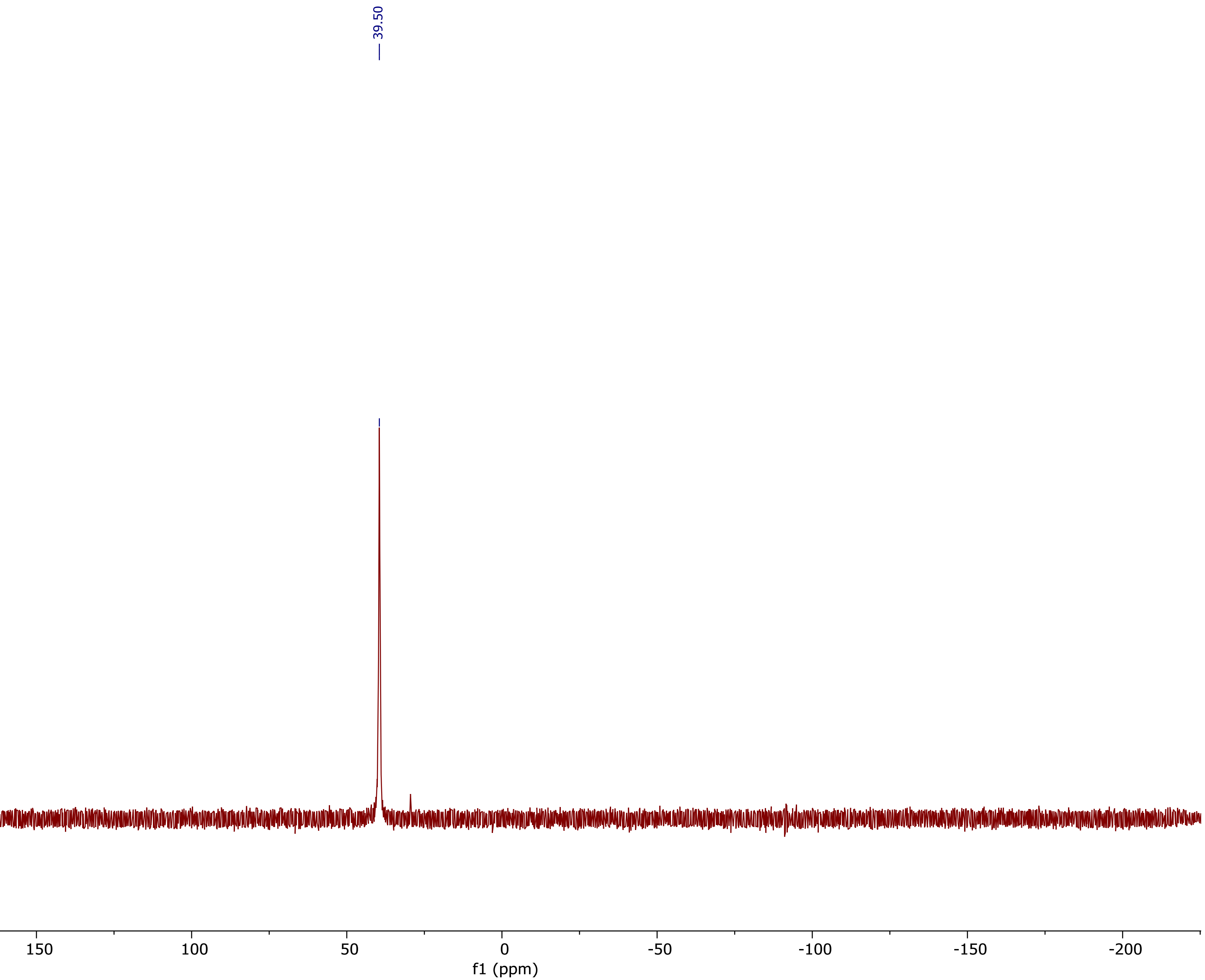

(AdBippyPhos)Pd^{II}(C_6H_4F)(OTf) - ^{19}F NMR (377 MHz, $CDCl_3$)

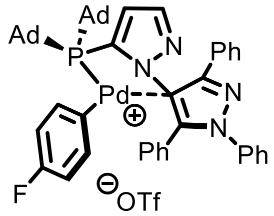

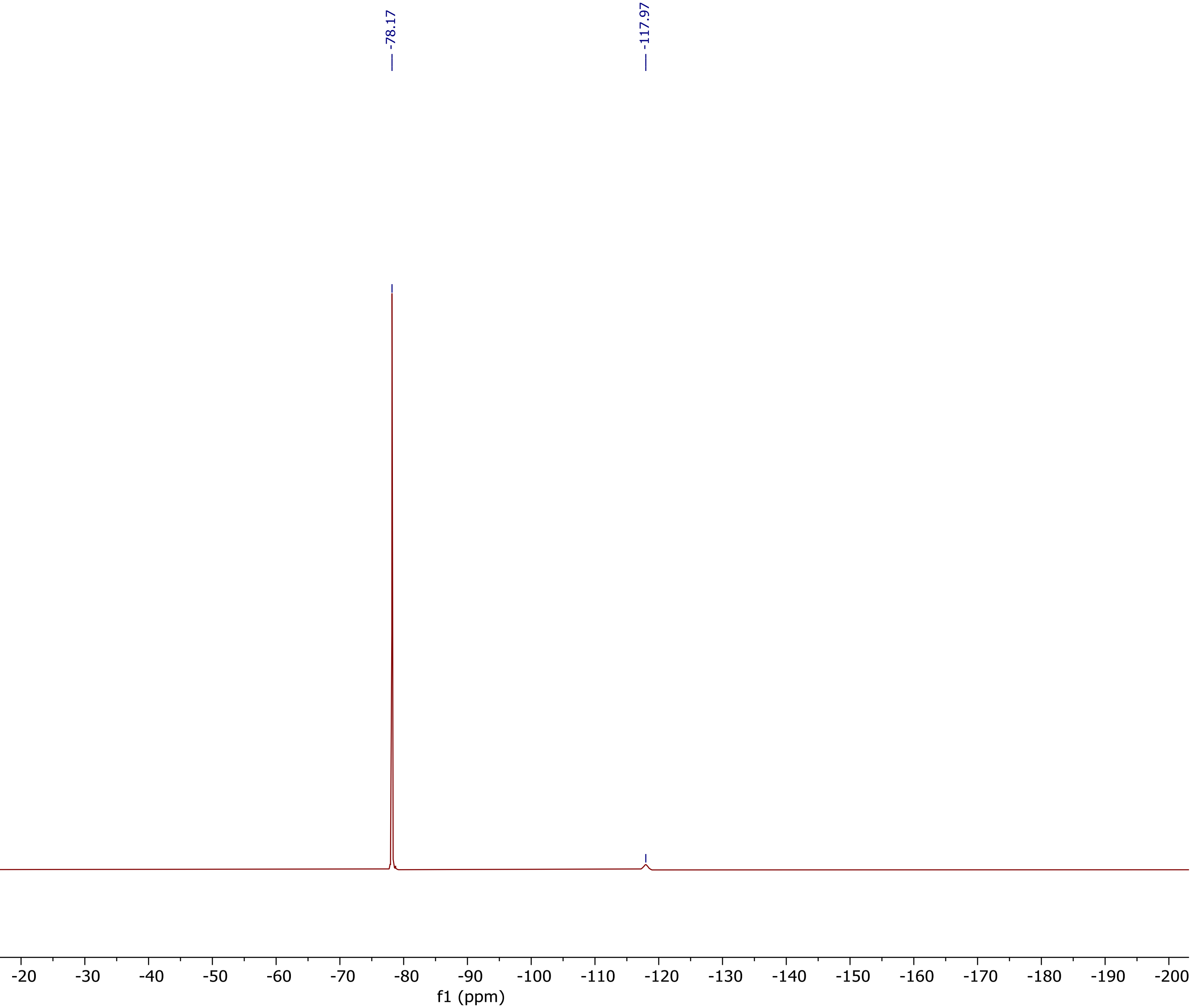

(AdBippyPhos)Pd^{II}(C_6H_4F)(OTf) - ^{19}F NMR (377 MHz, PhMe-d_8)

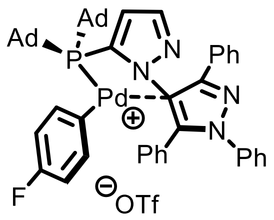

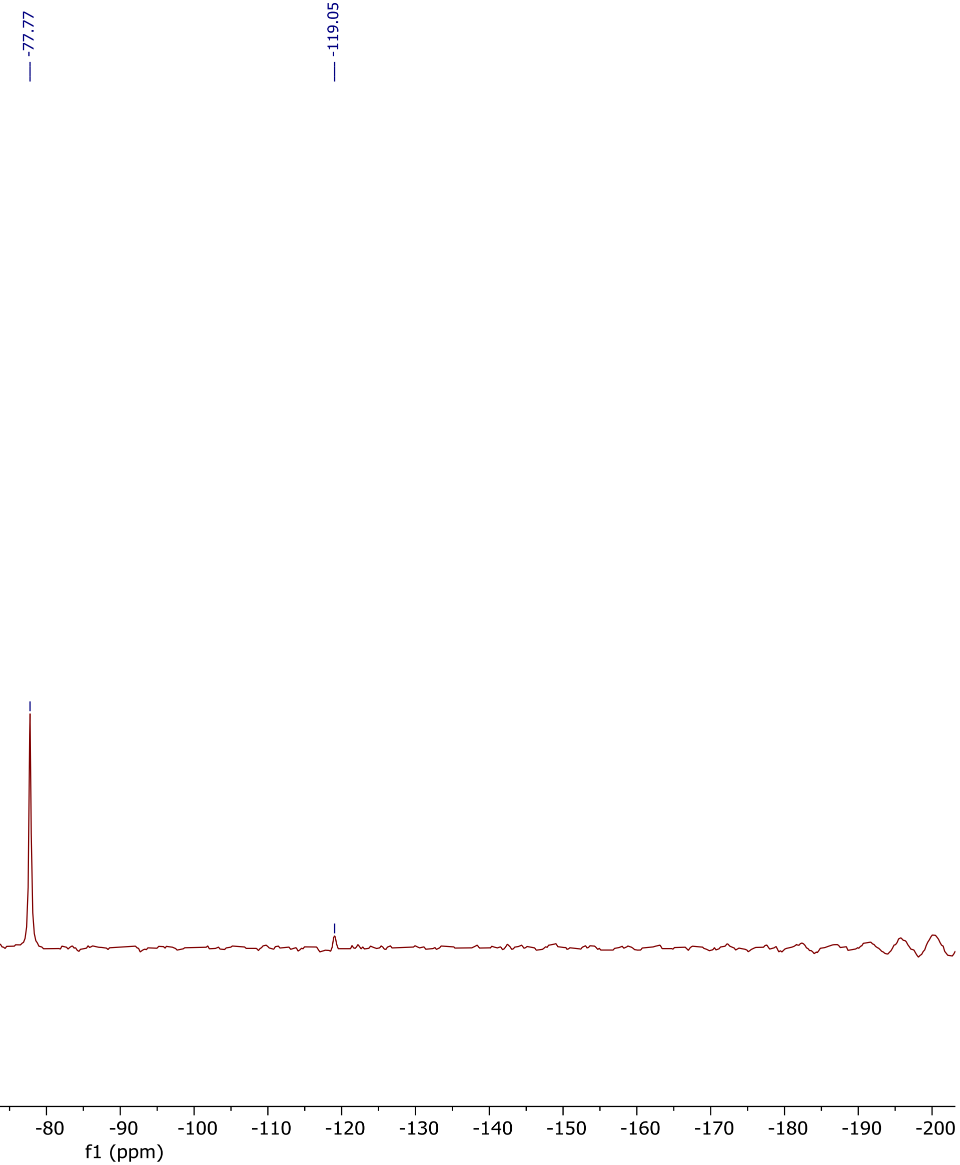

3 – ^{1}H NMR (400 MHz, $CDCl_3$)

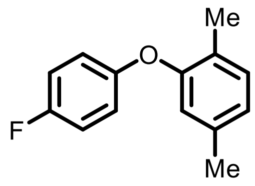

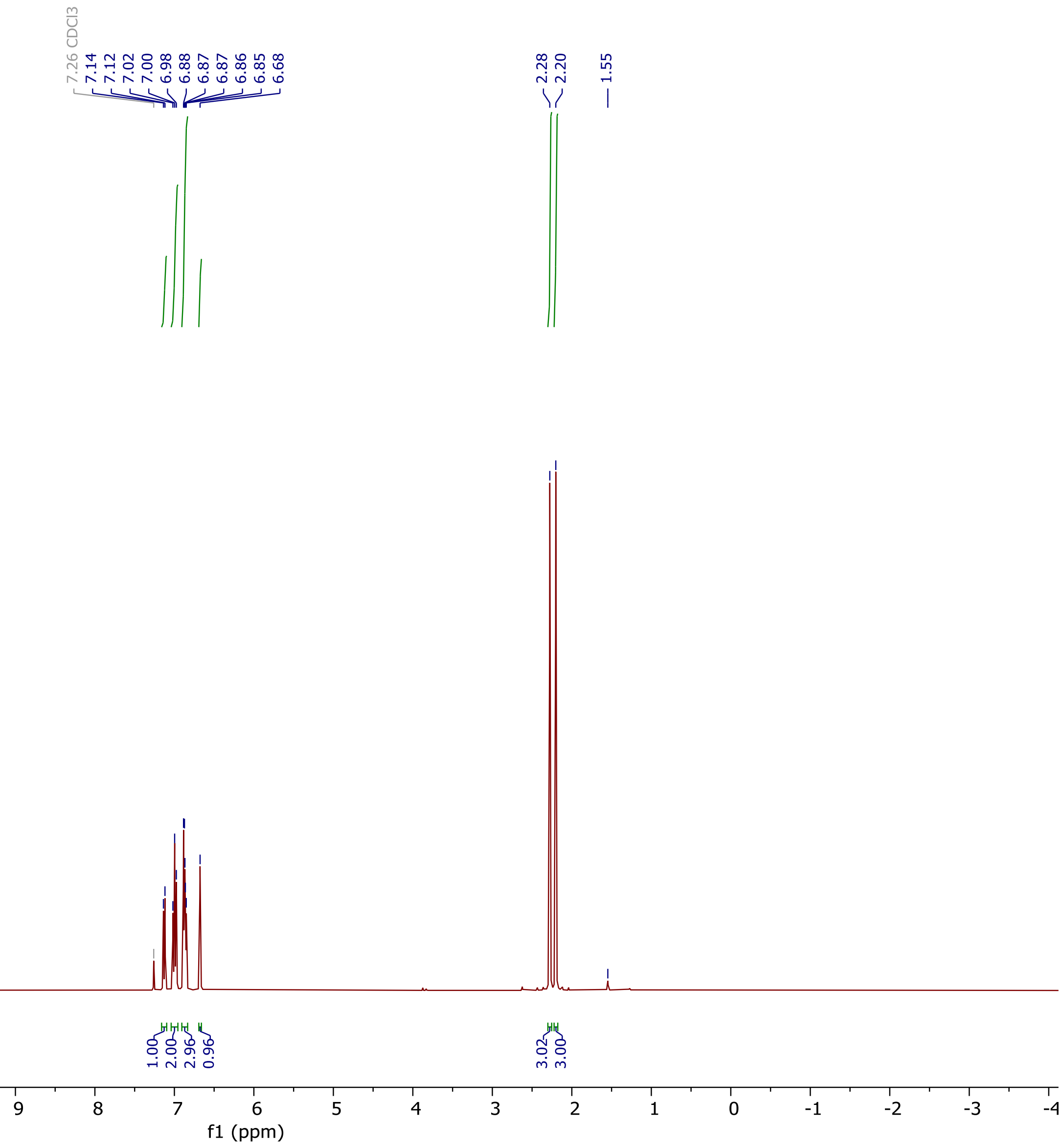

3 – $^{13}C\{^1H\}$ NMR (101 MHz, $CDCl_3$)

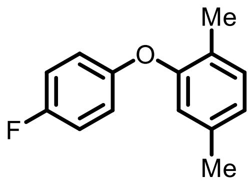

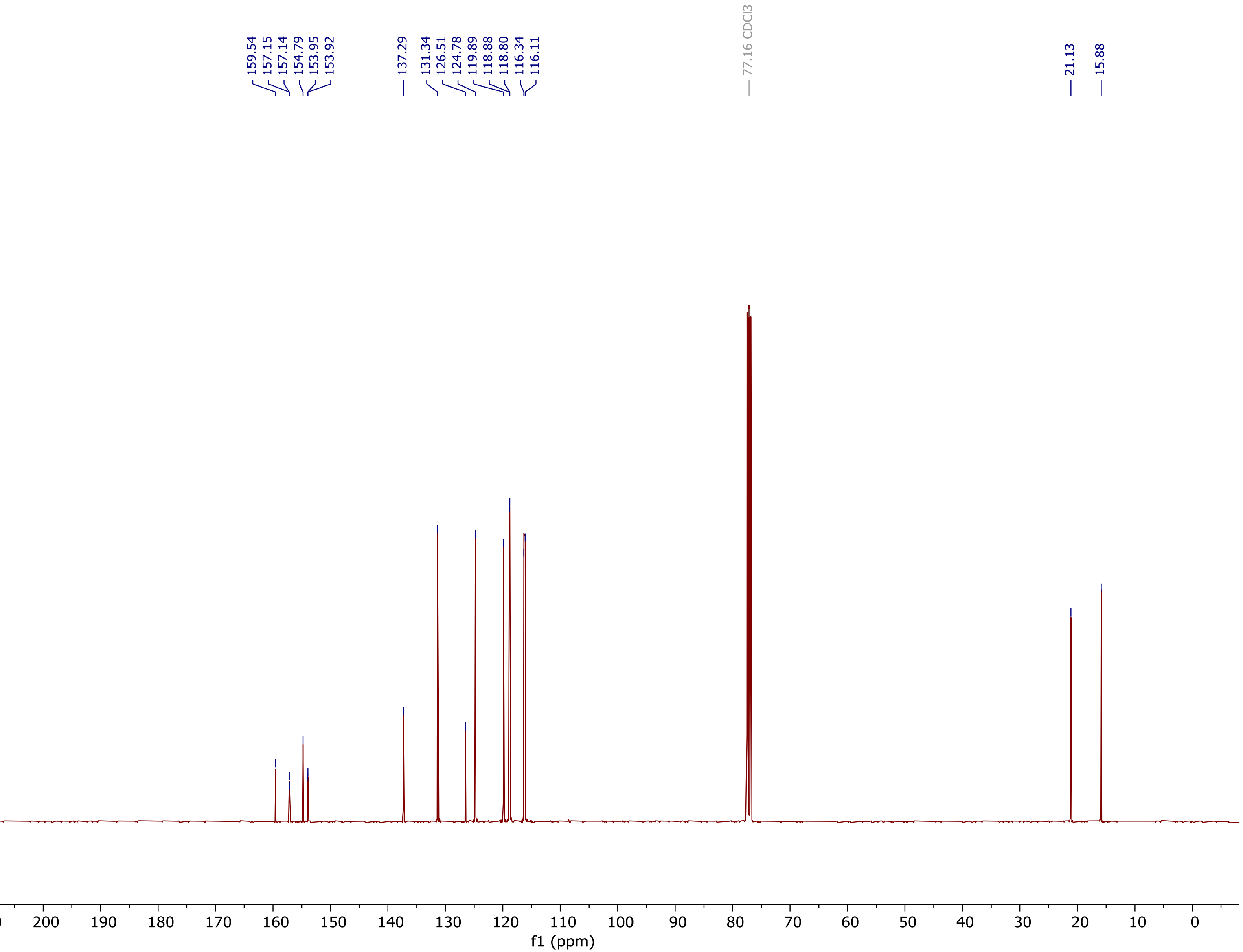

3 – ^{19}F NMR (376 MHz, $CDCl_3$)

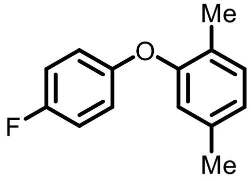

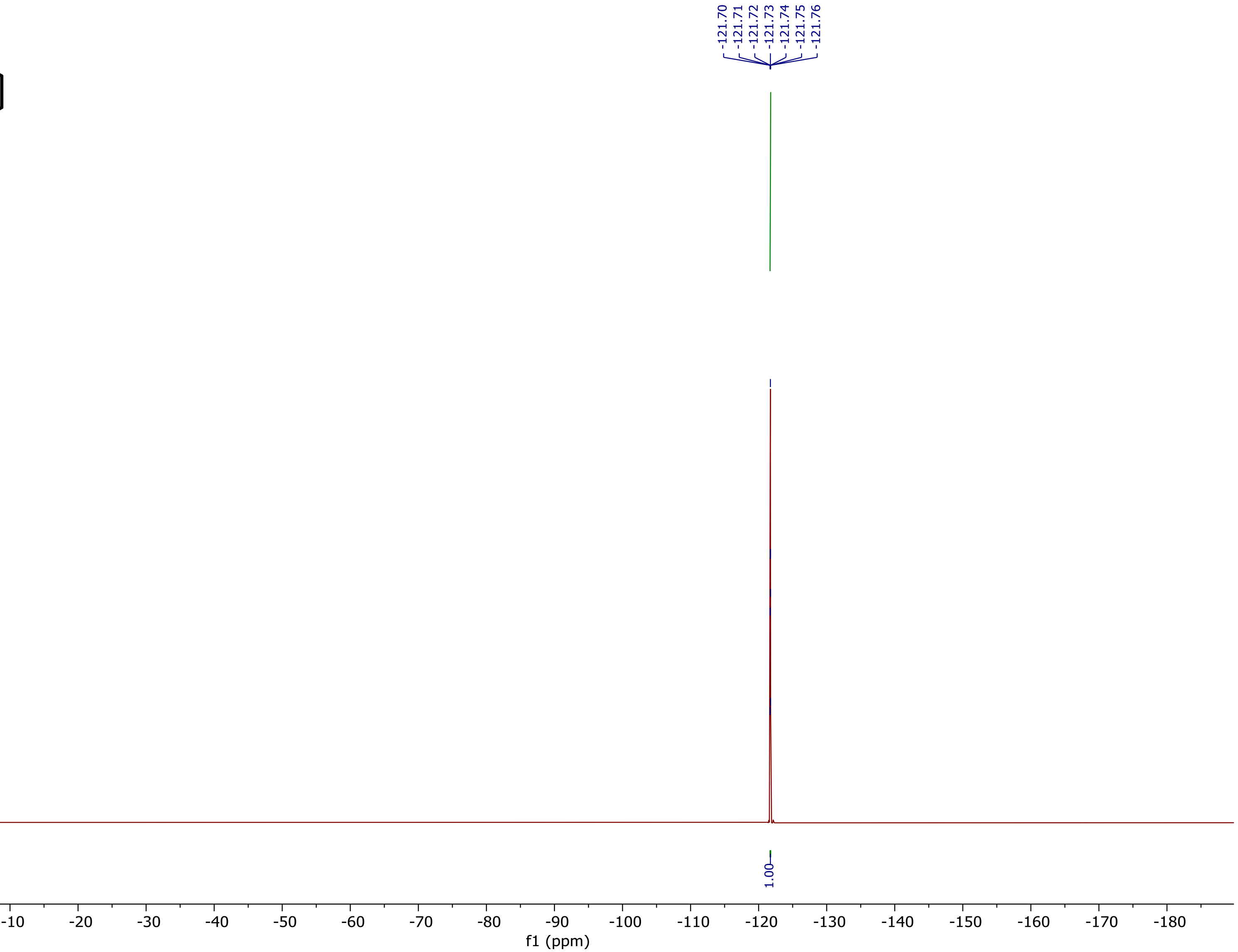

4 - ^{1}H NMR (500 MHz, $CDCl_3$)

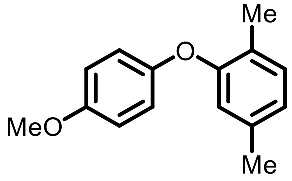

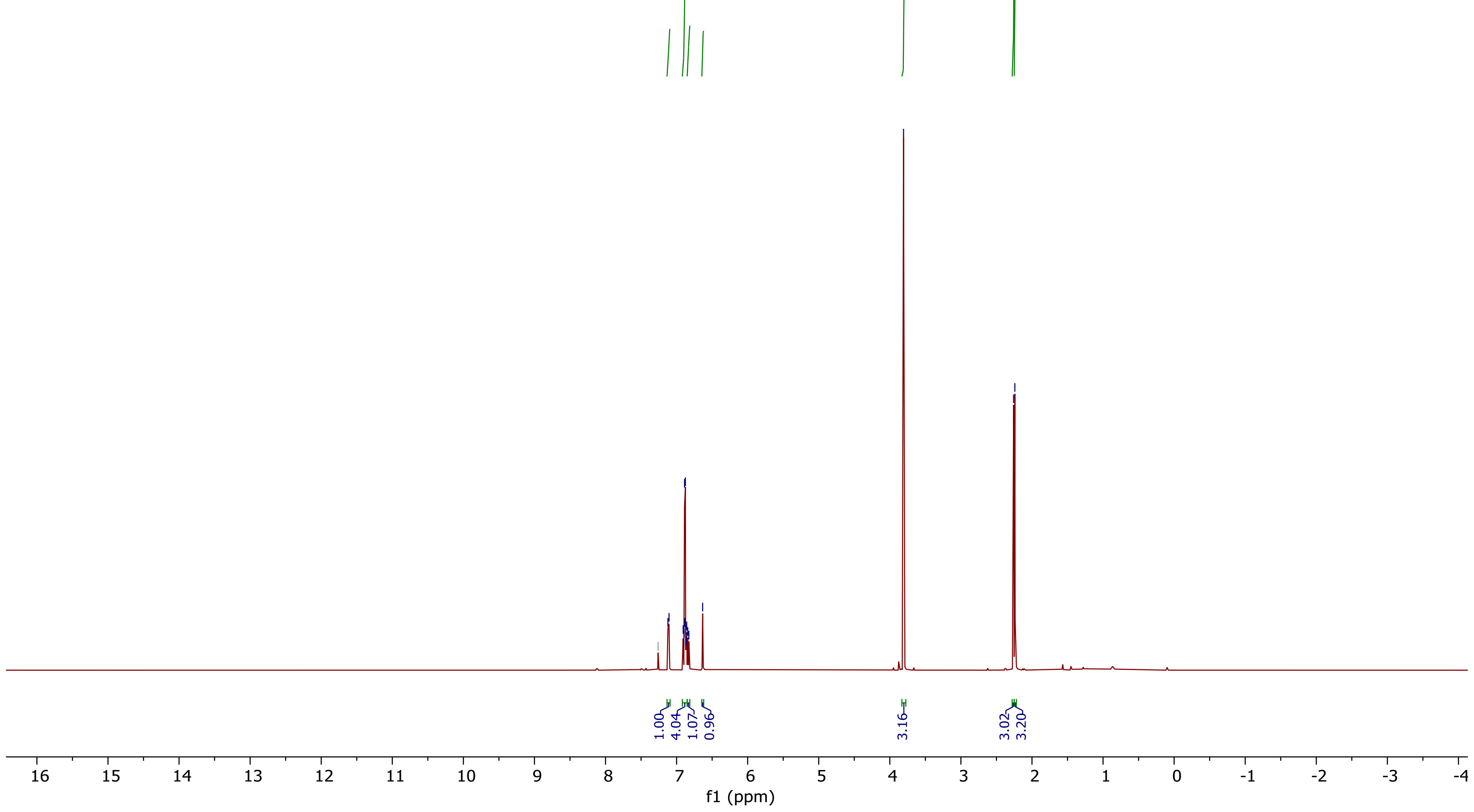

4 - $^{13}C\{^{1}H\}$ NMR (126 MHz, $CDCl_3$)

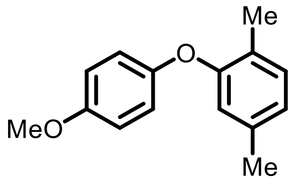

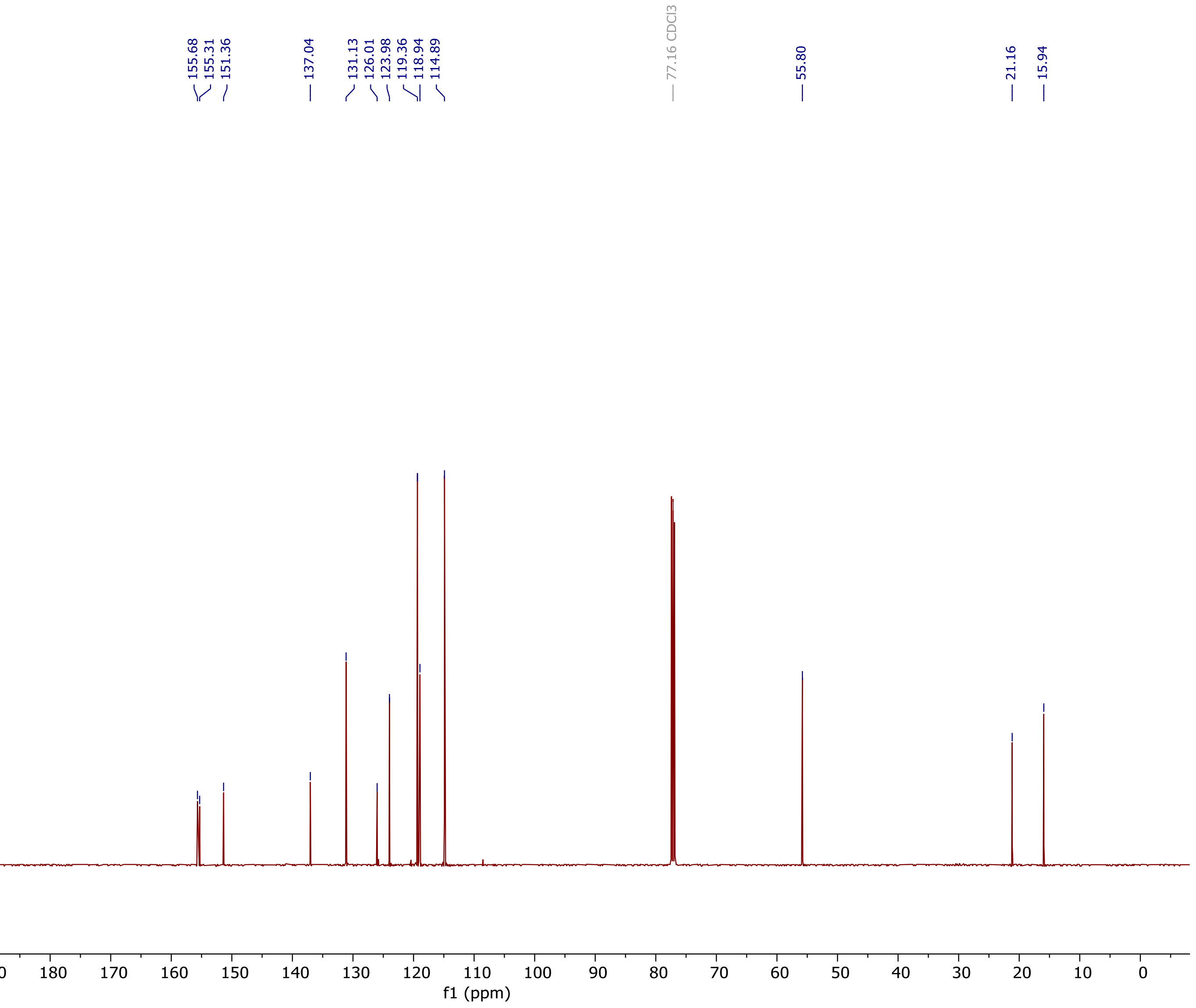

5 – ¹H NMR (400 MHz, CDCl₃) – *note: residual CHCl₃ solvent peak is not resolved from 1×H of product.*

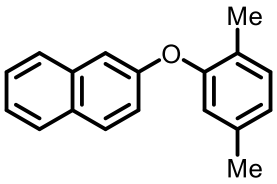

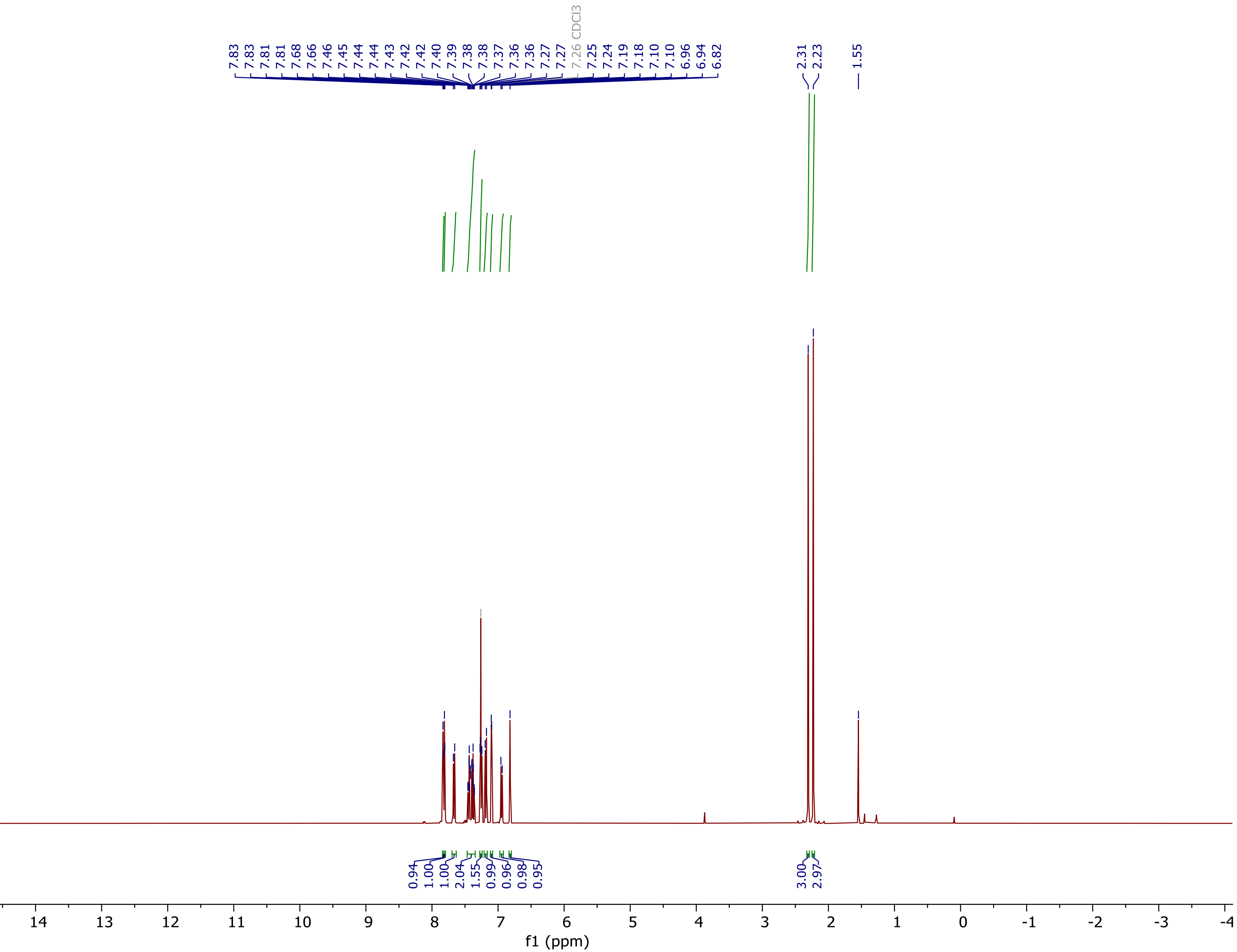

5 – ¹H NMR (400 MHz, CDCl$_3$)

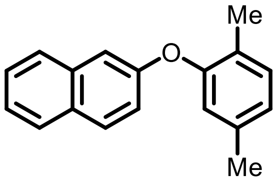

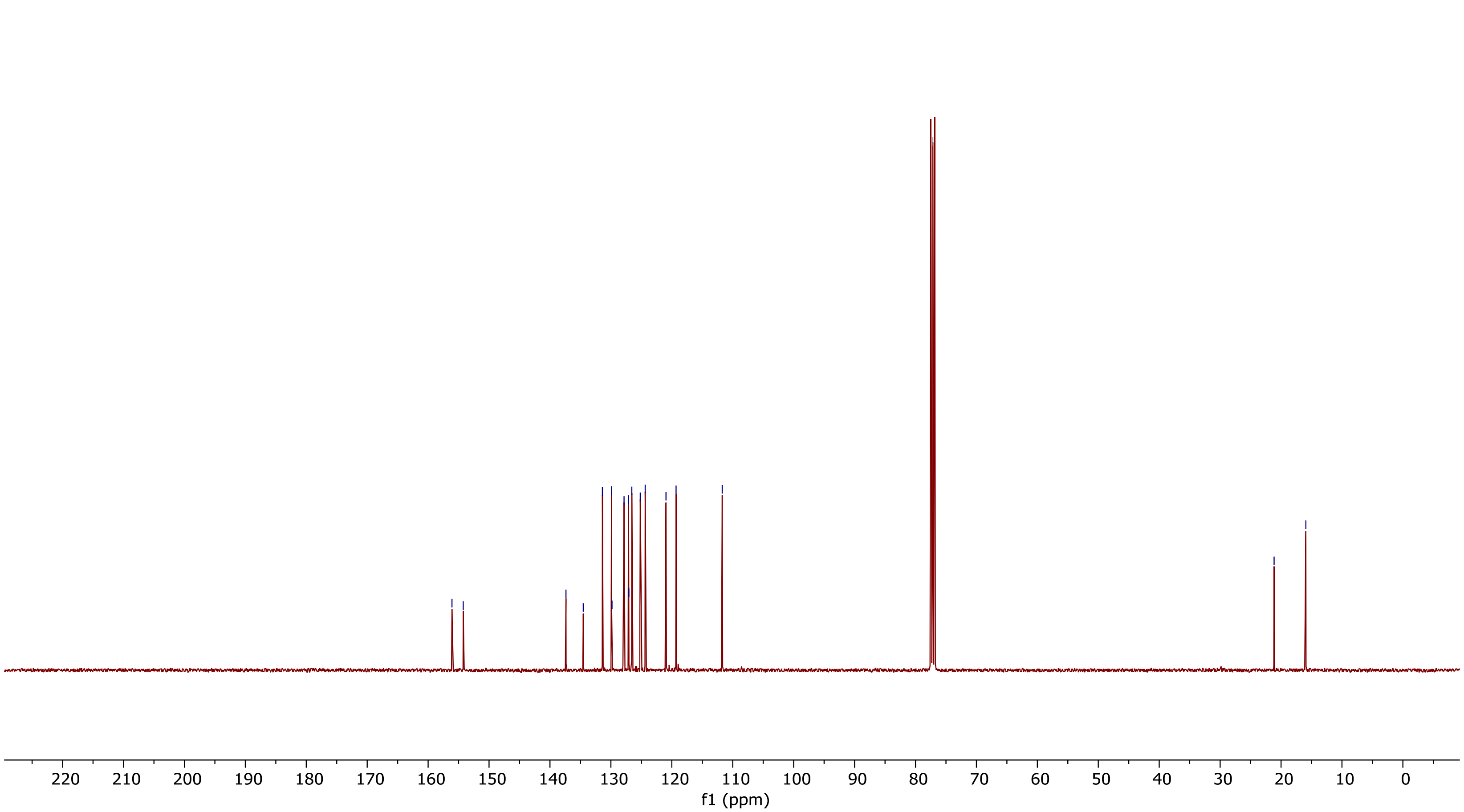

6 – ¹H NMR (400 MHz, $CDCl_3$)

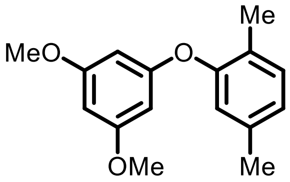

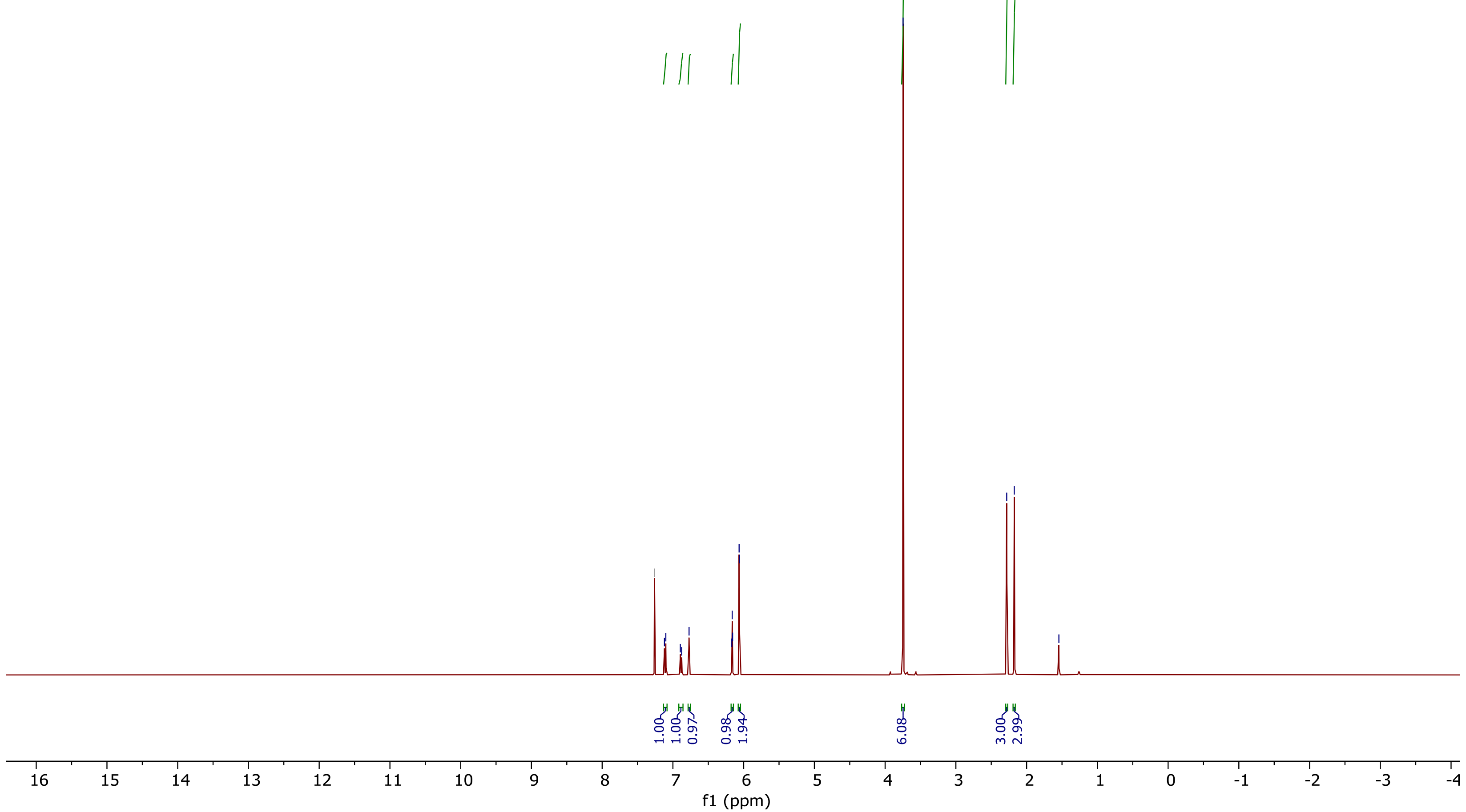

6 – $^{13}C\{^{1}H\}$ NMR (101 MHz, $CDCl_3$)

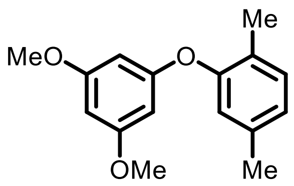

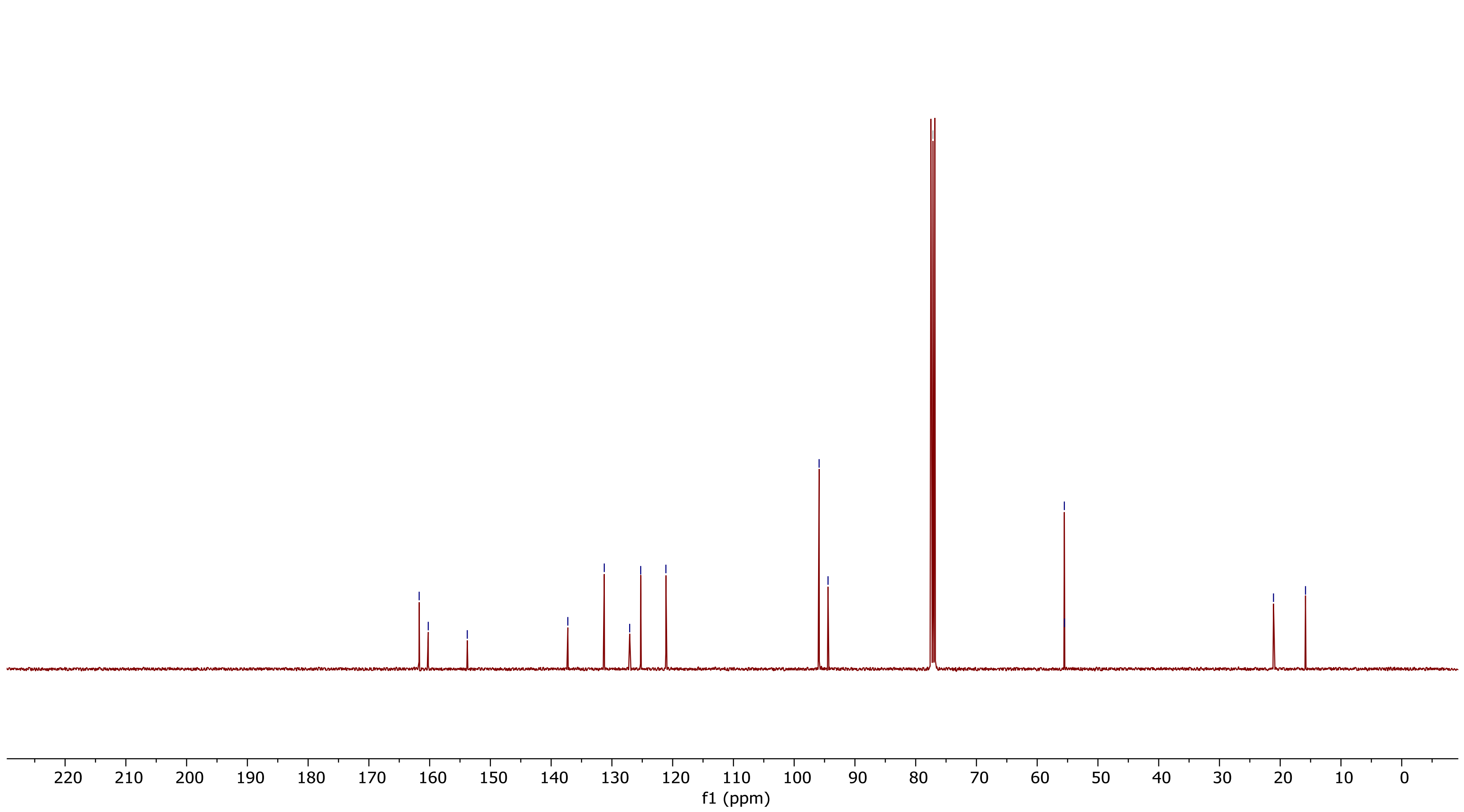

7 - ¹H NMR (400 MHz, $CDCl_3$)

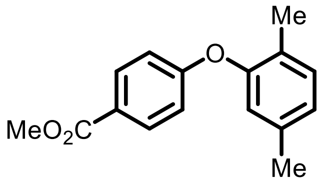

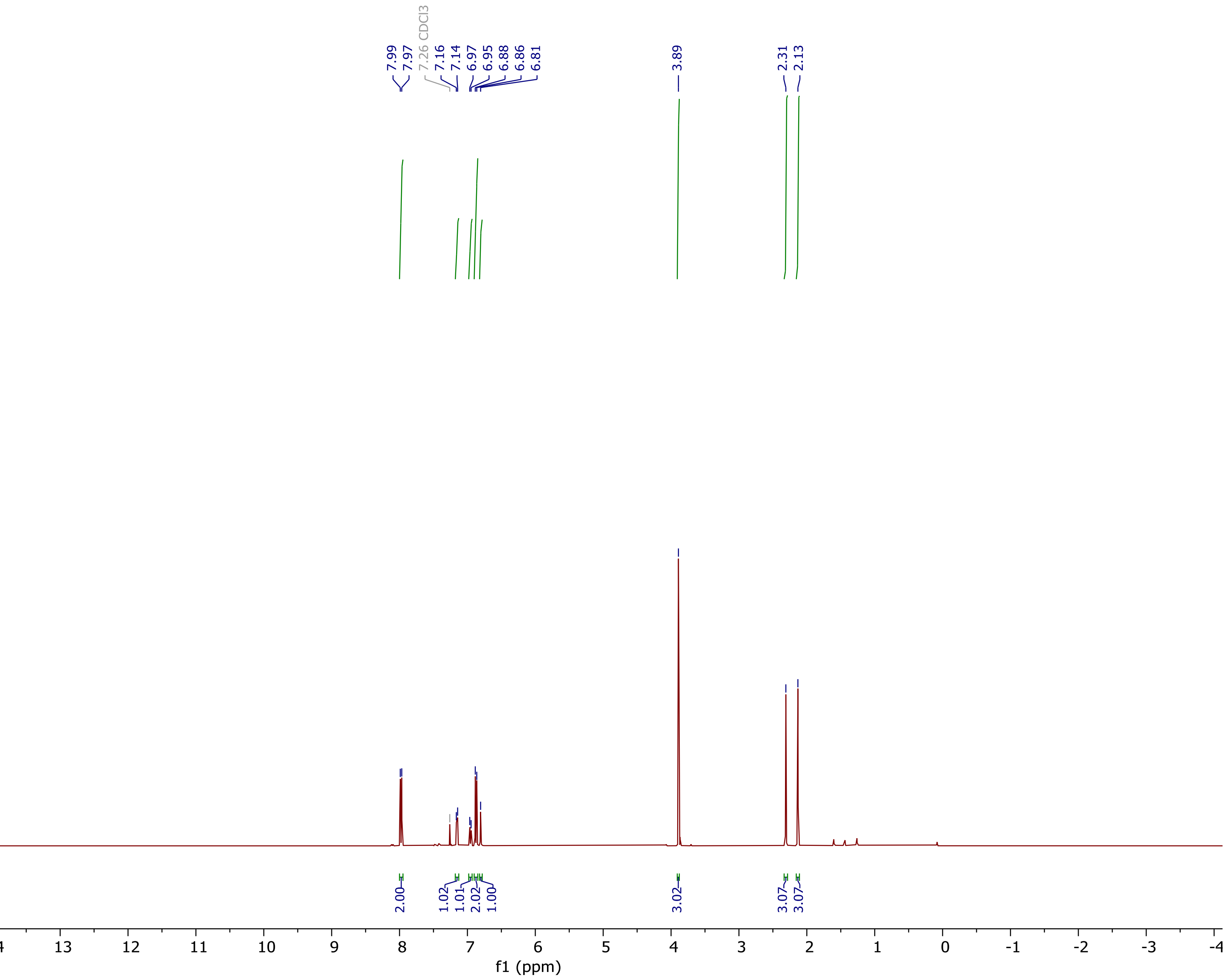

7 - $^{13}C\{^1H\}$ NMR (101 MHz, $CDCl_3$)

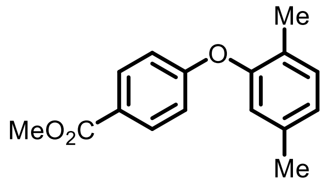

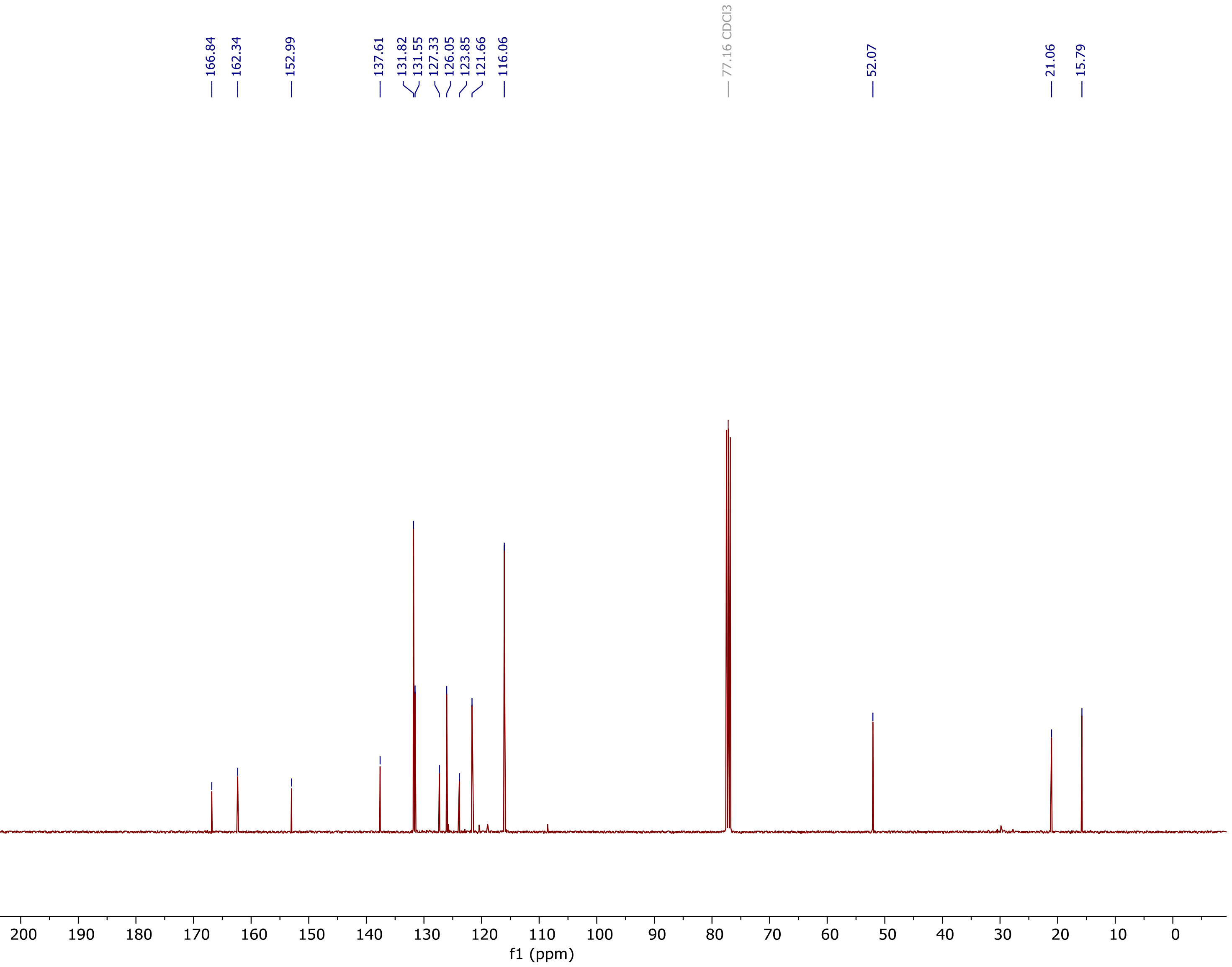

8 - ^{1}H NMR (500 MHz, $CDCl_3$)

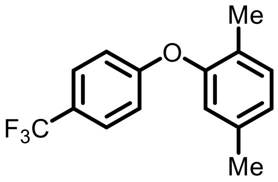

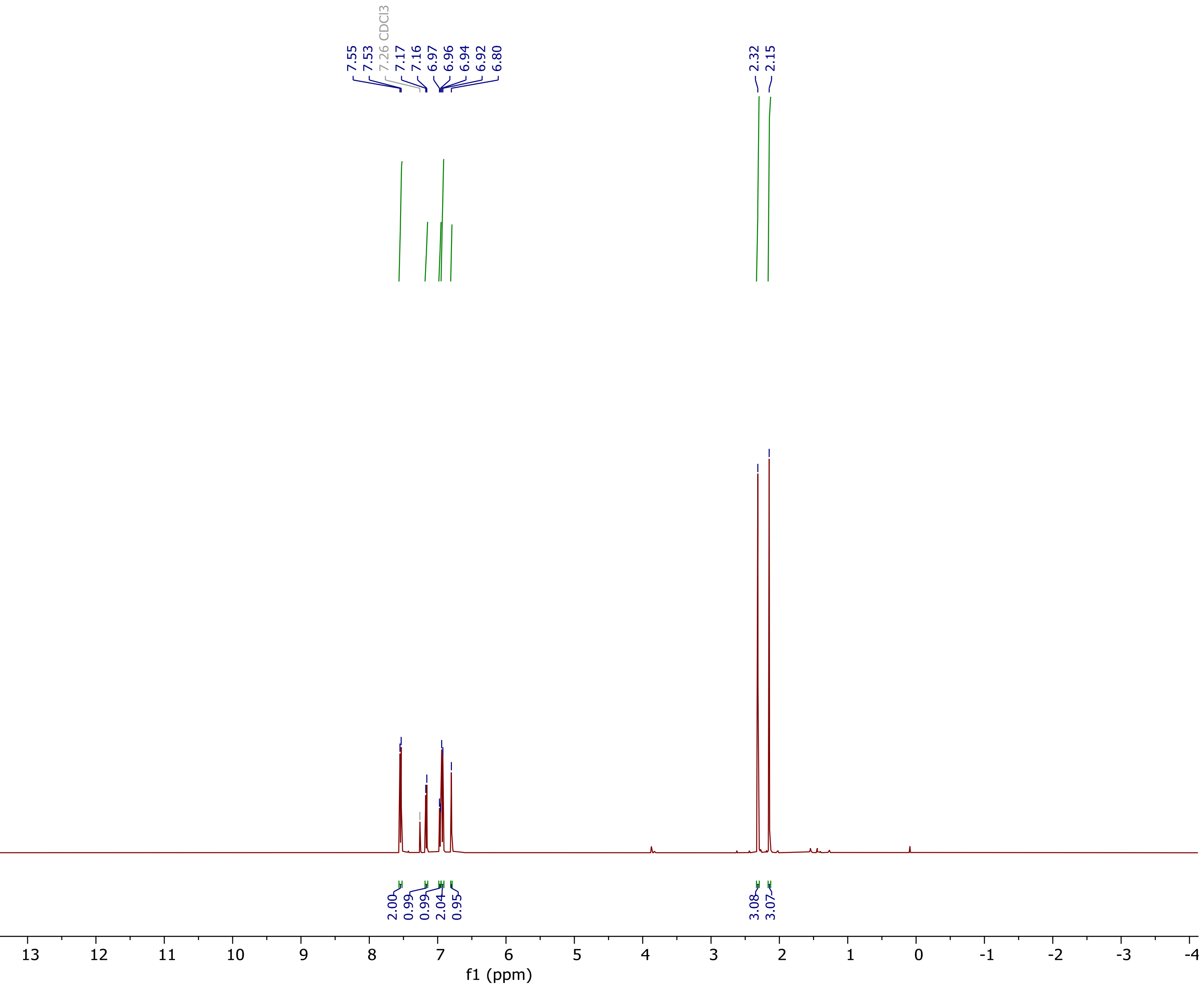

8 - $^{13}C\{^{1}H\}$ NMR (101 MHz, $CDCl_3$)

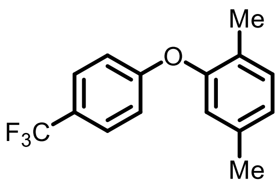

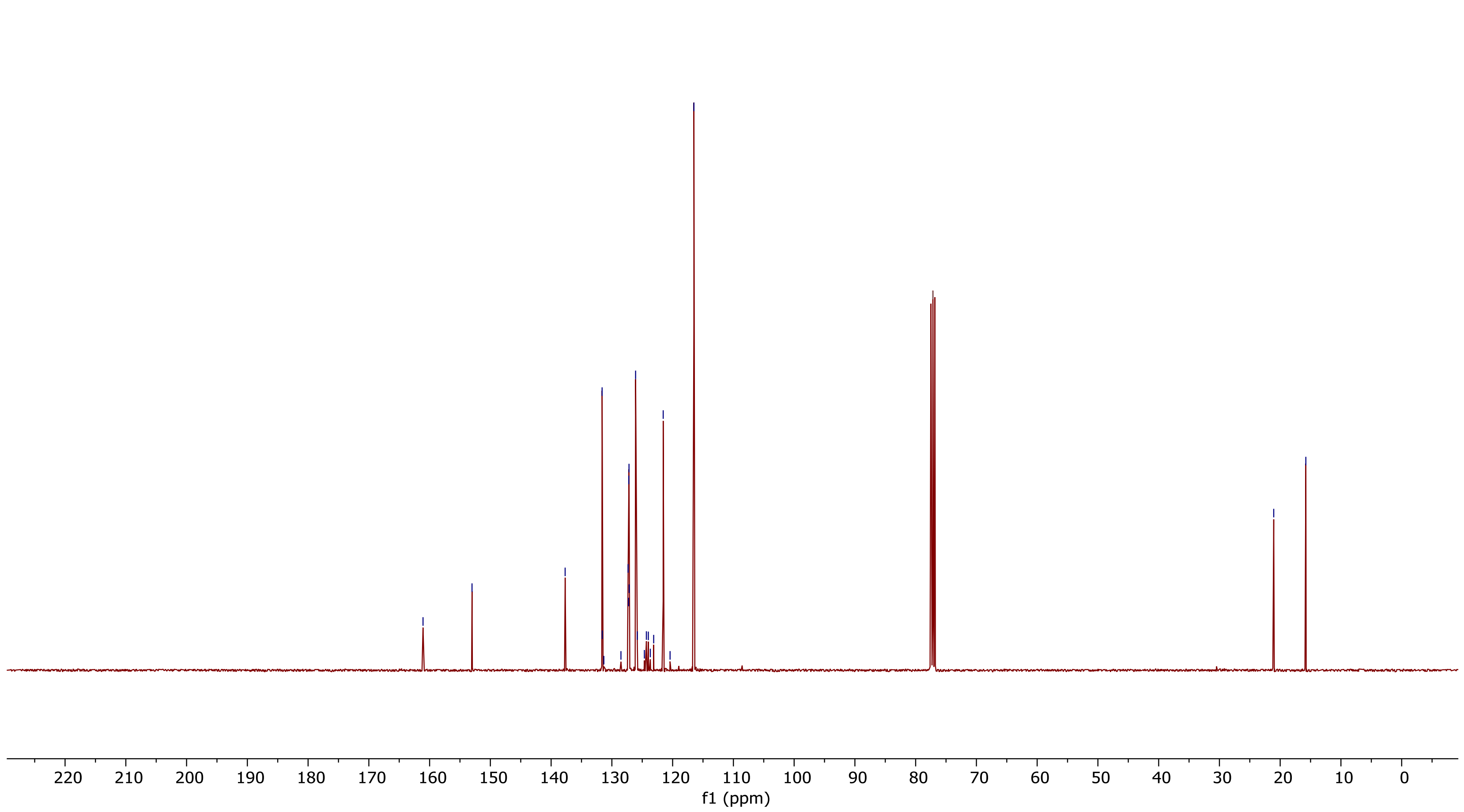

8 - ^{19}F NMR (376 MHz, $CDCl_3$)

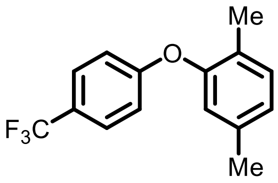

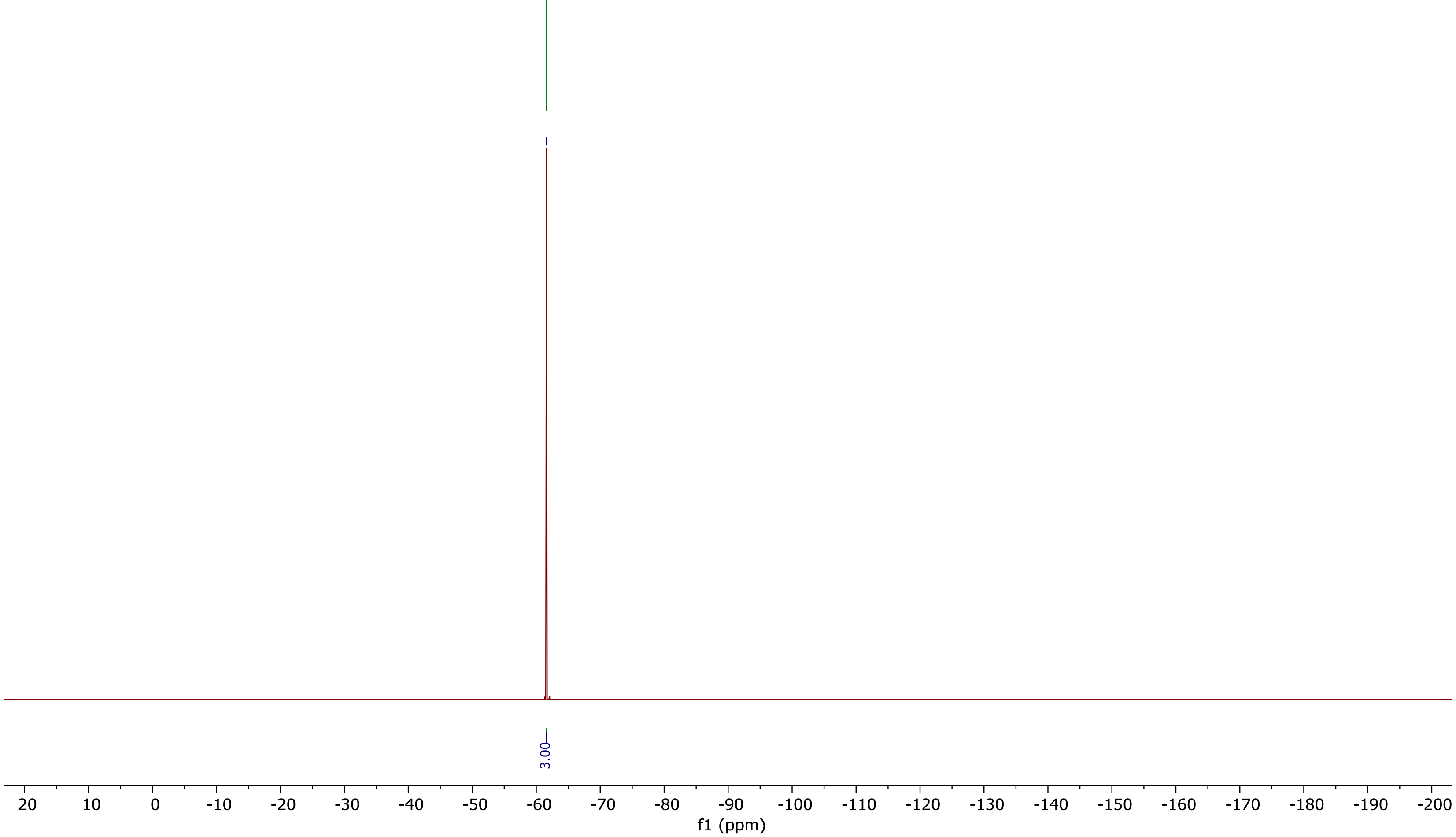

9 - ^{1}H NMR (500 MHz, $CDCl_3$)

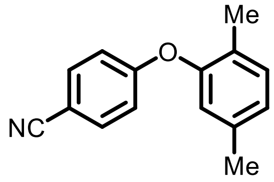

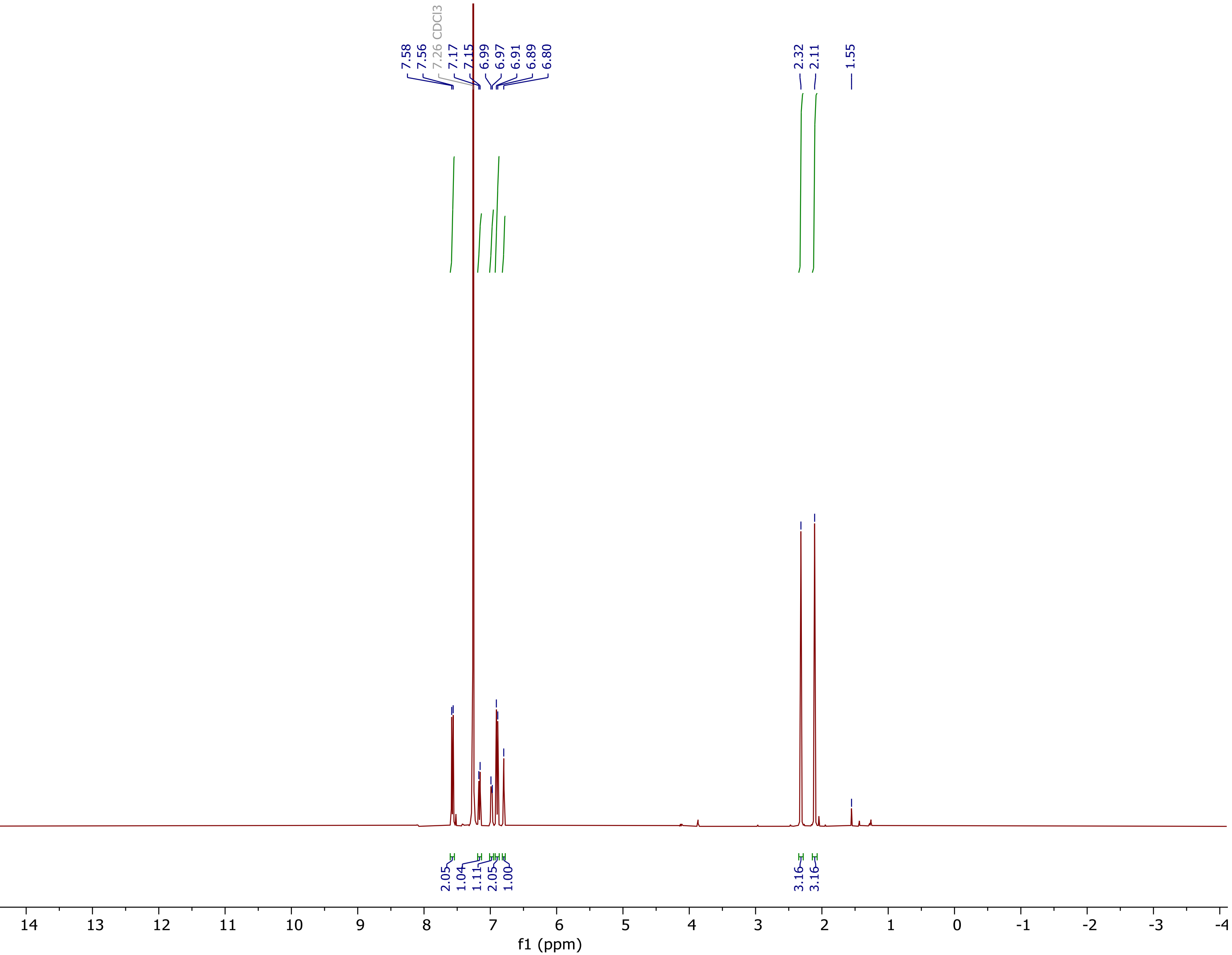

9 - $^{13}C\{^{1}H\}$ NMR (101 MHz, $CDCl_3$)

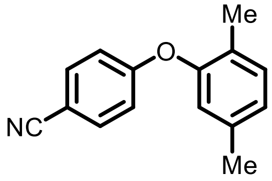

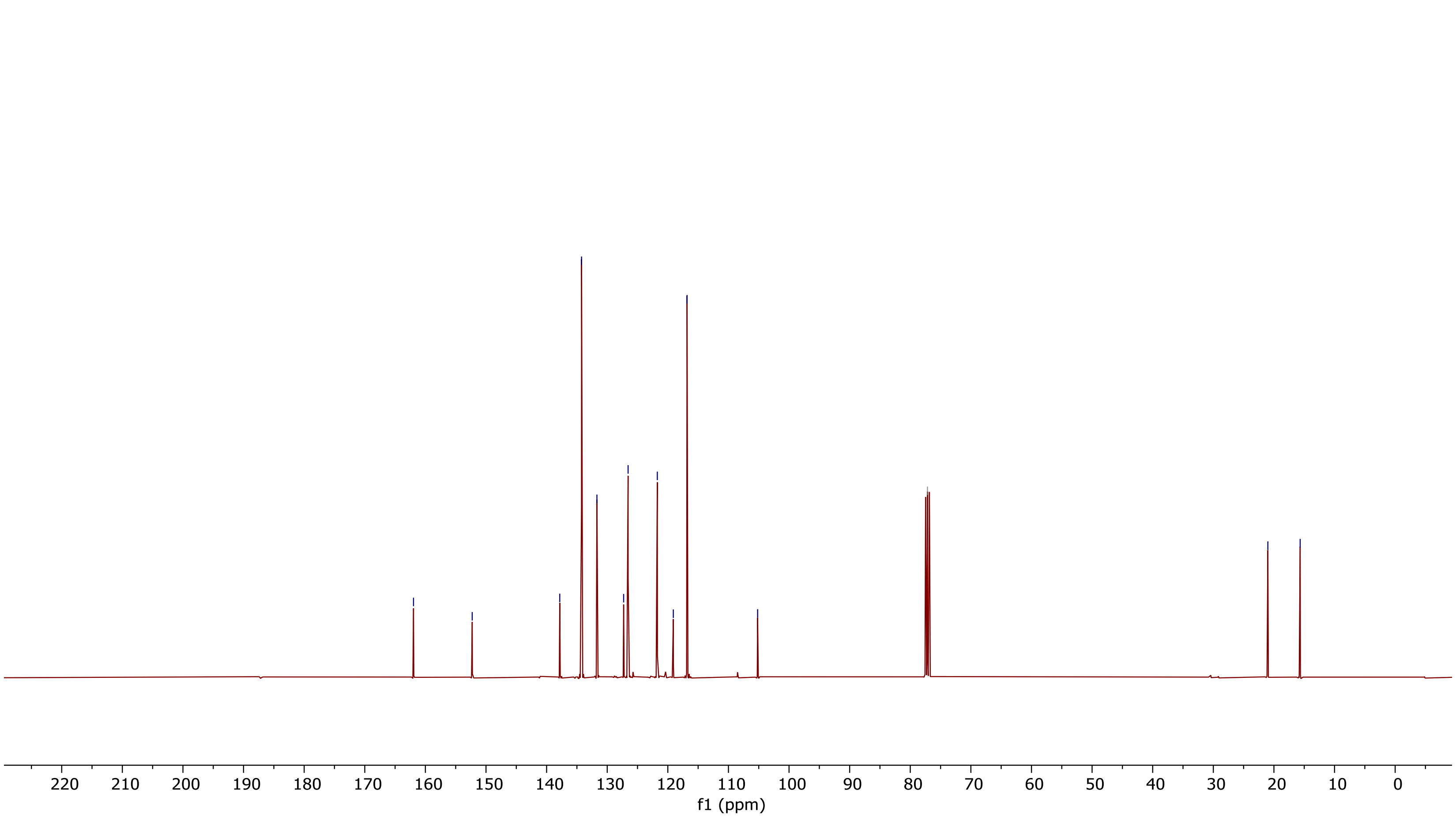

10 - ^{1}H NMR (400 MHz, $CDCl_3$)

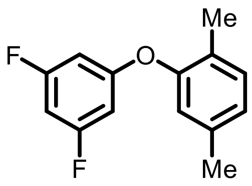

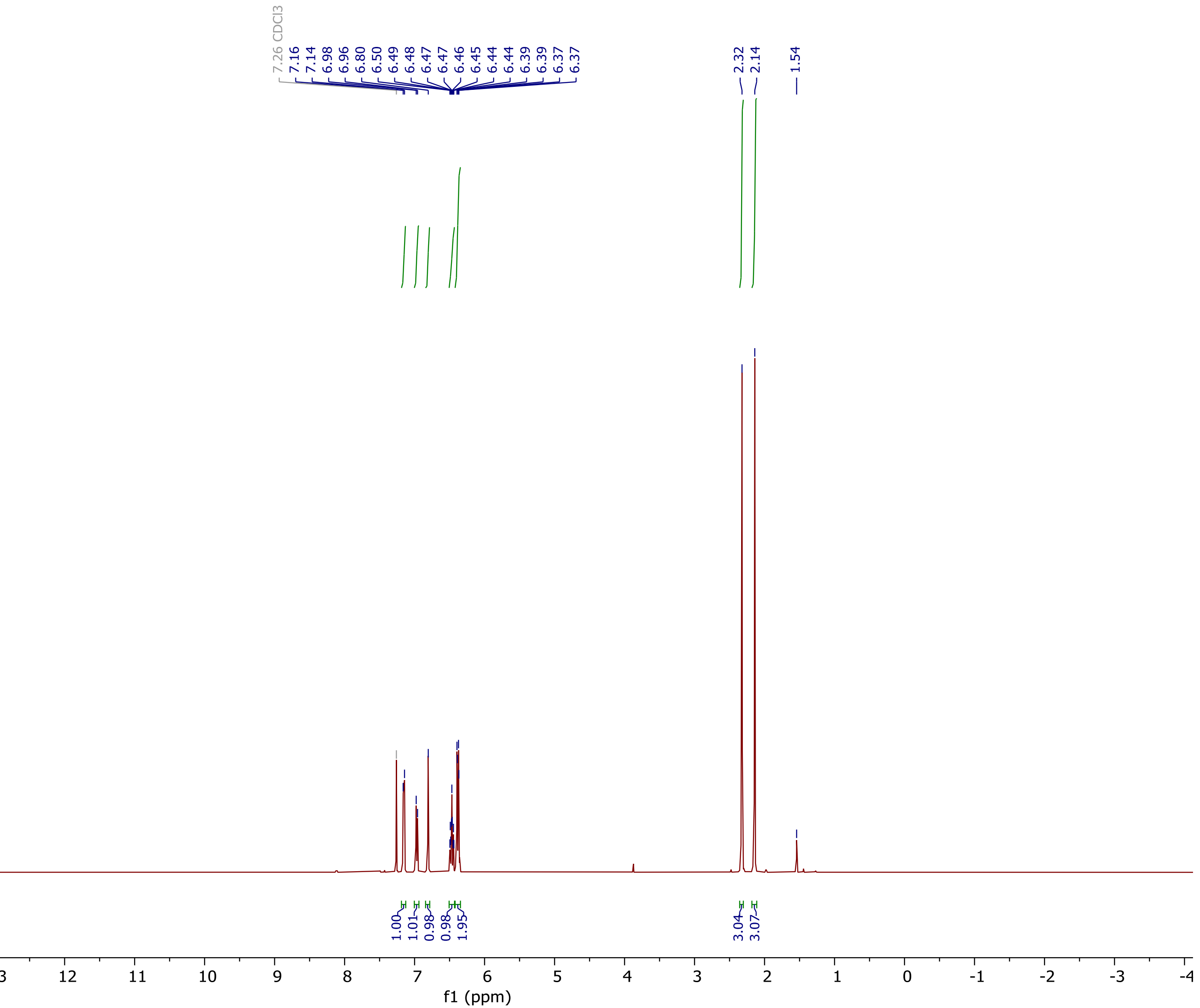

10 - $^{13}C\{^{1}H\}$ NMR (101 MHz, CDCl$_3$)

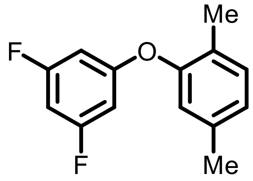

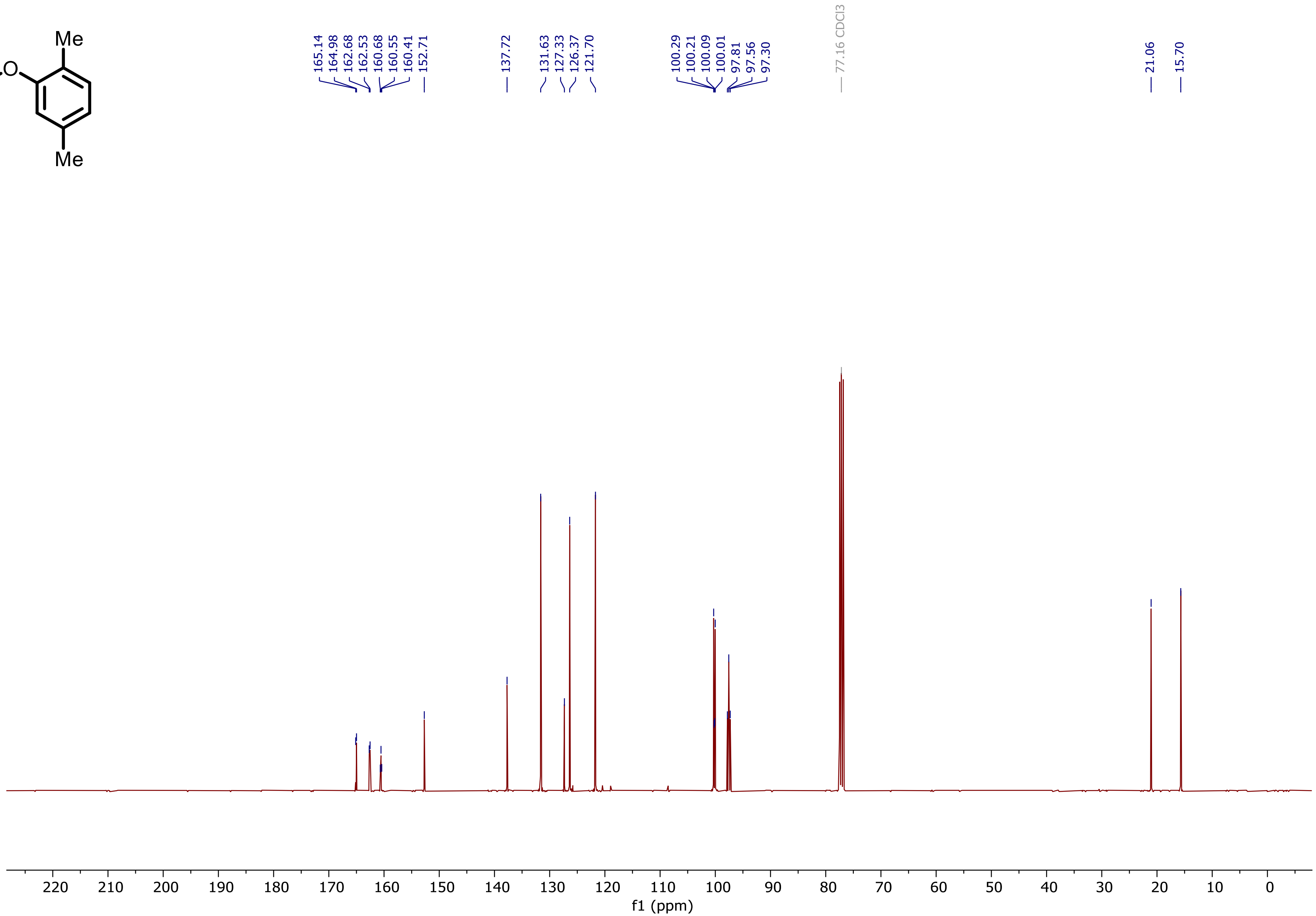

10 - ^{19}F NMR (377 MHz, $CDCl_3$)

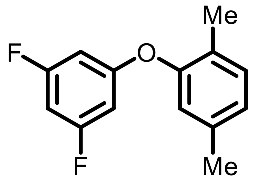

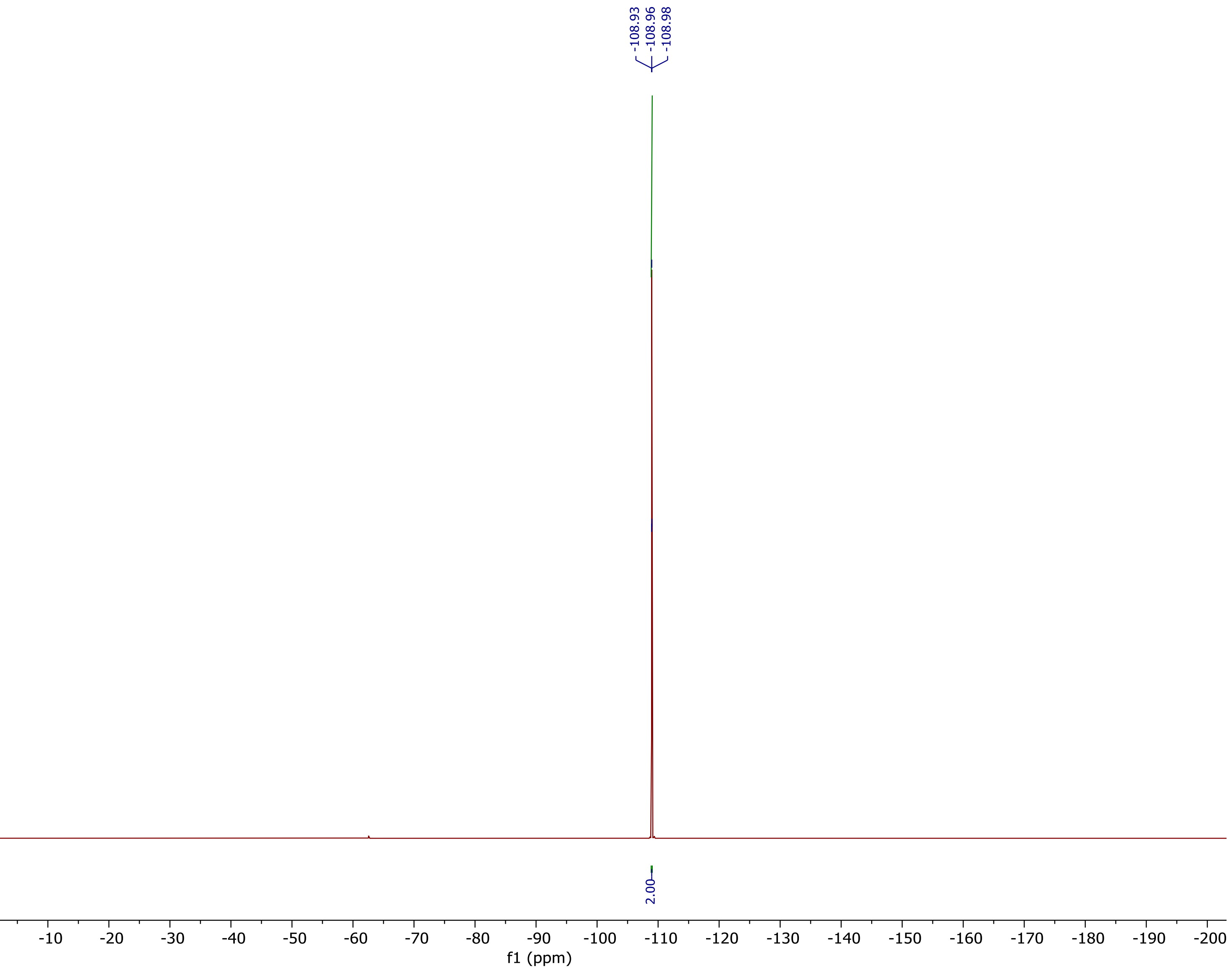

11 - ^{1}H NMR (500 MHz, $CDCl_3$)

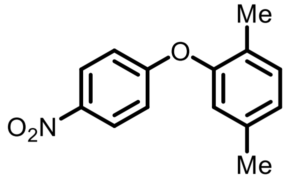

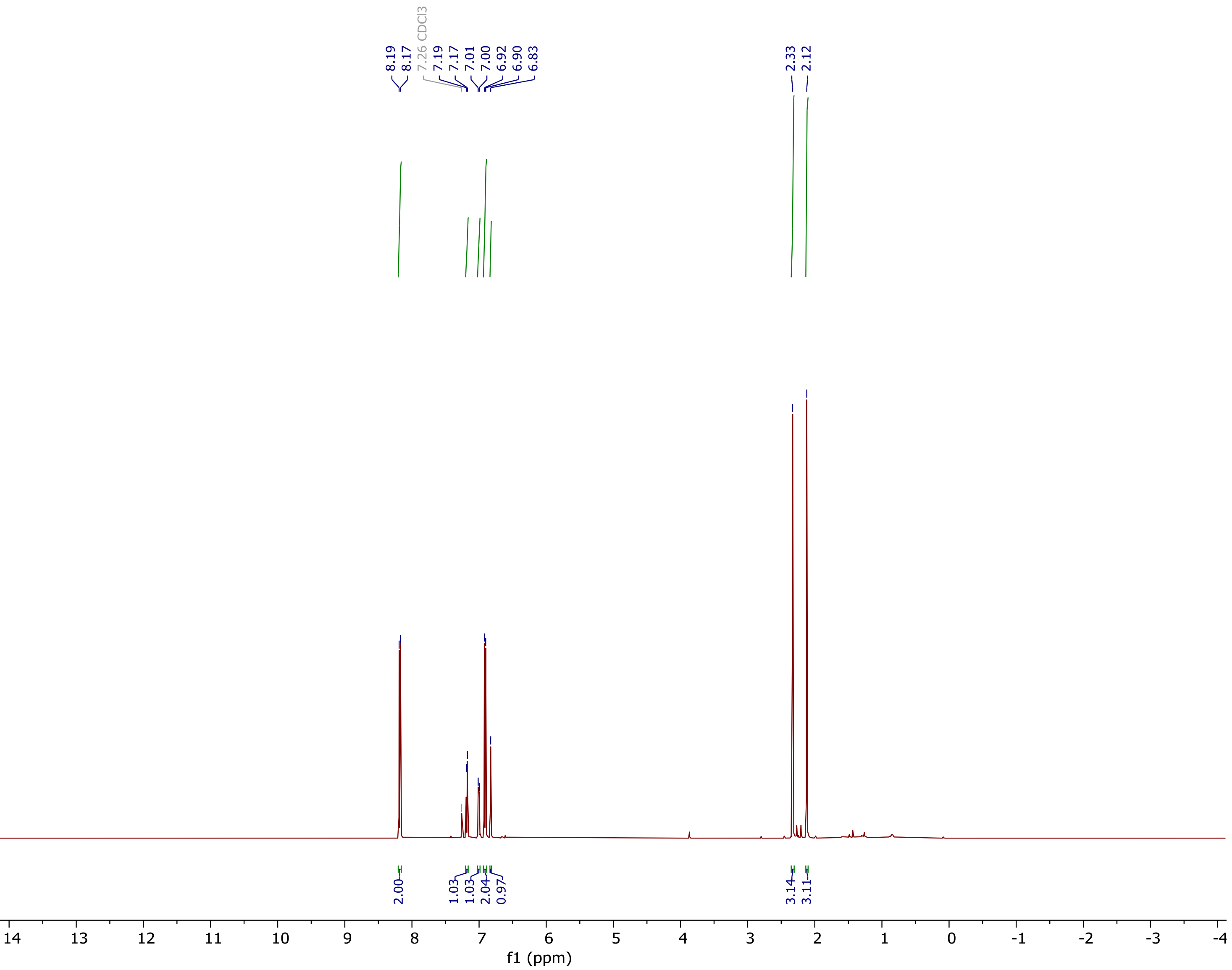

11 - $^{13}C\{^1H\}$ NMR (126 MHz, $CDCl_3$)

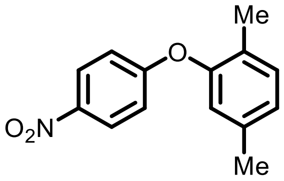

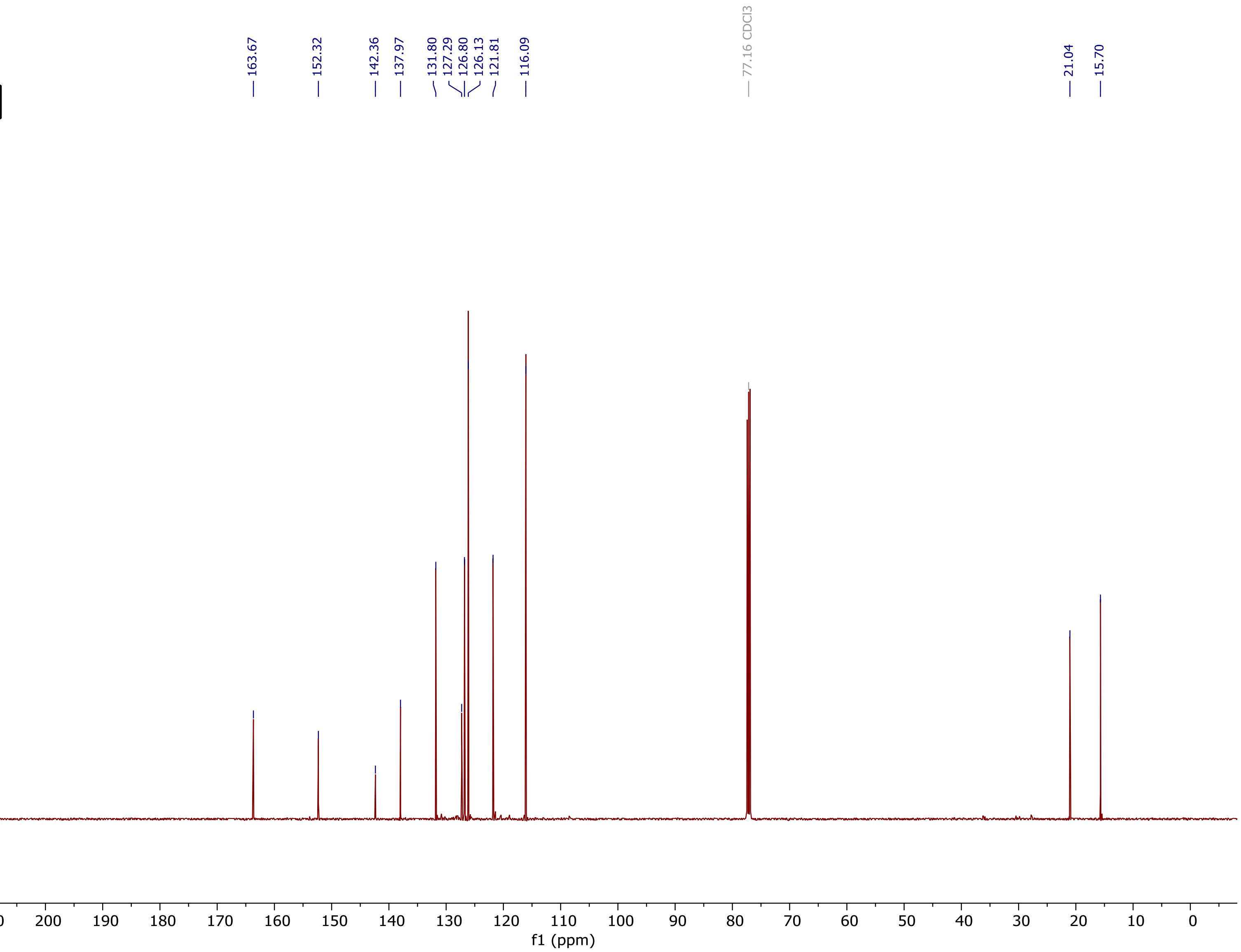

12 - ^{1}H NMR (400 MHz, $CDCl_3$)

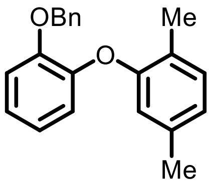

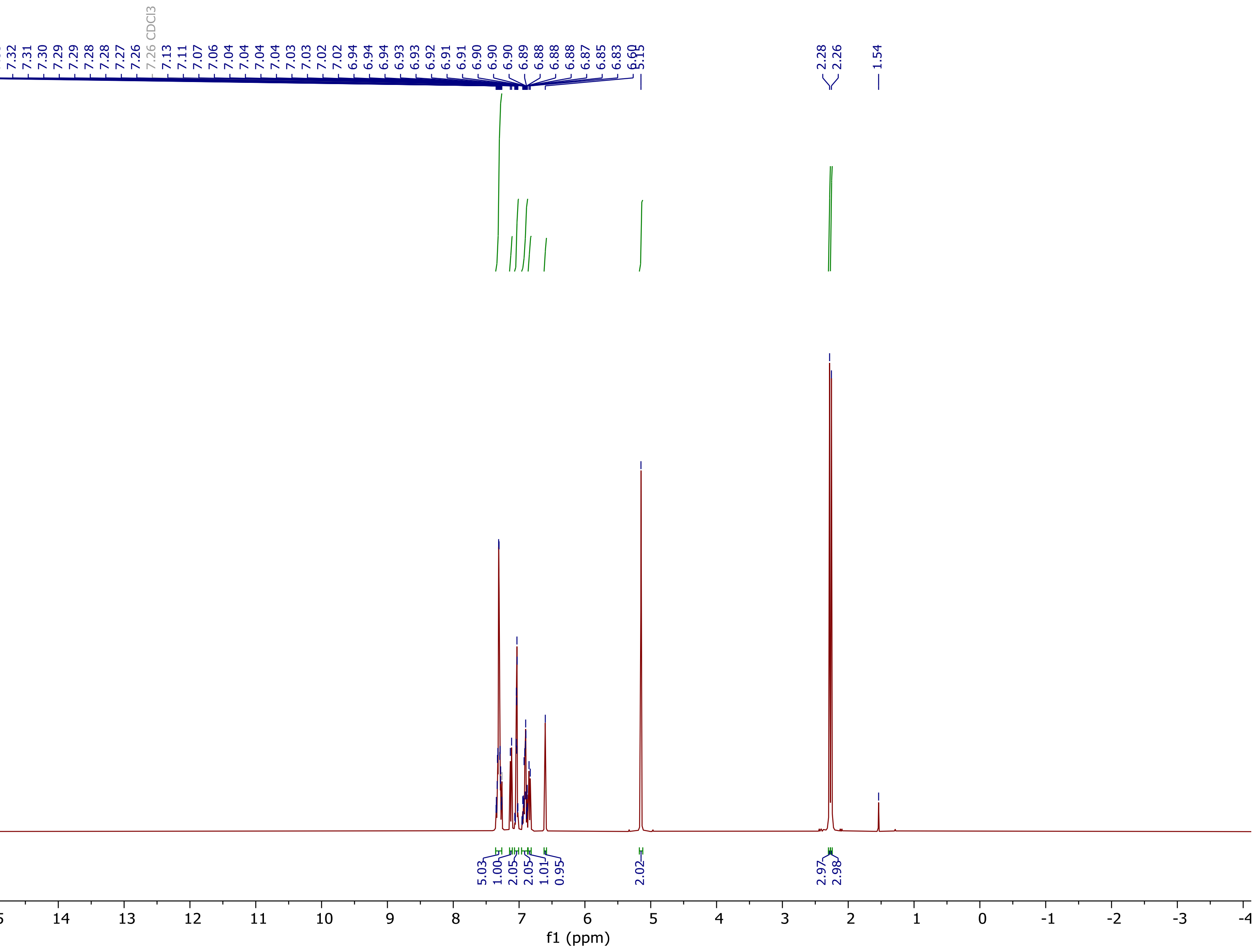

12 - $^{13}C\{^1H\}$ NMR (101 MHz, $CDCl_3$)

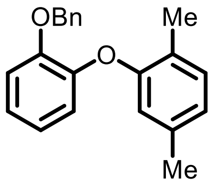

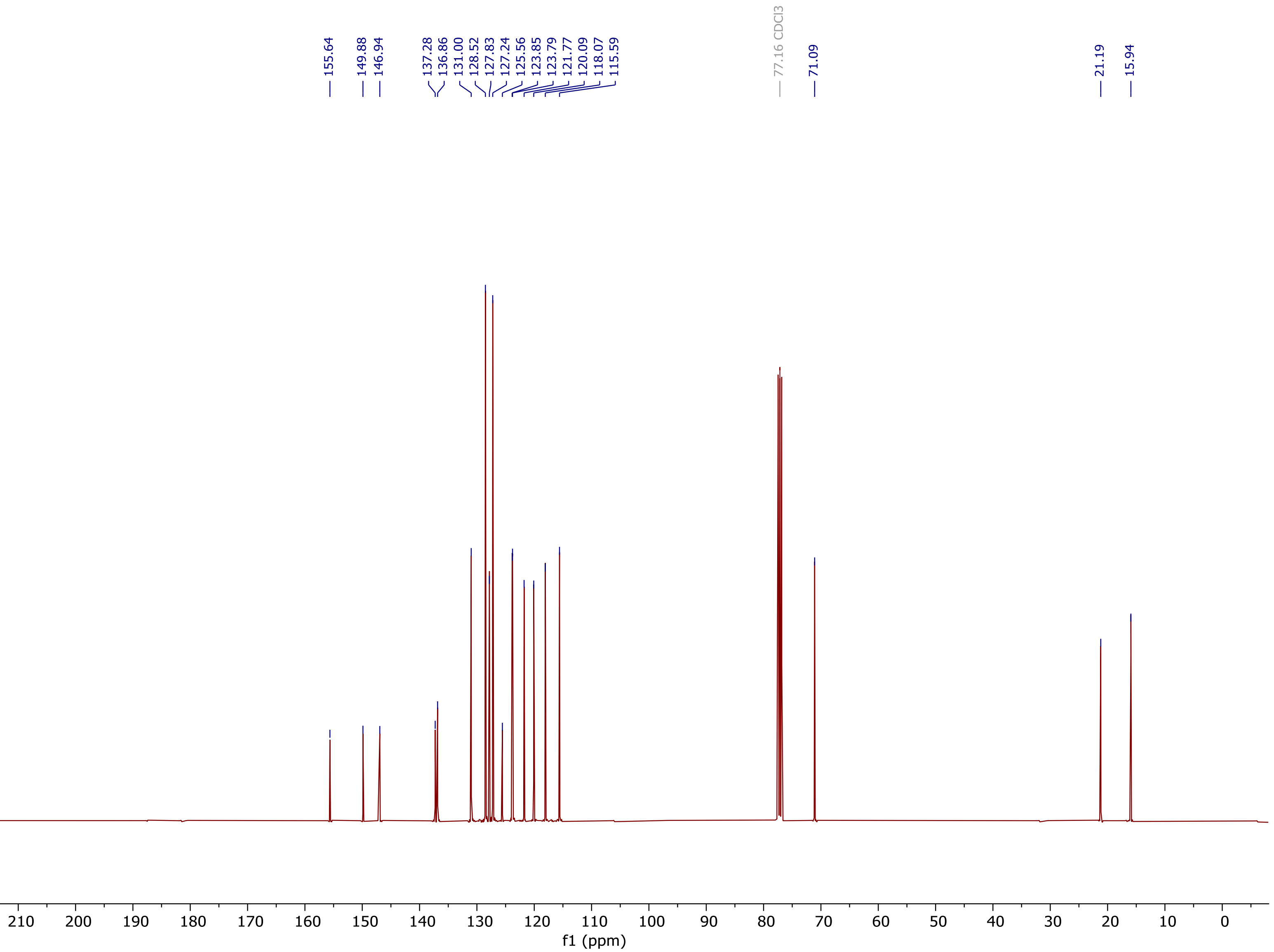

13 - ^{1}H NMR (400 MHz, $CDCl_3$)

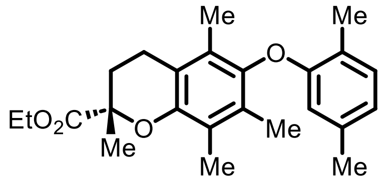

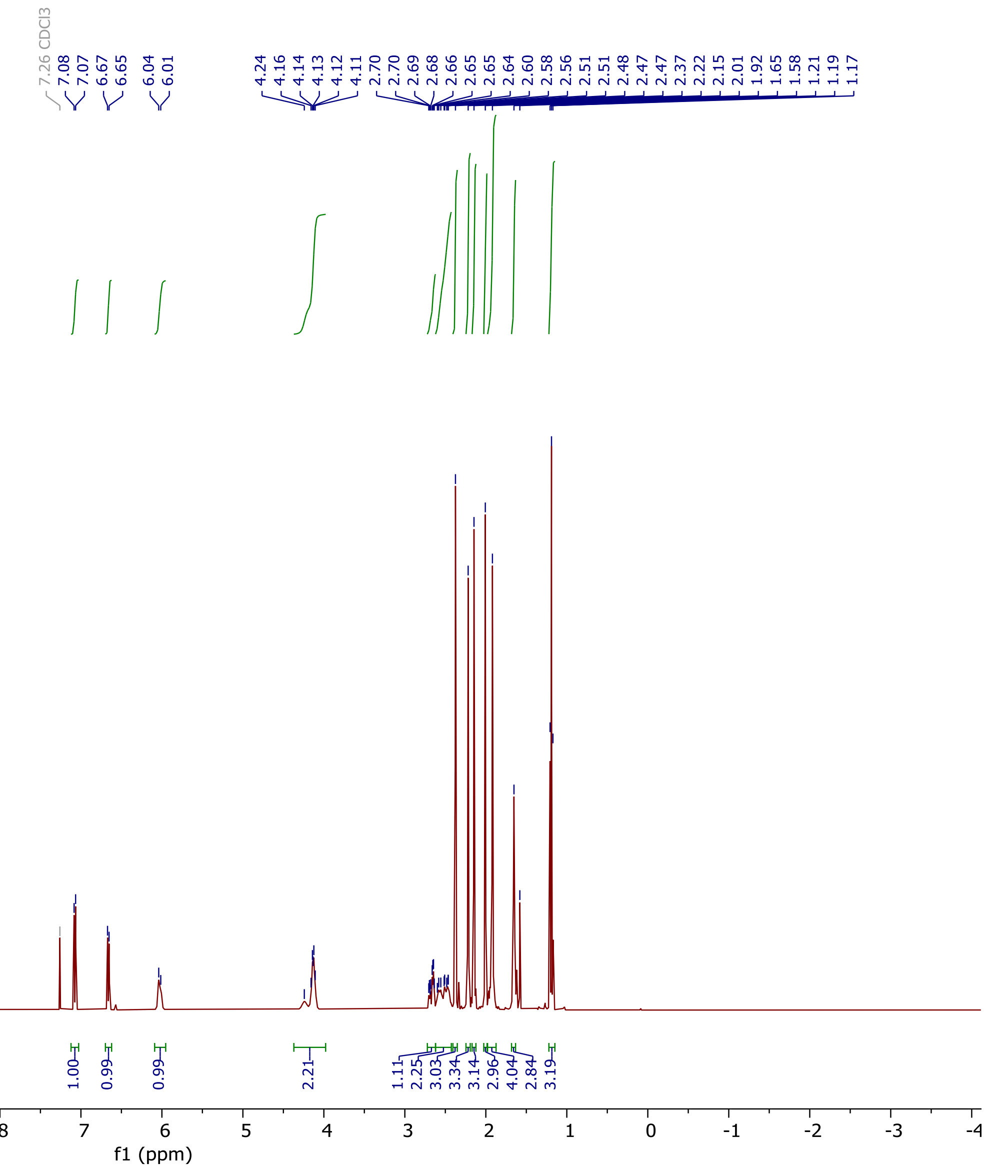

13 - $^{13}C\{^{1}H\}$ NMR (101 MHz, $CDCl_3$)

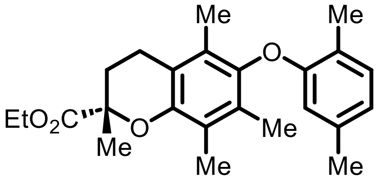

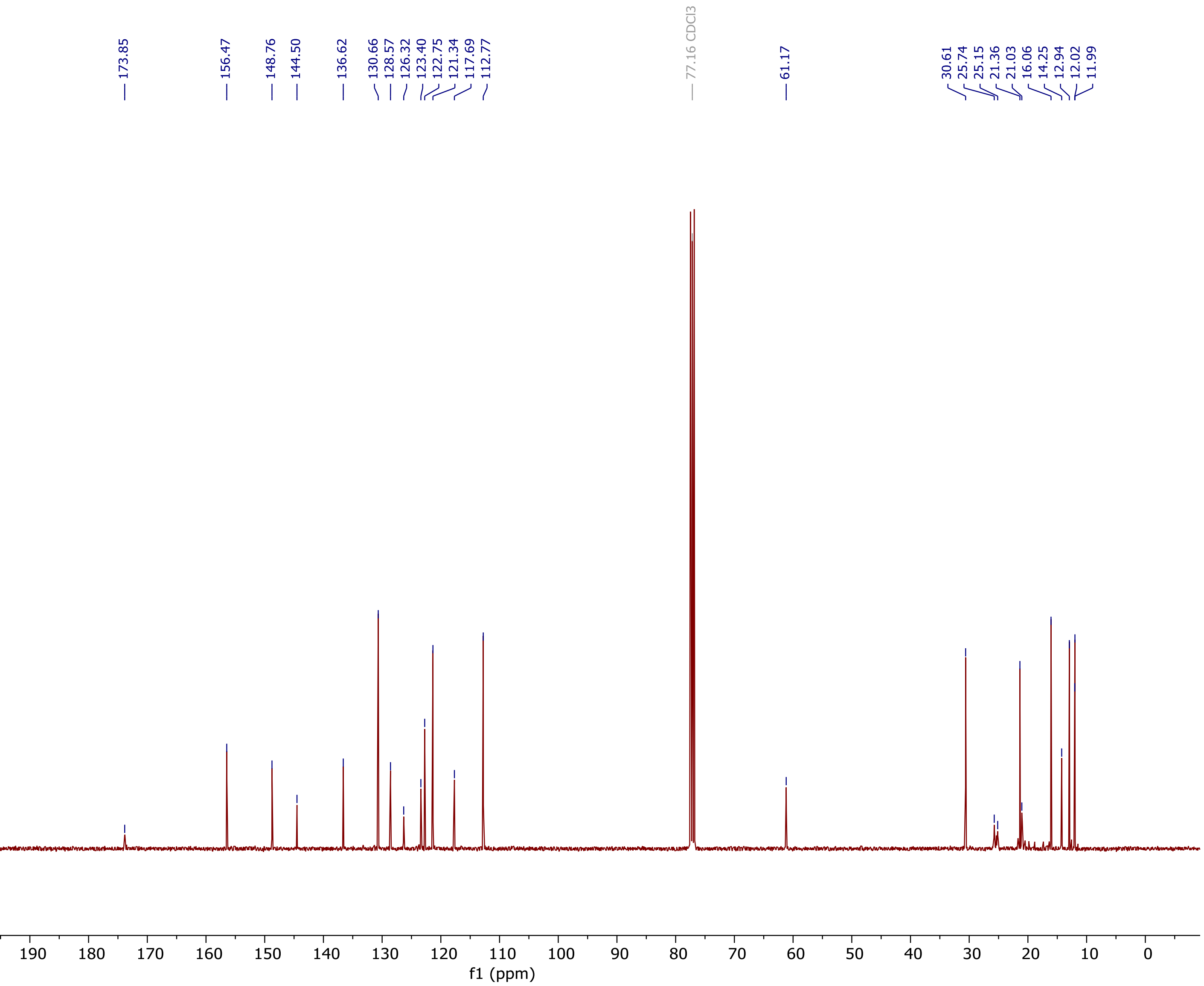

14 - ^{1}H NMR (500 MHz, $CDCl_3$)

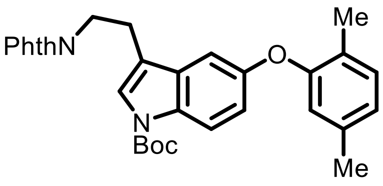

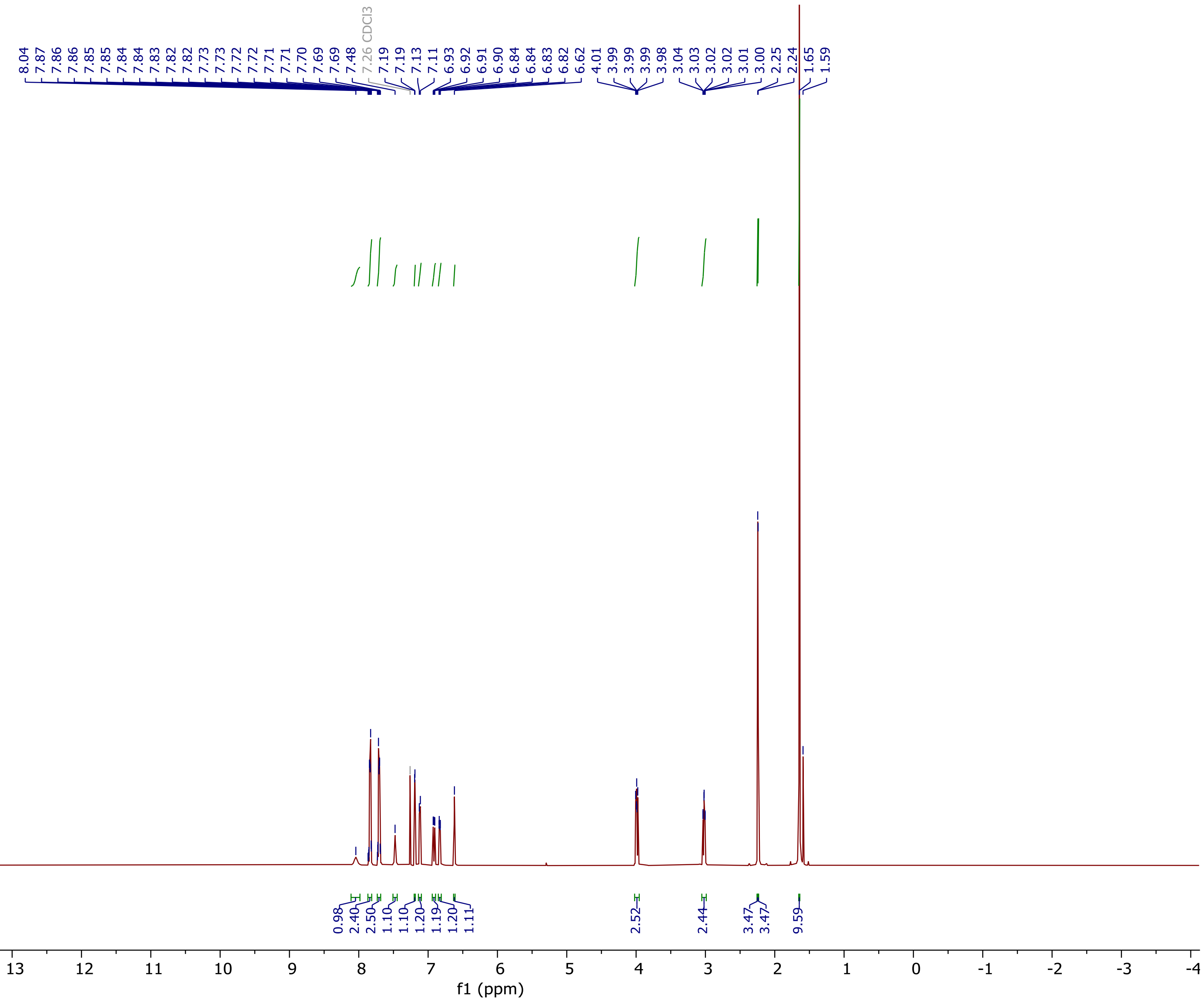

14 - $^{13}C\{^{1}H\}$ NMR (101 MHz, DMSO-d$_6$)

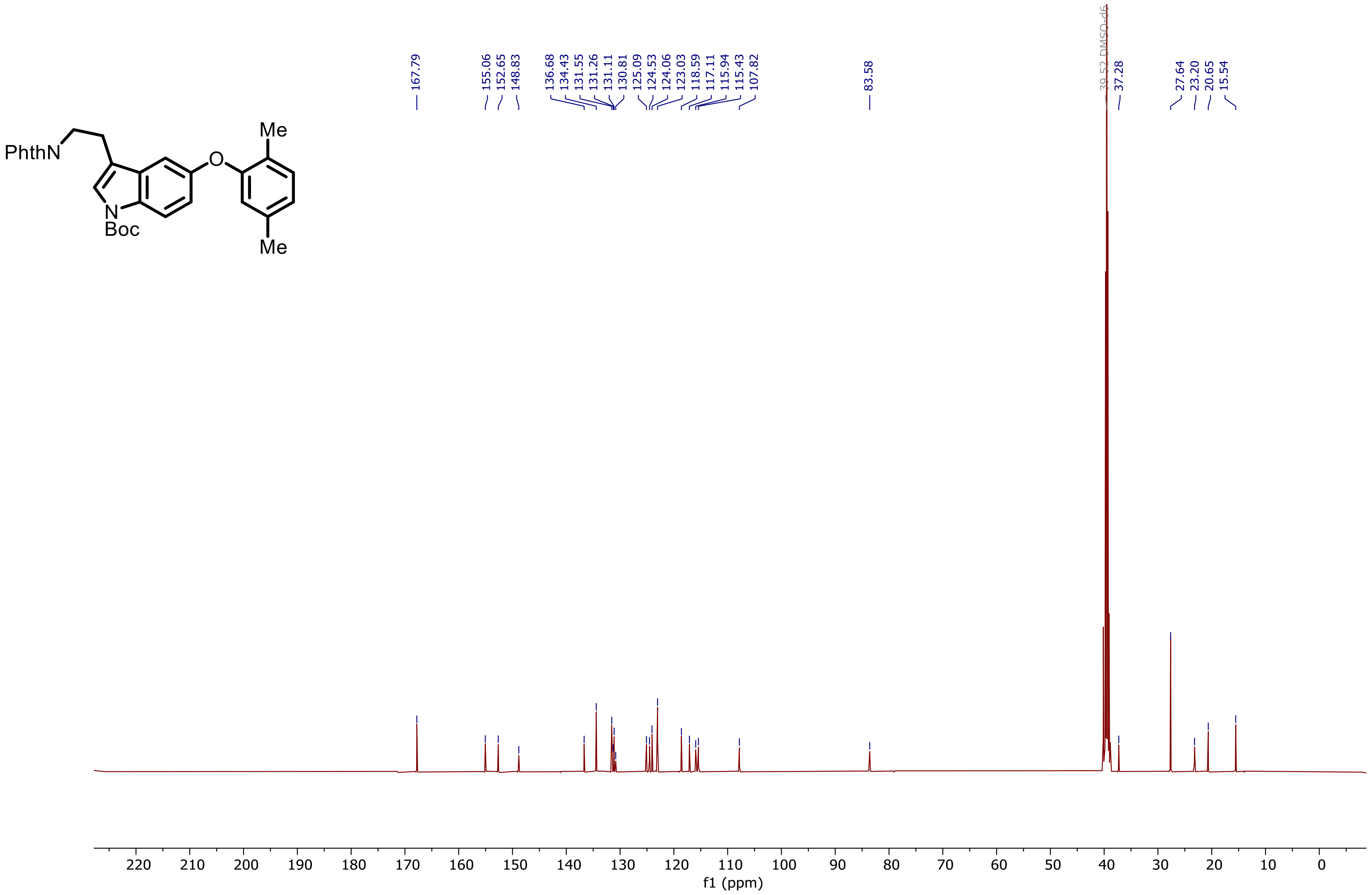

15 – ^{1}H NMR (400 MHz, $CDCl_3$)

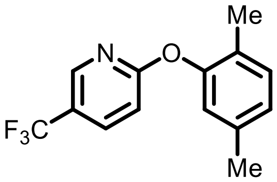

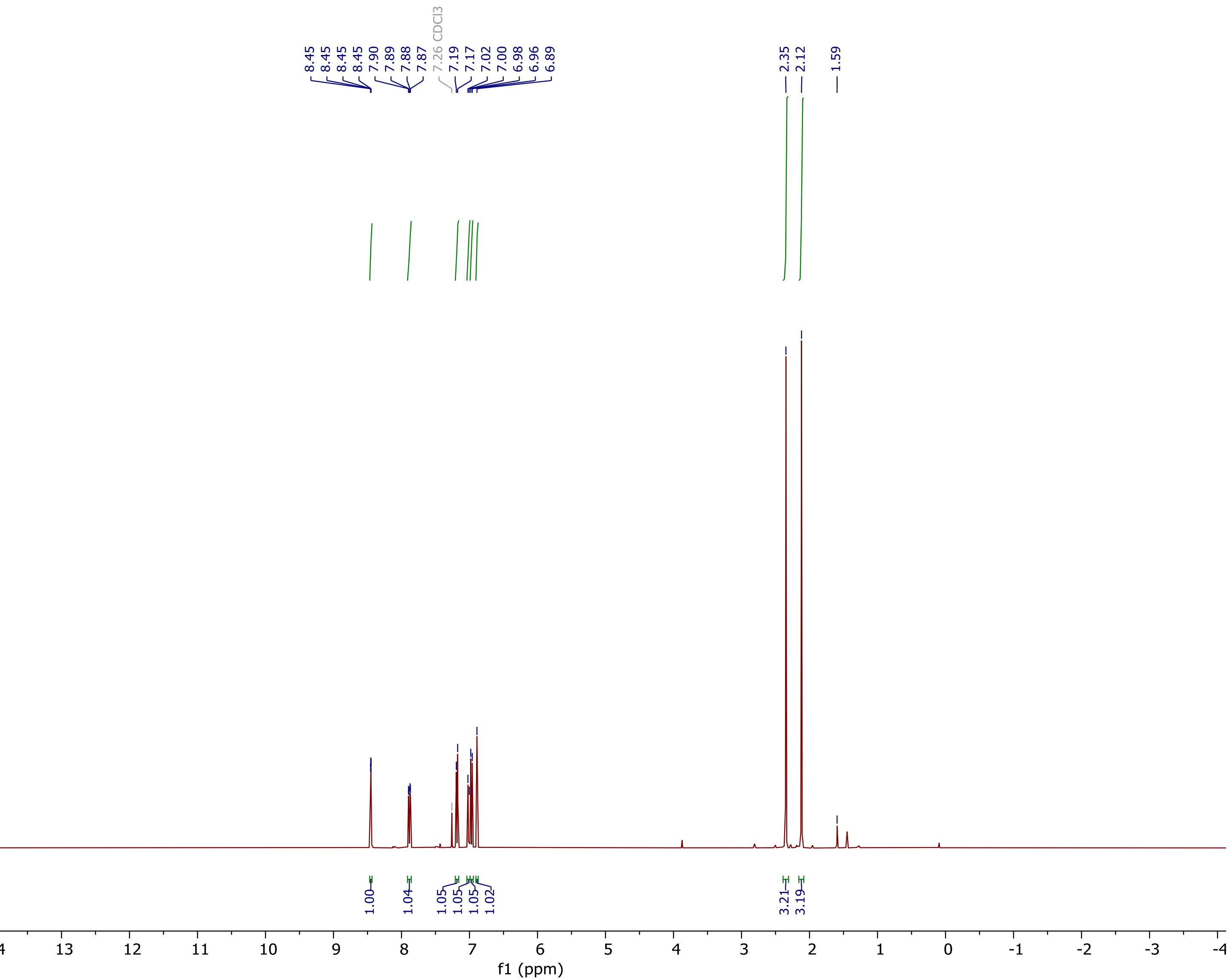

15 – ^{1}H NMR (400 MHz, $CDCl_3$)

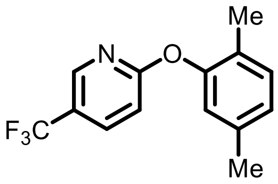

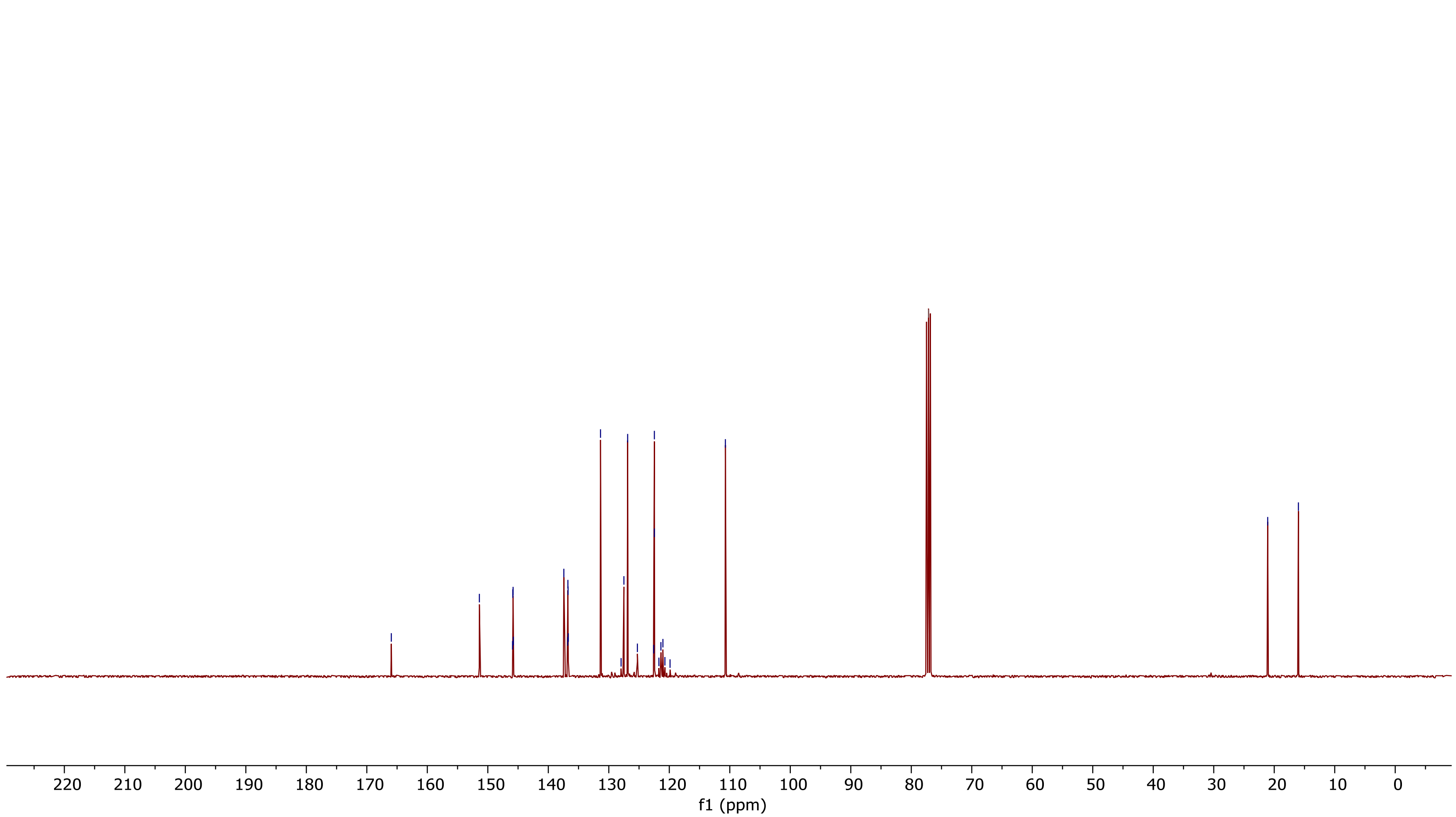

15 – ^{19}F NMR (376 MHz, $CDCl_3$)

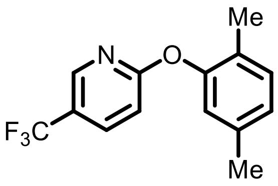

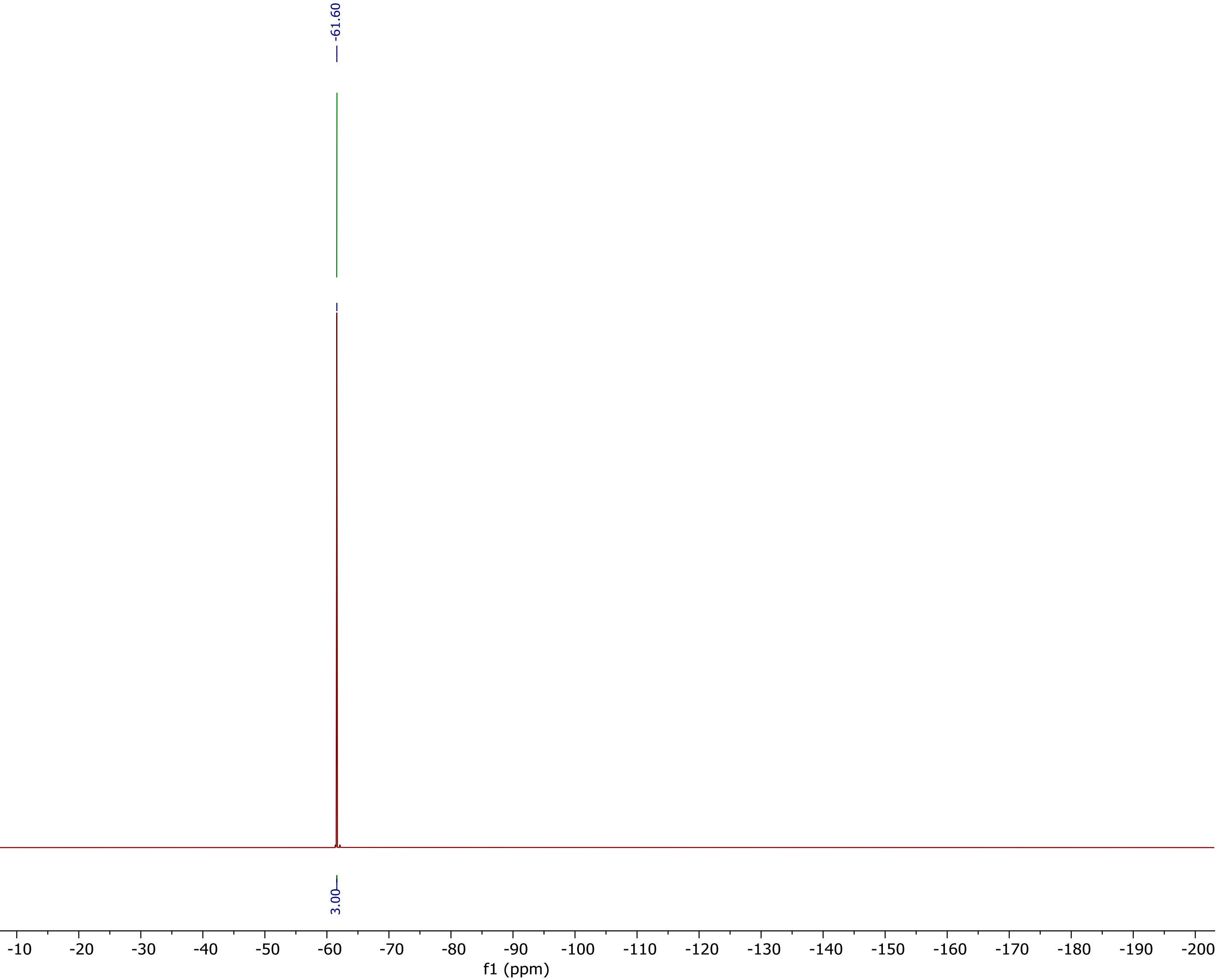

16 - ^{1}H NMR (400 MHz, $CDCl_3$)

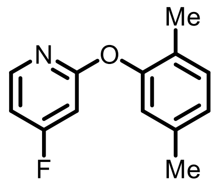

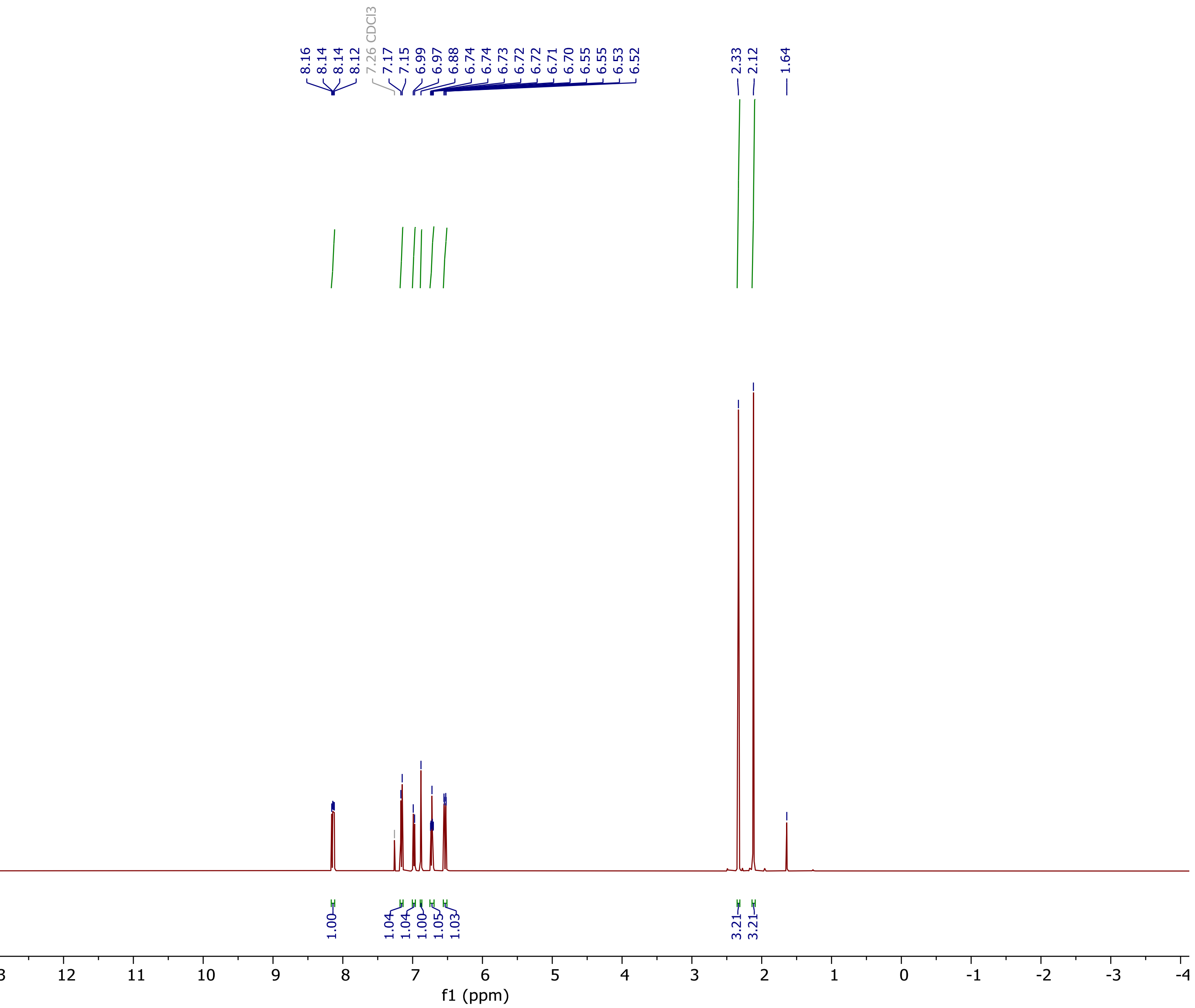

16 - $^{13}C\{^1H\}$ NMR (101 MHz, CDCl$_3$)

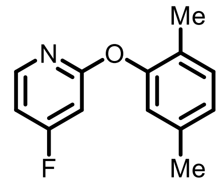

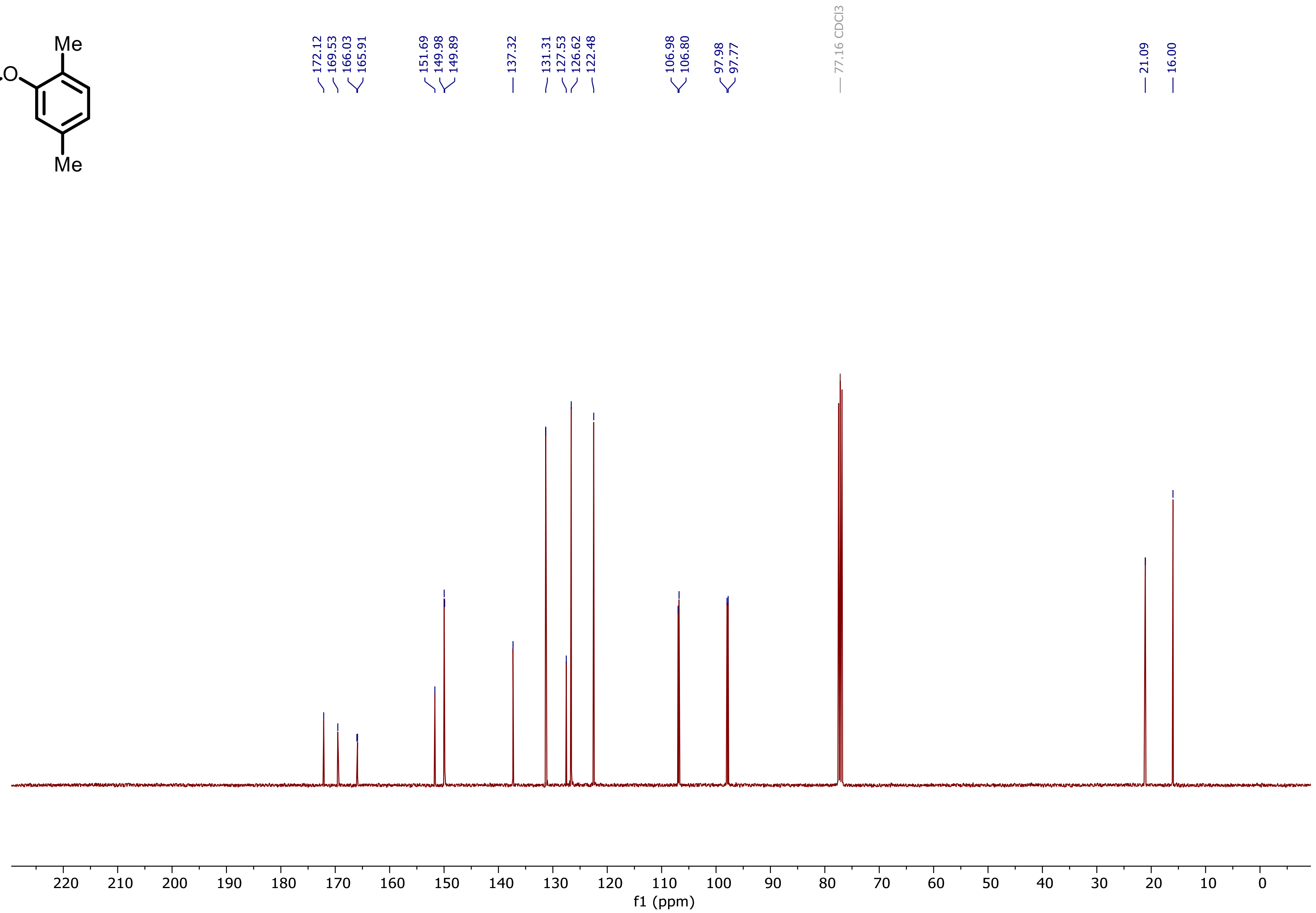

16 - ^{19}F NMR (376 MHz, $CDCl_3$)

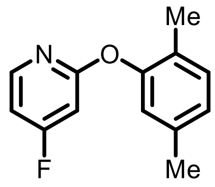

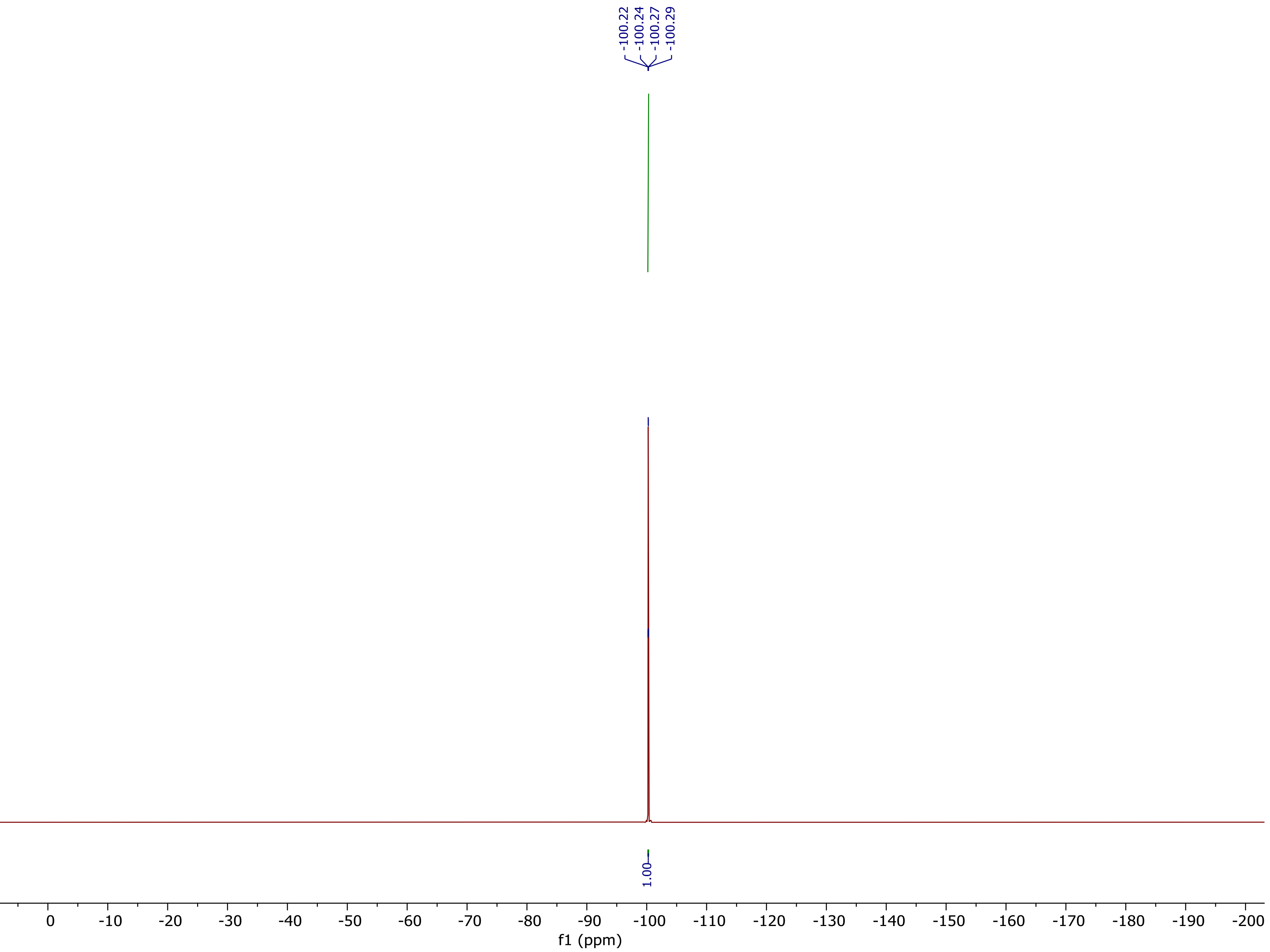

17 - ^{1}H NMR (400 MHz, $CDCl_3$)

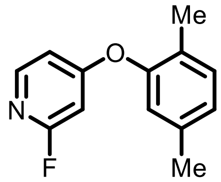

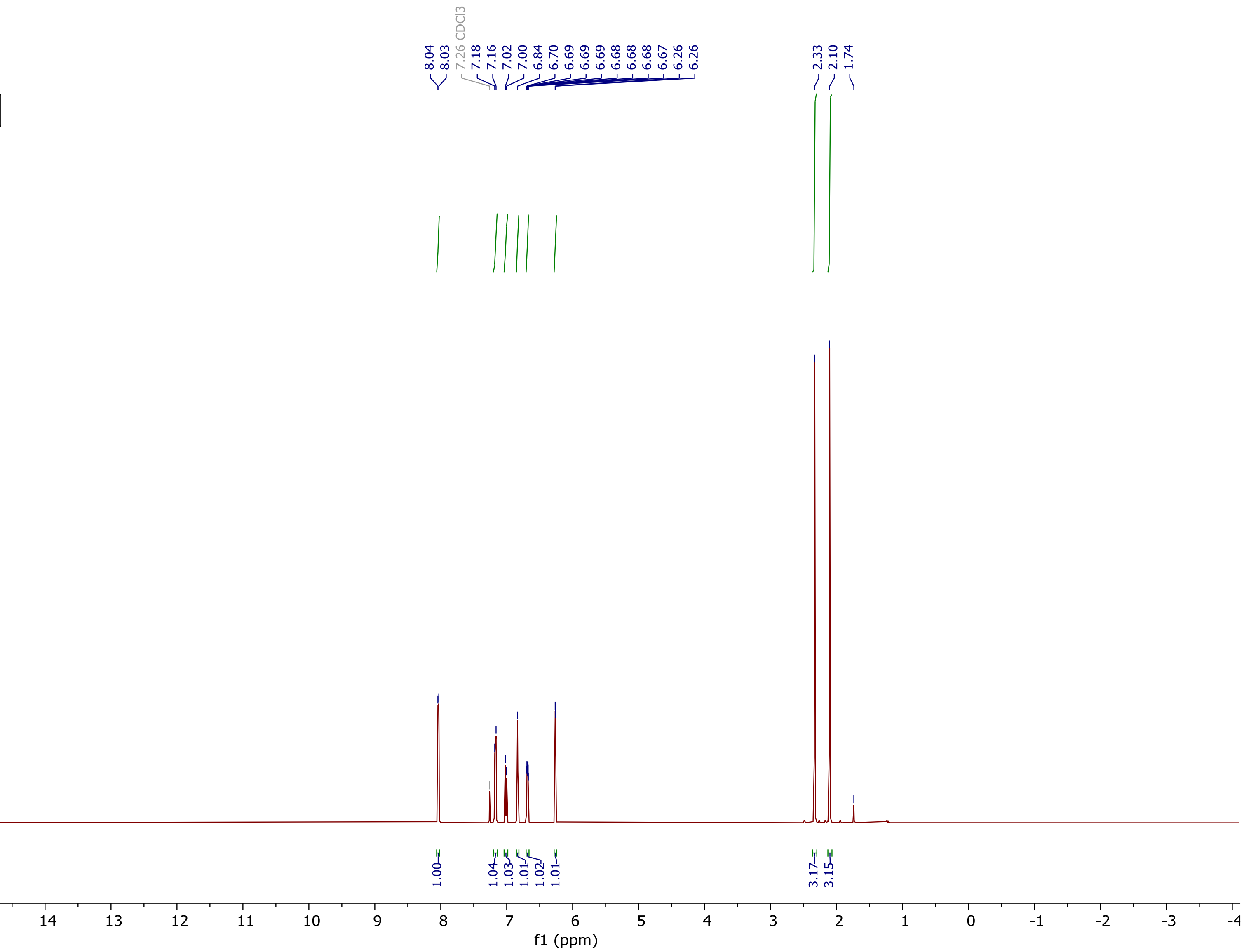

17 - $^{13}C\{^{1}H\}$ NMR (101 MHz, $CDCl_3$)

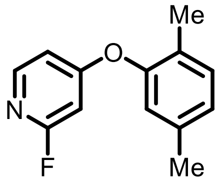

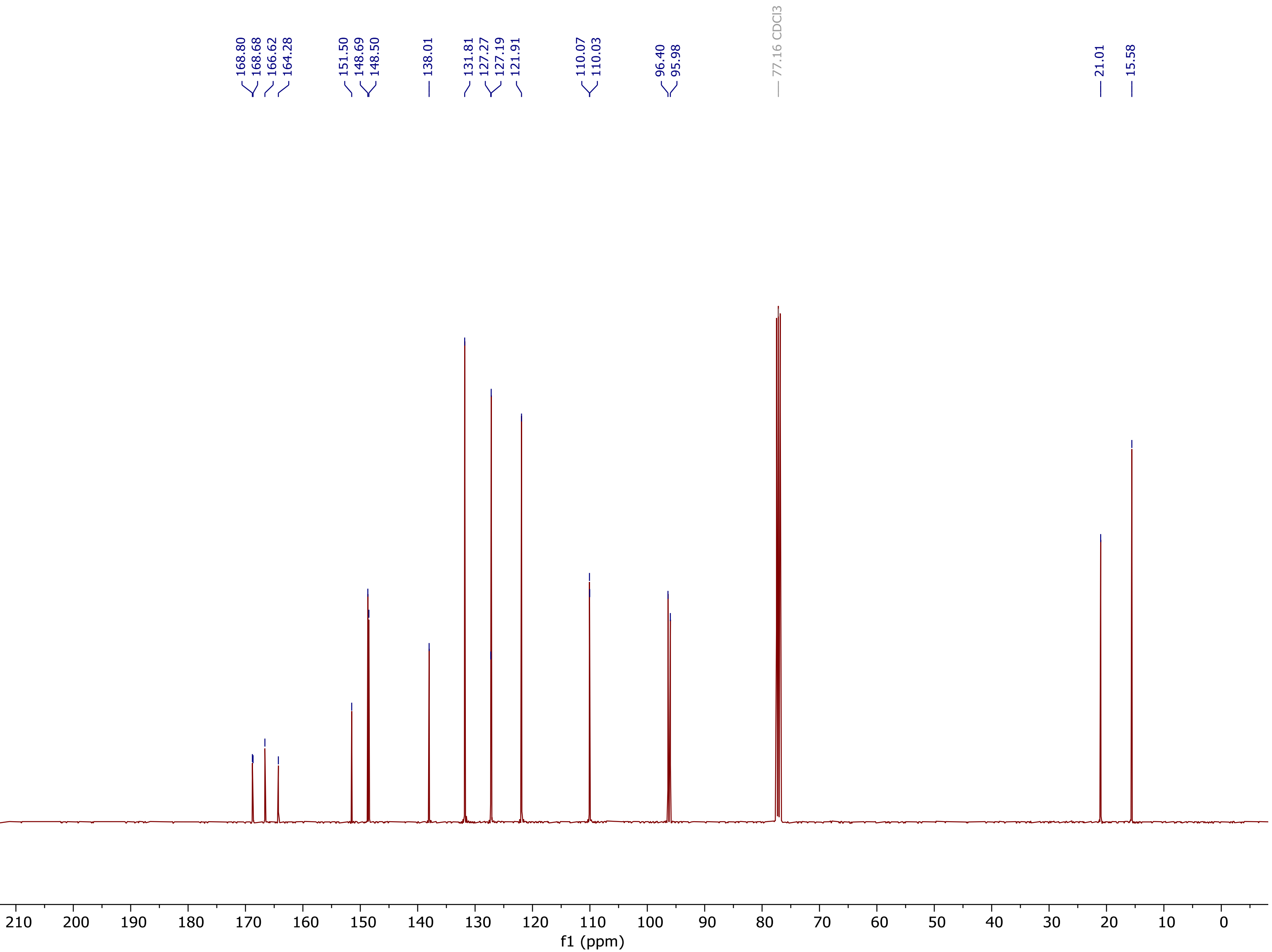

17 - ^{19}F NMR (376 MHz, $CDCl_3$)

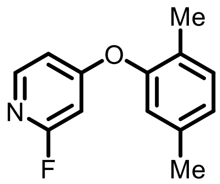

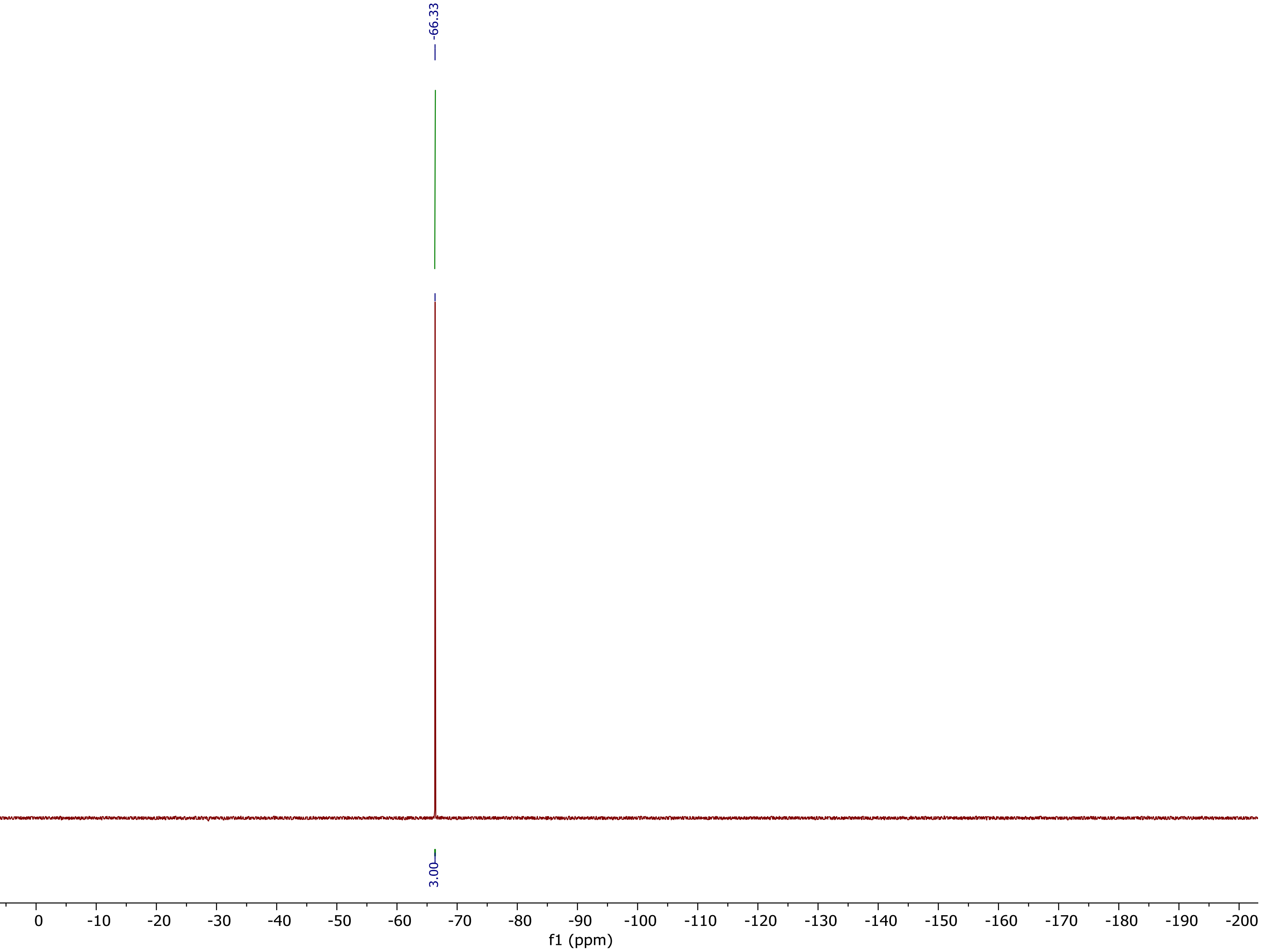

18 - ^{1}H NMR (500 MHz, $CDCl_3$)

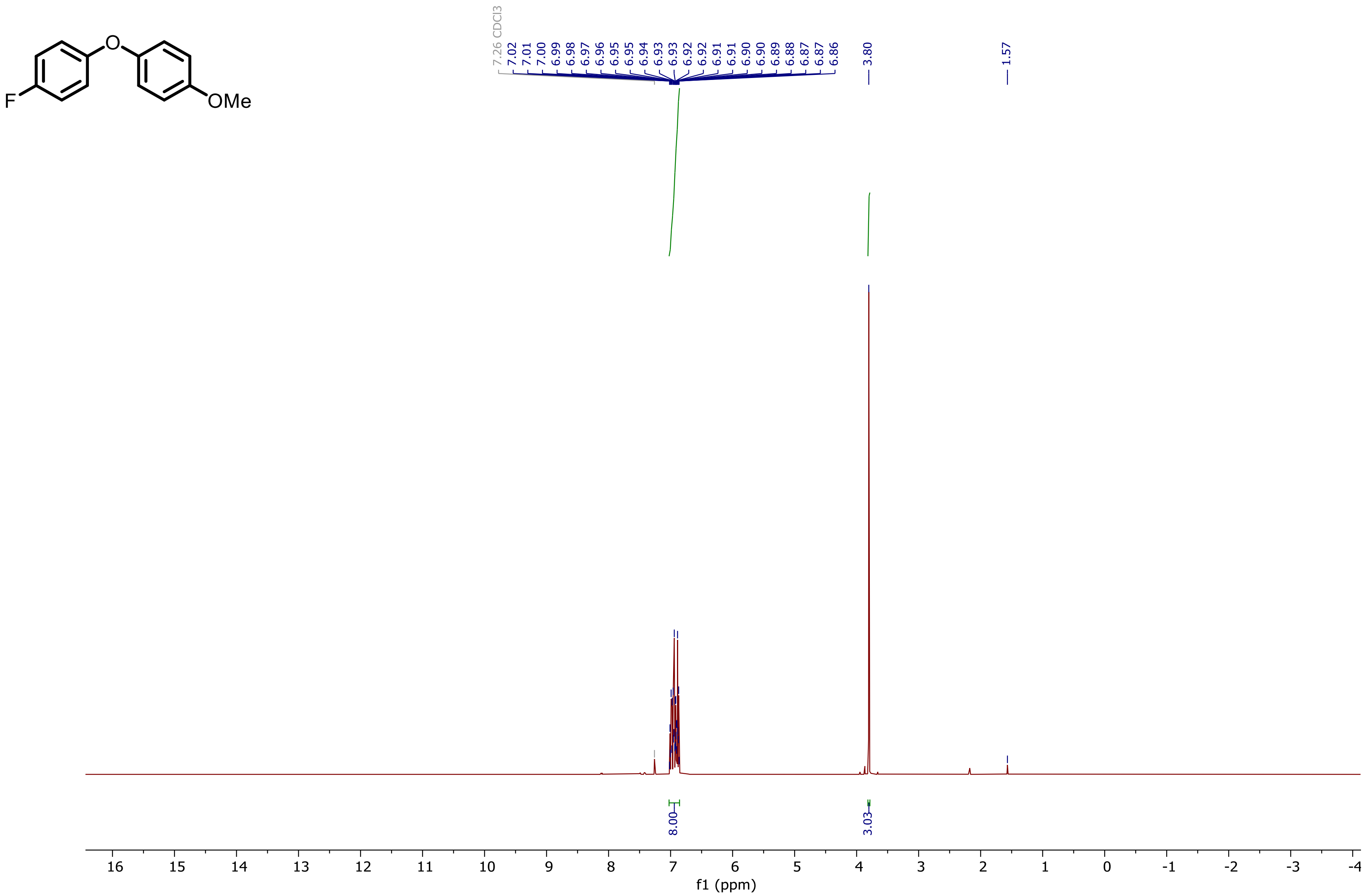

18 - $^{13}C\{^1H\}$ NMR (126 MHz, $CDCl_3$)

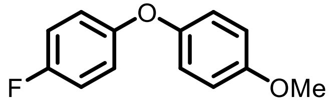

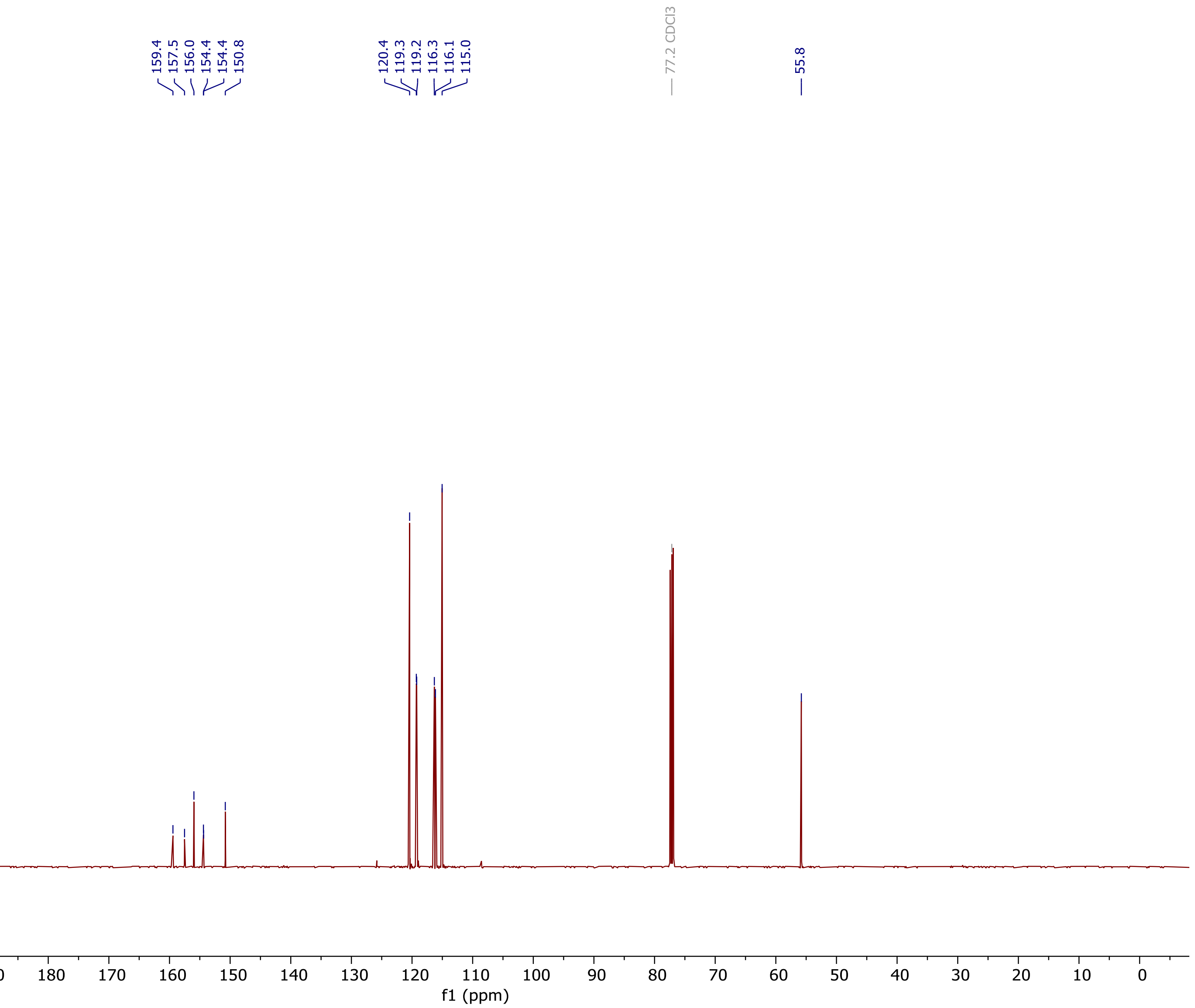

18 - ^{19}F NMR (376 MHz, $CDCl_3$)

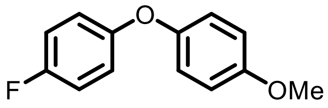

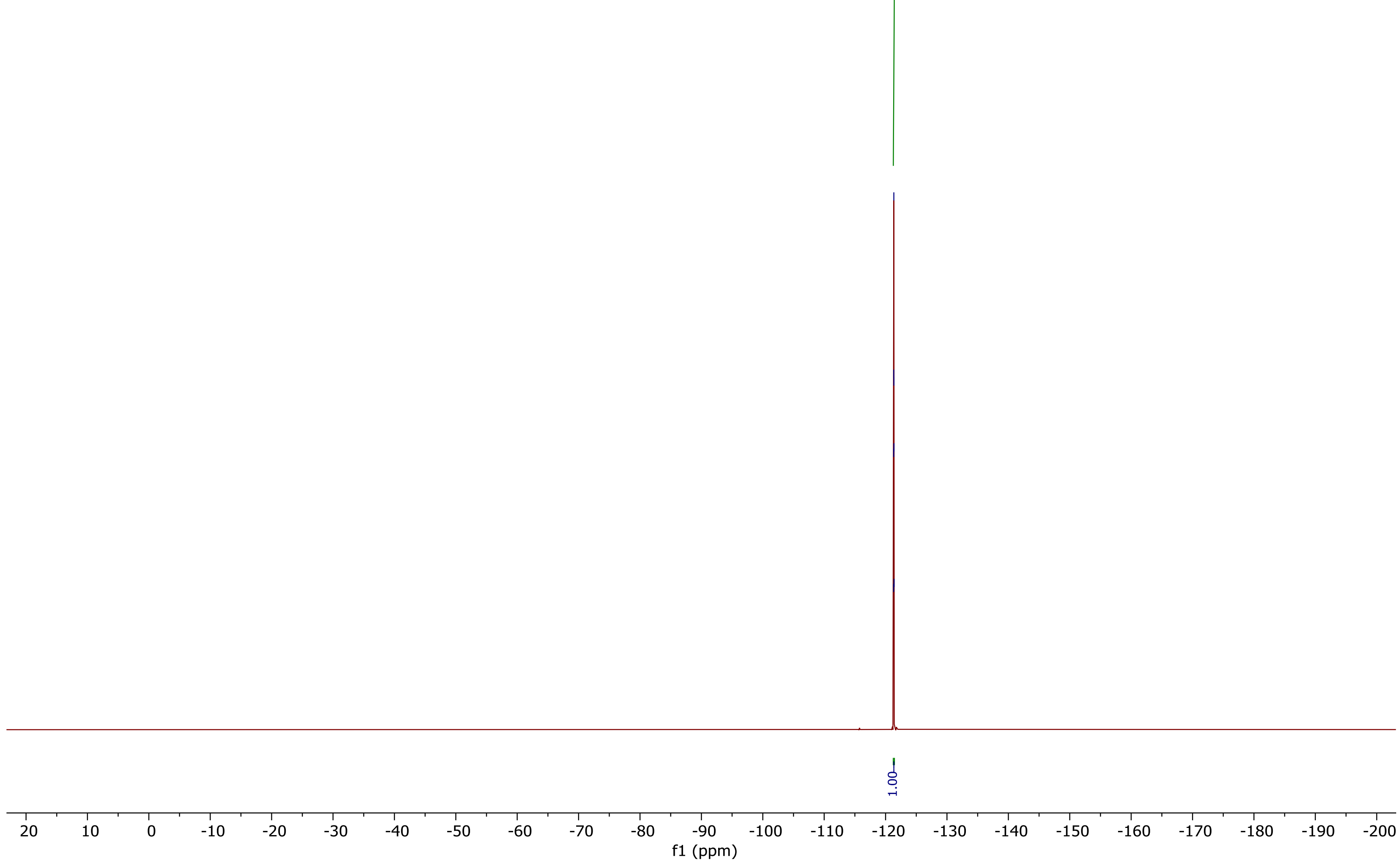

19 - ^{1}H NMR (400 MHz, $CDCl_3$)

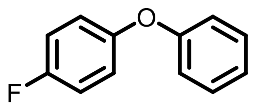

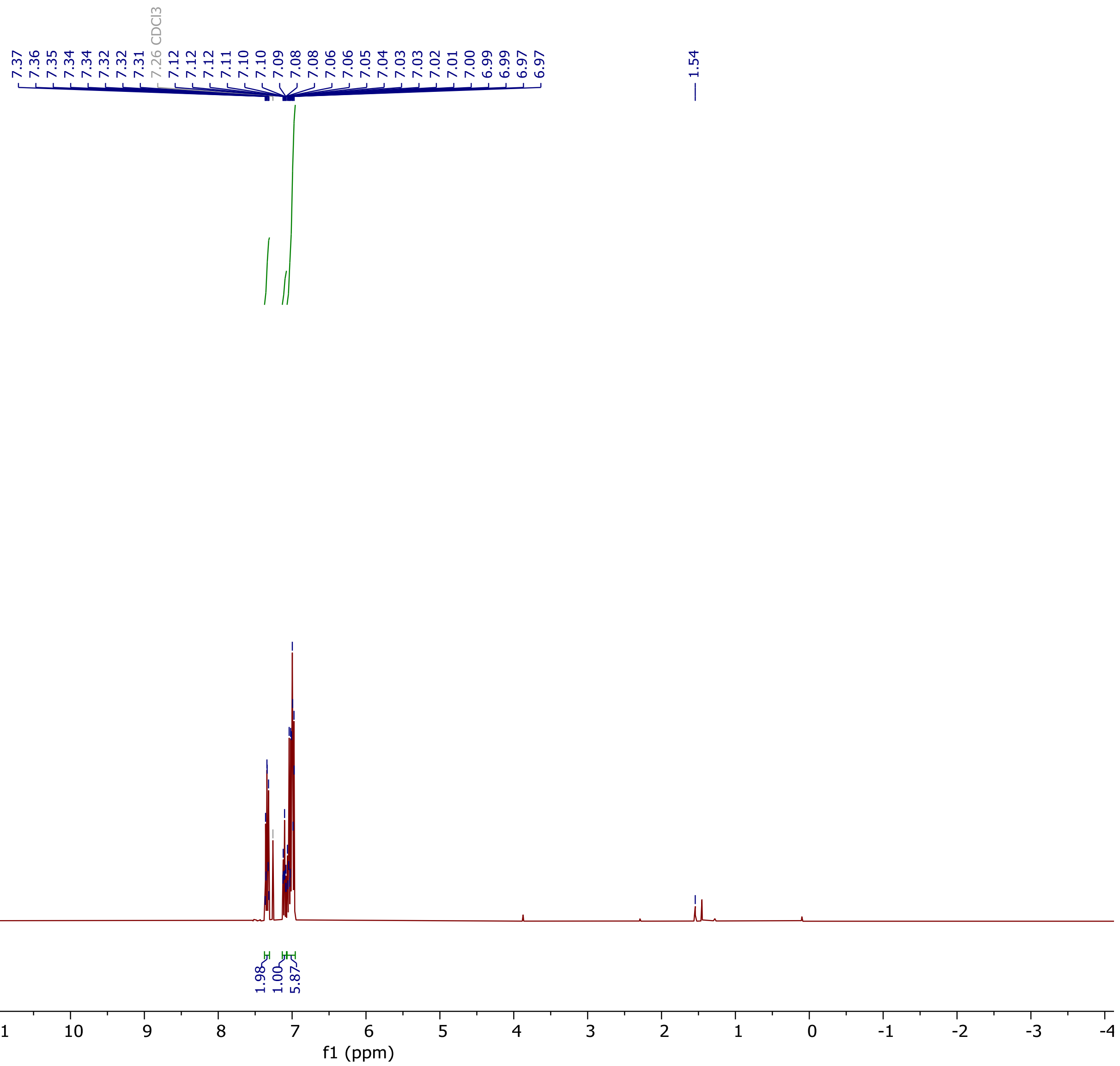

19 - $^{13}C\{^1H\}$ NMR (101 MHz, $CDCl_3$)

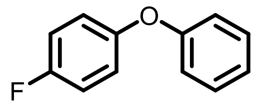

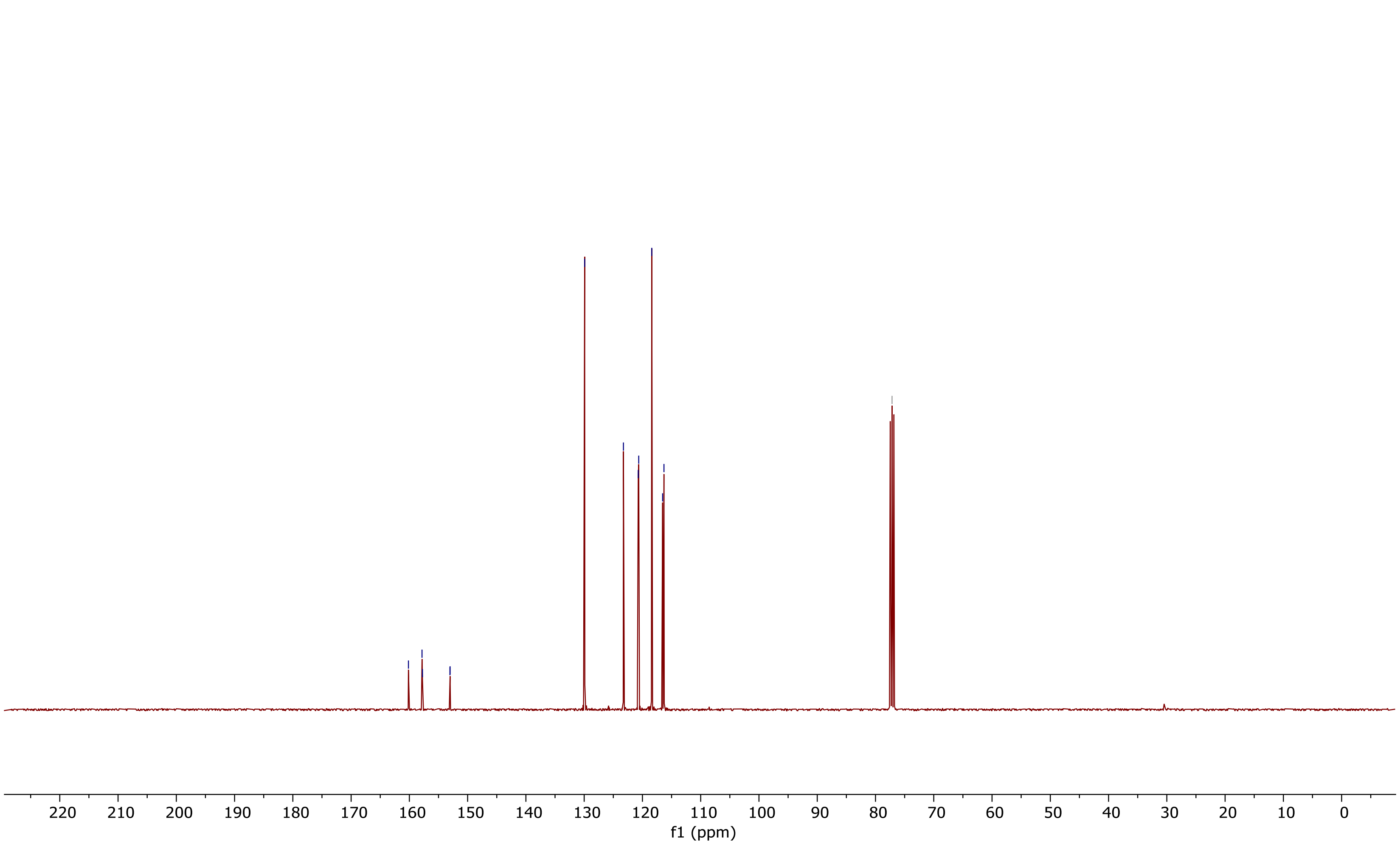

19 - ^{19}F NMR (376 MHz, $CDCl_3$)

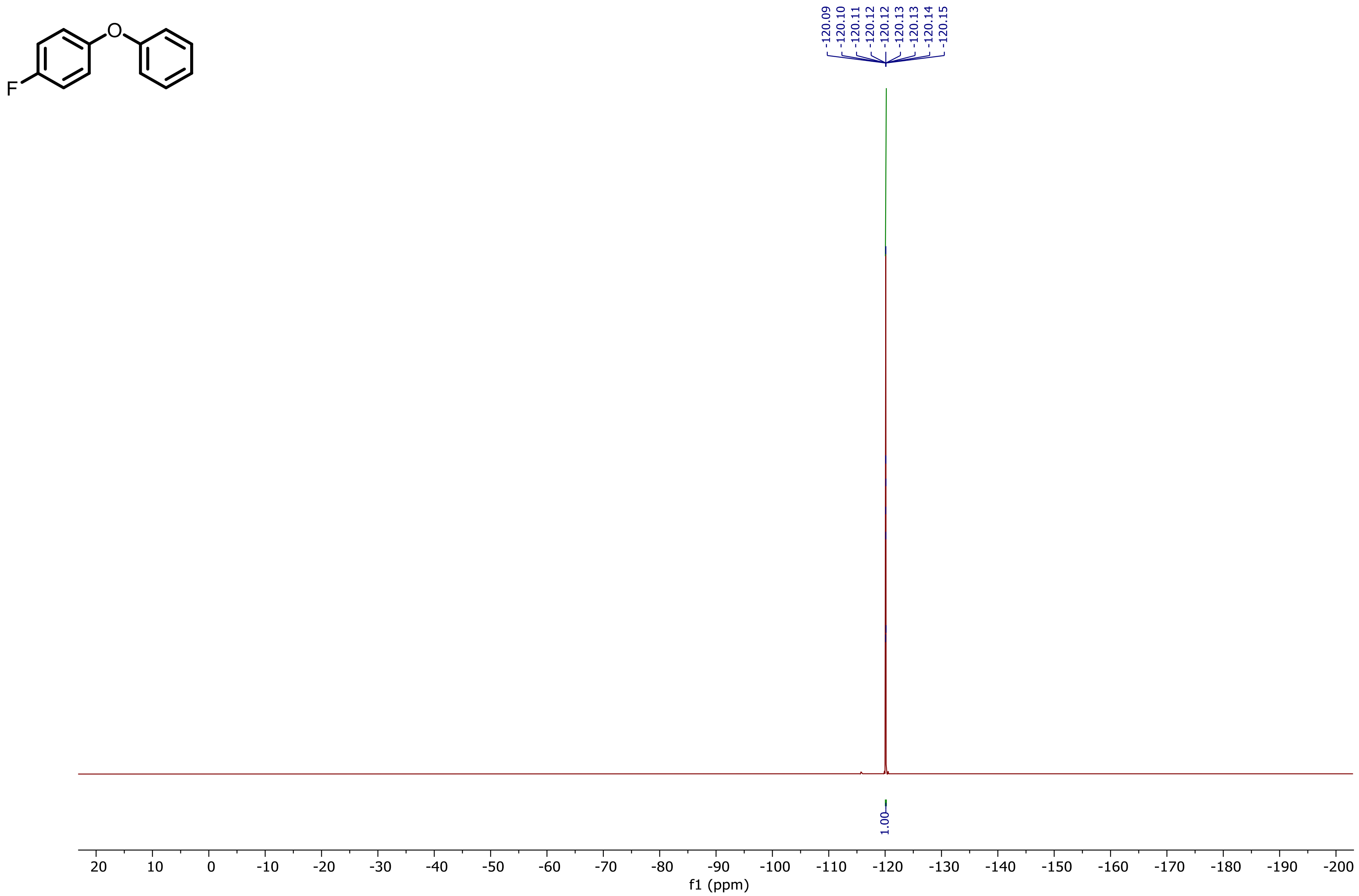

20 – ^{1}H NMR (400 MHz, $CDCl_3$)

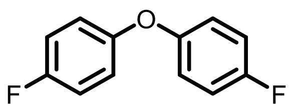

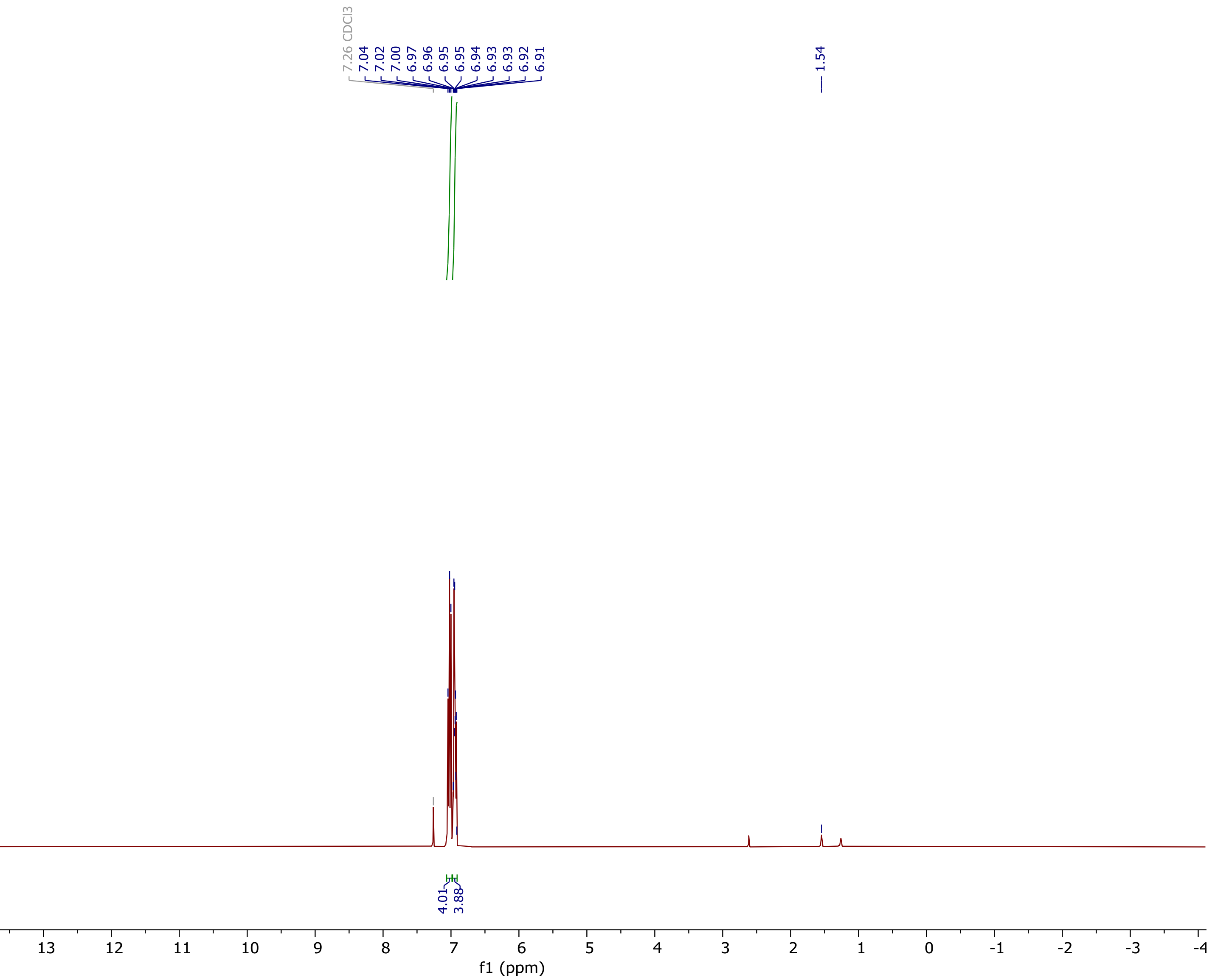

20 – $^{13}C\{^1H\}$ NMR (101 MHz, $CDCl_3$)

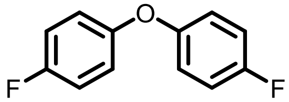

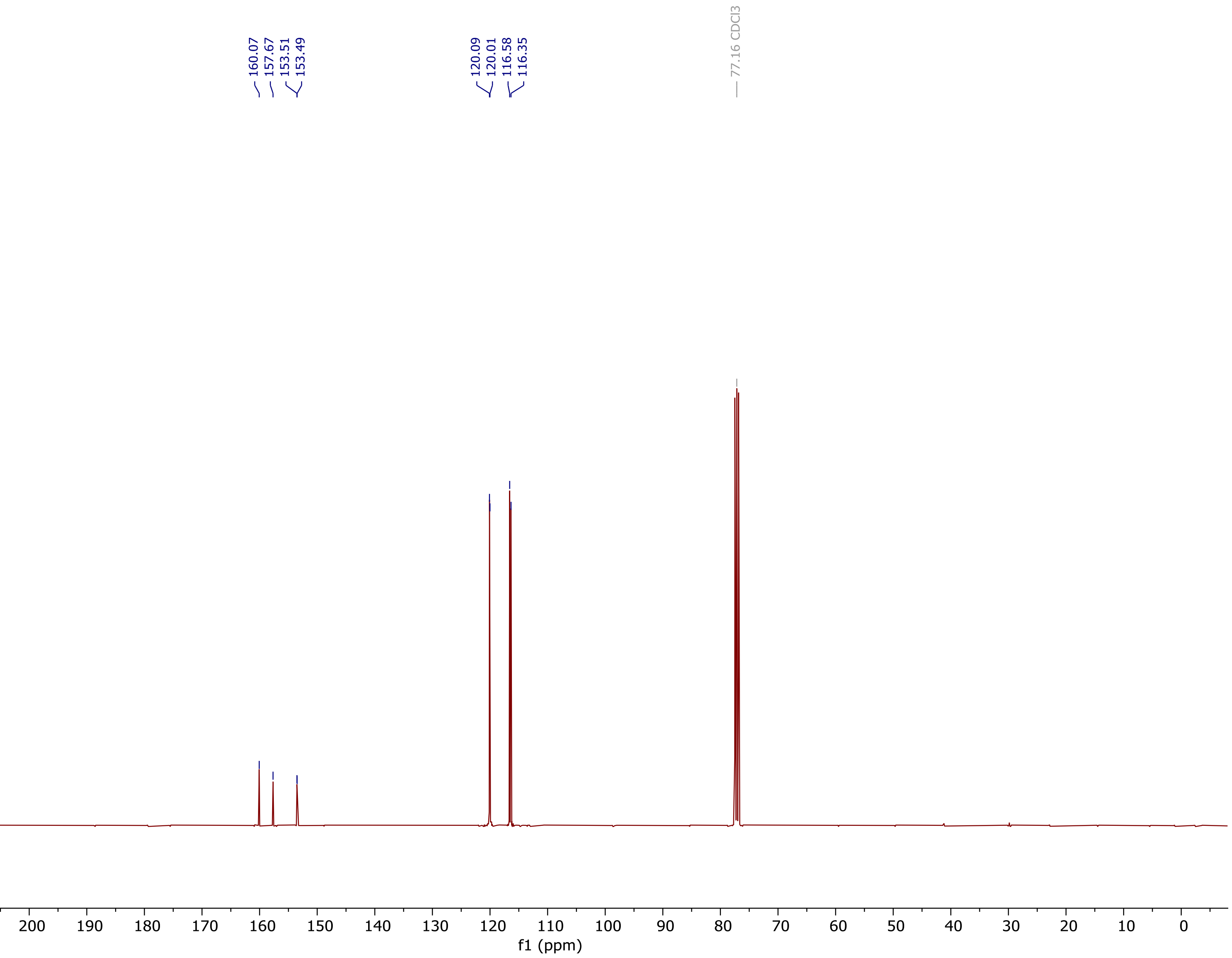

20 – ^{19}F NMR (377 MHz, $CDCl_3$)

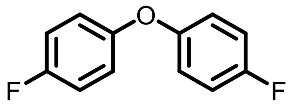

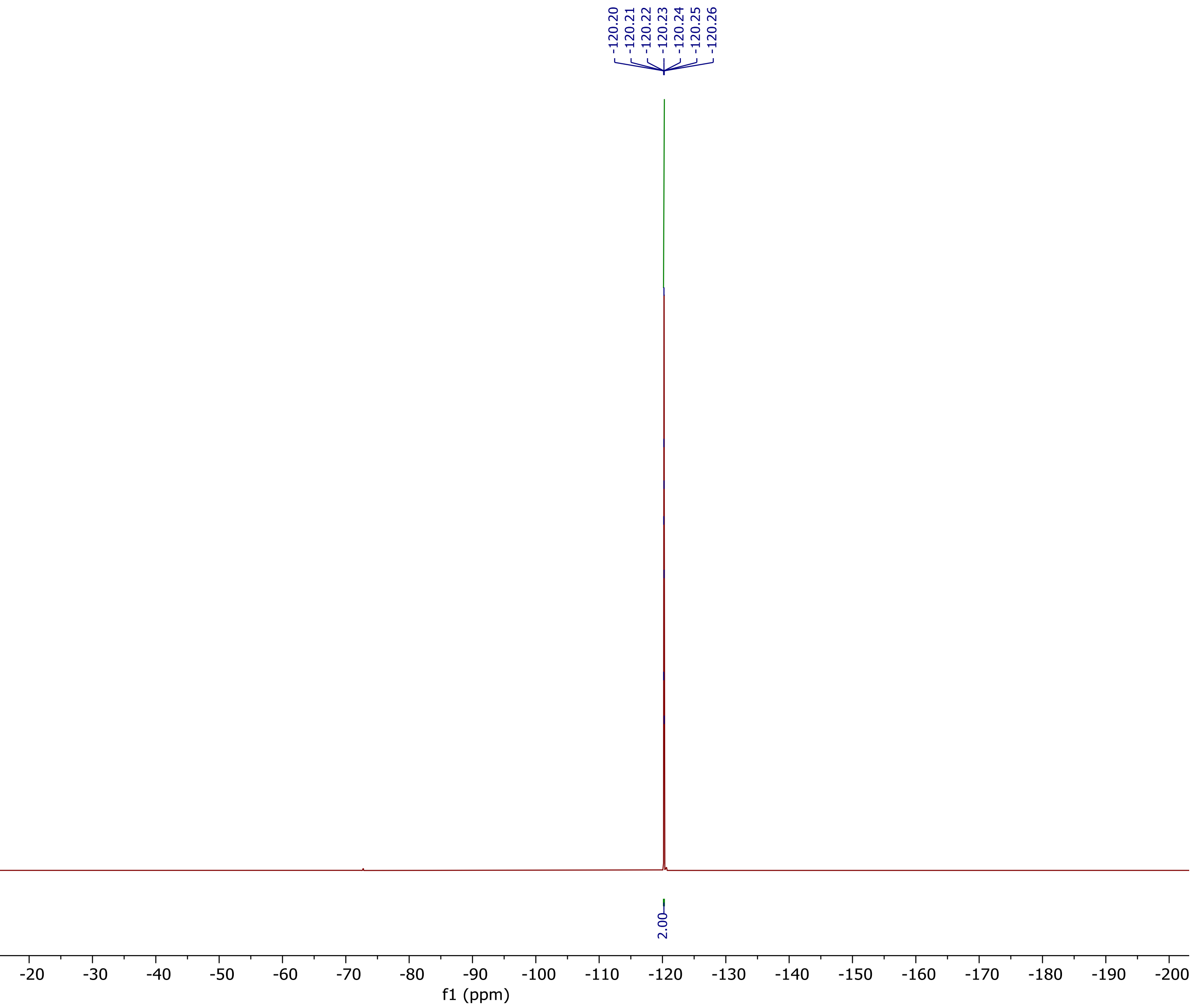

21 - ^{1}H NMR (400 MHz, CDCl$_3$)

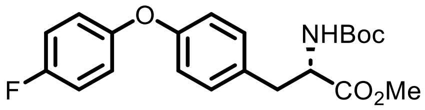

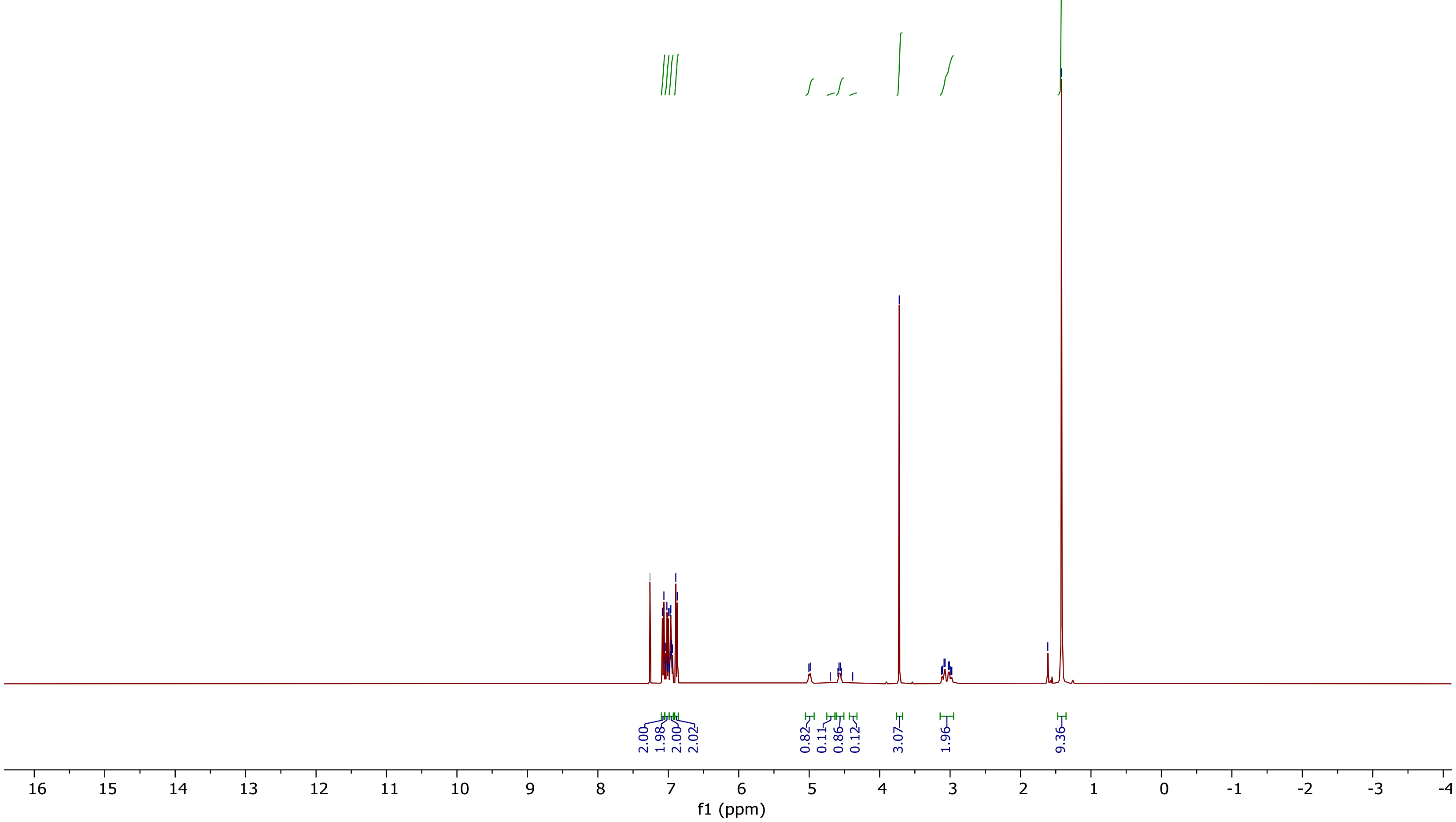

21 - $^{13}C\{^1H\}$ NMR (101 MHz, $CDCl_3$)

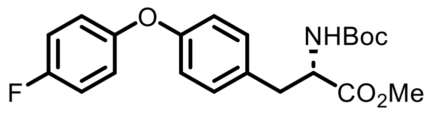

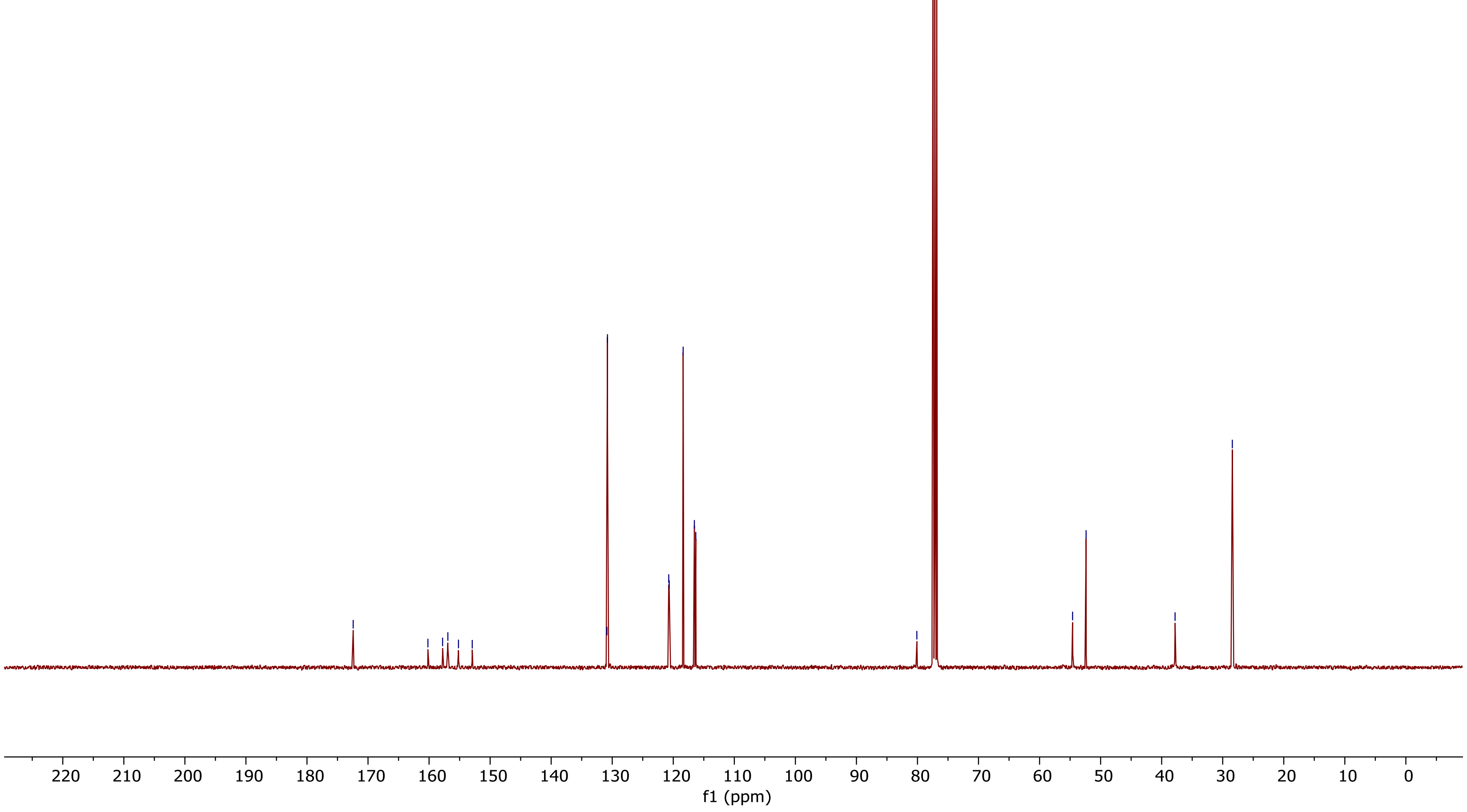

21 - ^{19}F NMR (376 MHz, $CDCl_3$)

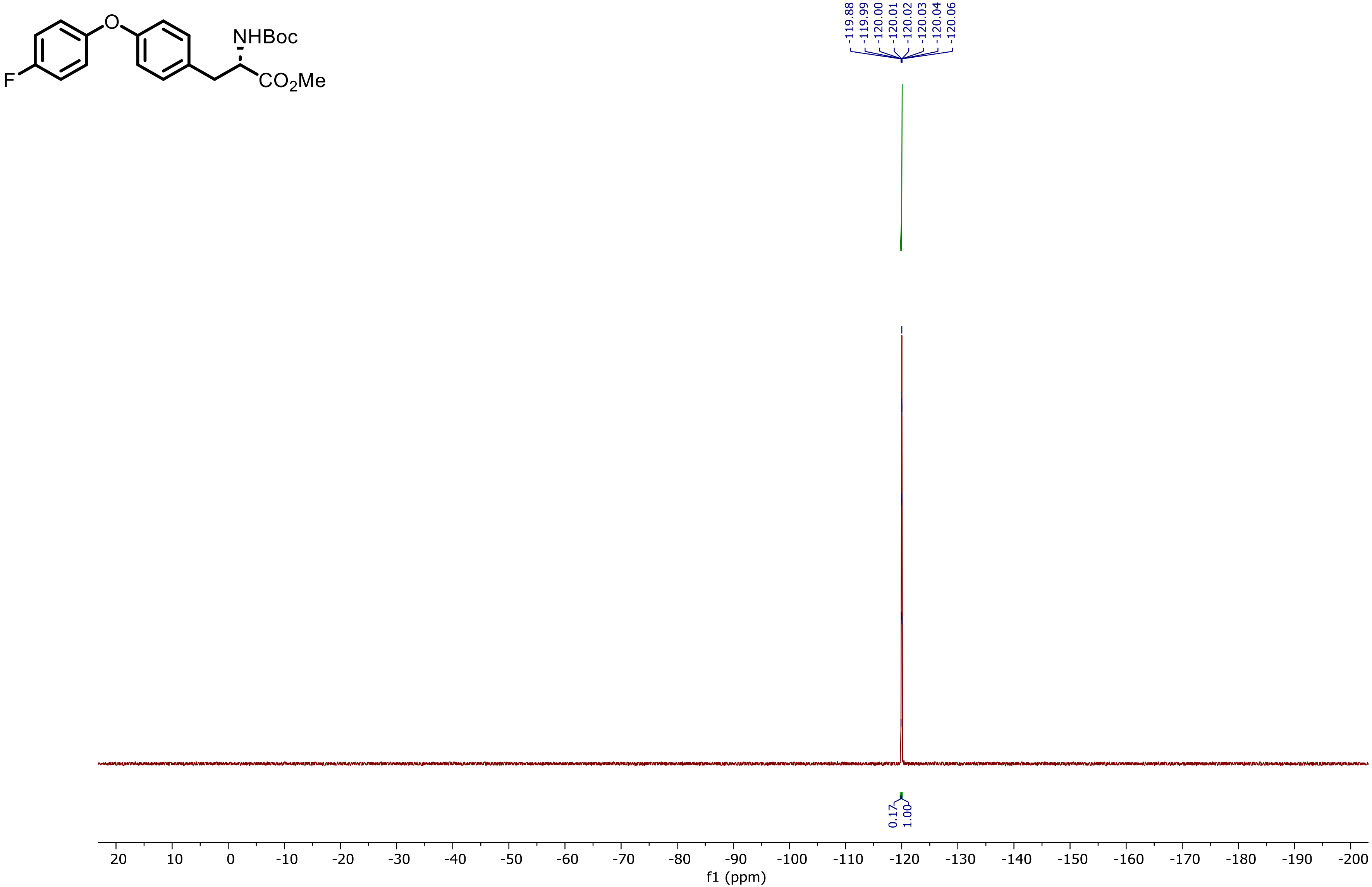

22 - ^{1}H NMR (400 MHz, $CDCl_3$)

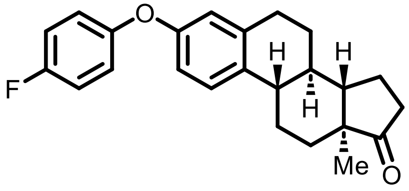

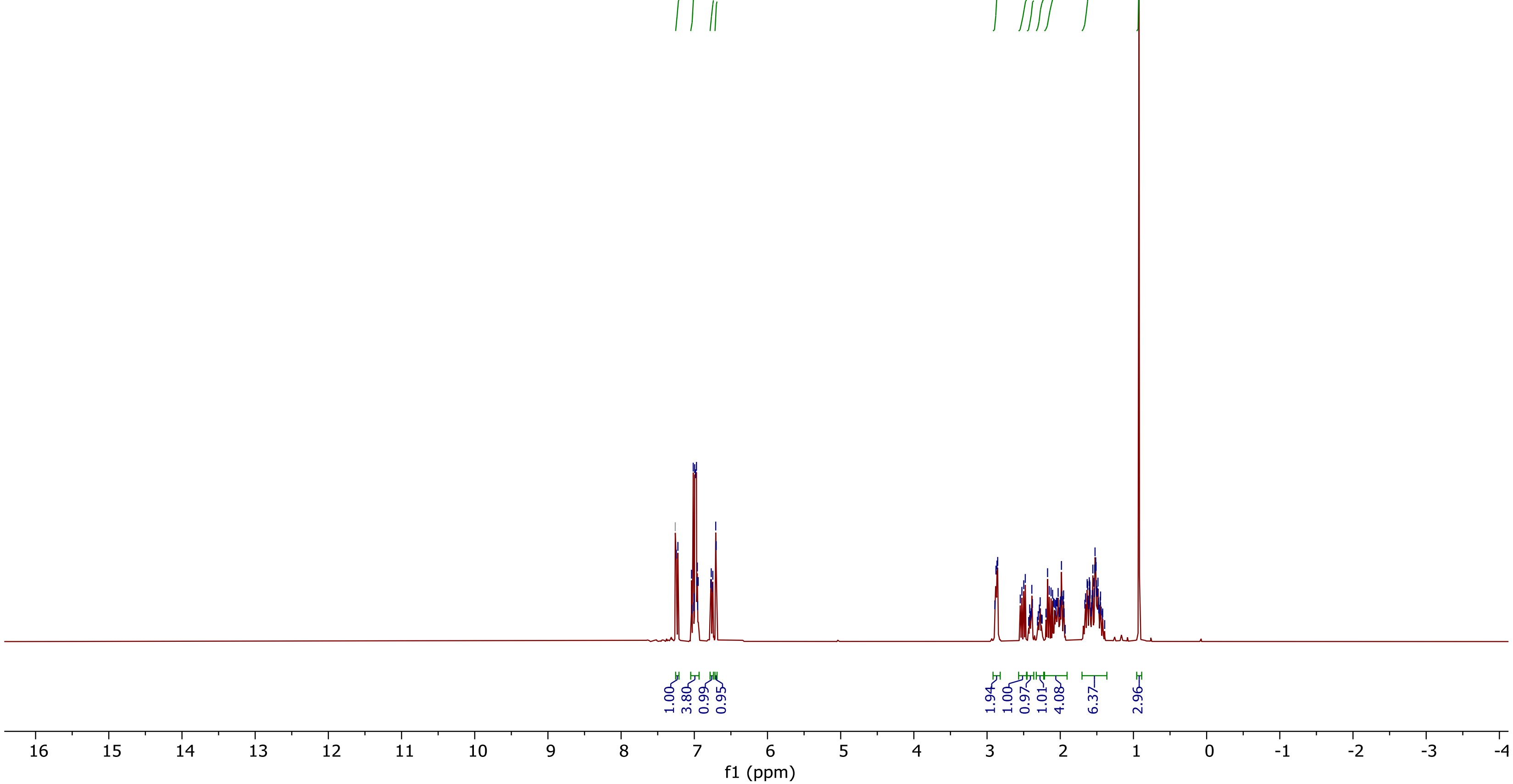

22 - $^{13}C\{^1H\}$ NMR (101 MHz, $CDCl_3$)

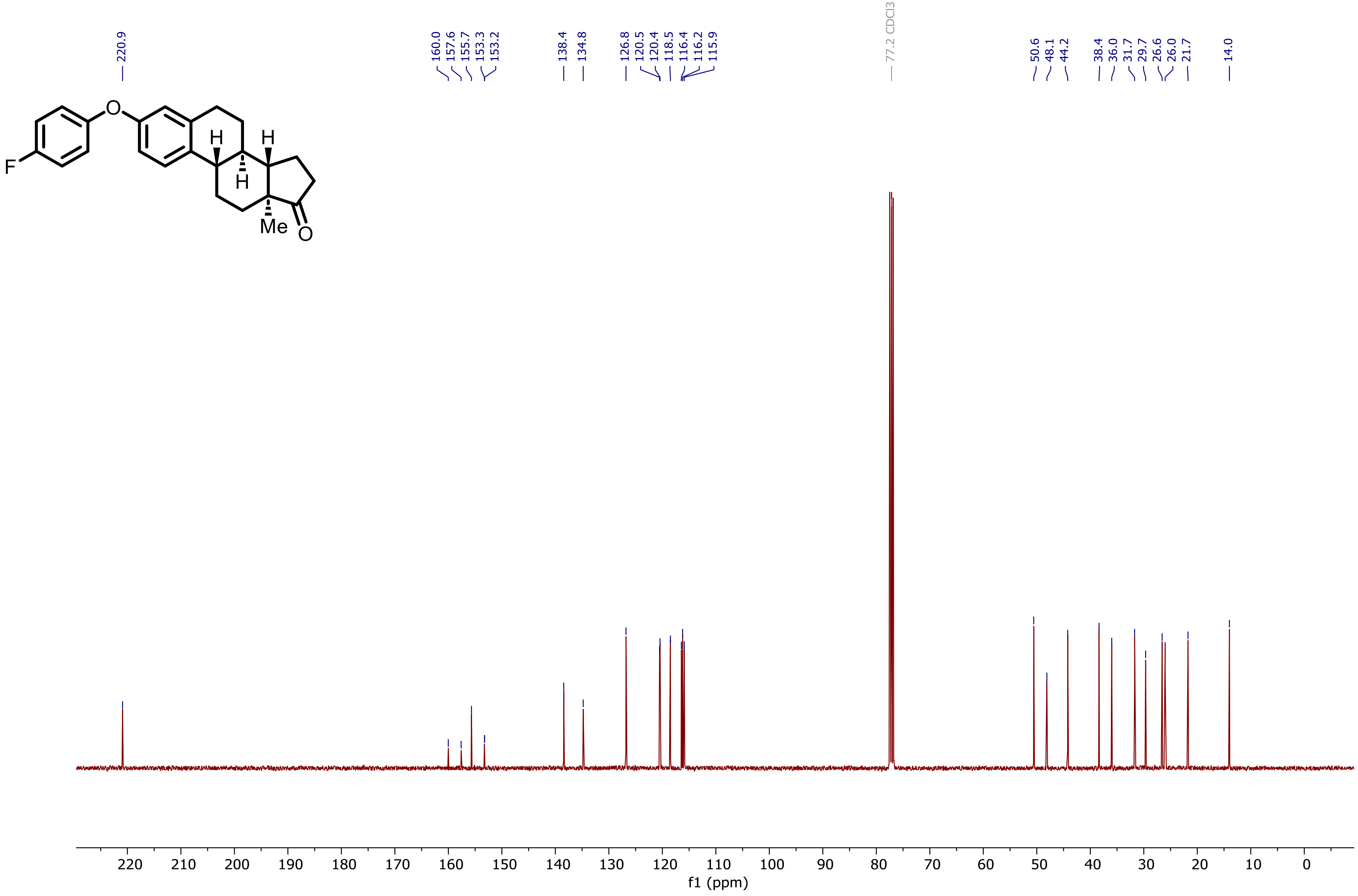

22 - ^{19}F NMR (376 MHz, $CDCl_3$)

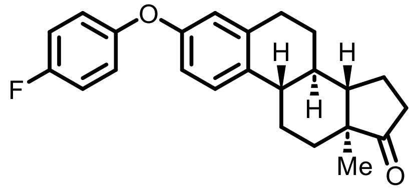

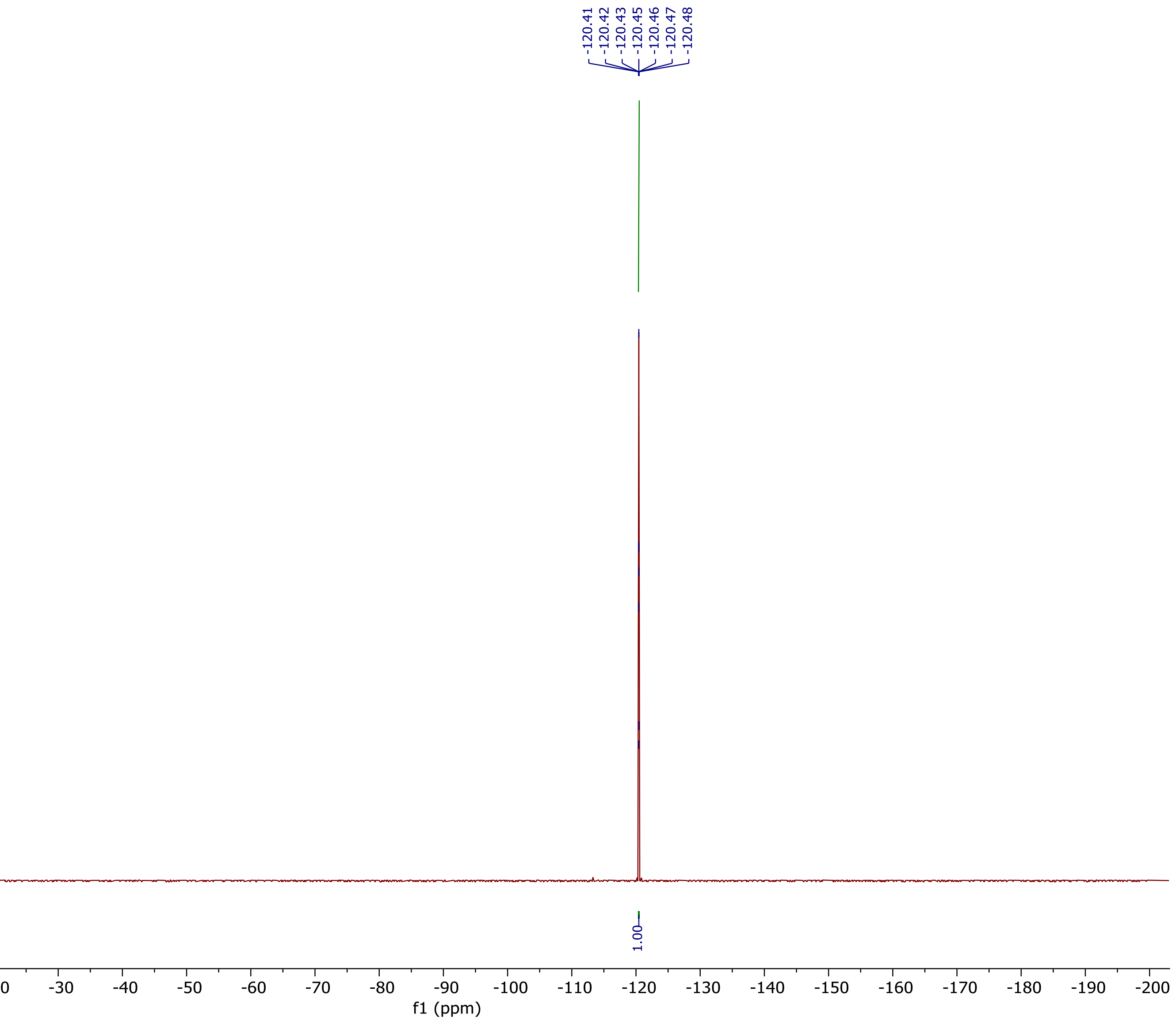

23 – ^{1}H NMR (400 MHz, $CDCl_3$)

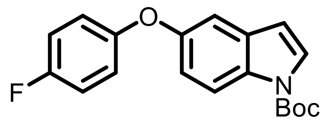

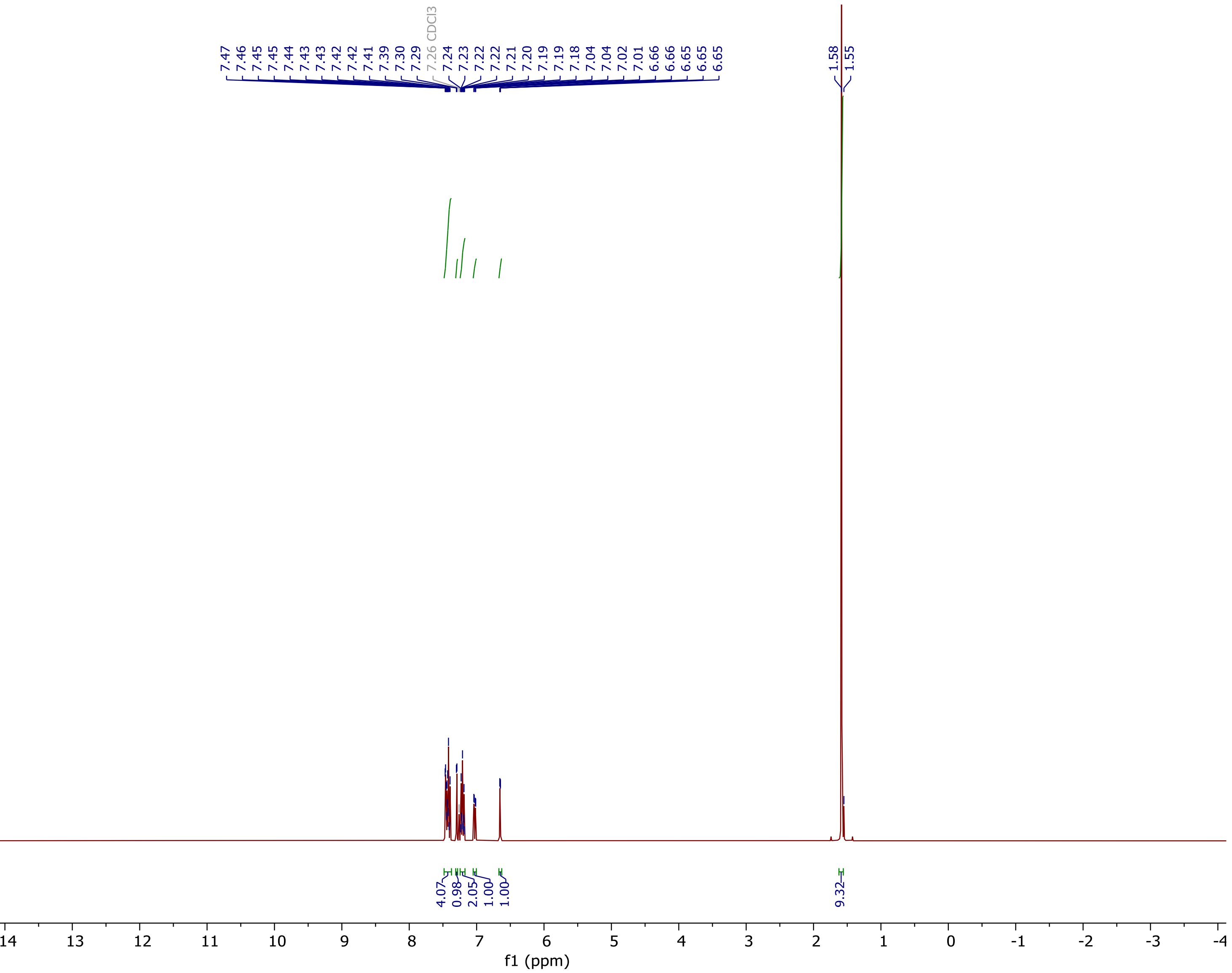

23 – $^{13}C\{^1H\}$ NMR (101 MHz, DMSO-d_6)

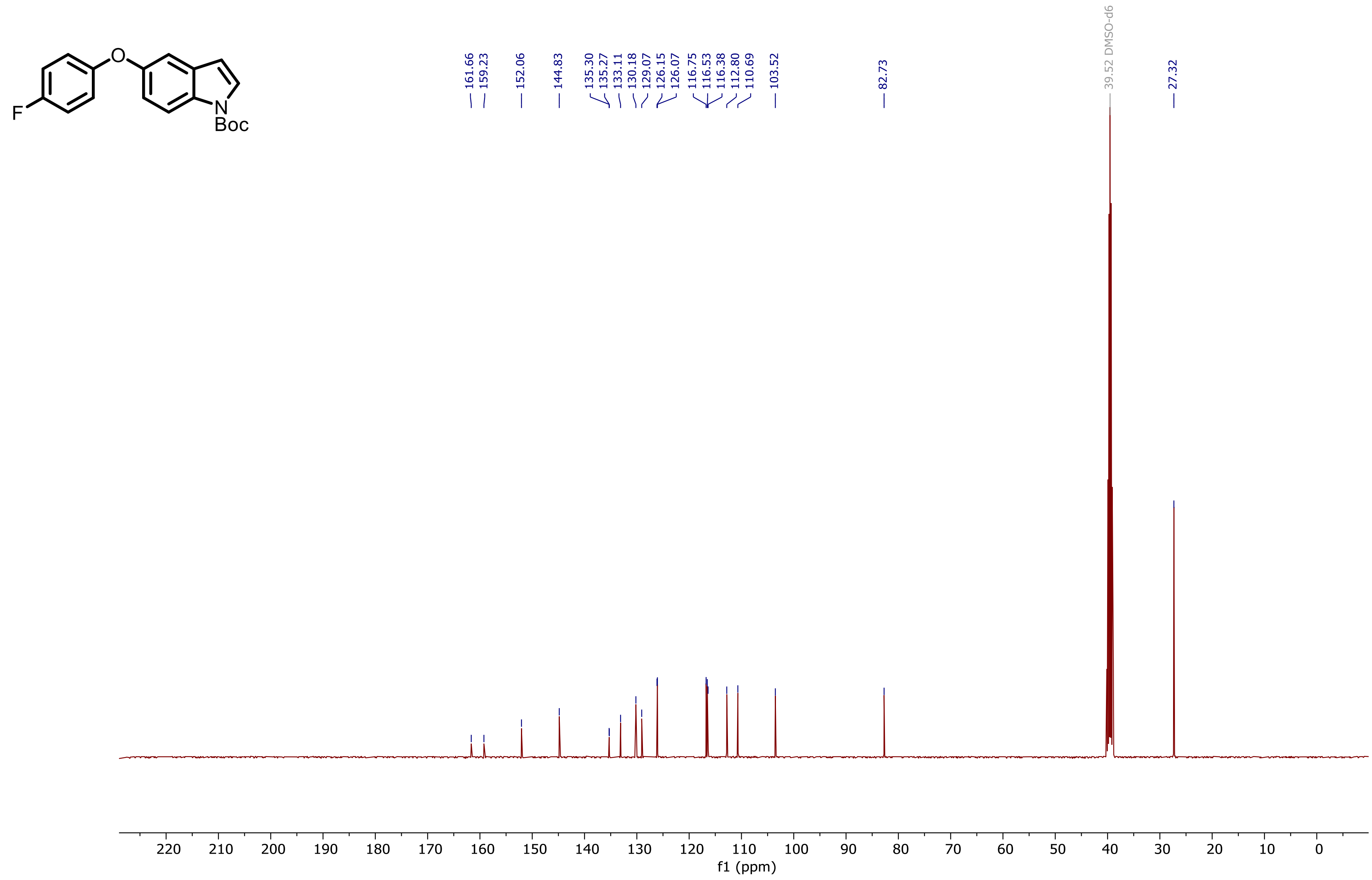

23 – ^{19}F NMR (376 MHz, $CDCl_3$)

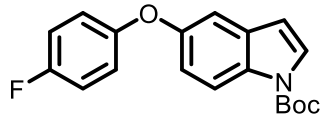

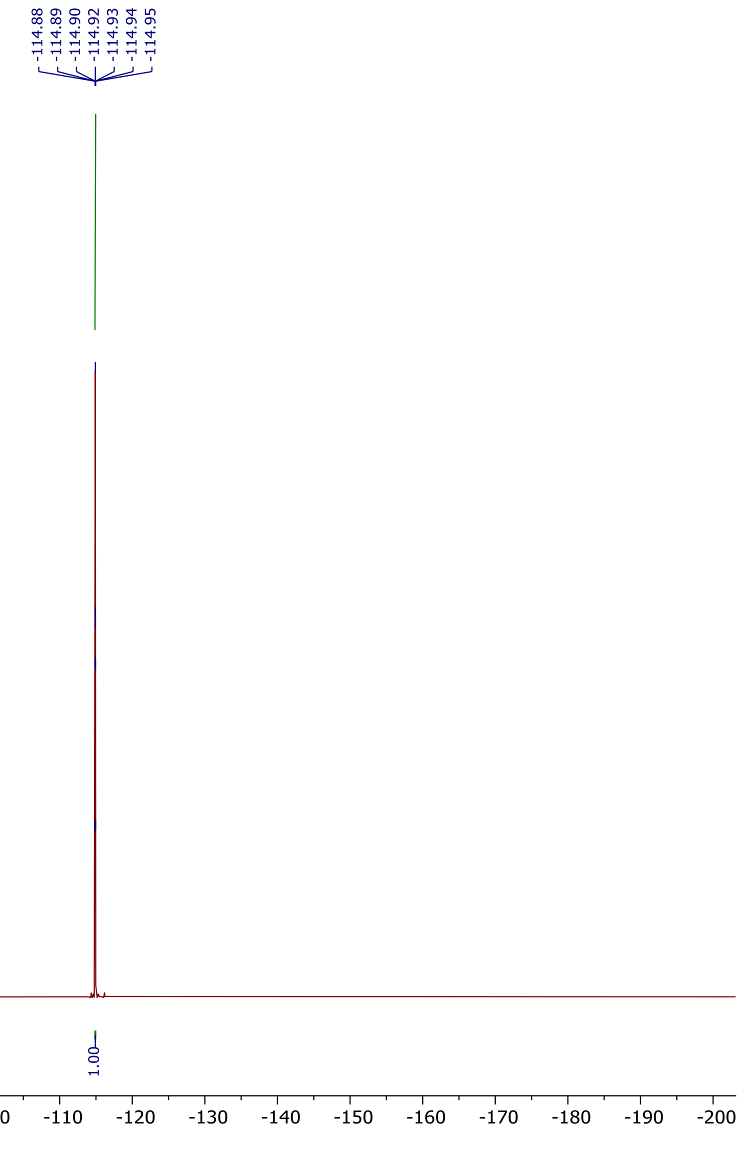

24 – ^{1}H NMR (400 MHz, $CDCl_3$) – *note: contains ca 1% N-Me carbazole, from Pd precatalyst*

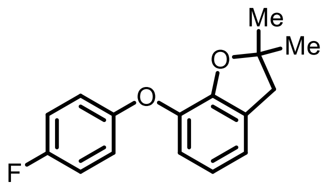

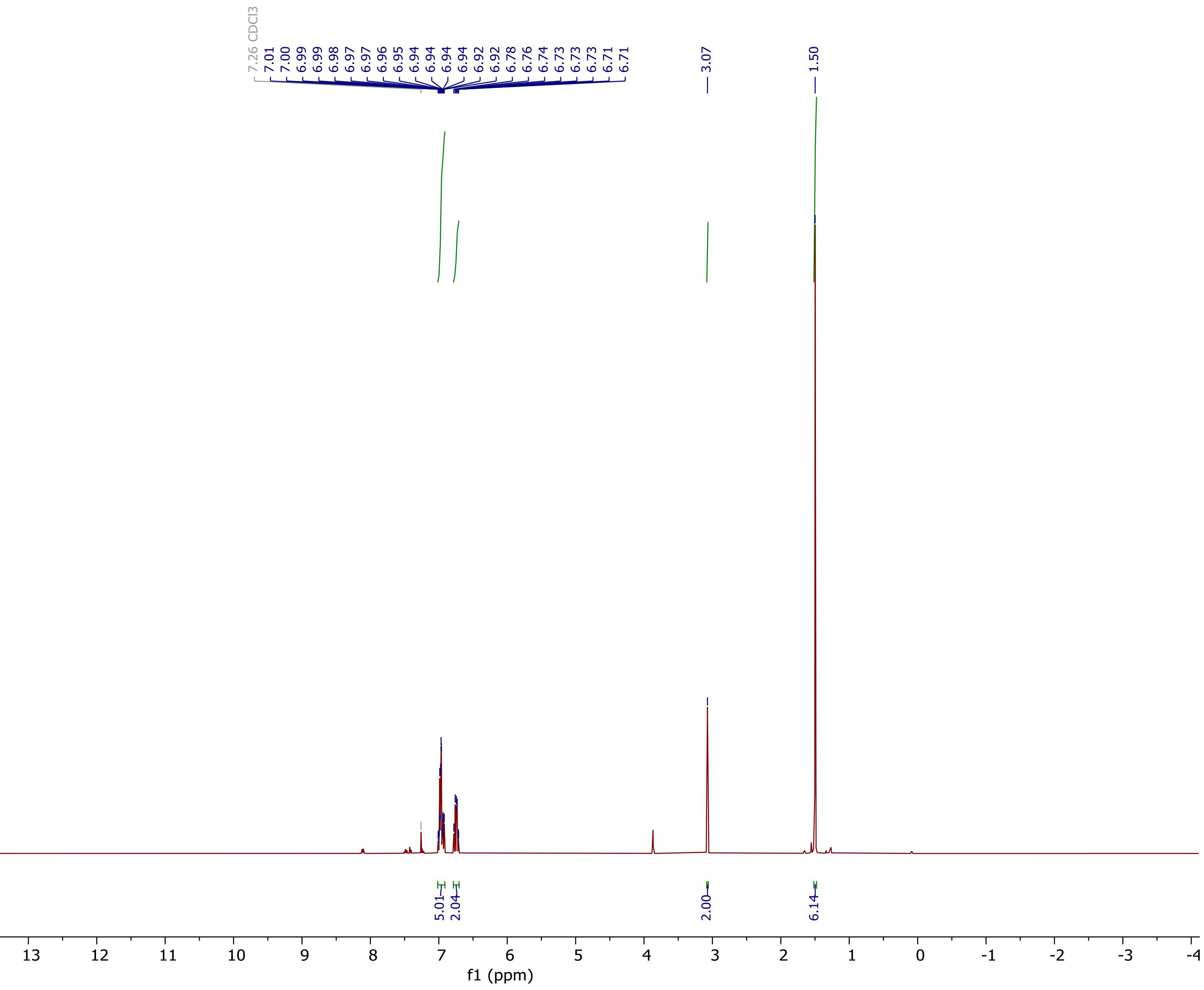

24 – ^{13}C{^{1}H} NMR (101 MHz, CDCl$_3$) – *note: contains ca 1% N-Me carbazole, from Pd precatalyst*

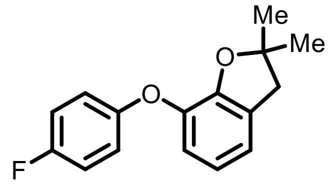

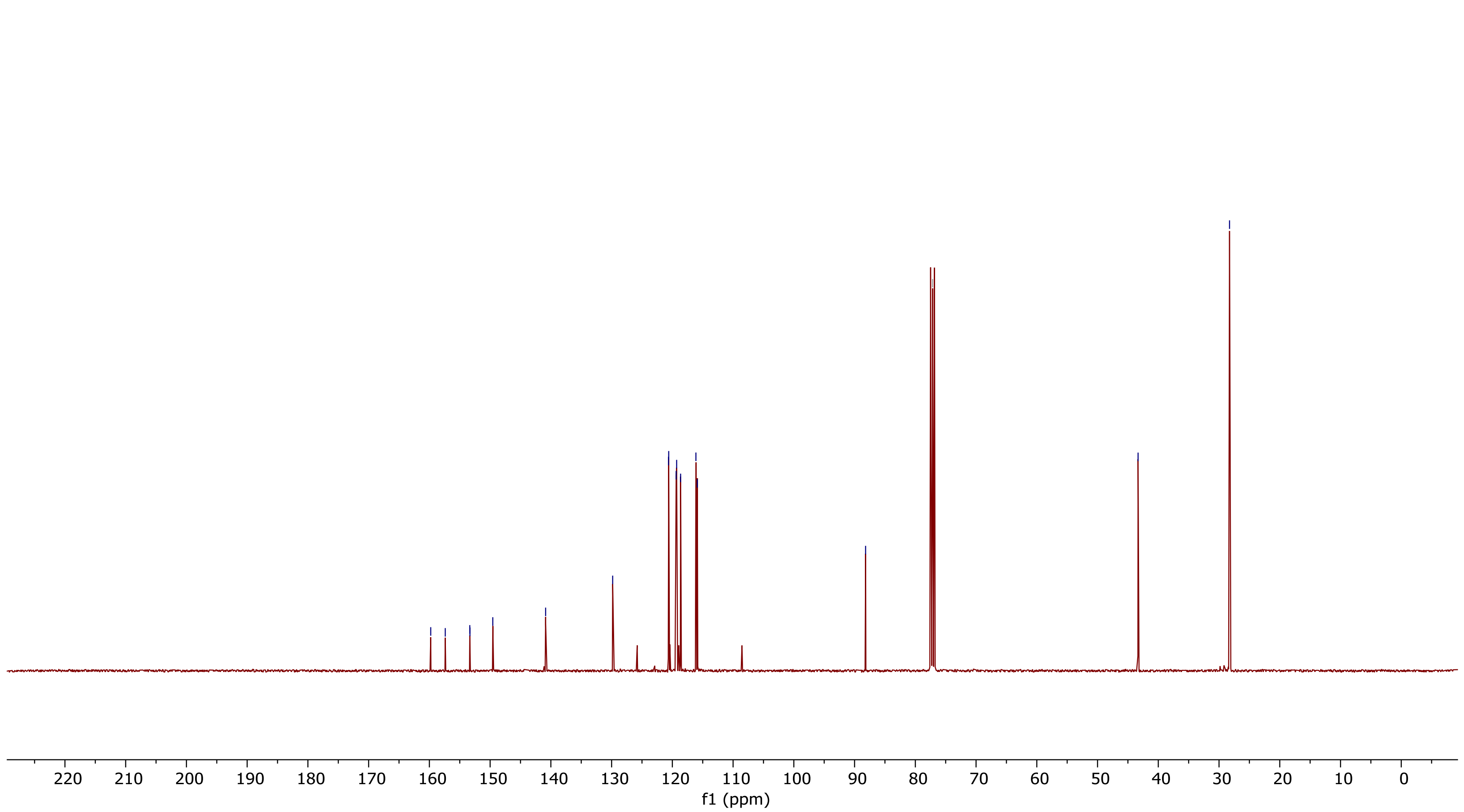

24 – ^{19}F NMR (376 MHz, $CDCl_3$)

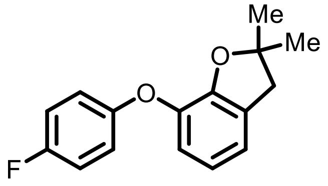

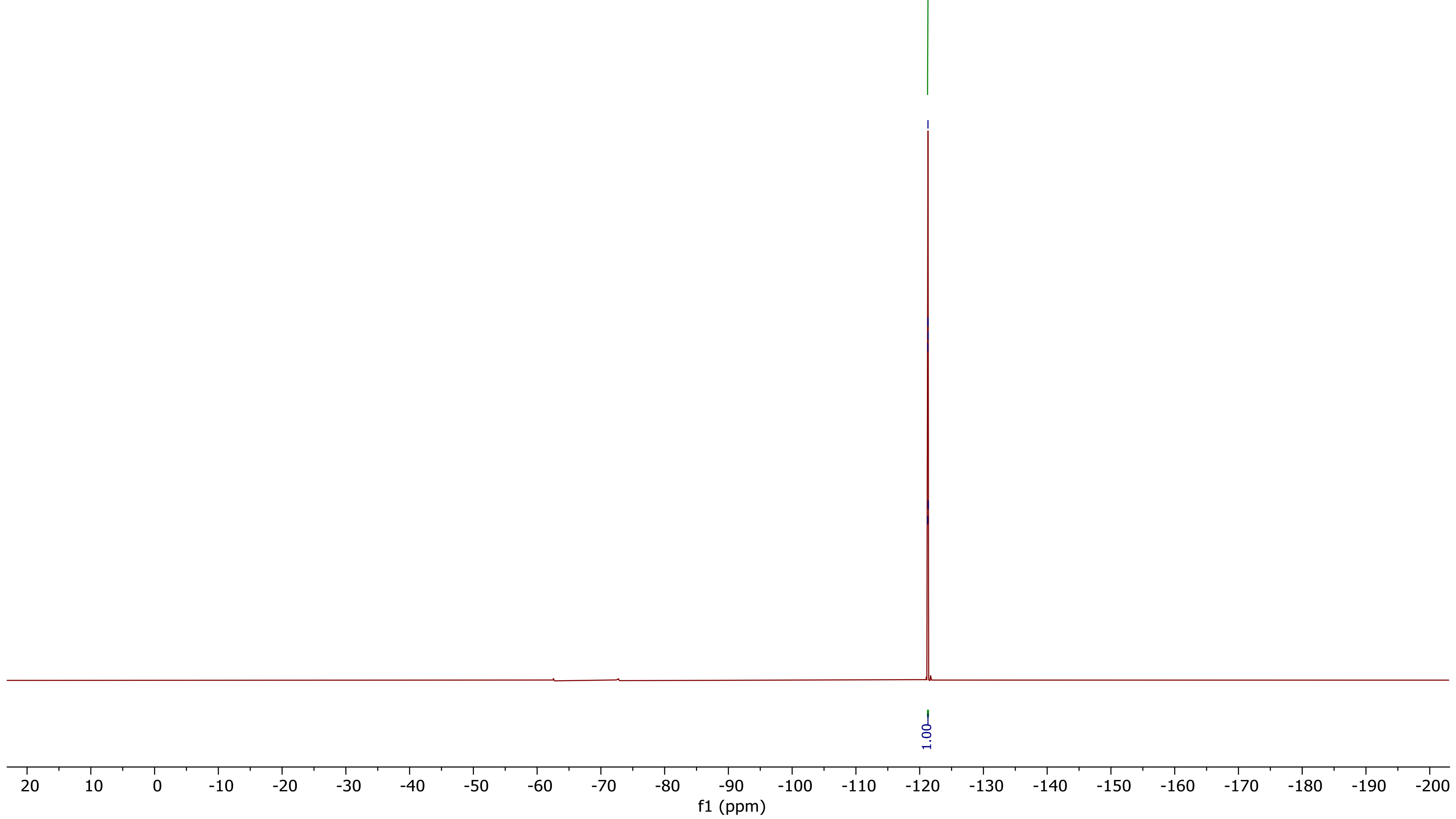

25 – ^{1}H NMR (400 MHz, $CDCl_3$)

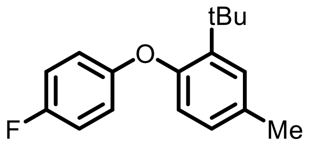

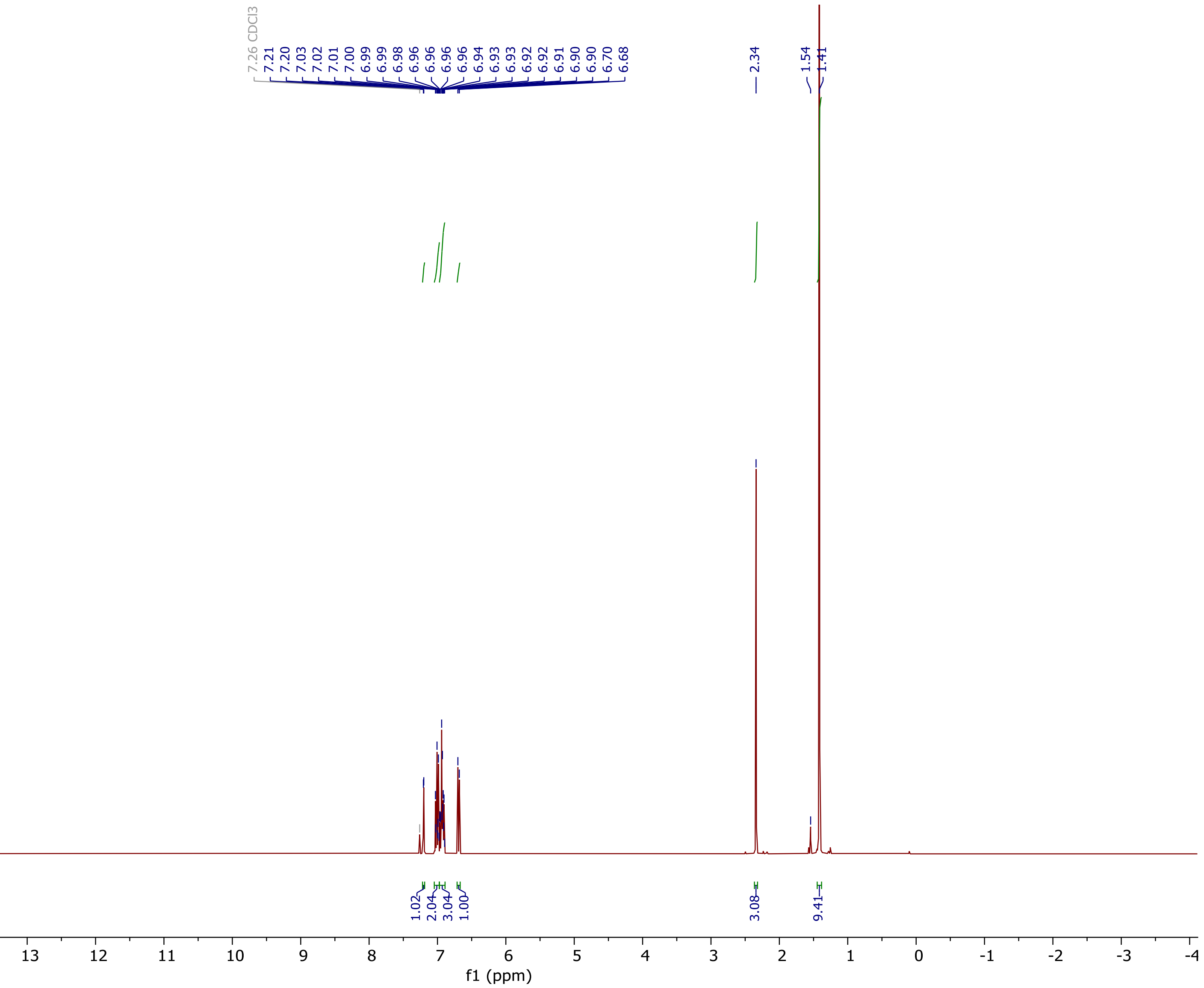

25 – $^{13}C\{^1H\}$ NMR (101 MHz, $CDCl_3$)

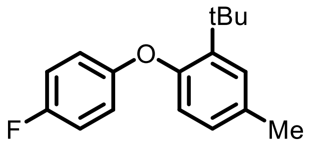

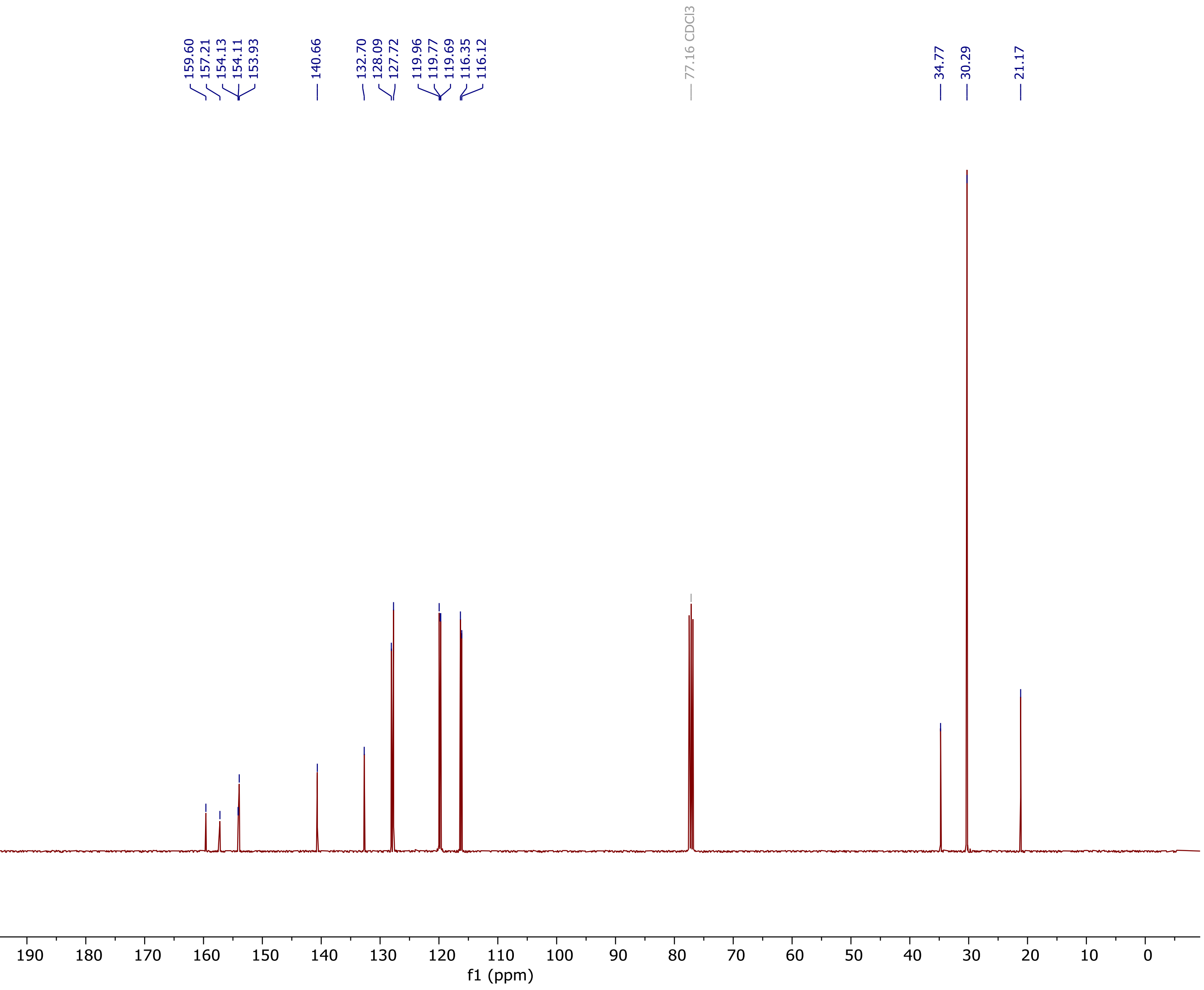

25 – ^{19}F NMR (376 MHz, $CDCl_3$)

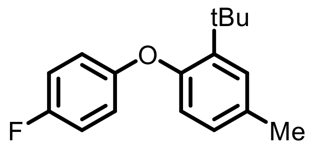

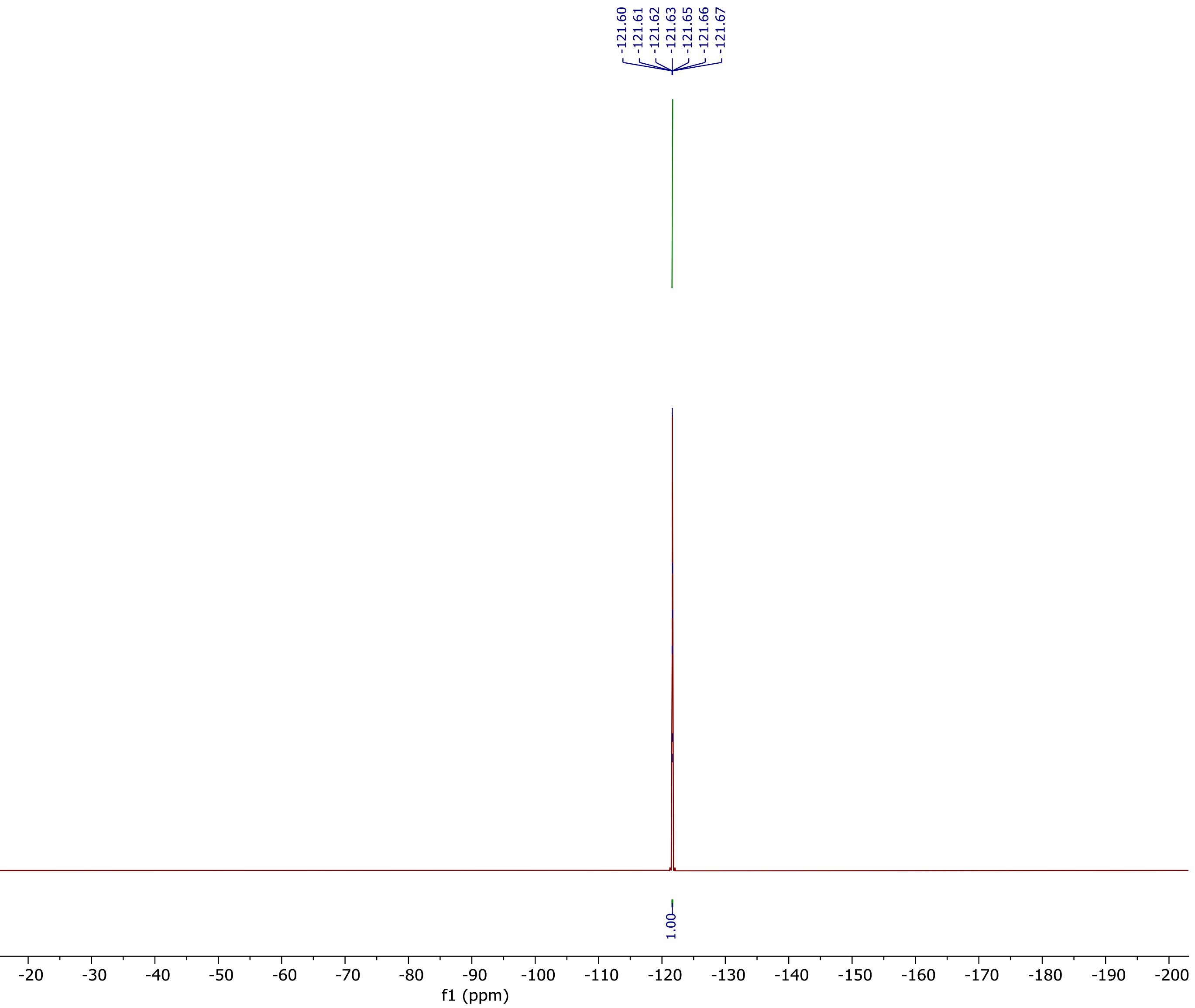

26 – ^{1}H NMR (500 MHz, CDCl$_3$)

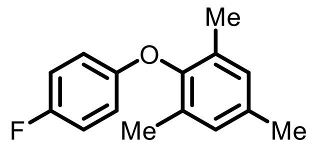

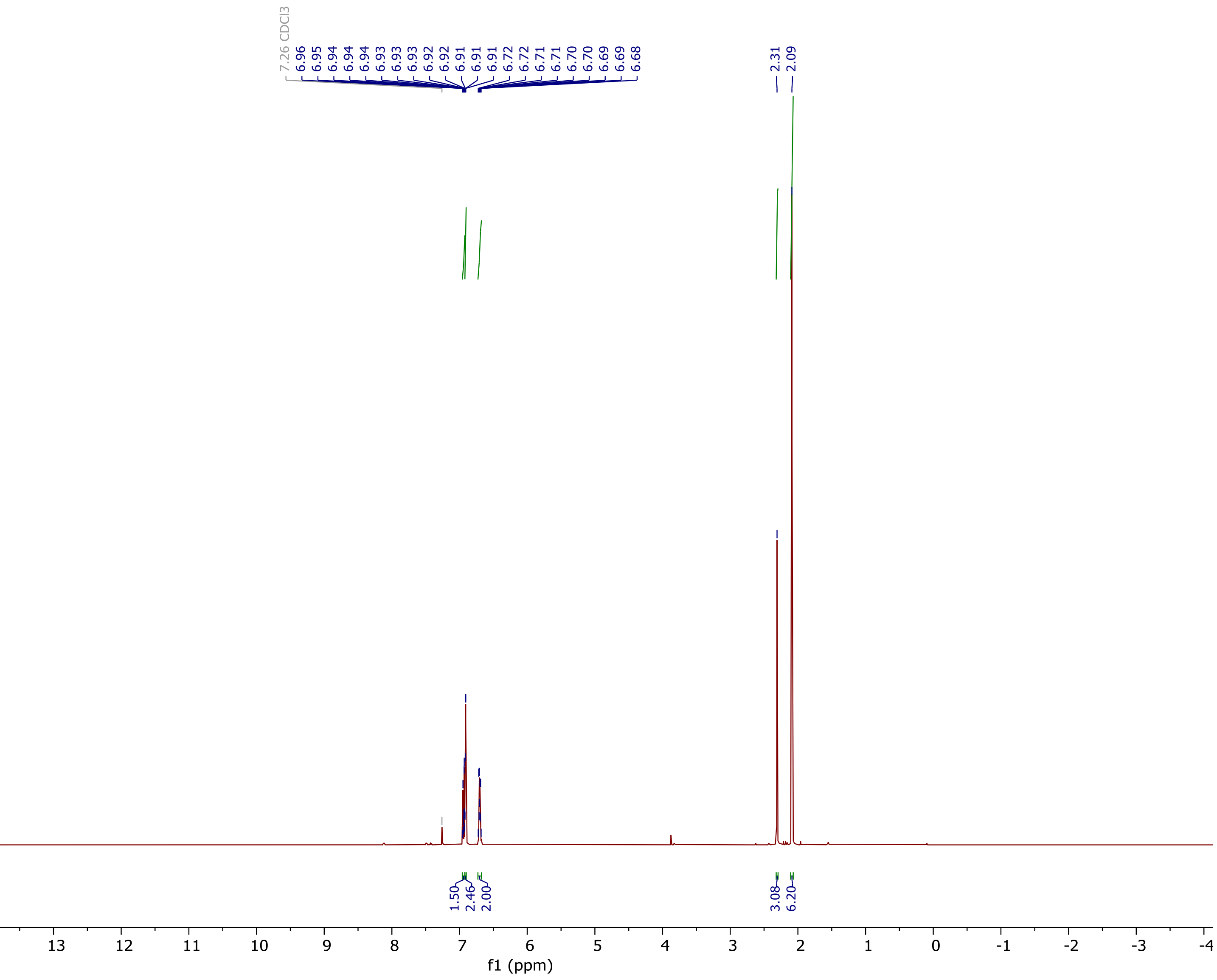

26 – $^{13}C\{^{1}H\}$ NMR (126 MHz, $CDCl_3$)

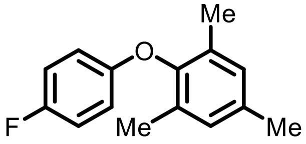

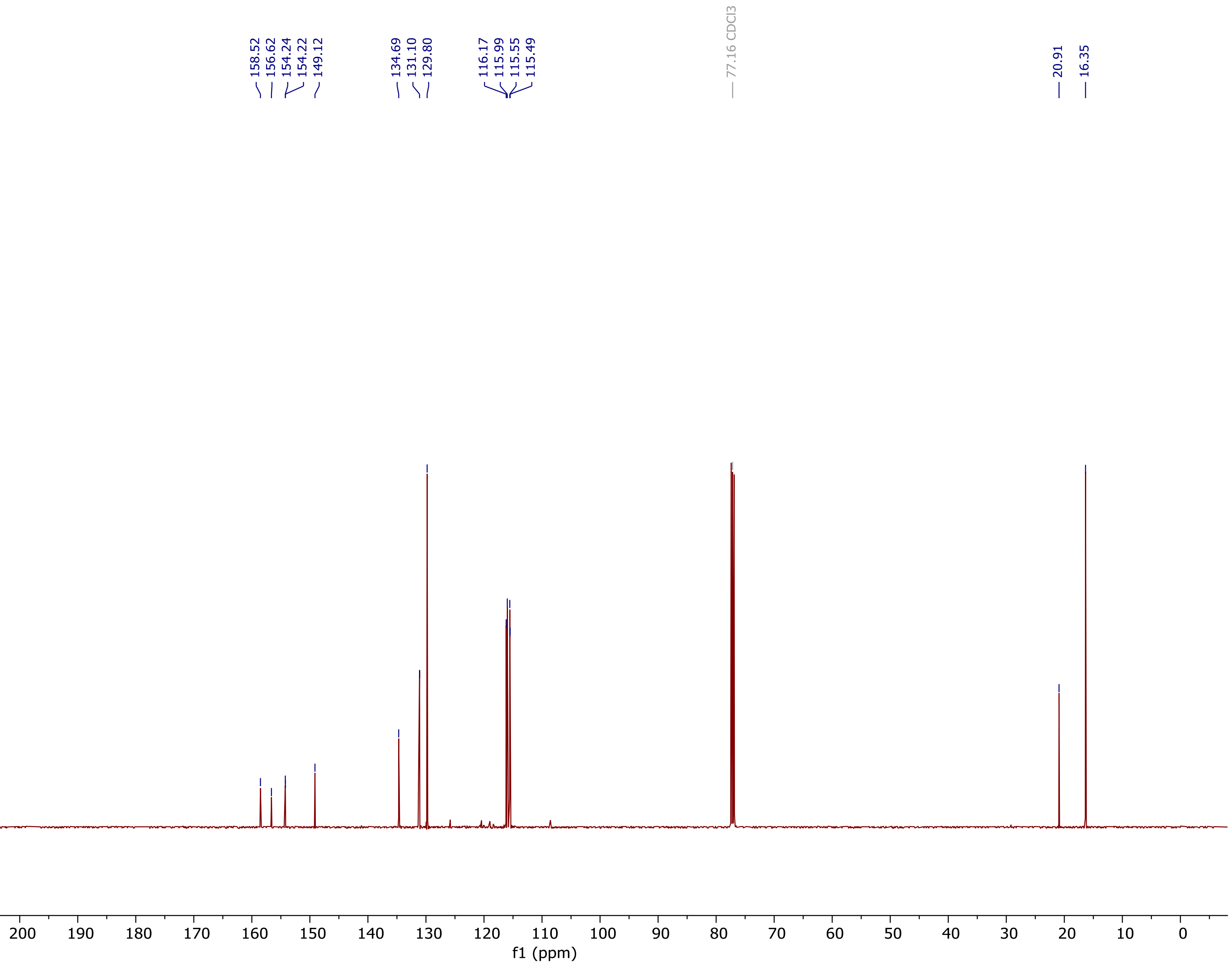

26 – ^{19}F NMR (376 MHz, $CDCl_3$)

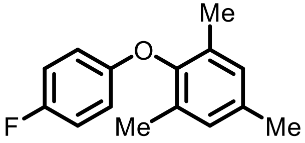

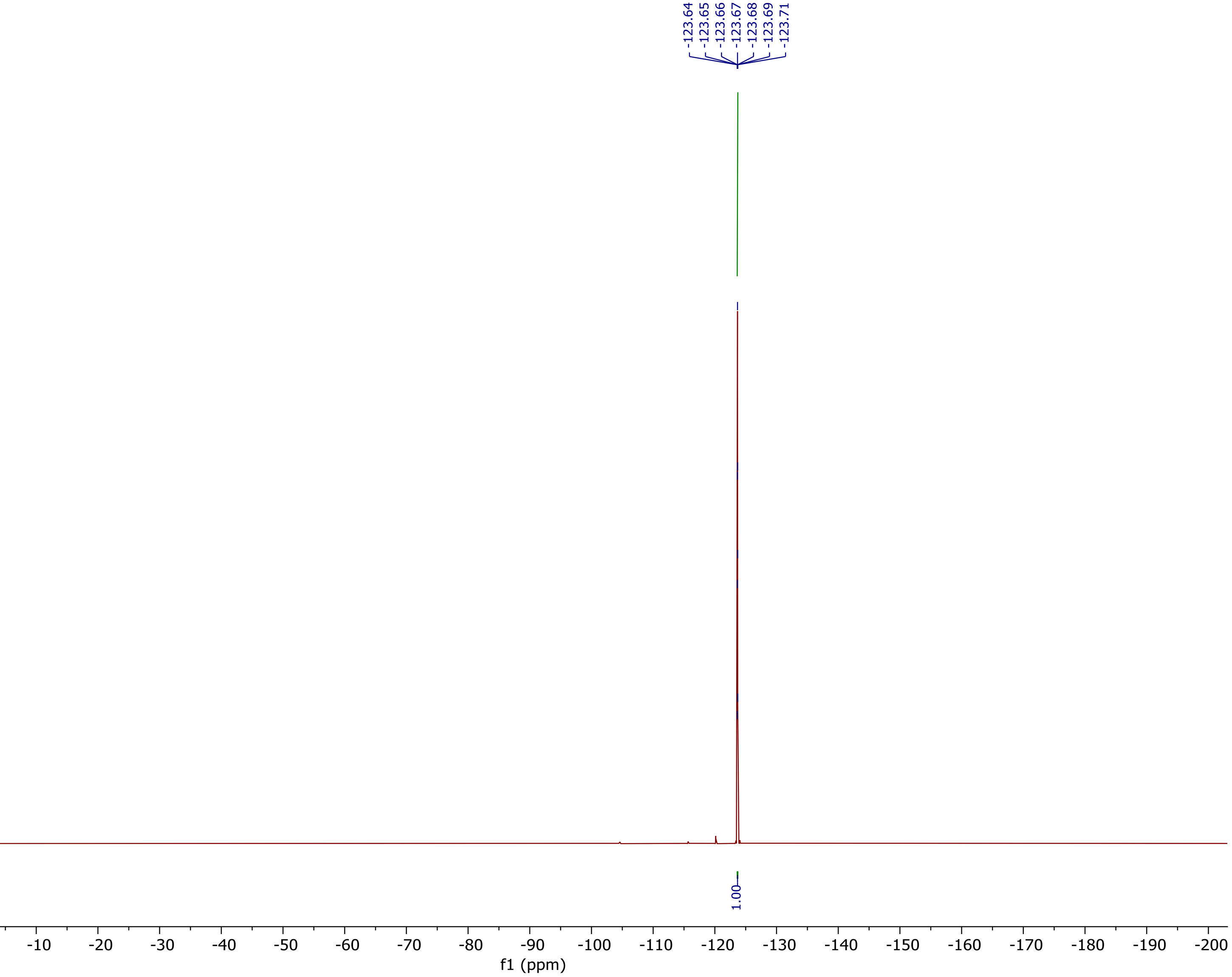

27 - ^{1}H NMR (400 MHz, $CDCl_3$)

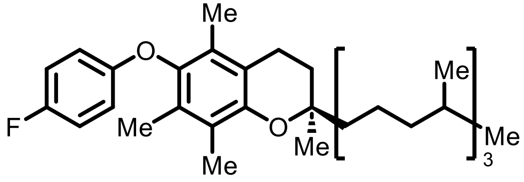

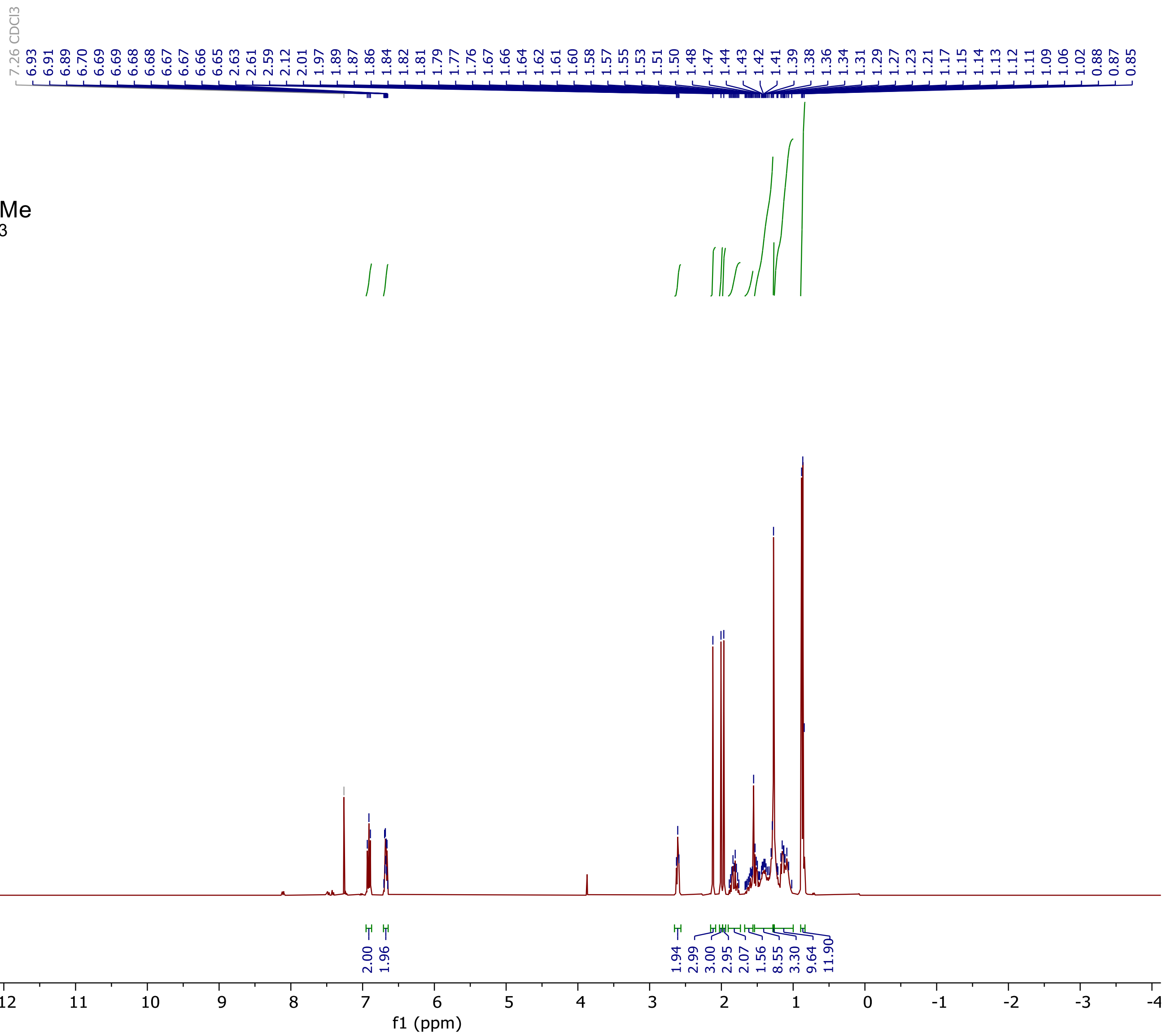

27 - $^{13}C\{^{1}H\}$ NMR (101 MHz, $CDCl_3$)

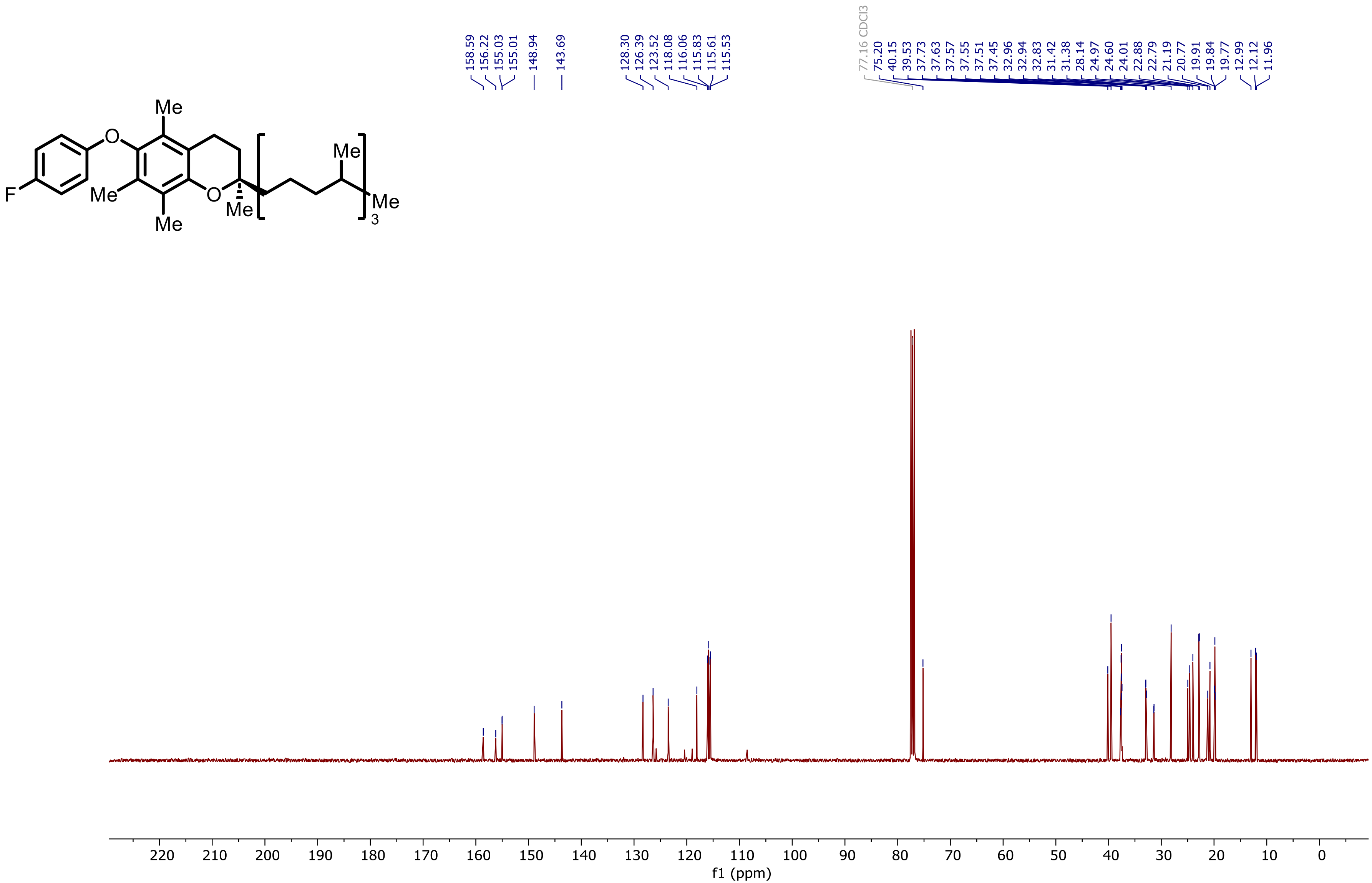

27 - ^{19}F NMR (376 MHz, CDCl$_3$)

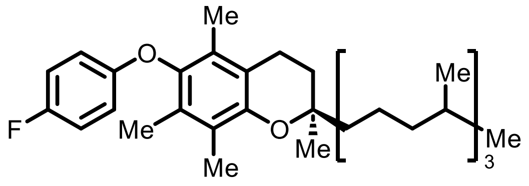

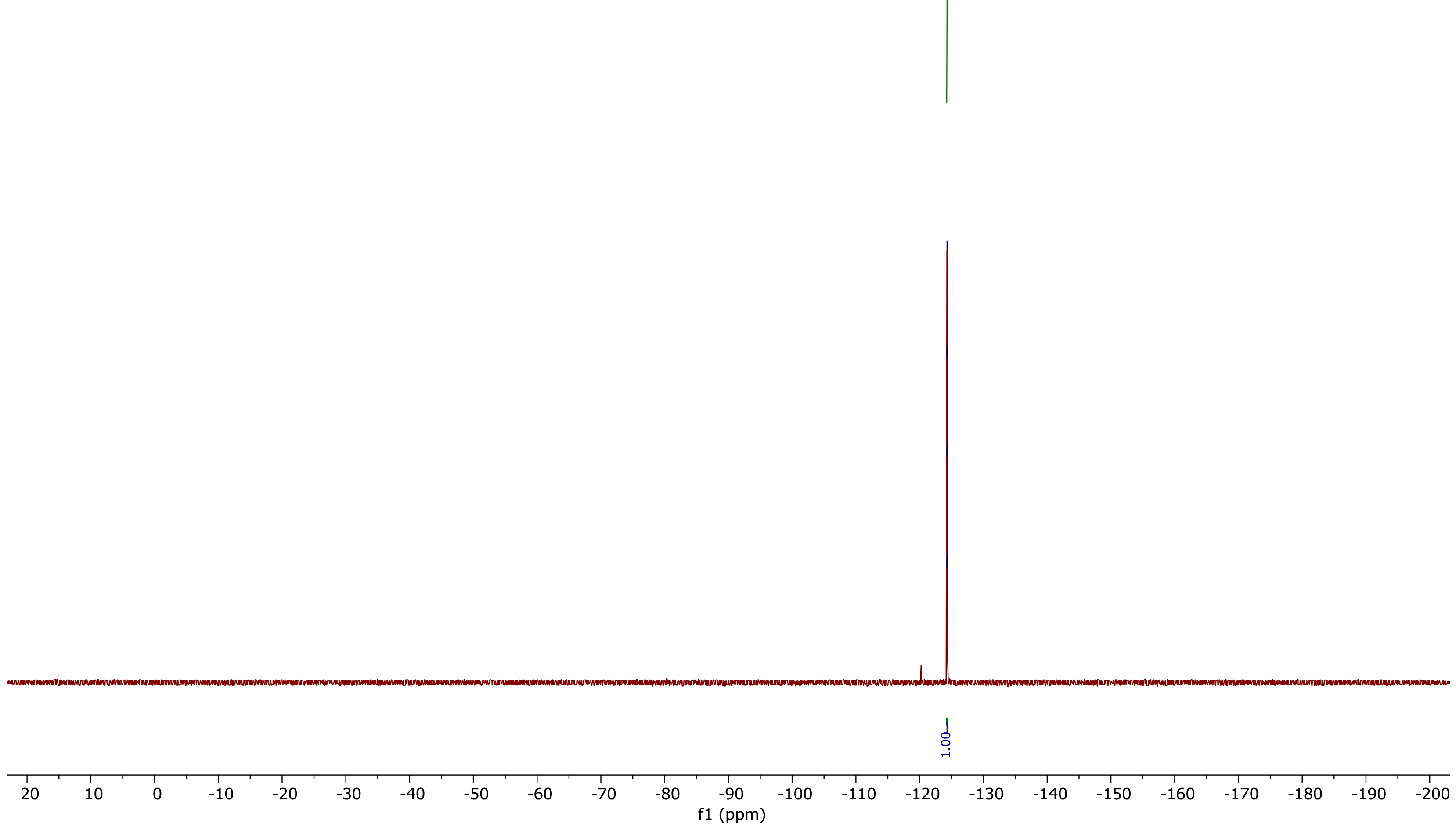

28 – ^{1}H NMR (400 MHz, $CDCl_3$)

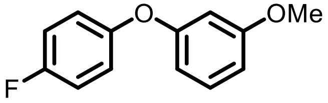

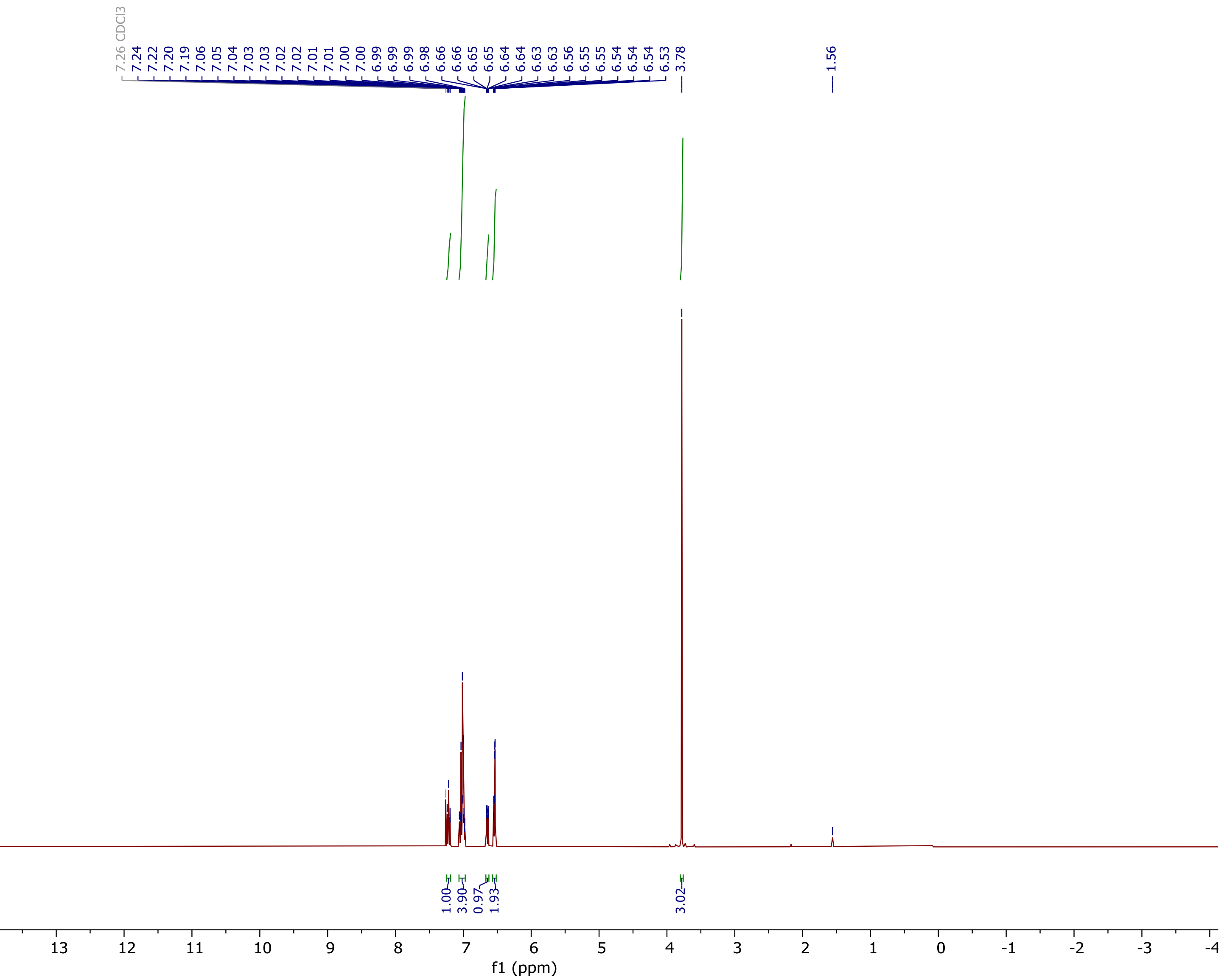

28 – $^{13}C\{^1H\}$ NMR (101 MHz, $CDCl_3$)

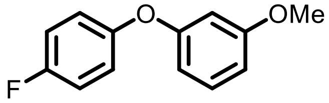

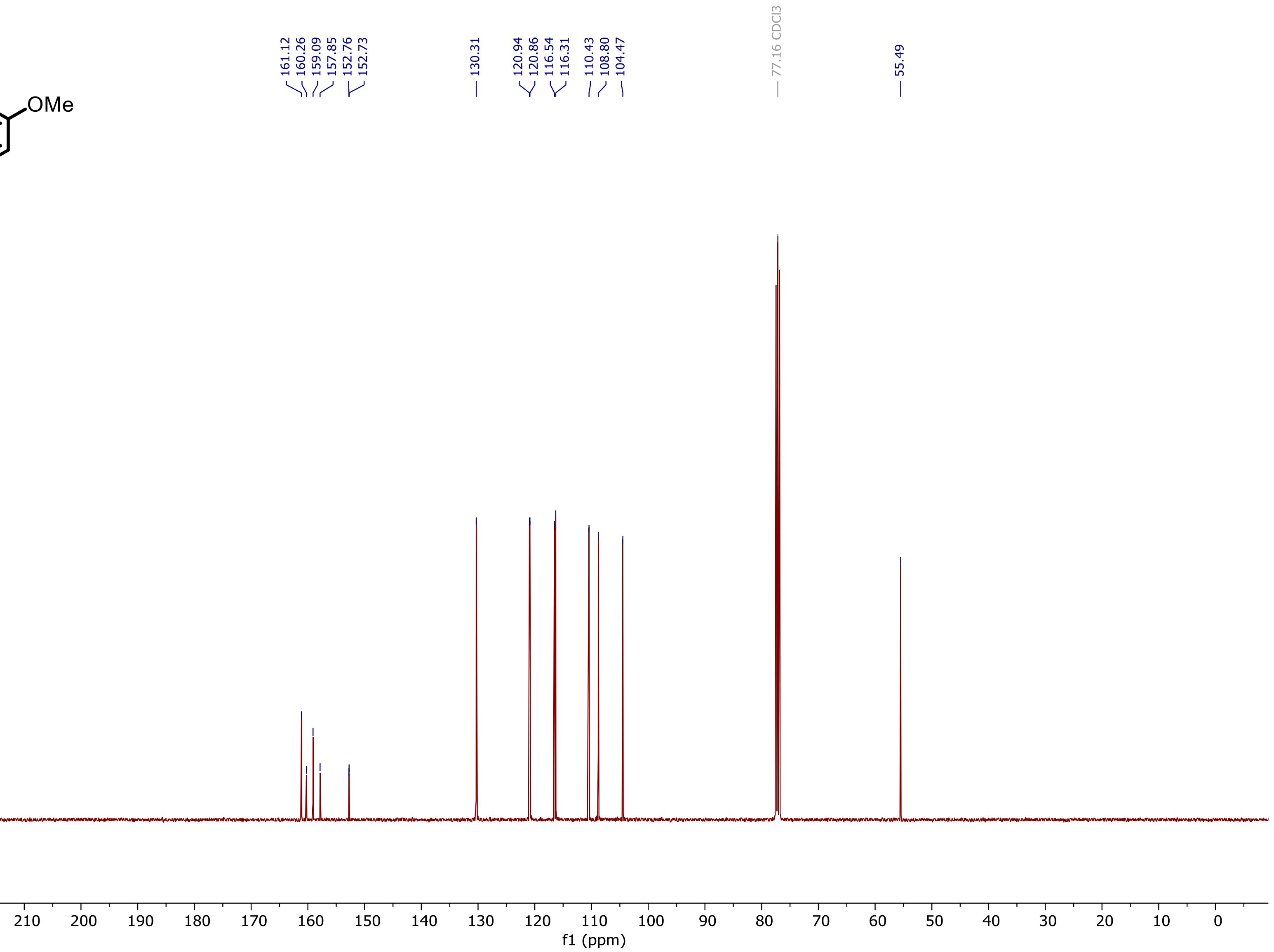

28 – ^{19}F NMR (376 MHz, $CDCl_3$)

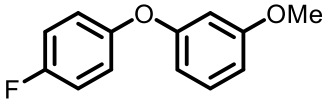

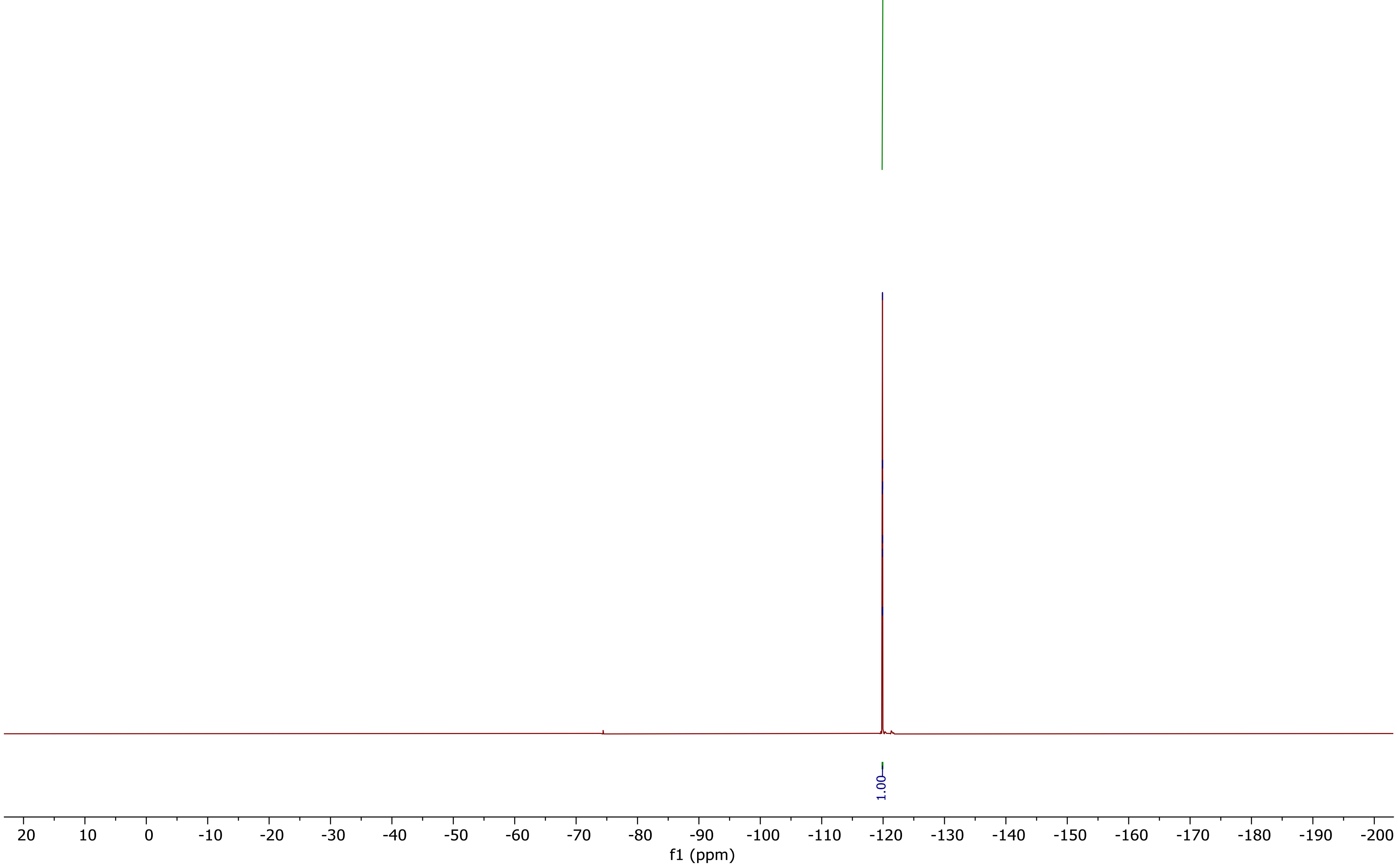

29 – ^{1}H NMR (500 MHz, $CDCl_3$)

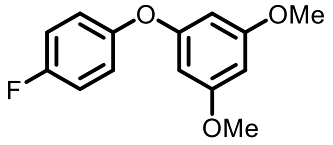

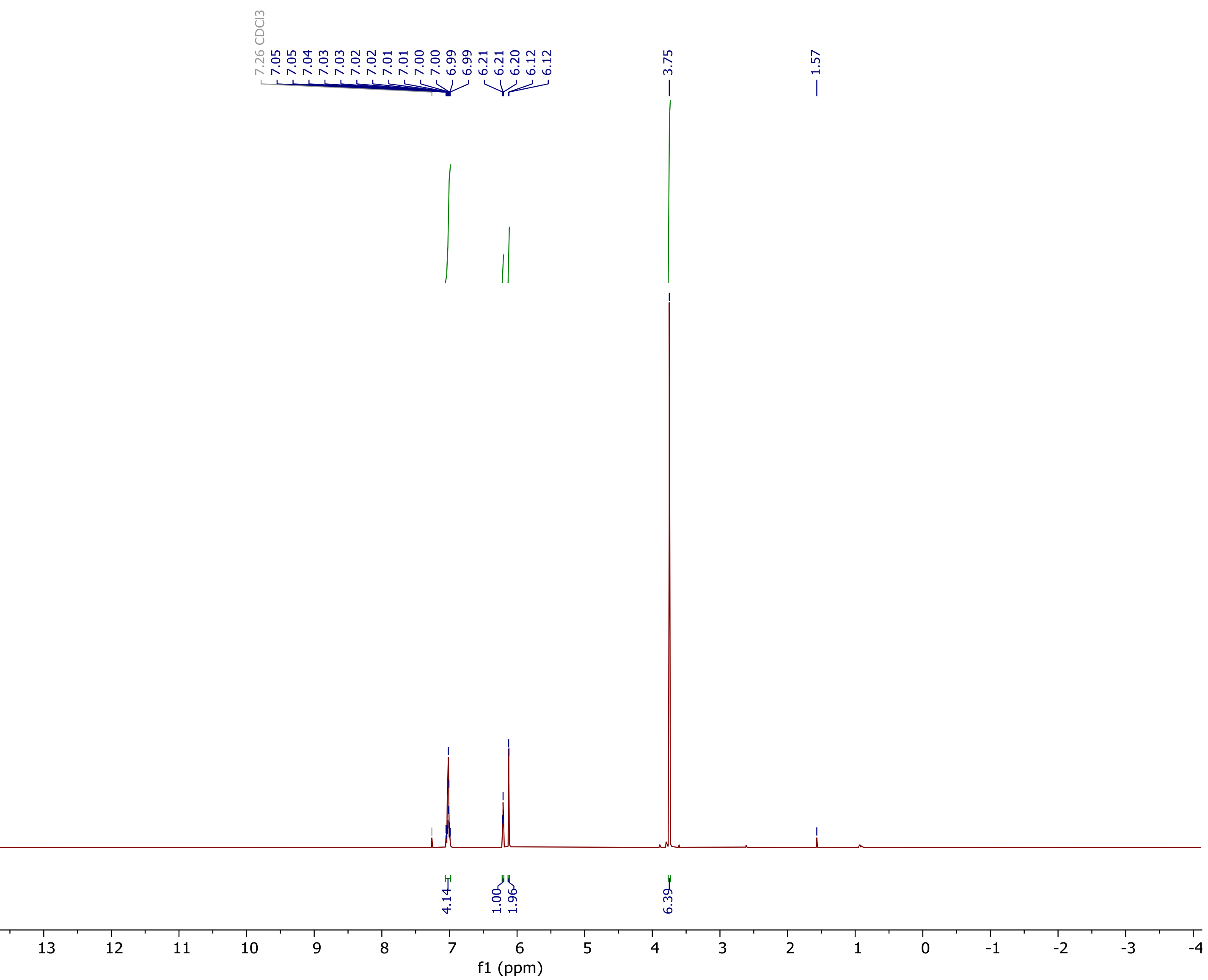

29 – $^{13}C\{^{1}H\}$ NMR (126 MHz, $CDCl_3$)

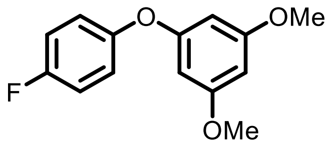

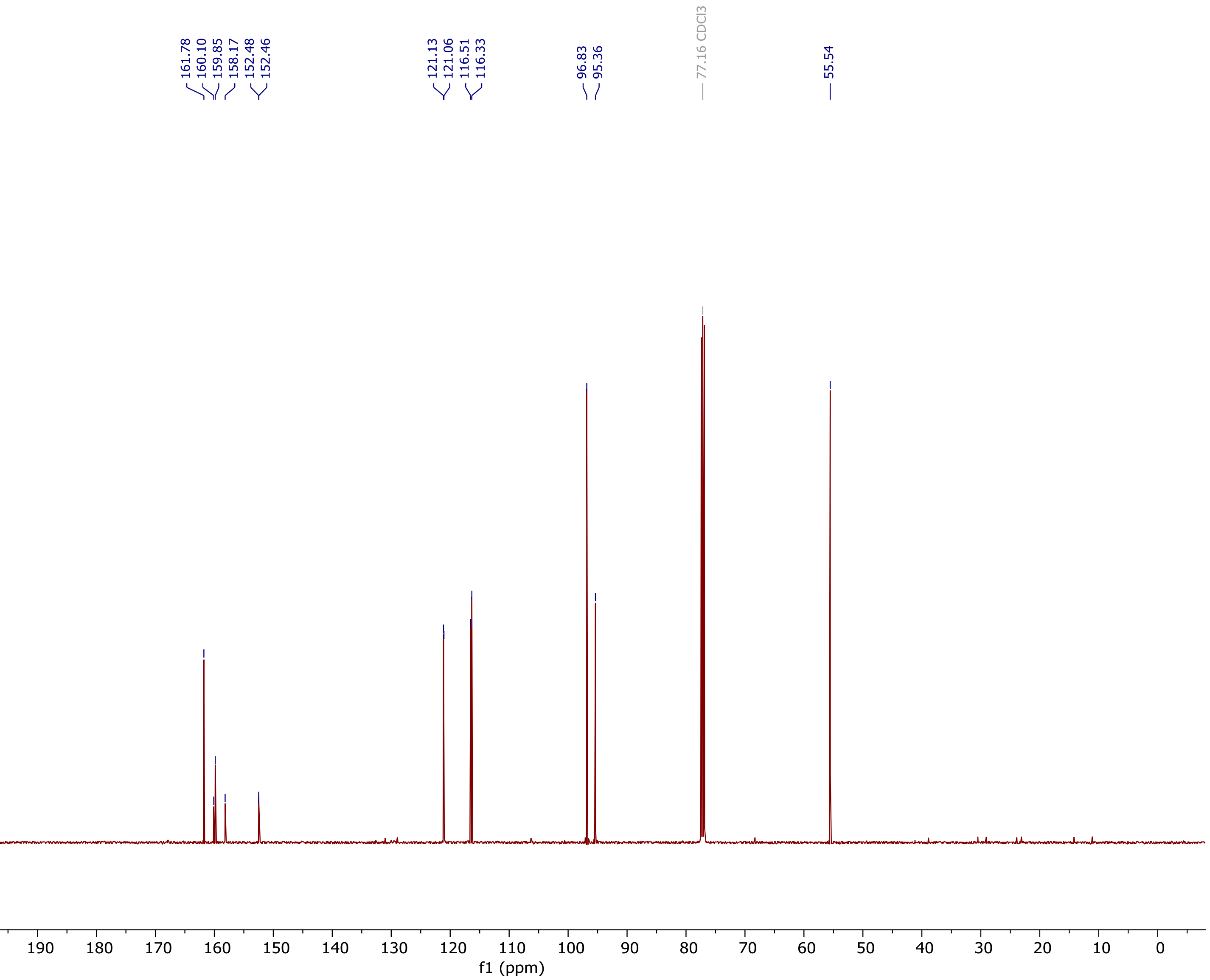

29 – ^{19}F NMR (376 MHz, $CDCl_3$)

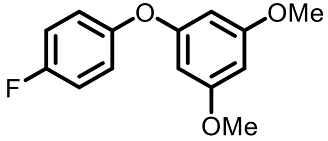

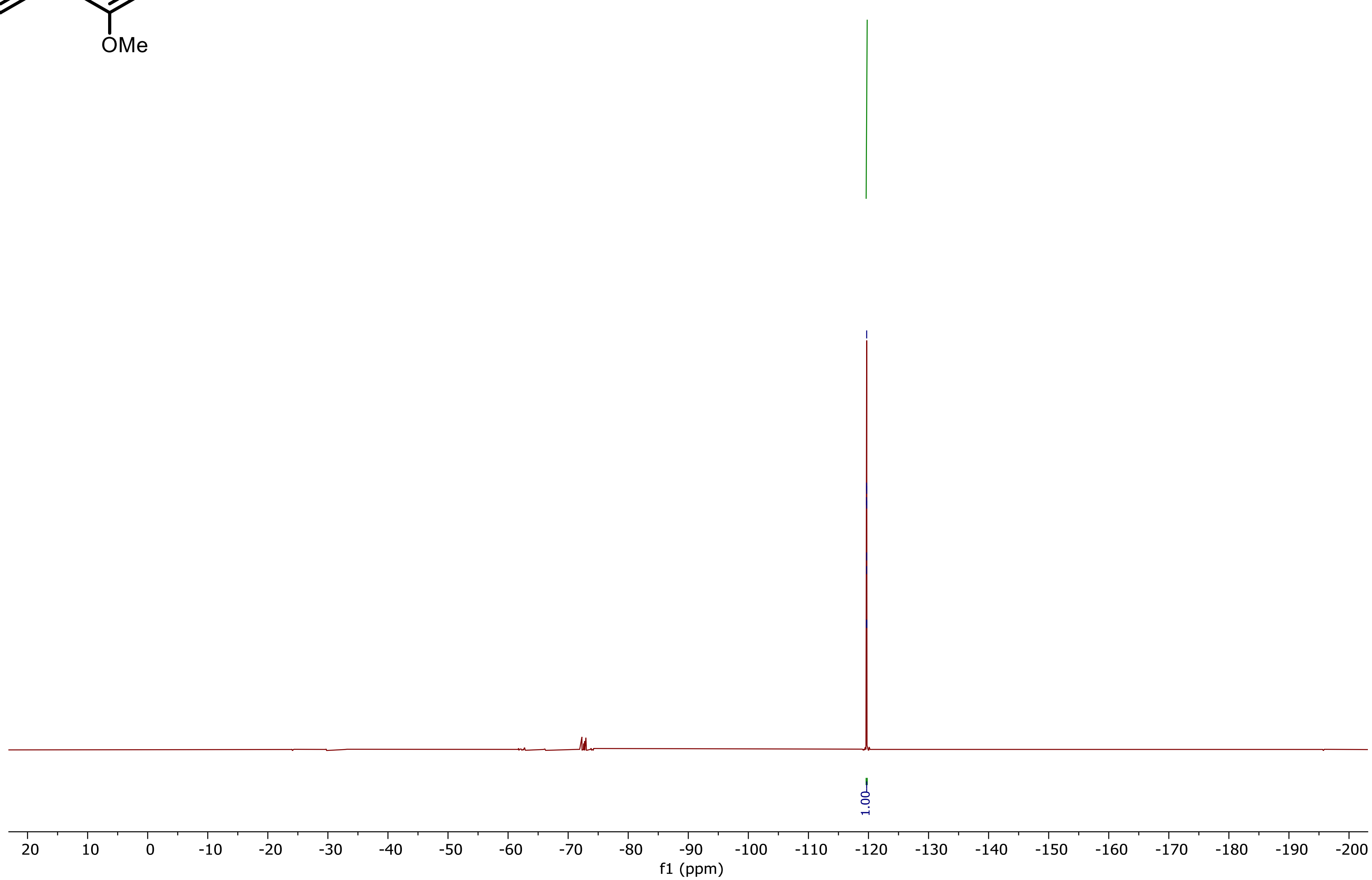

30 - ^{1}H NMR (500 MHz, CDCl$_3$)

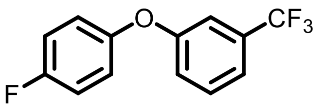

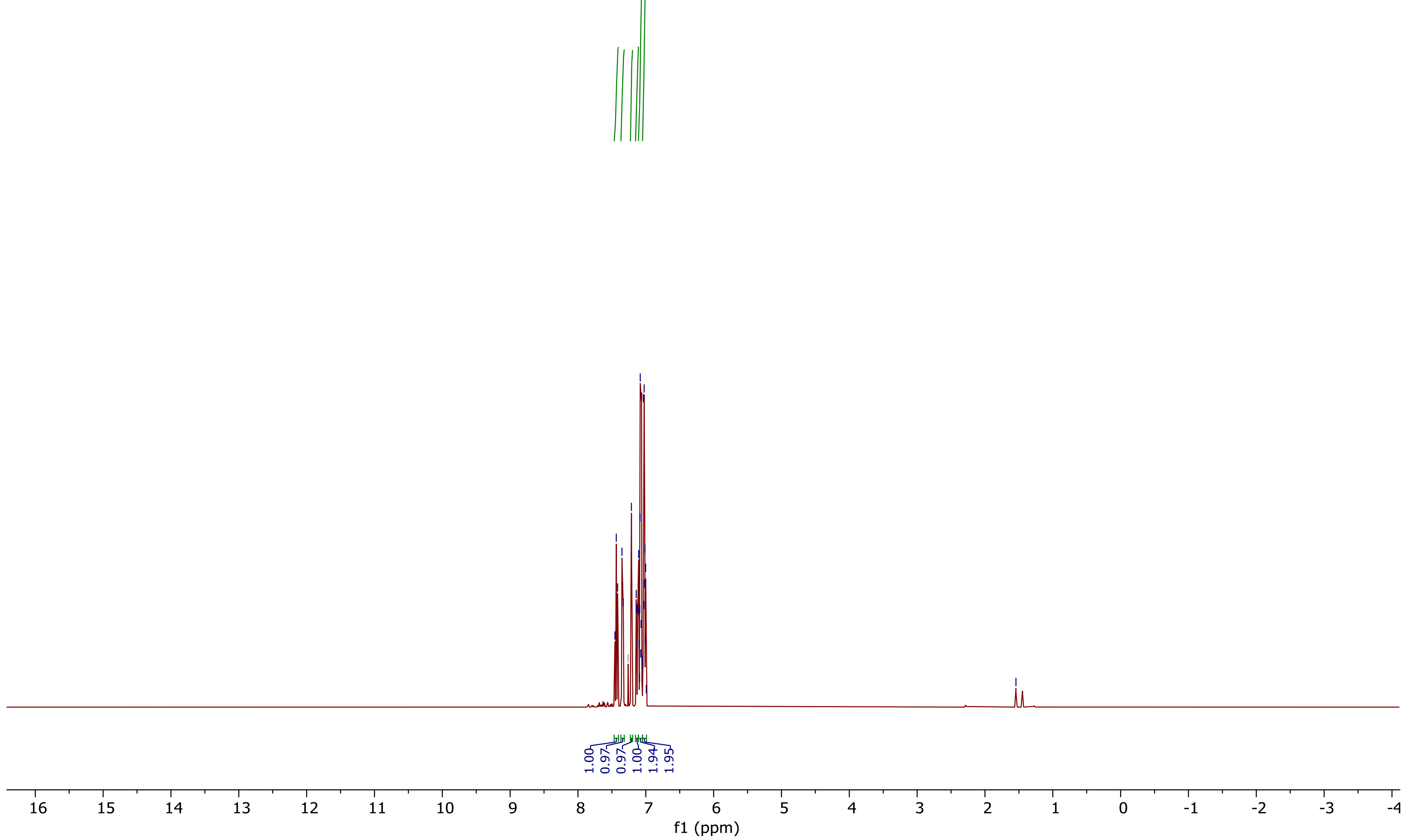

30 - $^{13}C\{^1H\}$ NMR (126 MHz, $CDCl_3$)

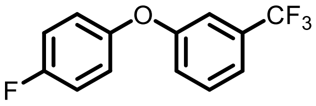

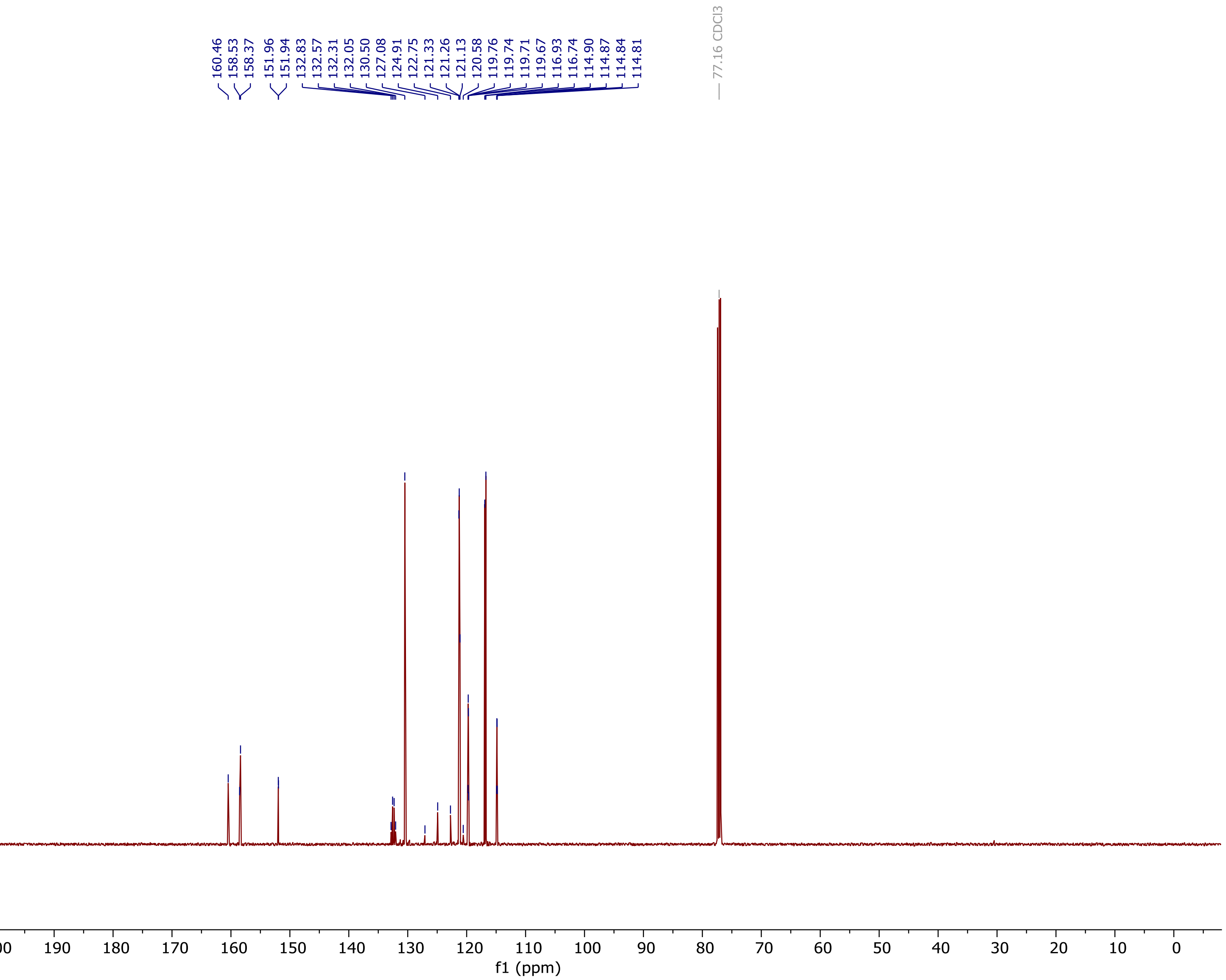

30 - ^{19}F NMR (377 MHz, CDCl$_3$)

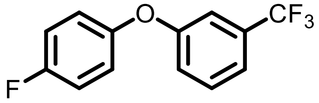

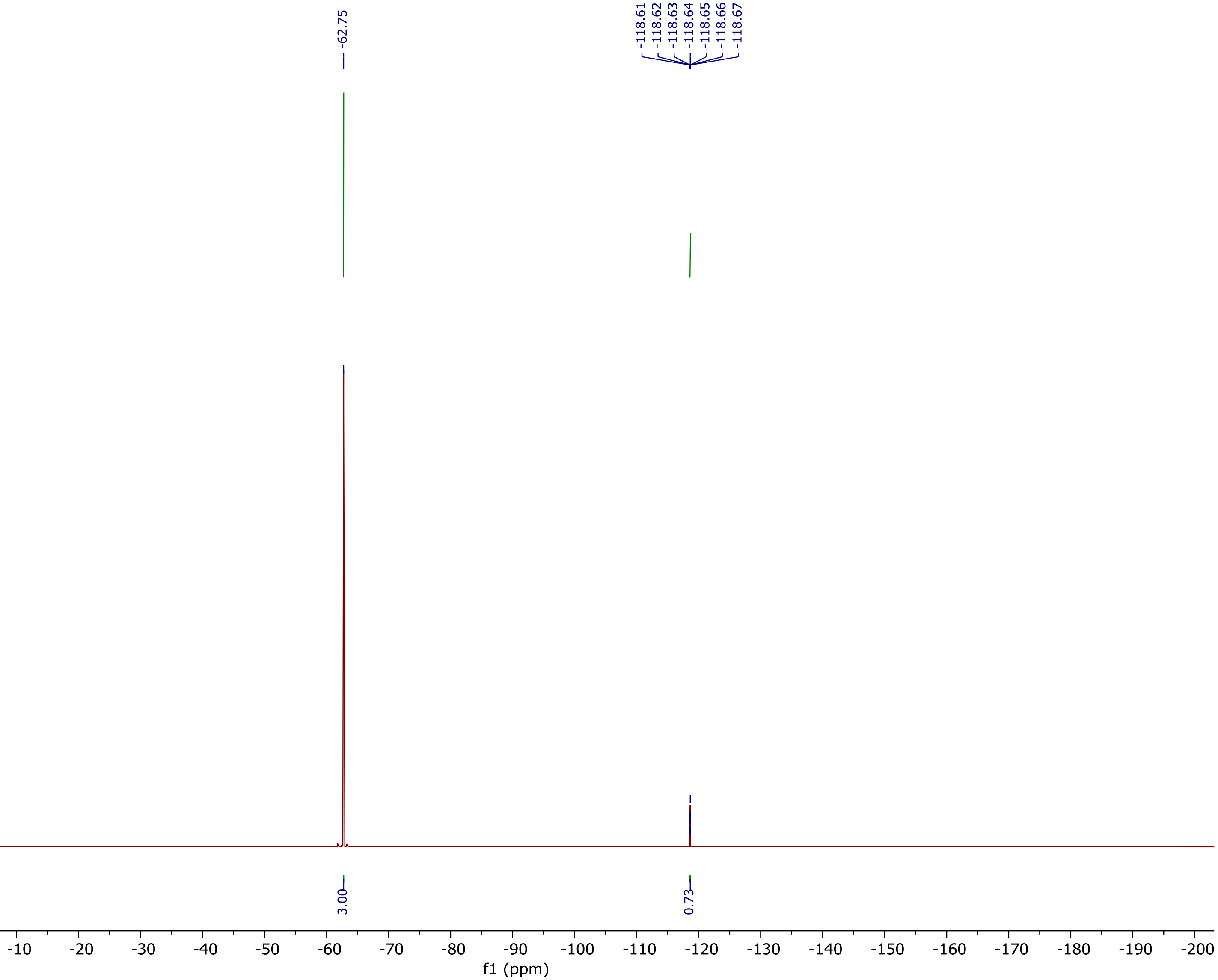

31 - ^{1}H NMR (500 MHz, $CDCl_3$) - *note: contains ca 1% N-Me carbazole, from Pd precatalyst*

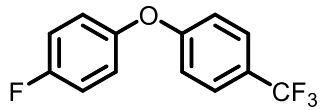

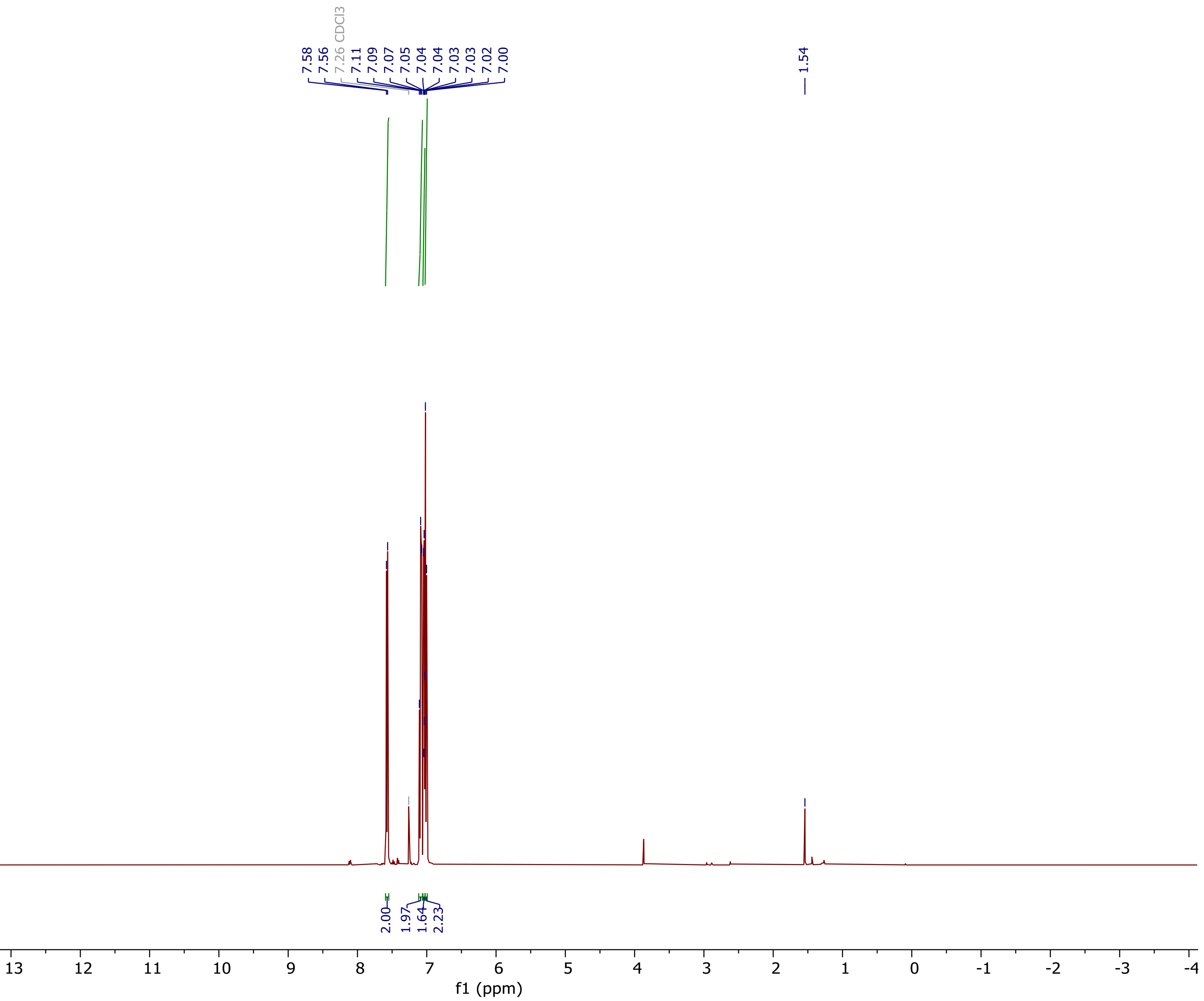

31 - $^{13}C\{^{1}H\}$ NMR (126 MHz, $CDCl_3$) - *note: contains ca 1% N-Me carbazole, from Pd precatalyst*

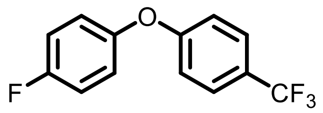

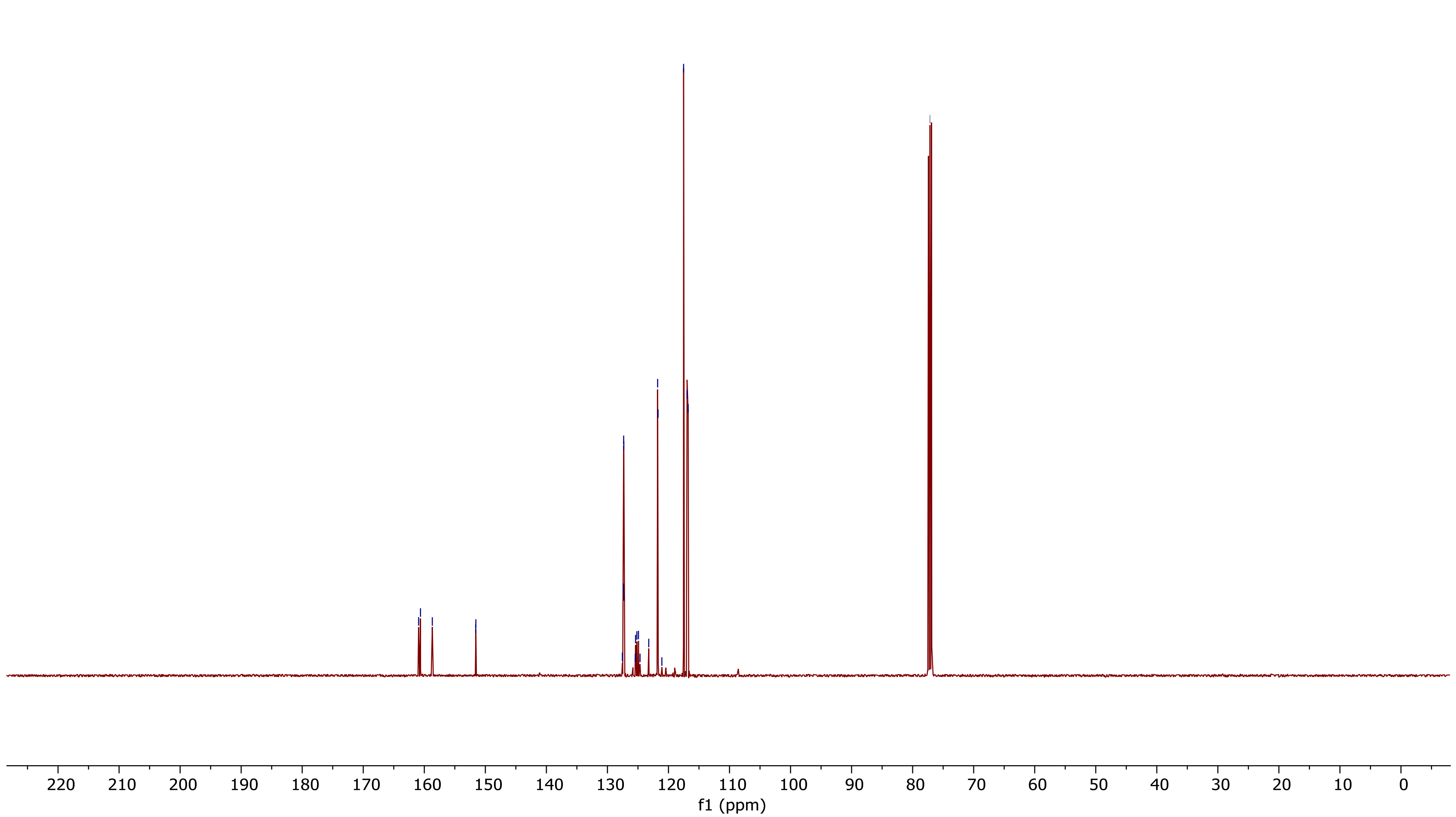

31 - ^{19}F NMR (376 MHz, $CDCl_3$)

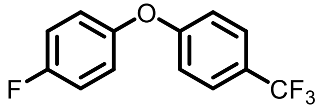

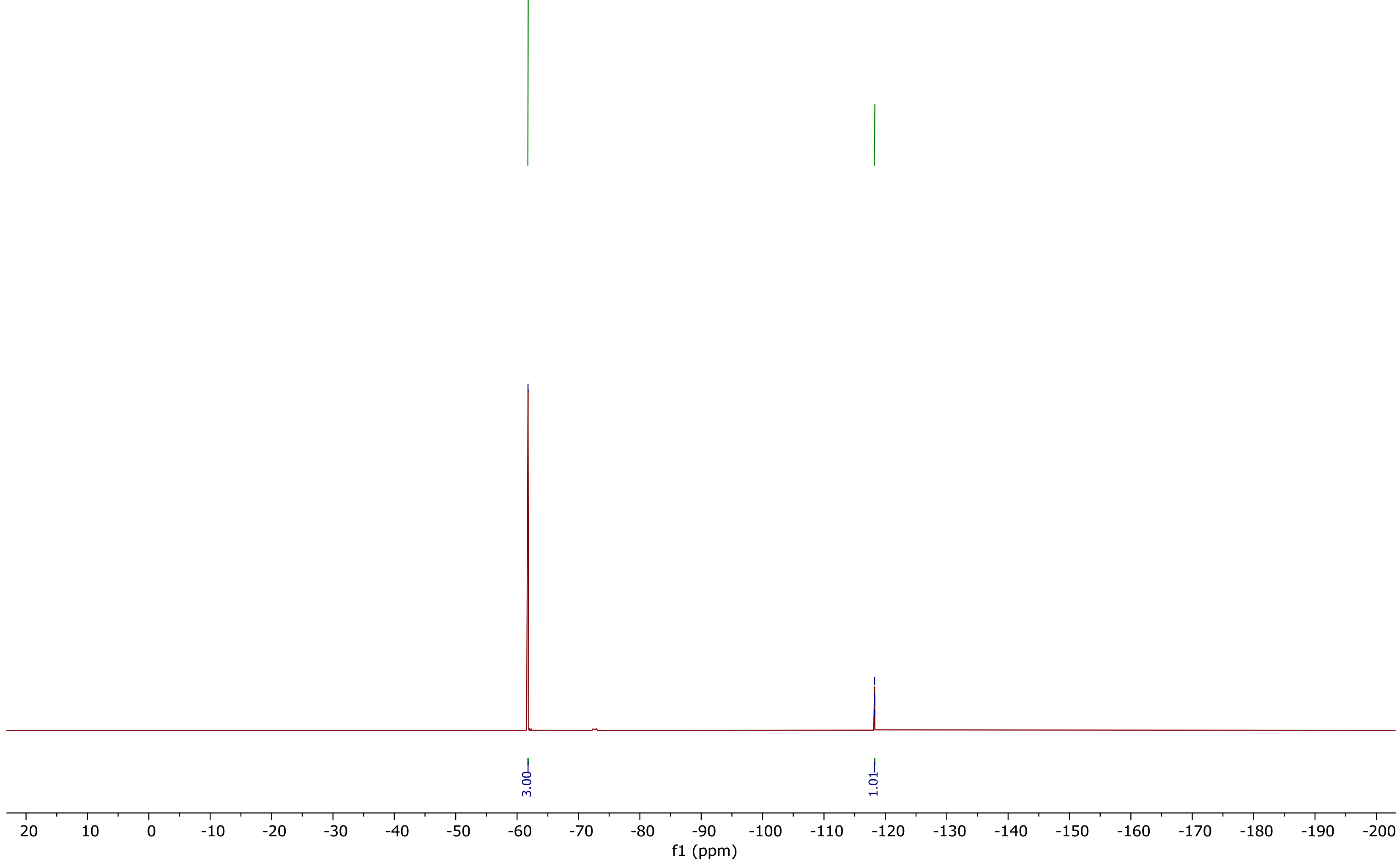

32 - ^{1}H NMR (400 MHz, $CDCl_3$)

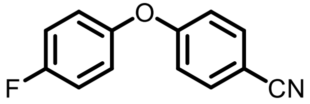

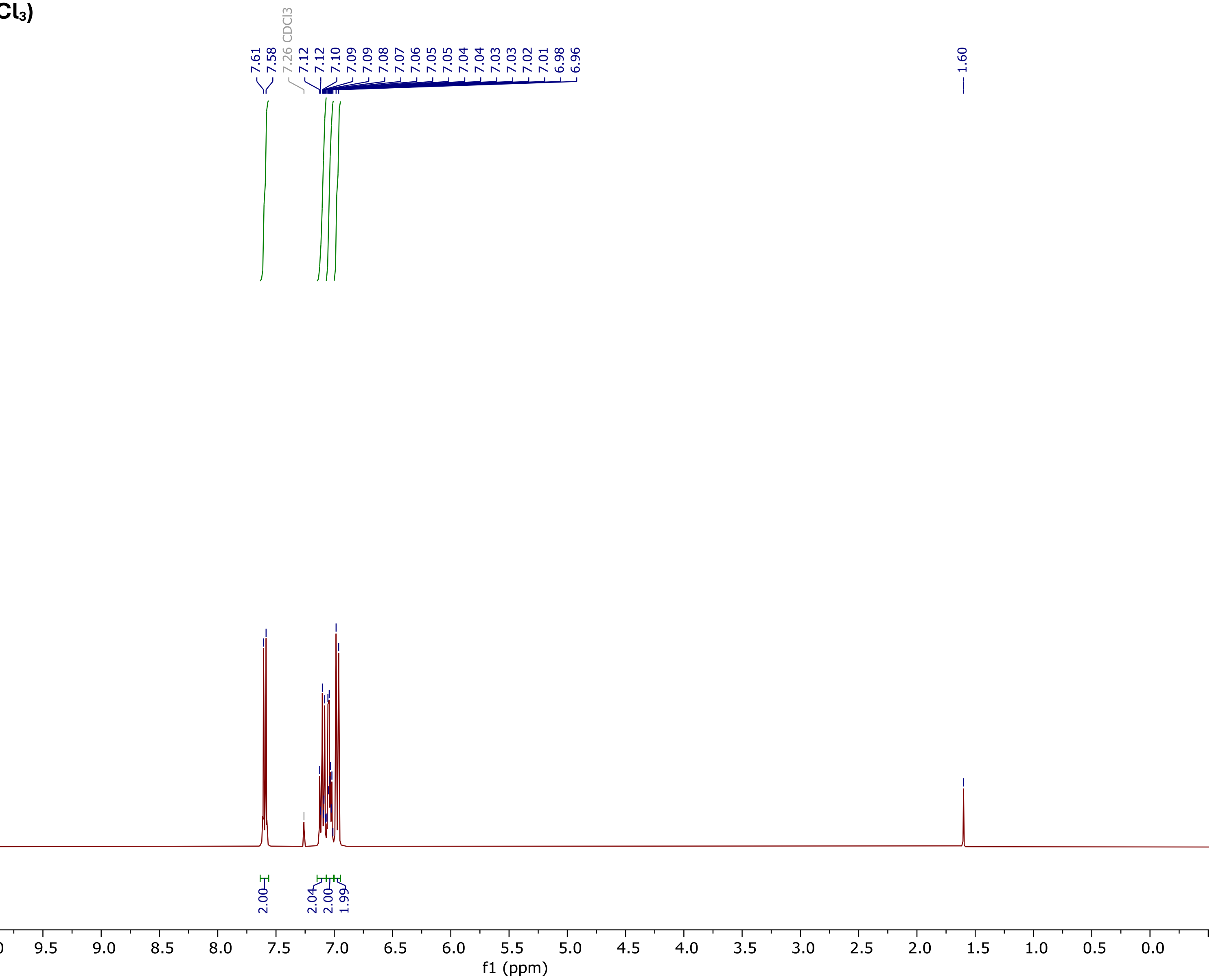

32 - $^{13}C\{^{1}H\}$ NMR (101 MHz, $CDCl_3$)

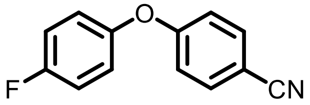

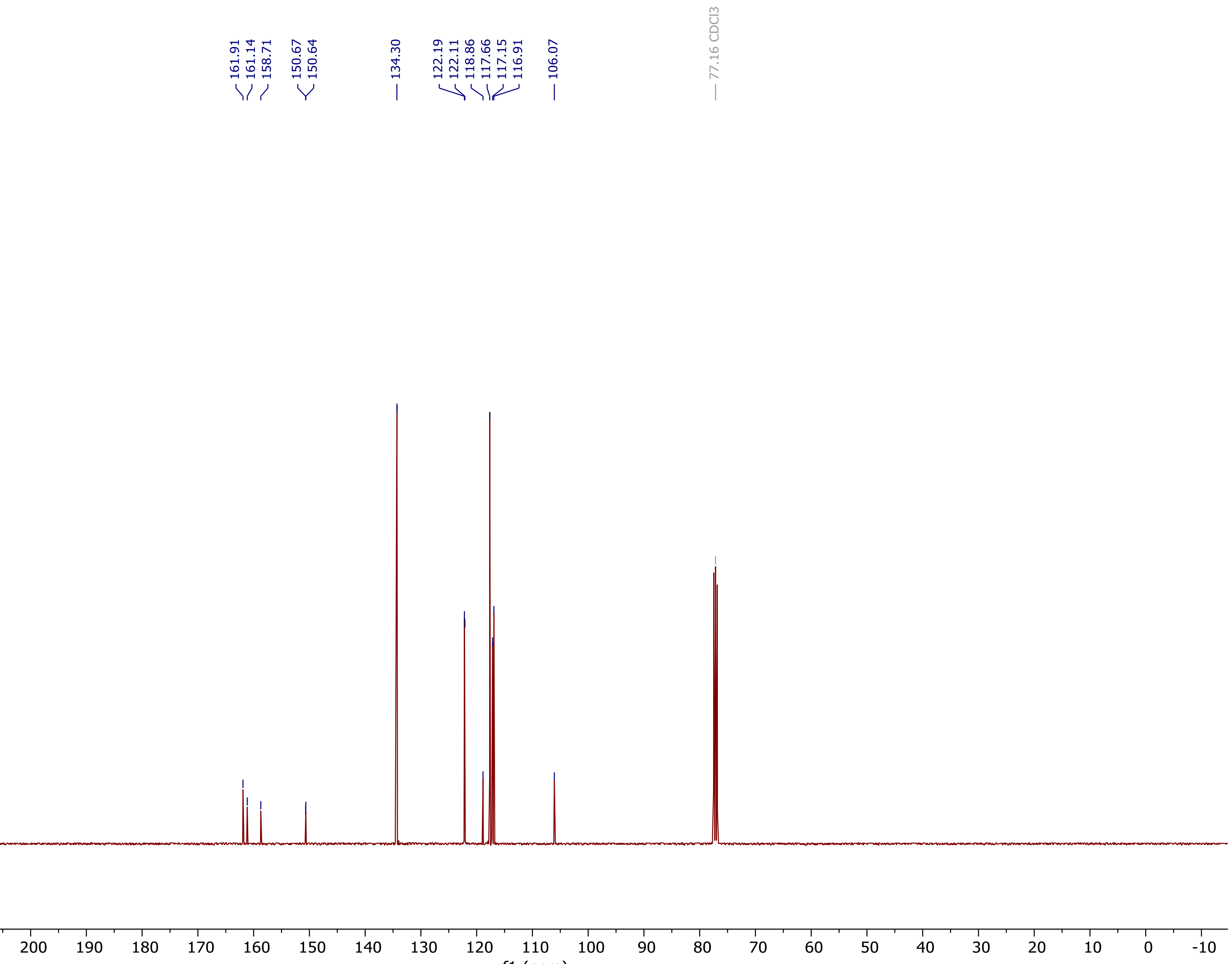

32 - ^{19}F NMR (376 MHz, $CDCl_3$)

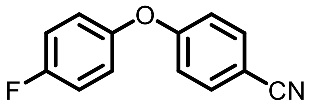

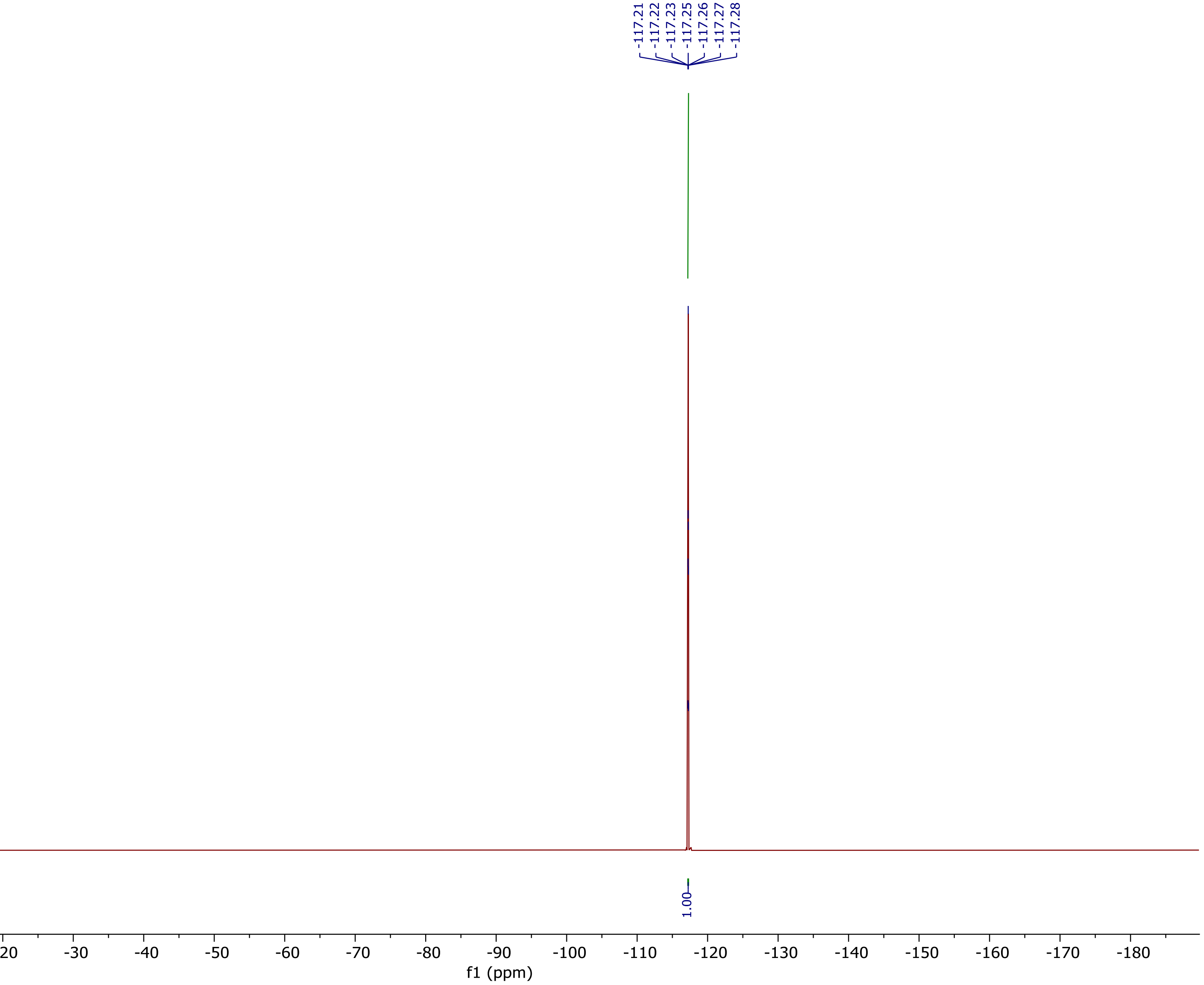

33 - ^{1}H NMR (400 MHz, $CDCl_3$)

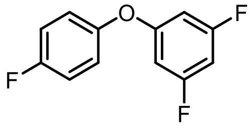

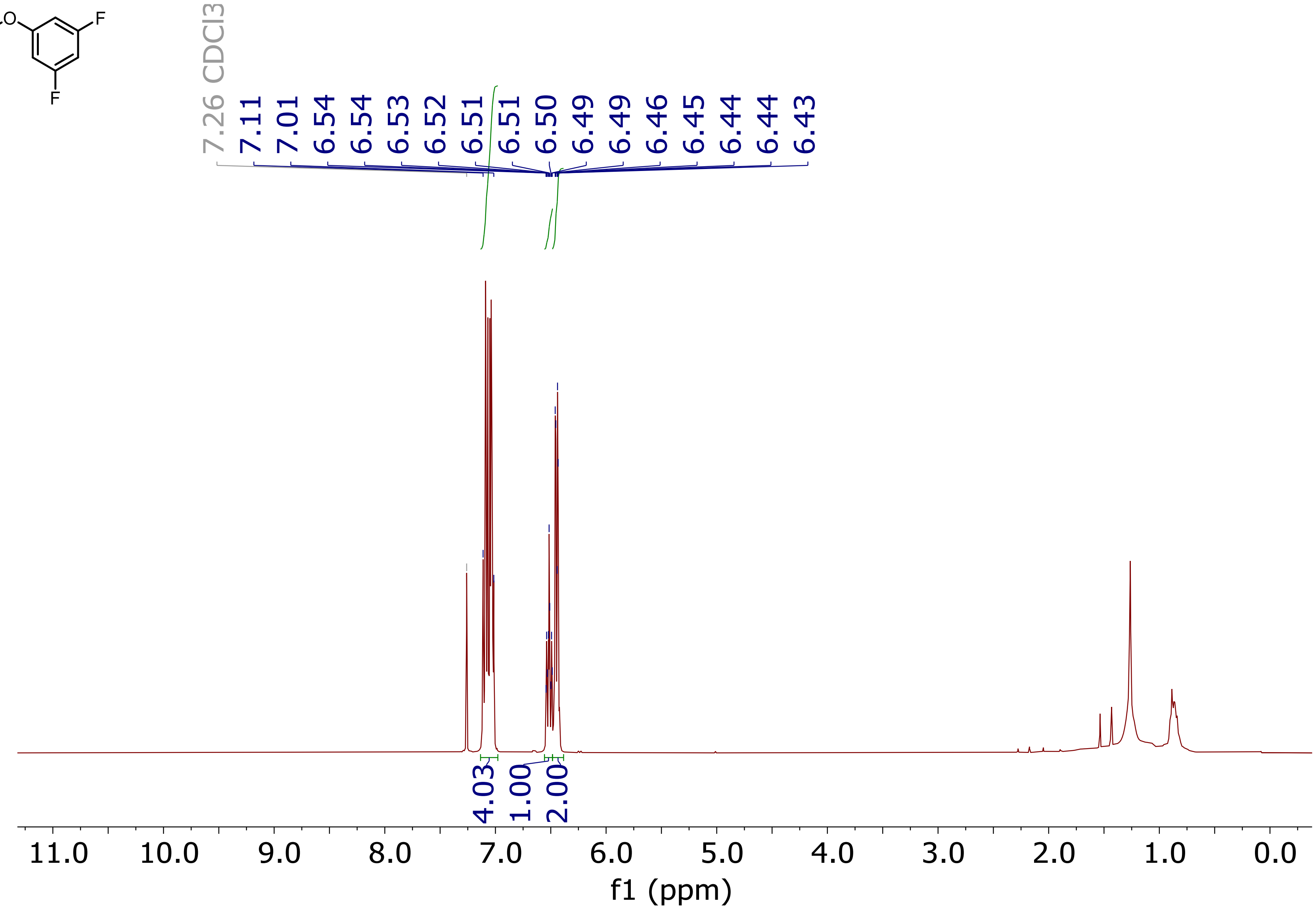

33 - $^{13}C\{^1H\}$ NMR (101 MHz, CDCl$_3$)

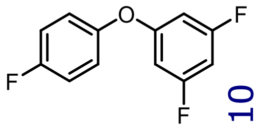

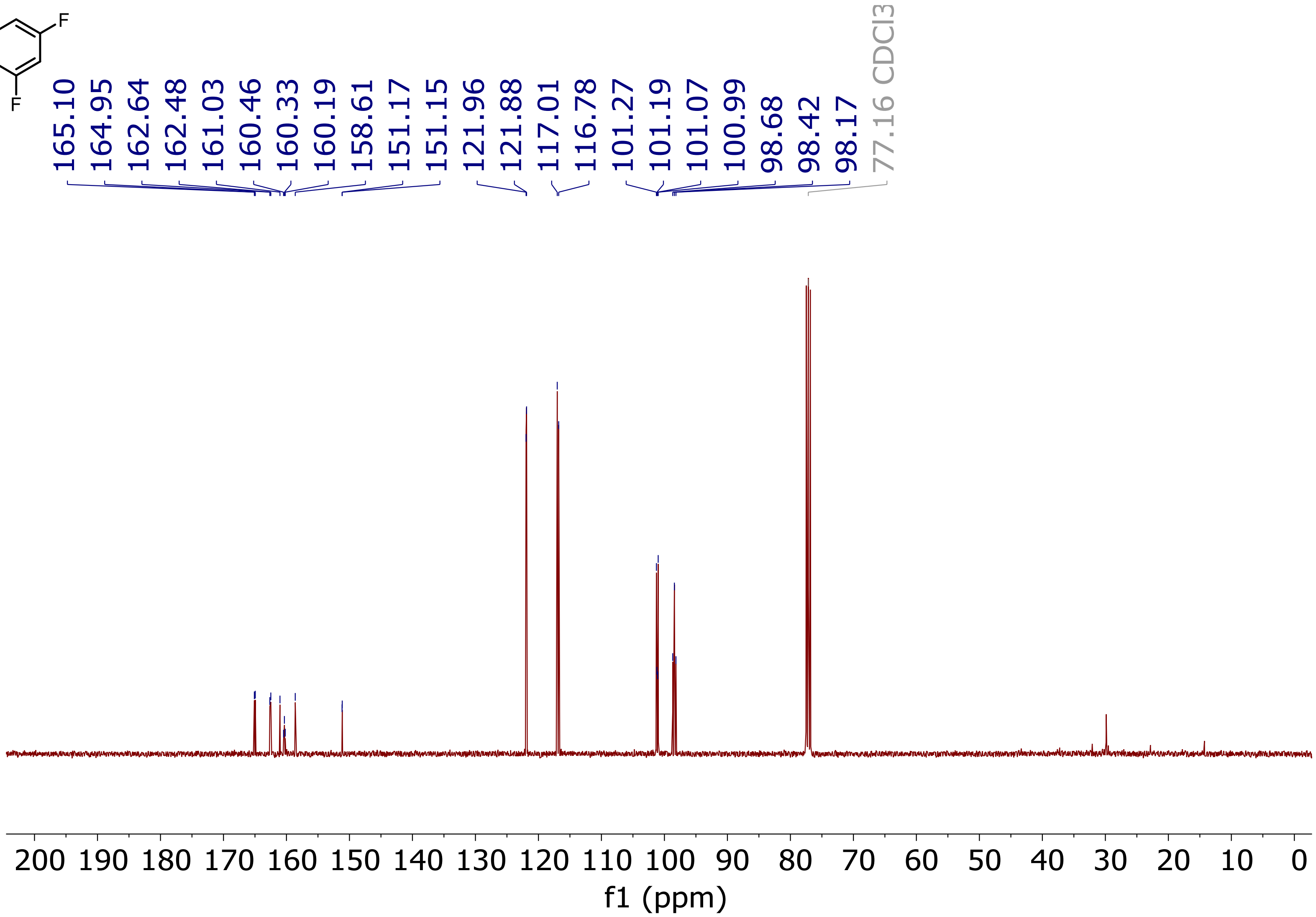

33 - ^{19}F NMR (376 MHz, $CDCl_3$)

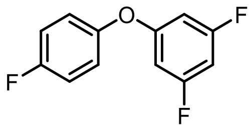

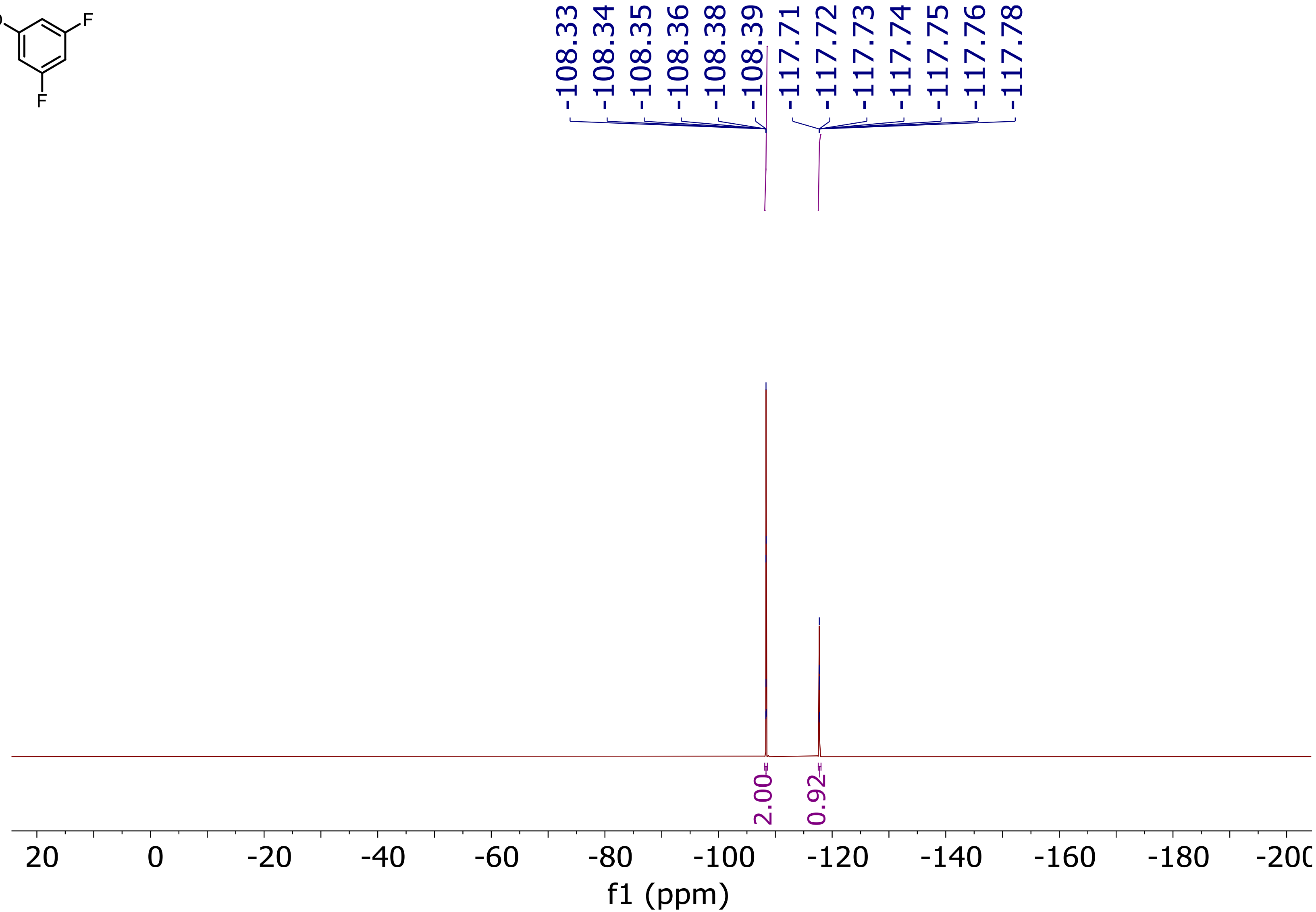

34 - ^{1}H NMR (400 MHz, $CDCl_3$)

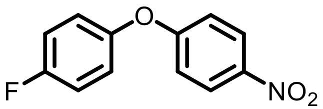

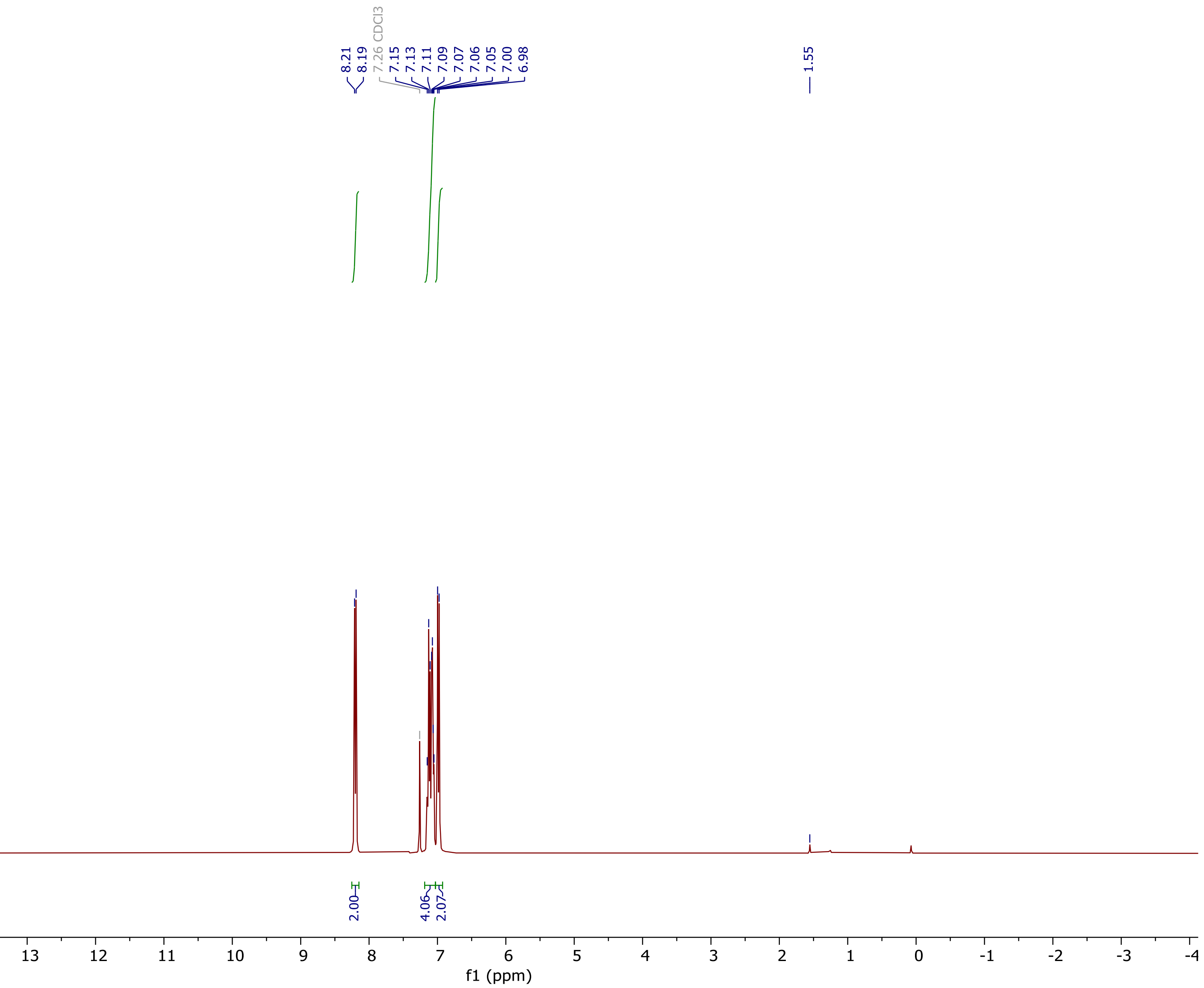

34 - $^{13}C\{^1H\}$ NMR (101 MHz, $CDCl_3$)

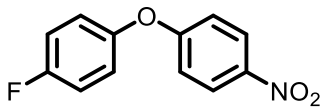

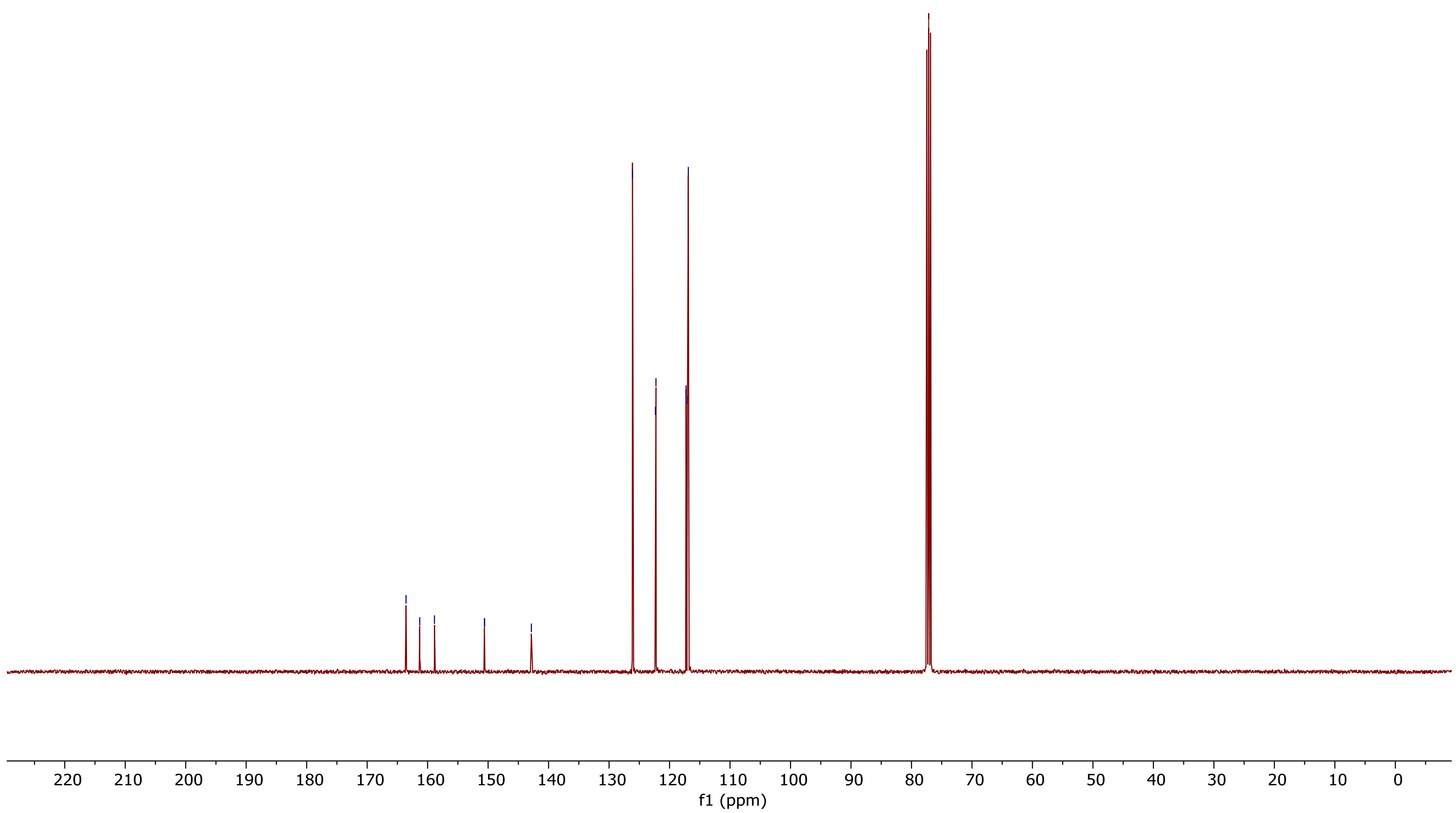

34 - ^{19}F NMR (376 MHz, $CDCl_3$)

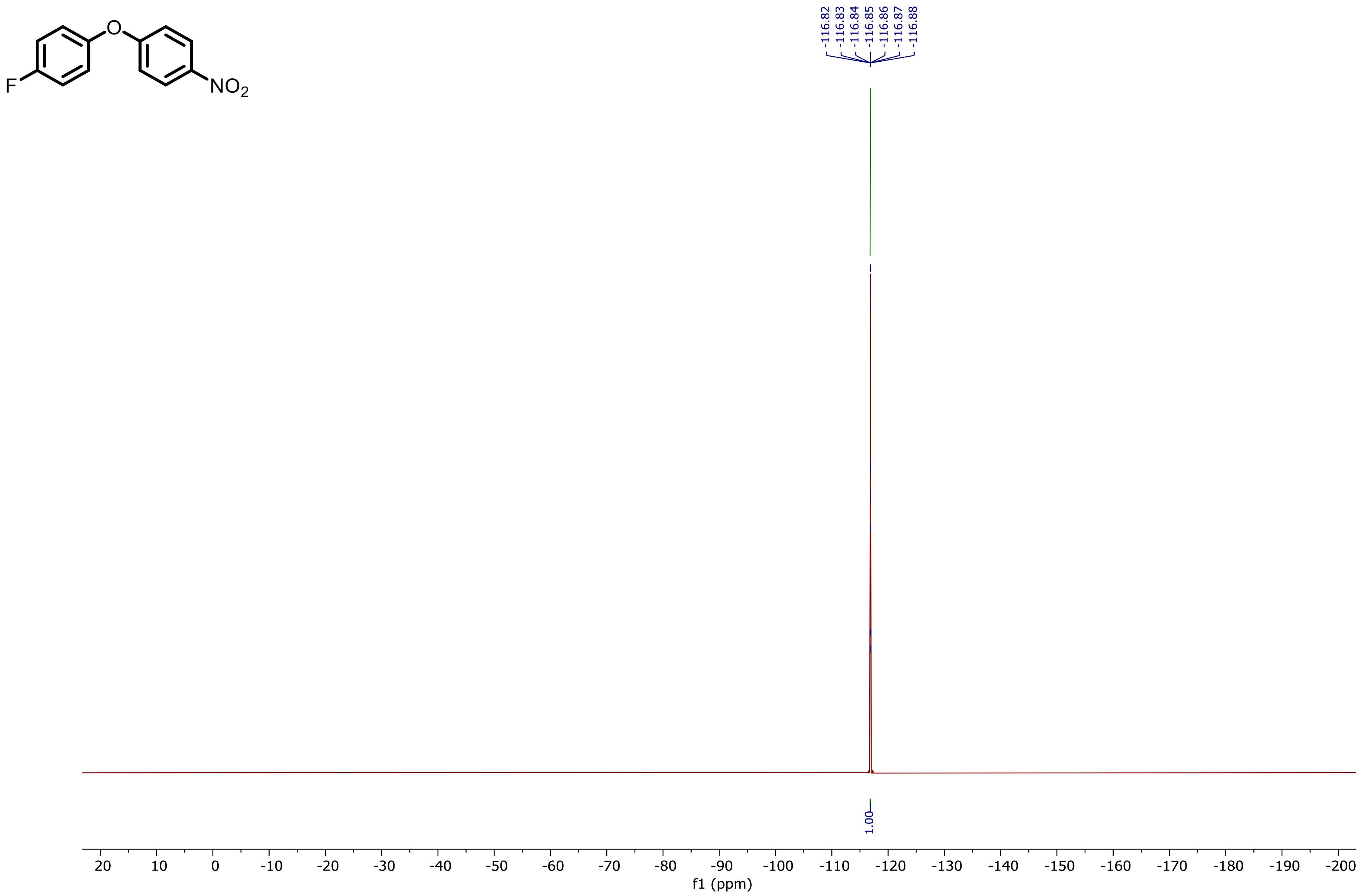

37 - ^{1}H NMR (400 MHz, $CDCl_3$)

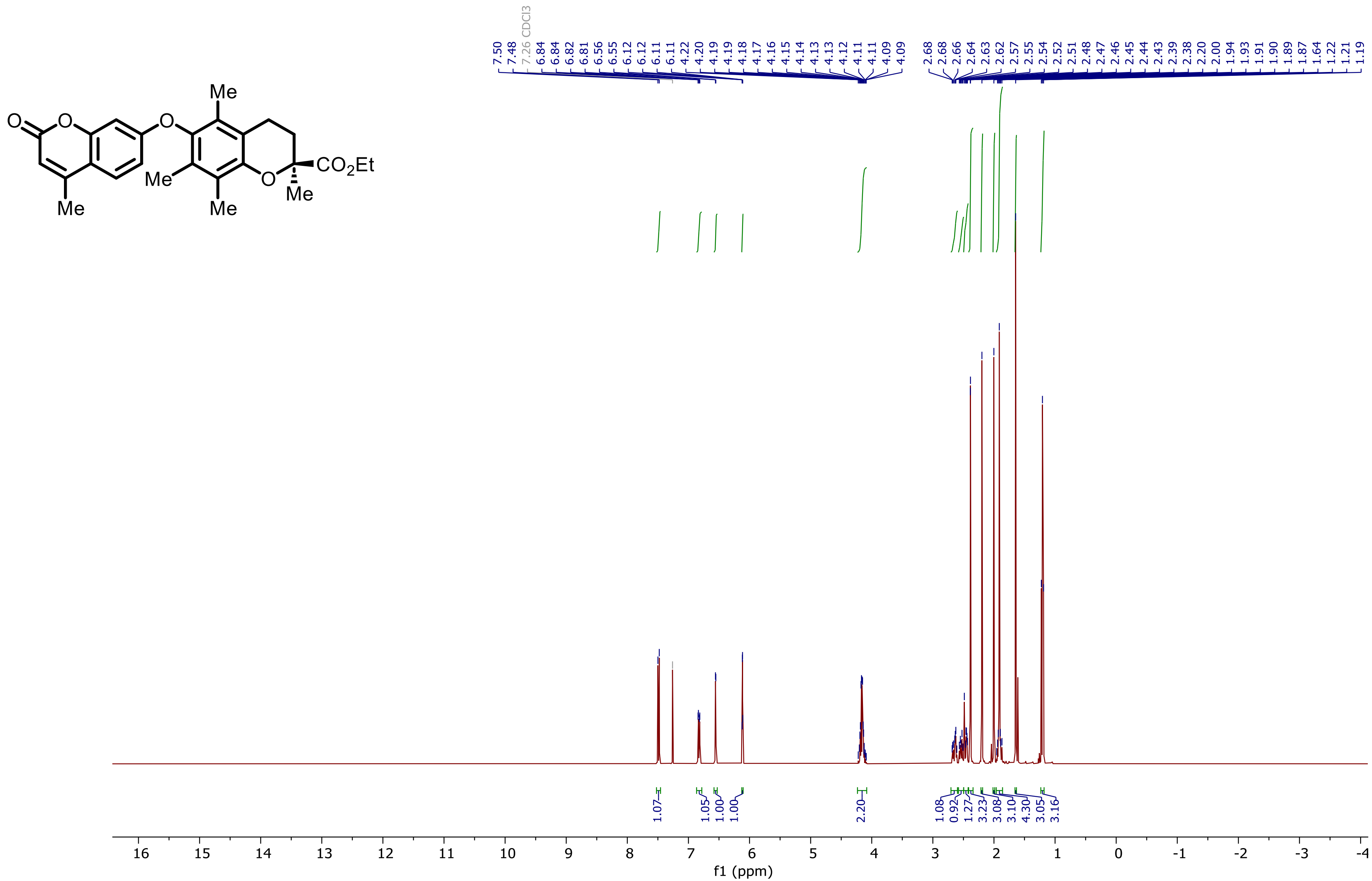

37 - $^{13}C\{^1H\}$ NMR (101 MHz, $CDCl_3$)

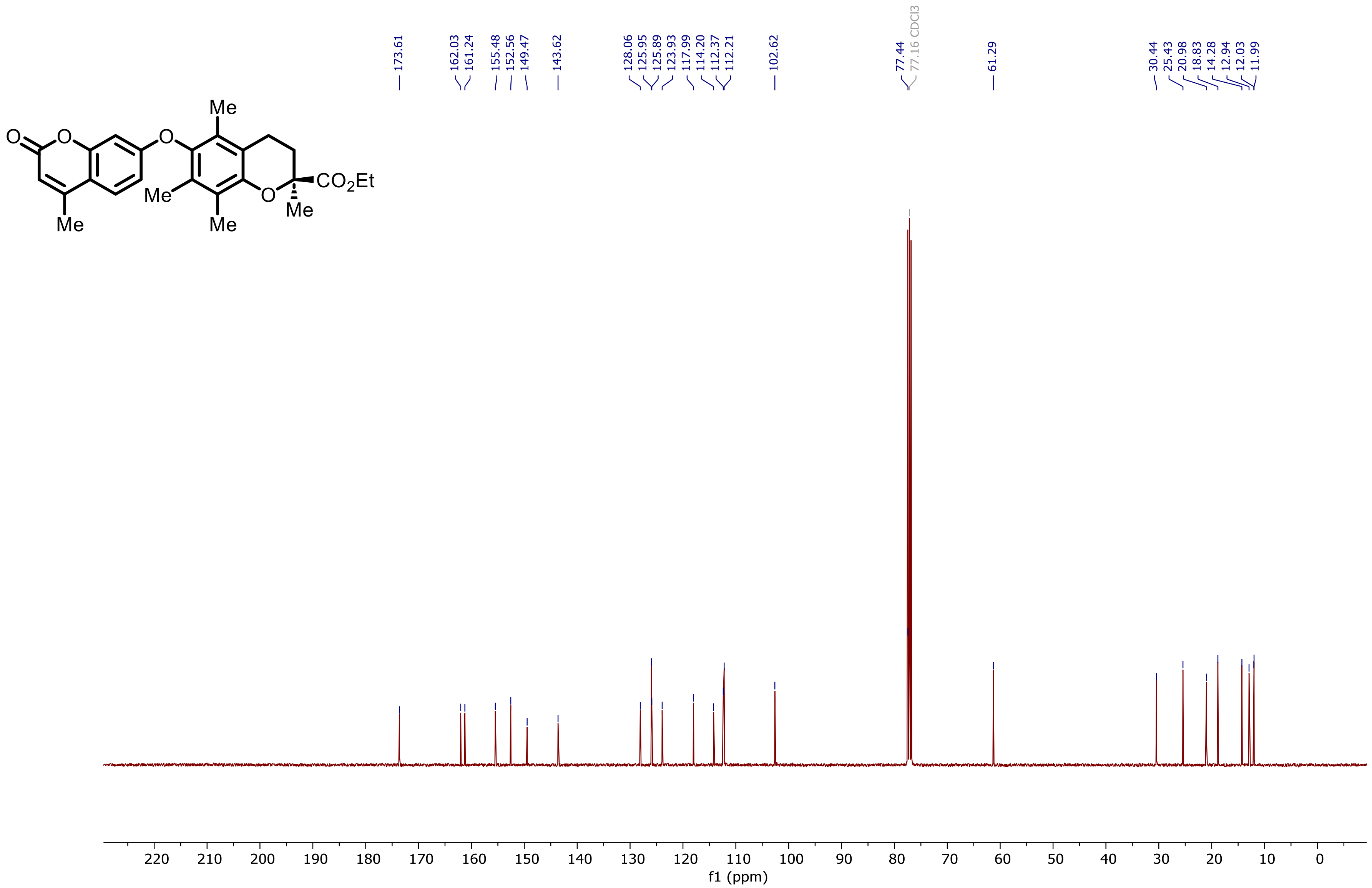

38 - ^{1}H NMR (400 MHz, $CDCl_3$)

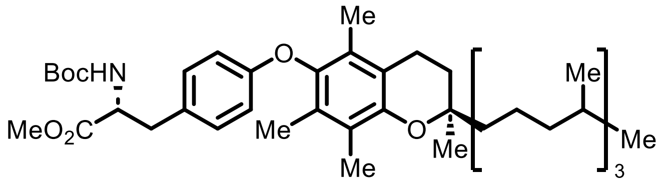

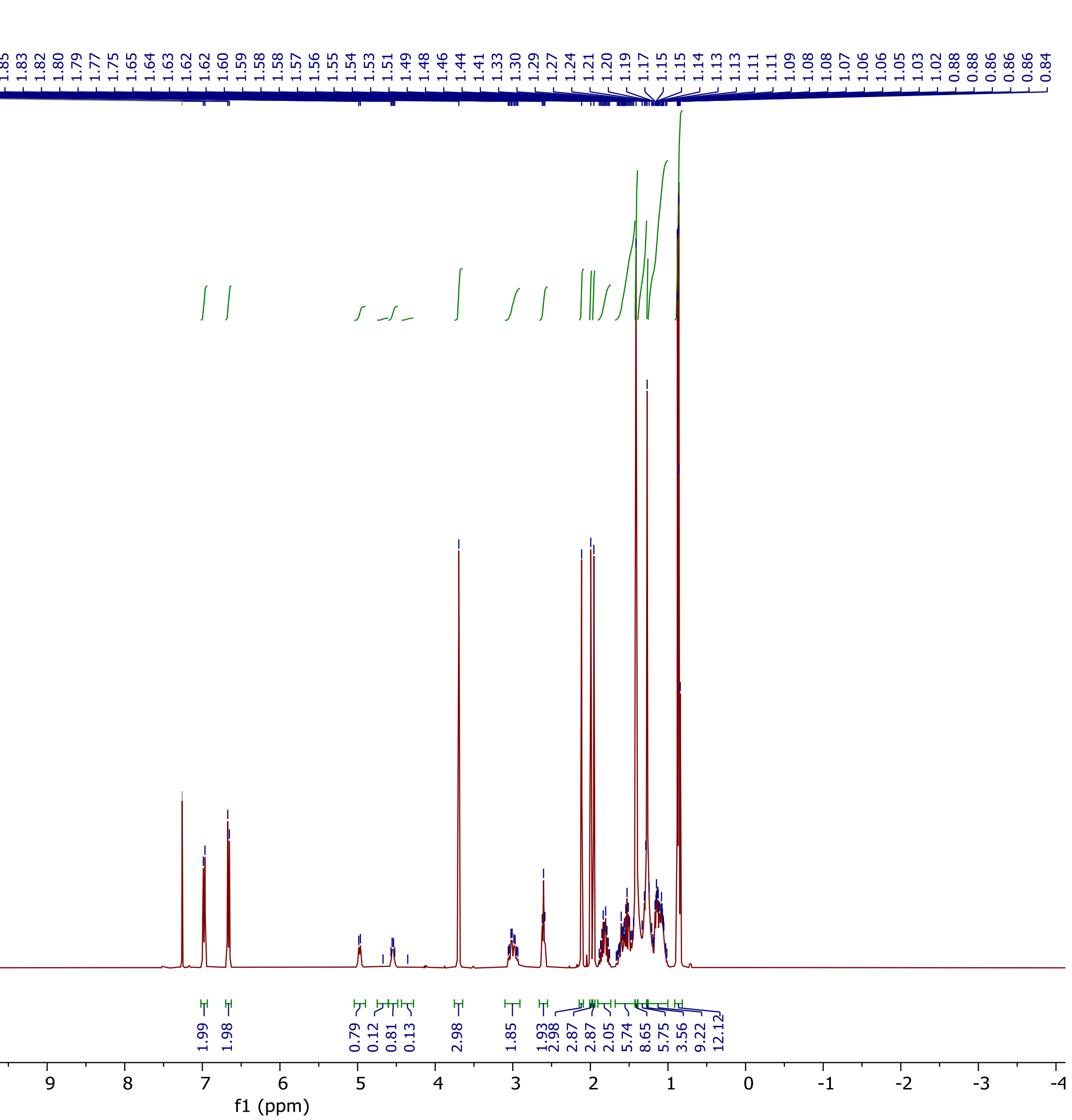

38 - $^{13}C\{^1H\}$ NMR (101 MHz, $CDCl_3$)

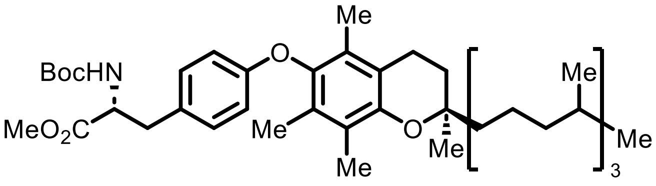

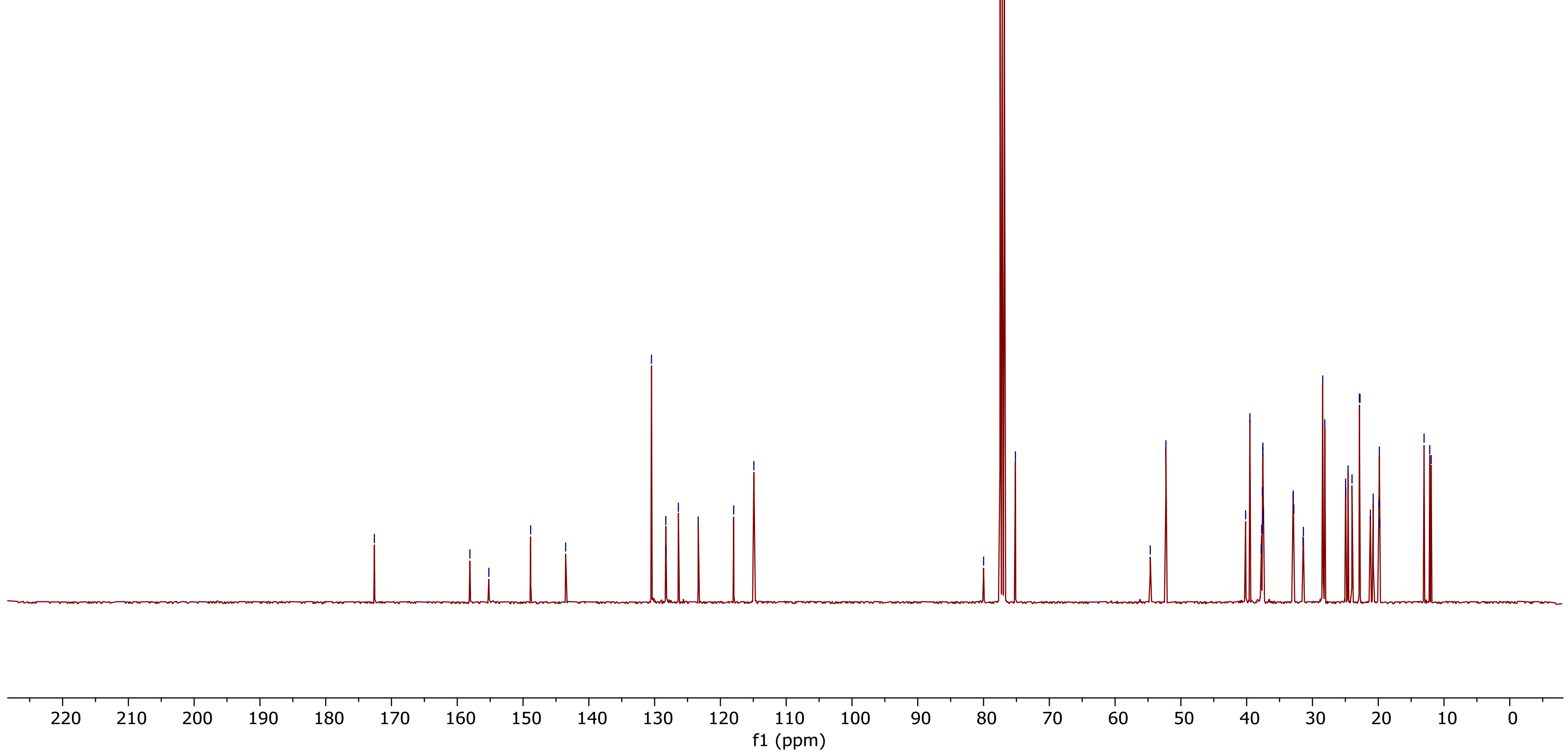

39 - ^{1}H NMR (400 MHz, $CDCl_3$)

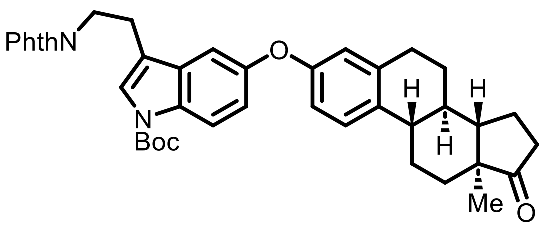

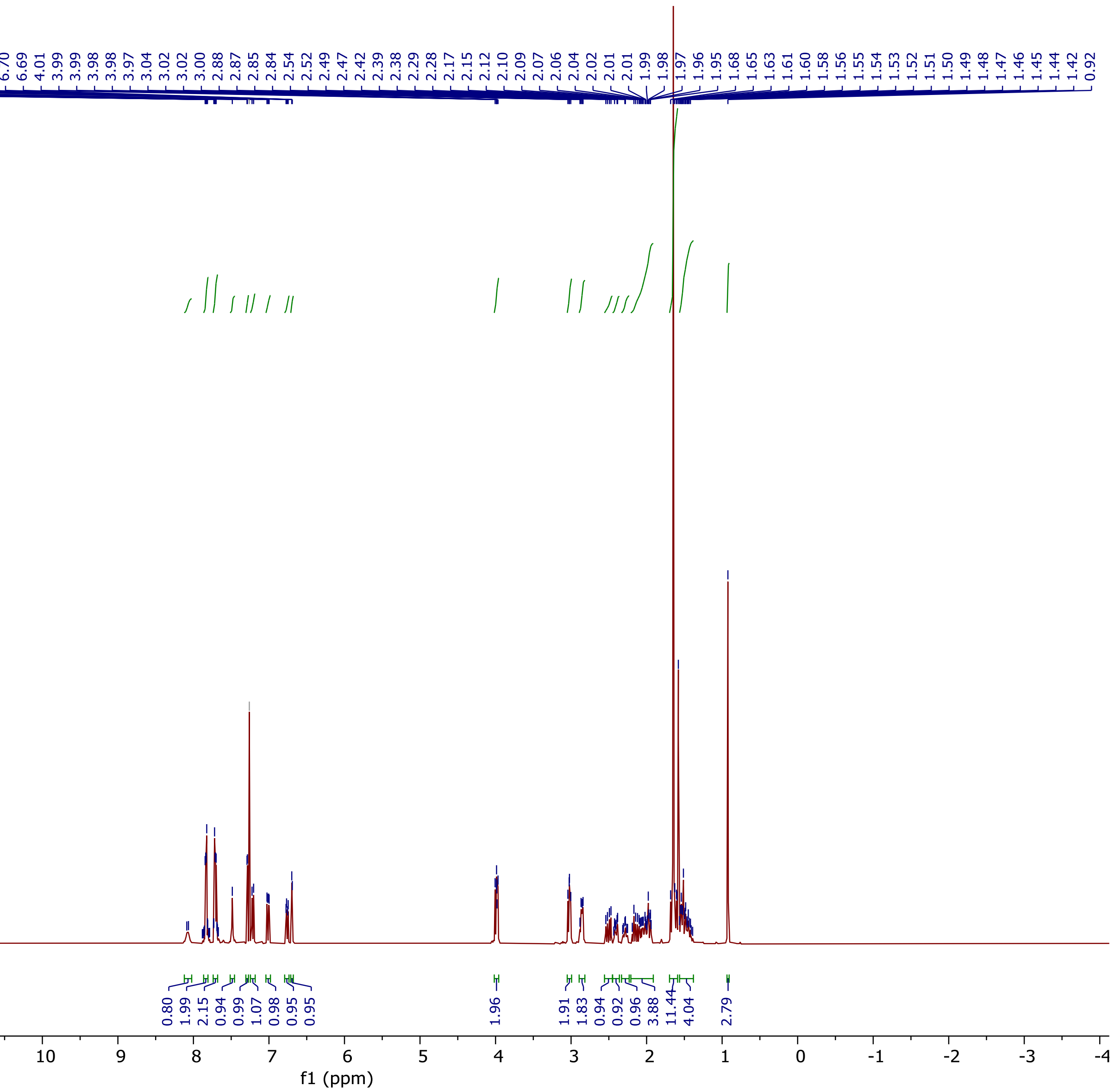

39 - ^{1}H NMR (400 MHz, $CDCl_3$)

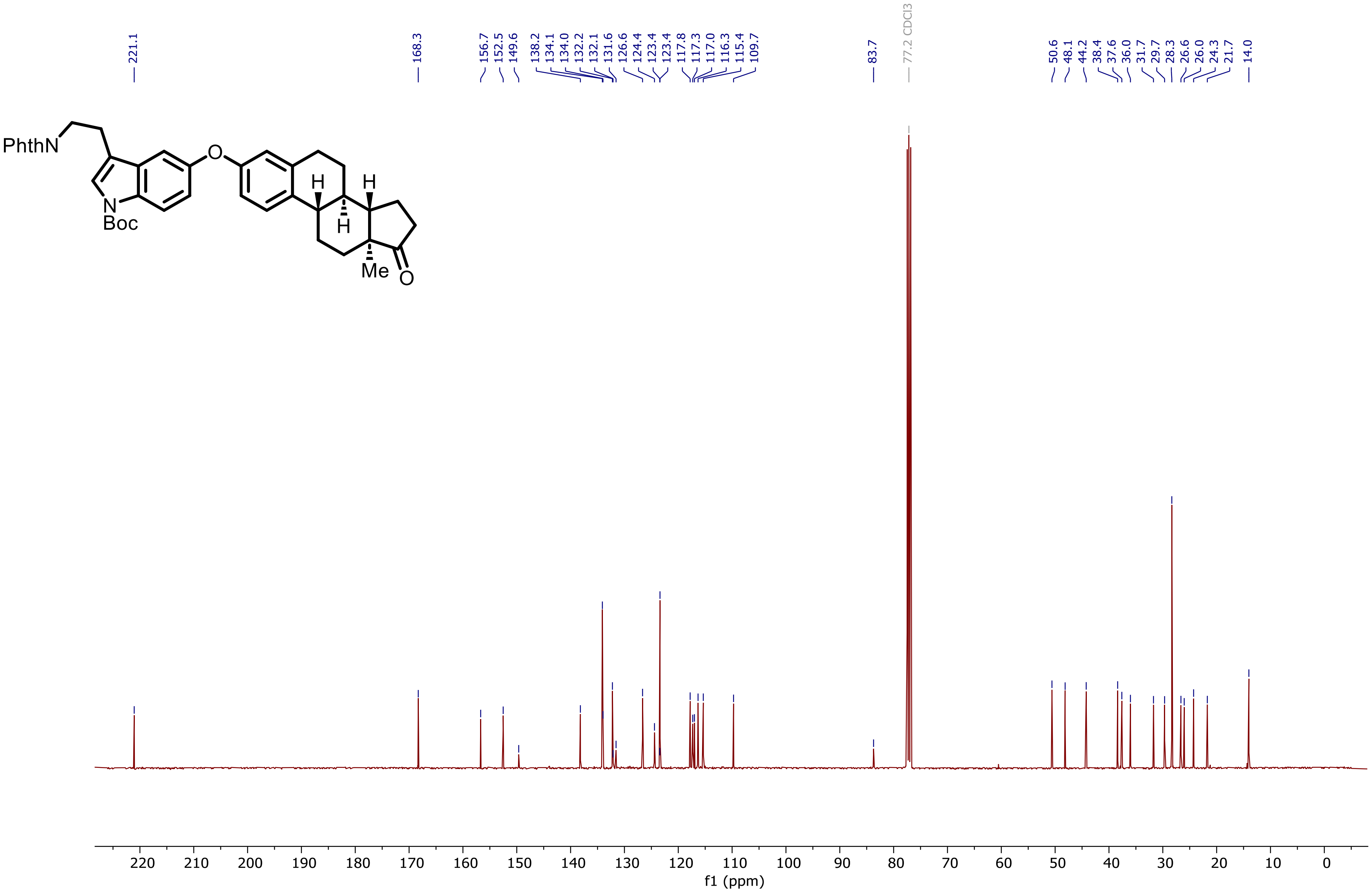

40 – ^{1}H NMR (400 MHz, $CDCl_3$)

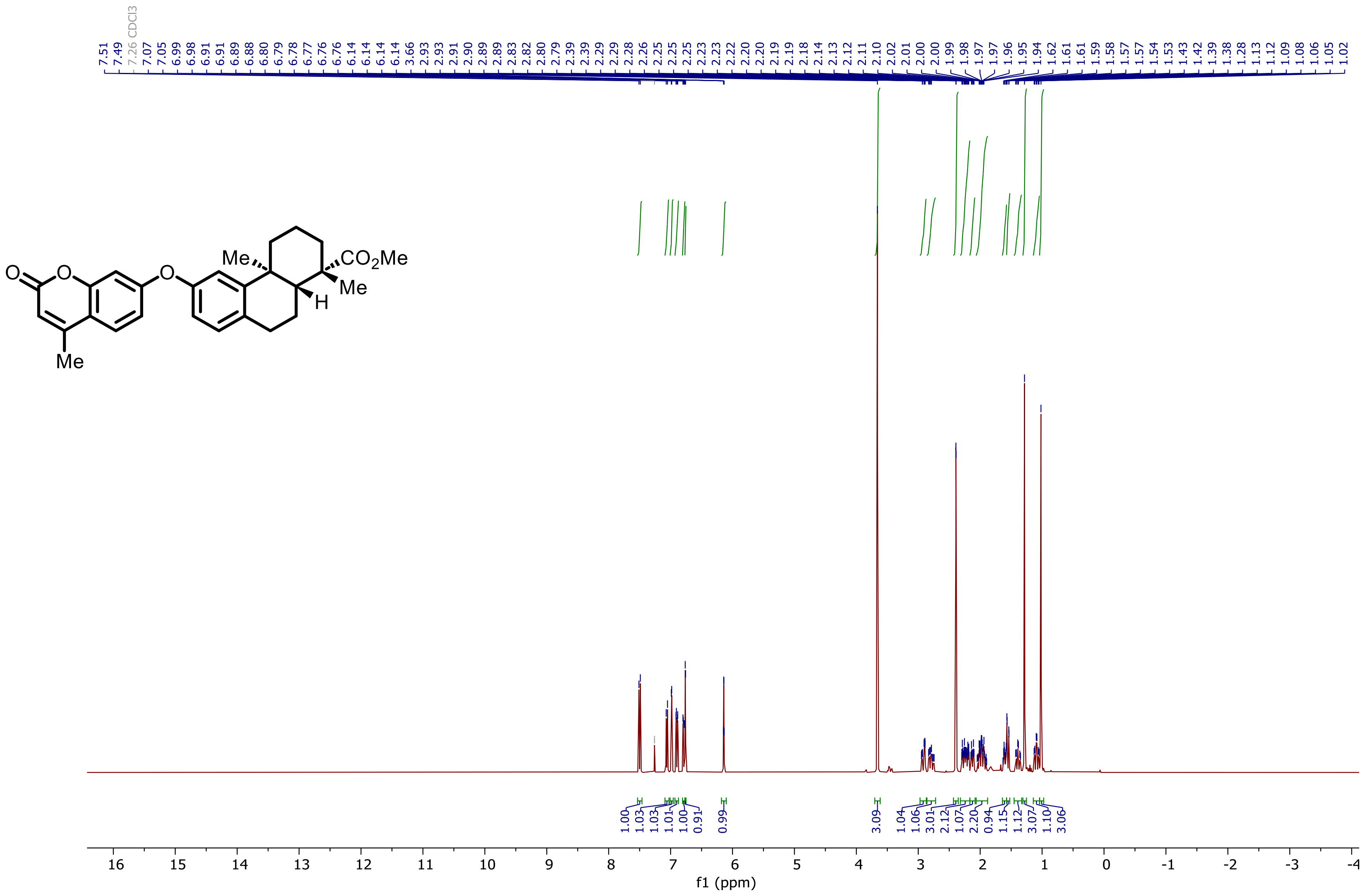

40 – $^{13}C\{^{1}H\}$ NMR (101 MHz, $CDCl_3$)

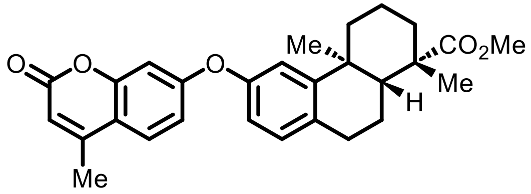

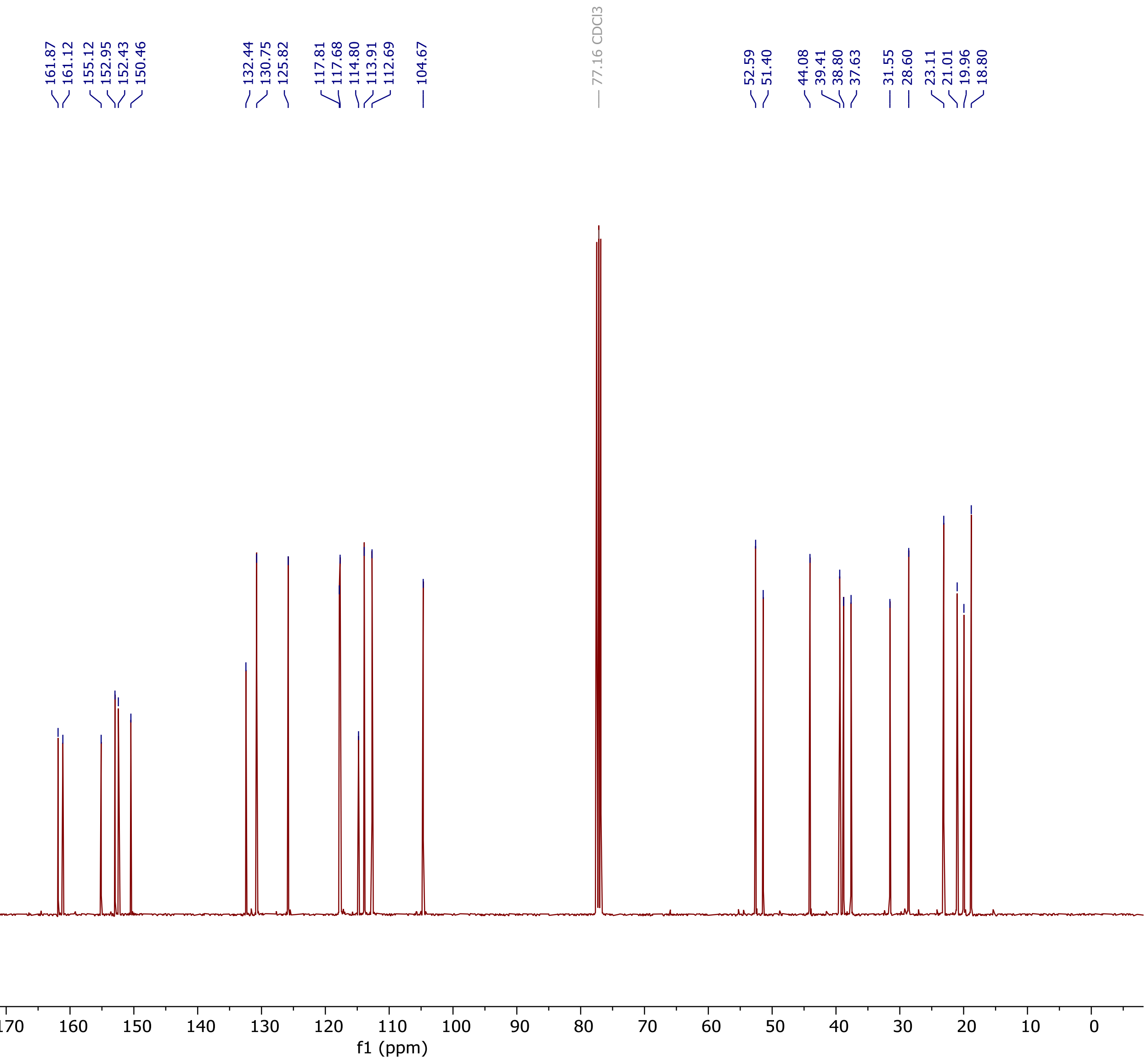

41 - ^{1}H NMR (400 MHz, $CDCl_3$)

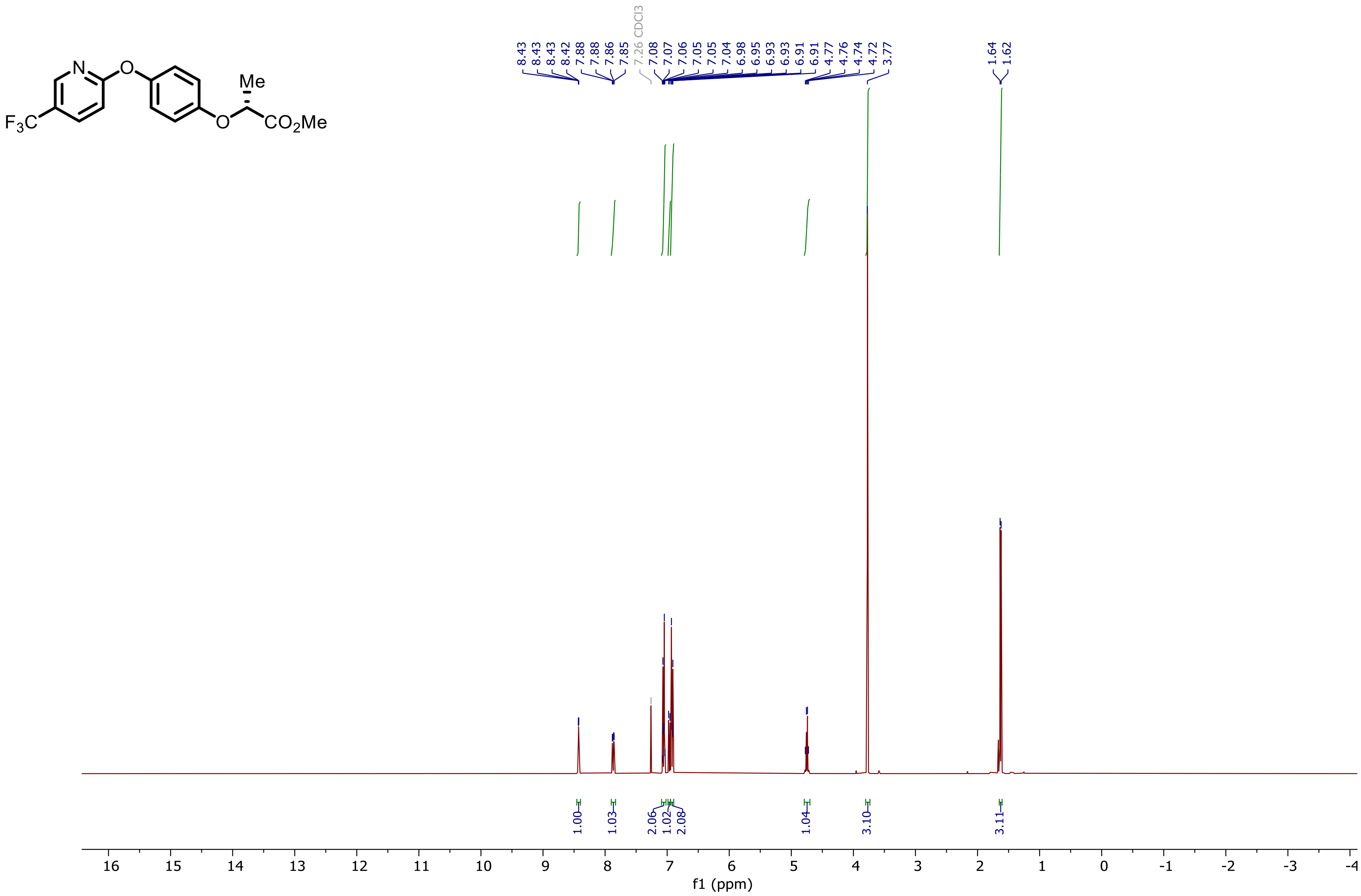

41 - $^{13}C\{^1H\}$ NMR (101 MHz, $CDCl_3$)

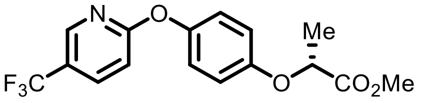

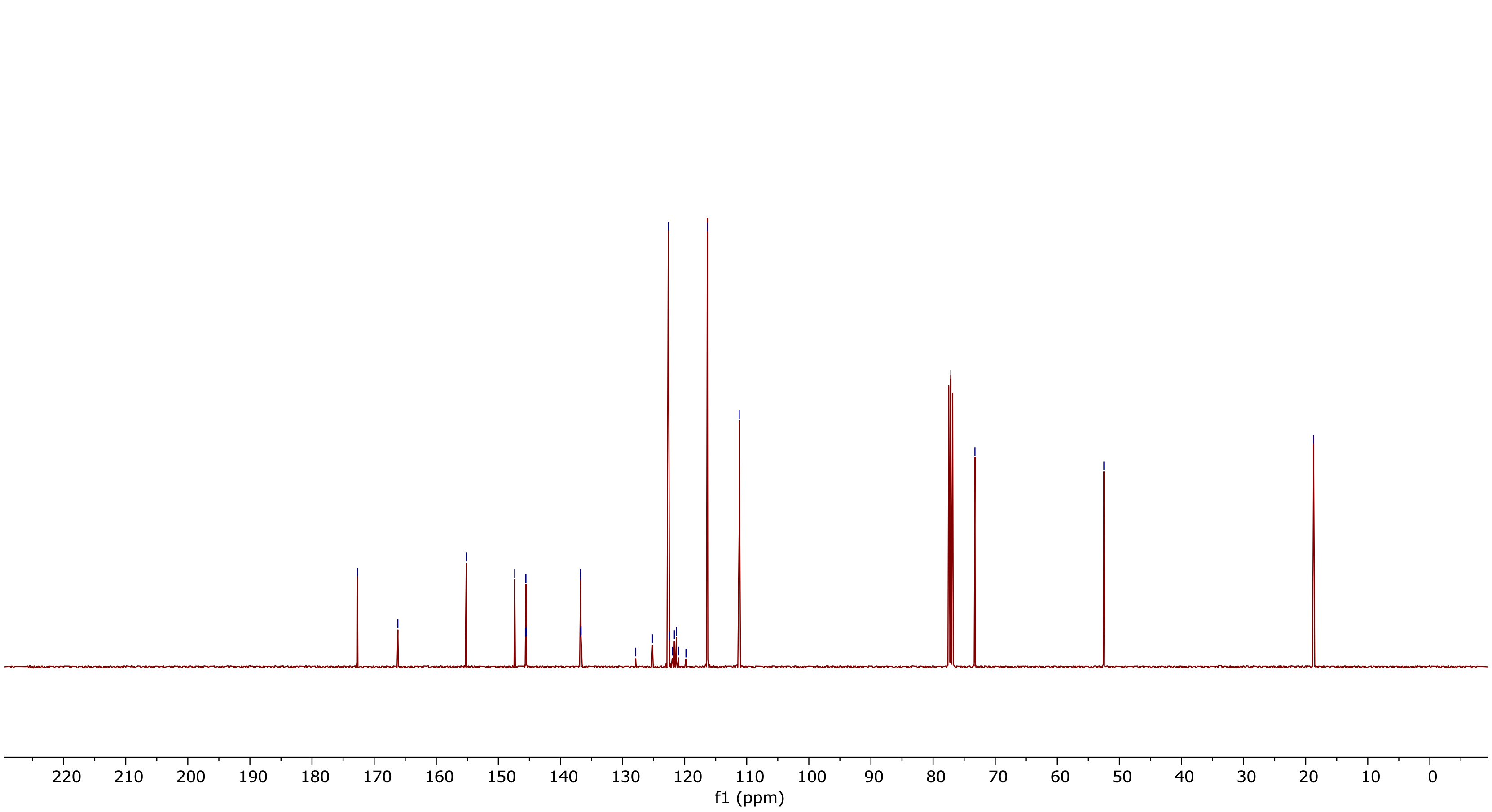

41 - ^{19}F NMR (376 MHz, $CDCl_3$)

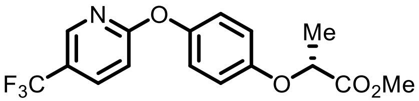

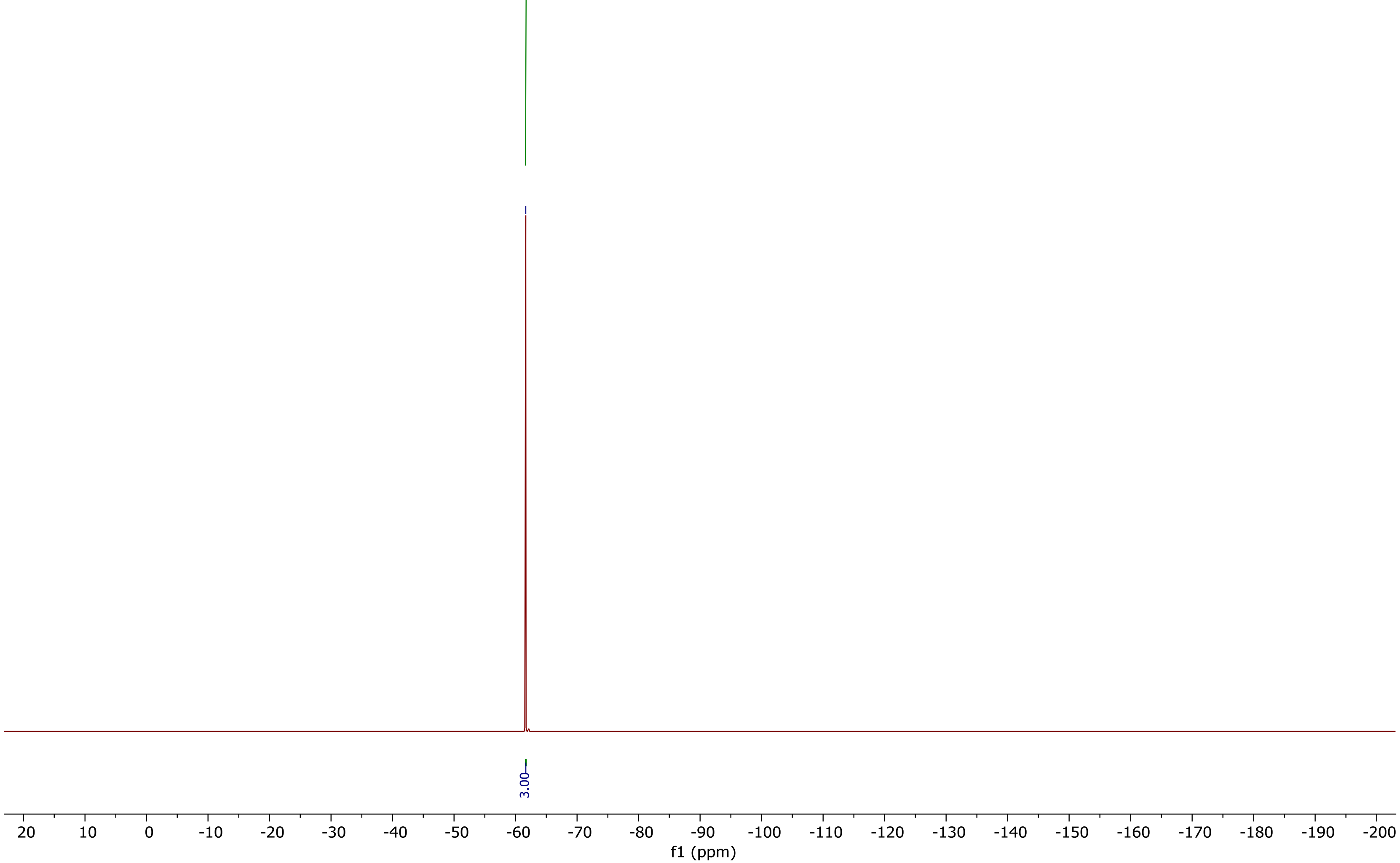

Supplement: Supplementary file 1 [file ja5c13469_si_001.pdf]
